# Fructose-containing food sources and blood pressure: A systematic review and meta-analysis of controlled feeding trials

**DOI:** 10.1371/journal.pone.0264802

**Published:** 2023-08-15

**Authors:** Qi Liu, Laura Chiavaroli, Sabrina Ayoub-Charette, Amna Ahmed, Tauseef A. Khan, Fei Au-Yeung, Danielle Lee, Annette Cheung, Andreea Zurbau, Vivian L. Choo, Sonia Blanco Mejia, Russell J. de Souza, Thomas M. S. Wolever, Lawrence A. Leiter, Cyril W. C. Kendall, David J. A. Jenkins, John L. Sievenpiper

**Affiliations:** 1 Department of Nutritional Sciences, Temerty Faculty of Medicine, University of Toronto, Toronto, Ontario, Canada; 2 Toronto 3D Knowledge Synthesis and Clinical Trials Unit, Clinical Nutrition and Risk Factor Modification Centre, St. Michael’s Hospital, Toronto, Ontario, Canada; 3 INQUIS Clinical Research Ltd. (formerly GI Labs), Toronto, Ontario, Canada; 4 Department of Family and Community Medicine, University of Toronto, Toronto, Ontario, Canada; 5 Department of Health Research Methods, Evidence, and Impact, Faculty of Health Sciences, McMaster University, Hamilton, Ontario, Canada; 6 Population Health Research Institute, Hamilton Health Sciences Corporation, Hamilton, Ontario, Canada; 7 Division of Endocrinology and Metabolism, Department of Medicine, St. Michael’s Hospital, Toronto, Ontario, Canada; 8 Department of Medicine, Temerty Faculty of Medicine, University of Toronto, Toronto, Ontario, Canada; 9 Li Ka Shing Knowledge Institute, St. Michael’s Hospital, Toronto, Ontario, Canada; 10 College of Pharmacy and Nutrition, University of Saskatchewan, Saskatoon, Saskatchewan, Canada; Brigham and Women’s Hospital and Harvard Medical School, UNITED STATES

## Abstract

Whether food source or energy mediates the effect of fructose-containing sugars on blood pressure (BP) is unclear. We conducted a systematic review and meta-analysis of the effect of different food sources of fructose-containing sugars at different levels of energy control on BP. We searched MEDLINE, Embase and the Cochrane Library through June 2021 for controlled trials ≥7-days. We prespecified 4 trial designs: substitution (energy matched substitution of sugars); addition (excess energy from sugars added); subtraction (excess energy from sugars subtracted); and *ad libitum* (energy from sugars freely replaced). Outcomes were systolic and diastolic BP. Independent reviewers extracted data. GRADE assessed the certainty of evidence. We included 93 reports (147 trial comparisons, N = 5,213) assessing 12 different food sources across 4 energy control levels in adults with and without hypertension or at risk for hypertension. Total fructose-containing sugars had no effect in substitution, subtraction, or *ad libitum* trials but decreased systolic and diastolic BP in addition trials (P<0.05). There was evidence of interaction/influence by food source: fruit and 100% fruit juice decreased and mixed sources (with sugar-sweetened beverages [SSBs]) increased BP in addition trials and the removal of SSBs (linear dose response gradient) and mixed sources (with SSBs) decreased BP in subtraction trials. The certainty of evidence was generally moderate. Food source and energy control appear to mediate the effect of fructose-containing sugars on BP. The evidence provides a good indication that fruit and 100% fruit juice at low doses (up to or less than the public health threshold of ~10% E) lead to small, but important reductions in BP, while the addition of excess energy of mixed sources (with SSBs) at high doses (up to 23%) leads to moderate increases and their removal or the removal of SSBs alone (up to ~20% E) leads to small, but important decreases in BP in adults with and without hypertension or at risk for hypertension.

**Trial registration:** Clinicaltrials.gov: NCT02716870.

## Introduction

Cardiovascular diseases are the leading cause of death globally, claiming the lives of 17.9 million people each year, or 32% of deaths worldwide [1]. Chronically elevated blood pressure (BP), also known as hypertension, is a leading modifiable risk factor for these diseases [2]. The global prevalence of hypertension has been increasing in the past decades [3]. A purported contributor to this increase in hypertension is the intake of sugars with a particular focus on fructose since it is thought to act as an unregulated substrate for de novo lipogenesis, bypassing negative feedback control, unlike its glucose counterpart [4–10]. Fructose has been implicated as a driver of hypertension as well as the development of obesity and diabetes [9, 11, 12], both of which further contribute to hypertension and its downstream complications [13]. The proposed mechanisms are supported by animal models, ecological studies, and some fructose over-feeding trials using levels of exposure well beyond population intakes, which have limited application to human health [14].

Emerging evidence indicates that the effect of fructose-containing sugars (sucrose, high-fructose corn syrup, fructose) is mediated by the food source in which they are consumed and the level of energy control. Systematic reviews and meta-analyses have shown that fructose-containing sugars providing excess energy, especially as sugar-sweetened beverages (SSBs), are associated with increased risk of obesity [15, 16], metabolic syndrome [17], diabetes [18], gout [19], and cardiovascular disease [20] in prospective cohort studies and increases in related intermediate cardiometabolic risk factors in controlled feeding trials [21–25]; whereas these adverse signals are not seen for other important food sources or total fructose in energy-matched substitution for other carbohydrates which would replace them as part of food reformulation strategies to reduce these sugars.

We have conducted a series of systematic reviews and meta-analyses to address these questions in relation to hypertension. We recently showed that in prospective cohort studies, SSBs are associated with increased risk of hypertension [26, 27], whereas total fructose intake at moderate doses [28] or other important foods sources [26] do not show the same relationship with higher intakes of fruit and yogurt, and moderate intake of 100% fruit juice even associated with lower risk of hypertension [26]. We also showed that in controlled feeding trials, total fructose (independent of food sources) in energy-matched substitution for other carbohydrates does not increase blood pressure (BP) and even decreases diastolic blood pressure and mean arterial pressure [29]. To complete this series, we aim to clarify the extent to which food source mediates the effect of fructose-containing sugars on blood pressure in controlled clinical trials. Therefore, we conducted a systematic review and meta-analysis of controlled trials of the effect of different food sources of fructose-containing sugars at different levels of energy control on blood pressure and assessed the certainty of evidence using GRADE.

## Methods

We followed the Cochrane Handbook for Systematic Reviews of Interventions (version 6.3) [30] for the conduct of our systematic review and meta-analysis and reported our results following the Preferred Reporting Items for Systematic Reviews and Meta-Analyses (PRISMA) guidelines [31] (S1 Table in S1 File). The study protocol is registered at ClinicalTrials.gov (NCT02716870). All relevant data are within the manuscript and its S1 File.

### Data sources and search strategy

We conducted a systematic search in MEDLINE, Embase, and the Cochrane Central Register of Controlled Studies databases through June 28^th^, 2021. S2, S3 Tables in S1 File present the search strategy based on the PICOTS framework; there were no language restrictions. Validated filters from the McMaster University Health Information Research Unit were applied to limit the database search to controlled studies only [32]. Manual searches of the reference lists of included studies complemented the systematic search.

### Study selection

We included randomized and non-randomized controlled feeding trials in humans of all health backgrounds and ages, with intervention periods ≥7 days investigating the effect of orally consumed fructose-containing sugars from various food sources compared with control diets free of or lower in fructose-containing sugars on systolic or diastolic blood pressure. We excluded studies of liquid meal replacement interventions and studies of interventions or comparators of rare sugars that contain fructose (e.g., isomaltulose, melezitose, or turanose) or were low calorie epimers of fructose (e.g., allulose, tagatose, sorbose). Reports were initially excluded based on review of their titles and abstracts by a single reviewer. Those reports that remained were then excluded based on review of the full text reports by at least 2 reviewers (QL, SA-C, DL, LC, FAY, AC, XQ, AA), leaving the final set of reports to be included in our syntheses. We prespecified four study design levels based on energy control: 1) ’substitution’ or isocaloric trials, in which energy from the food sources of fructose-containing sugars was substituted for other non-fructose-containing macronutrients under energy matched conditions; 2) ’addition’ trials, in which excess energy from the food sources of fructose-containing sugars was added to the background diet compared to the same diet alone without the excess energy (with or without the use of non-nutritive/low-calorie sweeteners to match sweetness); 3) ’subtraction’ trials, in which energy from the food sources of fructose-containing sugars was subtracted from background diets compared with the original background diets through displacement by water or low-calorie sweeteners or elimination altogether; and 4) *’ad libitum’* trials, in which energy from the food sources of fructose-containing sugars was freely replaced (that is, the participants could eat as much or as little as they like within reasonable limits e.g. intake required to be between 75 and 125% of predicted daily energy requirements) with other non-fructose-containing macronutrients without any strict control of either the study foods or the background diets, allowing for free replacement of energy. In reports containing more than one eligible trial comparison (a unique comparison between an intervention and control group in a trial), we included each available trial comparison.

### Data extraction

At least two reviewers (QL, SA-C, LC, AA) independently extracted data from eligible studies. Relevant information included food source of fructose-containing sugars, number of participants, setting, participant health status, study design, level of feeding control, randomization, comparator, fructose-containing sugars type, macronutrient profile of the diets, follow-up duration, energy balance, funding source and outcome data. S4 Table in S1 File shows the definitions for the different food sources of fructose-containing sugars. Authors were contacted for missing outcome data when it was indicated that blood pressure was measured but not reported. In the absence of outcome data and inability to obtain the original data from authors, values were extracted from figures using Plot Digitizer [33] where available.

### Risk of bias assessment

Included studies were assessed for risk of bias independently and in duplicate by ≥2 reviewers (QL, SA-C, LC, AA) using the Cochrane Risk of Bias Tool [30]. Assessment was done across six domains of bias (sequence generation, allocation concealment, blinding, incomplete outcome data, selective outcome reporting and other). Risk of bias for each domain was assessed as either “low” (proper methods taken to reduce bias), “high” (improper methods creating bias) or “unclear” (insufficient information provided). The “other” domain applied only to crossover trials; “high” risk of bias was given when there was no washout between interventions, otherwise the trial was rated as “low”. Reviewer discrepancies were resolved by consensus or arbitration by the senior author (JLS).

### Outcomes

The outcomes were systolic and diastolic blood pressure. Mean differences (MDs) between the intervention and control arm and their standard errors (SEs) were extracted for each eligible trial comparison (each unique comparison between an intervention and control group in a trial). If unavailable, they were derived from available data using published formulas [30]. Mean pairwise difference in change-from-baseline values were preferred over end values. When median data was provided, they were converted to mean data with corresponding variances using methods developed by Luo et al. (2018) [34] and Wan et al. (2014) [35]. When no variance data was available, the standard deviation (SD) was borrowed from a trial similar in size, participants and nature of intervention [36].

### Data syntheses and analyses

We used Stata software, version 16.1 (StataCorp, College Station, TX, USA) for all analyses. As our primary research question was to assess the effect of different food sources of fructose-containing sugars at different energy control levels, we performed separate pairwise meta-analyses for each of the four prespecified designs by energy control level (substitution, addition, subtraction and *ad libitum* trials) and assessed the interaction between food sources of fructose-containing sugars within each energy control level using the Cochrane Handbook’s recommended standard Q-test for subgroup differences (significance at *P*<0.10) [37–39].

The principal effect measures were the mean pair-wise differences in change-from-baseline (or alternatively, end differences) between the food sources of fructose-containing sugars arm and the comparator arm (significance at *P*<0.05). Data were analyzed using the generic inverse variance method with DerSimonian and Laird random-effects model [30, 40]. A fixed effects model was used when <5 trial comparisons were available [41]. Paired analyses were applied to all crossover trials with the use of a within-individual correlation coefficient between treatment of 0.5 as described by Elbourne et al. to calculate SEs [42–44]. Data were expressed as MDs with 95% confidence intervals (CIs). To mitigate a unit-of-analysis error, when arms of trials with multiple intervention or control arms were used more than once, the corresponding sample size was divided by the number of times it was used for calculation of the standard error [30].

Heterogeneity was assessed by visual inspection of the forest plots and using the Cochrane Q statistic and quantified using the *I*^*2*^ statistic [30]. We considered an *I*^*2*^≥50% and *P*_Q_<0.10 as evidence of substantial heterogeneity [30]. Sources of heterogeneity were explored by sensitivity analyses, including individual trial influence, altering pairwise comparison correlation coefficient and subgroup analyses. The influence analysis systematically removed each trial comparison from the meta-analysis with recalculation of the summary effect estimate. A trial whose removal explained the heterogeneity or changed the significance, direction, or magnitude (by more than the minimally important difference (MID) for systolic/diastolic BP, 2mmHg [45]) of the effect was considered an influential trial. To determine whether the overall results were robust to the use of different correlation coefficients in crossover trials, we also conducted sensitivity analyses using correlation coefficients of 0.25 and 0.75. If ≥10 trials were available [38, 46] we conduced subgroup analyses to explore sources of heterogeneity using meta-regression (significance at *P*<0.05). *A priori* subgroup analyses were conducted by participant health status, age, anti-hypertensive medication use, randomization, energy balance, baseline outcome levels, fructose sugars type, comparator type, study design, follow-up, feeding control, fructose-containing sugars dose, and funding. *Post-hoc* subgroup analyses were conducted by sugars regulatory designation and type of imputation done for deriving variances. Meta-regression analyses were used to assess the significance of each subgroup categorically and, when applicable, continuously.

If ≥6 trial comparisons were available [47], we assessed the effect modification by dose using meta-regression with linear and non-linear (using restricted cubic splines) dose-response gradients (significance at *P*<0.05). Non-linear dose-response gradients were estimated using restricted cubic splines with default knots set at the 15th, 50th and 85th percentiles of the exposure variable as recommended by Harrell [48] and assessed for departure from linearity. We also assessed non-linear dose-response threshold effects with three prespecified spline knots at public health thresholds of 5% [49, 50], 10% [50, 51], and 25% [52] total energy (%E).

If ≥10 trials were available, we assessed for small-study effects (publication bias) by visual inspection of contour-enhanced funnel plots and formal testing with Egger’s [53] and Begg’s [54] tests (significance at *P*<0.10) [55]. If there was evidence of publication bias, we adjusted for funnel plot asymmetry and assessed for small-study effects by imputing the missing trial data using the Duval and Tweedie trim-and-fill method [56].

### Certainty of the evidence

The certainty of the evidence was assessed using the Grading of Recommendations, Assessment, Development, and Evaluation (GRADE) approach and software (GRADEpro GDT, McMaster University and Evidence Prime Inc., Hamilton, Canada) [57]. The assessments were conducted by at least two independent reviewers (QL, SA-C, LC, AA) and discrepancies were resolved by consensus or arbitration by the senior author (JLS). The evidence was rated as high, moderate, low, or very low certainty. The included controlled trials were initially rated as high certainty by default and then downgraded or upgraded based on pre-specified criteria. Reasons for downgrading the evidence included risk of bias (assessed by the Cochrane Risk of Bias Tool [58]), inconsistency (substantial unexplained inter-study heterogeneity, *I*^*2*^>50% and *P*<0.10), indirectness (presence of factors that limit the generalizability of the results), imprecision (the 95% CI for effect estimates overlap the MID [2mmHg for systolic BP and diastolic BP] for benefit or harm), and publication bias (significant evidence of small study effects). The reason for upgrading the evidence was presence of a significant dose-response gradient [59–64]. We then used the MIDs to assess the importance of the magnitude of our point estimates using the effect size categories according to GRADE guidance [57, 65–67] as follows: large effect (≥5x MID); moderate effect (≥2x MID); small important effect (≥1x MID); and trivial/unimportant effect (< 1 MID).

## Results

### Search results

**Fig 1:**
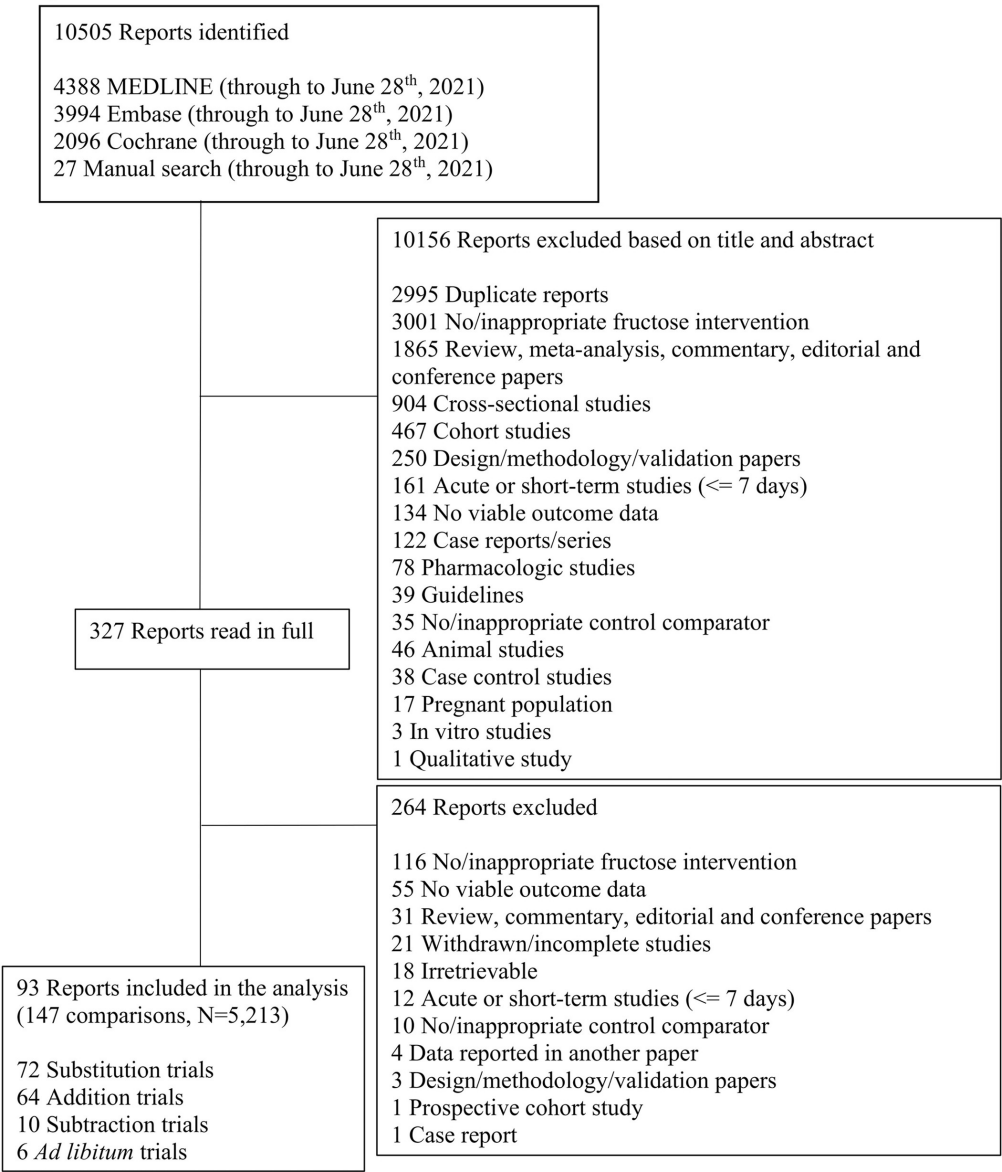
Flow of literature for the effect of food sources of fructose-containing sugars and blood pressure.

Fig 1 shows the flow of the literature. We retrieved 10,505 reports from databases and manual searches, 10,156 of which were excluded based on the title or abstract. Of the 327 reports reviewed in full text, 93 reports of controlled feeding trials (147 trial comparisons, N = 5,213) met the eligibility criteria [68–160]. These trials included twelve different food sources of fructose-containing sugars (SSBs; sweetened dairy; sweetened dairy alternative [soy]; 100% fruit juice; fruit; dried fruit; mixed fruit forms; sweetened cereal grains and bars; sweets and desserts; added nutritive [caloric] sweetener; and mixed sources [with SSBs], and mixed sources [without SSBs]) across four energy control levels: substitution (72 trial comparisons); addition (64 trial comparisons); subtraction (10 trial comparisons); and *ad libitum* (6 trial comparisons). The mixed sources (without SSBs) food category includes those trials in which the intervention included more than one of the food sources, excluding SSBs (e.g., sweets and desserts and fruits). Out of the fifteen authors who were contacted for missing blood pressure outcome data, nine responded and provided unpublished data [71, 85, 86, 106, 117, 124, 136, 141, 156].

### Trial characteristics

Table 1 and S5 Table in S1 File show the trial characteristics. Trial sizes ranged from a median of 11 participants (range 9–50) in *ad libitum* trials to 109 participants (range 12–240) in subtraction trials. Participants were a mix of adults with and without hypertension or at risk for hypertension (overweight/obesity or diabetes). There were approximately equal ratios of both sexes for substitution and addition trials with slightly more women than men, but there were proportionally more females for subtraction and *ad libitum* trials. Most participants were young adults with ages ranging from a median of 28 (range: 22–42) years in subtraction trials to 40 (range: 8–63) years in substitution trials. Most trials were conducted in an outpatient setting (80–100%), performed in American and European countries, and were parallel in design (53% in substitution, 64% in addition, 90% in subtraction, and 17% in *ad libitum* trials). Feeding control was mostly supplemented for substitution (65%), addition (84%), and subtraction (50%) trials, and metabolic for most *ad libitum* (67%) trials. Most studies were randomized (75%-100%), except *ad libitum* trials (33%). The dose of fructose-containing sugars ranged from a median of 6.7% (1–26%) in addition trials to 23% (23–23%) of total energy intake in *ad libitum* trials. The follow-up duration ranged from a median of two weeks (range 2–6.5 weeks) in *ad libitum* trials to 26.1 weeks (15.5–35.8 weeks) in subtraction trials. Most trials were funded by industry sources for substitution trials (65%), agency sources for addition trials (government, not-for-profit health agency, or university sources) (63%), agency and industry sources for *ad libitum* trials (83%), and subtraction trials were mostly funded by agency and industry (30%) or failed to reported funding sources (30%). The comparators for substitution trials were mostly mixed comparator (23/72, 32%) followed by starch (19/72, 26%) and glucose (17/72, 24%), diet alone for addition trials (43/64, 67%), non-nutritive sweetener for subtraction (6/10, 60%) and starch for *ad libitum* trials (3/6, 50%). The main food sources for substitution trials were SSBs (16/72, 22%) and mixed sources (with SSBs) (13/72, 18%). The main food sources for addition trials were SSBs (17/64, 27%) followed by 100% fruit juice (16/64, 25%) and fruit (16/64, 25%). SSBs were also the main food source for subtraction (8/10, 80%) and mixed sources (with SSBs) in *ad libitum* trials (6/6, 100%).

**Table 1:** Summary of trial characteristics*.

		
Trial characteristics	Substitution trials	Addition trials	Subtraction trials	*Ad libitum* trials
Trial comparisons (N)	72	64	10	6
Participants (median N (range))	29.5 (6.0–239.0)	30.0 (10.0–112.0)	109 (12–240)	11 (9–50)
Health status (N trials)	HMW = 32, OW/OB = 17, PreDM/DM = 11, MetS = 3, NAFLD = 3, HTN/PHTN = 2, Higher CVD Risk = 1, Osteoarthritis = 1, CKD = 2	HMW = 31, OW/OB = 8, PreDM/DM = 6, MetS = 4, HTN/PHTN = 6, PCOS = 3, Hemodialysis = 1, HIV = 3	HMW = 5, OW/OB = 5	HMW = 5, OW/OB = 1
Sex ratio (% male:female)^a^	43:57	43:57	11:89	26:74
Age (years; median (range))^a^	40 (8–63)	39.5 (22–67)	28 (22–42)	38.5 (31–45)
Age category ratio (%; adult: children: mixed)^a^	95:5:0	100: 0: 0	100: 0: 0	100: 0: 0
Antihypertensive medication use (%; No: yes: unclear: mixed)^a^	38:44:18	54:2:16:28	17:0:83:0	100: 0: 0: 0
Country (N trials)	Australia = 1, Denmark = 1, Finland = 7, Germany = 6, Greece = 3, India = 2, Iran = 3, Mexico = 2, Netherlands = 1, Poland = 2, Spain = 1, Sweden = 2, Switzerland = 9, UK = 3, USA = 29	Brazil = 1, Canada = 1, China = 1, Denmark = 9, Finland = 1, Germany = 1, India = 2, Indonesia = 1, Iran = 9, Israel = 1, Italy = 1, Malaysia = 3, Norway = 3, Pakistan = 1, Serbia = 1, Switzerland = 5, Thailand = 3, USA = 20	Mexico = 3, Switzerland = 2, UK = 1, USA = 4	Denmark = 4, UK = 2
Setting ratio (%; inpatients: outpatients: inpatients/outpatients)	3:80:17	6:83:11	0: 100: 0	0: 100: 0
Baseline SBP (mmHg; median (range))^b^	124.8 (104.2–166.7)	122 (111.4–153)	112.5 (101–127.4)	126 (116–136)
Baseline DBP (mmHg; median (range))^b^	74.3 (60–106.8)	75.2 (63.1–98.5)	71.3 (66.6–81.6)	78.3 (71.5–85)
Trial design ratio (%; crossover: parallel)	47:53	36:64	10:90	83:17
Feeding control ratio (%; met: sup: DA: met/sup: supp/DA)	14:65:15:6:0	5:84:0:11:0	0:50:20:0:30	67:33:0:0:0
Randomization ratio (%; yes: no: partial)^c^	78: 22: 0	75:25:0	100:0:0	33:67:0
Fructose-containing sugar dose (% of total energy intake; median (range))	13.7 (1.2–58)	6.7 (1–26)	14.4 (3–20)	23 (23–23)
Follow-up duration (median N (range) of weeks)	6 (1–52)	6 (2–52)	26 (16–36)	2 (2–7)
Funding sources (%; A: I: A+I: NR)	14:65:15:6	63:5:29:3	20:20:30:30	0:17:83:0
Fructose-containing sugar type (N trials)	Fructose = 17, sucrose = 16, honey = 1, fruit = 20, HFCS = 6, mixed type = 12	Fructose = 7, sucrose = 10, honey = 3, fruit = 39, HFCS = 3, mixed type = 2	Sucrose = 6, HFCS = 4	Sucrose = 4, mixed type = 2
Sugar regulatory designation (N trials)	Naturally occurring = 20, added = 41, mixed designations = 11	Naturally occurring = 39, added = 24, mixed designations = 1	Added = 10	Added = 4, mixed designations = 2
Comparator (N trials)	Starch = 19, glucose = 17, fat = 6, lactose = 4, protein = 1, nuts = 2, mixed comparators = 23	NNS = 12, water = 9, diet alone = 43	NNS = 6, water = 4	Starch = 3, fat = 2, mixed comparators = 1
Food sources of fructose-containing sugars (N trials)	SSB = 16, sweetened dairy = 3, sweetened dairy alternative (soy) = 1, fruit = 11, dried fruit = 8, mixed fruit forms = 1, sweetened cereal grains and bars = 1, sweets and desserts = 8, added nutritive (caloric) sweeteners = 7, mixed sources (with SSBs) = 13, mixed sources (without SSBs) = 3	SSB = 17, 100%FJ = 16, fruit = 16, dried fruit = 6, sweetened cereal grains and bars = 2, sweets and desserts = 3, added nutritive (caloric) sweetener = 3, mixed sources (with SSBs) = 1	SSB = 8, mixed source (with SSBs) = 2	Mixed sources (with SSBs) = 6

A = agency; A+I = agency and industry; CKD = chronic kidney disease, CVD = cardiovascular disease, DA = dietary advice; DBP = diastolic blood pressure; DM = diabetes mellitus, FJ = fruit juice; HFCS = high fructose corn syrup; HIV = human immunodeficiency virus, HMW = healthy mixed weight; HTN = hypertensive; I = industry; met = metabolic; MetS = metabolic syndrome; NAFLD = non-alcoholic fatty liver disease, NNS = non-nutritive sweetener; OB = obese; OW = overweight; PCOS = poly-cystic ovarian syndrome, PHTN = pre-hypertensive; SBP = systolic blood pressure; SSBs = sugar sweetened beverages; supp = supplemented; UK = United Kingdom; USA = United States of America.

* Values are rounded to nearest whole number except for baseline SBP outcomes.

^a^ Based on trials which report data.

^b^ Based on trial comparisons that reported baseline data (N = 1 trial missing baseline SBP and DBP substitution trials, N = 5 trials missing baseline SPB and DBP addition trials, and N = 4 trials missing baseline SBP and DBP *ad libitum* trials).

^c^ Partial randomization was assigned to a trial comparison which randomized only selected participants.

### Risk of bias

S1-S4 Figs in S1 File show a summary of the risk of bias (ROB) assessments of the included trials. Most trials were assessed as having unclear ROB in random sequence generation (75/147, 51%), allocation concealment (94/147, 64%), and incomplete outcome data domains (91/147, 62%) due to poor reporting, while most were low ROB in blinding (88/147, 60%) and selective outcome reporting (84/147, 57%) domains. Most cross-over trials were assessed as having high ROB in the “other” (carry-over effects) domain (32/62, 52%). Few studies were assessed as having high ROB, for random sequence generation (39/147, 27%), allocation concealment (24/147, 16%), blinding of participants and personnel (6/147, 4%), incomplete outcome data (1/147, 0.6%), selective outcome reporting (5/147, 3%), and other (carry-over effects) (32/147, 22%) ROB domains, respectively. Thus, there was no overall serious ROB in any trial comparisons except for in *ad libitum* trials for diastolic BP where 4 of the 6 trials had high ROB for sequence generation and allocation concealment (due to not being randomized).

### Systolic blood pressure

**Fig 2:**
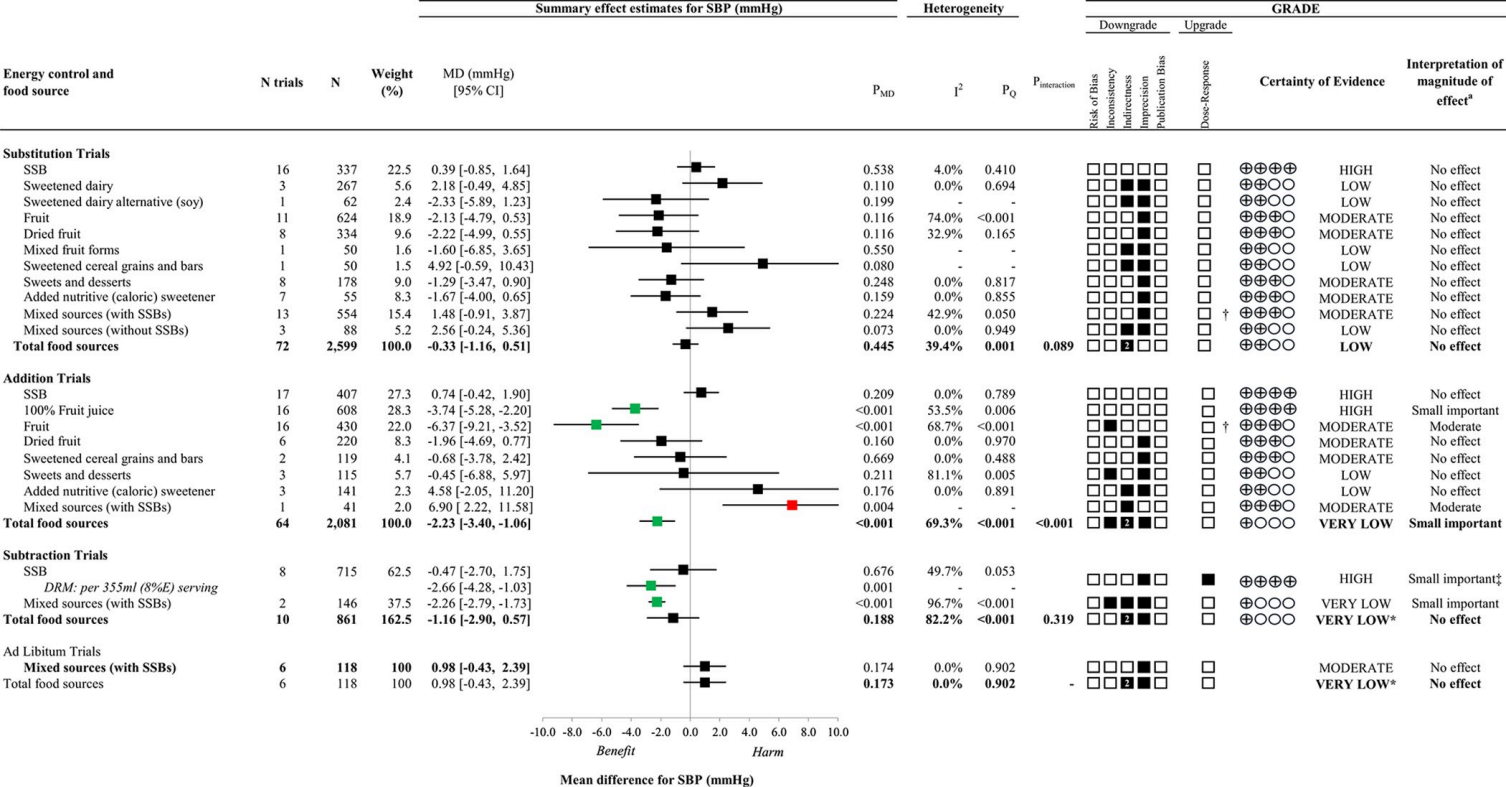
Summary plot for the effect of important food sources of fructose-containing sugars on systolic blood pressure (SBP). Data are weighted mean differences (95% confidence intervals). The bolded lines present the effect estimates for total fructose-containing sugars on SBP at each of the 4 levels of energy control. Where there was significant interaction or influence by food source, effect estimates for each individual food source are presented. Analyses were conducted by generic, inverse variance random effects models (at least five trials available) or fixed effects models (fewer than five trials available). Between-study heterogeneity was assessed by the Cochran Q statistic, where P_Q_<0.100 is considered statistically significant, and quantified by the *I*^*2*^ statistic, where *I*^*2*^≥50% is considered evidence of substantial heterogeneity. The Grading of Recommendations, Assessment, Development and Evaluation (GRADE) of randomized controlled trials are rated as "High" certainty of evidence and can be downgraded by five domains and upgraded by one domain. The white squares represent no downgrades, while filled black squares indicate a single downgrade or upgrades for each outcome, and the black square with a white “2” indicates a double downgrade for each outcome. CI = confidence interval; DRM, dose response model; GRADE = Grading of Recommendations, Assessment, Development and Evaluation; MD = mean difference; N = number; SSB = sugar-sweetened beverage; SBP = systolic blood pressure. ^a^ For the interpretation of the magnitude, we used the MIDs to assess the importance of magnitude of our point estimate using the effect size categories according to new GRADE guidance. * Where there was a significant interaction by food source (in substitution and addition trials), or influence by food source (in subtraction and *ad libitum* trials where SSBs and/or Mixed sources (with SSBs) were the sole food sources), we performed the GRADE analysis for each individual food source. †Not upgraded for dose-response (see S8 Table in S1 File for details). ‡The interpretation of the magnitude of the effect was based on the inverse linear dose-response gradient (see S8 Table in S1 File for details).

Fig 2 presents an overall summary of the effects of different food sources of fructose-containing sugars on systolic BP at four levels of energy control. S5-S8 Figs in S1 File present the individual forest plots for each level of energy control. Total fructose-containing sugars resulted in a reduction in systolic BP for addition trials (64 trials; MD: -2.23mmHg; 95% CI: -3.40, -1.06mmHg, *P*_MD_<0.001; substantial heterogeneity, *I*^*2*^ = 69.3%, *P*_Q_<0.001), whereas there was no effect in substitution trials (72 trials; MD: -0.33mmHg; 95% CI: -1.16, 0.51mmHg; *P*_MD_ = 0.445; no substantial heterogeneity, *I*^*2*^ = 39.4%, *P*_Q_ = 0.001), subtraction trials (10 trials; MD: -1.16mmHg; 95% CI: -2.90, 0.57mmHg; *P*_MD_ = 0.188; substantial heterogeneity, *I*^*2*^ = 82.2%, *P*_Q_<0.001), or *ad libitum* trials (6 trials; MD: 0.98mmHg; 95% CI: -0.43, 2.39mmHg; *P*_MD_ = 0.173; no heterogeneity, *I*^*2*^ = 0.0%, *P*_Q_ = 0.902).

An interaction by food source was detected in the substitution trials (*P* = 0.089), although none of the food sources showed an effect on systolic BP. An interaction by food source was also detected in addition trials (*P*<0.001): mixed sources (with SSBs) resulted in an increase in systolic BP (1 trial; MD: 6.90mmHg; 95% CI: 2.22, 11.58; *P*_MD_ = 0.004), while 100% fruit juice resulted in a reduction in systolic BP (16 trials; MD: -3.74mmHg; 95% CI: -5.28, -2.20mmHg; *P*_MD_<0.001; substantial heterogeneity, *I*^*2*^ = 53.5%, *P*_Q_ = 0.006) and fruit resulted in a reduction in systolic BP (16 trials; MD: -6.37mmHg; 95% CI: -9.21, -3.52mmHg; *P*_MD_<0.001; substantial heterogeneity, *I*^*2*^ = 68.7%, *P*_Q_<0.001), whereas no other food sources showed an effect on systolic BP with variable directions of effect. Although we were unable to assess interaction by food source for systolic BP in subtraction and *ad libitum* trials, there was evidence of influence by food source on systolic BP. In subtraction trials, the removal of mixed sources (with SSBs) resulted in a reduction in systolic BP (2 trials; MD: -2.26mmHg; 95% CI: -2.79, -1.76mmHg; *P*_MD_<0.001; substantial heterogeneity, *I*^*2*^ = 96.7%, *P*_Q_<0.001). The lack of effect on systolic BP was specific to a single food source (SSBs) in *ad libitum* trials.

### Diastolic blood pressure

**Fig 3:**
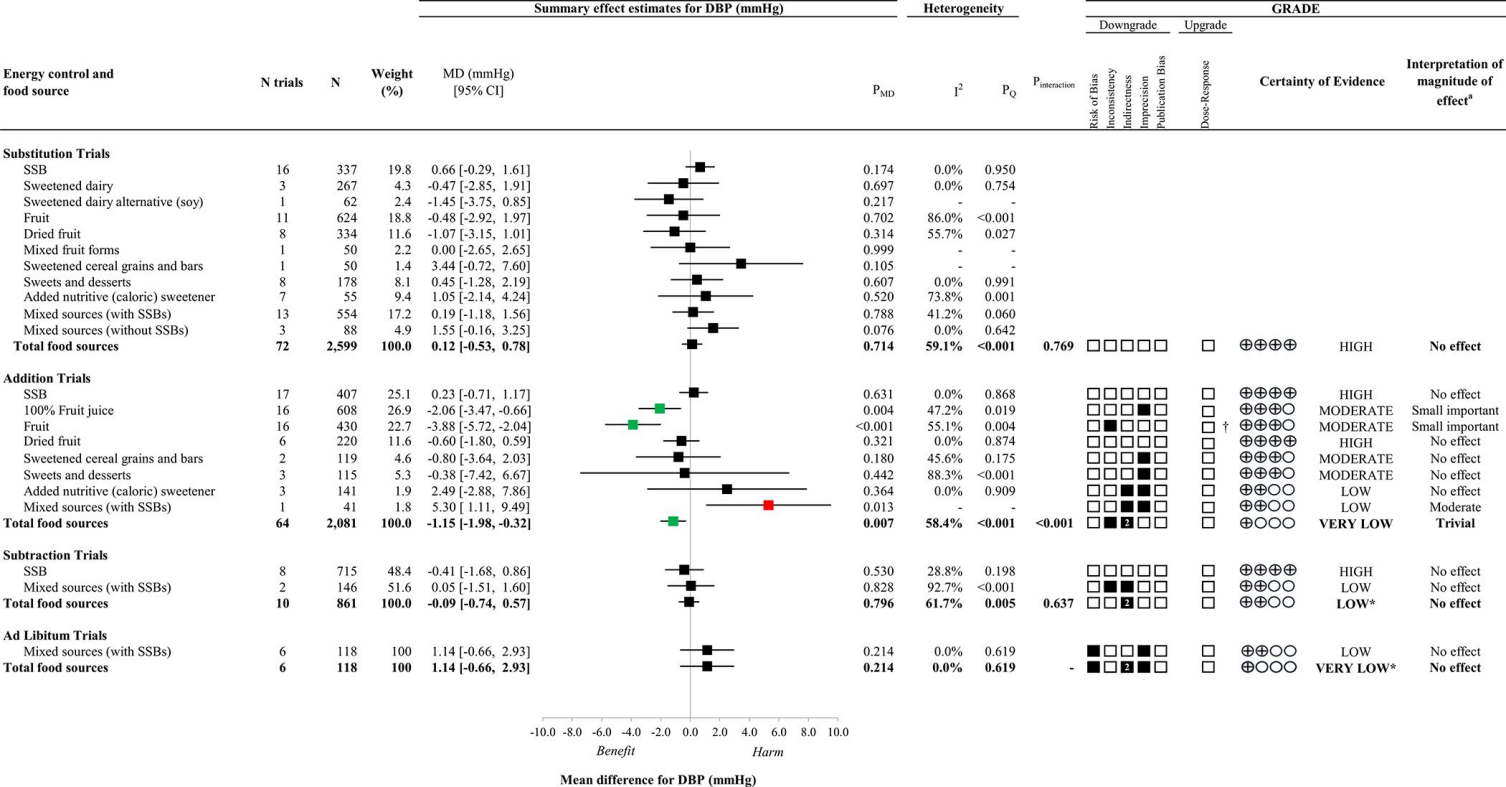
Summary plot for the effect of important food sources of fructose-containing sugars on diastolic blood pressure (DBP). Data are weighted mean differences (95% confidence intervals). The bolded lines present the effect estimates for total fructose-containing sugars on DBP at each of the 4 levels of energy control. Where there was significant interaction or influence by food source, effect estimates for each individual food source are presented. Analyses conducted by generic, inverse variance random effects models (at least five trials available) or fixed effects models (fewer than five trials available). Between-study heterogeneity was assessed by the Cochran Q statistic, where P_Q_<0.100 is considered statistically significant, and quantified by the *I*^*2*^ statistic, where *I*^*2*^≥50% is considered evidence of substantial heterogeneity. The Grading of Recommendations, Assessment, Development and Evaluation (GRADE) of randomized controlled trials are rated as "High" certainty of evidence and can be downgraded by five domains and upgraded by one domain. The white squares represent no downgrades, while filled black squares indicate a single downgrade or upgrades for each outcome, and the black square with a white “2” indicates a double downgrade for each outcome. CI = confidence interval; DBP = diastolic blood pressure; GRADE = Grading of Recommendations, Assessment, Development and Evaluation; MD = mean difference; N = number; SSB = sugar-sweetened beverage. ^a^ For the interpretation of the magnitude, we used the MIDs to assess the importance of magnitude of our point estimate using the effect size categories according to new GRADE guidance. *Where there was a significant interaction by food source (in substitution and addition trials) or influence by food source (in subtraction and *ad libitum* trials where SSBs and/or Mixed sources (with SSBs) were the sole food sources), we performed the GRADE analysis for each individual food source. †Not upgraded for dose-response (see S8 Table in S1 File for details).

Fig 3 presents an overall summary of the effects of different food sources of fructose-containing sugars on diastolic BP at four levels of energy control. S9-S12 Figs in S1 File present the individual forest plots for each level of energy control. Total fructose-containing sugars resulted in a reduction in diastolic BP in addition trials (64 trials; MD: -1.15mmHg; 95% CI: -1.98, -0.32mmHg, *P*_MD_ = 0.007; substantial heterogeneity, *I*^*2*^ = 58.4%, *P*_Q_<0.001), whereas there was no effect in substitution trials (72 trials; MD: 0.12mmHg; 95% CI: -0.53, 0.78mmHg; *P*_MD_ = 0.714; substantial heterogeneity, *I*^*2*^ = 59.1%, *P*_Q_<0.001), subtraction trials (10 trials; MD: -0.09mmHg; 95% CI: -0.74, 0.57mmHg; *P*_MD_ = 0.796; substantial heterogeneity, *I*^*2*^ = 61.7%, *P*_Q_ = 0.005), or *ad libitum* trials (6 trials; MD: 1.14mmHg; 95% CI: -0.66, 2.93mmHg; *P*_MD_ = 0.214; no heterogeneity, *I*^*2*^ = 0.0%, *P*_Q_ = 0.619).

An interaction by food source was detected in addition trials (*P*<0.001) for diastolic BP: mixed sources (with SSBs) resulted in an increase in diastolic BP (1 trial; MD: 5.30mmHg; 95% CI: 1.11, 9.49mmHg; *P*_MD_ = 0.013), while 100% fruit juice resulted in a reduction in diastolic BP (16 trials; MD: -2.06mmHg; 95% CI: -3.47, -0.66mmHg; *P*_MD_ = 0.004; no substantial heterogeneity, *I*^*2*^ = 47.2%, *P*_Q_ = 0.019) and fruit resulted in a reduction in diastolic BP (16 trials; MD: -3.88mmHg; 95% CI: -5.72, -2.04mmHg; *P*_MD_<0.001; substantial heterogeneity, *I*^*2*^ = 55.1%, *P*_Q_ = 0.004), whereas no other food sources showed an effect on diastolic BP with variable directions of effect. There was no evidence of an interaction by food source in substitution trials or subtraction trials, and we were unable to assess an interaction by food source in *ad libitum* trials for diastolic BP. There was, however, evidence of influence by food source on diastolic BP in subtraction and *ad libitum* trials. The lack of effect on diastolic BP was specific to a single food source (mixed sources [with SSBs]) in *ad libitum* trials and 2 food sources (SSBs and mixed sources [with SSBs]) in subtraction trials.

### Sensitivity analyses

S13-S20 Figs in S1 File present the influence analyses for total fructose-containing sugars at the 4 levels of energy control. Removal of single trial comparisons resulted in a gain of significance for the reduction in systolic BP in subtraction trials [119] and increase in diastolic BP in *ad libitum* trials [145] and provided a partial explanation of the evidence of substantial heterogeneity for systolic BP in subtraction trials (Vazquez-Duran et al. 2016 water arm [114]) and diastolic BP in substitution trials [110].

S21-S46 Figs in S1 File present the influence analyses for individual food sources for those analyses that showed evidence of an interaction or influence by food source. Removal of single trial comparisons resulted in a gain of significance for the reduction in systolic BP for fruit [142] and dried fruit [137] in substitution trials and for the reduction in systolic BP for the removal of SSBs [119] and resulted in a change in the direction of effect (Vazquez-Duran et al. 2016 NSBs arm [114]) for diastolic BP in subtraction trials. Removal of single trial comparisons also provided a partial explanation of the evidence of substantial heterogeneity for the effect of fruit on systolic BP in substitution trials [110], 100% fruit juice on systolic BP in addition trials [118, 140] and sweets and desserts on diastolic BP in addition trials [101].

S6 Table in S1 File shows sensitivity analyses for the different correlation coefficients (0.25 and 0.75) used in paired analyses of crossover trials. The use of these different correlation coefficients did not alter the direction, magnitude, or significance of the effect or evidence for heterogeneity for any outcomes across food sources and levels of energy control. The exceptions were the use of a correlation of 0.75 which led to a significant reduction for the effect of added nutritive (caloric) sweetener on systolic BP in substitution trials (7 trials; MD: -1.85mmHg; 95% CI: -3.56, -0.13mmHg; *P*_MD_ = 0.035, I^2^ = 0.00%, *P*_Q_ = 0.587) and a significant increase for the effect of mixed sources (without SSBs) on systolic BP in substitution trials (3 trials; MD: 2.63mmHg; 95% CI: 0.87, 4.40mmHg; *P*_MD_ = 0.004, I^2^ = 0.00%, *P*_Q_ = 0.889) and of 0.25 which led to a partial explanation of heterogeneity for the effect of fruit on diastolic BP in addition trials (16 trials; MD: -4.14mmHg; 95% CI: -5.97, -2.31; *P*_MD_<0.001, I^2^ = 47.45%, *P*_Q_ = 0.018).

### Subgroup analyses

S47-S58 Figs in S1 File present the subgroup analyses for the effect of important food sources of fructose-containing sugars, where there were at least 10 trial comparisons, on blood pressure. In the 4 analyses for systolic BP and diastolic BP in either substitution or addition trials, there was significant effect modification of the following in 3 of the 4 analyses: fructose-containing sugars type (fruit decreased BP while others generally showed no effect or harmful effect), regulatory designation (naturally occurring decreased BP while others generally showed no effect or harmful effect) and dose (<10%E decreased blood pressure, while other generally showed no effect); the following in 2 of 4 analyses: baseline blood pressure, medication use (mixed decrease, others no effect) and follow up (≤8wks decrease, >8wks no effect); the following in 1 of the 4 analyses: age, funding, type of mean difference, risk of bias categories, feeding control, and comparator. For subtraction trials, there was significant effect modification on systolic BP and diastolic BP in at least one of the following: design, follow-up, and risk of bias (selective outcome reporting).

S59-S76 Figs in S1 File present the subgroup analyses for the effect of individual food sources, where there was a significant interaction or influence by food source and at least 10 trial comparisons, on blood pressure. For systolic BP in substitution trials, SSBs, fruit and mixed sources (with SSBs), and in addition analyses for both systolic BP and diastolic BP, SSBs, 100% fruit juice and fruit were analyzed. In all analyses except for SSBs on systolic BP in addition trials, there was significant effect modification in at least one of the following: funding, age, health status, baseline systolic BP or diastolic BP, follow-up, risk of bias categories, antihypertensive medication use, design, and dose.

S77-S83 Figs in S1 File present the continuous meta regression analyses for the effect of important food sources of fructose-containing sugars on blood pressure. In both substitution and addition trials, baseline systolic BP or diastolic BP were significant. Where baseline blood pressure level increased, important food sources of fructose-containing sugars had a greater reduction in blood pressure. There was also a significant continuous meta regression for age in substitution trials on diastolic BP, and in addition trials on systolic BP where with increasing age, important food sources of fructose-containing sugars had a greater reduction in blood pressure.

S83-S91 Figs in S1 File present the continuous meta regression analyses for the effect of individual food sources including SSBs, fruit, mixed sources (with SSBs) for systolic BP in substitution trials, and SSBs, 100% fruit juice and fruit in addition analyses for both systolic and diastolic BP. In substitution trials for systolic BP, there was a negative association for baseline SBP for both fruit and mixed sources (with SSBs), for follow-up for fruit and for dose for mixed sources (with SSBs). In addition trials for systolic and diastolic BP, there was a positive association for dose for fruit, while there was a negative association for follow-up for 100% fruit juice for systolic BP only.

### Dose response analyses

S92-S128 Figs in S1 File present linear and non-linear dose-response analyses. For substitution trials, although there was no dose response of total fructose-containing sugars, when assessed by food sources, there was an inverse linear dose response for the effect of mixed sources (with SSBs) on systolic BP (*P* = 0.009, S100 Fig, panel F in S1 File) where greater reductions were seen with larger doses, however this was no longer significant with the removal of one trial with a dose of nearly 60%E (*P* = 0.204) [111]. For addition trials, there was a significant positive linear dose response gradient for the effect of total fructose-containing sugars on systolic BP and diastolic BP (coef_linear_:0.34; 95% CI, 0.21 to 0.47, *P*_linear_<0.001, S93 Fig in S1 File; coef_linear_: 0.24; 95% CI, 0.13 to 0.34, *P*_linear_<0.001, S97 Fig in S1 File, respectively), and when assessed by food source, there was a positive linear dose response for the effect of fruit on systolic BP and diastolic BP in addition trials (16 trials, coef_linear_: 4.8mmHg; 95% CI: 1.2 to 8.5, *P*_linear_ = 0.009, S101 Fig panel D in S1 File, and coef_linear_: 3.4mmHg; 95% CI: 0.8 to 6.1, *P*_linear_ = 0.012, per serving (5%E) of fruit, S103 Fig, panel D in S1 File, respectively) where greater reductions were seen with smaller doses; however reductions were seen in systolic and diastolic BP across the entire dose response range. There was a dose response at the public health threshold of 25% E for the effect of SSBs sugars dose on systolic BP in addition trials (17 trials, *P* = 0.038, S119 Fig in S1 File); however, there was only 1 trial with a dose >25% E. For subtraction trials, there was a significant linear dose response for the effect of removal of SSBs on systolic BP (8 trials, coef_linear_: -2.66mmHg; 95% CI: -4.28 to -1.03, *P*_linear_ = 0.001, per serving (355ml, 8%E) of SSB, S102 Fig in S1 File) where greater reductions were seen with greater removal of SSBs. There was also a non-linear u-shaped dose response for the effect of the removal of SSBs on diastolic BP in subtraction trials (8 trials, *P* = 0.003, S104 Fig in S1 File) and at the public threshold of 5% and 10% of energy (8 trials, *P* = 0.031, for each, S128 Fig in S1 File).

### Publication bias

S129-S144 Figs in S1 File present the publication bias assessments for all outcomes.

There was evidence of funnel plot asymmetry for the effect of SSBs on diastolic BP in addition trials (Egger’s test *P* = 0.036). Adjustment for funnel plot asymmetry with the imputation of 13 missing trials by The Duval and Tweedie trim-and-fill method, however, did not alter the magnitude or significance of the effect, suggesting that there was no meaningful influence of publication bias on the results. Publication bias could not be assessed in *ad libitum* comparisons, or for certain food sources where there was significant interaction by food source, as there were <10 trials available for these analyses.

### GRADE assessment

Figs 2, 3 and S7, S8 Tables in S1 File present the GRADE assessments. To support GRADE assessments, additional post-hoc subgroup analyses to further explore indirectness (S145-S159 Figs in S1 File) on blood pressure methodology, fasted state, and outcome consideration were conducted and did not show any evidence of effect modifications either in analyses for total fructose containing sugars or across food sources (where applicable). The certainty of evidence for the effect of total fructose-containing sugars on systolic BP was low in substitution trials (no effect) and very low in addition (moderate reduction), subtraction (no effect), and *ad libitum* (no effect) trials, owing to double downgrades for indirectness across the 4 levels of energy control and single downgrades for inconsistency in addition trials and imprecision in addition, subtraction and *ad libitum* trials. The certainty of evidence for the effect of total fructose-containing sugars on diastolic BP was high for substitution trials (no effect), low for subtraction (no effect) and very low for addition (trivial reduction) and *ad libitum* trials (no effect), owing to double downgrades for indirectness in addition, subtraction and *ad libitum* trials and single downgrades for risk of bias (*ad libitum*), inconsistency (addition) or imprecision (*ad libitum*).

Because there was a significant interaction by food source in substitution trials for systolic BP and addition trials for systolic BP and diastolic BP and influence of individual food sources in subtraction trials and *ad libitum* trials, we assessed the certainty of evidence for individual food sources in these analyses. The certainty of evidence was moderate for the effect of mixed sources (with SSBs) on systolic BP in addition trials (moderate increase), owing to a single downgrade for indirectness, high for the effect of 100% fruit juice in addition trials (small important reduction) and moderate for the effect of fruit in addition trials (moderate reduction), owing to a single downgrade for inconsistency. The certainty of evidence was low for the effect of mixed sources (with SSBs) on diastolic BP in addition trials (moderate increase), owing to downgrades for indirectness and imprecision, and moderate for the effects of 100% fruit juice in addition trials (small important reduction) and fruit in addition trials (small important reduction), owing to single downgrades for imprecision and inconsistency, respectively. The certainty of the evidence was high for the effect of removing SSBs on systolic BP in subtraction trials (small important reduction) owing to a downgrade for imprecision and an upgrade for linear dose response, and very low for the effects of removing mixed sources (with SSBs) on systolic BP in subtraction trials (small important reduction). The certainty of evidence varied from high to low for all other food sources owing to downgrades for risk of bias, inconsistency, indirectness, and/or imprecision.

## Discussion

Our systematic review and meta-analysis of 93 reports (147 trial comparisons) in 5,213 in participants with and without hypertension or at risk for hypertension assessed the effects of 12 different food sources (SSBs; sweetened dairy; sweetened dairy alternative [soy]; 100% fruit juice; fruit; dried fruit; mixed fruit forms; sweetened cereal grains and bars; sweets and desserts; added nutritive [caloric] sweetener; mixed sources [with SSBs] and mixed sources [without SSBs]) with a median dose of 7% (1–26%) to 23% (23–23%) of total energy across four different levels of energy control over median follow-up of 2–26 weeks. Total fructose-containing sugars led to small important reductions of 2.2mmHg in systolic BP and trivial reductions of 1.15mmHg in diastolic BP in addition trials. There was no effect at the other levels of energy control in substitution, addition, subtraction, or *ad libitum* trials. There was an interaction or influence by food source. 100% fruit juice and fruit at lower doses that did not exceed the public health threshold of ~10% E led to small important reductions (-3.7mmHg in systolic and -2.06mmHg in diastolic BP) and moderate reductions (-6.37mmHg in systolic and -3.88mmHg in diastolic BP), respectively, in addition trials. On the other hand, mixed sources (with SSBs) at high doses providing 23% excess energy led to moderate increases of 6.9 mmHg in systolic BP and 5.3 mmHg in diastolic BP in addition trials and the removal of a median 5% of excess energy from mixed sources (with SSBs) led to small important reductions (-2.2mmHg) in systolic BP in subtraction trials. The removal of a median of 15% excess energy from SSBs also led to small important reductions in systolic BP with evidence of a linear dose response gradient (removal of one serving (355ml, 8%E) of SSBs was associated with a systolic BP reduction of 2.2 mmHg) in subtraction trials. Other important food sources of fructose-containing sugars showed no effect on BP.

### Findings in relation to the literature

Our findings are in agreement with a previous systematic review and meta-analysis by Ha et al. [29] in that they did not demonstrate an adverse effect of total fructose-containing sugars on blood pressure. However, Ha et al. [29] demonstrated beneficial reductions on diastolic BP and mean arterial pressure when fructose-containing sugars were substituted for other carbohydrates in energy-matched conditions and no effects on systolic BP or diastolic BP in addition trials, whereas our findings showed no effect on either systolic BP or diastolic BP in energy-matched conditions, but a benefit when consumed as excess calories, which was driven by the effects of fruit and fruit juice. The discrepancy in findings may be the result of the much large number of trials included in the present analysis (72 substitution and 64 addition trials compared to 13 and 2, respectively). Further, another systematic review and meta-analysis found that substitutions of free sugars, as defined by the WHO [49], for complex carbohydrates had no significant effects on blood pressure [161], agreeing with our findings.

Although the moderate harmful effects of mixed sources (with SSBs) on systolic BP and diastolic BP in addition trials were based on only one trial, we also found significant beneficial dose response effects of removing SSBs from the diet in subtraction trials on systolic BP (based on 8 trials). However, we did not find a significant effect of SSBs alone on either systolic BP or diastolic BP in addition trials. The lack of effect of SSBs in addition trials compared to the observed linear dose response effect in subtraction trials of SSBs may be due to the shorter duration (median 3-wk, range 2-26wk vs median 26-wk, range 1-52wk, respectively) and more normal body weight of the study participants (14/17 trials in normal mixed weight adults, 3/17 overweight or obese vs 3/8 in normal mixed weight adults, 3/8 overweight or obese and 2/8 overweight/obese with either high or low liver fat, respectively). The findings of the potential harmful effect of SSBs are supported by previous literature connecting SSB intake and hypertension and high blood pressure. Our previous systematic review and meta-analysis of prospective cohorts found a significant 10% dose response increase in risk of incident hypertension per 1-serving (355 mL)/day SSB intake, with a wide coverage of cohorts [26]. Other recent systematic reviews and meta-analyses have identified similar associations between SSBs intake and incident hypertension [162–164]. Most proposed mechanisms from analyses of both observational and clinical trials link SSBs consumed as excess calories directly to other adverse health conditions like type 2 diabetes [165, 166] and weight gain [15], which secondarily raise blood pressure. However, SSBs consumed as excess energy has been shown to increase blood uric acid [22], which is a commonly proposed pathway by which fructose intake may lead to hypertension [4–6, 8–10].

We found that fructose-containing sugar doses of up to 10% daily energy intake consumed in the form of fruit demonstrated beneficial linear dose response effects on blood pressure across the dose response range, with greater reductions seen with smaller doses and diminishing as doses increase. This dose relationship has been seen with cohort studies examining fruit and vegetable intake and incident hypertension [167–169]. The evidence of beneficial effects of fruit concur with recent systematic reviews and meta-analyses, including our previous one that showed a significant dose response of a 6% decrease in risk of incident hypertension per 240g serving /day of fruit intake [26]. One popular hypothesis of the beneficial effects of fruit consumption pertains to their high flavonoid contents [170]. These flavonoids have been shown to decrease endothelial dysfunction, inflammation and oxidative stress—important factors in the development of hypertension–as well as decrease blood pressure [171–175]. Various fruits are also rich in potassium with small amounts of magnesium and calcium, the combination of which has been shown to decrease blood pressure [176]. Ensuring adequate potassium intake, via the inclusion of fruits in the diet, can help individuals achieve a lower blood pressure, particularly in individuals with hypertension [177].

The beneficial effect of 100% fruit juice we found on blood pressure is likely due to the same nutrients and bio-compounds as found in fruit though the lack of fiber, the intake of which is also linked to lower blood pressure [178, 179], may explain our findings of 100% fruit juice’s smaller reductions on blood pressure compared to fruit. However, it is worth noting that our prior systematic review and meta-analysis found a U-shaped dose-dependent relationship between incident hypertension and 100% fruit juice intake, with maximum protection shown around 0.5–1 serving (50–150 mL)/day intake and suggestion of harmful associations over 200 mL/day intake. A recent systematic review and meta-analysis found a similar dose-dependent relationship of 100% fruit juice intake in prospective cohorts with cardiovascular event risk with the benefit-harm threshold at 170 mL/day [180]. The authors attributed the benefit of 100% fruit juice to the significant decrease in both systolic BP and diastolic BP they also identified in randomized controlled trials [180]. Thus, there may be potential for harmful effects on blood pressure at higher doses of 100% fruit juice intake that is not captured in our current analysis.

Furthermore, the predominant subgroup effects we identified (i.e., significant effect modification by: fructose-containing sugar type, regulatory designation, and dose) in substitution and addition trials seem to support the food sources interactions observed, as they capture the significant effects of fruit and 100% fruit juice as natural sources of sugars where the source is fruit, and these studies generally were lower doses compared to studies of other food sources.

Historically, the link between fructose and hypertension comes from animal models where animals were fed high fructose diets (>60%E), mainly as free fructose, to induce hypertension and insulin resistance [6]. In addition to the evidence from animal models, there are some human trials demonstrating that acute ingestion of very high doses of fructose or fructose-containing sugars (~12–15%E as a single bolus) increases blood pressure postprandially [181, 182]. The present study only included trials with >1-week intervention, thus was not designed to explore the acute postprandial effects of different food sources of fructose-containing sugars. However, the present study did not demonstrate evidence of harm in the range of 1–52 weeks (median 6-weeks) follow-up period. Evidence from one study provided an exception where mixed sources (with SSBs) consumed at high doses providing 23% excess energy in addition to the habitual diet, led to an increase in blood pressure. The results of this study highlight that the effect of energy and not fructose per se should be considered to be of importance for longer term effect. Furthermore, there is a lack of evidence that fructose consumption is associated with an elevated risk of developing hypertension over the long term (median 18-years), particularly if consumed at less than 10%E [28]. In exploring potential effect modification by follow-up duration in our subgroup analyses, follow-up duration was only significant in the subgroup analysis of the effect of fruit in substitution trials of SBP (trials with >8-weeks follow-up duration showed a significant reduction in SBP, whereas trials with ≤8-weeks showed no statistically significant effect). In continuous analyses, we found a significant negative association for fruit on SBP in substitution trials and 100% fruit juice on SBP in addition trials (where greater follow-up was associated with greater reduction in SBP). Although it appears there is some benefit with longer follow-up, this was not consistent across the food sources analyzed and we were unable to conduct these analyses for most food sources owing to inadequate trial comparisons (<10 trial comparisons).

Since there are metabolic differences between free fructose and glucose, and possibly when bound as the disaccharide sucrose, in our subgroup analyses, we did include an assessment of effect modification by fructose type where we compared trials of sucrose, HFCS and free fructose. In our trials of SSBs, neither in substitution nor addition analyses, did we see significant effect modification by fructose type. This is supported by reviews showing no harmful effects (including on blood pressure and other cardiovascular risk factors) of HFCS, sucrose or fructose alone, when consumed isocalorically (energy matched conditions) [14, 21, 23, 24, 29]. When it comes to food sources, the dose, food matrix, and other aspects within the foods (e.g. bioactives) influence the effect, as we observed reductions in BP for fruit and 100% fruit juice.

Altogether, our results highlight the importance of considering whole foods or dietary patterns, rather than just nutrients (i.e. fructose or fructose-containing sugars), and the energy conditions under which these foods are consumed. There is limited evidence to suggest intakes of food sources of fructose-containing sugars are harmful on blood pressure. The exception where we see a harmful increase in blood pressure is when mixed sources (with SSBs) are consumed as excess calories in addition to the participants habitual diet, in comparison to the control group where participants are consuming only their habitual diet. Conversely, the removal of mixed sources (with SSBs), or of SSBs alone, from the habitual diet, as a reduction in calories, compared to participants still consuming these foods, resulted in a reduction in BP. Therefore, when it comes to mixed sources (with SSBs), the effects on blood pressure are likely mediated by energy and not fructose itself. Most other food sources of fructose-containing sugars showed no effect, except fruit and 100% fruit juice which showed reductions in BP, likely due to the dose of fructose-containing sugars (<10%E), the food matrix, and the contribution of bioactive compounds.

### Strengths and limitations

Our systematic review and meta-analysis has several strengths. First, we conducted a comprehensive and reproducible search and selection process of the literature examining the effect of food sources of fructose-containing sugars on blood pressure. Second, we collated and synthesized the totality of available evidence from a large body (93 studies, 147 trial comparisons, N = 5,213) of controlled intervention studies, which give the greatest protection against bias. Third, we had comprehensive exploration of possible sources of heterogeneity. Fourth, we evaluated the shape and strength of the dose-response relationships. Fifth, we assessed the overall quality of evidence using the GRADE assessment approach.

Our analyses also presented limitations. First, our diastolic BP *ad libitum* analysis was downgraded for serious ROB due to 4 of the 6 trials not being randomized and sensitivity analyses revealing a difference in effect where those 4 trials with high ROB increased diastolic BP whereas the overall pooled effect showed no effect. Second, there was evidence of very serious indirectness resulting in double downgrades in all overall pooled analyses of total fructose-containing sugars for substitution and addition trials, except for diastolic BP in substitution trials, due to significant interaction or influence of food source. Another source of very serious indirectness was the limited number of food sources of fructose-containing sugars available for some analyses. Subtraction and *ad libitum* trials were double downgraded due to having only one or two food sources available (SSBs and/or mixed sources (with SSBs)), limiting the ability to assess differences in food sources, and thus it is unclear whether these effects hold for other important food sources of fructose-containing sugars. The differences in methodologies used to measure blood pressure, whether measurements were taken in the fasted state, and whether blood pressure was considered a primary or secondary outcome are additional potential sources of indirectness. We performed post-hoc subgroup analyses (S145-S159 Figs in S1 File) on blood pressure methodology, fasted state, and outcome consideration and did not find any evidence of effect modifications either in analyses for total fructose containing sugars or across food sources (where applicable), so we did not downgrade for serious indirectness in any of these cases. Third, some analyses (e.g., fruit in addition analyses) were downgraded for serious inconsistency due to substantial unexplained heterogeneity. Lastly, some analyses were downgraded for imprecision due to crossing the prespecified minimally importance difference for harm or benefit as we cannot rule out clinically important benefit and/or harm.

Weighing the strengths and limitations, the certainty of evidence was generally moderate (moderate to low) for the increasing effect of mixed sources (with SSBs) and moderate (moderate to high) for the decreasing effect of 100% fruit juice and fruit in addition trials, high for the decreasing effect of the removal of SSBs in subtraction trials, very low for the decreasing effect of the removal of mixed sources (with SSBs), and moderate (low to high) for the effect of all other comparisons on systolic and diastolic BP.

### Implications

As dietary guidelines shift toward a more food-based approach, our findings may have implications for guiding recommendations on the prevention and management of high blood pressure. Although not all individuals meet the hypertension cut-offs at ≥140 mmHg systolic BP and/or ≥90 mmHg diastolic BP, even prehypertensive individuals (systolic BP of 120–139 mmHg or diastolic BP of 80–90 mmHg) are at significantly higher risk for cardiovascular risks and complications [183–189]. Therefore, there is a need to develop preventative and treatment strategies for hypertension, as well as prehypertension. Our findings demonstrate the importance of focusing on specific foods and the energy conditions under which they are consumed, rather than prescribing limits on total fructose-containing sugars. Currently, guidelines generally recommend adhering to a DASH or Mediterranean diet abundant in fruits, vegetables, whole grains, and plant proteins, and limited in sweets and sugar-sweetened beverages [190–194]. We found that the effect of consuming fruit on systolic BP in addition conditions was beyond the -2 mmHg minimally important benefit for blood pressure, which translates to a 10% lower risk of stroke mortality and 7% lower risk of mortality from other vascular causes [45]. Thus, an emphasis on fresh fruit alongside a limitation on SSBs should be a centerpiece in current dietary guidelines for the prevention and management of high blood pressure. Our research also supports the differentiation between added and natural sugars in dietary guidelines, given that our research indicates benefit on blood pressure from moderate intakes of 100% fruit juice which contains only natural sugars.

## Conclusion

In conclusion, the effect of fructose-containing sugars on blood pressure appears to be mediated by both energy control and food source. The addition of excess energy from mixed sources (with SSBs) at high doses (up to 23%) increases, while the removal of excess energy (up to ~20% E) from SSBs and mixed sources (with SSBs) reduces systolic and diastolic BP, whereas fruit and 100% fruit juice at low doses (up to or less than the public health threshold of ~10% E) reduce systolic and diastolic BP. These effects were not seen for other important food sources of fructose-containing sugars at any level of energy control. Our confidence in the estimates is generally moderate. The available evidence provides a good indication that fruit and 100% fruit juice at low doses lead to small important reductions in BP, while the addition of mixed sources (with SSBs) at high doses leads to moderate increases and their removal or the removal of SSBs alone, leads to small important decreases in this population. The main sources of uncertainty across the analyses were indirectness and imprecision. There remains a need for more high-quality randomized trials assessing a broader variety of food sources of fructose-containing sugars to provide more precise estimates. In the meantime, these findings suggest policy and guideline makers should consider the role of energy and food source for the prevention and management of hypertension and continue to encourage SSBs reduction strategies.

## Supporting information

S1 File (PDF)

## Abbreviations

BP: blood pressure
CI: confidence interval
GRADE: grading of recommendations assessment, development, and evaluation
MD: mean difference
MID: minimally important difference
PICOTS: population, intervention, comparator, outcome, time and study design
PRISMA: Reporting Items for Systematic Reviews and Meta-Analysis
SD: standard deviation; SE, standard error
SSB: sugar-sweetened beverages
