# Supplementary material for: Fructose-containing food sources and blood pressure: A systematic review and meta-analysis of controlled feeding trials
Source: PLoS One. 2023 Aug 15;18(8):e0264802. doi: 10.1371/journal.pone.0264802 (PMC10427023; doi:10.1371/journal.pone.0264802)
Supplement: S1 File — (PDF) [file pone.0264802.s001.pdf]

Supporting information for "Fructose-containing sugars and blood pressure: a systematic review and meta-analysis of controlled feeding trials"

S1 Table. PRISMA Checklist. 10

S2 Table. Search strategy for controlled trials assessing the effect of important food sources of fructose-containing sugars on blood pressure. 12

S3 Table. PICOTS framework of the search strategy. 14

S4 Table. Food source definitions. 15

S5 Table. Trial characteristics. 17

S6 Table. Sensitivity analyses of the use of correlation coefficients of 0.25 and 0.75 for crossover trials in the primary analysis of the effect of important food sources of fructose-containing sugars on systolic and diastolic blood pressure (mmHg) and by important food sources of fructose-containing sugars. 47

S7 Table. GRADE certainty of evidence assessment for the effect of fructose-containing sugars on blood pressure by trial energy control. 50

S8 Table. GRADE certainty of evidence assessment for the effect of fructose-containing sugars on blood pressure acid by important food source of fructose-containing sugars. 53

Figures 60

S1 Fig. Risk of bias proportion graph for the effect of important food sources of fructose-containing sugars on SBP and DBP (mmHg) in substitution trials*. 60

S2 Fig. Risk of bias proportion graph for the effect of important food sources of fructose-containing sugars on SBP and DBP in addition trials*. 61

S3 Fig. Risk of bias proportion graph for the effect of important food sources of fructose-containing sugars on SBP and DBP in subtraction trials*. 62

S4 Fig. Risk of bias proportion graph for the effect of important food sources of fructose-containing sugars on SBP and DBP in *ad libitum* trials*. 63

S5 Fig (1 of 3). Forest plot of controlled trials of the effect of important food sources of fructose-containing sugars on SBP (mmHg) in substitution trials. 64

S5 Fig (2 of 3). Forest plot of controlled trials of the effect of important food sources of fructose-containing sugars on SBP (mmHg) in substitution trials. 65

S5 Fig (3 of 3). Forest plot of controlled trials of the effect of important food sources of fructose-containing sugars on SBP (mmHg) in substitution trials. 66

S6 Fig (1 of 2). Forest plot of controlled trials of the effect of important food sources of fructose-containing sugars on SBP (mmHg) in addition trials. 67

S6 Fig (2 of 2). Forest plot of controlled trials of the effect of important food sources of fructose-containing sugars on SBP (mmHg) in addition trials. 68

S7a Fig. Forest plot of controlled trials of the effect of important food sources of fructose-containing sugars on SBP (mmHg) in subtraction trials. 69

S7b Fig. Forest plot of controlled trials of the effect of mixed sources (with SSBs) on SBP (mmHg) in subtraction trials*. 70

S8 Fig. Forest plot of controlled trials of the effect of important food sources of fructose-containing sugars on SBP (mmHg) in *ad libitum* trials. 71

S9 Fig (1 of 3). Forest plot of controlled trials of the effect of important food sources of fructose-containing sugars on DBP (mmHg) in substitution trials. 72

S9 Fig (2 of 3). Forest plot of controlled trials of the effect of important food sources of fructose-containing sugars on DBP (mmHg) in substitution trials.....73

S9 Fig (3 of 3). Forest plot of controlled trials of the effect of important food sources of fructose-containing sugars on DBP (mmHg) in substitution trials.....74

S10 Fig (1 of 2). Forest plot of controlled trials of the effect of important food sources of fructose-containing sugars on DBP (mmHg) in addition trials.....75

S10 Fig (2 of 2). Forest plot of controlled trials of the effect of important food sources of fructose-containing sugars on DBP (mmHg) in addition trials.....76

S11 Fig. Forest plot of controlled trials of the effect of important food sources of fructose-containing sugars on DBP (mmHg) in subtraction trials. 77

S12 Fig. Forest plot of controlled trials of the effect of important food sources of fructose-containing sugars on DBP (mmHg) in *ad libitum* trials.....78

S13 Fig. Sensitivity analysis of the systematic removal of each trial for the effect of important food sources of fructose-containing sugars on SBP (mmHg) in substitution trials. 79

S14 Fig. Sensitivity analysis of the systematic removal of each trial for the effect of important food sources of fructose-containing sugars on SBP (mmHg) in addition trials. 81

S15 Fig. Sensitivity analysis of the systematic removal of each trial for the effect of important food sources of fructose-containing sugars on SBP (mmHg) in subtraction trials.....83

S16 Fig. Sensitivity analysis of the systematic removal of each trial for the effect of important food sources of fructose-containing sugars on SBP (mmHg) in *ad libitum* trials.....84

S17 Fig. Sensitivity analysis of the systematic removal of each trial for the effect of important food sources of fructose-containing sugars on DBP (mmHg) in substitution trials. 85

S18 Fig. Sensitivity analysis of the systematic removal of each trial for the effect of important food sources of fructose-containing sugars on DBP (mmHg) in addition trials. 87

S19 Fig. Sensitivity analysis of the systematic removal of each trial for the effect of important food sources of fructose-containing sugars on DBP (mmHg) in subtraction trials. 89

S20 Fig. Sensitivity analysis of the systematic removal of each trial for the effect of important food sources of fructose-containing sugars on DBP (mmHg) in *ad libitum* trials.....90

S21 Fig. Sensitivity analysis of the systematic removal of each trial for the effect of SSBs on SBP (mmHg) in substitution trials.....91

S22 Fig. Sensitivity analysis of the systematic removal of each trial for the effect of sweetened dairy on SBP (mmHg) in substitution trials.....92

S23 Fig. Sensitivity analysis of the systematic removal of each trial for the effect of fruit on SBP (mmHg) in substitution trials. 93

S24 Fig. Sensitivity analysis of the systematic removal of each trial for the effect of dried fruit on SBP (mmHg) in substitution trials.....94

S25 Fig. Sensitivity analysis of the systematic removal of each trial for the effect of sweets and desserts on SBP (mmHg) in substitution trials. 95

S26 Fig. Sensitivity analysis of the systematic removal of each trial for the effect of added nutritive (caloric) sweetener on SBP (mmHg) in substitution trials. 96

S27 Fig. Sensitivity analysis of the systematic removal of each trial for the effect of mixed sources (with SSBs) on SBP (mmHg) in substitution trials. 97

S28 Fig. Sensitivity analysis of the systematic removal of each trial for the effect of mixed sources (without SSBs) on SBP (mmHg) in substitution trials. 98

S29 Fig. Sensitivity analysis of the systematic removal of each trial for the effect of SSBs on SBP (mmHg) in addition trials.....99

S30 Fig. Sensitivity analysis of the systematic removal of each trial for the effect of 100% fruit juice on SBP (mmHg) in addition trials. 100

S31 Fig. Sensitivity analysis of the systematic removal of each trial for the effect of fruit on SBP (mmHg) in addition trials. 101

S32 Fig. Sensitivity analysis of the systematic removal of each trial for the effect of dried fruit on SBP (mmHg) in addition trials. 102

S33 Fig. Sensitivity analysis of the systematic removal of each trial for the effect of sweetened cereal grains and bars on SBP (mmHg) in addition trials. 103

S34 Fig. Sensitivity analysis of the systematic removal of each trial for the effect of sweets and desserts on SBP (mmHg) in addition trials. 104

S35 Fig. Sensitivity analysis of the systematic removal of each trial for the effect of added nutritive (caloric) sweetener on SBP (mmHg) in addition trials. 104

S36 Fig. Sensitivity analysis of the systematic removal of each trial for the effect of SSBs on SBP (mmHg) in subtraction trials. 106

S37 Fig. Sensitivity analysis of the systematic removal of each trial for the effect of mixed sources (with SSBs) on SBP (mmHg) in subtraction trials*. 107

S38 Fig. Sensitivity analysis of the systematic removal of each trial for the effect of SSBs on DBP (mmHg) in addition trials. 108

S39 Fig. Sensitivity analysis of the systematic removal of each trial for the effect of 100% fruit juice on DBP (mmHg) in addition trials. 109

S40 Fig. Sensitivity analysis of the systematic removal of each trial for the effect of fruit on DBP (mmHg) in addition trials. 110

S41 Fig. Sensitivity analysis of the systematic removal of each trial for the effect of dried fruit on DBP (mmHg) in addition trials. 111

S42 Fig. Sensitivity analysis of the systematic removal of each trial for the effect of sweetened cereal grains and bars on DBP (mmHg) in addition trials. 112

S43 Fig. Sensitivity analysis of the systematic removal of each trial for the effect of sweets and desserts on DBP (mmHg) in addition trials. 113

S44 Fig. Sensitivity analysis of the systematic removal of each trial for the effect of added nutritive (caloric) sweetener on DBP (mmHg) in addition trials. 114

S45 Fig. Sensitivity analysis of the systematic removal of each trial for the effect of SSBs on DBP (mmHg) in subtraction trials. 115

S46 Fig. Sensitivity analysis of the systematic removal of each trial for the effect of mixed sources (with SSBs) on DBP (mmHg) in subtraction trials. 116

S47 Fig (part 1 of 3). Subgroup analyses for the effect of important food sources of fructose-containing sugars on SBP (mmHg) in substitution trials. 117

S47 Fig (part 2 of 3). Subgroup analyses for the effect of important food sources of fructose-containing sugars on SBP (mmHg) in substitution trials. 119

S47 Fig (part 3 of 3). Subgroup analyses for the effect of important food sources of fructose-containing sugars on SBP (mmHg) in substitution trials. 121

S48 Fig. Risk of bias (using The Cochrane Collaboration Tool) subgroup analysis for the effect of important food sources of fructose-containing sugars on SBP (mmHg) in substitution trials. 123

S49 Fig (part 1 of 3). Subgroup analyses for the effect of important food sources of fructose-containing sugars on SBP (mmHg) in addition trials. 125

S49 Fig (part 2 of 3). Subgroup analyses for the effect of important food sources of fructose-containing sugars on SBP (mmHg) in addition trials. 127

S49 Fig (part 3 of 3). Subgroup analyses for the effect of important food sources of fructose-containing sugars on SBP (mmHg) in addition trials. 129

S50 Fig. Risk of bias (using The Cochrane Collaboration Tool) subgroup analysis for the effect of important food sources of fructose-containing sugars on SBP (mmHg) in addition trials. 131

S51 Fig (part 1 of 3). Subgroup analyses for the effect of important food sources of fructose-containing sugars on SBP (mmHg) in subtraction trials. 133

S51 Fig (part 2 of 3). Subgroup analyses for the effect of important food sources of fructose-containing sugars on SBP (mmHg) in subtraction trials. 134

S51 Fig (part 3 of 3). Subgroup analyses for the effect of important food sources of fructose-containing sugars on SBP (mmHg) in subtraction trials. 135

S52 Fig. Risk of bias (using The Cochrane Collaboration Tool) subgroup analysis for the effect of important food sources of fructose-containing sugars on SBP (mmHg) in subtraction trials. 136

S53 Fig (part 1 of 3). Subgroup analyses for the effect of important food sources of fructose-containing sugars on DBP (mmHg) in substitution trials. 138

S53 Fig (part 2 of 3). Subgroup analyses for the effect of important food sources of fructose-containing sugars on DBP (mmHg) in substitution trials. 140

S53 Fig (part 3 of 3). Subgroup analyses for the effect of important food sources of fructose-containing sugars on DBP (mmHg) in substitution trials. 142

S54 Fig. Risk of bias (using The Cochrane Collaboration Tool) subgroup analysis for the effect of important food sources of fructose-containing sugars on DBP (mmHg) in substitution trials. 143

S55 Fig (part 1 of 3). Subgroup analyses for the effect of important food sources of fructose-containing sugars on DBP (mmHg) in addition trials. 145

S55 Fig (part 2 of 3). Subgroup analyses for the effect of important food sources of fructose-containing sugars on DBP (mmHg) in addition trials. 147

S55 Fig (part 3 of 3). Subgroup analyses for the effect of important food sources of fructose-containing sugars on DBP (mmHg) in addition trials. 149

S56 Fig. Risk of bias (using The Cochrane Collaboration Tool) subgroup analysis for the effect of important food sources of fructose-containing sugars on DBP (mmHg) in addition trials. 150

S57 Fig (part 1 of 3). Subgroup analyses for the effect of important food sources of fructose-containing sugars on DBP (mmHg) in subtraction trials. 152

S57 Fig (part 2 of 3). Subgroup analyses for the effect of important food sources of fructose-containing sugars on DBP (mmHg) in subtraction trials. 153

S57 Fig (part 3 of 3). Subgroup analyses for the effect of important food sources of fructose-containing sugars on DBP (mmHg) in subtraction trials. 154

S58 Fig. Risk of bias (using The Cochrane Collaboration Tool) subgroup analysis for the effect of important food sources of fructose-containing sugars on DBP (mmHg) in subtraction trials. 155

S59 Fig (part 1 of 3). Subgroup analyses for the effect of SSBs on SBP (mmHg) in substitution trials. 156

S59 Fig (part 2 of 3). Subgroup analyses for the effect of SSBs on SBP (mmHg) in substitution trials. 157

S59 Fig (part 3 of 3). Subgroup analyses for the effect of SSBs on SBP (mmHg) in substitution trials. 158

S60 Fig. Risk of bias (using The Cochrane Collaboration Tool) subgroup analysis for the effect of SSBs on SBP (mmHg) in substitution trials. 159

S61 Fig (part 1 of 3). Subgroup analyses for the effect of fruit on SBP (mmHg) in substitution trials. 161

S61 Fig (part 2 of 3). Subgroup analyses for the effect of fruit on SBP (mmHg) in substitution trials. 163

S61 Fig (part 3 of 3). Subgroup analyses for the effect of fruit on SBP (mmHg) in substitution trials. 164

S62 Fig. Risk of bias (using The Cochrane Collaboration Tool) subgroup analysis for the effect of fruit on SBP (mmHg) in substitution trials. 165

S63 Fig (part 1 of 3). Subgroup analyses for the effect of mixed sources (with SSBs) on SBP (mmHg) in substitution trials. 167

S63 Fig (part 2 of 3). Subgroup analyses for the effect of mixed sources (with SSBs) on SBP (mmHg) in substitution trials. 169

S63 Fig (part 3 of 3). Subgroup analyses for the effect of mixed sources (with SSBs) on SBP (mmHg) in substitution trials. 170

S64 Fig. Risk of bias (using The Cochrane Collaboration Tool) subgroup analysis for the effect of mixed sources (with SSBs) on SBP (mmHg) in substitution trials. 171

S65 Fig (part 1 of 3). Subgroup analyses for the effect of SSBs on SBP (mmHg) in addition trials. 172

S65 Fig (part 2 of 3). Subgroup analyses for the effect of SSBs on SBP (mmHg) in addition trials. 173

S65 Fig (part 3 of 3). Subgroup analyses for the effect of SSBs on SBP (mmHg) in addition trials. 174

S66 Fig. Risk of bias (using The Cochrane Collaboration Tool) subgroup analysis for the effect of SSBs on SBP (mmHg) in addition trials. 175

S67 Fig (part 1 of 3). Subgroup analyses for the effect of 100% fruit juice on SBP (mmHg) in addition trials. 177

S67 Fig (part 2 of 3). Subgroup analyses for the effect of 100% fruit juice on SBP (mmHg) in addition trials. 179

S67 Fig (part 3 of 3). Subgroup analyses for the effect of 100% fruit juice on SBP (mmHg) in addition trials. 180

S68 Fig. Risk of bias (using The Cochrane Collaboration Tool) subgroup analysis for the effect of 100% fruit juice on SBP (mmHg) in addition trials. 181

S69 Fig (part 1 of 3). Subgroup analyses for the effect of fruit on SBP (mmHg) in addition trials. 183

S69 Fig (part 2 of 3). Subgroup analyses for the effect of fruit on SBP (mmHg) in addition trials. 185

S69 Fig (part 3 of 3). Subgroup analyses for the effect of fruit on SBP (mmHg) in addition trials. 186

S70 Fig. Risk of bias (using The Cochrane Collaboration Tool) subgroup analysis for the effect of fruit on SBP (mmHg) in addition trials. 187

S71 Fig (part 1 of 3). Subgroup analyses for the effect of SSBs on DBP (mmHg) in addition trials. 189

S71 Fig (part 2 of 3). Subgroup analyses for the effect of SSBs on DBP (mmHg) in addition trials. 190

S71 Fig (part 3 of 3). Subgroup analyses for the effect of SSBs on DBP (mmHg) in addition trials. 191

S72 Fig. Risk of bias (using The Cochrane Collaboration Tool) subgroup analysis for the effect of SSBs on DBP (mmHg) in addition trials. 192

S73 Fig (part 1 of 3). Subgroup analyses for the effect of 100% fruit juice on DBP (mmHg) in addition trials. 194

S73 Fig (part 2 of 3). Subgroup analyses for the effect of 100% fruit juice on DBP (mmHg) in addition trials. 196

S73 Fig (part 3 of 3). Subgroup analyses for the effect of 100% fruit juice on DBP (mmHg) in addition trials. 197

S74 Fig. Risk of bias (using The Cochrane Collaboration Tool) subgroup analysis for the effect of 100% fruit juice on DBP (mmHg) in addition trials. 198

S75 Fig (part 1 of 3). Subgroup analyses for the effect of fruit on DBP (mmHg) in addition trials. 200

S75 Fig (part 2 of 3). Subgroup analyses for the effect of fruit on DBP (mmHg) in addition trials. 202

S75 Fig (part 3 of 3). Subgroup analyses for the effect of fruit on DBP (mmHg) in addition trials. 203

S76 Fig. Risk of bias (using The Cochrane Collaboration Tool) subgroup analysis for the effect of fruit on DBP (mmHg) in addition trials. 204

S77 Fig. Continuous meta-regression analysis for the effect of important food sources of fructose-containing sugars on SBP (mmHg) in substitution trials. 206

S78 Fig. Continuous meta-regression analysis for the effect of important food sources of fructose-containing sugars on SBP (mmHg) in addition trials. 207

S79 Fig. Continuous meta-regression analysis for the effect of important food sources of fructose-containing sugars on SBP (mmHg) in subtraction trials. 208

S80 Fig. Continuous meta-regression analysis for the effect of important food sources of fructose-containing sugars on DBP (mmHg) in substitution trials. 209

S81 Fig. Continuous meta-regression analysis for the effect of important food sources of fructose-containing sugars on DBP (mmHg) in addition trials. 210

S82 Fig. Continuous meta-regression analysis for the effect of important food sources of fructose-containing sugars on DBP (mmHg) in subtraction trials. 211

S83 Fig. Continuous meta-regression analysis for the effect of SSBs on SBP (mmHg) in substitution trials. 212

S84 Fig. Continuous meta-regression analysis for the effect of fruit on SBP (mmHg) in substitution trials. 213

S85 Fig. Continuous meta-regression analysis for the effect of mixed sources (with SSBs) on SBP (mmHg) in substitution trials. 214

S86 Fig. Continuous meta-regression analysis for the effect of SSBs on SBP (mmHg) in addition trials. 215

S87 Fig. Continuous meta-regression analysis for the effect of 100% fruit juice on SBP (mmHg) in addition trials. 216

S88 Fig. Continuous meta-regression analysis for the effect of fruit on SBP (mmHg) in addition trials. 217

S89 Fig. Continuous meta-regression analysis for the effect of SSBs on DBP (mmHg) in addition trials. 218

S90 Fig. Continuous meta-regression analysis for the effect of 100% fruit juice on DBP (mmHg) in addition trials. 219

S91 Fig. Continuous meta-regression analysis for the effect of fruit on DBP (mmHg) in addition trials. 220

S92 Fig. Linear meta-regression analyses for the effect of food sources of fructose-containing sugars dose on SBP (mmHg) in substitution trials. 221

S93 Fig. Linear meta-regression analyses for the effect of food sources of fructose-containing sugars dose on SBP (mmHg) in addition trials. 222

S94 Fig. Linear meta-regression analyses for the effect of food sources of fructose-containing sugars dose on SBP (mmHg) in subtraction trials. 223

S95 Fig. Linear meta-regression analyses for the effect of food sources of fructose-containing sugars dose on SBP (mmHg) in *ad libitum* trials. 224

S96 Fig. Linear meta-regression analyses for the effect of food sources of fructose-containing sugars dose on DBP (mmHg) in substitution trials. 225

S97 Fig. Linear meta-regression analyses for the effect of food sources of fructose-containing sugars dose on DBP (mmHg) in addition trials. 226

S98 Fig. Linear meta-regression analyses for the effect of food sources of fructose-containing sugars dose on DBP (mmHg) in subtraction trials. 227

S99 Fig. Linear meta-regression analyses for the effect of food sources of fructose-containing sugars dose on DBP (mmHg) in *ad libitum* trials. 228

S100 Fig. Linear meta-regression analyses for the effect of individual food sources of fructose-containing sugars dose on SBP (mmHg) in substitution trials. 229

.... 229

.... 229

S101 Fig. Linear meta-regression analyses for the effect of individual food sources of fructose-containing sugars dose on SBP (mmHg) in addition trials. 231

S102 Fig. Linear meta-regression analyses for the effect of SSB dose on SBP (mmHg) in subtraction trials. 232

S103 Fig. Linear meta-regression analyses for the effect of individual food sources of fructose-containing sugars dose on DBP (mmHg) in addition trials. 233

S104 Fig. Linear meta-regression analyses for the effect of individual food sources of fructose-containing sugars dose on DBP (mmHg) in subtraction trials. 234

S105 Fig. Non-linear dose-response analysis using public thresholds of 5, 10, and 25% of energy for the effect of food sources of fructose-containing sugars dose (% of total energy) on SBP (mmHg) in substitution trials. 235

S106 Fig. Non-linear dose-response analysis using public thresholds of 5, 10, and 25% of energy for the effect of food sources of fructose-containing sugars dose (% of total energy) on SBP (mmHg) in addition trials. 236

S107 Fig. Non-linear dose-response analysis using public thresholds of 5 and 10% of energy for the effect of food sources of fructose-containing sugars dose (% of total energy) on SBP (mmHg) in subtraction trials. 237

S108 Fig. Non-linear dose-response analysis using public thresholds of 25% of energy for the effect of food sources of fructose-containing sugars dose (% of total energy) on SBP (mmHg) in *ad libitum* trials. 238

S109 Fig. Non-linear dose-response analysis using public thresholds of 5, 10, and 25% of energy for the effect of food sources of fructose-containing sugars dose (% of total energy) on DBP (mmHg) in substitution trials. 239

S110 Fig. Non-linear dose-response analysis using public thresholds of 5, 10, and 25% of energy for the effect of food sources of fructose-containing sugars dose (% of total energy) on DBP (mmHg) in addition trials. 240

S111 Fig. Non-linear dose-response analysis using public thresholds of 5 and 10% of energy for the effect of food sources of fructose-containing sugars dose (% of total energy) on DBP (mmHg) in subtraction trials. 241

S112 Fig. Non-linear dose-response analysis using public thresholds of 25% of energy for the effect of food sources of fructose-containing sugars dose (% of total energy) on DBP (mmHg) in *ad libitum* trials. 242

S113 Fig. Non-linear dose-response analysis using public threshold of 10% of energy for the effect of SSB sugars dose (% of total energy) on SBP (mmHg) in substitution trials. 243

S114 Fig. Non-linear dose-response analysis using public thresholds of 5 and 10% of energy for the effect of fruit sugars dose (% of total energy) on SBP (mmHg) in substitution trials. 244

S115 Fig. Non-linear dose-response analysis using public thresholds of 5 and 10% of energy for the effect of dried fruit sugars dose (% of total energy) on SBP (mmHg) in substitution trials. 245

S116 Fig. Non-linear dose-response analysis using public thresholds of 10 and 25% of energy for the effect of sweets and desserts sugars dose (% of total energy) on SBP (mmHg) in substitution trials. 246

S117 Fig. Non-linear dose-response analysis using public thresholds of 10% of energy for the effect of added nutritive (caloric) sweetener sugars dose (% of total energy) on SBP (mmHg) in substitution trials. 247

S118 Fig. Non-linear dose-response analysis using public thresholds of 10 and 25% of energy for the effect of mixed sources (with SSBs) sugars dose (% of total energy) on SBP (mmHg) in substitution trials. 248

S119 Fig: Non-linear dose-response analysis using public thresholds of 10 and 25% of energy for the effect of SSB sugars dose (% of total energy) on SBP (mmHg) in addition trials. 249

S120 Fig. Non-linear dose-response analysis using public thresholds of 5 and 10% of energy for the effect of 100% fruit juice sugars dose (% of total energy) on SBP (mmHg) in addition trials.....................250

S121 Fig. Non-linear dose-response analysis using public thresholds of 5 and 10% of energy for the effect of fruit sugars dose (% of total energy) on SBP (mmHg) in addition trials.....................251

S122 Fig. Non-linear dose-response analysis using public thresholds of 5 and 10% of energy for the effect of dried fruit sugars dose (% of total energy) on SBP (mmHg) in addition trials.....................252

S123 Fig. Non-linear dose-response analysis using public thresholds of 5 and 10% of energy for the effect of SSB sugars dose (% of total energy) on SBP (mmHg) in subtraction trials. 253

S124 Fig. Non-linear dose-response analysis using public thresholds of 10 and 25% of energy for the effect of SSB sugars dose (% of total energy) on DBP (mmHg) in addition trials.....................254

S125 Fig. Non-linear dose-response analysis using public thresholds of 5 and 10% of energy for the effect of 100% fruit juice sugars dose (% of total energy) on DBP (mmHg) in addition trials. 255

S126 Fig. Non-linear dose-response analysis using public thresholds of 5 and 10% of energy for the effect of fruit sugars dose (% of total energy) on DBP (mmHg) in addition trials. 256

S127 Fig. Non-linear dose-response analysis using public thresholds of 5 and 10% of energy for the effect of dried fruit sugars dose (% of total energy) on DBP (mmHg) in addition trials. 257

S128 Fig. Non-linear dose-response analysis using public thresholds of 5 and 10% of energy for the effect of SSB sugars dose (% of total energy) on DBP (mmHg) in subtraction trials. 258

S129 Fig. Publication bias funnel plots for the effect of important food sources of fructose-containing sugars on SBP (mmHg) in substitution trials. 259

S130 Fig. Publication bias funnel plots for the effect of important food sources of fructose-containing sugars on SBP (mmHg) in addition trials. 260

S131 Fig. Publication bias funnel plots for the effect of important food sources of fructose-containing sugars on SBP (mmHg) in subtraction trials.....................261

S132 Fig. Publication bias funnel plots for the effect of important food sources of fructose-containing sugars on DBP (mmHg) in substitution trials.....................262

S133 Fig. Publication bias funnel plots for the effect of important food sources of fructose-containing sugars on DBP (mmHg) in addition trials.....................263

S134 Fig. Publication bias funnel plots for the effect of important food sources of fructose-containing sugars on DBP (mmHg) in subtraction trials. 264

S135 Fig. Publication bias funnel plots for the effect of SSBs on SBP (mmHg) in substitution trials. 265

S136 Fig. Publication bias funnel plots for the effect of fruit on SBP (mmHg) in substitution trials..........266

S137 Fig. Publication bias funnel plots for the effect of mixed sources (with SSBs) on SBP (mmHg) in substitution trials. 267

S138 Fig. Publication bias funnel plots for the effect of SSBs on SBP (mmHg) in addition trials...............268

S139 Fig. Publication bias funnel plots for the effect of 100% fruit juice on SBP (mmHg) in addition trials. 269

S140 Fig. Publication bias funnel plots for the effect of fruit on SBP (mmHg) in addition trials................270

S141 Fig. Publication bias funnel plots for the effect of SSBs on DBP (mmHg) in addition trials. 271

S142 Fig. Trim and Fill funnel plot for the effect of SSBs on DBP (mmHg) in addition trials. 272

S143 Fig. Publication bias funnel plots for the effect of 100% fruit juice on DBP (mmHg) in addition trials. 273

S144 Fig. Publication bias funnel plots for the effect of fruit on DBP (mmHg) in addition trials. 274

S145 Fig. Post-hoc subgroup analyses for the effect of important food sources of fructose-containing sugars on SBP (mmHg) in substitution trials. 275

S146 Fig. Post-hoc subgroup analyses for the effect of important food sources of fructose-containing sugars on SBP (mmHg) in addition trials 276

S147 Fig. Post-hoc subgroup analyses for the effect of important food sources of fructose-containing sugars on SBP (mmHg) in subtraction trials...277

S148 Fig. Post-hoc subgroup analyses for the effect of important food sources of fructose-containing sugars on DBP (mmHg) in substitution trials...278

S149 Fig. Post-hoc subgroup analyses for the effect of important food sources of fructose-containing sugars on DBP (mmHg) in addition trials....279

S150 Fig. Post-hoc subgroup analyses for the effect of important food sources of fructose-containing sugars on DBP (mmHg) in subtraction trials. 280

S151 Fig. Post-hoc subgroup analyses for the effect of SSBs on SBP (mmHg) in substitution trials. 281

S152 Fig. Post-hoc subgroup analyses for the effect of Fruit on SBP (mmHg) in substitution trials. 282

S153 Fig. Post -hoc subgroup analyses for the effect of Mixed sources (with SSBs) on SBP (mmHg) in substitution trials. 283

S154 Fig. Post-hoc subgroup analyses for the effect of SSBs on SBP (mmHg) in addition trials. 284

S155 Fig. Post-hoc subgroup analyses for the effect of Fruit on SBP (mmHg) in addition trials. 285

S156 Fig. Post-hoc subgroup analyses for the effect of 100% Fruit Juice on SBP (mmHg) in addition trials. 286

S157 Fig. Post-hoc subgroup analyses for the effect of SSBs on DBP (mmHg) in addition trials................287

S158 Fig. Post-hoc subgroup analyses for the effect of Fruit on DBP (mmHg) in addition trials. 288

S159 Fig. Post-hoc subgroup analyses for the effect of 100% Fruit Juice on DBP (mmHg) in addition trials. 289

S1 Table. PRISMA Checklist.

Section/topic	#	Checklist item	Reported on page #
**TITLE**			
Title	1	Identify the report as a systematic review, meta-analysis, or both.	Title page
**ABSTRACT**			
Structured summary	2	Provide a structured summary including, as applicable: background; objectives; data sources; study eligibility criteria, participants, and interventions; study appraisal and synthesis methods; results; limitations; conclusions and implications of key findings; systematic review registration number.	1
**INTRODUCTION**			
Rationale	3	Describe the rationale for the review in the context of what is already known.	2
Objectives	4	Provide an explicit statement of questions being addressed with reference to participants, interventions, comparisons, outcomes, and study design (PICOS).	2-3
**METHODS**			
Protocol and registration	5	Indicate if a review protocol exists, if and where it can be accessed (e.g., Web address), and, if available, provide registration information including registration number.	3
Eligibility criteria	6	Specify study characteristics (e.g., PICOS, length of follow-up) and report characteristics (e.g., years considered, language, publication status) used as criteria for eligibility, giving rationale.	3-4
Information sources	7	Describe all information sources (e.g., databases with dates of coverage, contact with study authors to identify additional studies) in the search and date last searched.	3-4
Search	8	Present full electronic search strategy for at least one database, including any limits used, such that it could be repeated.	S2-S3 Tables
Study selection	9	State the process for selecting studies (i.e., screening, eligibility, included in systematic review, and, if applicable, included in the meta-analysis).	4
Data collection process	10	Describe method of data extraction from reports (e.g., piloted forms, independently, in duplicate) and any processes for obtaining and confirming data from investigators.	5
Data items	11	List and define all variables for which data were sought (e.g., PICOS, funding sources) and any assumptions and simplifications made.	5
Risk of bias in individual studies	12	Describe methods used for assessing risk of bias of individual studies (including specification of whether this was done at the study or outcome level), and how this information is to be used in any data synthesis.	5
Summary measures	13	State the principal summary measures (e.g., risk ratio, difference in means).	5-6
Synthesis of results	14	Describe the methods of handling data and combining results of studies, if done, including measures of consistency (e.g., I^2) for each meta-analysis.	6-7

Risk of bias across studies	15	Specify any assessment of risk of bias that may affect the cumulative evidence (e.g., publication bias, selective reporting within studies).	8
Additional analyses	16	Describe methods of additional analyses (e.g., sensitivity or subgroup analyses, meta-regression), if done, indicating which were pre-specified.	7
**RESULTS**			
Study selection	17	Give numbers of studies screened, assessed for eligibility, and included in the review, with reasons for exclusions at each stage, ideally with a flow diagram.	8-9, Fig 1
Study characteristics	18	For each study, present characteristics for which data were extracted (e.g., study size, PICOS, follow-up period) and provide the citations.	9-11
Risk of bias within studies	19	Present data on risk of bias of each study and, if available, any outcome level assessment (see item 12).	11-12
Results of individual studies	20	For all outcomes considered (benefits or harms), present, for each study: (a) simple summary data for each intervention group (b) effect estimates and confidence intervals, ideally with a forest plot.	S5-S12 Figs
Synthesis of results	21	Present results of each meta-analysis done, including confidence intervals and measures of consistency.	12-15, Figs 2,3
Risk of bias across studies	22	Present results of any assessment of risk of bias across studies (see Item 15).	18-19
Additional analysis	23	Give results of additional analyses, if done (e.g., sensitivity or subgroup analyses, meta-regression [see Item 16]).	15-18
**DISCUSSION**			
Summary of evidence	24	Summarize the main findings including the strength of evidence for each main outcome; consider their relevance to key groups (e.g., healthcare providers, users, and policy makers).	19-23, 24-25
Limitations	25	Discuss limitations at study and outcome level (e.g., risk of bias), and at review-level (e.g., incomplete retrieval of identified research, reporting bias).	23-24
Conclusions	26	Provide a general interpretation of the results in the context of other evidence, and implications for future research.	25
**FUNDING**			
Funding	27	Describe sources of funding for the systematic review and other support (e.g., supply of data); role of funders for the systematic review.	Statement in submission portal

S2 Table. Search strategy for controlled trials assessing the effect of important food sources of fructose-containing sugars on blood pressure.

Database and search terms		
MEDLINE	Embase	The Cochrane Library of Controlled Trials
1 exp fructose/	1 exp fructose/	1 exp fructose/
2 fructose.mp.	2 fructose.mp.	2 fructose.mp.
3 exp sucrose/	3 exp sucrose/	3 exp sucrose/
4 sucrose.mp.	4 sucrose.mp.	4 sucrose.mp.
5 sugar*.mp.	5 sugar*.mp.	5 sugar*.mp.
6 SSB.mp.	6 SSB.mp.	6 SSB.mp.
7 sweetened.mp.	7 sweetened.mp.	7 sweetened.mp.
8 soft drink*.mp.	8 soft drink*.mp.	8 soft drink*.mp.
9 cola.mp.	9 exp Honey/	9 cola.mp.
10 exp Honey/	10 honey.mp.	10 exp carbonated beverages/
11 honey.mp.	11 fruit.mp.	11 carbonated beverage*.mp.
12 fruit.mp.	12 exp fruit/	12 exp Energy Drinks/
13 exp fruit/	13 exp Carbonated Beverages/	13 energy drink*.mp.
14 exp soft drink/	14 carbonated beverage*.mp.	14 exp honey/
15 exp carbonated beverage/	15 exp energy drink/	15 honey.mp.
16 carbonated beverage*.mp.	16 energy drink*.mp.	16 exp fruit/
17 exp energy drink/	17 exp soft drink/	17 fruit.mp.
18 energy drink*.mp.	18 HFCS.mp.	
19 HFCS.mp.	19 cola.mp.	19 sugar*sweetened* beverage*.mp.
20 sugar* sweetened beverage*.mp.	20 sugar* sweetened beverage*.mp.	20 HFCS.mp.
21 hypertensive*.mp.	21 hypertensive*.mp.	21 hypertensive*.mp.
22 exp Hypertension/	22 exp Hypertension/	22 exp hypertension/
23 hypertension*.mp.	23 hypertension*.mp.	23 blood pressure.mp.
24 blood pressure.mp.	24 blood pressure.mp.	24 exp blood pressure/
25 exp Blood Pressure/	25 exp Blood Pressure/	25 systolic blood pressure.mp.
26 systolic blood pressure.mp.	26 systolic blood pressure.mp.	26 SBP.mp.
27 SBP.mp.	27 SBP.mp.	27 diastolic blood pressure.mp.
28 diastolic blood pressure.mp.	28 diastolic blood pressure.mp.	28 DBP.mp.
	29 DBP.mp.	29 hypertension*.mp.

29 DBP.mp.<br>30 or/1-20<br>31 or/21-29<br>32 30 and 31<br>33 limit 32 to animals<br>34 32 not 33<br>35 clinical trial.mp.<br>36 clinical trial.pt.<br>37 random:.mp.<br>38 tu.xs.<br>39 or/35-38<br>40 34 and 39	30 or/1-20<br>31 or/21-29<br>32 30 and 31<br>33 limit 32 to animals<br>34 32 not 33<br>35 limit 34 to animal studies<br>36 34 not 35<br>37 random:.tw.<br>38 clinical trial:.mp.<br>39 exp health care quality/<br>40 or/37-39<br>41 36 and 40	30 or/1-19<br>31 or/20-20<br>32 30 and 31

S3 Table. PICOTS framework of the search strategy.

**PICOTS framework defined in the present systematic review and meta-analysis**					
**Participants**	**Interventions**	**Comparators**	**Outcomes**	**Time**	**Study design**
Individuals of all ages and health backgrounds	Food sources of fructose-containing sugars	Diets and foods free or lower (minimum 5g sugar difference) in fructose-containing sugars	Systolic blood pressure, diastolic blood pressure, mean difference and 95% confidence intervals	≥7 days	Controlled trials done in humans

PICOTS=participants, interventions, comparators, outcomes, time and study design

S4 Table. Food source definitions.

Food source of fructose-containing sugars	Definition
SSB	Carbonated or non-carbonated beverages where all or the majority of sugars are added sugars. This also includes interventions where sugars were provided to participants as crystalline packages and where they are instructed to add or incorporate into beverages.
Sweetened dairy	Animal dairy products sweetened with added sugars and where the control includes non-dairy products. These would contain both added and naturally occurring sugars.
Sweetened dairy alternatives (soy)	Soy-based dairy products sweetened with added sugars and where the control includes non-soy-based dairy products.
Sweetened dairy alternative (other)	Other plant-based dairy products sweetened with added sugars and where the control includes non-plant-based dairy products.
Fruit drink	Fruit drinks which are derived from fruit juices or fruit flavouring with added sugars. These may contain both added and naturally occurring sugars.
100% Fruit juice	Fruit juice which is derived 100% from fruits with no added sugar. The one exception was cranberry juice, in which a small amount of added sugars was added for palatability.
Fruit	Includes whole fruit, freeze-dried powdered fruit, smoothies in which the only difference between intervention groups is the fruit present. The dose of the sugars under investigation is naturally occurring coming from fruit.
Dried fruit	Includes unsweetened and sweetened dried fruit. Sugars can be naturally occurring, or both naturally occurring and added.
Mixed fruit forms	Interventions include two or more of the food sources of fruit sugars (i.e., fruit, dried fruit, 100% fruit juice). Sugars are naturally occurring coming from fruit.
Sweetened cereal grains and bars	Includes sweetened dried cereal, nut bars and fruit and nut bars. Sugars are added.
Sweets and desserts	Includes cookies, cakes, muffins, confectionaries,

	fondant, etc. Sugars are added.
Added nutritive (caloric) sweetener	Sugars provided to participants as crystalline packages or syrup or honey, where they are instructed to add or incorporate it to various foods. Sugars are added regulatory designations.
Mixed sources (with SSBs)	Interventions where fructose-containing sugars were consumed in the form of SSBs in addition to other food sources. Examples include whole dietary interventions. Sugars can be added, or both naturally occurring and added.
Mixed sources (without SSBs)	Interventions include two or more of the above food sources of fructose-containing sugars with the exception of SSBs. Sugars can be added, or both naturally occurring and added.

SSB=sugar-sweetened beverages

S5 Table. Trial characteristics.

Study, Year	Participants (M, F)	Setting	Mean Age, years*	BW, kg*	BMI, kg/m² *	Baseline SBP, mmHg*	Baseline DBP, mmHg*	Design	Feeding Control [a]	Randomization	Dose, g/d (% E)[b]	Interventions	Food matrix	Diet [c]	E Balance [d]	F/U	Funding [e]	Med use
Substitution Trials																		
**SSB**																		
**Aeberli et al. 2011**	29 NW (29 M, 0 F)	OP, Switzerland	26.3 (6.6)	73.7 (8.8)	22.4 (1.9)	125.9 (8.5)	74.1 (8.5)	C	Supp	Yes					Positive	3 wk	A, I	No
Intervention											80 (~13)	Fructose	Fructose SSB	~55:32:13				
Intervention											80 (~13)	Sucrose	Sucrose SSB	~55:32:13				
Intervention											40 (~7)	Fructose	Fructose SSB	~51:35:14				
Control												Glucose	Glucose SSB (40g)	~53:32:13				
Control												Glucose	Glucose SSB (80g)	~57:31:13				
**Aeberli et al. 2013**	9 NW (9 M, 0 F)	OP, Switzerland	22.8 (1.7)	NR	22.6 (1.4)	NR	NR	C	Supp	Yes					Positive	3 wk	A, I	No
Intervention											80 (~14)	Fructose	Fructose SSB	~56:31:13				
Intervention											80 (~14)	Sucrose	sucrose SSB	~54:30:16				
Control												Glucose	Glucose SSB	54:31:14				
**Chiu et al. 2020**	30 OW/OB (30 M, 0 F)	OP, USA	15.3 (1.5)	86.8 (15.7)	1.8 (0.5)[f]	119 (9)	60 (6)	C	Supp	Yes				49:36:16	Neutral	3 wk	A, I	No
Intervention											80 (22)	HFCS	24 oz/d SSB					
Control												Lactose	Energy-equivalent amount of 2% milk (22 oz/d)					
**Engel et al. 2018**	29 OW/OB (12 M, 17 F)	OP, Denmark						P	Supp	Yes					Positive	6 mo	A, I	Unclear
Intervention	14 OW/OB (8 M, 6 F)		37.8 (8)	94.9 (11.6)	30.8 (2.8)	123.4 (8.9)	74.4 (9.7)				~106 (~21)	Sucrose	1 L/d sucrose-sweetened regular cola (50% glucose 50% fructose)	54:31:13				

Study, Year	Participants (M, F)	Setting	Mean Age, years*	BW, kg*	BMI, kg/m^2*	SBP, mmHg*	DBP, mmHg*	Design	Feeding Control [a]	Randomization	Dose, g/d (% E)[b]	Interventions	Food matrix	Diet [c]	E Balance [d]	F/U	Funding [e]	Med use
Control	15 OW/OB (4 M, 11 F)		37,7 (9.1)	94.0 (17.1)	31.4 (3.0)	124.6 (14.9)	76.0 (8.5)					Lactose	1 L/d semi-skim milk (1.5%)	`47:33:18				
**Geidl-Flueck et al. 2021**	68 OW/OB (68 M, 0 F)	OP, Switzerland	22.7 (2.4)	71.5 (7.7)	21.8 (1.6)			P	Supp	Yes					Positive	7wk	A	No
Intervention	23 OW/OB (23 M, 0 F)			69.2 (7.7)	21.2 (2.3)	122.6 (8.8)	67.3 (11.8)				79.8 (16.2)	Fructose	200ml fructose SSB consumed 3 times/day with meals	55:30:15				
Intervention	23 OW/OB (23 M, 0 F)			75.5 (7.3)	22.9 (1.4)	126.2 (7.2)	67.2 (8.1)				79.8 (14.3)	Sucrose	200ml sucrose SSB consumed 3 times/day with meals	52:33:16				
Control	22 OW/OB (22 M, 0 F)			71.6 (6.8)	22 (2.3)	125.7 (9.0)	71.6 (8.6)				79.8 (14.3)	Glucose	200 ml glucose SSB consumed 3 times/day with meals	56:31:15				
**Hieronimus et al. 2020**	84 MW	IP/OP, USA		NR				P	Met/Supp	No				55:30:15	Positive	2 wk	A	No
Intervention	28 MW (13F, 15M)		27 (7)		24.9 (4)	117.08 (9.96)	72.65 (7.22)				`(25)	HFCS	25% HFCS koolaid					
Intervention	28 MW (13F, 15M)		27 (6)		25.4 (3.7)	117.08 (9.75)	71.67 (6.51)				`(25)	Fructose	25% Fructose koolaid					
Control	28 MW (13F, 15M)		26 (6)		25.8 (3.4)	118.9 (11.2)	73.85 (8.26)					Glucose	25% Glucose koolaid					
**Koivisto and Yki-Järvinen 1993**	10 DM2 (4 M, 6 F)	IP, Finland	61 (10)	81.9 (15.4)	27.5 (4.1)			C	Met	Yes				50:30:20	Neutral	4 wk	A, I	Unclear
Intervention				82.0 (15.8)		142 (25.3)	85 (12.6)				~55 (~10)	Fructose	Fructose dissolved in water					
Control				81.8 (15.8)		137 (15.8)	83 (12.6)					Starch	Glucose dissolved in water					
**Sigala et al. 2020**	52 MW (27, 25)	IP/OP, USA		NR				P	Met/Supp	No				55:30:15	Positive	2 wk	A	No
Intervention	24 MW (12M, 12F)		25.9 (6.3)		25.3 (3.4)	114.3 (8.4)	72.2 (5.5)				`(25)	Sucrose	25% Sucrose Koolaid					
Control	28 MW (15M, 13F)		26 (6)		25.8 (3.4)	118.9 (11.2)	73.85 (8.26)					Glucose	25% Glucose koolaid					
**Silbernagel et al. 2011**	20 MW (12 M, 8 F)	OP, Germany	30.5 (8.9)	80.5 (4.2)	25.9 (2.3)			P	Supp	Yes				50:35:15	Positive	4 wk	A	Unclear

Study, Year	Participants (M, F)	Setting	Mean Age, years*	BW, kg*	BMI, kg/m^2 *	SBP, mmHg*	DBP, mmHg*	Design	Feeding Control [a]	Randomization	Dose, g/d (% E)[b]	Interventions	Food matrix	Diet [c]	E Balance [d]	F/U	Funding [e]	Med use
Intervention	10 MW (7 M, 3 F)		32.8 (9.3)	80.3 (9.1)	25.5 (2.2)	120 (12.6)	79 (9.5)				150 (~22.1)	Fructose	Fructose dissolved in water					
Control	10 MW (5 M, 5 F)		28.2 (8.4)	80.7 (7.5)	26.2 (2.4)							Glucose	Glucose dissolved in water					
**Stanhope et al. 2009**	32 OW/OB (16M, 16F)	IP/OP, USA						P	Met/Supp	No				55:30:15	Neutral	10 wk	A, I	No
Intervention	17 OW/OB (9M, 8F)		53 (40-27)	86.7 (10.7)	29.3 (2.6)	120 (8.2)	76 (4.1)				(25)	Fructose	25% E as fructose-sweetened Kool-Aid					
Control	15 OW/OB (7M, 8F)		54 (40-27)	85.9 (10.5)	29.4 (3.2)	122 (7.7)	77 (3.9)					Glucose	25% E as glucose-sweetened Kool-Aid					
**Swarbrick et al. 2008**	7 OW/OB (0 M, 7 F)	IP, USA	(50-72)	75.7 (24.3)	29.1 (5.8)			C	Met	No				55:30:15	Neutral		A	Unclear
Intervention						130 (14)	70 (12)				~125 (25)	Fructose	Fructose SSB (12 % solution flavored with unsweetened drink mix)			10 wk		
Control						NR	NR					Starch	Complex carbohydrate sources (bread, rice, pasta)			4 wk		
**Sweetened dairy**																		
**Angelopoulos et al. 2015**	267 MW (96M,171F)	OP, USA	37.7 (12.1)	73.8 (12.4)	26.3 (3.3)	109.18 (10.15)	69.78 (8.69)	P	Supp	Yes					Neutral	10 wk	I	Unclear
Intervention	65 MW		38.7 (12.2)	75.7 (14)	26.5 (3.33)	107.71 (10.51)	69.68 (8.83)				~50 (9)	Fructose	9% Fructose in milk	52:28:19				
Intervention	64 MW		39.8 (13.2)	73.5 (12.4)	26.8 (3.5)	111.14 (10.28)	68.96 (8.34)				~104 (18)	Sucrose	18% Sucrose in milk	57:25:17				
Intervention	61 MW		36.3 (10.7)	73.7 (11.9)	26.3 (3.2)	108.61 (9.7)	69.48 (9.71)				~108 (18)	HFCS	18% HFCS in milk	55:26:18				
Control	77 MW		36.1 (12.1)	72.3 (11.3)	25.7 (3.14)	109.17 (10.09)	71.14 (8.02)					Glucose	9% Glucose in milk	52:29:19				

Study, Year	Participants (M, F)	Setting	Mean Age, years*	BW, kg*	BMI, kg/m^2 *	SBP, mmHg*	DBP, mmHg*	Design	Feeding Control [a]	Randomization	Dose, g/d (% E)[b]	Interventions	Food matrix	Diet [c]	E Balance [d]	F/U	Funding [e]	Med use
**Sweetened dairy alternative (soy)**																		
**Maleki et al. 2019**	62 NAFLD (19M, 43F)	OP, Iran						P	Supp	Yes				55:30:15	Neutral	8 wk	A, I	No
Intervention	31 NAFLD (10M, 21F)		46.16 (10.53)	83.03 (9.98)	30.85 (3.53)	127.41 (7.15)	83.48 (4.78)				5.6 (1.2)[g]	Sucrose	240 mL soy milk/d with a low-calorie diet					
Control	31 NAFLD (9M, 22F)		45.16 (9.86)	84.97 (14.02)	31.29 (3.77)	125.51 (7.16)	82.06 (4.98)					Mixed	Low-calorie diet					
**Fruit**																		
**Agebratt et al. 2016**	30 MW (18 M, 12 F)	OP, Sweden	23.5 (3.7)		22.3 (1.9)			P	DA	Yes				NR	Positive	8 wk	A	Unclear
Intervention	15 MW (7 M, 8 F)			66.5 (8.7)	22.2 (1.6)	110.9(7.7)	67.6 (6.7)				96 (~14.6)	Fruit	7 kcal/kg BW/d Fruit					
Control	15 MW (11 M, 4 F)			73.6 (9.0)	22.5 (2.3)	113.5 (7.2)	66.7 (7.0)					Fat	7 kcal/kg BW/d Walnuts					
**Amani et al. 2014**	36 DM2 (13 M, 23 F)	OP, Iran	51.6 (11.1)					P	Supp	Yes				NR	Positive	6 wk	A,I	No
Intervention	19 DM2		51.9 (8.2)	75.79 (9.02)	27.36 (4.23)	129.95 (14.9)	84.2 (8.03)				~24.45 (~5.6)	Fruit	50g pure freeze dried strawberry powder with 2 cups of water, on top of a diet low in flavenoid-rich foods					
Control	17 DM2		51.1 (13.8)	73.38 (11.98)	28.58 (4.7)	127.6 (15.6)	86.76 (6.3)					Lactose	12g Lactose, 4g pectin, 4g sugar-free instant strawberry powder with 2 cups of water, on top of a diet low in flavenoid-rich foods					

Study, Year	Participants (M, F)	Setting	Mean Age, years*	BW, kg*	BMI, kg/m^2 *	SBP, mmHg*	DBP, mmHg*	Design	Feeding Control [a]	Randomization	Dose, g/d (% E)[b]	Interventions	Food matrix	Diet [c]	E Balance [d]	F/U	Funding [e]	Med use
**Beisner et al. 2020 (lean)**	6 NW (6F, 0M)	OP, Germany	26 (2)	NR	22.5 (1.5)	104.2 (7.4)	69.2 (6.7)	C	Supp	No				55:30: 15	Neutral	1 wk	A	No
Intervention											`(20)	Fruit	High fruit and vegetable diet					
Control												Mixed comparator	Diet avoiding fructose foods; <2% fructose					
**Beisner et al. 2020 (OB)**	6 OW/OB (6F, 0M)	OP, Germany	30 (3)	NR	41.5 (4)	122.5 (9.9)	83.3 (6.8)	C	Supp	No				55:30: 15	Neutral	1 wk	A	No
Intervention											`(20)	Fruit	High fruit and vegetable diet					
Control												Mixed comparator	Diet avoiding fructose foods; <2% fructose					
**Du et al. 2019**	49 Osteoarthritis (14 M, 35 F)	OP, USA	55.6 (45-79)	NR	32.2 (6.2)			P	Supp	Yes				NR	Positive	4 mo	I	Unclear
Intervention	27 Osteoarthritis (9 M, 18 F)					136.2 (19.8)	80.2 (9.7)				30 (6)[h]	Fruit	40g/d freeze dried blueberry powder dissolved in 10-12 oz water					
Control	22 Osteoarthritis (5 M, 17 F)					125.5 (18.5)	76.8 (11.2)					Maltodextrin	40 g/d maltodextrin powder dissolved in 10-12 oz water					
**Hegde et al. 2013**	123 DM2	OP, India	58.0 (9.2)	NR	24.9 (3.9)			P	DA	No				NR	Positive	3 mo	A	Unclear
Intervention	60 DM2		58.5 (9.6)		24.4 (3.9)	140.2 (20.6)	83.5 (9.8)				~16.5 (~3.3)	Fruit	Incorporate 2 fruit/d into usual diet					
Control	63 DM2		57.5 (8.9)		25.3 (3.9)	139.6 (21.0)	84.1 (9.9)					Mixed	Usual diet					
**Lehtonen et al. 2011**	80 OW/OB (0 M, 80 F)	OP, Finland	44.2 (6.2)	81.6 (8.5)	29.6 (2.1)	133.3 (14.3)	84.2 (7.9)	C	Supp	Yes				NR	Neutral	33/35d	A,I	No

Study, Year	Participants (M, F)	Setting	Mean Age, years*	BW, kg*	BMI, kg/m^2*	SBP, mmHg*	DBP, mmHg*	Design	Feeding Control [a]	Randomization	Dose, g/d (% E)[b]	Interventions	Food matrix	Diet [c]	E Balance [d]	F/U	Funding [e]	Med use
Intervention											8.4 (1.7)[i]	Fruit	100 g/d billberry (frozen, whole)					
Control												Mixed	Usual diet					
**Madero et al. 2011**	131 OW/OB (29 M, 102 F)	OP, Mexico	38.3 (8.8)	80.9 (13.4)	32.4 (4.5)			P	DA	Yes				55:30:15	Negative	6 wk	A	No
Intervention	65 OW/OB (15 M, 50 F)		40.2 (8.1)	79.1 (13.4)	32.8 (4.5)	109.1 (9.1)	69.64 (5.2)				~50-70 (~13.6)	Fruit	Various fruits					
Control	66 OW/ OB (14 M, 52 F)		37.6 (9.3)	82.7 (13.3)	32.9 (4.5)	108.9 (9.1)	69.01 (5.1)					Starch	Low fructose diet substituted with cereal products					
**Navaei et al. 2019**	40 MetS (10 M, 30 F)	OP, USA	59 (5)	NR	33.2 (4.9)	136 (11)	82 (6)	C	Supp	Yes					Neutral	3 mo	I	Mixed
Intervention											~16 (3.2)[j]	Fruit	2 medium fresh pears (166 g/d)	48:37:16				
Control												NSB	50 g calorie matched NNS powder and 480 mL water/d	47:39:16				
**Puupponen-Pimia et al. 2013**	32 MetS (13 M, 19 F)	OP, Finland						P	Supp	Yes				NR	Neutral	8 wk	A	Mixed
Intervention	20 MetS (10 M, 10 F)		53 (6.5)	92.4 (14.4)	31.8 (4.4)	139.2 (18.9)	91.5 (9)				~32 (6.4)	Fruit	300 g fresh berries (strawberry, rasberry, and cloudberry) replacing other carbs					
Control	12 MetS (3 M, 9 F)		49.8 (7.1)	93.1 (10.8)	32.9 (3.4)	144.3 (12.3)	92.4 (8.4)					Mixed	Usual diet					
**Singh et al. 1997**	101 HTN, HCL (88 M, 13 F)	OP, India	50.5 (8.5)					P	Supp	Yes					Neutral	6 mo	NR	No
Intervention	52 HTN, HCL (43 M, 9 F)		49.1 (7.5)	67.8 (9.6)	25.2 (3.1)	165.2 (7.5)	107.0 (4.6)				~36.8 (~7)	Fruit	412 g/d guava	63:23:14				
Control	49 HTN, HCL (45 M, 4 F)		52.0 (9.2)	69.2 (11.4)	25.6 (3.6)	168.3 (8.6)	106.5 (4.1)				~9 (~1.7)	Mixed	Refined carbs, saturated fat, and	57:29:14				

cholesterol

Study, Year	Participants (M, F)	Setting	Mean Age, years*	BW, kg*	BMI, kg/m^2*	SBP, mmHg*	DBP, mmHg*	Design	Feeding Control [a]	Randomization	Dose, g/d (% E)[b]	Interventions	Food matrix	Diet [c]	E Balance [d]	F/U	Funding [e]	Med use
**Dried fruit**																		
**Anderson et al. 2014**	46 MetS (21M, 25F)	OP, USA	60.6 (NR)					P	Supp	Yes				NR	Neutral	3 mo	I	Mixed
Intervention	31 MetS (12 M, 19 F)		60.3 (NR)	86.3 (12.2)	30.0 (2.8)	135.0 (12.8)	84.4 (11.1)				~60 (~12)	Dried fruit	84 g/d Raisins					
Control	15 MetS (9 M, 6 F)		61.1 (NR)	85.2 (12.4)	29.2 (2.3)	129.2 (6.2)	78.9 (7.4)					Mixed	Processed snacks					
**Bays et al. 2015**	46 DM2 (27M, 19F)	OP, USA	58.4 (NR)	NR				P	Supp	Yes				NR	Neutral	3 mo	I	Mixed
Intervention	27 DM2 (17 M, 10 F)		58 (NR)		34 (5)	129 (16)	74 (10)				~60 (~12)	Dried fruit	84 g/d Raisins					
Control	19 DM2 (10 M, 9 F)		59 (NR)		37 (7)	130 (13)	75 (9)					Mixed	Processed snacks					
**Kaliora et al. 2016**	44 NAFLD (NR)	OP, Greece						P	Supp	Yes				50:30:20	Negative	6 mo	A, I	Unclear
Intervention	23 NAFLD (NR)		50.7 (10.9)[k]	85.7 (14.3)	29.5 (4.3)	129.1 (13.4)	80.3 (9.1)				24.2 (~4.98)[j]	Dried fruit	36g/d Corinthian currants					
Control	21 NAFLD (NR)		51.6 (9.4)[k]	82.0 (3.0)	28.2 (3.7)	130.3 (13.2)	81.4 (8.7)					Mixed	Snacks (low fat yogurt, mini crackers, or bread with low fat cheese)					
**Kanellos et al. 2014**	48 H (25 M, 23 F)	OP, Greece						P	Supp	Yes				NR	Neutral	6 mo	A, I	Mixed
Intervention	26 H (15 M, 11 F)		63.7 (6.3)	83.4 (13.8)	30.5 (4.4)	131.8 (11.8)	76.8 (10.5)				24 (4.9)	Dried fruit	24 g/d Corinthian raisins					
Control	22 H (10 M, 12 F)		63 (8.5)	81.2 (14.3)	30.4 (5.5)	129.3 (16.1)	75.3 (8.0)					Starch	Snacks					
**Kanellos et al. 2017**	33 Smokers	OP, Greece						P	Supp	Yes					Neutral	4 wk	A, I	No
Intervention	20 Smokers		30.8 (7.5)[k]	77.5 (13.8)	24.4 (2.68)	117.2 (10.3)	74.7 (8.9)				60 (~12)[j]	Dried fruit	90 g/d Raisins	41:27:13				

Study, Year	Participants (M, F)	Setting	Mean Age, years*	BW, kg*	BMI, kg/m^2*	SBP, mmHg*	DBP, mmHg*	Design	Feeding Control [a]	Randomization	Dose, g/d (% E)[b]	Interventions	Food matrix	Diet [c]	E Balance [d]	F/U	Funding [e]	Med use
Control	13 Smokers		29.8 (5.0)[k]	78.1 (14.1)	23.9 (3.24)	117.2 (10.5)	77.1 (8.2)					Mixed	Snacks (low fat yogurt, mini crackers, or bread with low fat cheese)	43:33:17				
**Lehtonen et al. 2011**	80 OW/OB (0 M, 80 F)	OP, Finland	44.2 (6.2)	81.6 (8.5)	29.6 (2.1)	133.3 (14.3)	84.2 (7.9)	C	Supp	Yes				NR	Neutral	33-35 d	A, I	No
Intervention											10.3 (~2)[l]	Dried fruit	100 g/d Sea Buckthorn berry (air dried)					
Control												Mixed	Usual diet					
**Puglisi et al. 2008 (walk control)**	22 MW (11 M, 11 F)	OP, USA	56.3 (4.6)	78.6 (16.0)	27.7 (3.8)			P	Supp	Yes					Neutral	6 wk	I	Unclear
Intervention	10 MW (5 M, 5 F)		57.8 (5.2)	78.4 (15.9)	27.5 (3.8)	123.0 (14)	80.6 (8.4)				86 (~17.2)[j]	Dried fruit	1 cup/d raisins w/ a polyphenol restricted diet and walking	56:29:15				
Control	12 MW (6 M, 6 F)		55.0 (3.8)	78.7 (16.8)	27.9 (3.9)	120.7 (9.9)	78 (5.8)					Mixed	Polyphenol restricted diet and walking	43:40:16				
**Puglisi et al. 2008 (run-in control)**	12 MW (6 M, 6 F)	OP, USA	54.4 (3.5)	70.8 (12.2)	24.9 (2.3)			C	Supp	No					Neutral		I	Unclear
Intervention						124.3 (15.2)	79,7 (10.6)				86 (~17.2)[j]	Dried fruit	1 cup/d raisins w/ a polyphenol restricted diet	`56:31:15		6 wk		
Control						NR	NR					Mixed	Polyphenol restricted diet			2 wk		
**Sweetened fruit and nut bars**																		
**Jones et al. 2014**	50 MW	OP, USA	26.2 (7.2)	69.0 (16.0)	23.6 (3.7)	120.56	75.25	P	Supp	Yes				NR	Neutral	3 mo	I	
Intervention	25 MW										6 (~1.2)	Sucrose[m]	Honey roasted peanuts					
Control	25 MW											Fat	Unsalted peanuts					

Study, Year	Participants (M, F)	Setting	Mean Age, years*	BW, kg*	BMI, kg/m^2*	SBP, mmHg*	DBP, mmHg*	Design	Feeding Control [a]	Randomization	Dose, g/d (% E)[b]	Interventions	Food matrix	Diet [c]	E Balance [d]	F/U	Funding [e]	Med use
**Sweets and desserts**																		
**Claesson et al. 2009**	25 MW (11 M, 14 F)	OP, Sweden	23.4 (2.7)	68.0 (6.7)	22.2 (1.7)			P	Supp	Yes					Positive	2 wk	A	Unclear
Intervention	12 MW (5 M, 7 F)		23.2 (3.5)	67.3 (7.6)	22.2 (1.4)	116 (8.3)	72 (7.3)				278 (~37)	Sucrose	Candy, 20kcal/kg BW/d	66:22: 11				
Control	13 MW (6 M, 7 F)		23.6 (1.8)	68.7 (6.1)	22.2 (2.0)	117 (8.5)	69 (8.1)					Fat	Peanuts, 20kcal/kg BW/d	32:48: 18				
**Dikariyanto et a. 2020**	105 pre-CVD (31M, 74F)	OP, UK		NR				P	Supp	Yes					Neutral	6 wk	A, I	Mixed
Intervention	51 pre-CVD (15M, 36F)		56.0 (10.7)		26.7 (4.5)	124.4 (15.1)	80.6 (7.7)				`(21)	Sucrose	5 Sweet and savory mini-muffins daily (400kcal). 70:30 ratio of sweet and savory muffins.	47:36: 17				
Control	54 pre-CVD (16M, 38F)		56.3 (10.3)		27.3 (4.4)	126.2 (17.6)	83.8 (10.8)				`(2.3)	Nuts	63g/d Almonds (399.42 kcal).	35:47: 18				
**Hallfrisch et al. 1983 (HI)**	12 HI (12 M, 0 F)	IP/OP, USA	39.5 (7.3)	81.4 (8.0)	NR	123	70	C	Met	No				43:42: 15	Neutral	5 wk	NR	Unclear
Intervention											~101.3 (15)	Fructose	Fructose wafer					
Intervention											~50.6 (7.5)	Fructose	Fructose wafer					
Control												Starch	Starch wafer					
**Hallfrisch et al. 1983 (H)**	12 MW (12 M, 0 F)	IP/OP, USA	39.8 (8.3)	80.5 (11.1)	NR	122	71	C	Met	No				43:42: 15	Neutral	5 wk	NR	Unclear
Intervention											~101.3 (15)	Fructose	Fructose wafer					
Intervention											~50.6 (7.5)	Fructose	Fructose wafer					
Control												Starch	Starch wafer					
**Israel et al. 1983**	24 MW (12 M, 12 F)	IP/OP, USA	36.8 (21-51)	72.6 (42-103.6)	NR	119 (14.7)	74 (8.8)	C	Met	Yes				44:42: 14	Neutral	6 wk	NR	Unclear
Intervention											(33)	Sucrose	33% sucrose patty					
Intervention											(18)	Sucrose	18% sucrose patty					
Control											(5)	Starch	5% sucrose patty					

Study, Year	Participants (M, F)	Setting	Mean Age, years*	BW, kg*	BMI, kg/m^2*	SBP, mmHg*	DBP, mmHg*	Design	Feeding Control[a]	Randomization	Dose, g/d (% E)[b]	Interventions	Food matrix	Diet[c]	E Balance[d]	F/U	Funding[e]	Med use
**Added nutritive (caloric) sweetener**																		
**Beisner et al. 2020 (lean)**	6 NW (6F, 0M)	OP, Germany	26 (2)	NR	22.5 (1.5)	104.2 (7.4)	69.2 (6.7)	C	Supp	No				55:30:15	Neutral	1 wk	A	No
Intervention											`(20)	HFCS	Diet supplemented with 100g/d HFCS					
Control												Mixed comparator	Diet avoiding fructose foods; <2% fructose					
**Beisner et al. 2020 (OB)**	6 OW/OB (6F, 0M)	OP, Germany	30 (3)	NR	41.5 (4)	122.5 (9.9)	83.3 (6.8)	C	Supp	No				55:30:15	Neutral	1 wk	A	No
Intervention											`(20)	HFCS	Diet supplemented with 100g/d HFCS					
Control												Mixed comparator	Diet avoiding fructose foods; <2% fructose					
**Cooper et al. 1988**	17 DM2 (6 M, 11 F)	OP, Australia	62.2 (14.0)	69.1 (2.8)	26.0 (3.0)	139 (30.9)	81 (18.6)	C	Supp	Yes				NR	Positive	6 wk	I	Mixed
Intervention											28 (8.2)	Sucrose	28 g sucrose added to hot beverages, fruit juice, milk, cereals, stewed fruit					
Control												Starch	30 g starch and saccharin added to hot beverages, fruit juice, milk, cereals, stewed fruit					
**Despland et al. 2017**	8 MW (8 M 0 F)	IP/OP, Switzerland	NR	NR	NR			C	Met	Yes				55:30:15	Neutral	7-8 d	A	No
Intervention						117.3 (7.4)	66.7 (7.4)				~125 (25)	Honey	25% starch substituted with robinia honey					

Study, Year	Participants (M, F)	Setting	Mean Age, years*	BW, kg*	BMI, kg/m2 *	SBP, mmHg*	DBP, mmHg*	Design	Feeding Control [a]	Randomization	Dose, g/d (% E)[b]	Interventions	Food matrix	Diet [c]	E Balance [d]	F/U	Funding [e]	Med use
Intervention						118.8 (7.4)	66.7 (6.5)				~125 (25)	Fructose, glucose	25% starch substituted with fructose + glucose solution comparable to honey composition					
Control						117.5 (4.0)	63.0 (5.4)					Starch	Low-sugar diet					
**Koh et al. 1988 (IGT)**	9 IGT (3 M, 6 F)	OP, USA	54 (18)	74.5 (15)	NR	126	72	C	Supp	No					Neutral	4 wk	NR	Unclear
Intervention											(15)	Fructose	Fructose packets added to Fruit juice, milk, water or baked goods	~53:32:16				
Control												Glucose	Glucose packets added to Fruit juice, milk, water or baked goods					
**Koh et al. 1988 (NGT)**	9 MW (3 M, 6 F)	OP, USA	50 (15)	65.9 (13.6)	NR	126	72	C	Supp	No					Neutral	4 wk	NR	Unclear
Intervention											(15)	Fructose	Fructose packets added to Fruit juice, milk, water or baked goods	~53:32:16				
Control												Glucose	Glucose packets added to Fruit juice, milk, water or baked goods					
**Mixed sources (with SSBs)**																		
**Black et al. 2006**	13 MW (13 M, 0 F)	OP, UK	33 (11)	86.0 (12.3)	26.6 (3.2)	127 (10.8)	69 (10.8)	C	Met	Yes				55:33:12	Neutral	6 wk	A	Unclear
Intervention											~199 (25)	Sucrose	High sucrose diet (25% E)					

Study, Year	Participants (M, F)	Setting	Mean Age, years*	BW, kg*	BMI, kg/m^2 *	SBP, mmHg*	DBP, mmHg*	Design	Feeding Control [a]	Randomization	Dose, g/d (% E)[b]	Interventions	Food matrix	Diet [c]	E Balance [d]	F/U	Funding [e]	Med use
Control												Starch	Low sucrose diet (10% E)					
**Brymora et al. 2012**	28 CKD (17 M, 11 F)	OP, Poland	59 (15)	85.8 (11.5)	29.9 (4.2)	NR	NR	C	DA	No				55:30:15	Neutral		A	Unclear
Intervention											~59 (10.2)	Fructose, sucrose	Usual diet			2 wk		
Intervention											~53 (9.1)	Fructose, sucrose	Resumed diet post low fructose diet			6 wk		
Control												Starch	Isocaloric low fructose diet through reduction of fruits and added sugars			6 wk		
**Dominiguez-Coello et al. 2020**	239 OW/OB (87 M, 152 F)	OP, Spain	47.2 (8.6)	94.4 (13.5)	34.6 (2.9)	130.2 (15.6)	82.2 (9.8)	P	DA	Yes					Negative	6 mo	A	Unclear
Intervention	118 OW/OB (45 M, 73 F)		48.0 (8.7)	93.9 (13.4)	34.4 (2.8)	130.4 (15.3)	81.7 (8.6)				~33 (9.8)	Fructose	Low-calorie diet	49:30:21				
Control	121 OW/OB (42 M, 79 F)		46.3 (8.4)	95.0 (13.6)	34.8 (3.0)	130.1 (15.9)	82.6 (10.7)					Mixed	Low-calorie, low-fructose diet	49:35:17				
**Jalilvand et al. 2020**	40 DM2 (24F, 16M)	OP, Iran						P	DA	Yes				55:30:15	Neutral	8 wk	A	Mixed
Intervention	20 DM2 (12F, 8M)		53.03 (7.38)	63.31 (6)	24.18 (1.58)	130.75 (11.84)	80.25 (8.34)				25 (5.0)	Mixed fructose types	Conventional diabetic diet from ADA 2017 guideline.					
Control	20 DM2 (12F, 8M)		53 (7.96)	63.60 (5.41)	24.56 (1.35)	132.75 (12.51)	80.50 (9.71)				8.7 (1.8)	Mixed comparator	Restrict sucrose-sweetened and artificially sweetened drinks and foods.					

Study, Year	Participants (M, F)	Setting	Mean Age, years*	BW, kg*	BMI, kg/m² *	SBP, mmHg*	DBP, mmHg*	Design	Feeding Control [a]	Randomization	Dose, g/d (% E)[b]	Interventions	Food matrix	Diet [c]	E Balance [d]	F/U	Funding [e]	Med use
**Lehtonen et al. 2010**	50 OW (0 M, 50 F)	OP, Finland	42.9 (35-52)	81.8	29.4			P	Supp	Yes					Neutral	20 wk	A, I	No
Intervention	28 OW (0 M, 28 F)			81.9	29.3 (2.2)	133.8 (13.8)	87.8 (8.4)				~14.7 (~3.3)[l]	Fruit	Medley of berry containing products	~50:32:17				
Control	22 OW (0 M, 22 F)			81.7	29.5 (1.8)	128.1 (10.7)	83.3 (6.4)					Mixed	Snacks	~46:35:19				
**Lewis et al. 2013**	13 OW/ OB (9 M, 4 F)	OP, UK	46.1 (6.9)	92 (10.5)	31.7 (3.2)	NR	NR	C	Met	Yes					Neutral	6 wk	I	Unclear
Intervention											~101.8 (15)	Sucrose	High sucrose diet (15% E)	~55:33:12				
Control												Starch	Low sucrose diet (5% E)	~55:33:12				
**Luukkonen et al. 2018**	38 OW/OB (17 M, 21 F)	OP, Finland		92.1 (17)		NR	NR	P	Supp	Yes					Positive	3 wk	A	Unclear
Intervention	12 OW/OB (6 M, 6 F)		45 (10)		33 (6)						250 (~33)	Mix	2.8 dL orange juice, 4.3 dL SSBs, and 200 g candy	64:24:12				
Control	12 OW/OB (5 M, 7 F)		52 (10)		31 (6)							Fat	36 g olive oil, 26 g pesto, 54 g pecan nuts, and 20 g butter	60:23:13				
Control	14 OW/OB (6 M, 8 F)		48 (8)		30 (6)							Fat	30 g coconut oil, 40 g butter, 100 g of 40% fat containing blue cheese	26:59:15				
**Madero et al. 2015**	72 H OW/OB (44 M, 38 F)	OP, Mexico			NR			P	DA	Yes				NR	Neutral	4 wk	NR	No
Intervention	38 OW/OB (22 M, 16 F)		48 (7)	86 (16)		124 (13)	84 (8)				164 (23)	Fruit, Sucrose	Low-sodium meal plan, includes added sugars and fruits					
Control	42 H OW/OB (22 M, 12 F)		44 (9)	90 (16)		125 (11)	86 (7)					Starch	Low sodium and low fructose meal plan - fruit and added sugar replaced by					

carbohydrate

Study, Year	Participants (M, F)	Setting	Mean Age, years*	BW, kg*	BMI, kg/m^2*	SBP, mmHg*	DBP, mmHg*	Design	Feeding Control [a]	Randomization	Dose, g/d (% E)[b]	Interventions	Food matrix	Diet [c]	E Balance [d]	F/U	Funding [e]	Med use
**Maki et al. 2020**	30 MetS (11 M, 19 F)	OP, USA	54.1 (10.4)	89.6 (13.1)	31.9 (3.8)	127 (11.5)	81 (10.4)	C	Supp	Yes					Neutral	4 wk	I	Mixed
Intervention											25 (4.7)	Sucrose	Foods included ready-to-eat cereals with milk, waffles with syrup, granola bar, fruits, nuts, and cheese	~60:31:12				
Control												Protein	Rotation of 3 egg-based breakfasts each providing 2 eggs/d, 6 d/wk	~41:32:26				
**Nier et al. 2018**	13 NAFLD (7 M, 5 F)	OP, Germany	7.73 (0.84)	NR	NR			P	DA	No				NR	Neutral	1 yr	A	Unclear
Intervention	6 NAFLD (3 M, 3 F)					111 (12.2)	69 (7.3)				NR	Sucrose, Fructose	*Ad libitum* sucrose and fructose diet					
Control	7 NAFLD (4 M, 3 F)					107 (10.6)	69 (10.6)					Starch	Reduce fructose intake to ~50% replace them with foods containing less fructose of the same food category					
**Palacios et al. 2020**	33 PDM (17M, 16F)	OP, USA	48.3 (12.6)	88.6 (17.2)	30.5 (4)	125 (13.2)	78.3 (9.8)	C	Supp	Yes					Neutral	6 wk	I	Mixed
Intervention											36 (6.4)	Mixed fructose types	Various carb snacks: dried mango, jello pudding, cookies, fruit and grain bar, baked chips, mini bagel, rice cakes, dinner rolls, pretzels	53:32:16				

Study, Year	Participants (M, F)	Setting	Mean Age, years*	BW, kg*	BMI, kg/m^2*	SBP, mmHg*	DBP, mmHg*	Design	Feeding Control [a]	Randomization	Dose, g/d (% E)[b]	Interventions	Food matrix	Diet [c]	E Balance [d]	F/U	Funding [e]	Med use
Control												Nuts	Energy matched almonds (1.5 oz x 2/d)	37:47:19				
**Schwimmer et al. 2019**	40 NAFLD (40M, 0F)	OP, USA	(11-16)					P		Yes					Neutral	8 wk	A	Unclear
Intervention	20 NAFLD (20M, 0F)		13.4 (1.9)	88.7 (26.3)	32.3 (6.3)	117.6 (12.5)	65.3 (10.9)		DA		~88.2 (22)	Sucrose, Fructose	Usual diet which included ≥ 8 oz juice or SSB/wk	49:33:19				
Control	20 NAFLD (20M, 0F)		12.8 (1.8)	88.1 (21.5)	33.7 (5.6)	116.2 (9.3)	66.1 (6.7)		Met		~62.4 (15)	Mixed	Low-sugar diet (<3% E as free sugars); foods with free-sugars substituted with low/no-added-sugar foods	43:36:22				
**Surwit et al. 1997**	42 OB (0 M, 42 F)	OP, USA	40.2 (7.6)					P	Met	Yes					Negative	6 wk	A, I	Unclear
Intervention	20 OB (0 M, 20 F)		40.6 (8.2)	96.1 (13.7)	35.9 (4.8)	139.50 (16.00)	74.85 (11.08)				121.2 (43)	Sucrose	High-sucrose, low fat diet	73:11:19				
Control	22 OB (0 M, 22 F)		40.3 (7.3)	96.7 (12.6)	34.9 (4.4)	131.82 (13.52)	72.82 (9.02)					Starch	Low-sucrose, low fat diet	71:11:20				
**Vos et al. 2009**	10 NAFLD	OP, USA		NR	NR			P	DA	Yes					Neutral	6 mo	A	Unclear
Intervention	4 NAFLD		12.5 (2)			118.0 (7.2)	66.8 (2.8)				31 (7.02)	Sucrose	Low-fat diet, based on the American Heart Association recommendations	51:31:19				
Control	6 NAFLD		13.3 (1.59)			122.5 (9.8)	60.7 (8.8)				23 (5.78)	Fat	Low-fructose diet; elimination of sugar-containing beverages, fruit juice, and food with HFCS	49:37:16				

Study, Year	Participants (M, F)	Setting	Mean Age, years*	BW, kg*	BMI, kg/m^2*	SBP, mmHg*	DBP, mmHg*	Design	Feeding Control [a]	Randomization	Dose, g/d (% E)[b]	Interventions	Food matrix	Diet [c]	E Balance [d]	F/U	Funding [e]	Med use
**Van Meijl et al. 2011**	35 OW/OB (10 M, 25 F)	OP, Netherlands	49.5 (13.2)	NR	32.0 (3.8)	125 (19.9)	88 (13.2)	C	Supp	Yes					Neutral	8 wk	I	Mixed
Intervention											70.2 (~12.8)	Sucrose	Fruit Juice (600 mL), fruit biscuits (43 g)	53:30:16				
Control												Lactose	Low fat milk (500 mL), low fat yogurt (150 g)	46:33:19				
**Addition Trials**																		
**SSB**																		
**Aeberli et al. 2011**	29 NW (29 M, 0 F)	OP, Switzerland	26.3 (6.6)	73.7 (8.8)	22.4 (1.9)	125.9 (8.5)	74.1 (8.5)	C	Supp	Yes					Positive	3 wk	A, I	No
Intervention											80 (~13)	Fructose	Fructose SSB	~55:32:13				
Intervention											80 (~13)	Sucrose	Sucrose SSB	~55:32:13				
Intervention											40 (~7)	Fructose	Fructose SSB	~51:35:14				
Control												Mixed	Reduce free fructose intake	~46:38:16				
**Engel et al. 2018**	45 OW/OB (16 M, 29 F)	OP, Denmark						P	Supp	Yes					Positive	6 mo	A, I	Unclear
Intervention	14 OW/OB (8 M, 6 F)		37.8 (8)	94.9 (11.6)	30.8 (2.8)	123.4 (8.9)	74.4 (9.7)				~106 (~21)	Sucrose	1 L/d sucrose-sweetened regular cola (50% glucose, 50% fructose)	54:31:13				
Control	16 OW/OB (5 M, 11 F)		39 (7.3)	98.4 (21.9)	31.5 (4.5)	124.2 (10.9)	74.9 (8.9)					Water	Water	46:34:15				
Control	15 OW/OB (3 M, 12 F)		39 (7.6)	94.5 (12.6)	33.4 (4.1)	131.5 (14.1)	81.2 (7.9)					NNS	1 L/d aspartame-sweetened NSB	46:34:15				
**Geidl-Flueck et al. 2021**	70 OW/OB (70 M, 0 F)	OP, Switzerland	22.7 (2.4)	71.5 (7.7)	21.8 (1.6)			P	Supp	Yes					Positive	7wk	A	No

Study, Year	Participants (M, F)	Setting	Mean Age, years*	BW, kg*	BMI, kg/m^2*	SBP, mmHg*	DBP, mmHg*	Design	Feeding Control [a]	Randomization	Dose, g/d (% E)[b]	Interventions	Food matrix	Diet [c]	E Balance [d]	F/U	Funding [e]	Med use
Intervention	23 OW/OB (23 M, 0 F)			69.2 (7.7)	21.2 (2.3)	122.6 (8.8)	67.3 (11.8)				79.8 (16.2)	Fructose	200ml fructose SSB consumed 3 times/day with meals	55:30:15				
Intervention	23 OW/OB (23 M, 0 F)			75.5 (7.3)	22.9 (1.4)	126.2 (7.2)	67.2 (8.1)				79.8 (14.3)	Sucrose	200ml sucrose SSB consumed 3 times/day with meals	52:33:16				
Control	24 OW/OB (24 M, 0 F)			70.4 (8.1)	21 (2.8)	127 (10.7)	69.7 (10.1)					Diet alone	Usual diet with abstinence of SSB intake, no supplementation	48:34:18				
**Hieronimus et al. 2020**	84 MW	OP/IP, USA						P	Supp+Met	No				55:30:15	Positive	2w	A	A
Intervention	28 MW (15 M, 13 F)		27 (6)	76 (13)	25 (4)	117.08 (9.96)	72.65 (7.22)				(25)	fructose	fructose as 25%E					
Intervention	22 MW (11 M, 11 F)		26 (5)	73 (15)	25 (4)	115.02 (8.73)	71.36 (4.87)				(17.5)	fructose	fructose as 17.5%E					
Intervention	28 MW (15 M, 13 F)		27 (7)	73 (15)	25 (4)	117.08 (9.75)	71.67 (6.51)				(25)	HFCS	HFCS as 25%E					
Intervention	16 MW (7 M, 9 F)		25 (5)	70 (14)	24 (3)	116.05 (10.07)	72.4 (6.77)				(17.5)	HFCS	HFCS as 17.5%E					
Control	23 MW (11 M, 12 F)		25 (6)	72 (11)	25 (3)	112.35 (11.49)	69.21 (8.57)					aspartame	aspartame					
**Njike et al. 2011**	39 OW (6 M, 33 F)	OP, USA	52.2 (10.6)					C	Supp	Yes			Sucrose		Positive	6 wk	A, I	Unclear
Intervention			52.2 (11.0)	179.9 (24.1)	30.5 (3.4)	123.8 (13.7)	68.3 (10.7)				91 (18.2)	Sucrose	Sugar-sweetened hot cocoa beverage	~55:29:15				
Intervention			51.9 (10.8)	179.5 (23.2)	30.3 (3.4)	123.6 (11.7)	67.3 (11.1)				110 (26)	Sucrose	Placebo beverage (no cocoa with sugar)	~54:30:16				
Control			52.5 (10.4)	178.9 (23.9)	30.2 (3.4)	122.8 (14.7)	68.6 (11.4)					NNS	Sugar-free hot cocoa beverage	~48:35:17				
**Sigala et al. 2020**	47 MW	IP/OP, USA		NR				P	Supp+Met	No				55:30:15	Positive	2 wk	A	
Intervention	24 MW (12M, 12F)		25.9 (6.3)		25.3 (3.4)	114.3 (8.4)	72.2 (5.5)				`(25)	Sucrose	25% Sucrose Koolaid					
Control	23 MW (11M, 12F)		25.4 (6.2)		24.8 (3.3)	112.35 (11.49)	69.2 (8.6)					NNS	Aspartame Koolaid					

Study, Year	Participants (M, F)	Setting	Mean Age, years*	BW, kg*	BMI, kg/m^2 *	SBP, mmHg*	DBP, mmHg*	Design	Feeding Control [a]	Randomization	Dose, g/d (% E)[b]	Interventions	Food matrix	Diet [c]	E Balance [d]	F/U	Funding [e]	Med use
**Silbernagel et al. 2011**	10 MW (7 M, 3 F)	OP, Germany	32.8 (9.3)	80.3 (9.1)	25.5 (2.2)			C	Supp	No				50:35: 15	Positive		A	No
Intervention						120 (12.6)	79 (9.5)				150 (~22.1)	Fructose	Fructose dissolved in water			4 wk		
Control						NR	NR					Diet alone	Usual diet, no beverage			2 wk		
**Stanhope et al. 2009**	17 OW/OB (9M, 8F)	IP/OP, USA	53 (40-27)	86.7 (10.7)	29.3 (2.6)			C	Met/Supp	No				55:30: 15	Neutral		A, I	No
Intervention						120 (8.2)	76 (4.1)				(25)	Fructose	25% E as fructose-sweetened Kool-Aid			10 wk		
Control						NR	NR					Diet alone	Usual diet, no beverage			2 wk		
**Stanhope et al. 2015**	41 MW (20 M, 21 F)							P	Met/Supp	No				55:30: 15	Positive	2 wk	A	No
Intervention	18 MW (9 M, 9 F)		28 (6)	70.9 (2.4)	24.9 (3.8)	114.88 (9.8)	72.8 (8.2)				`(10)	HFCS	10% HFCS Koolaid					
Control	23 MW (11 M, 12 F)		25 (6)	72 (11)	25 (3)	112.35 (11.49)	69.2 (8.6)					NNS	Aspartame Koolaid					
**100% Fruit Juice**																		
**Abedini et al. 2020**	42 PCOS (0M, 42F)	OP, Iran						P		Yes					Positive	8 wk	A	
Intervention	21 PCOS (0M, 21 F)		24.76 (5.2)	78.04 (12.1)	29.65 (3.2)	116.4±1.4/75.0±1.5 (SE)			Supp		24.75 (4.7)	Fruit	45mL/d concentrated pomegranate juice +180mL water, and routine recommendations for PCOS	56:30: 14				
Control	21 PCOS (0M, 21 F)		25.57 (5.0)	83.26 (12.2)	31.86 (5.3)	116.1±0.25/76.4±1.7 (SE)			DA			Diet alone	Routine recommendations for PCOS	59:27: 14				
**Aghababaee et al. 2015**	72 HLD (54M, 18F)	OP, Iran						P	Supp	Yes					Positive	8 wk	A	Unclear
Intervention	36 HLD		45.08 (7.5)	85.32 (16.67)	29.20 (4.45)	121.81 (9.64)	80.55 (5.31)				24.2 (3.7)	Fruit	300 mL Blackberry juice/d	~58:28 :14				
Control	36 HLD		45.61 (8.69)	78.91 (9.79)	28.02 (3.27)	120.69 (9.11)	81.11 (5.74)					Diet alone	Usual diet with berry restriction	~58:29 :13				

Study, Year	Participants (M, F)	Setting	Mean Age, years*	BW, kg*	BMI, kg/m^2 *	SBP, mmHg*	DBP, mmHg*	Design	Feeding Control [a]	Randomization	Dose, g/d (% E)[b]	Interventions	Food matrix	Diet [c]	E Balance [d]	F/U	Funding [e]	Med use
**Alatas et al. 2019**	112 pre-HTN	OP, indonesia	NR	NR	24.6 (4.16)			P	Supp	Yes				NR	Positive	3 mo	A	No
Intervention	54 pre-HTN					129.93 (10.83)	81.51 (9.75)				28 (5.6)	Fruit	Soursop 200g/d					
Control	58 pre-HTN					130.5 (8.85)	82.5 (7.68)					Diet alone	No soursop					
**Amagase et al. 2009**	60 MW	OP, China	NR					P	Supp	Yes				NR	Positive	4 wk	I	No
Intervention	30 MW			58.7 (5.3)	61.78 (9.69)	118.63 (13.4)	74.7 (8.0)				~12 (2.4)	Fruit	L. Barbarum (goji berry) juice 120mL/d					
Control	30 MW			59.05 (5.6)	60.91 (9.53)	118.33 (12.8)	73.6 (8.0)					NNS	Artificially sweetened placebo drink 120mL/d					
**Asgary et al. 2014**	21 HTN (6 M, 15 F)	OP, Iran						P	Supp	No				NR	Positive	2 wk	A	Unclear
Intervention	11 HTN (3 M, 8 F)		58.91 (5.06)	68 (10.05)	26.79 (3.47)	130.91 (13)	80 (8.94)				~24.6 (~4.9)	Fruit	150 mL/d pomegranate juice					
Control	10 HTN (3 M, 7 F)		46.90 (12.36)	73.5 (5.98)	27.95 (4.14)	128 (13.17)	85 (8.5)					Water	150 mL/d Water					
**Banini et al. 2006**	23 OW (11 M, 12 F)	OP, USA		NR				P	Supp	Yes				~50:31:19	Positive	4w	A, I	Unclear
Intervention	8 OW (3 M, 5 F)		50 (13)		29.3 (4.0)	127.8 (14.4)	76.8 (9.1)				22 (4)[k]	Fruit	150mL Muscadine grape juice					
Control	15 OW (8 M, 7 F)		56 (7.5)		27.5 (5.4)	123.1 (16.7)	71.1 (9.3)					Diet alone	No Muscadine grape juice					
**Esmaeilinezhad et al. 2020**	86 PCOS (0M, 86F)	OP, Iran		NR				P	Supp	Yes				NR	Positive	8 wk	A	Unclear
Intervention	22 PCOS (0M, 22F)		29.3 (7.46)		26.25 (2.93)	137 (7)	98 (8)				~46.9 (~9.4)	Fruit	2L/wk pom juice					
Intervention	22 PCOS (0M, 22F)		30.04 (6.39)		25.75 (2.45)	133 (10)	96 (11)				~46.9 (~9.4)	Fruit	2L/wk pom juice+inulin and probiotics					
Control	21 PCOS (0M, 21 F)		30.60 (7.43)		26.77 (1.7)	132 (6)	93 (5)					Water	2L/wk water+flavor +red food colouring					
Control	21 PCOS (0M, 21 F)		29.52 (5.82)		26.29 (1.70)	134 (9)	97(8)					Water	2L/wk water+flavor +red food colouring+inulin and probiotics					

Study, Year	Participants (M, F)	Setting	Mean Age, years*	BW, kg*	BMI, kg/m^2*	SBP, mmHg*	DBP, mmHg*	Design	Feeding Control [a]	Randomization	Dose, g/d (% E)[b]	Interventions	Food matrix	Diet [c]	E Balance [d]	F/U	Funding [e]	Med use
**Kojadinovic et al. 2017**	23 MS (0 M, 23 F)	OP, Serbia	(40-60)	NR				P	Supp	Yes				NR	Positive	6 wk	A	Unclear
Intervention	12 MS (0 M, 12 F)				31.98 (3.57)	133.33 (15.4)	82.78 (7.08)				39 (7.8)	Fruit	300 mL/d Polyphenol-rich pomegranate juice					
Control	11 MS (0 M, 11 F)				27.83 (7.84)	134.91 (13.88)	84.09 (11.23)					Water	300 mL/d water					
**Lazavi et al. 2018**	46 DM2 (19M, 27F)	OP, Iran						P	Supp	Yes					Positive	8 wk	A	Mixed
Intervention	23 DM2 (9M, 14F)		56.86 (8.47)	74.76 (11.22)	29.22 (3.98)	136.9 (21.7)	85.47 (13.95)				17 (2.9)	Fruit	200 mL/d Barberry juice/d	~56:24:20				
Control	23 DM2 (10M, 13F)		53.95 (6.57)	72.56 (9.90)	27.78 (3.45)	135.23 (11.67)	86.19 (8.5)					Diet alone	No barberry juice	~57:23:20				
**Neto et al. 2017**	26 HTN (9M, 17F)	OP, Brazil		NR				P	Supp	Yes				NR	Positive	4 wk	NR	Yes
Intervention	14 HTN (5M, 9F)		50.5 (3.74)		25.5 (1.87)	123.7 (12.7)	78 (11.2)				17.7 (3.5)[h]	Fruit	100 mL/d Whole red grape juice for women, 150mL/d for men					
Control	12 HTN (4M, 8F)		53.3 (3.46)		26.5 (2.08)	125.3 (7.97)	85 (5.89)					Diet alone	No grape juice					
**Ravn-Haren et al. 2013**	23 MW (9 M, 14 F)	OP, Denmark	36.2 (17.9)	NR	22.3 (2.6)	122 (15.5)	76.2 (11.3)	C	Supp	Yes				NR	Positive	4 wk	A	No
Intervention											63 (~12.6)	Fruit	Polyphenolic and pectin restricted diet with 500 mL clear apple juice /d					
Intervention											51 (~11.8)	Fruit	Polyphenolic and pectin restricted diet with 500 mL cloudy apple juice /d					
Control											2.1 (~0.004)	Diet alone	Polyphenolic and pectin restricted diet with 22 g apple pomace/d					

Study, Year	Participants (M, F)	Setting	Mean Age, years*	BW, kg*	BMI, kg/m^2 *	SBP, mmHg*	DBP, mmHg*	Design	Feeding Control [a]	Randomization	Dose, g/d (% E)[b]	Interventions	Food matrix	Diet [c]	E Balance [d]	F/U	Funding [e]	Med use
Control												Diet alone	Polyphenolic and pectin restricted diet					
**Shema-Didi et al. 2014**	65 Hemodialysis (35M, 30F)	IP, Israel	66.5 (11.8)	NR				P	Supp	Yes				NR	Positive	1 yr	A	Mixed
Intervention	44 Hemodialysis (NR)				27.9 (7.8)	145.6 (21.9)	70.9 (11.4)				≥ 5.7 (1.1)	Fruit	100 mL pomegranate juice, minimum 3x/wk					
Control	21 Hemodialysis (NR)				26.3 (5.2)	129.6 (27.2)	62.0 (15.7)					NNS	100 mL placebo (sweetened with NNS's), minimum 3x/week					
**Fruit**																		
**Amani et al. 2014**	19 DM2	OP, Iran	51.9 (8.2)	75.79 (9.02)	27.36 (4.23)			C	Supp	Yes				NR	Positive		A	No
Intervention						130.0 (14.9)	84.3 (8.0)				25 (5.6)[j]	Fruit	50g pure freeze dried strawberry powder with 2 cups of water, on top of a diet low in flavenoid-rich foods			6 wk		
Control						NR	NR					Diet alone	Diet low in flavenoid-rich foods			2 wk		
**Basu et al. 2010 (BB)**	48 MetS (4 M, 44 F)	OP, USA	49.8 (15.3)	NR	37.8 (11.2)	NR	NR	P	Supp	Yes				NR	Positive	8 wk	A, I	Mixed
Intervention	25 MetS (2 M, 23 F)		51.5 (15.0)		38.1 (7.5)						30 (~6)	Fruit	Freeze dried blueberry beverage					
Control	23 MetS (2 M, 21 F)		48.0 (15.8)		37.5 (14.4)							Water	Water					
**Basu et al. 2010 (SB)**	27 MetS (2 M, 25 F)	OP, USA	46.7 (16.6)	102.3 (9.5)	37.8 (8.9)			P	Supp	Yes					Neutral	8 wk	A, I	Mixed
Intervention	15 MetS (0 M, 15 F)		48.0 (20.5)	102.0 (11.6)	39.0 (7.7)	135 (14.3)	85 (12.4)				~14.6 (~3.2)[j]	Fruit	Freeze dried strawberry beverage	45:37:13				
Control	12 MetS (2 M, 10 F)		45.0 (10.4)	102.7 (6.6)	36.4 (10.4)	130 (9.0)	80 (7.3)					Water	Water	46:35:15				

Study, Year	Participants (M, F)	Setting	Mean Age, years*	BW, kg*	BMI, kg/m^2*	SBP, mmHg*	DBP, mmHg*	Design	Feeding Control [a]	Randomization	Dose, g/d (% E)[b]	Interventions	Food matrix	Diet [c]	E Balance [d]	F/U	Funding [e]	Med use
**Cressey et al. 2014 (DM2)**	15 DM2	OP, Thailand	52.8 (5.23)					C	Supp	No					Positive		A	Unclear
Intervention				61.8 (13.3)	25.8 (4.7)	134 (21.4)	78 (7.6)				~30.6 (5.6)	Fruit	1 banana/d (250 g)	~57:25:18		4 wk		
Control				62.3 (13.0)	25.9 (4.6)	NR	NR					Diet alone	No banana	~53:29:19		8 wk		
**Cressey et al. 2014 (HCL/HD)**	15 HCL	OP, Thailand	43.1 (7.5)					C	Supp	No					Positive		A	Unclear
Intervention				59.6 (11.8)	24.0 (3.94)	124 (12.0)	78 (10.5)				~61.2 (~10.6)	Fruit	2 banana/d (500 g)	~57:26:17		12 wk		
Control				59.3 (12.1)	24.1 (4.2)	NR	NR					Diet alone	No banana	~49:34:17		8 wk		
**Cressey et al. 2014 (HCL/LD)**	15 HCL	OP, Thailand	44.8 (10.3)					C	Supp	No					Positive		A	Unclear
Intervention				61.5 (10.9)	24.8 (4.0)	127 (9.3)	82 (11.9)				~30.6 (5.9)	Fruit	1 banana/d (250 g)	~56:27:17		12 wk		
Control				61.5 (10.7)	24.8 (4.3)	NR	NR					Diet alone	No banana	~47:35:17		8 wk		
**Dow et al. 2012**	71 OW/OB (17 M, 54 F)	OP, USA	41.2 (11)	91.7 (13.6)	32.1 (4.1)	119.1 (16.6)	80.6 (10.5)	P	Supp	Yes					Positive	6 wk	A	Unclear
Intervention	39 OW/OB (19 M, 29 F)					118.4 (16.9)	80 (10.2)				~24 (4.8)[j]	Fruit	~450 g grapefruit/d (1/2 fruit 3x/d)	~50:34:16				
Control	32 OW/OB (7 M, 25 F)					119.1 (15.9)	81.3 (11.1)					Diet alone	Low fruit/vegetable diet (restricted fruits and veg with high polyphenol and carotenoid content)	~48:37:16				
**Franck et al. 2020**	48 risk of MetS (16M, 32F)	OP, Canada		NR				P	Supp	Yes					Positive	8 wk	I	No
Intervention	24 (7M, 17F)		32.46 (10.12)		30.42 (5)	112.8±1 1.0	71.9 (8.9)				12.4 (2.4)	Fruit	280g frozen raspberries (2 cups)/d	48:35:17				
Control	24 (9M, 15F)		31.92 (8.05)		29.38 (3.94)	110.8±1 1.2	68.9 (8.7)					Diet alone	Usual diet, no supplementation	42:40:18				

Study, Year	Participants (M, F)	Setting	Mean Age, years*	BW, kg*	BMI, kg/m^2*	SBP, mmHg*	DBP, mmHg*	Design	Feeding Control [a]	Randomization	Dose, g/d (% E)[b]	Interventions	Food matrix	Diet [c]	E Balance [d]	F/U	Funding [e]	Med use
**Karlsen et al. 2013 (optimal BP)**	23 Optimal BP (23M, 0F)	OP, Norway						P	Supp	Yes					Positive	8 wk	A	Mixed
Intervention	12 Optimal BP (12M, 0F)		57 (45-73)[f]	76.8 (55.0-98.2)	24.7 (18.8-32.2)	111 (89-119)	74 (64-79)				13.3 (2.7)[g]	Fruit	195 g/d Kiwi	~43:35:16				
Control	11 Optimals BP (11M, 0F)		56 (44-71)[f]	83.7 (66.2-99.8)	24.8 (21.6-30.4)	112 (108-117)	75 (70-79)					Diet alone	No kiwi	~42:34:16				
**Karlsen et al. 2013 (normal/high-normal BP)**	24 Normal/High BP (24M, 0F)	OP, Norway						P	Supp	Yes					Positive	8 wk	A	Mixed
Intervention	12 Normal/High (12M, 0F)		57 (45-73)	76.8 (55.0-98.2)	24.7 (18.8-32.2)	127 (120-132)	84 (80-89)				13.3 (2.7)[g]	Fruit	195 g/d Kiwi	~43:35:16				
Control	12 Normal/High BP (12M, 0F)		56 (44-71)	83.7 (66.2-99.8)	24.8 (21.6-30.4)	127 (120-135)	82 (80-89)					Diet alone	No kiwi	~42:34:16				
**Karlsen et al. 2013 (HTN)**	20 HTN (20M, 0F)	OP, Norway						P	Supp	Yes					Positive	8 wk	A	Mixed
Intervention	9 HTN (9M, 0F)		57 (45-73)	76.8 (55.0-98.2)	24.7 (18.8-32.2)	153 (140-175)	99 (90-110)				13.3 (2.7)[j]	Fruit	195 g/d Kiwi	~43:35:16				
Control	11 HTN (11M, 0F)		56 (44-71)	83.7 (66.2-99.8)	24.8 (21.6-30.4)	153 (140-187)	98 (91-102)					Diet alone	No kiwi	~42:34:16				
**Kumari et al. 2016**	45 MW (31 M, 14 F)	OP, India	18-25					P	Supp	Yes				NR	Positive	6 wk	A	No
Intervention	15 MW			59 (8.9)	21.8 (2.4)	123 (8)	77 (8)				~35.7 (7.1)[j]	Fruit	400 g/d guava with peel					
Intervention	15 MW			63 (10.8)	22.2 (2.8)	124 (10)	76 (9)				~35.7 (7.1)[j]	Fruit	400 g/d guava without peel					
Control	15 MW			62 (11.9)	21.7 (3.1)	114 (10)	72 (7)					Diet alone	No guava					
**Ravn-Haren et al. 2013**	23 MW (9 M, 14 F)	OP, Denmark	36.2 (17.9)	NR	22.3 (2.6)	122 (15.5)	76.2 (11.3)	C	Supp	Yes				NR	Positive	4 wk	A	No

Study, Year	Participants (M, F)	Setting	Mean Age, years*	BW, kg*	BMI, kg/m^2*	SBP, mmHg*	DBP, mmHg*	Design	Feeding Control [a]	Randomization	Dose, g/d (% E)[b]	Interventions	Food matrix	Diet [c]	E Balance [d]	F/U	Funding [e]	Med use
Intervention											51 (~10.2) [q]	Fruit	Polyphenolic and pectin restricted diet with 550 g whole apples/d					
Control											2.1 (~0.004)	Diet alone	Polyphenolic and pectin restricted diet with 22 g apple pomace/d					
Control												Diet alone	Polyphenolic and pectin restricted diet, no apple					
**Schell et al. 2019**	22 DM2 (5 M, 20 F)	OP, USA	54 (21)	104 (55)	35.3 (10)	151 (27.5)	94 (15)	P	Supp	Yes					Positive	4 wk	A, I	Mixed
Intervention											11.05 (2.21)	Fruit	250 g frozen raspberries per day	48:34:17				
Control												Diet alone	No raspberries	45:37:16				
**Dried fruit**																		
**Ahmed et al. 2010**	70 MW	OP, Pakistan	43 (10)	NR	NR			P	Supp	Yes				NR	Positive	8 wk	NR	Unclear
Intervention	35 MW					131.83 (5.3)	108.66 (22.04)				~8.8 (1.8)	Dried fruit	Dried prunes (22.86 g, 6 prunes) soaked in water overnight with mixture consumed in the morning					
Control	35 MW					133.11 (11.7)	86.98 (6.1)					Water	Water					
**Al- Dashti et al. 2019**	27 MW (0 M, 27 F)	OP, USA	59 (5.2)	NR	24.5 (4.2)	115 (15.6)	75 (10.4)	C	Supp	Yes				NR	Positive	2 wk	A, I	No
Intervention											~16 (3.8)	Dried fruit	~42g of prunes incorporated in usual diet					

Study, Year	Participants (M, F)	Setting	Mean Age, years*	BW, kg*	BMI, kg/m^2*	SBP, mmHg*	DBP, mmHg*	Design	Feeding Control [a]	Randomization	Dose, g/d (% E)[b]	Interventions	Food matrix	Diet [c]	E Balance [d]	F/U	Funding [e]	Med use
Intervention											~5.3 (1.2)	Dried fruit	~14g of prunes incorporated in usual diet					
Control												Diet alone	Usual diet - run-in phase					
Control												Diet alone	Usual diet - washout phase					
**Irannejad et al. 2020**	72 DM2 (21 M, 51 F)	OP, Iran						P	Supp	Yes					Positive	12 w	A	
Intervention	36 DM2 (8 M, 28 F)		53 (8)	76 (13)	29 (4)	133.85 (15.81)	88.19 (10.96)				~14.3 (3.8)	fruit	30g/d z. vulgaris	70:18:16				
Control	36 DM2 (13 M, 23 F)		57 (6)	75 (11)	28 (4)	123.85 (12.66)	86.80 (17.4)					diet alone	diet alone	69:17:15				
**Sweetened cereal grains and bars**																		
**Davidi et al. 2011**	94 OW (20 M, 64 F)	OP, USA						P	Supp	Yes				NR	Positive	8 wk	A	Mixed
Intervention	49 OW (11 M, 38 F)		52.6 (13)	89.2 (17.3)	32.7 (5.3)	125.9 (10.8)	75.6 (7.1)				24 (5)	Mixed	Sweetened grain/nut bar					
Control	45 OW (9 M, 36 F)		54.8 (15.7)	94.6 (20.4)	34.7 (5.3)	129.2 (12.8)	74.3 (9.1)					Usual diet	Baseline diet					
**Mietus-Snyder et al. 2012**	25 MW (10M, 15F)	OP, USA	44.9 (15.8)	NR	25.7 (14.3)	NR	NR	C	Supp	No				NR	Positive	2 wk	A, I	Unclear
Intervention											15.2 (3)	Mixed	2 fruit & nut bars/d					
Control												Diet alone	No fruit & nut bars					
**Sweets and desserts**																		
**Ayoobi et al. 2017**	44 DM2 (17 M, 27 F)	OP, Iran	52 (6)	NR	NR	NR	NR	P	Supp	Yes					Positive	8w	A	
Intervention	21 DM2 (7 M, 14 F)		51 (8)								~5.5 (~1.1)	sucrose	84% dark chocolate	56:27:18				
Control	23 DM2 (10 M, 13 F)		51 (8)									diet alone	no chocolate	58:23:19				
**Leskinen et al. 2014**	50 MW (21 M, 29 F)	OP, Finland						P	Supp	No				NR	Positive	2 wk	A, I	No

Study, Year	Participants (M, F)	Setting	Mean Age, years*	BW, kg*	BMI, kg/m^2 *	SBP, mmHg*	DBP, mmHg*	Design	Feeding Control [a]	Randomization	Dose, g/d (% E)[b]	Interventions	Food matrix	Diet [c]	E Balance [d]	F/U	Funding [e]	Med use
Intervention	20 MW (8 M, 12 F)		33.6 (2)	70 (9)	23.3 (1.9)	120 (8)	71 (8)				~88 (17.6)	Sucrose	~50-60g/d d Liquorice (Halva or Kouvola brand)p providing 290-370mg glycyrrhizin					
Control	30 MW (13 M, 17 F)		33.8 (8.1)	71 (9)	23.0 (2.7)	116 (14)	72 (11)					Diet alone	No liquorice					
**Martini et al. 2021**	21 NW (11F, 10M)	OP, Italy	22.9 (2.3)	67 (12.4)	22.3 (7.8)	116.6 (7.8)	73.7 (30.4)	C	Supp	Yes					Positive	4 wk	I	
Intervention											43.4 (8.2)	Sucrose	1 cup espresso + 2 cocoa-based products containing coffee/d	47:37:16				
Control												Water	1 cup espresso/d	49:38:13				
**Added nutritive (caloric) sweetener**																		
**Tang et al. 2020**	95 HIV (81 M, 14 F)	IP, Malaysia	(21-58)					P	Met	Yes				NR	Positive	6 mo	A	
Intervention	26 HIV			56 (8)	22 (3)	117.31 (12.9)	71.50 (8.1)				16.4 (3.2)	honey	Tualang honey (20g/d)					
Intervention	24 HIV			59 (8)	22 (3)	112.42 (10.8)	70.92 (7.9)				32.8 (6.2)	honey	Tualang honey (40g/d)					
Intervention	22 HIV			55 (7)	21 (2)	111.55 (12.9)	70.59 (8.5)				49.3 (9.0)	honey	Tualang honey (60g/d)					
Control	23 HIV			58 (7)	23 (3)	118.95 (16.4)	75.59 (12)					diet alone	No honey					
**Mixed sources (with SSBs)**																		
**Raben et al. 2002**	41 OW/OB (6M, 35F)	OP, Denmark						P	Supp	Yes					Positive	10 wk	A, I	Unclear
Intervention	21 OW/OB (NR)		33.3 (9.17)	82.5 (7.79)	28.0 (2.29)	119 (11.5)	72.6 (9.62)				~160 (23)	Sucrose	Sucrose-sweetened foods: soft drinks, fruit juices, yogurt, marmalade, ice cream, stewed fruit.	~57:29:11				

Study, Year	Participants (M, F)	Setting	Mean Age, years*	BW, kg*	BMI, kg/m^2*	SBP, mmHg*	DBP, mmHg*	Design	Feeding Control [a]	Randomization	Dose, g/d (% E)[b]	Interventions	Food matrix	Diet [c]	E Balance [d]	F/U	Funding [e]	Med use
Control	20 OW/OB (NR)		37.1 (9.84)	79.2 (8.94)	27.6 (2.24)	115.8 (8.49)	72.8 (8.94)					NNS	Artificially sweetened foods: soft drinks, fruit juices, yogurt, marmalade, ice cream, stewed fruit.	~45:33:15				
**Subtraction Trials**																		
**SSB**																		
**Al-Dujaili et al. 2017**	16 MW (8M, 8F)	OP, UK	27.75 (13.75)	74.82 kg (16.59)	26.33 (5.26)	114.5 (12.7)	70.8 (9.4)	C	Supp	Yes				NR	Negative	1 wk	NR	No
Intervention												NNS	Stevia added to hot beverages					
Control											15 (3)	Sucrose	Sugar added to hot beverages					
**Campos et al. 2015 (G1)**	12 OW/OB, IHCL<60 mmol/l (3 M, 9 F)	OP, Switzerland	28.3 (6.5)					P	Supp	Yes					Negative	3 mo	A	No
Intervention	6 OW/OB			85.6 (11.3)	30.1(4.9)	115.3 (15.9)	70 (11.5)					NNS	Replace SSB with NSB	~46:38:16				
Control	6 OW/OB			78.5 (7.1)	26.9(1.2)	118.2 (11.3)	66.9 (8.3)				86.8 (~15)	Sucrose	Usual SSB consumption (≥ 2 SSB/d)	~51:34:15				
**Campos et al. 2015 (G2)**	15 OW/OB, IHCL>60 mmol/l (11 M, 4 W)	OP, Switzerland	29.1 (6.9)					P	Supp	Yes					Negative	3 mo	A	No
Intervention	7 OW/OB			102.2 (12.2)	32.5 (4.5)	127.8 (17.0)	74.7 (10.2)					NNS	Replace SSB with NSB	~46:38:16				
Control	8 OW/OB			100.0 (11.6)	33.8(5.6)	127 (10.8)	70.6 (5.6)				86.8 (~15)	Sucrose	Usual SSB consumption (≥ 2 SSB/d)	~51:34:15				
**Hernandez-Cordero et al. 2014**	240 OW/OB (0 M, 240 F)	OP, Mexico						P	DA/Supp	Yes				NR	Negative	9 mo	A, I	No

Study, Year	Participants (M, F)	Setting	Mean Age, years *	BW, kg*	BMI, kg/m^2 *	SBP, mmHg*	DBP, mmHg*	Design	Feeding Control [a]	Randomization	Dose, g/d (% E)[b]	Interventions	Food matrix	Diet [c]	E Balance [d]	F/U	Funding [e]	Med use
Intervention	120 OW/OB (0 M, 120 F)		33.5 (6.7)	76.9 (3.3)	31.0 (1.1)	100 (3.3)	68.3 (8.7)					Water	Substitute water for SSBs, general recommendations for healthy eating					
Control	120 OW/OB (0 M, 120 F)		33.4 (6.7)	76.0 (3.3)	31.0 (1.1)	102 (3.3)	70 (7.7)				~102.7 (20)	Sucrose	Usual SSB consumption (≥250 kcal/d), general recommendations for healthy eating					
**Tate et al. 2012**	246 OW/OB	OP, USA						P	DA/Supp	Yes				NR	Negative	6 mo	I	No
Intervention	84 OW/OB		41.2 (11.2)	100.9 (15.7)	36.1 (6.2)	125.9 (13.8)	82.1 (9.1)				~33.7 (~8.7)	NNS	≥2 SSB/d replaced with NSB					
Intervention	87 OW/OB		43.2 (10.6)	98.4 (17.0)	35.8 (5.2)	125.2 (14.8)	80.5 (9.3)				~33.7 (~8.7)	Water	≥2 SSB/d replaced with water					
Control	75 OW/OB		41.6 (10.4)	102.6 (18.3)	36.8 (6.2)	124.3 (10.3)	80.6 (6.7)				~55.7 (~13.8)	HFCS	Usual SSB consumption of ≥280 kcal/d					
**Ebbeling et al. 2020**	186 MW	OP, USA						P	Supp	Yes				NR	Negative	12 mo	A, I	No
Intervention	60 MW (36 M, 24 F)		26.7 (5.7)	76.8 (16.7)	26.1 (5.2)	109.1 (13.5)	66.6 (8.1)					NNS	NSB replacing usual SSB intake					
Intervention	66 MW (39 M, 27 F)		27.9 (6.0)	77.5 (16.1)	26.6 (4.6)	105.7 (9.7)	67.7 (7.4)					Water	Water replacing usual SSB intake					
Control	60 MW (36 M, 24 F)		25.9 (5.1)	75.5 (15.6)	25.8 (4.7)	108 (8.8)	66.6 (7.6)				~84.5 (15.3)	HFCS	Usual beverage intake, no NSB					
**Mixed sources (with SSBS)**																		
**Vazquez-Duran et al. 2016**	146 MW (26M, 120F)	OP, Mexico	21.99 (0.25)	66.78 kg (1.10)	26.24 (0.36)	109.49 (0.93)	71.91 (0.71)	P	Met	Yes	NR			NR	Negative	6 mo	NR	Unclear
Intervention	48 MW		22.55 (0.51)	69.13 kg (2.11)	27.27 (0.70)	110.53 (1.78)	71.45 (1.22)					Water	Allowed only water, coffee or tea with no sugar					
Intervention	49 MW		21.46 (0.31)	65.48 kg (1.92)	25.48 (0.62)	107.56 (1.59)	71.3 (1.26)					NSB	Allowed only NSB, water, coffee or tea with no sugar					
Control	49 MW		21.98 (0.45)	65.75 kg (1.67)	26.00 (0.55)	110.43 (1.44)	73 (1.2)					Sucrose	Usual SSB intake (a variety of sweetened drinks, including juices)					

Study, Year	Participants (M, F)	Setting	Mean Age, years*	BW, kg*	BMI, kg/m^2 *	SBP, mmHg*	DBP, mmHg*	Design	Feeding Control [a]	Randomization	Dose, g/d (% E)[b]	Interventions	Food matrix	Diet [c]	E Balance [d]	F/U	Funding [e]	Med use
***Ad libitum* Trials**																		
**Mixed sources (with SSBs)**																		
**Markey et al. 2016**	50 MW (16 M, 34 F)	OP, UK	31.6 (9.5)	69.8 (11.4)	24.0 (3.3)			C	Supp	Yes					Neutral	8 wk	I	No
Intervention	22 MW (7 M, 15 F)		31.6 (10.2)	70.5 (13.1)	24.2 (3.3)	116 (11)	71 (7)				~30 (~6)	Sucrose	Exchange ≥1 food portion and ≥1 beverage per day from habitual diet with sugar containing products	54:30: 14				
Control	28 MW (9 M, 19 F)		31.1 (9.2)	69.3 (10.1)	23.9 (3.4)	116 (12)	72 (8)					NNS	Exchange ≥1 food portion and ≥1 beverage per day from habitual diet with sugar reformulated products	48:33: 15				
**Poppitt et al. 2002**	39 MetS (12M, 27F)	OP, UK						P	Supp	Yes					Neutral	6 mo	A, I	Unclear
Intervention	14 MetS (6 M, 8 F)		45.9 (5.0)	89.3 (15.7)	30.9 (3.0)	138 (22)	84 (13)				~165.4 (29)[n]	Sucrose	*Ad libitum* low-fat SCHO diet, including SSB	~59:20 :22				
Control	11 MetS (1M, 10F)		48.6 (4.4)	91.4 (9.2)	33.1 (3.3)	132 (14)	87 (10)					Mixed comparator	*Ad libitum* usual diet, with no restrictions on beverages	49:30: 20				
Control	14 MetS (5 M, 9 F)		44.2 (5.5)	91.2 (9.5)	32.3 (3.6)	136 (17)	86 (13)					Starch	*Ad libitum* low fat CCHO diet, with NSB or ASB only	53:23: 24				

Study, Year	Participants (M, F)	Setting	Mean Age, years*	BW, kg*	BMI, kg/m2 *	SBP, mmHg*	DBP, mmHg*	Design	Feeding Control [a]	Randomization	Dose, g/d (% E)[b]	Interventions	Food matrix	Diet [c]	E Balance [d]	F/U	Funding [e]	Med use
**Raben et al. 1997 (PO)**	9 PO (0 M, 9 F)	OP, Denmark	39 (9.0)	63.9 (5.4)	22.9 (2.1)	NR	NR	C	Met	No					Neutral	2 wk	A, I	Unclear
Intervention											(23)	Sucrose	*Ad libitum* sucrose diet, including SSB	59:28:13				
Control											(2)	Starch	*Ad libitum* starch diet, without SSB	59:28:13				
Control											(2)	Fat	*Ad libitum* fat diet, without SSB	41:46:13				
**Raben et al. 1997 (H)**	11 MW (0 M, 11 F)	OP, Denmark	38 (9.9)	61.9 (4.0)	22.6 (1.3)	NR	NR	C	Met	No					Neutral	2 wk	A, I	Unclear
Intervention											(23)	Sucrose	*Ad libitum* sucrose diet, including SSB	59:28:13				
Control											(2)	Starch	*Ad libitum* starch diet, without SSB	59:28:13				
Control											(2)	Fat	*Ad libitum* fat diet, without SSB	41:46:13				

[a]Metabolic feeding control included provision of all study foods, supplement feeding control included provision of study supplements only, and dietary advice included dietary counseling without the provision of any dietary foods or supplements.
[b]Doses preceded by "~" represent approximate amounts calculatd on the basis of average body weight or energy intake reported by participants. In the absence of this data, an average of 70 kg body weight or 2000 kcal/d was assumed.
[c]Total energy intake in the form of carbohydrate:fat:protein.
[d]Positive energy balance included interventions designed to consume excess calories on top of a baseline diet. Negative energy balance included interventions designed to create a caloric deficit compared to the baseline diet. Neutral energy balance included interventions designed to continue habitual caloric intake.
[e]Agency funding included government, not-for profit health agencies or University sources.
[f]Z-scores reported fro pediatric studies
[g]Fructose-containing sugar dose estimated based on Mandasoy products (plain soymilk)
[h]Fructose-containing sugar dose estimated based on data from United States Department of Agriculture (USDA) nutrient database
[i]Fructose-containing sugar dose estimated based on data from the Finland National Food Composition Database
[j]Fructose-containing sugar dose estimated based on data from the Canadian Nutrient File
[k]Data based on baseline participants, including drop-outs.
[l]Fructose-containing sugar dose estimated based on data from the Nutritionix Database (UK region)
[m]Although honey roasted peanuts were provided as the intervention, sucrose was the main sugar used to sweeten the study products
[n]Fructose-containing sugar dose estimated from simple carbohydrate intake
A=agency; BB=blueberry; C=crossover; CVD=cardiovascular disease; DA=dietary advice; DM2= diabetes mellitus type 2; E=energy F=female; F/U=follow-up; H=healthy; HD=high dose; HCL=hypercholesterolemic; HFCS=high fructose corn syrup; HI=hyperinsulinemic; HLD=hyperlipidemic; HTN=hypertensive; I=industry; IGT=impaired glucose tolerance; LD=low dose; IP=inpatient; M=male; Med=medication; Met=metabolic; MetS=metabolic syndrome; mo=months; MW=mixed weight; NAFLD=non-alcoholic fatty liver disease; NGT=normal glucose tolerance; NNS=non-nutritive sweetener; NR=not reported; NSB=non-nutritive sweetened beveragae; NW=normal weight; OB=obese; OP=outpatient; OW=overweight; P=parallel; PDM=pre-diabetes mellitus, PCOS=polycystic ovarian syndrome; PO=post-obese; SB=strawberry; SSB=sugar sweetened beverage; Supp=supplemented; wk=weeks

S6 Table. Sensitivity analyses of the use of correlation coefficients of 0.25 and 0.75 for crossover trials in the primary analysis of the effect of important food sources of fructose-containing sugars on systolic and diastolic blood pressure (mmHg) and by important food sources of fructose-containing sugars.

**Blood Pressure Outcome**	MD (95% CI), P-value<br>**I^2, P-value**		
	Correlation Coefficient<br>**used in the Primary Analysis**	Correlation Coefficient used in<br>**Sensitivity Analyses**	
**Energy Design and Food Source (no. crossover trials/total)**	**0·5**	**0·25**	**0·75**
**SBP (mmHg)**			
**Substitution (34/72)**	**-0·33 [-1·16, 0·51], p_{MD}=0·445<br>I^2=39·44%, p_Q=0·001**	**-0·30 [-1·16, 0·56], p_{MD}=0·494<br>I^2=37·84%, p_Q=0·001**	**-0·26 [-1·05, 0·54], p_{MD}=0·527<br>I^2=49·24%, p_Q<0.001**
SSB (8/16)	0·39 [-0·86, 1·64], p_{MD}=0·538<br>I^2=4·04%, p_Q=0·538	0·48 [-0·78, 1·34], p_{MD}=0·456<br>I^2=0·00%, p_Q=0·500	0·28 [-1·03, 1·59], p_{MD}=0·673<br>I^2=22·59%, p_Q=0·197
Sweetened dairy (0/3)	2·18 [-0·49, 4·85], p_{MD}=0·110<br>I^2=0·00, p_Q=0·694	**NA**	**NA**
Sweetened dairy alternatives (soy) (0/1)	-2·33 [-5·89, 1·23], p_{MD}=0·119<br>I^2=·, p_Q=·	**NA**	**NA**
Fruit (4/11)	-2·13 [-4·79, 0·53], p_{MD}=0·116<br>I^2=73·97, p_Q<0.001	-2·24 [-4·96, 0·48], p_{MD}=0·106<br>I^2=73·64%, p_Q<0.001	-1·95 [-4·41, 0·51], p_{MD}=0·121<br>I^2=74·94%, p_Q<0.001
Dried Fruit (2/8)	-2·22 [-4·99, 0·55], p_{MD}=0·116<br>I^2=32·94, p_Q=0·165	-2·22 [-5·04, 0·60], p_{MD}=0·122<br>I^2=32·86%, p_Q=0·166	-2·22 [-4·87, 0·44], p_{MD}=0·102<br>I^2=33·19, p_Q=0·163
Mixed fruit forms (0/1)	-1·66 [-6·85, 3·65], p_{MD}=0·550<br>I^2=·, p_Q=·	**NA**	**NA**
Sweetened fruit and nut bars (0/1)	4·92 [-0·96, 10·43], p_{MD}=0·080<br>I^2=·, p_Q=·	**NA**	**NA**
Sweets and desserts (6/8)	-1·29 [-3·47, 0·90], p_{MD}=0·248<br>I^2=0·00%, p_Q=0·817	-1·28 [-3·67, 1·12], p_{MD}=0·297<br>I^2=0·00%, p_Q=0·928	-1·31 [-3·11, 0·49], p_{MD}=0·153<br>I^2=1·43%, p_Q=0·418
Added nutritive (caloric) sweetener (7/7)	-1·67 [-4·00, 0·65], p_{MD}=0·159<br>I^2=0·00%, p_Q=0·855	-1·60 [-4·40, 1·19], p_{MD}=0·261<br>I^2=0·00%, p_Q=0·935	-1·85 [-3·56, -0·13], **p_{MD}=0·035**<br>I^2=0·00%, p_Q=0·587
Mixed sources (with SSBs) (4/13)	1·48 [-0·91, 3·87], p_{MD}=0·224<br>I^2=42·94%, p_Q=0·050	1·54 [-0·91, 3·98], p_{MD}=0·219<br>I^2=40·21%, p_Q=0·066	1·41 [-0·91, 3·72], p_{MD}=0·234<br>I^2=49·61%, p_Q=0·022
Mixed sources (without SSBs) (3/3)	2·56 [-0·24, 5·36], p_{MD}=0·073<br>I^2=0·00%, p_Q=0·949	2·75 [0·44, 5·07], **p_{MD}=0·020**<br>I^2=0·00%, p_Q=0·955	2·63 [0·87, 4·40], **p_{MD}=0·004**<br>I^2=0·00%, p_Q=0·889
**Addition (24/64)**	**-2·23 [-3·40, -1·06], p_{MD}<0.001<br>I^2=69·32%, p_Q<0.001**	**-2·24 [-3·46, -1·03], p_{MD}<0·001<br>I^2=68·45%, p_Q<0.001**	**-2·22 [-3·23, -1·30],p_{MD}<0·001<br>I^2=71·45%, p_Q<0.001**
SSB (7/17)	0·74 [0·42, 1·90], p_{MD}=0.209<br>I^2=0·00%, p_Q=0·789	0·89 [-0·33, 2·10], p_{MD}=0·152<br>I^2=0·00%, p_Q=0·908	0·40 [-0·75, 1·56], p_{MD}=0·492<br>I^2=8·00%, p_Q=0·361
100% Fruit juice (4/16)	-3·74 [-5·28, -2·20], p_{MD}<0.001<br>I^2=2·15%, p_Q=0·006	-3·85 [-5·42, -2·27], p_{MD}<0·001<br>I^2=50·63%, p_Q=0·011	-3·59 [-5·08, -2·09], p_{MD}<0·001<br>I^2=59·46%, p_Q=0·001
Fruit (7/16)	-6·37 [-9·21, -3·52], p_{MD}<0·001<br>I^2=68·65%, p_Q<0·001	-6·47 [-9·33, -3·61], p_{MD}<0·001<br>I^2=63·76%, p_Q<0·001	-6·28 [-9·05, -3·50], p_{MD}<0·001<br>I^2=75·61%, p_Q<0·001
Dried fruit (4/6)	-1·96 [-4·69, 0·77], p_{MD}=0·160<br>I^2=0·00%, p_Q=0·970	-2·00 [-5·14, 1·14], p_{MD}=0·211<br>I^2=0·00%, p_Q=0·980	-1·90 [-3·99, 0·20], p_{MD}=0·076<br>I^2=0·00%, p_Q=0·930
Sweetened cereal grains and bars (1/2)	-0·68 [-3·78, 2·43], p_{MD}=0·669<br>I^2=0·00, p_Q=0.488	-0·68 [-3·78, 2·43], p_{MD}=0·669<br>I^2=0·00%, p_Q=0·488	-0·68 [-3·78, 2·43], p_{MD}=0·669<br>I^2=0·00%, p_Q=0·488
Sweets and desserts (1/3)	1·62 [-0·92, 4·17], p_{MD}=0·211<br>I^2=81·80, p_Q=0·005	-1·62 [-9·20, 4·17], p_{MD}=0·211<br>I^2=81·80%, p_Q=0·005	1·13 [-1·13, 3·38], p_{MD}=0·327<br>I^2=82·23%, p_Q=0.004
Added nutritive (caloric) sweetener (0/3)	4·58 [-2·05, 11·20], p_{MD}=0·176<br>I^2=0·00%, p_Q=0·891	NA	NA

Mixed sources (with SSBs) (0/1)	6·90 [2·23, 11·58], p_{MD}=0.004<br>I^2=·%, p_Q=·	··	··
**Subtraction (1*/10)**	**-1·16 [-2·90, 0·57], p_{MD}=0·188<br>I^2=82.23%, p_Q<0.001**	**NA**	**NA**
SSB (1*/8)	-0·47 [-2·70, 1·75], p_{MD}=0·676<br>I^2=49.72%, p_Q=0.053	NA	NA
Mixed sources (with SSBs) (0/2)	-2·26 [-2·79, 1·73], p_{MD}<0·001<br>I^2=96.69%, p_Q<0.001	NA	**NA**
***Ad libitum* (5/6)**	**0·98 [-0·43, 2·39], p_{MD}=0·174<br>I^2=0·00%, p_Q=0·902**	**0·99 [-0·42, 2·41], p_{MD}=0·169<br>I^2=0·00%, p_Q=0·952**	**0·92 [-0·47, 2·30], p_{MD}=0·194<br>I^2=0·00%, p_Q=0·726**
Mixed sources (with SSBs) (5/6)	0·98 [-0·43, 2·39], p_{MD}=0·174<br>I^2=0·00%, p_Q=0·902	0·99 [-0·42, 2·41], p_{MD}=0·169<br>I^2=0·00%, p_Q=0·952	0·92 [-0·47, 2·30], p_{MD}=0·194<br>I^2=0·00%, p_Q=0·726
**DBP (mmHg)**			
**Substitution (34/72)**	**0·12 [-0·53, 0·78], p_{MD}=0·714<br>I^2=59·09%, p_Q<0.001**	**0·03 [-0·65, 0·70], p_{MD}=0·942<br>I^2=56·30%, p_Q<0.001**	**0·26 [-0·36, 0·0], p_{MD}=0·401<br>I^2=64·87%, p_Q<0.001**
SSB (8/17)	0·66 [-0·29, 1·61], p_{MD}=0·174<br>I^2=0·00%, p_Q=0·950	0·72 [-0·34, 1·77], p_{MD}=0·183<br>I^2=0·00%, p_Q=0·966	0·57 [-0·20, 1·34], p_{MD}=0·149<br>I^2=0·00%, p_Q=0·889
Sweetened dairy (0/3)	-0·47 [-2·85, 1·91], p_{MD}=0·697<br>I^2=0·00, p_Q=0·754	NA	NA
Sweetened dairy alternatives (soy) (0/1)	-1·45 [-3·75, 0·85], p_{MD}=0·217<br>I^2=·, p_Q=·	NA	NA
Fruit (4/11)	-0·48 [-2·93, 1·97], p_{MD}=0·702<br>I^2=85·96%, p_Q<0·001	-0·64 [-3·04, 1·77], p_{MD}=0·605<br>I^2=83·84%, p_Q<0·001	-0·30 [-2·81, 2·21], p_{MD}=0·815<br>I^2=89·09%, p_Q<0·001
Dried Fruit (2/8)	-1·07 [-3.15, 1·01], p_{MD}=0·314<br>I^2=55·65%, p_Q=0·027	-1·08 [-3.20, 1·04], p_{MD}=0·316<br>I^2=55·57%, p_Q=0·027	-1·05 [-3·06, 0·96], p_{MD}=0·307<br>I^2=55·86%, p_Q=0·026
Mixed fruit forms (0/1)	0·00 [-2·65, 2·65], p_{MD}=1·00<br>I^2=·, p_Q=·	NA	NA
Sweetened fruit and nut bars (0/1)	3·44 [-0·72, 7·60], p_{MD}=0·105<br>I^2=·%, p_Q=·	NA	NA
Sweets and desserts (6/8)	0·46 [-1·28, 2·19], p_{MD}=0·607<br>I^2=0·00%, p_Q=0·991	0·40 [-1·49, 2·28], p_{MD}=0·682<br>I^2=0·00%, p_Q=0·997	0·56 [-0·88, 1·99], p_{MD}=0·447<br>I^2=0·00%, p_Q=0·939
Added nutritive (caloric) sweetener (7/7)	1·05 [-2·14, 4·24], p_{MD}=0·520<br>I^2=73·82%, p_Q=0·001	0·79 [-2·49, 4·06], p_{MD}=0·638<br>I^2=67·81%, p_Q=0·005	1·32 [-1·62, 4·25], p_{MD}=0·379<br>I^2=80·22%, p_Q<0·001
Mixed sources (with SSBs) (4/13)	0·19 [-1·18, 1·56], p_{MD}=0·788<br>I^2=41·22%, p_Q=0·060	0·27 [-1·08, 1·63], p_{MD}=0·692<br>I^2=36·34%, p_Q=0·092	0·07 [-1·33, 1·46], p_{MD}=0·925<br>I^2=50·18%, p_Q=0·020
Mixed sources (without SSBs) (3/3)	1·55 [-0·16, 3·25], p_{MD}=0·076<br>I^2=0·00%, p_Q=0·642	1·61 [-0·18, 3·39], p_{MD}=0·077<br>I^2=0·00%, p_Q=0·658	1·41 [-0·11, 2·94], p_{MD}=0·069<br>I^2=0·00%, p_Q=0·606
**Addition (24/64)**	**-1·15 [-1·98, -0·32], p_{MD}=0.007<br>I^2=58·35%, p_Q<0·001**	**-1·17 [-2·03, -0·30], p_{MD}=0·009<br>I^2=56·93%, p_Q<0·001**	**-1·13 [-1·90, 0·36], p_{MD}=0·004<br>I^2=61·90%, p_Q<0·001**
SSB (7/17)	0·23 [-0·71, 1·17], p_{MD}=0·631<br>I^2=0·00%, p_Q=0·868	0·23 [-0·76, 1·21], p_{MD}=0·652<br>I^2=0·00%, p_Q=0·882	0·24 [-0·60, 1·08], p_{MD}=0·573<br>I^2=0·00%, p_Q=0·824
100% Fruit juice (4/16)	-2·07 [-3·47, -0·66], p_{MD}=0.004<br>I^2=47·16%, p_Q=0·019	-2·10 [-3·55, -0·65], p_{MD}=0·004<br>I^2=45·54%, p_Q=0·025	-2·01 [-3·35, -0·67], p_{MD}=0·003<br>I^2=51·19%, p_Q=0·010
Fruit (7/16)	-3·89 [-5·73, -2·05], p_{MD}<0·001<br>I^2=55·06, p_Q=0·004	-4·14 [-5·97, -2·31], p_{MD}<0·001<br>**I^2=47.45·, p_Q=0·018**	-3·60 [-5·43, -1·77], p_{MD}<0·001<br>I^2=65·75, p_Q<0.001
Dried fruit (4/6)	-0·60 [-1·80, 0·59], p_{MD}=0.321<br>I^2=0·00%, p_Q=0·874	-0·52 [-1·81, 0·76], p_{MD}=0.426<br>I^2=0·00%, p_Q=0·925	-0·75 [-1·76, 0·26], p_{MD}=0.146<br>I^2=0·00%, p_Q=0·713
Sweetened cereal grains and bars (1*/2)	-0·47 [-2·37, 1·42], p_{MD}=0·625<br>I^2=45·62, p_Q=0·175	NA	NA
Sweets and desserts (1/3)	0·90 [-1·39, 3·19], p_{MD}=0·442<br>I^2=88·29%, p_Q<0.001	1·38 [-1·09, 3·85], p_{MD}=0·274<br>I^2=87·55%, p_Q<0.001	0·05 [-1·87, 1·98], p_{MD}=0·957<br>I^2=89·41%, p_Q<0.001
Added nutritive (caloric) sweetener (0/3)	2·49 [-2·88, 7·86], p_{MD}=0·364<br>I^2=0·00, p_Q=0·909	NA	NA
Mixed sources (with SSBs) (0/1)	5·30 [1·11, 9·50], p_{MD}=0·013<br>I^2=·, p_Q=·	NA	NA

**Subtraction (1*/10)**	**-0·09 [-0·74, 0·57], p_{MD}=0·796 I^2=61·69%, p_Q=0·005**	**NA**	**NA**
SSB (1*/8)	-0·41 [-1·68, 0·86], p_{MD}=0·530 I^2=28·79%, p_Q=0·198	NA	NA
Mixed sources (with SSBs) (0/2)	0·05 [-0·38, 0·47], p_{MD}=0·828 I^2=92·66%, p_Q=0·198	NA	NA
***Ad libitum* (5/6)**	**1·14 [-0·66, 2·93], p_{MD}=0·214 I^2=0·00%, p_Q=0·619**	**1·21 [-0·95, 3·37], p_{MD}=0·273 I^2=0·00%, p_Q=0·778**	**0·92 [-0·47, 2·30], p_{MD}=0·194 I^2=0·00%, p_Q=0·726**
Mixed sources (with SSBs) (5/6)	1·14 [-0·66, 2·93], p_{MD}=0·214 I^2=0·00%, p_Q=0·619	1·21 [-0·95, 3·37], p_{MD}=0·273 I^2=0·00%, p_Q=0·778	1·90 [-0·02, 3·82], p_{MD}=0·052 I^2=22·08%, p_Q=0·268

CI=confidence interval; MD=mean difference; no.=number
*For this crossover trial, a correlation coefficient was not used since a p-value was provided for the mean difference between treatment changes.

S7 Table. GRADE certainty of evidence assessment for the effect of fructose-containing sugars on blood pressure by trial energy control.

Outcome and trial (N)	Design	Quality assessment							Effect (MD [95%CI], P_{MD})	Quality[a]	Interpretation of magnitude of effect[b]
		Downgrades					Upgrades				
		Risk of bias	Inconsistency	Indirectness	Imprecision	Publication bias	Dose response				
**Systolic blood pressure (mmHg)**											
Substitution (72)	Randomized and non-randomized trials	Not serious	Not serious	Very serious[1]	Not serious	None	None	↔	-0.33 [-1.16 to 0.51], P=0.445	⊕⊕◯◯ Low	No effect
Addition (64)	Randomized and non-randomized trials	Not serious	Serious[2]	Very serious[3]	Serious[4]	None	Linear DR, no upgrade[5]	↓	-2.23 [-3.40 to -1.06], P<0.001	⊕◯◯◯ Very low	Small important
Subtraction (10)	Randomized trials	Not serious	Not serious[6]	Very serious[7]	Serious[8]	None	None	↔	-1.16 [-2.90 to 0.57], P=0.188	⊕◯◯◯ Very low	No effect
Ad libitum (6)	Randomized and non-randomized trials	Not serious[9]	Not serious	Very serious[10]	Serious[11]	None[12]	None	↔	0.98 [-0.43 to 2.39], P=0.173	⊕◯◯◯ Very low	No effect
**Diastolic blood pressure (mmHg)**											
Substitution (72)	Randomized and non-randomized trials	Not serious	Not serious[13]	Not serious	Not serious	None	None	↔	0.12 [-0.53 to 0.78], P=0.714	⊕⊕⊕⊕ High	No effect
Addition (64)	Randomized and non-randomized trials	Not serious	Serious[14]	Very serious[15]	Not serious	None	Linear DR, no upgrade[16]	↓	-1.15 [-1.98 to -0.32], P=0.007	⊕◯◯◯ Very low	Trivial
Subtraction (10)	Randomized trials	Not serious	Not serious[17]	Very serious[18]	Not serious	None	None	↔	-0.09 [-0.74 to 0.57], P=0.796	⊕⊕◯◯ Low	No effect
Ad libitum (6)	Randomized and non-randomized trials	Serious[19]	Not serious	Very serious[20]	Serious[21]	None[12]	None	↔	1.14 [-0.66 to 2.93], p=0.214	⊕◯◯◯ Very low	No effect

[a] Since all included trials were randomized or non-randomized controlled trials, the certainty of the evidence was graded as high for all outcomes by default and then downgraded or upgraded based on pre-specified criteria. Criteria for downgrades included risk of bias (downgraded if the majority of trials were considered to be at high risk of bias); inconsistency (downgraded if there was substantial unexplained heterogeneity [$I^2 \geq 50\%$, $P_Q < 0.10$]; indirectness (downgraded if there were factors absent or present relating to the participants, interventions, or outcomes that limited the generalizability of the results); imprecision (downgraded if the 95% confidence interval crossed the minimally important difference [MID] for harm or benefit set at 2mmHg for SBP and DBP;

Lewington S, Clarke R, Qizilbash N, Peto R, Collins R; Prospective Studies Collaboration. Lancet. 2002 Dec 14;360(9349):1903-13.); and publication bias (downgraded if there is evidence of publication bias based on funnel plot asymmetry and/or significant Egger's or Begg's tests (P<0.10) with confirmation by adjustment by Duval and Tweedie trim-and-fill analysis). Criteria for upgrades included a significant dose-response gradient.

[b] Interpretation of the magnitude of effect was done using the effect size categories according to new GRADE guidance (Santesso et al. J Clin Epidemiol. 2020;119:126-35, Schünemann et al. The GRADE Working Group, 2013. Available from https:// https://gdt.gradepro.org/app/handbook/handbook.htm. Accessed 06 Jun 2021): large, moderate, small important, and trivial/unimportant effect. We defined these effect size categories by adapting GRADE criteria (Guyatt et al. Journal of Clinical Epidemiology 64 (2011) 1311e1316) for upgrading the magnitude of effect using our pre-specified MID (2mmHg): large effect (≥5x MID); moderate effect (≥2x MID); small important effect (≥1x MID); and trivial/unimportant effect (< 1 MID).

CI=confidence interval; DR=dose response; MD=mean difference

[1] Double downgrade for very serious indirectness as there was a significant interaction by food source (P=0.089) indicating that there is biological plausibility of differences in behaviour of foods due to the food matrices.

[2] Downgrade for serious inconsistency, due to substantial unexplained heterogeneity I^2=69%, P_Q <0.001.

[3] Double downgrade for very serious indirectness as there was a significant interaction by food source (P<0.001) indicating that there is biological plausibility of differences in behaviour of foods due to the food matrices.

[4] Downgrade for serious imprecision as the 95% confidence interval (-3.40 to -1.06) overlaps the MID of clinically important benefit for SBP (2mmHg).

[5] Although a significant dose response was detected ($coef_{linear}$=0.34 [95% CI, 0.21 to 0.47], P_{linear}<0.001), we did not upgrade for dose response as we detected a significant test for interaction by food source (p<0.001), with fruit driving the signal for dose response.

[6] Although there was substantial heterogeneity in the analysis, we did not downgrade for serious inconsistency, since it was explained when the study by Vazquez-Duran et al. 2016 (water) was removed as part of a priori sensitivity analyses (Original: I^2=82%, P_Q<0.001; after study removed: I^2=43%, P_Q=0.078).

[7] Double downgrade for very serious indirectness as there were only 2 food sources available for analyses (SSB and Mixed sources (with SSBs)), thus limiting the ability to assess differences in food sources.

[8] Downgrade for serious imprecision as the 95% confidence interval (-2.90 to 0.57) overlaps the MID of clinically important benefit for SBP (2mmHg).

[9] No downgrade for serious risk of bias. Although 4 of the 6 trials had high ROB for sequence generation and allocation concealment (due to not being randomized), sensitivity analyses revealed no difference in effect between those 4 trials with high ROB (MD:

0.35mmHg; 95% CI: -5.60, 6.29; P_{MD}=0.909), those 2 with low ROB (MD: 1.02mmHg; 95% CI: -0.43, 2.47; P_{MD}=0.170), in these ROB domains and the overall pooled effect (MD: 0.98; 95% CI: -0.43 to 2.39; P_{MD}=0.174).
[10] Double downgrade for very serious indirectness as there was only one food source available for analyses (SSB and other food sources), thus limiting the ability to assess differences in food sources.
[11] Downgrade for serious imprecision as the 95% confidence interval (-0.43 to 2.39) overlaps the MID of clinically important harm for SBP (2mmHg).
[12] No downgrade for publication bias, as publication bias could not be assessed due to lack of power for assessing funnel plot asymmetry and small study effects (<10 trial comparisons included in the meta-analysis).
[13] Although there was substantial heterogeneity in the analysis, we did not downgrade for serious inconsistency, since it was explained when the study by Singh et al. 1997 was removed as part of a priori sensitivity analyses (Original: I^2=59%, P_Q<0.001; after study removed: I^2=31%, P_Q=0.008).
[14] Downgrade for serious inconsistency, due to substantial unexplained heterogeneity I^2=58%, P_Q <0.001.
[15] Double downgrade for very serious indirectness as there was a significant interaction by food source (p<0.001) indicating that there is biological plausibility of differences in behaviour of foods due to the food matrices.
[16] Although a significant dose response was detected ($coef_{linear}$=0.24 [95% CI, 0.13 to 0.34], P_{linear}<0.001), we did not upgrade for dose response as we detected a significant test for interaction by food source (p<0.001), with fruit driving the signal for dose response.
[17] Although there was substantial heterogeneity in the analysis, we did not downgrade for serious inconsistency, since it was explained when the study by Vazquez-Duran et al. 2016 (water) was removed as part of a priori sensitivity analyses (Original: I^2=62%, P_Q=0.005; after study removed: I^2=49%, P_Q=0.049).
[18] Double downgrade for very serious indirectness as there were only 2 food sources available for analyses (SSB and SSB and other food sources), thus limiting the ability to assess differences in food sources.
[19] Downgrade for serious risk of bias. Four of the 6 trials had high ROB for sequence generation and allocation concealment (due to not being randomized) and sensitivity analyses revealed a difference in effect where those 4 trials with high ROB increased SBP (MD: 3.75mmHg; 95% CI: 0.22, 7.28; P_{MD}=0.038), whereas the overall pooled effect showed no effect (MD: 1.14; 95% CI: -0.66 to 2.92; P_{MD}=0.214) and those 2 with low ROB had no effect (MD: 0.23mmHg; 95% CI: -1.85, 2.31; P_{MD}=0.820).
[20] Double downgrade for very serious indirectness as there was only one food source available for analyses (SSB and other food sources), thus limiting the ability to assess differences in food sources.
[21] Downgrade for serious imprecision as the 95% confidence interval (-0.66 to 2.93) overlaps the MID of clinically important harm for SBP (2mmHg).

S8 Table. GRADE certainty of evidence assessment for the effect of fructose-containing sugars on blood pressure acid by important food source of fructose-containing sugars.

		Quality assessment								
		Downgrades					Upgrades			
Outcome and trial (N)	Design	Risk of bias	Inconsistency	Indirectness	Imprecision	Publication bias	Dose response	Effect (MD [95%CI], P_{MD})	Quality[a]	Interpretation of magnitude of effect[b]
**SBP in substitution trials (mmHg)**										
SSB (16)	Randomized and non-randomized trials	Not serious	Not serious	Not serious	Not serious	None	None	↔ 0.39 [-0.85 to 1.64], P=0.538	⊕⊕⊕⊕ High	No effect
Sweetened dairy (3)	Randomized trials	Not serious	Not serious	Serious[3]	Serious[1]	None[4]	None[5]	↔ 2.18 [-0.49 to 4.85], P=0.110	⊕⊕◯◯ Low	No effect
Sweetened dairy alternative (soy) (1)	Randomized trials	Not serious	Not serious[6]	Serious[7]	Serious[1]	None[4]	None[5]	↔ -2.33 [-5.89 to 1.23], P=0.199	⊕⊕◯◯ Low	No effect
Fruit (11)	Randomized and non-randomized trials	Not serious	Not serious[8]	Not serious	Serious[1]	None	None	↔ -2.13 [-4.79 to 0.53], P=0.116	⊕⊕⊕◯ Moderate	No effect
Dried fruit (8)	Randomized and non-randomized trials	Not serious	Not serious	Not serious	Serious[1]	None[4]	None	↔ -2.22 [-4.99 to 0.55], P=0.116	⊕⊕⊕◯ Moderate	No effect
Mixed fruit forms (1)	Randomized trials	Not serious	Not serious[6]	Serious[9]	Serious[1]	None[4]	None[5]	↔ -1.60 [-6.85 to 3.65], P=0.550	⊕⊕◯◯ Low	No effect
Sweetened cereal grains and bars (1)	Randomized trials	Not serious	Not serious[6]	Serious[10]	Serious[1]	None[4]	None[5]	↔ 4.92 [-0.59 to 10.43], P=0.080	⊕⊕◯◯ Low	No effect
Sweets and desserts (8)	Randomized and non-randomized trials	Not serious	Not serious	Not serious	Serious[1]	None[4]	None	↔ -1.29 [-3.47 to 0.90], P=0.248	⊕⊕⊕◯ Moderate	No effect
Added nutritive (caloric) sweetener (7)	Randomized and non-randomized trials	Not serious	Not serious	Not serious	Serious[1]	None[4]	None	↔ -1.67 [-4.00 to 0.65], P=0.159	⊕⊕⊕◯ Moderate	No effect
Mixed sources (with SSBs) (13)	Randomized and non-randomized trials	Not serious	Not serious	Not serious	Serious[1]	None	Linear DR, no upgrade[2]	↔ 1.48 [-0.91 to 3.87], P=0.224	⊕⊕⊕◯ Moderate	No effect

Mixed sources (without SSBs) (3)	Randomized trials	Not serious	Not serious	Serious[11]	Serious[1]	None[4]	None[5]	↔	2.56 [-0.24 to 5.36], P=0.073	⊕⊕◯◯ Low	No effect
**SBP in addition trials (mmHg)**											
SSB (17)	Randomized and non-randomized trials	Not serious[12]	Not serious	Not serious	Not serious	None	None	↔	0.74 [-0.42 to 1.90], P=0.209	⊕⊕⊕⊕ High	No effect
100% Fruit juice (16)	Randomized and non-randomized trials	Not serious	Not serious[14]	Not serious	Not serious	None[4]	None	↓	-3.74 [-5.28 to -2.20], P<0.001	⊕⊕⊕⊕ High	Small important
Fruit (16)	Randomized and non-randomized trials	Not serious	Serious[15]	Not serious	Not serious	None	Linear DR, no upgrade[16]	↓	-6.37 [-9.21 to -3.52], P<0.001	⊕⊕⊕◯ Moderate	Moderate
Dried fruit (6)	Randomized and non-randomized trials	Not serious[17]	Not serious	Not serious	Serious[1]	None[4]	None	↔	-1.96 [-4.69 to 0.77], P=0.160	⊕⊕⊕◯ Moderate	No effect
Sweetened cereal grains and bars (2)	Randomized and non-randomized trials	Not serious[18]	Not serious	Not serious	Serious[1]	None[4]	None[5]	↔	-0.68 [-3.78 to 2.42], P=0.669	⊕⊕⊕◯ Moderate	No effect
Sweets and desserts (3)	Randomized and non-randomized trials	Not serious	Serious[19]	Not serious	Serious[1]	None[4]	None[5]	↔	-0.45 [-6.88 to 5.97], P=0.211	⊕⊕◯◯ Low	No effect
Added nutritive (caloric) sweetener (3)	Randomized trials	Not serious	Not serious	Serious[20]	Serious[1]	None[4]	None[5]	↔	4.58 [-2.05 to 11.20], P=0.176	⊕⊕◯◯ Low	No effect
Mixed sources (with SSBs) (1)	Randomized trials	Not serious	Not serious[6]	Serious[13]	Not serious	None	None[5]	↑	6.90 [2.22 to 11.58], P=0.004	⊕⊕⊕◯ Moderate	Moderate
**SBP subtraction trials (mmHg)**											
SSB (8)	Randomized trials	Not serious	Not serious	Not serious	Serious[1]	None[4]	Linear DR[21]	↓	-0.47 [-2.70 to 1.75], P=0.676<br>**Beta -2.66mmHg [-4.28 to -1.03] per 1 serving of SSB (355ml, 8%E), P=0.001**	⊕⊕⊕⊕ High	Small important
Mixed sources (with SSBs) (2)	Randomized trials	Not serious	Serious[22]	Serious[23]	Serious[1]	None[4]	None[5]	↓	-2.26 [-2.79 to -1.73], P<0.001	⊕◯◯◯ Very low	Small important
**SBP in *ad libitum* trials (mmHg)**											

Mixed sources (with SSBs) (6)	Randomized and non-randomized trials	Not serious[24]	Not serious	Not serious	Serious[1]	None[4]	None	↔	0.98 [-0.43 to 2.39], P=0.173	⊕⊕⊕◯ Moderate	No effect
**DBP in addition trials (mmHg)**											
SSB (17)	Randomized and non-randomized trials	Not serious[25]	Not serious	Not serious	Not serious	None[26]	None	↔	0.23 [-0.71 to 1.17], P=0.631	⊕⊕⊕⊕ High	No effect
100% Fruit juice (16)	Randomized and non-randomized trials	Not serious	Not serious	Not serious	Serious[1]	None	None	↓	-2.06 [-3.47 to -0.66], P=0.004	⊕⊕⊕◯ Moderate	Small important
Fruit (16)	Randomized and non-randomized trials	Not serious	Serious[27]	Not serious	Not serious	None	Linear DR, no upgrade[28]	↓	-3.88 [-5.72 to -2.04], P<0.001	⊕⊕⊕◯ Moderate	Small important
Dried fruit (6)	Randomized and non-randomized trials	Not serious[29]	Not serious	Not serious	Not serious	None[4]	None	↔	-0.60 [-1.80 to 0.59], P=0.321	⊕⊕⊕⊕ High	No effect
Sweetened cereal grains and bars (2)	Randomized and non-randomized trials	Not serious[18]	Not serious	Not serious	Serious[1]	None[4]	None[5]	↔	-0.80 [-3.64 to 2.03], P=0.180	⊕⊕⊕◯ Moderate	No effect
Sweets and desserts (3)	Randomized and non-randomized trials	Not serious	Not serious[30]	Not serious	Serious[1]	None[4]	None[5]	↔	-0.38 [-7.42 to 6.67], P=0.442	⊕⊕⊕◯ Moderate	No effect
Added nutritive (caloric) sweetener (3)	Randomized trials	Not serious	Not serious	Serious[20]	Serious[1]	None[4]	None[5]	↔	2.49 [-2.88 to 7.86], P=0.364	⊕⊕◯◯ Low	No effect
Mixed sources (with SSBs) (1)	Randomized trials	Not serious	Not serious[6]	Serious[13]	Serious[1]	None[4]	None[5]	↑	5.30 [1.11 to 9.49], P=0.013	⊕⊕◯◯ Low	Moderate
**DBP subtraction trials (mmHg)**											
SSB (8)	Randomized trials	Not serious	Not serious	Not serious	Not serious	None[4]	Non-linear DR, no upgrade[31]	↔	-0.41 [-1.68 to 0.86], P=0.530	⊕⊕⊕⊕ High	No effect
Mixed sources (with SSBs) (2)	Randomized trials	Not serious	Serious[32]	Serious[23]	Not serious	None[4]	None[5]	↔	0.05 [-1.51 to 1.60], P=0.828	⊕⊕◯◯ Low	No effect

**DBP in *ad libitum* trials (mmHg)**											
Mixed sources (with SSBs) (6)	Randomized and non-randomized trials	Serious[33]	Not serious	Not serious	Serious[1]	None[4]	None	↔	1.14 [-0.66 to 2.93], P=0.214	⊕⊕◯◯ Low	No effect

[a] Since all included trials were randomized or non-randomized controlled trials, the certainty of the evidence was graded as high for all outcomes by default and then downgraded or upgraded based on pre-specified criteria. Criteria for downgrades included risk of bias (downgraded if the majority of trials were considered to be at high risk of bias); inconsistency (downgraded if there was substantial unexplained heterogeneity [$I^2 \geq 50\%$, $P_Q < 0.10$]; indirectness (downgraded if there were factors absent or present relating to the participants, interventions, or outcomes that limited the generalizability of the results); imprecision (downgraded if the 95% confidence interval crossed the minimally important difference [MID] for harm set at 2mmHg for SBP and DBP; Lewington S, Clarke R, Qizilbash N, Peto R, Collins R; Prospective Studies Collaboration. Lancet. 2002 Dec 14;360(9349):1903-13.); and publication bias (downgraded if there is evidence of publication bias based on funnel plot asymmetry and/or significant Egger's or Begg's tests (P<0.10) with confirmation by adjustment by Duval and Tweedie trim-and-fill analysis). Criteria for upgrades included a significant dose-response gradient.

[b] Interpretation of the magnitude of effect was done using the effect size categories according to new GRADE guidance (Santesso et al. J Clin Epidemiol. 2020;119:126-35, Schünemann et al. The GRADE Working Group, 2013. Available from https:// https://gdt.gradepro.org/app/handbook/handbook.htm. Accessed 06 Jun 2021): large, moderate, small important, and trivial/unimportant effect. We defined these effect size categories by adapting GRADE criteria (Guyatt et al. Journal of Clinical Epidemiology 64 (2011) 1311e1316) for upgrading the magnitude of effect using our pre-specified MID (2mmHg): large effect (≥5x MID); moderate effect (≥2x MID); small important effect (≥1x MID); and trivial/unimportant effect (< 1 MID).

CI=confidence interval; DR=dose response; MD=mean difference; MID=minimally important difference; SSB=sugar-sweetened beverage.

[1] Downgrade for serious imprecision as the 95% confidence interval overlaps the MID of clinically important harm or benefit for SBP (2mmHg).

[2] Although there was a positive linear dose response (P=0.009) for the effect of Mixed sources (with SSBs) on SBP in substitution trials, we did not upgrade since the dose response was no longer significant with the removal of one outlier (P=0.204).

[3] Downgrade for serious indirectness as all trial comparisons come from one study of all healthy mixed weight, younger (mean 38y) adult participants which leads to poor applicability of the results to the general population.
[4] No downgrade for publication bias, as publication bias could not be assessed due to lack of power for assessing funnel plot asymmetry and small study effects (<10 trial comparisons included in the meta-analysis).
[5] No upgrade for dose-response, as dose-response could not be assessed as <6 trials were available.
[6] No downgrade for serious inconsistency, as heterogeneity could not be assessed due to the inclusion of only one trial comparison.
[7] Downgrade for serious indirectness as the one trial comparison includes 31 overweight adult participants which leads to poor applicability of the results to the general population.
[8] Although there was substantial heterogeneity in the analysis of the effect of fruit on SBP in substitution trials, we did not downgrade for serious inconsistency, since it was explained when the study by Singh et al. 1997 was removed as part of a priori sensitivity analyses (Original: I^2=74%, P_Q<0.001; after study removed: I^2=37%, P_Q=0.109).
[9] Downgrade for serious indirectness as the one trial comparison includes 50 healthy mixed weight adult participants which leads to poor applicability of the results to the general population.
[10] Downgrade for serious indirectness as the one trial comparison includes 50 healthy mixed weight, young (mean 26y) adult participants which leads to poor applicability of the results to the general population.
[11] Downgrade for serious indirectness as all trial comparisons includes overweight adult participants, one of which was overweight and prediabetes, which leads to poor applicability of the results to the general population.
[12] No downgrade for serious risk of bias. Although 8 of the 17 trials had high ROB for sequence generation and allocation concealment (due to not being randomized), subgroup analyses revealed no effect modification for either sequence generation (P=0.144) or allocation concealment (P=0.576).
[13] Downgrade for serious indirectness as the one trial comparison includes 31 overweight adult participants which leads to poor applicability of the results to the general population.
[14] Although there was substantial heterogeneity in the analysis, we did not downgrade for serious inconsistency, since it was explained when the study by Abedini et al. 2020 or Lazavi et al. 2018 were removed as part of a priori sensitivity analyses (Original: I^2=53.5%, P_Q=0.006; after removal of Abedini et al. 2020: I^2=47%, P_Q=0.023; or after removal of Lazavi et al. 2018: I^2=41%, P_Q=0.048).
[15] Downgrade for serious inconsistency, due to substantial unexplained heterogeneity I^2=69%, P_Q <0.001.
[16] Although there was a significant positive linear dose response gradient for the effect of fruit on SBP in addition trials ($Coef_{linear}$: 0.96; 95% CI: 0.24 to 1.69, P=0.009) where greater reductions were seen with smaller doses, we did not upgrade since the estimated dose response was below 0, representing a reduction in SBP across the dose response range.
[17] No downgrade for serious risk of bias. Although 4 of the 6 trials had high ROB for sequence generation and allocation concealment (due to not being randomized), sensitivity analyses revealed no difference in effect between those 4 trials with high ROB (MD: -

1.84mmHg; 95% CI: -5.02, 1.35; P_{MD}=0.259), those 2 with unclear ROB (MD: -2.30mmHg; 95% CI: -7.62, 3.02; P_{MD}=0.397), in these ROB domains and the overall pooled effect (MD: -1.96; 95% CI: -4.69 to 0.77; P_{MD}=0.160).

[18] No downgrade for serious risk of bias. Although 1 of the 2 trials had high ROB for sequence generation and allocation concealment (due to not being randomized), sensitivity analyses revealed no difference in effect between the 2 trials nor with the overall pooled estimate as all showed no effect.

[19] Downgrade for serious inconsistency, due to substantial unexplained heterogeneity I^2=81%, P_Q <0.001.

[20] Downgrade for serious indirectness as the 3 trial comparisons are all from one study of 141 HIV positive, young (mean 40y) adult participants which leads to poor applicability of the results to the general population.

[21] Upgrade for significant inverse linear dose response gradient ($coeff_{linear}$: -0.33; 95% CI: -0.54 to -0.13, P=0.001).

[22] Downgrade for serious inconsistency, due to substantial unexplained heterogeneity I^2=97%, P_Q <0.001.

[23] Downgrade for serious indirectness as the 2 trial comparisons are from one study of 146 health mixed weight, young (mean 22y) adult participants which leads to poor applicability of the results to the general population.

[24] No downgrade for serious risk of bias. Although 4 of the 6 trials had high ROB for sequence generation and allocation concealment (due to not being randomized), sensitivity analyses revealed no difference in effect between those 4 trials with high ROB (MD: 0.35mmHg; 95% CI: -5.60, 6.29; P_{MD}=0.909), those 2 with low ROB (MD: 1.02mmHg; 95% CI: -0.43, 2.47; P_{MD}=0.170), in these ROB domains and the overall pooled effect (MD: 0.98; 95% CI: -0.43 to 2.39; P_{MD}=0.174).

[25] No downgrade for serious risk of bias. Although 8 of the 17 trials had high ROB for sequence generation and allocation concealment (due to not being randomized), subgroup analyses revealed no effect modification for either sequence generation (P=0.742) or allocation concealment (P=0.446).

[26] Although a significant publication bias was detected at P=0.036 in Egger's test, we did not downgrade for publication bias as the imputation of 7 trials from trim-and-fill analyses did not change the significance on the overall effect (Original MD: 0.23; 95% CI: -0.71 to 1.17; imputed MD: -0.33; 95% CI: -1.19 to 0.53).

[27] Downgrade for serious inconsistency, due to substantial unexplained heterogeneity I^2=55%, P_Q =0.004.

[28] Although there was a significant positive linear dose response gradient for the effect of fruit on DBP in addition trials ($Coef_{linear}$: 0.68; 95% CI: 0.15 to 1.21, P=0.012) where greater reductions were seen with smaller doses, we did not upgrade since the estimated dose response was below 0, representing a reduction in SBP across the dose response range.

[29] No downgrade for serious risk of bias. Although 4 of the 6 trials had high ROB for sequence generation and allocation concealment (due to not being randomized), sensitivity analyses revealed no difference in effect between those 4 trials with high ROB (MD: --1.14mmHg; 95% CI: -3.01, 0.73; P_{MD}=0.230), those 2 with unclear ROB (MD: -0.23mmHg; 95% CI: -1.79, 1.32; P_{MD}=0.770), in these ROB domains and the overall pooled effect (MD: -0.60; 95% CI: -1.80 to 0.59; P_{MD}=0.321).

[30] Although there was substantial heterogeneity in the analysis of the effect of sweets and desserts on DBP in addition trials, we did not downgrade for serious inconsistency, since it was explained when the study by Leskinen et al. 2014 was removed as part of a priori sensitivity analyses (Original: I^2=88%, P_Q<0.001; after study removed: I^2=31%, P_Q=0.229).
[31] Although we observed a non-linear dose response showing an inverse dose response gradient up to a dose of about 15%E after which it appears to become positive, we did not upgrade for this dose response because the magnitude of the entire dose response effect remained trivial (<1 MID, 2mmHg) over the dose response range.
[32] Downgrade for serious inconsistency, due to substantial unexplained heterogeneity I^2=93%, P_Q <0.001.
[33] Downgrade for serious risk of bias. Four of the 6 trials had high ROB for sequence generation and allocation concealment (due to not being randomized) and sensitivity analyses revealed a difference in effect where those 4 trials with high ROB increased SBP (MD: 3.75mmHg; 95% CI: 0.22, 7.28; P_{MD}=0.038), whereas the overall pooled effect showed no effect (MD: 1.14; 95% CI: -0.66 to 2.92; P_{MD}=0.214) and those 2 with low ROB had no effect (MD: 0.23mmHg; 95% CI: -1.85, 2.31; P_{MD}=0.820).

Figures

S1 Fig. Risk of bias proportion graph for the effect of important food sources of fructose-containing sugars on SBP and DBP (mmHg) in substitution trials*.

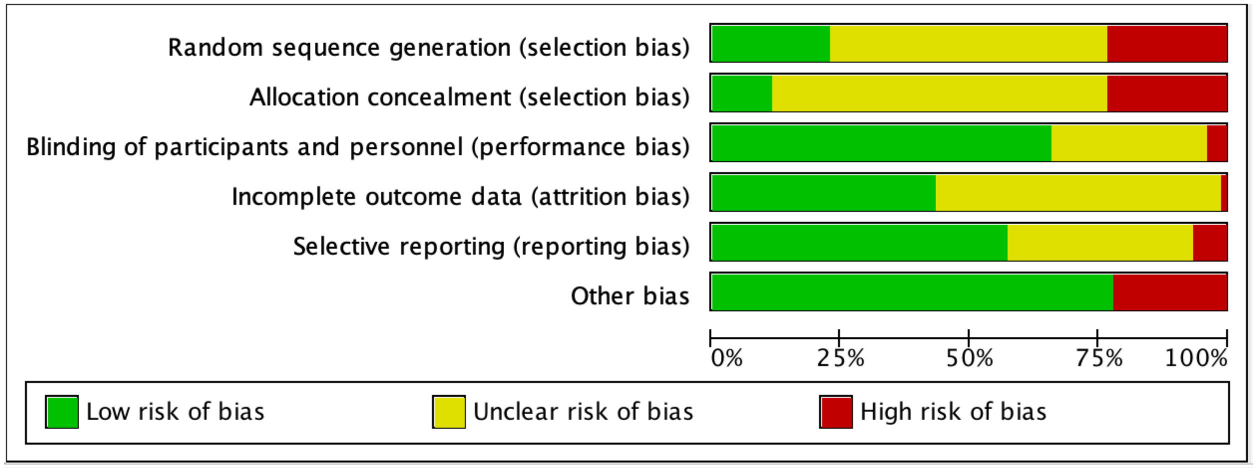

Colored bars represent the proportion of trials assessed as low (green), unclear (yellow) or high (red) risk of bias for the six domains of bias above according to criteria set by the Cochrane Risk of Bias tool in the 72 included controlled trial comparisons.
High other risk of bias (carry-over effect) was given to crossover trials which had no washout between interventions. Trials which did not have this characteristic were rated as Low.
*all trial comparisons and their risk of bias assessments in substitution trials for SBP and DBP were identical, as presented here.

DBP=diastolic blood pressure, SBP=systolic blood pressure

S2 Fig. Risk of bias proportion graph for the effect of important food sources of fructose-containing sugars on SBP and DBP in addition trials*.

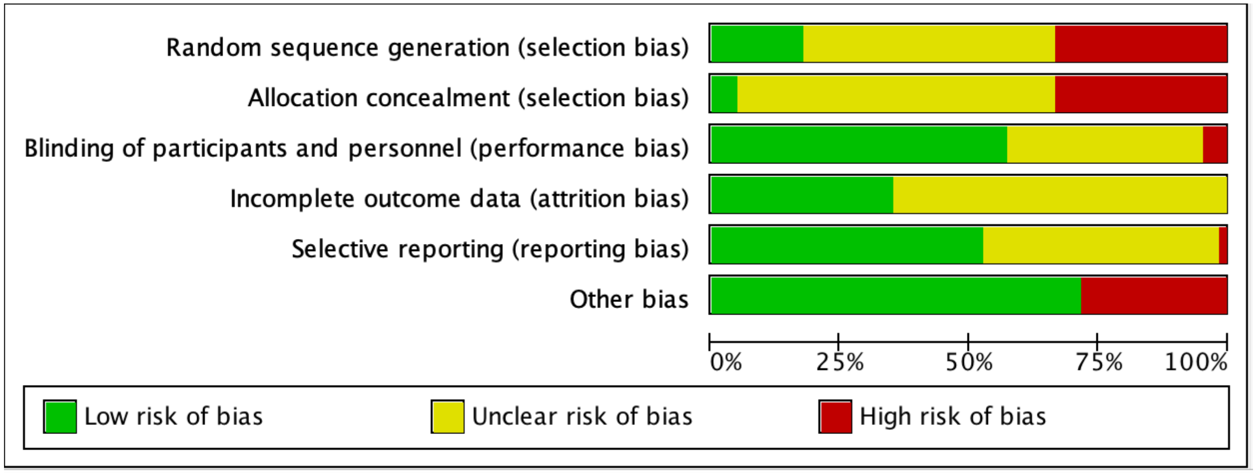

Colored bars represent the proportion of trials assessed as low (green), unclear (yellow) or high (red) risk of bias for the six domains of bias above according to criteria set by the Cochrane Risk of Bias tool in the 64 included controlled trial comparisons.
High other risk of bias (carry-over effect) was given to crossover trials which had no washout between interventions. Trials which did not have this characteristic were rated as Low.
*all trial comparisons and their risk of bias assessments in substitution trials for SBP and DBP were identical, as presented here.

DBP=diastolic blood pressure, SBP=systolic blood pressure

S3 Fig. Risk of bias proportion graph for the effect of important food sources of fructose-containing sugars on SBP and DBP in subtraction trials*.

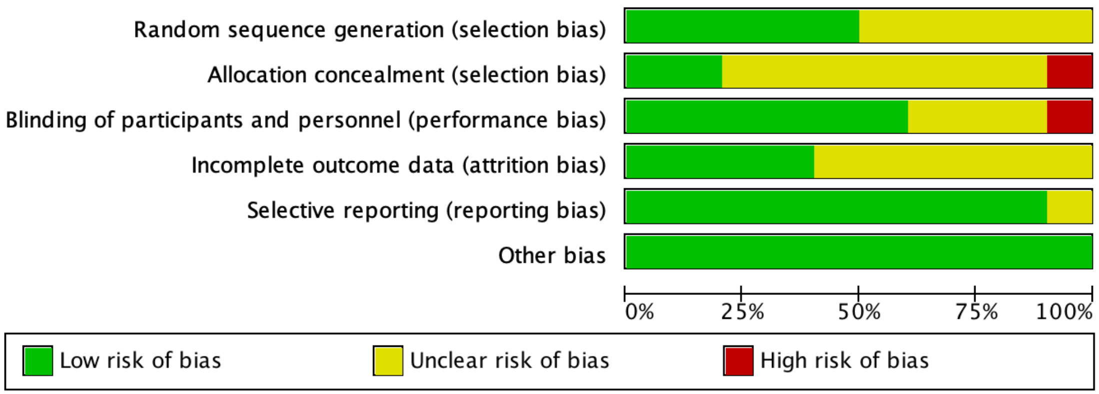

Colored bars represent the proportion of trials assessed as low (green), unclear (yellow) or high (red) risk of bias for the six domains of bias above according to criteria set by the Cochrane Risk of Bias tool in the 10 included controlled trial comparisons.
High other risk of bias (carry-over effect) was given to crossover trials which had no washout between interventions. Trials which did not have this characteristic were rated as Low.
*all trial comparisons and their risk of bias assessments in substitution trials for SBP and DBP were identical, as presented here.

DBP=diastolic blood pressure, SBP=systolic blood pressure

S4 Fig. Risk of bias proportion graph for the effect of important food sources of fructose-containing sugars on SBP and DBP in *ad libitum* trials*.

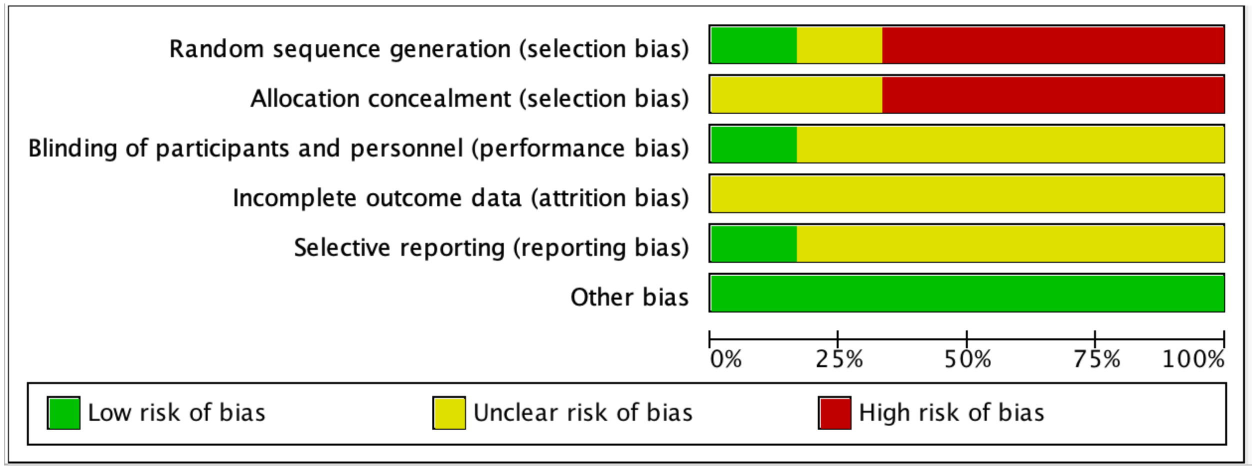

Colored bars represent the proportion of trials assessed as low (green), unclear (yellow) or high (red) risk of bias for the six domains of bias above according to criteria set by the Cochrane Risk of Bias tool in the 6 included controlled trial comparisons.
High other risk of bias (carry-over effect) was given to crossover trials which had no washout between interventions. Trials which did not have this characteristic were rated as Low.
*all trial comparisons and their risk of bias assessments in substitution trials for SBP and DBP were identical, as presented here.

DBP=diastolic blood pressure, SBP=systolic blood pressure

S5 Fig (1 of 3). Forest plot of controlled trials of the effect of important food sources of fructose-containing sugars on SBP (mmHg) in substitution trials.

Systolic Blood Pressure

Substitution Trials

Trial or subgroup	N Control	N Intervention	Mean difference with 95% CI	Weight (%)	Risk of bias A	B	C	D	E	F
**01. SSB**										
Aeberli et al. 2011 (HG vs. HF)	29	29	1.30 [-2.95 to 5.55]	2.03	U	U	L	L	L	L
Aeberli et al. 2011 (HG vs. HS)	29	29	3.30 [-0.92 to 7.52]	2.05	U	U	L	L	L	L
Aeberli et al. 2011 (MG vs. MF)	29	29	-1.00 [-4.11 to 2.11]	2.69	U	U	L	L	L	L
Aeberli et al. 2013 (HG vs HF)	9	9	-2.67 [-11.79 to 6.45]	0.70	U	L	L	L	H	L
Aeberli et al. 2013 (HG vs HS)	9	9	-2.00 [-11.60 to 7.60]	0.64	U	L	L	L	H	L
Chiu et al., 2020	30	30	2.00 [-0.00 to 4.00]	3.45	L	L	L	L	L	L
Engel et al. 2017	15	14	5.90 [-2.13 to 13.93]	0.87	U	U	H	U	L	L
Geidl-Flueck et al. 2021 (GLC vs FRC)	22	23	-1.00 [-7.91 to 5.91]	1.09	L	U	L	U	L	L
Geidl-Flueck et al. 2021 (GLC vs SUC)	22	23	-3.00 [-10.01 to 4.01]	1.07	L	U	L	U	L	L
Hieronimus et al. 2020 (Frc25 vs. Glc 25)	28	28	-4.57 [-9.38 to 0.24]	1.77	H	H	L	U	L	L
Hieronimus et al. 2020 (HFCS25 vs. Glc25)	28	28	-1.31 [-6.10 to 3.48]	1.78	H	H	L	U	L	L
Koivisto & Yki-Jävinen 1993	10	10	5.00 [-7.54 to 17.54]	0.40	U	U	L	U	U	L
Sigala et al. 2020	28	24	-0.22 [-5.61 to 5.17]	1.54	H	H	L	U	U	L
Silbernagel et al. 2011	10	10	-5.00 [-13.32 to 3.32]	0.82	L	L	L	U	U	L
Stanhope et al. 2009	15	17	1.20 [-4.34 to 6.74]	1.49	H	H	L	U	U	L
Swarbrick et al. 2008	7	7	3.00 [-24.44 to 30.44]	0.09	H	H	L	L	U	L
Heterogeneity: τ^2 = 0.27, I^2 = 4.04%, H^2 = 1.04			0.39 [-0.85 to 1.64]							
Test of $\theta_i = \theta_j$: Q(15) = 15.63, p = 0.41										
**02. Sweetened dairy**										
Angelopoulos et al. 2015 (FRC)	77	65	2.35 [-2.23 to 6.93]	1.88	U	U	L	U	L	L
Angelopoulos et al. 2015 (HFCS)	77	61	3.53 [-1.14 to 8.20]	1.83	U	U	L	U	L	L
Angelopoulos et al. 2015 (SUC)	77	64	0.68 [-3.93 to 5.29]	1.86	U	U	L	U	L	L
Heterogeneity: τ^2 = 0.00, I^2 = 0.00%, H^2 = 1.00			2.18 [-0.49 to 4.85]							
Test of $\theta_i = \theta_j$: Q(2) = 0.73, p = 0.69										
**03. Sweetened dairy alternative (soy)**										
Maleki et al. 2019	31	31	-2.33 [-5.89 to 1.23]	2.42	U	U	U	U	L	L
Heterogeneity: τ^2 = 0.00, I^2 = .%, H^2 = .			-2.33 [-5.89 to 1.23]							
Test of $\theta_i = \theta_j$: Q(0) = 0.00, p = .										
**04. Fruit**										
Agebratt et al. 2016	15	15	-4.90 [-9.80 to -0.00]	1.74	L	U	U	L	L	L
Amani et al. 2014	17	19	-10.95 [-23.84 to 1.94]	0.38	U	U	L	U	H	L
Beisner et al. 2020 (OB, fruit)	6	6	0.90 [-4.55 to 6.35]	1.52	H	H	L	L	L	H
Beisner et al. 2020 (lean, fruit)	6	6	1.70 [-4.38 to 7.78]	1.31	H	H	L	L	L	H
Du et al. 2019	22	25	-8.23 [-20.63 to 4.17]	0.41	U	U	L	U	U	L
Hegde et al. 2013	63	60	-3.60 [-10.27 to 3.07]	1.15	H	H	U	L	U	L
Lehtonen et al. 2011 (bilberry)	80	80	-0.20 [-2.74 to 2.34]	3.08	U	U	U	L	H	L
Madero et al. 2011	66	65	1.61 [-0.24 to 3.46]	3.55	L	L	L	U	L	L
Navaei et al. 2019	40	40	-2.00 [-5.52 to 1.52]	2.44	L	U	L	U	L	L
Puupponen-Pimia et al. 2013	12	20	3.30 [-7.07 to 13.67]	0.56	H	H	U	L	L	L
Singh et al. 1997	48	52	-8.10 [-11.09 to -5.11]	2.77	L	L	L	U	U	L
Heterogeneity: τ^2 = 11.97, I^2 = 73.97%, H^2 = 3.84			-2.13 [-4.79 to 0.53]							
Test of $\theta_i = \theta_j$: Q(10) = 38.41, p = 0.00										

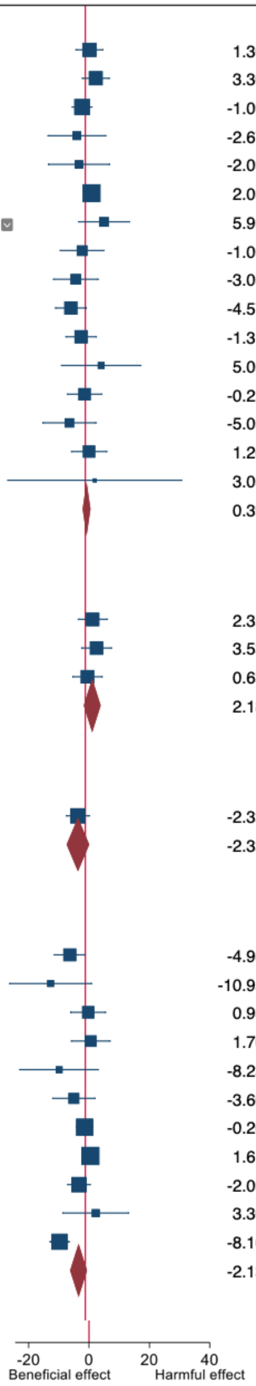

S5 Fig (2 of 3). Forest plot of controlled trials of the effect of important food sources of fructose-containing sugars on SBP (mmHg) in substitution trials.

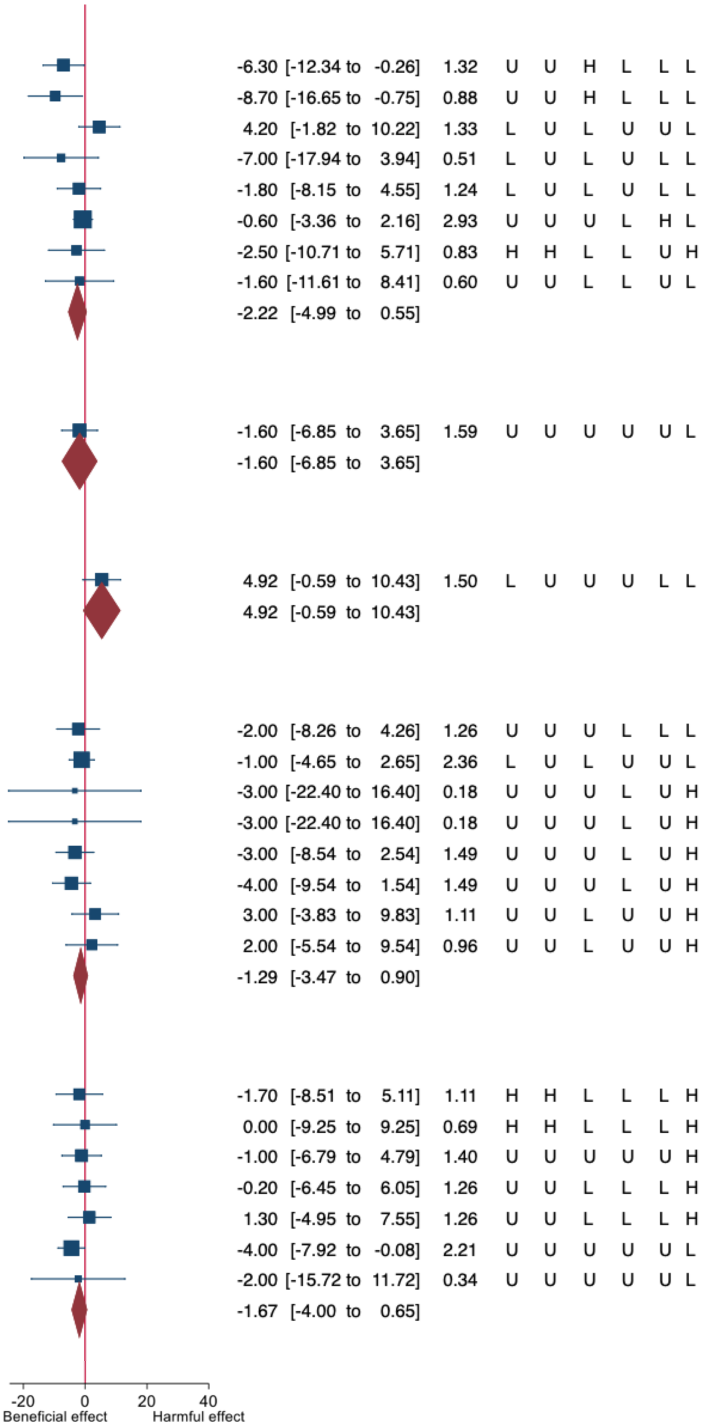

**05. Dried fruit**										
Anderson et al. 2014	15	31	-6.30 [-12.34 to -0.26]	1.32	U	U	H	L	L	L
Bays et al. 2015	19	27	-8.70 [-16.65 to -0.75]	0.88	U	U	H	L	L	L
Kaliora et al. 2016	21	23	4.20 [-1.82 to 10.22]	1.33	L	U	L	U	U	L
Kanellos et al. 2014	22	26	-7.00 [-17.94 to 3.94]	0.51	L	U	L	U	L	L
Kanellos et al. 2017	14	22	-1.80 [-8.15 to 4.55]	1.24	L	U	L	U	L	L
Lehtonen et al. 2011 (seaberry)	80	80	-0.60 [-3.36 to 2.16]	2.93	U	U	U	L	H	L
Puglisi et al. 2008 (run-in vs. R alone)	12	12	-2.50 [-10.71 to 5.71]	0.83	H	H	L	L	U	H
Puglisi et al. 2008 (walk vs. R+walk)	12	10	-1.60 [-11.61 to 8.41]	0.60	U	U	L	L	U	L
Heterogeneity: $\tau^2 = 4.89$, $I^2 = 32.94\%$, $H^2 = 1.49$			-2.22 [-4.99 to 0.55]							
Test of $\theta_i = \theta_j$: Q(7) = 10.44, p = 0.17										
**06. Mixed fruit forms**										
Lehtonen et al. 2010	22	28	-1.60 [-6.85 to 3.65]	1.59	U	U	U	U	U	L
Heterogeneity: $\tau^2 = 0.00$, $I^2 = .\%$, $H^2 = .$			-1.60 [-6.85 to 3.65]							
Test of $\theta_i = \theta_j$: Q(0) = -0.00, p = .										
**07. Sweetened cereal grains and bars**										
Jones et al. 2014	25	25	4.92 [-0.59 to 10.43]	1.50	L	U	U	U	L	L
Heterogeneity: $\tau^2 = 0.00$, $I^2 = .\%$, $H^2 = .$			4.92 [-0.59 to 10.43]							
Test of $\theta_i = \theta_j$: Q(0) = -0.00, p = .										
**08. Sweets and desserts**										
Claesson et al. 2009	13	12	-2.00 [-8.26 to 4.26]	1.26	U	U	U	L	L	L
Dikariyanto et a. 2020	54	51	-1.00 [-4.65 to 2.65]	2.36	L	U	L	U	U	L
Hallfrisch et al. 1983 (C, 15%)	12	12	-3.00 [-22.40 to 16.40]	0.18	U	U	U	L	U	H
Hallfrisch et al. 1983 (C, 7.5%)	12	12	-3.00 [-22.40 to 16.40]	0.18	U	U	U	L	U	H
Hallfrisch et al. 1983 (HI, 15%)	12	12	-3.00 [-8.54 to 2.54]	1.49	U	U	U	L	U	H
Hallfrisch et al. 1983 (HI, 7.5%)	12	12	-4.00 [-9.54 to 1.54]	1.49	U	U	U	L	U	H
Israel et al. 1983 (18%)	12	12	3.00 [-3.83 to 9.83]	1.11	U	U	L	U	U	H
Israel et al. 1983 (33%)	12	12	2.00 [-5.54 to 9.54]	0.96	U	U	L	U	U	H
Heterogeneity: $\tau^2 = 0.00$, $I^2 = 0.00\%$, $H^2 = 1.00$			-1.29 [-3.47 to 0.90]							
Test of $\theta_i = \theta_j$: Q(7) = 3.67, p = 0.82										
**09. Added nutritive (caloric) sweetener**										
Beisner et al. 2020 (OB, HFS)	6	6	-1.70 [-8.51 to 5.11]	1.11	H	H	L	L	L	H
Beisner et al. 2020 (lean, HFS)	6	6	0.00 [-9.25 to 9.25]	0.69	H	H	L	L	L	H
Cooper et al. 1988	17	17	-1.00 [-6.79 to 4.79]	1.40	U	U	U	U	U	H
Despland et al. 2017 (honey)	8	8	-0.20 [-6.45 to 6.05]	1.26	U	U	L	L	L	H
Despland et al. 2017 (sugars)	8	8	1.30 [-4.95 to 7.55]	1.26	U	U	L	L	L	H
Koh et al. 1988 (IGTS)	9	9	-4.00 [-7.92 to -0.08]	2.21	U	U	U	U	U	L
Koh et al. 1988 (NGTS)	9	9	-2.00 [-15.72 to 11.72]	0.34	U	U	U	U	U	L
Heterogeneity: $\tau^2 = 0.00$, $I^2 = 0.00\%$, $H^2 = 1.00$			-1.67 [-4.00 to 0.65]							
Test of $\theta_i = \theta_j$: Q(6) = 2.62, p = 0.86										

S5 Fig (3 of 3). Forest plot of controlled trials of the effect of important food sources of fructose-containing sugars on SBP (mmHg) in substitution trials.

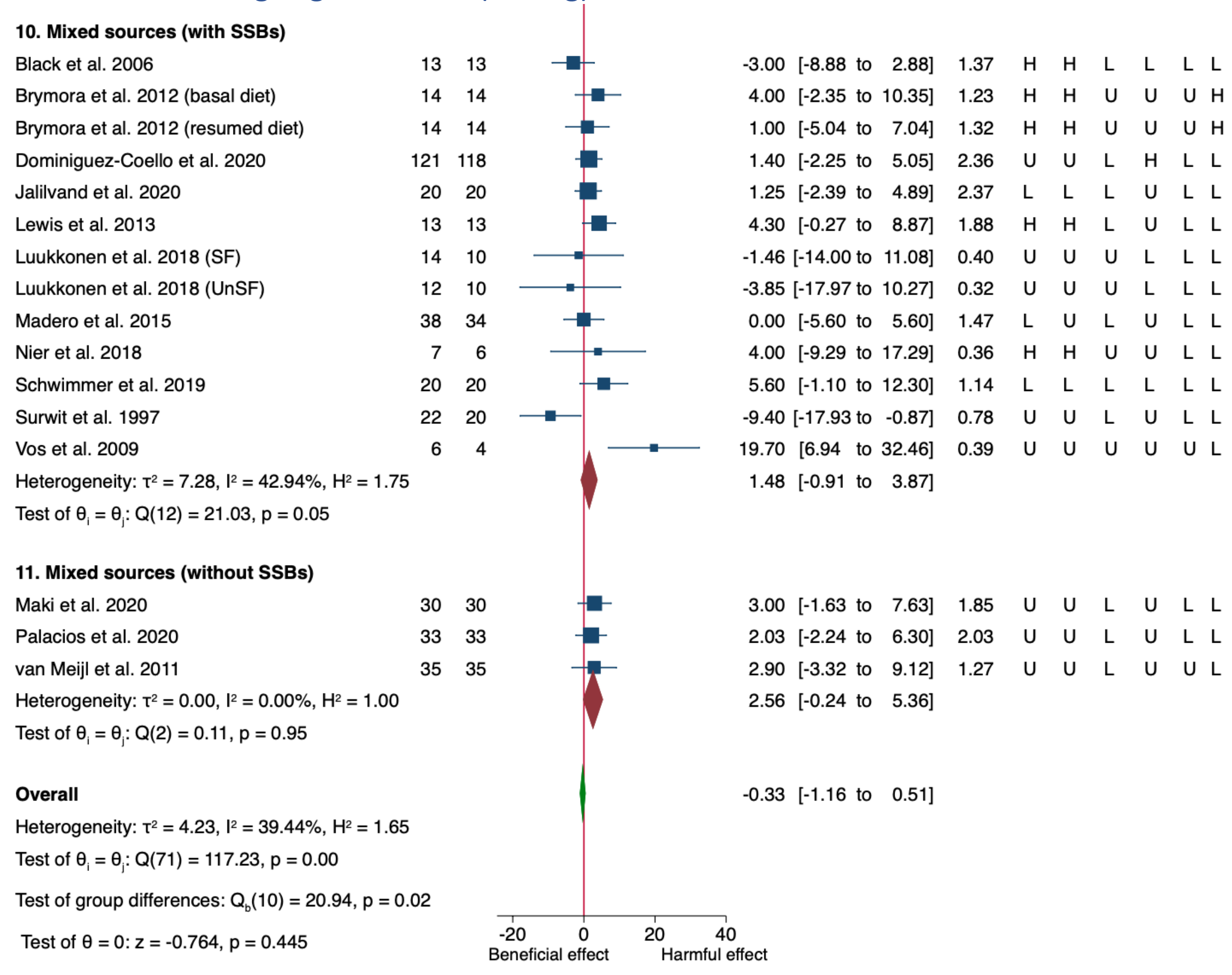

Pooled effect estimates for each subgroup and overall effect are represented by the diamonds. Data are expressed as weighted mean differences with 95% confidence intervals using the generic inverse-variance method and random effects DerSimonian-Laird model. Paired analyses were applied to all crossover trials.
Inter-study heterogeneity was assessed using the Cochrane Q statistic and quantified using the I^2 statistic, with significance set at p<0.100 and $I^2 \geq 50\%$ considered to be evidence of substantial heterogeneity. Pooled effect summary estimate was calculated with the χ^2 test. Test for group differences was calculated with meta-regression, which uses the Wald test.
Risk of Bias Legend: (H) High Risk; (L) Low Risk; (U) Unclear. The letters represent the following risk of bias domains: A, random sequence generation (selection bias); B, allocation concealment (selection bias); C, blinding of participants and personnel and outcome assessors (performance bias); D, incomplete outcome data (attrition bias); E, selective reporting (reporting bias); and F, other bias. High other risk of bias was given to crossover trials which had no washout between interventions. Trials which did not have this characteristic were rated as Low.
CI, confidence interval; C=control; FRC=fructose; Frc25= 25% fructose; GLC=glucose; Glc25= 25% glucose; HF=high fructose; HFCS25= 25% high fructose corn syrup; HFS=high fructose syrup; HG=high glucose; HI=hyperinsulinemic; HS=high sucrose; IGTS=impaired glucose-tolerant subjects; MF=medium fructose; MG=medium glucose; NGTS=normal glucose tolerant subjects; OB=obese; R=raisin; SF=saturated fat; SUC=sucrose; UnSF=unsaturated fat

S6 Fig (1 of 2). Forest plot of controlled trials of the effect of important food sources of fructose-containing sugars on SBP (mmHg) in addition trials.

Systolic Blood Pressure

Addition Trials

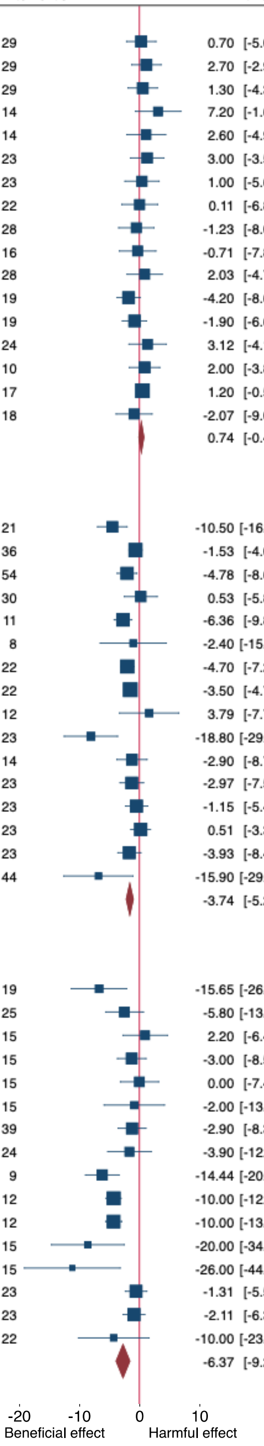

Trial or subgroup	N Control	N Intervention	Mean difference with 95% CI	Weight (%)	A	B	C	D	E	F
**1. SSB**										
Aeberli et al. 2011 (control vs. HF)	29	29	0.70 [-5.01 to 6.41]	1.70	U	U	L	L	L	L
Aeberli et al. 2011 (control vs. HS)	29	29	2.70 [-2.98 to 8.38]	1.71	U	U	L	L	L	L
Aeberli et al. 2011 (control vs. MF)	29	29	1.30 [-4.35 to 6.95]	1.71	U	U	L	L	L	L
Engel et al. 2017 (NSB)	15	14	7.20 [-1.61 to 16.01]	1.09	U	U	H	U	L	L
Engel et al. 2017 (water)	16	14	2.60 [-4.99 to 10.19]	1.30	U	U	H	U	L	L
Geidl-Flueck et al. 2021 (CNTRL vs FRC)	24	23	3.00 [-3.55 to 9.55]	1.51	L	U	L	U	L	L
Geidl-Flueck et al. 2021 (CNTRL vs SUC)	24	23	1.00 [-5.65 to 7.65]	1.49	L	U	L	U	L	L
Hieronimus et al. 2020 (Frc17.5)	23	22	0.11 [-6.86 to 7.08]	1.42	H	H	L	U	L	L
Hieronimus et al. 2020 (Frc25)	23	28	-1.23 [-8.05 to 5.59]	1.45	H	H	L	U	L	L
Hieronimus et al. 2020 (HFCS17.5)	23	16	-0.71 [-7.80 to 6.38]	1.39	H	H	L	U	L	L
Hieronimus et al. 2020 (HFCS25)	23	28	2.03 [-4.77 to 8.83]	1.45	H	H	L	U	L	L
Njike et al. 2011 (placebo)	19	19	-4.20 [-8.64 to 0.24]	2.02	L	U	L	U	L	L
Njike et al. 2011 (sugar-sweetened)	19	19	-1.90 [-6.64 to 2.84]	1.95	L	U	L	U	L	L
Sigala et al. 2020	23	24	3.12 [-4.12 to 10.36]	1.36	H	H	L	U	U	L
Silbernagel et al. 2011	10	10	2.00 [-3.88 to 7.88]	1.66	H	H	L	U	U	H
Stanhope et al. 2009	17	17	1.20 [-0.55 to 2.95]	2.69	H	H	L	U	U	H
Stanhope et al. 2015 (HFCS10)	23	18	-2.07 [-9.06 to 4.92]	1.41	H	H	L	U	L	L
Heterogeneity: $\tau^2 = 0.00$, $I^2 = 0.00\%$, $H^2 = 1.00$			0.74 [-0.42 to 1.90]							
Test of $\theta_i = \theta_j$: Q(16) = 11.33, p = 0.79										
**2. 100% Fruit juice**										
Abedini et al. 2020	21	21	-10.50 [-16.24 to -4.76]	1.69	L	L	L	U	L	L
Aghababaee et al. 2015	36	36	-1.53 [-4.06 to 1.00]	2.52	L	U	U	L	U	L
Alatas et al. 2019	58	54	-4.78 [-8.61 to -0.95]	2.19	L	U	L	U	L	L
Amagase et al. 2009	30	30	0.53 [-5.81 to 6.87]	1.55	U	L	L	L	U	L
Asgary et al. 2014	10	11	-6.36 [-9.84 to -2.88]	2.28	H	H	L	L	L	L
Banini et al. 2006	15	8	-2.40 [-15.22 to 10.42]	0.64	U	U	U	L	U	L
Esmaeilinezhad et al. 2020 (PJ vs. placebo)	21	22	-4.70 [-7.27 to -2.13]	2.51	H	H	L	U	L	L
Esmaeilinezhad et al. 2020 (SPJ vs. SB)	21	22	-3.50 [-4.70 to -2.30]	2.78	H	H	L	U	L	L
Kojadinovic et al. 2017	11	12	3.79 [-7.71 to 15.29]	0.76	U	U	U	U	U	L
Lazavi et al. 2018	23	23	-18.80 [-29.15 to -8.45]	0.88	L	L	U	L	L	L
Neto et al. 2017	12	14	-2.90 [-8.77 to 2.97]	1.66	U	U	L	U	U	L
Ravn-Haren et al. 2013 (control vs. clear juice)	23	23	-2.97 [-7.56 to 1.62]	1.99	U	U	L	U	U	H
Ravn-Haren et al. 2013 (control vs. cloudy juice)	23	23	-1.15 [-5.45 to 3.15]	2.06	U	U	L	U	U	H
Ravn-Haren et al. 2013 (pomace vs. clear juice)	23	23	0.51 [-3.32 to 4.34]	2.19	U	U	L	U	U	H
Ravn-Haren et al. 2013 (pomace vs. cloudy juice)	23	23	-3.93 [-8.44 to 0.58]	2.01	U	U	L	U	U	H
Shema-Didi et al. 2014	21	44	-15.90 [-29.27 to -2.53]	0.60	U	U	L	L	L	L
Heterogeneity: $\tau^2 = 4.03$, $I^2 = 53.45\%$, $H^2 = 2.15$			-3.74 [-5.28 to -2.20]							
Test of $\theta_i = \theta_j$: Q(15) = 32.22, p = 0.01										
**3. Fruit**										
Amani et al. 2014	19	19	-15.65 [-26.37 to -4.93]	0.84	H	H	U	L	H	H
Basu et al. 2010 (blueberry)	23	25	-5.80 [-13.16 to 1.56]	1.34	U	U	L	U	L	L
Basu et al. 2010 (strawberry)	12	15	2.20 [-6.44 to 10.84]	1.12	U	U	U	U	L	L
Cressey et al. 2014 (HCL, 250g)	15	15	-3.00 [-8.55 to 2.55]	1.74	H	H	U	L	U	H
Cressey et al. 2014 (HCL, 500g)	15	15	0.00 [-7.40 to 7.40]	1.33	H	H	U	L	U	H
Cressey et al. 2014 (T2D, 250g)	15	15	-2.00 [-13.72 to 9.72]	0.74	H	H	U	L	U	H
Dow et al. 2012	32	39	-2.90 [-8.31 to 2.51]	1.77	U	U	U	L	L	L
Franck et al. 2020	24	24	-3.90 [-12.34 to 4.54]	1.15	U	U	U	U	L	L
Karlsen et al. 2013 (hypertensives)	11	9	-14.44 [-20.95 to -7.92]	1.52	U	U	U	U	L	L
Karlsen et al. 2013 (normal/high-normal BP)	12	12	-10.00 [-12.94 to -7.06]	2.42	U	U	U	U	L	L
Karlsen et al. 2013 (optimal BP)	11	12	-10.00 [-13.12 to -6.88]	2.38	U	U	U	U	L	L
Kumari et al. 2016 (with peel)	15	15	-20.00 [-34.04 to -5.96]	0.56	L	U	U	U	U	L
Kumari et al. 2016 (without peel)	15	15	-26.00 [-44.54 to -7.46]	0.35	L	U	U	U	U	L
Ravn-Haren et al. 2013 (control vs. whole apple)	23	23	-1.31 [-5.53 to 2.91]	2.09	U	U	L	U	U	H
Ravn-Haren et al. 2013 (pomace vs. whole apple)	23	23	-2.11 [-6.31 to 2.09]	2.09	U	U	L	U	U	H
Schell et al. 2019	22	22	-10.00 [-23.72 to 3.72]	0.58	U	U	L	L	L	L
Heterogeneity: $\tau^2 = 19.07$, $I^2 = 68.65\%$, $H^2 = 3.19$			-6.37 [-9.21 to -3.52]							
Test of $\theta_i = \theta_j$: Q(15) = 47.85, p = 0.00										

Column heading for A–F: Risk of bias

S6 Fig (2 of 2). Forest plot of controlled trials of the effect of important food sources of fructose-containing sugars on SBP (mmHg) in addition trials.

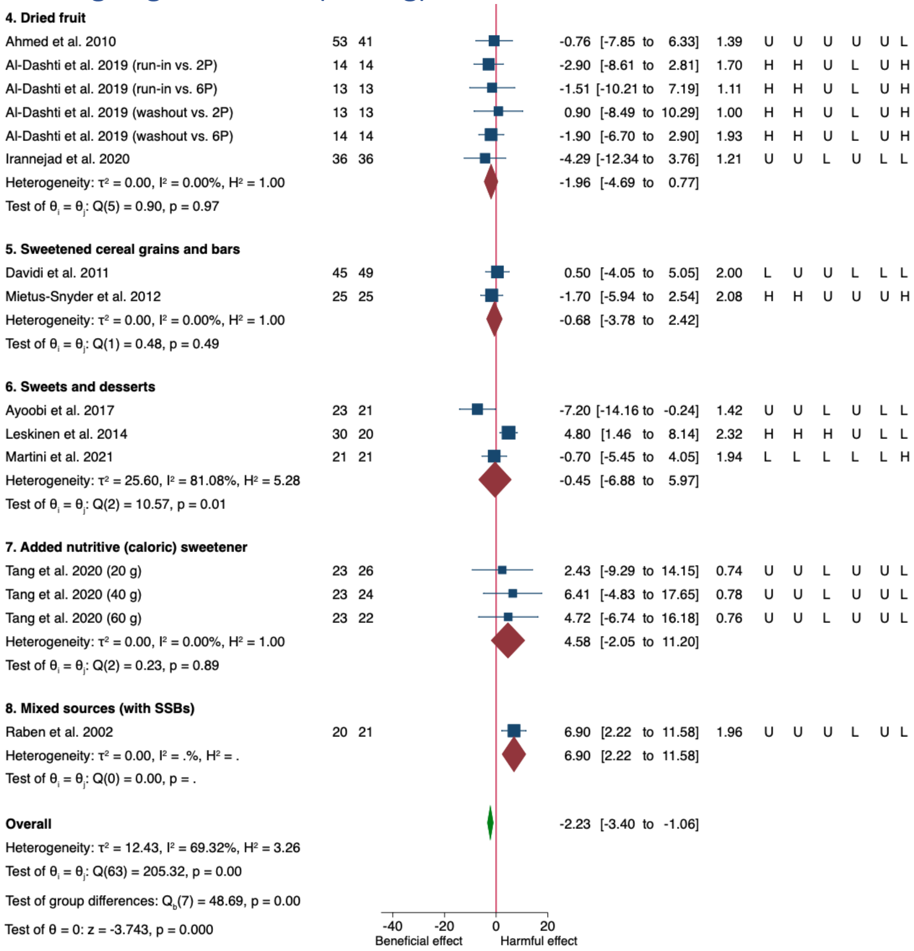

Pooled effect estimates for each subgroup and overall effect are represented by the diamonds. Data are expressed as weighted mean differences with 95% confidence intervals using the generic inverse-variance method and random effects DerSimonian-Laird model. Paired analyses were applied to all crossover trials. Inter-study heterogeneity was assessed using the Cochrane Q statistic and quantified using the I^2 statistic, with significance set at p<0.100 and I^2≥50% considered to be evidence of substantial heterogeneity. Pooled effect summary estimate was calculated with the χ^2 test. Test for group differences was calculated with meta-regression, which uses the Wald test.

Risk of Bias Legend: (H) High Risk; (L) Low Risk; (U) Unclear. The letters represent the following risk of bias domains: A, random sequence generation (selection bias); B, allocation concealment (selection bias); C, blinding of participants and personnel and outcome assessors (performance bias); D, incomplete outcome data (attrition bias); E, selective reporting (reporting bias); and F, other bias. High other risk of bias was given to crossover trials which had no washout between interventions. Trials which did not have this characteristic were rated as Low.

CI, confidence interval; CNTRL=control; FRC=fructose; Frc17.5= 17.5% fructose; Frc25=25% fructose; HCL=hypercholesterolemic; HFCS10=10% high fructose corn syrup HFCS17.5=17.5% high fructose corn syrup; HFCS25=25% high fructose corn syrup; HF=high fructose; HS=high sucrose; MF=medium fructose; NSB=non-nutritive sweetened beverage; P=prunes; PF=pomegranate juice; SB=symbiotic beverage; SPJ=symbiotic pomegranate juice; SUC=sucrose; T2D=type 2 diabetes

S7a Fig. Forest plot of controlled trials of the effect of important food sources of fructose-containing sugars on SBP (mmHg) in subtraction trials.

Systolic Blood Pressure

Subtraction Trials

Trial or subgroup	N Control	N Intervention	Mean difference with 95% CI	Weight (%)	A	B	C	D	E	F
**1. SSB**										
Al-Dujaili et al. 2017	16	16	4.60 [1.54 to 7.66]	12.09	U	U	U	L	U	L
Campos et al. 2015 (G1)	6	6	-5.60 [-21.43 to 10.23]	1.13	U	U	U	U	L	L
Campos et al. 2015 (G2)	7	8	-1.90 [-14.26 to 10.46]	1.78	U	U	U	U	L	L
Ebbeling et al. 2020 (ASB)	60	60	-2.40 [-6.81 to 2.01]	8.59	U	U	L	L	L	L
Ebbeling et al. 2020 (water)	60	66	0.00 [-4.30 to 4.30]	8.83	U	U	L	L	L	L
Hernandez-Cordero et al. 2014	120	120	-1.40 [-3.06 to 0.26]	16.46	L	H	H	L	L	L
Tate et al. 2012 (NSB)	75	84	-1.90 [-7.20 to 3.40]	6.89	L	L	L	U	L	L
Tate et al. 2012 (water)	75	87	-2.70 [-8.07 to 2.67]	6.77	L	L	L	U	L	L
Heterogeneity: $\tau^2 = 4.24$, $I^2 = 49.72\%$, $H^2 = 1.99$			-0.47 [-2.70 to 1.75]							
Test of $\theta_i = \theta_j$: Q(7) = 13.92, p = 0.05										
**2. Mixed sources (with SSBs)**										
Vazquez-Duran et al. 2016 (NSB)	49	49	-0.85 [-1.58 to -0.13]	18.76	L	U	L	U	L	L
Vazquez-Duran et al. 2016 (water)	49	48	-3.81 [-4.58 to -3.05]	18.69	L	U	L	U	L	L
Heterogeneity: $\tau^2 = 4.24$, $I^2 = 96.69\%$, $H^2 = 30.20$			-2.33 [-5.23 to 0.57]							
Test of $\theta_i = \theta_j$: Q(1) = 30.20, p = 0.00										
**Overall**			-1.16 [-2.90 to 0.57]							
Heterogeneity: $\tau^2 = 4.03$, $I^2 = 82.23\%$, $H^2 = 5.63$										
Test of $\theta_i = \theta_j$: Q(9) = 50.64, p = 0.00										
Test of group differences: p = 0.319, $I^2 = 81.87\%$, p_Q <0.001										
Test of $\theta = 0$: z = -1.317, p = 0.188										

(Columns A–F: Risk of bias)

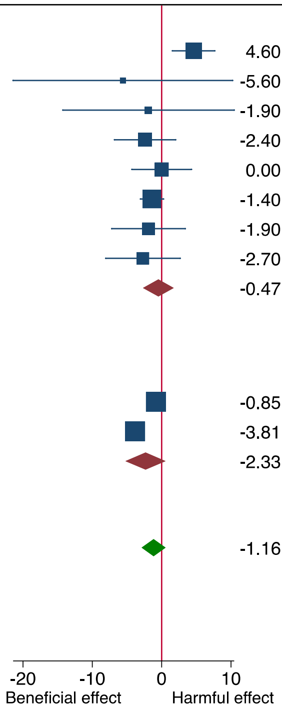

Pooled effect estimates for each subgroup and overall effect are represented by the diamonds. Data are expressed as weighted mean differences with 95% confidence intervals using the generic inverse-variance method and random effects DerSimonian-Laird model. Paired analyses were applied to all crossover trials. Inter-study heterogeneity was assessed using the Cochrane Q statistic and quantified using the I^2 statistic, with significance set at p<0.100 and $I^2 \geq 50\%$ considered to be evidence of substantial heterogeneity. Pooled effect summary estimate was calculated with the χ^2 test. Test for group differences was calculated with meta-regression, which uses the Wald test.

Risk of Bias Legend: (H) High Risk; (L) Low Risk; (U) Unclear. The letters represent the following risk of bias domains: A, random sequence generation (selection bias); B, allocation concealment (selection bias); C, blinding of participants and personnel and outcome assessors (performance bias); D, incomplete outcome data (attrition bias); E, selective reporting (reporting bias); and F, other bias. High other risk of bias was given to crossover trials which had no washout between interventions. Trials which did not have this characteristic were rated as Low.

CI, confidence interval; ASB=artificially sweetened beverage; G1=group 1; G2=group 2; NSB=non-nutritive sweetened beverage

S7b Fig. Forest plot of controlled trials of the effect of mixed sources (with SSBs) on SBP (mmHg) in subtraction trials*.

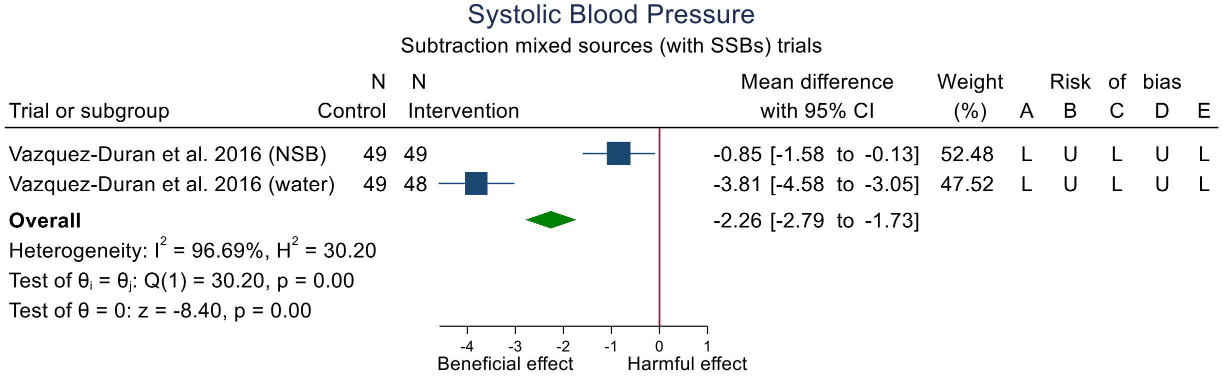

Test of θ = 0: z = -8.399, p = 0.000

* In the rare occasion when all the individual study estimates were significant and on one side of unity, but the 95% CI of the pooled random-effects estimate included zero, suggested an over dispersion of the between study variance (tau), a well-known limitation of the random-effects model[1], we performed a fixed effects model.
Pooled effect estimates for each subgroup and overall effect are represented by the diamonds. Data are expressed as weighted mean differences with 95% confidence intervals using the generic inverse-variance method and fixed effects model. Paired analyses were applied to all crossover trials. Inter-study heterogeneity was assessed using the Cochrane Q statistic and quantified using the I^2 statistic, with significance set at $p<0.100$ and $I^2 \geq 50\%$ considered to be evidence of substantial heterogeneity. Pooled effect summary estimate was calculated with the χ^2 test. Test for group differences was calculated with meta-regression, which uses the Wald test.
Risk of Bias Legend: (H) High Risk; (L) Low Risk; (U) Unclear. The letters represent the following risk of bias domains: A, random sequence generation (selection bias); B, allocation concealment (selection bias); C, blinding of participants and personnel and outcome assessors (performance bias); D, incomplete outcome data (attrition bias); E, selective reporting (reporting bias); and F, other bias. High other risk of bias was given to crossover trials which had no washout between interventions. Trials which did not have this characteristic were rated as Low.
CI, confidence interval; ASB=artificially sweetened beverage; G1=group 1; G2=group 2; NSB=non-nutritive sweetened beverage

S8 Fig. Forest plot of controlled trials of the effect of important food sources of fructose-containing sugars on SBP (mmHg) in *ad libitum* trials.

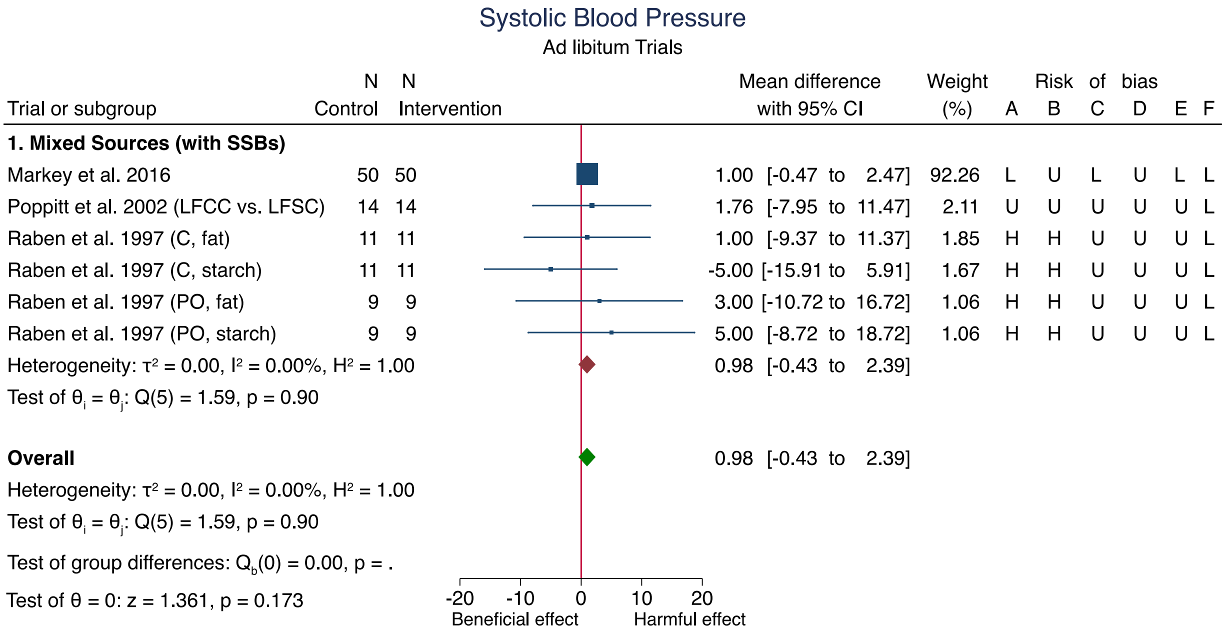

Pooled effect estimates for each subgroup and overall effect are represented by the diamonds. Data are expressed as weighted mean differences with 95% confidence intervals using the generic inverse-variance method and random effects DerSimonian-Laird model. Paired analyses were applied to all crossover trials. Inter-study heterogeneity was assessed using the Cochrane Q statistic and quantified using the I^2 statistic, with significance set at p<0.100 and $I^2 \geq 50\%$ considered to be evidence of substantial heterogeneity. Pooled effect summary estimate was calculated with the χ^2 test. Test for group differences was calculated with meta-regression, which uses the Wald test.

Risk of Bias Legend: (H) High Risk; (L) Low Risk; (U) Unclear. The letters represent the following risk of bias domains: A, random sequence generation (selection bias); B, allocation concealment (selection bias); C, blinding of participants and personnel and outcome assessors (performance bias); D, incomplete outcome data (attrition bias); E, selective reporting (reporting bias); and F, other bias. High other risk of bias was given to crossover trials which had no washout between interventions. Trials which did not have this characteristic were rated as Low.

CI, confidence interval; C=control; LFCC=low fat complex carbohydrates; LFSC=low fat simple carbohydrates; PO=post obese

S9 Fig (1 of 3). Forest plot of controlled trials of the effect of important food sources of fructose-containing sugars on DBP (mmHg) in substitution trials.

Diastolic Blood Pressure

SubstitutionTrials

Trial or subgroup	N Control	N Intervention	Mean difference with 95% CI	Weight (%)	A	B	C	D	E	F
**01. SSB**										
Aeberli et al. 2011 (HG vs. HF)	29	29	2.70 [-1.39 to 6.79]	1.45	U	U	L	L	L	L
Aeberli et al. 2011 (HG vs. HS)	29	29	1.20 [-2.10 to 4.50]	1.80	U	U	L	L	L	L
Aeberli et al. 2011 (MG vs. MF)	29	29	0.00 [-2.27 to 2.27]	2.37	U	U	L	L	L	L
Aeberli et al. 2013 (HG vs HF)	9	9	0.00 [-6.84 to 6.84]	0.72	U	L	L	L	H	L
Aeberli et al. 2013 (HG vs HS)	9	9	1.89 [-7.05 to 10.83]	0.46	U	L	L	L	H	L
Chiu et al., 2020	30	30	0.00 [-1.89 to 1.89]	2.59	L	L	L	L	L	L
Engel et al. 2018	15	14	6.30 [0.27 to 12.33]	0.87	U	U	H	U	L	L
Geidl-Flueck et al. 2021 (GLC vs FRC)	22	23	2.80 [-4.11 to 9.71]	0.71	L	U	L	U	L	L
Geidl-Flueck et al. 2021 (GLC vs SUC)	22	23	1.50 [-4.68 to 7.68]	0.84	L	U	L	U	L	L
Hieronimus et al. 2020 (Frc25 vs. Glc 25)	28	28	-0.75 [-4.35 to 2.85]	1.66	H	H	L	U	L	L
Hieronimus et al. 2020 (HFCS25 vs. Glc25)	28	28	0.84 [-2.75 to 4.43]	1.67	H	H	L	U	L	L
Koivisto & Yki-Jävinen 1993	10	10	0.00 [-7.84 to 7.84]	0.58	U	U	L	U	U	L
Sigala et al. 2020	28	24	1.57 [-2.45 to 5.59]	1.48	H	H	L	U	U	L
Silbernagel et al. 2011	10	10	-3.00 [-11.77 to 5.77]	0.48	L	L	L	U	U	L
Stanhope et al. 2009	15	17	0.78 [-1.99 to 3.55]	2.08	H	H	L	U	U	L
Swarbrick et al. 2008	7	7	-1.00 [-24.52 to 22.52]	0.08	H	H	L	L	U	L
Heterogeneity: $\tau^2 = 0.00$, $I^2 = 0.00\%$, $H^2 = 1.00$			0.66 [-0.29 to 1.61]							
Test of $\theta_i = \theta_j$: Q(15) = 7.28, p = 0.95										
**02. Sweetened dairy**										
Angelopoulos et al. 2015 (FRC)	77	65	-0.53 [-4.57 to 3.51]	1.47	U	U	L	U	L	L
Angelopoulos et al. 2015 (HFCS)	77	61	0.75 [-3.54 to 5.04]	1.37	U	U	L	U	L	L
Angelopoulos et al. 2015 (SUC)	77	64	-1.51 [-5.56 to 2.54]	1.46	U	U	L	U	L	L
Heterogeneity: $\tau^2 = 0.00$, $I^2 = 0.00\%$, $H^2 = 1.00$			-0.47 [-2.85 to 1.91]							
Test of $\theta_i = \theta_j$: Q(2) = 0.56, p = 0.75										
**03. Sweetened dairy alternative (soy)**										
Maleki et al. 2019	31	31	-1.45 [-3.75 to 0.85]	2.35	U	U	U	U	L	L
Heterogeneity: $\tau^2 = 0.00$, $I^2 = .\%$, $H^2 = .$			-1.45 [-3.75 to 0.85]							
Test of $\theta_i = \theta_j$: Q(0) = -0.00, p = .										
**04. Fruit**										
Agebratt et al. 2016	15	15	-1.50 [-5.61 to 2.61]	1.44	L	U	U	L	L	L
Amani et al. 2014	17	19	-3.20 [-7.56 to 1.16]	1.35	U	U	L	U	H	L
Beisner et al. 2020 (lean, HFS)	6	6	5.80 [0.98 to 10.62]	1.19	H	H	L	L	L	H
Beisner et al. 2020 (lean, fruit)	6	6	6.70 [1.76 to 11.64]	1.15	H	H	L	L	L	H
Du et al. 2019	22	25	-2.44 [-7.85 to 2.96]	1.02	U	U	L	U	U	L
Hegde et al. 2013	63	60	-1.50 [-4.81 to 1.81]	1.80	H	H	U	L	U	L
Lehtonen et al. 2011 (bilberry)	80	80	-0.20 [-1.94 to 1.54]	2.69	U	U	U	L	H	L
Madero et al. 2011	66	65	-0.87 [-3.86 to 2.12]	1.96	L	L	L	U	L	L
Navaei et al. 2019	40	40	1.00 [-0.96 to 2.96]	2.55	L	U	L	U	L	L
Puupponen-Pimia et al. 2013	12	20	0.20 [-5.96 to 6.36]	0.85	H	H	U	L	L	L
Singh et al. 1997	48	52	-6.80 [-8.39 to -5.21]	2.77	L	L	L	U	U	L
Heterogeneity: $\tau^2 = 13.35$, $I^2 = 85.96\%$, $H^2 = 7.12$			-0.48 [-2.92 to 1.97]							
Test of $\theta_i = \theta_j$: Q(10) = 71.22, p = 0.00										

Risk of bias: columns A–F

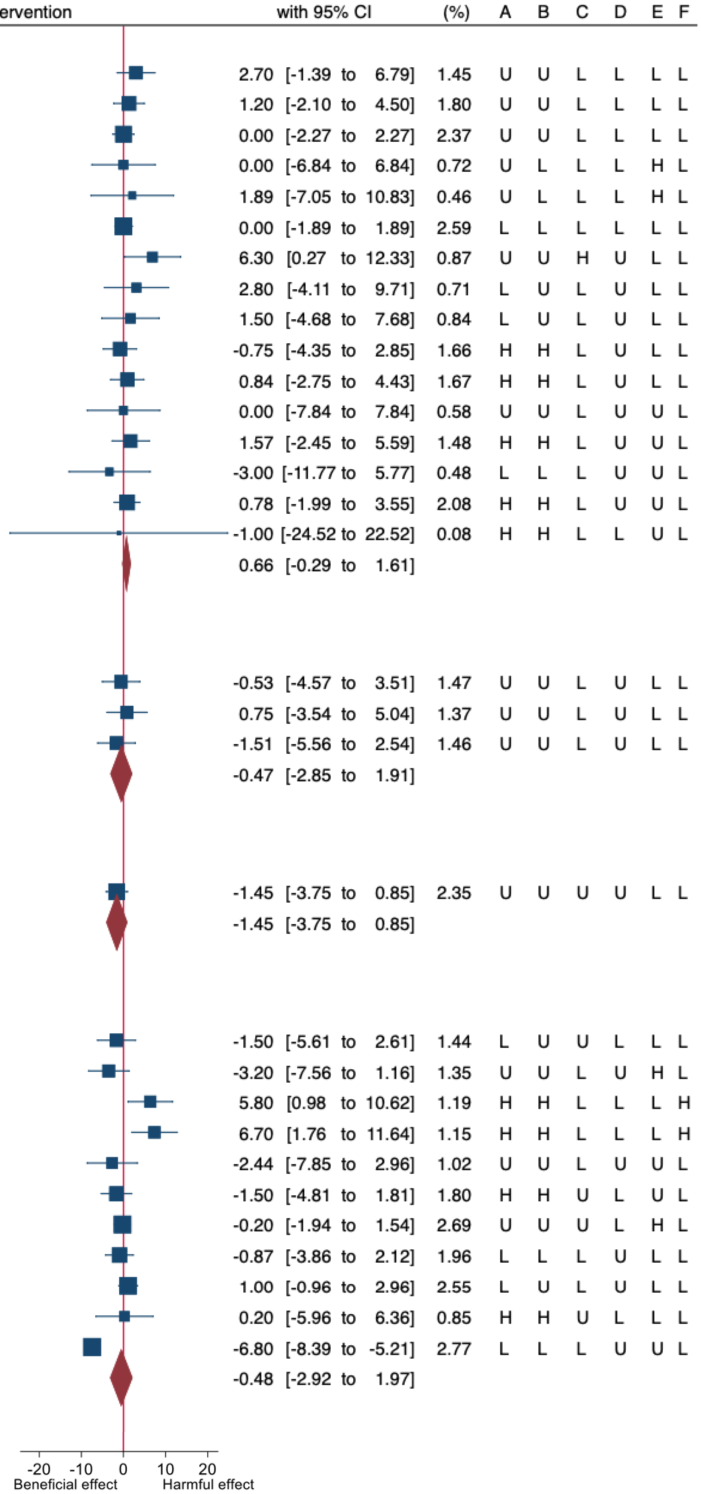

S9 Fig (2 of 3). Forest plot of controlled trials of the effect of important food sources of fructose-containing sugars on DBP (mmHg) in substitution trials.

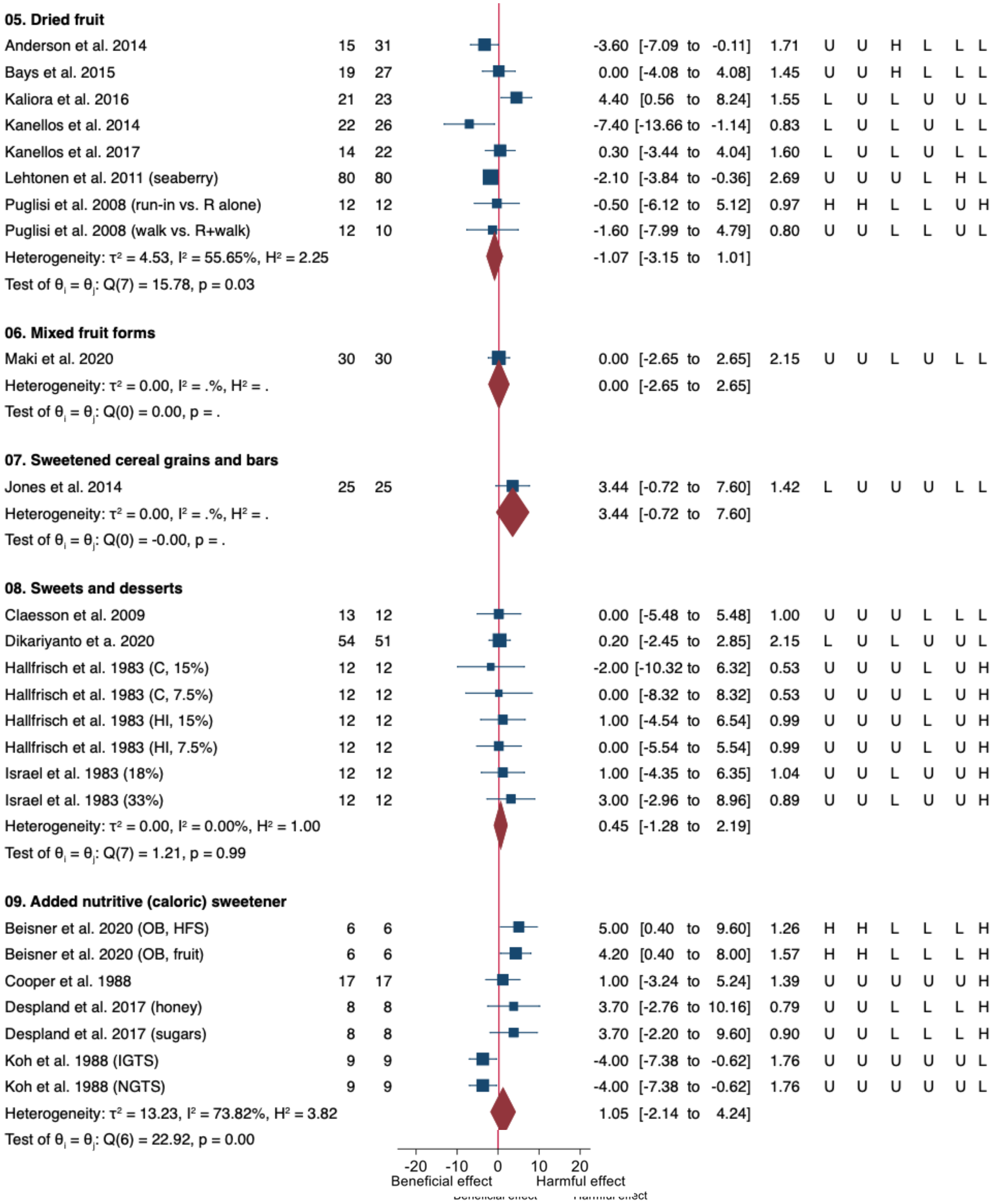

S9 Fig (3 of 3). Forest plot of controlled trials of the effect of important food sources of fructose-containing sugars on DBP (mmHg) in substitution trials.

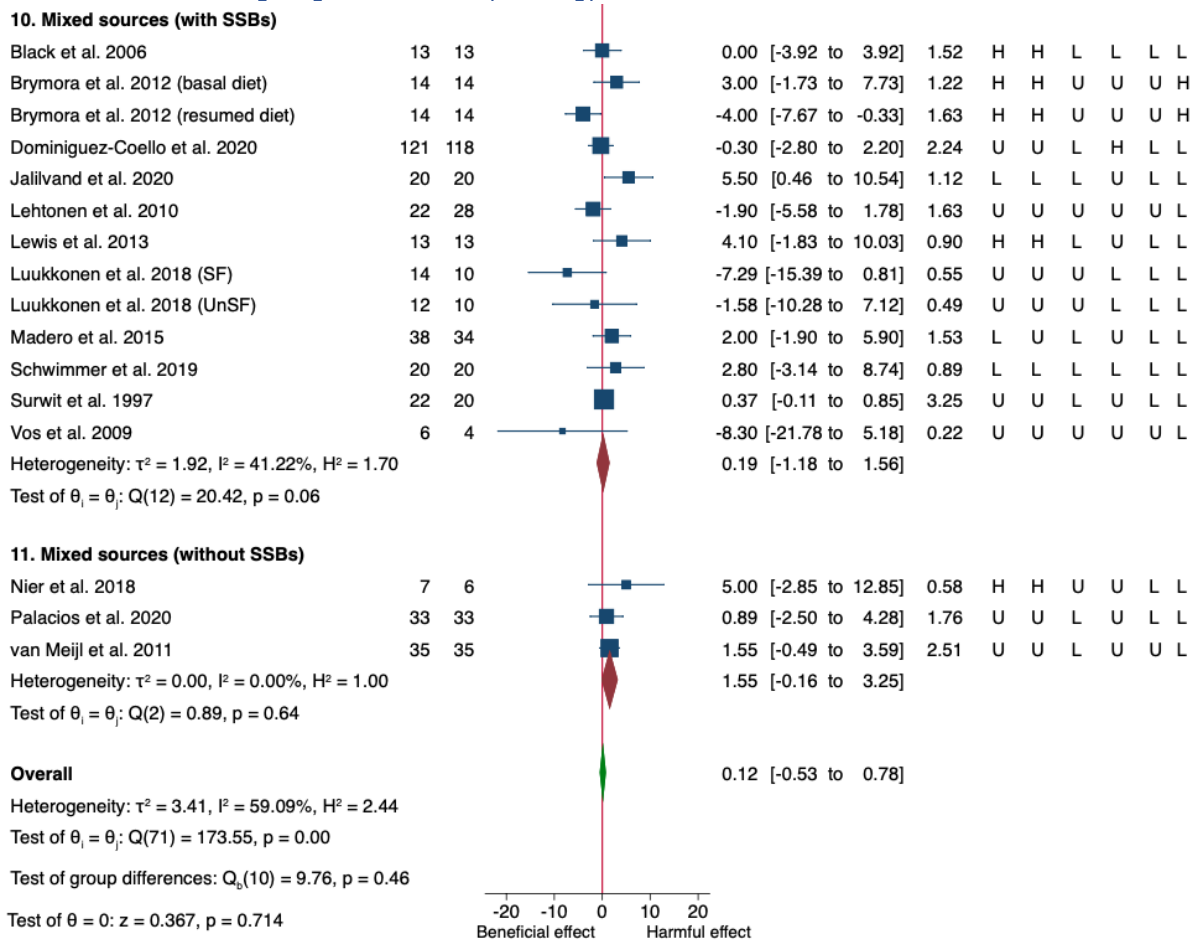

Pooled effect estimates for each subgroup and overall effect are represented by the diamonds. Data are expressed as weighted mean differences with 95% confidence intervals using the generic inverse-variance method and random effects DerSimonian-Laird model. Paired analyses were applied to all crossover trials. Inter-study heterogeneity was assessed using the Cochrane Q statistic and quantified using the I^2 statistic, with significance set at p<0.100 and I^2≥50% considered to be evidence of substantial heterogeneity. Pooled effect summary estimate was calculated with the χ^2 test. Test for group differences was calculated with meta-regression, which uses the Wald test.

Risk of Bias Legend: (H) High Risk; (L) Low Risk; (U) Unclear. The letters represent the following risk of bias domains: A, random sequence generation (selection bias); B, allocation concealment (selection bias); C, blinding of participants and personnel and outcome assessors (performance bias); D, incomplete outcome data (attrition bias); E, selective reporting (reporting bias); and F, other bias. High other risk of bias was given to crossover trials which had no washout between interventions. Trials which did not have this characteristic were rated as Low.

CI, confidence interval; C=control; FRC=fructose; Frc25= 25% fructose; GLC=glucose; Glc25= 25% glucose; HF=high fructose; HFCS25= 25% high fructose corn syrup; HFS=high fructose syrup; HG=high glucose; HI=hyperinsulinemic; HS=high sucrose; IGTS=impaired glucose-tolerant subjects; MF=medium fructose; MG=medium glucose; NGTS=normal glucose tolerant subjects; OB=obese; R=raisin; SF=saturated fat; SUC=sucrose; UnSF=unsaturated fat

S10 Fig (1 of 2). Forest plot of controlled trials of the effect of important food sources of fructose-containing sugars on DBP (mmHg) in addition trials.

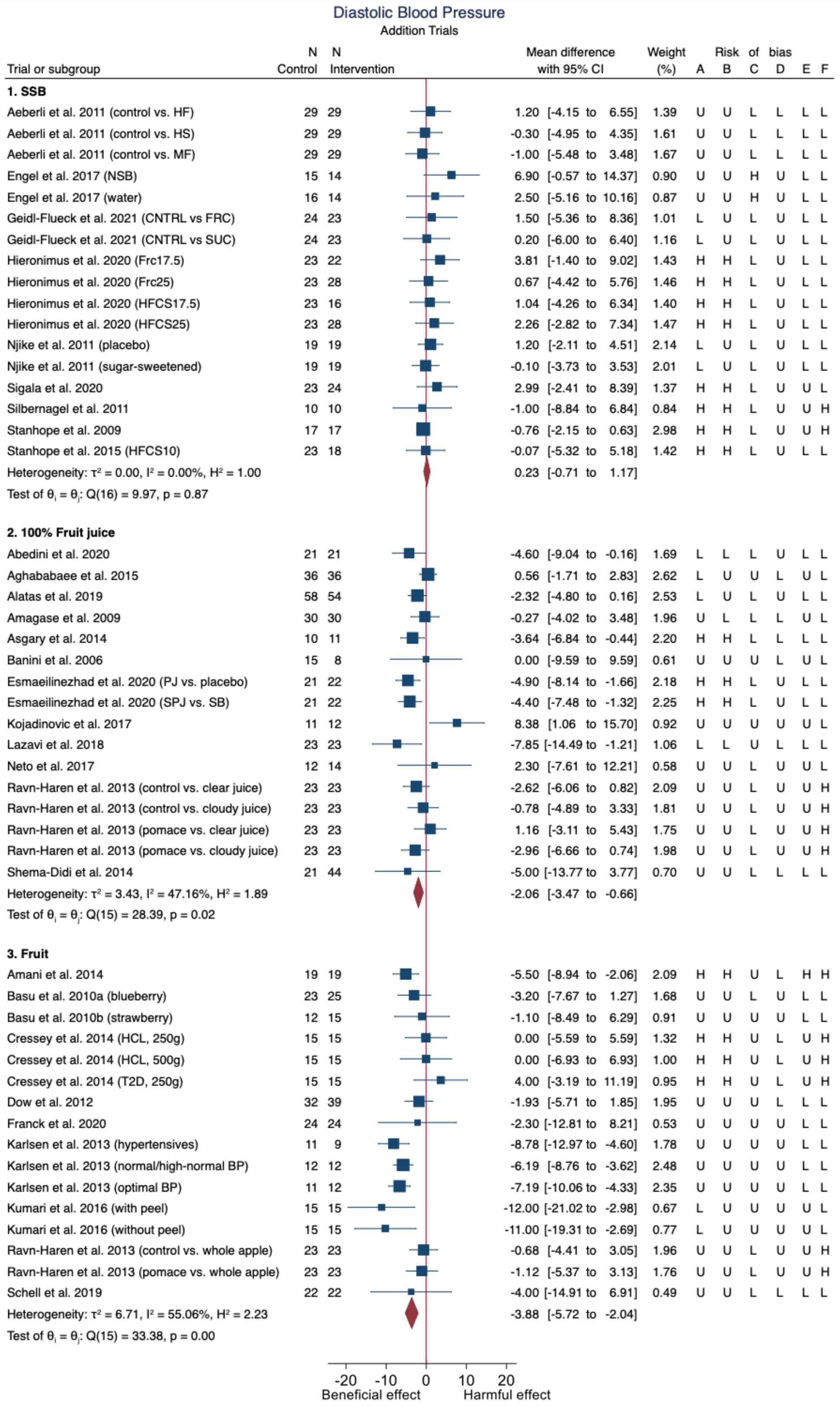

S10 Fig (2 of 2). Forest plot of controlled trials of the effect of important food sources of fructose-containing sugars on DBP (mmHg) in addition trials.

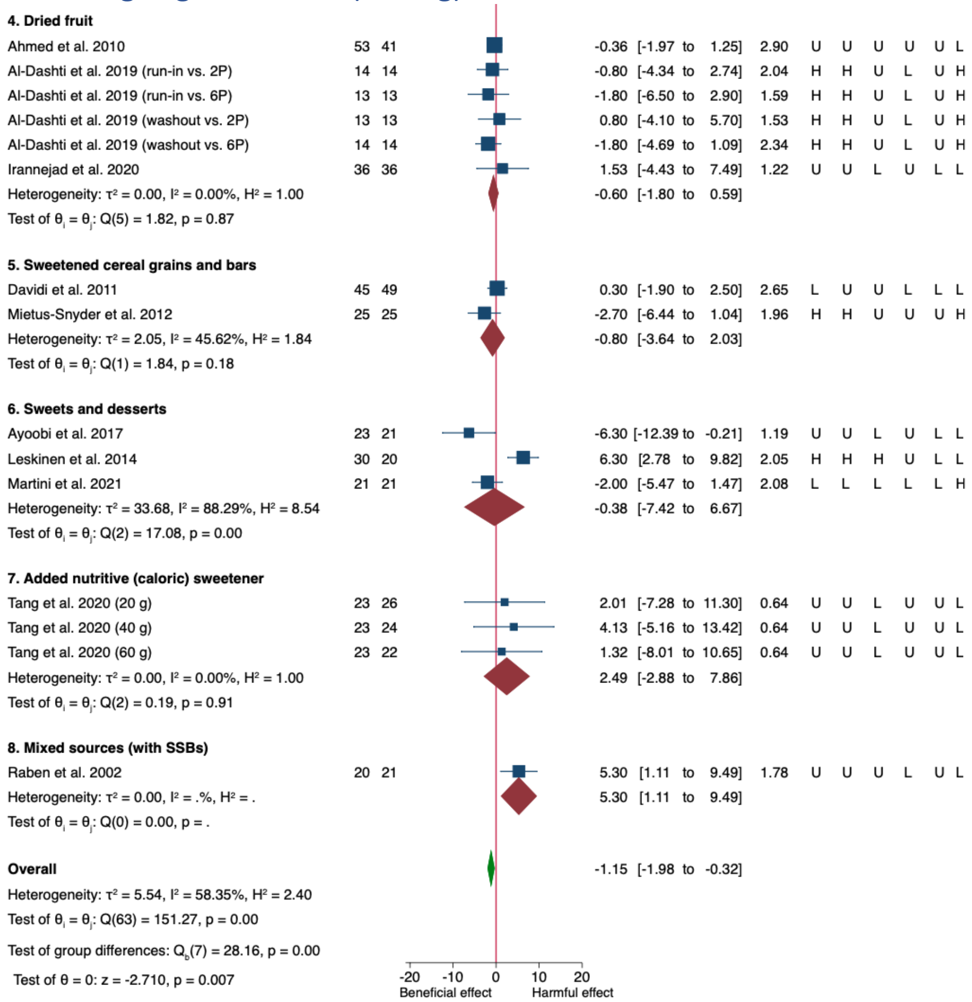

Pooled effect estimates for each subgroup and overall effect are represented by the diamonds. Data are expressed as weighted mean differences with 95% confidence intervals using the generic inverse-variance method and random effects DerSimonian-Laird model. Paired analyses were applied to all crossover trials. Inter-study heterogeneity was assessed using the Cochrane Q statistic and quantified using the I^2 statistic, with significance set at p<0.100 and $I^2 \geq 50\%$ considered to be evidence of substantial heterogeneity. Pooled effect summary estimate was calculated with the χ^2 test. Test for group differences was calculated with meta-regression, which uses the Wald test.

Risk of Bias Legend: (H) High Risk; (L) Low Risk; (U) Unclear. The letters represent the following risk of bias domains: A, random sequence generation (selection bias); B, allocation concealment (selection bias); C, blinding of participants and personnel and outcome assessors (performance bias); D, incomplete outcome data (attrition bias); E, selective reporting (reporting bias); and F, other bias. High other risk of bias was given to crossover trials which had no washout between interventions. Trials which did not have this characteristic were rated as Low.

CI, confidence interval; CNTRL=control; FRC=fructose; Frc17.5= 17.5% fructose; Frc25=25% fructose; HCL=hypercholesterolemic; HFCS10=10% high fructose corn syrup HFCS17.5=17.5% high fructose corn syrup; HFCS25=25% high fructose corn syrup; HF=high fructose; HS=high sucrose; MF=medium fructose; NSB=non-nutritive sweetened beverage; P=prunes; PF=pomegranate juice; SB=symbiotic beverage; SPJ=symbiotic pomegranate juice; SUC=sucrose; T2D=type 2 diabetes

S11 Fig. Forest plot of controlled trials of the effect of important food sources of fructose-containing sugars on DBP (mmHg) in subtraction trials.

Diastolic Blood Pressure

Subtraction Trials

Trial or subgroup	N Control	N Intervention	Mean difference with 95% CI	Weight (%)	A	B	C	D	E	F
**1. SSB**										
Al-Dujaili et al. 2017	16	16	5.10 [0.55 to 9.65]	1.96	U	U	U	L	U	L
Campos et al. 2015 (G1)	6	6	-1.50 [-13.03 to 10.03]	0.32	U	U	U	U	L	L
Campos et al. 2015 (G2)	7	8	-4.70 [-13.48 to 4.08]	0.55	U	U	U	U	L	L
Ebbeling et al. 2020 (ASB)	60	60	-1.50 [-5.23 to 2.23]	2.82	U	U	L	L	L	L
Ebbeling et al. 2020 (water)	60	66	-2.30 [-5.92 to 1.32]	2.98	U	U	L	L	L	L
Hernandez-Cordero et al. 2014	120	120	0.10 [-0.05 to 0.25]	32.20	L	H	H	L	L	L
Tate et al. 2012 (NSB)	75	84	-1.20 [-4.18 to 1.78]	4.22	L	L	L	U	L	L
Tate et al. 2012 (water)	75	87	-1.50 [-4.89 to 1.89]	3.36	L	L	L	U	L	L
Heterogeneity: τ^2 = 0.89, I^2 = 28.79%, H^2 = 1.40			-0.41 [-1.68 to 0.86]							
Test of $\theta_i = \theta_j$: Q(7) = 9.83, p = 0.20										
**2. Mixed sources (with SSBs)**										
Vazquez-Duran et al. 2016 (NSB)	49	49	0.84 [0.24 to 1.44]	25.80	L	U	L	U	L	L
Vazquez-Duran et al. 2016 (water)	49	48	-0.75 [-1.34 to -0.15]	25.80	L	U	L	U	L	L
Heterogeneity: τ^2 = 1.17, I^2 = 92.66%, H^2 = 13.63			0.05 [-1.51 to 1.60]							
Test of $\theta_i = \theta_j$: Q(1) = 13.63, p = 0.00										
**Overall**			-0.09 [-0.74 to 0.57]							
Heterogeneity: τ^2 = 0.34, I^2 = 61.69%, H^2 = 2.61										
Test of $\theta_i = \theta_j$: Q(9) = 23.49, p = 0.01										
Test of group differences: p = 0.637, I^2 = 65.89%, p_Q = 0.003										
Test of θ = 0: z = -0.258, p = 0.796										

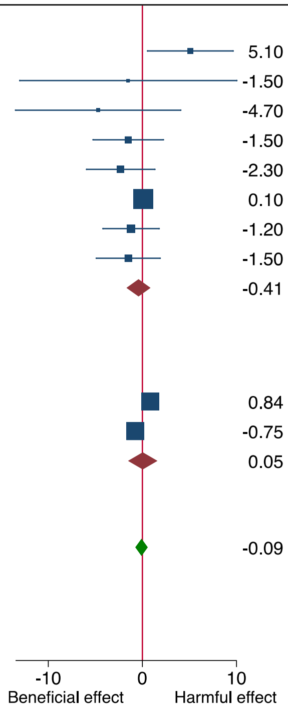

Pooled effect estimates for each subgroup and overall effect are represented by the diamonds. Data are expressed as weighted mean differences with 95% confidence intervals using the generic inverse-variance method and random effects DerSimonian-Laird model. Paired analyses were applied to all crossover trials. Inter-study heterogeneity was assessed using the Cochrane Q statistic and quantified using the I^2 statistic, with significance set at p<0.100 and $I^2 \geq 50\%$ considered to be evidence of substantial heterogeneity. Pooled effect summary estimate was calculated with the χ^2 test. Test for group differences was calculated with meta-regression, which uses the Wald test.

Risk of Bias Legend: (H) High Risk; (L) Low Risk; (U) Unclear. The letters represent the following risk of bias domains: A, random sequence generation (selection bias); B, allocation concealment (selection bias); C, blinding of participants and personnel and outcome assessors (performance bias); D, incomplete outcome data (attrition bias); E, selective reporting (reporting bias); and F, other bias. High other risk of bias was given to crossover trials which had no washout between interventions. Trials which did not have this characteristic were rated as Low.

CI, confidence interval; ASB=artificially sweetened beverage; G1=group 1; G2=group 2; NSB=non-nutritive sweetened beverage

S12 Fig. Forest plot of controlled trials of the effect of important food sources of fructose-containing sugars on DBP (mmHg) in *ad libitum* trials.

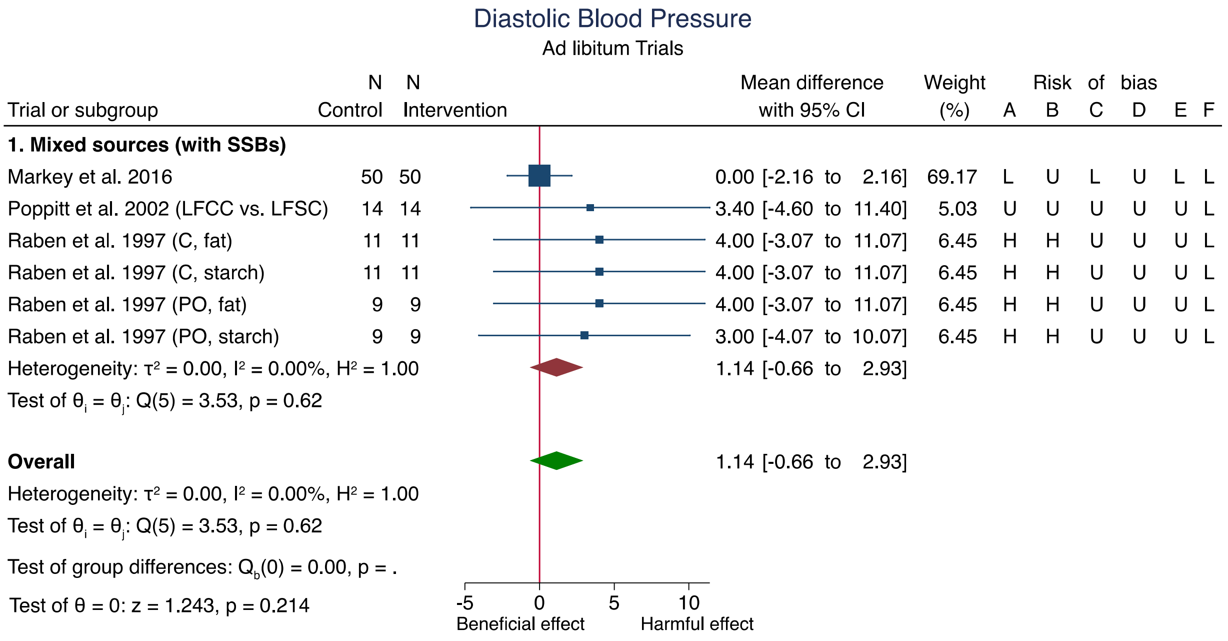

Pooled effect estimates for each subgroup and overall effect are represented by the diamonds. Data are expressed as weighted mean differences with 95% confidence intervals using the generic inverse-variance method and random effects DerSimonian-Laird model. Paired analyses were applied to all crossover trials. Inter-study heterogeneity was assessed using the Cochrane Q statistic and quantified using the I^2 statistic, with significance set at $p<0.100$ and $I^2 \geq 50\%$ considered to be evidence of substantial heterogeneity. Pooled effect summary estimate was calculated with the χ^2 test. Test for group differences was calculated with meta-regression, which uses the Wald test.

Risk of Bias Legend: (H) High Risk; (L) Low Risk; (U) Unclear. The letters represent the following risk of bias domains: A, random sequence generation (selection bias); B, allocation concealment (selection bias); C, blinding of participants and personnel and outcome assessors (performance bias); D, incomplete outcome data (attrition bias); E, selective reporting (reporting bias); and F, other bias. High other risk of bias was given to crossover trials which had no washout between interventions. Trials which did not have this characteristic were rated as Low.

CI, confidence interval; C=control; LFCC=low fat complex carbohydrates; LFSC=low fat simple carbohydrates; PO=post obese

S13 Fig. Sensitivity analysis of the systematic removal of each trial for the effect of important food sources of fructose-containing sugars on SBP (mmHg) in substitution trials.

Influence analysis

Systolic blood pressure (mmHg) in substitution trials

**Trial removed**	Mean difference with 95% CI	P_{effect}	I^2 (%)	P_Q
**Overall**	-0.33 [-1.16 to 0.50]	0.445	39	< 0.001
Aeberli et al. 2011 (HG vs. HF)	-0.36 [-1.21 to 0.49]	0.406	40	< 0.001
Aeberli et al. 2011 (HG vs. HS)	-0.40 [-1.24 to 0.44]	0.350	39	< 0.001
Aeberli et al. 2011 (MG vs. MF)	-0.31 [-1.17 to 0.55]	0.479	40	< 0.001
Aeberli et al. 2013 (HG vs HF)	-0.31 [-1.15 to 0.53]	0.471	40	< 0.001
Aeberli et al. 2013 (HG vs HS)	-0.32 [-1.16 to 0.52]	0.463	40	< 0.001
Agebratt et al. 2016	-0.24 [-1.08 to 0.60]	0.568	38	< 0.001
Amani et al. 2014	-0.28 [-1.12 to 0.56]	0.503	39	< 0.001
Anderson et al. 2014	-0.24 [-1.08 to 0.60]	0.565	38	< 0.001
Angelopoulos et al. 2015 (FRC)	-0.38 [-1.23 to 0.47]	0.382	40	< 0.001
Angelopoulos et al. 2015 (HFCS)	-0.40 [-1.24 to 0.44]	0.354	39	< 0.001
Angelopoulos et al. 2015 (SUC)	-0.35 [-1.20 to 0.50]	0.425	40	< 0.001
Bays et al. 2015	-0.25 [-1.08 to 0.58]	0.553	38	< 0.001
Beisner et al. 2020 (OB, HFS)	-0.31 [-1.15 to 0.53]	0.470	40	< 0.001
Beisner et al. 2020 (OB, fruit)	-0.35 [-1.20 to 0.50]	0.424	40	< 0.001
Beisner et al. 2020 (lean, HFS)	-0.33 [-1.17 to 0.51]	0.444	40	< 0.001
Beisner et al. 2020 (lean, fruit)	-0.35 [-1.20 to 0.50]	0.412	40	< 0.001
Black et al. 2006	-0.29 [-1.13 to 0.55]	0.502	40	< 0.001
Brymora et al. 2012 (basal diet)	-0.38 [-1.22 to 0.46]	0.376	39	< 0.001
Brymora et al. 2012 (resumed diet)	-0.34 [-1.19 to 0.51]	0.425	40	< 0.001
Chiu et al., 2020	-0.41 [-1.26 to 0.44]	0.346	38	< 0.001
Claesson et al. 2009	-0.31 [-1.16 to 0.53]	0.479	40	< 0.001
Cooper et al. 1988	-0.32 [-1.16 to 0.52]	0.463	40	< 0.001
Despland et al. 2017 (honey)	-0.33 [-1.17 to 0.51]	0.447	40	< 0.001
Despland et al. 2017 (sugars)	-0.35 [-1.20 to 0.50]	0.420	40	< 0.001
Dikariyanto et a. 2020	-0.31 [-1.16 to 0.54]	0.475	40	< 0.001
Dominiguez-Coello et al. 2020	-0.37 [-1.22 to 0.48]	0.396	40	< 0.001
Du et al. 2019	-0.29 [-1.12 to 0.55]	0.492	39	< 0.001
Engel et al. 2017	-0.38 [-1.22 to 0.46]	0.374	39	< 0.001
Geidl-Flueck et al. 2021 (GLC vs FRC)	-0.32 [-1.16 to 0.52]	0.459	40	< 0.001
Geidl-Flueck et al. 2021 (GLC vs SUC)	-0.30 [-1.15 to 0.54]	0.489	40	< 0.001
Hallfrisch et al. 1983 (C, 15%)	-0.32 [-1.16 to 0.52]	0.453	40	< 0.001
Hallfrisch et al. 1983 (C, 7.5%)	-0.32 [-1.16 to 0.52]	0.453	40	< 0.001
Hallfrisch et al. 1983 (HI, 15%)	-0.29 [-1.13 to 0.56]	0.507	40	< 0.001
Hallfrisch et al. 1983 (HI, 7.5%)	-0.27 [-1.11 to 0.57]	0.529	39	< 0.001
Hegde et al. 2013	-0.29 [-1.13 to 0.55]	0.503	40	< 0.001
Hieronimus et al. 2020 (Frc25 vs. Glc 25)	-0.25 [-1.09 to 0.59]	0.561	39	< 0.001
Hieronimus et al. 2020 (HFCS25 vs. Glc25)	-0.31 [-1.16 to 0.54]	0.476	40	< 0.001
Israel et al. 1983 (18%)	-0.36 [-1.21 to 0.48]	0.397	40	< 0.001
Israel et al. 1983 (33%)	-0.35 [-1.19 to 0.49]	0.417	40	< 0.001
Jalilvand et al. 2020	-0.37 [-1.23 to 0.49]	0.401	40	< 0.001
Jones et al. 2014	-0.40 [-1.24 to 0.44]	0.343	39	< 0.001
Kaliora et al. 2016	-0.39 [-1.23 to 0.45]	0.367	39	< 0.001
Kanellos et al. 2014	-0.29 [-1.13 to 0.55]	0.495	40	< 0.001
Kanellos et al. 2017	-0.31 [-1.16 to 0.53]	0.475	40	< 0.001
Koh et al. 1988 (IGTS)	-0.24 [-1.08 to 0.60]	0.573	38	< 0.001
Koh et al. 1988 (NGTS)	-0.32 [-1.16 to 0.52]	0.455	40	< 0.001
Koivisto & Yki-Jävinen 1993	-0.35 [-1.19 to 0.49]	0.417	40	< 0.001
Lehtonen et al. 2010	-0.31 [-1.16 to 0.53]	0.479	40	< 0.001
Lehtonen et al. 2011 (bilberry)	-0.33 [-1.19 to 0.53]	0.450	40	< 0.001
Lehtonen et al. 2011 (seaberry)	-0.32 [-1.18 to 0.54]	0.466	40	< 0.001
Lewis et al. 2013	-0.41 [-1.24 to 0.42]	0.333	38	< 0.001
Luukkonen et al. 2018 (SF)	-0.32 [-1.16 to 0.52]	0.453	40	< 0.001
Luukkonen et al. 2018 (UnSF)	-0.31 [-1.15 to 0.53]	0.463	40	< 0.001
Madero et al. 2011	-0.40 [-1.26 to 0.45]	0.363	38	< 0.001
Madero et al. 2015	-0.33 [-1.18 to 0.52]	0.443	40	< 0.001
Maki et al. 2020	-0.39 [-1.23 to 0.45]	0.367	39	< 0.001
Maleki et al. 2019	-0.28 [-1.13 to 0.57]	0.523	40	< 0.001
Navaei et al. 2019	-0.29 [-1.14 to 0.56]	0.511	40	< 0.001
Nier et al. 2018	-0.34 [-1.18 to 0.50]	0.425	40	< 0.001
Palacios et al. 2020	-0.38 [-1.23 to 0.47]	0.386	40	< 0.001
Puglisi et al. 2008 (run-in vs. R alone)	-0.31 [-1.15 to 0.53]	0.474	40	< 0.001
Puglisi et al. 2008 (walk vs. R+walk)	-0.32 [-1.16 to 0.52]	0.458	40	< 0.001
Puupponen-Pimia et al. 2013	-0.35 [-1.19 to 0.49]	0.419	40	< 0.001
Schwimmer et al. 2019	-0.39 [-1.22 to 0.45]	0.356	39	< 0.001
Sigala et al. 2020	-0.33 [-1.18 to 0.52]	0.448	40	< 0.001
Silbernagel et al. 2011	-0.29 [-1.13 to 0.55]	0.502	40	< 0.001
Singh et al. 1997	-0.03 [-0.76 to 0.70]	0.929	21	0.062
Stanhope et al. 2009	-0.35 [-1.20 to 0.50]	0.418	40	< 0.001
Surwit et al. 1997	-0.25 [-1.08 to 0.58]	0.548	38	< 0.001
Swarbrick et al. 2008	-0.33 [-1.17 to 0.51]	0.442	40	< 0.001
Vos et al. 2009	-0.39 [-1.20 to 0.42]	0.339	35	0.002
van Meijl et al. 2011	-0.37 [-1.22 to 0.48]	0.392	40	< 0.001

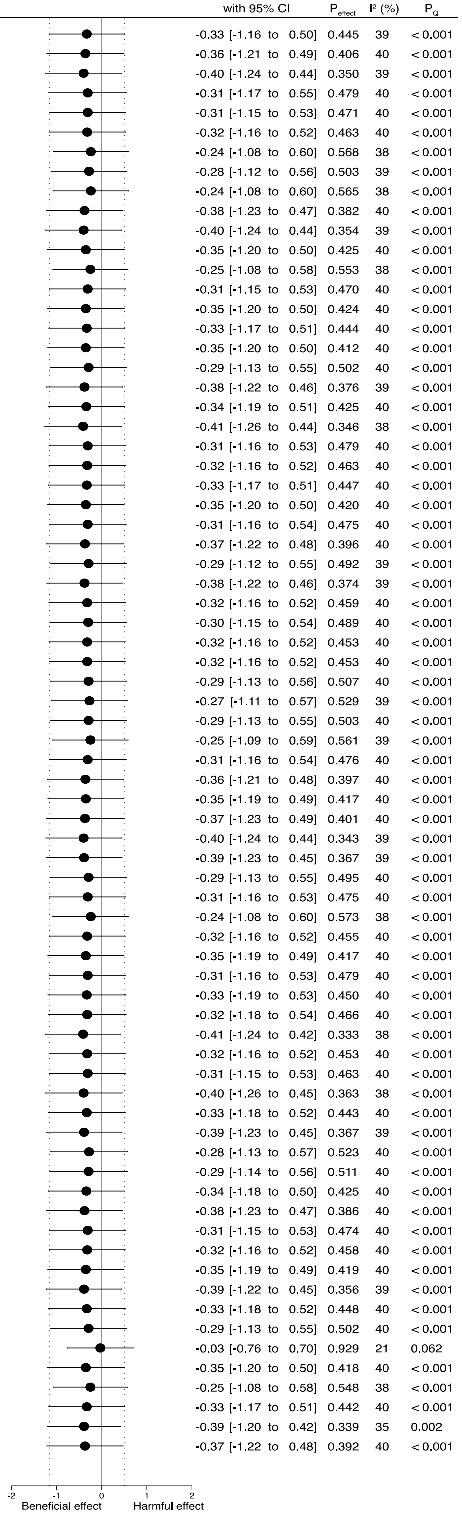

Influence analysis: removal of each trial, one at a time and recalculation of the overall effect and heterogeneity

CI=confidence interval; C=control; FRC=fructose; Frc25= 25% fructose; GLC=glucose; Glc25= 25% glucose; HF=high fructose; HFCS25= 25% high fructose corn syrup; HFS=high fructose syrup; HG=high glucose; HI=hyperinsulinemic; HS=high sucrose; IGTS=impaired glucose-tolerant subjects; MF=medium fructose; MG=medium glucose; NGTS=normal glucose tolerant subjects; OB=obese; R=raisin; SF=saturated fat; SUC=sucrose; UnSF=unsaturated fat

S14 Fig. Sensitivity analysis of the systematic removal of each trial for the effect of important food sources of fructose-containing sugars on SBP (mmHg) in addition trials.

Influence analysis

Systolic blood pressure (mmHg) in addition trials

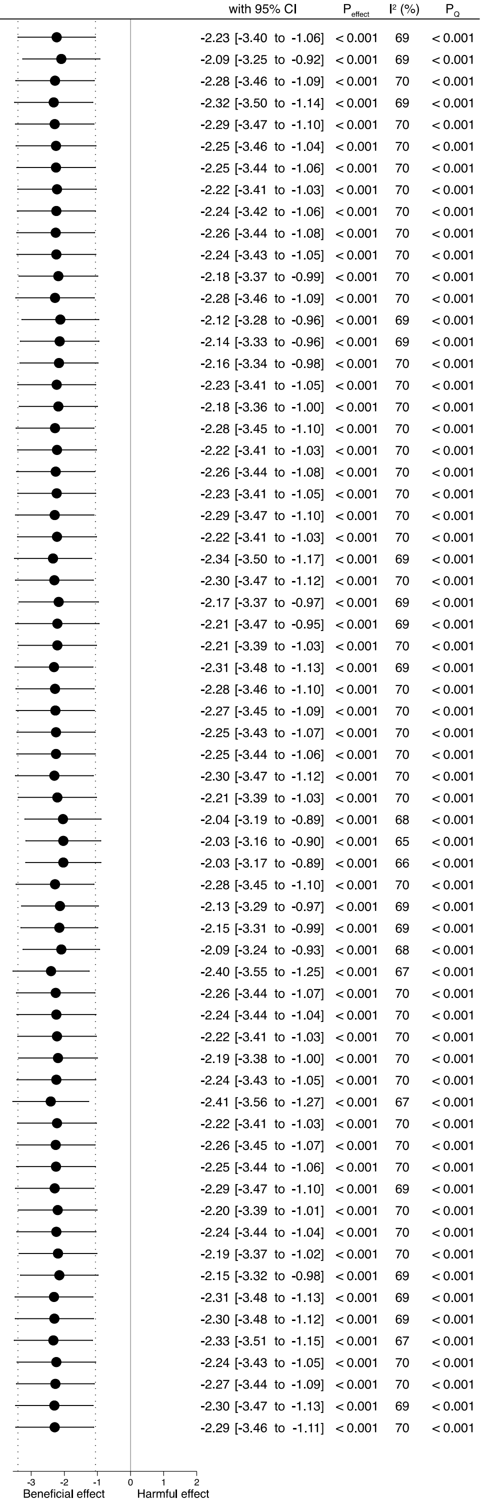

Trial removed	Mean difference with 95% CI	P_{effect}	I^2 (%)	P_Q
**Overall**	-2.23 [-3.40 to -1.06]	< 0.001	69	< 0.001
Abedini et al. 2020	-2.09 [-3.25 to -0.92]	< 0.001	69	< 0.001
Aeberli et al. 2011 (control vs. HF)	-2.28 [-3.46 to -1.09]	< 0.001	70	< 0.001
Aeberli et al. 2011 (control vs. HS)	-2.32 [-3.50 to -1.14]	< 0.001	69	< 0.001
Aeberli et al. 2011 (control vs. MF)	-2.29 [-3.47 to -1.10]	< 0.001	70	< 0.001
Aghababaee et al. 2015	-2.25 [-3.46 to -1.04]	< 0.001	70	< 0.001
Ahmed et al. 2010	-2.25 [-3.44 to -1.06]	< 0.001	70	< 0.001
Al-Dashti et al. 2019 (run-in vs. 2P)	-2.22 [-3.41 to -1.03]	< 0.001	70	< 0.001
Al-Dashti et al. 2019 (run-in vs. 6P)	-2.24 [-3.42 to -1.06]	< 0.001	70	< 0.001
Al-Dashti et al. 2019 (washout vs. 2P)	-2.26 [-3.44 to -1.08]	< 0.001	70	< 0.001
Al-Dashti et al. 2019 (washout vs. 6P)	-2.24 [-3.43 to -1.05]	< 0.001	70	< 0.001
Alatas et al. 2019	-2.18 [-3.37 to -0.99]	< 0.001	70	< 0.001
Amagase et al. 2009	-2.28 [-3.46 to -1.09]	< 0.001	70	< 0.001
Amani et al. 2014	-2.12 [-3.28 to -0.96]	< 0.001	69	< 0.001
Asgary et al. 2014	-2.14 [-3.33 to -0.96]	< 0.001	69	< 0.001
Ayoobi et al. 2017	-2.16 [-3.34 to -0.98]	< 0.001	70	< 0.001
Banini et al. 2006	-2.23 [-3.41 to -1.05]	< 0.001	70	< 0.001
Basu et al. 2010 (blueberry)	-2.18 [-3.36 to -1.00]	< 0.001	70	< 0.001
Basu et al. 2010 (strawberry)	-2.28 [-3.45 to -1.10]	< 0.001	70	< 0.001
Cressey et al. 2014 (HCL, 250g)	-2.22 [-3.41 to -1.03]	< 0.001	70	< 0.001
Cressey et al. 2014 (HCL, 500g)	-2.26 [-3.44 to -1.08]	< 0.001	70	< 0.001
Cressey et al. 2014 (T2D, 250g)	-2.23 [-3.41 to -1.05]	< 0.001	70	< 0.001
Davidi et al. 2011	-2.29 [-3.47 to -1.10]	< 0.001	70	< 0.001
Dow et al. 2012	-2.22 [-3.41 to -1.03]	< 0.001	70	< 0.001
Engel et al. 2017 (NSB)	-2.34 [-3.50 to -1.17]	< 0.001	69	< 0.001
Engel et al. 2017 (water)	-2.30 [-3.47 to -1.12]	< 0.001	70	< 0.001
Esmaeilinezhad et al. 2020 (PJ vs. placebo)	-2.17 [-3.37 to -0.97]	< 0.001	69	< 0.001
Esmaeilinezhad et al. 2020 (SPJ vs. SB)	-2.21 [-3.47 to -0.95]	< 0.001	69	< 0.001
Franck et al. 2020	-2.21 [-3.39 to -1.03]	< 0.001	70	< 0.001
Geidl-Flueck et al. 2021 (CNTRL vs FRC)	-2.31 [-3.48 to -1.13]	< 0.001	69	< 0.001
Geidl-Flueck et al. 2021 (CNTRL vs SUC)	-2.28 [-3.46 to -1.10]	< 0.001	70	< 0.001
Hieronimus et al. 2020 (Frc17.5)	-2.27 [-3.45 to -1.09]	< 0.001	70	< 0.001
Hieronimus et al. 2020 (Frc25)	-2.25 [-3.43 to -1.07]	< 0.001	70	< 0.001
Hieronimus et al. 2020 (HFCS17.5)	-2.25 [-3.44 to -1.06]	< 0.001	70	< 0.001
Hieronimus et al. 2020 (HFCS25)	-2.30 [-3.47 to -1.12]	< 0.001	70	< 0.001
Irannejad et al. 2020	-2.21 [-3.39 to -1.03]	< 0.001	70	< 0.001
Karlsen et al. 2013 (hypertensives)	-2.04 [-3.19 to -0.89]	< 0.001	68	< 0.001
Karlsen et al. 2013 (normal/high-normal BP)	-2.03 [-3.16 to -0.90]	< 0.001	65	< 0.001
Karlsen et al. 2013 (optimal BP)	-2.03 [-3.17 to -0.89]	< 0.001	66	< 0.001
Kojadinovic et al. 2017	-2.28 [-3.45 to -1.10]	< 0.001	70	< 0.001
Kumari et al. 2016 (with peel)	-2.13 [-3.29 to -0.97]	< 0.001	69	< 0.001
Kumari et al. 2016 (without peel)	-2.15 [-3.31 to -0.99]	< 0.001	69	< 0.001
Lazavi et al. 2018	-2.09 [-3.24 to -0.93]	< 0.001	68	< 0.001
Leskinen et al. 2014	-2.40 [-3.55 to -1.25]	< 0.001	67	< 0.001
Martini et al. 2021	-2.26 [-3.44 to -1.07]	< 0.001	70	< 0.001
Mietus-Snyder et al. 2012	-2.24 [-3.44 to -1.04]	< 0.001	70	< 0.001
Neto et al. 2017	-2.22 [-3.41 to -1.03]	< 0.001	70	< 0.001
Njike et al. 2011 (placebo)	-2.19 [-3.38 to -1.00]	< 0.001	70	< 0.001
Njike et al. 2011 (sugar-sweetened)	-2.24 [-3.43 to -1.05]	< 0.001	70	< 0.001
Raben et al. 2002	-2.41 [-3.56 to -1.27]	< 0.001	67	< 0.001
Ravn-Haren et al. 2013 (control vs. clear juice)	-2.22 [-3.41 to -1.03]	< 0.001	70	< 0.001
Ravn-Haren et al. 2013 (control vs. cloudy juice)	-2.26 [-3.45 to -1.07]	< 0.001	70	< 0.001
Ravn-Haren et al. 2013 (control vs. whole apple)	-2.25 [-3.44 to -1.06]	< 0.001	70	< 0.001
Ravn-Haren et al. 2013 (pomace vs. clear juice)	-2.29 [-3.47 to -1.10]	< 0.001	69	< 0.001
Ravn-Haren et al. 2013 (pomace vs. cloudy juice)	-2.20 [-3.39 to -1.01]	< 0.001	70	< 0.001
Ravn-Haren et al. 2013 (pomace vs. whole apple)	-2.24 [-3.44 to -1.04]	< 0.001	70	< 0.001
Schell et al. 2019	-2.19 [-3.37 to -1.02]	< 0.001	70	< 0.001
Shema-Didi et al. 2014	-2.15 [-3.32 to -0.98]	< 0.001	69	< 0.001
Sigala et al. 2020	-2.31 [-3.48 to -1.13]	< 0.001	69	< 0.001
Silbernagel et al. 2011	-2.30 [-3.48 to -1.12]	< 0.001	69	< 0.001
Stanhope et al. 2009	-2.33 [-3.51 to -1.15]	< 0.001	67	< 0.001
Stanhope et al. 2015 (HFCS10)	-2.24 [-3.43 to -1.05]	< 0.001	70	< 0.001
Tang et al. 2020 (20 g)	-2.27 [-3.44 to -1.09]	< 0.001	70	< 0.001
Tang et al. 2020 (40 g)	-2.30 [-3.47 to -1.13]	< 0.001	69	< 0.001
Tang et al. 2020 (60 g)	-2.29 [-3.46 to -1.11]	< 0.001	70	< 0.001

Influence analysis: removal of each trial, one at a time and recalculation of the overall effect and heterogeneity

CI=confidence interval; CNTRL=control; FRC=fructose; Frc17.5= 17.5% fructose; Frc25=25% fructose; HCL=hypercholesterolemic; HFCS10=10% high fructose corn syrup HFCS17.5=17.5% high fructose corn syrup; HFCS25=25% high fructose corn syrup; HF=high fructose; HS=high sucrose; MF=medium fructose; NSB=non-nutritive sweetened beverage; P=prunes; PF=pomegranate juice; SB=symbiotic beverage; SPJ=symbiotic pomegranate juice; SUC=sucrose; T2D=type 2 diabetes

S15 Fig. Sensitivity analysis of the systematic removal of each trial for the effect of important food sources of fructose-containing sugars on SBP (mmHg) in subtraction trials.

Influence analysis

Systolic blood pressure (mmHg) in subtraction trials

**Trial removed**	Mean difference with 95% CI	P_{effect}	I^2 (%)	P_Q
**Overall**	-1.16 [-2.90 to 0.58]	0.188	82	< 0.001
Al-Dujaili et al. 2017	-1.97 [-3.53 to -0.41]	0.013	75	< 0.001
Campos et al. 2015 (G1)	-1.11 [-2.86 to 0.64]	0.215	84	< 0.001
Campos et al. 2015 (G2)	-1.15 [-2.92 to 0.62]	0.202	84	< 0.001
Ebbeling et al. 2020 (ASB)	-1.04 [-2.89 to 0.81]	0.268	84	< 0.001
Ebbeling et al. 2020 (water)	-1.28 [-3.11 to 0.55]	0.173	84	< 0.001
Hernandez-Cordero et al. 2014	-1.10 [-3.14 to 0.94]	0.287	84	< 0.001
Tate et al. 2012 (NSB)	-1.11 [-2.94 to 0.72]	0.235	84	< 0.001
Tate et al. 2012 (water)	-1.05 [-2.87 to 0.77]	0.259	84	< 0.001
Vazquez-Duran et al. 2016 (NSB)	-1.21 [-3.45 to 1.02]	0.290	76	< 0.001
Vazquez-Duran et al. 2016 (water)	-0.53 [-1.95 to 0.89]	0.467	43	0.078

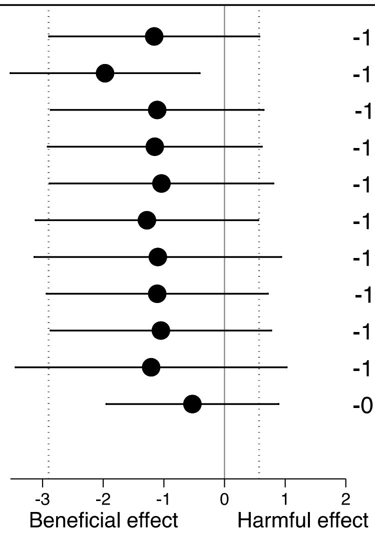

Influence analysis: removal of each trial, one at a time and recalculation of the overall effect and heterogeneity

CI=confidence interval; ASB=artificially sweetened beverage; G1=group 1; G2=group 2; NSB=non-nutritive sweetened beverage

S16 Fig. Sensitivity analysis of the systematic removal of each trial for the effect of important food sources of fructose-containing sugars on SBP (mmHg) in *ad libitum* trials.

Influence analysis

Systolic blood pressure (mmHg) in ad libitum trials

Trial removed	Mean difference with 95% CI	P_{effect}	I^2 (%)	P_Q
**Overall**	0.98 [-0.43 to 2.39]	0.173	0	0.902
Markey et al. 2016	0.73 [-4.34 to 5.80]	0.777	0	0.812
Poppitt et al. 2002 (LFCC vs. LFSC)	0.96 [-0.47 to 2.39]	0.186	0	0.815
Raben et al. 1997 (C, fat)	0.98 [-0.44 to 2.40]	0.178	0	0.810
Raben et al. 1997 (C, starch)	1.08 [-0.34 to 2.50]	0.136	0	0.981
Raben et al. 1997 (PO, fat)	0.96 [-0.46 to 2.38]	0.185	0	0.825
Raben et al. 1997 (PO, starch)	0.94 [-0.47 to 2.35]	0.195	0	0.868

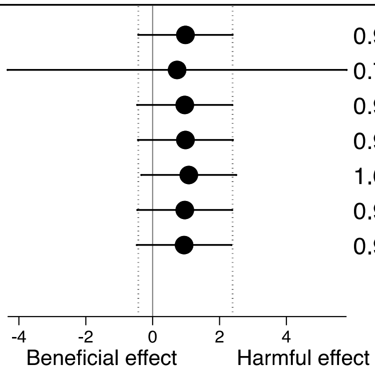

Influence analysis: removal of each trial, one at a time and recalculation of the overall effect and heterogeneity

CI=confidence interval; C=control; LFCC=low fat complex carbohydrates; LFSC=low fat simple carbohydrates; PO=post obese

S17 Fig. Sensitivity analysis of the systematic removal of each trial for the effect of important food sources of fructose-containing sugars on DBP (mmHg) in substitution trials.

Influence analysis

Diastolic blood pressure (mmHg) in substitution trials

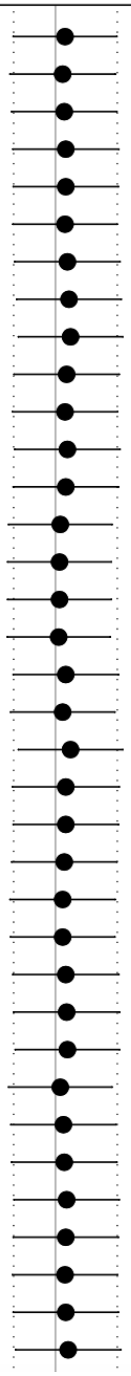

**Trial removed**	Mean difference with 95% CI	P_{effect}	I^2 (%)	P_Q
**Overall**	0.12 [-0.53 to 0.77]	0.714	59	< 0.001
Aeberli et al. 2011 (HG vs. HF)	0.09 [-0.57 to 0.75]	0.800	59	< 0.001
Aeberli et al. 2011 (HG vs. HS)	0.11 [-0.55 to 0.77]	0.755	60	< 0.001
Aeberli et al. 2011 (MG vs. MF)	0.13 [-0.54 to 0.80]	0.701	60	< 0.001
Aeberli et al. 2013 (HG vs HF)	0.13 [-0.54 to 0.80]	0.710	60	< 0.001
Aeberli et al. 2013 (HG vs HS)	0.12 [-0.54 to 0.78]	0.730	60	< 0.001
Agebratt et al. 2016	0.15 [-0.51 to 0.81]	0.661	60	< 0.001
Amani et al. 2014	0.17 [-0.49 to 0.83]	0.618	59	< 0.001
Anderson et al. 2014	0.19 [-0.47 to 0.85]	0.582	59	< 0.001
Angelopoulos et al. 2015 (FRC)	0.14 [-0.52 to 0.80]	0.690	60	< 0.001
Angelopoulos et al. 2015 (HFCS)	0.12 [-0.54 to 0.78]	0.731	60	< 0.001
Angelopoulos et al. 2015 (SUC)	0.15 [-0.51 to 0.81]	0.660	60	< 0.001
Bays et al. 2015	0.13 [-0.54 to 0.80]	0.707	60	< 0.001
Beisner et al. 2020 (OB, HFS)	0.06 [-0.60 to 0.71]	0.865	59	< 0.001
Beisner et al. 2020 (OB, fruit)	0.05 [-0.61 to 0.71]	0.872	59	< 0.001
Beisner et al. 2020 (lean, HFS)	0.05 [-0.61 to 0.71]	0.883	58	< 0.001
Beisner et al. 2020 (lean, fruit)	0.04 [-0.61 to 0.69]	0.907	58	< 0.001
Black et al. 2006	0.13 [-0.54 to 0.80]	0.707	60	< 0.001
Brymora et al. 2012 (basal diet)	0.09 [-0.57 to 0.75]	0.795	59	< 0.001
Brymora et al. 2012 (resumed diet)	0.19 [-0.47 to 0.85]	0.576	59	< 0.001
Chiu et al., 2020	0.13 [-0.55 to 0.81]	0.698	60	< 0.001
Claesson et al. 2009	0.13 [-0.54 to 0.80]	0.709	60	< 0.001
Cooper et al. 1988	0.11 [-0.55 to 0.77]	0.739	60	< 0.001
Despland et al. 2017 (honey)	0.09 [-0.57 to 0.75]	0.779	59	< 0.001
Despland et al. 2017 (sugars)	0.09 [-0.57 to 0.75]	0.789	59	< 0.001
Dikariyanto et a. 2020	0.13 [-0.54 to 0.80]	0.713	60	< 0.001
Dominiguez-Coello et al. 2020	0.14 [-0.53 to 0.81]	0.688	60	< 0.001
Du et al. 2019	0.15 [-0.51 to 0.81]	0.656	59	< 0.001
Engel et al. 2018	0.06 [-0.60 to 0.71]	0.846	59	< 0.001
Geidl-Flueck et al. 2021 (GLC vs FRC)	0.10 [-0.56 to 0.76]	0.756	60	< 0.001
Geidl-Flueck et al. 2021 (GLC vs SUC)	0.11 [-0.55 to 0.77]	0.738	60	< 0.001
Hallfrisch et al. 1983 (C, 15%)	0.14 [-0.52 to 0.80]	0.687	60	< 0.001
Hallfrisch et al. 1983 (C, 7.5%)	0.13 [-0.54 to 0.80]	0.710	60	< 0.001
Hallfrisch et al. 1983 (HI, 15%)	0.12 [-0.54 to 0.78]	0.731	60	< 0.001
Hallfrisch et al. 1983 (HI, 7.5%)	0.13 [-0.54 to 0.80]	0.709	60	< 0.001
Hegde et al. 2013	0.16 [-0.50 to 0.82]	0.648	59	< 0.001

Hegde et al. 2013	0.16 [-0.50 to 0.82]	0.648	59	< 0.001
Hieronimus et al. 2020 (Frc25 vs. Glc 25)	0.14 [-0.53 to 0.81]	0.679	60	< 0.001
Hieronimus et al. 2020 (HFCS25 vs. Glc25)	0.11 [-0.55 to 0.77]	0.738	60	< 0.001
Israel et al. 1983 (18%)	0.12 [-0.54 to 0.78]	0.732	60	< 0.001
Israel et al. 1983 (33%)	0.10 [-0.56 to 0.76]	0.772	59	< 0.001
Jalilvand et al. 2020	0.06 [-0.60 to 0.71]	0.862	59	< 0.001
Jones et al. 2014	0.07 [-0.59 to 0.73]	0.826	59	< 0.001
Kaliora et al. 2016	0.05 [-0.61 to 0.71]	0.879	58	< 0.001
Kanellos et al. 2014	0.18 [-0.47 to 0.83]	0.590	58	< 0.001
Kanellos et al. 2017	0.12 [-0.55 to 0.79]	0.717	60	< 0.001
Koh et al. 1988 (IGTS)	0.19 [-0.47 to 0.85]	0.566	58	< 0.001
Koh et al. 1988 (NGTS)	0.19 [-0.47 to 0.85]	0.566	58	< 0.001
Koivisto & Yki-Jävinen 1993	0.13 [-0.54 to 0.80]	0.710	60	< 0.001
Lehtonen et al. 2010	0.16 [-0.50 to 0.82]	0.641	59	< 0.001
Lehtonen et al. 2011 (bilberry)	0.14 [-0.54 to 0.82]	0.684	60	< 0.001
Lehtonen et al. 2011 (seaberry)	0.19 [-0.48 to 0.86]	0.587	58	< 0.001
Lewis et al. 2013	0.09 [-0.57 to 0.75]	0.797	59	< 0.001
Luukkonen et al. 2018 (SF)	0.16 [-0.50 to 0.82]	0.630	59	< 0.001
Luukkonen et al. 2018 (UnSF)	0.13 [-0.53 to 0.79]	0.693	60	< 0.001
Madero et al. 2011	0.15 [-0.51 to 0.81]	0.668	60	< 0.001
Madero et al. 2015	0.10 [-0.56 to 0.76]	0.779	59	< 0.001
Maki et al. 2020	0.13 [-0.54 to 0.80]	0.703	60	< 0.001
Maleki et al. 2019	0.16 [-0.51 to 0.83]	0.631	59	< 0.001
Navaei et al. 2019	0.11 [-0.56 to 0.78]	0.758	59	< 0.001
Nier et al. 2018	0.09 [-0.56 to 0.75]	0.779	59	< 0.001
Palacios et al. 2020	0.11 [-0.55 to 0.77]	0.741	60	< 0.001
Puglisi et al. 2008 (run-in vs. R alone)	0.13 [-0.53 to 0.79]	0.698	60	< 0.001
Puglisi et al. 2008 (walk vs. R+walk)	0.14 [-0.52 to 0.80]	0.682	60	< 0.001
Puupponen-Pimia et al. 2013	0.12 [-0.55 to 0.79]	0.713	60	< 0.001
Schwimmer et al. 2019	0.10 [-0.56 to 0.76]	0.768	59	< 0.001
Sigala et al. 2020	0.10 [-0.56 to 0.76]	0.761	60	< 0.001
Silbernagel et al. 2011	0.14 [-0.52 to 0.80]	0.679	60	< 0.001
Singh et al. 1997	0.24 [-0.28 to 0.76]	0.382	31	0.008
Stanhope et al. 2009	0.11 [-0.56 to 0.78]	0.741	60	< 0.001
Surwit et al. 1997	0.16 [-0.57 to 0.89]	0.662	58	< 0.001
Swarbrick et al. 2008	0.13 [-0.52 to 0.78]	0.710	60	< 0.001
Vos et al. 2009	0.14 [-0.52 to 0.80]	0.674	59	< 0.001
van Meijl et al. 2011	0.09 [-0.58 to 0.76]	0.793	59	< 0.001

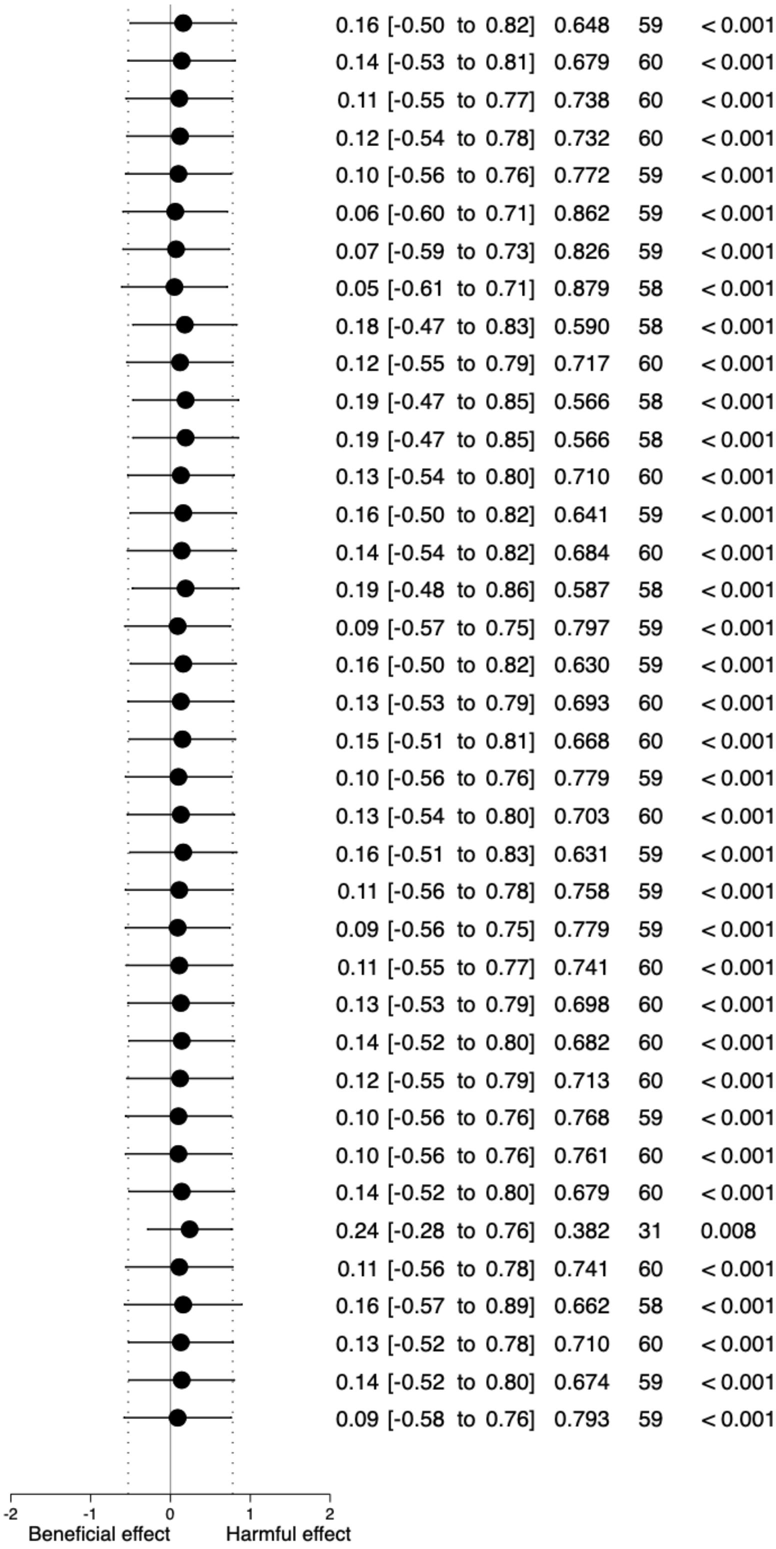

Influence analysis: removal of each trial, one at a time and recalculation of the overall effect and heterogeneity

CI=confidence interval; C=control; FRC=fructose; Frc25= 25% fructose; GLC=glucose; Glc25= 25% glucose; HF=high fructose; HFCS25= 25% high fructose corn syrup; HFS=high fructose syrup; HG=high glucose; HI=hyperinsulinemic; HS=high sucrose; IGTS=impaired glucose-tolerant subjects; MF=medium fructose; MG=medium glucose; NGTS=normal glucose tolerant subjects; OB=obese; R=raisin; SF=saturated fat; SUC=sucrose; UnSF=unsaturated fat

S18 Fig. Sensitivity analysis of the systematic removal of each trial for the effect of important food sources of fructose-containing sugars on DBP (mmHg) in addition trials.

Influence analysis

Diastolic blood pressure (mmHg) in addition trials

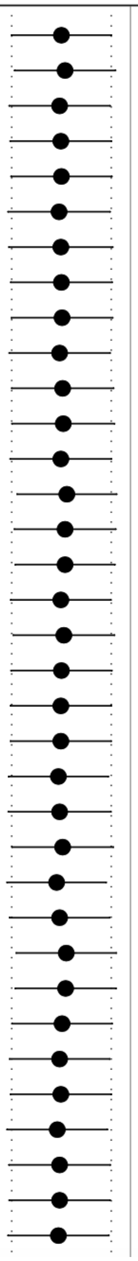

Trial removed	Mean difference with 95% CI	P_{effect}	I^2 (%)	P_Q
**Overall**	-1.15 [-1.98 to -0.32]	0.007	58	< 0.001
Abedini et al. 2020	-1.09 [-1.93 to -0.25]	0.011	58	< 0.001
Aeberli et al. 2011 (control vs. HF)	-1.18 [-2.02 to -0.34]	0.006	59	< 0.001
Aeberli et al. 2011 (control vs. HS)	-1.16 [-2.00 to -0.31]	0.007	59	< 0.001
Aeberli et al. 2011 (control vs. MF)	-1.15 [-1.99 to -0.31]	0.008	59	< 0.001
Aghababaee et al. 2015	-1.19 [-2.04 to -0.34]	0.006	58	< 0.001
Ahmed et al. 2010	-1.16 [-2.02 to -0.29]	0.008	59	< 0.001
Al-Dashti et al. 2019 (run-in vs. 2P)	-1.15 [-1.99 to -0.30]	0.008	59	< 0.001
Al-Dashti et al. 2019 (run-in vs. 6P)	-1.14 [-1.98 to -0.29]	0.008	59	< 0.001
Al-Dashti et al. 2019 (washout vs. 2P)	-1.18 [-2.02 to -0.34]	0.006	59	< 0.001
Al-Dashti et al. 2019 (washout vs. 6P)	-1.13 [-1.98 to -0.28]	0.009	59	< 0.001
Alatas et al. 2019	-1.12 [-1.98 to -0.26]	0.010	59	< 0.001
Amagase et al. 2009	-1.16 [-2.00 to -0.31]	0.007	59	< 0.001
Amani et al. 2014	-1.06 [-1.89 to -0.23]	0.012	57	< 0.001
Asgary et al. 2014	-1.09 [-1.94 to -0.25]	0.011	58	< 0.001
Ayoobi et al. 2017	-1.09 [-1.92 to -0.26]	0.010	58	< 0.001
Banini et al. 2006	-1.16 [-1.99 to -0.32]	0.007	59	< 0.001
Basu et al. 2010a (blueberry)	-1.11 [-1.96 to -0.27]	0.010	59	< 0.001
Basu et al. 2010b (strawberry)	-1.15 [-1.99 to -0.31]	0.007	59	< 0.001
Cressey et al. 2014 (HCL, 250g)	-1.16 [-2.00 to -0.32]	0.007	59	< 0.001
Cressey et al. 2014 (HCL, 500g)	-1.16 [-2.00 to -0.32]	0.007	59	< 0.001
Cressey et al. 2014 (T2D, 250g)	-1.20 [-2.03 to -0.37]	0.005	58	< 0.001
Davidi et al. 2011	-1.18 [-2.03 to -0.32]	0.006	58	< 0.001
Dow et al. 2012	-1.13 [-1.98 to -0.28]	0.009	59	< 0.001
Engel et al. 2017 (NSB)	-1.23 [-2.05 to -0.41]	0.004	58	< 0.001
Engel et al. 2017 (water)	-1.18 [-2.01 to -0.34]	0.006	59	< 0.001
Esmaeilinezhad et al. 2020 (PJ vs. placebo)	-1.07 [-1.91 to -0.24]	0.012	58	< 0.001
Esmaeilinezhad et al. 2020 (SPJ vs. SB)	-1.08 [-1.92 to -0.24]	0.012	58	< 0.001
Franck et al. 2020	-1.14 [-1.97 to -0.30]	0.007	59	< 0.001
Geidl-Flueck et al. 2021 (CNTRL vs FRC)	-1.18 [-2.01 to -0.34]	0.006	59	< 0.001
Geidl-Flueck et al. 2021 (CNTRL vs SUC)	-1.16 [-2.00 to -0.32]	0.007	59	< 0.001
Hieronimus et al. 2020 (Frc17.5)	-1.22 [-2.06 to -0.39]	0.004	58	< 0.001
Hieronimus et al. 2020 (Frc25)	-1.18 [-2.02 to -0.33]	0.006	59	< 0.001
Hieronimus et al. 2020 (HFCS17.5)	-1.18 [-2.02 to -0.34]	0.006	59	< 0.001
Hieronimus et al. 2020 (HFCS25)	-1.20 [-2.04 to -0.36]	0.005	58	< 0.001

Irannejad et al. 2020	-1.18 [-2.02 to -0.34]	0.006	59	< 0.001
Karlsen et al. 2013 (hypertensives)	-1.03 [-1.84 to -0.21]	0.013	55	< 0.001
Karlsen et al. 2013 (normal/high-normal BP)	-1.03 [-1.84 to -0.21]	0.013	55	< 0.001
Karlsen et al. 2013 (optimal BP)	-1.02 [-1.82 to -0.22]	0.013	54	< 0.001
Kojadinovic et al. 2017	-1.24 [-2.06 to -0.42]	0.003	57	< 0.001
Kumari et al. 2016 (with peel)	-1.08 [-1.91 to -0.26]	0.010	57	< 0.001
Kumari et al. 2016 (without peel)	-1.08 [-1.90 to -0.26]	0.010	58	< 0.001
Lazavi et al. 2018	-1.08 [-1.91 to -0.25]	0.011	58	< 0.001
Leskinen et al. 2014	-1.33 [-2.13 to -0.53]	0.001	53	< 0.001
Martini et al. 2021	-1.13 [-1.98 to -0.28]	0.009	59	< 0.001
Mietus-Snyder et al. 2012	-1.12 [-1.97 to -0.27]	0.010	59	< 0.001
Neto et al. 2017	-1.17 [-2.01 to -0.33]	0.006	59	< 0.001
Njike et al. 2011 (placebo)	-1.20 [-2.04 to -0.36]	0.005	58	< 0.001
Njike et al. 2011 (sugar-sweetened)	-1.17 [-2.02 to -0.32]	0.007	59	< 0.001
Raben et al. 2002	-1.28 [-2.10 to -0.46]	0.002	56	< 0.001
Ravn-Haren et al. 2013 (control vs. clear juice)	-1.12 [-1.97 to -0.27]	0.010	59	< 0.001
Ravn-Haren et al. 2013 (control vs. cloudy juice)	-1.15 [-1.99 to -0.30]	0.007	59	< 0.001
Ravn-Haren et al. 2013 (control vs. whole apple)	-1.16 [-2.00 to -0.31]	0.007	59	< 0.001
Ravn-Haren et al. 2013 (pomace vs. clear juice)	-1.19 [-2.03 to -0.35]	0.006	59	< 0.001
Ravn-Haren et al. 2013 (pomace vs. cloudy juice)	-1.11 [-1.96 to -0.27]	0.010	59	< 0.001
Ravn-Haren et al. 2013 (pomace vs. whole apple)	-1.15 [-1.99 to -0.30]	0.008	59	< 0.001
Schell et al. 2019	-1.13 [-1.97 to -0.29]	0.008	59	< 0.001
Shema-Didi et al. 2014	-1.12 [-1.96 to -0.28]	0.009	59	< 0.001
Sigala et al. 2020	-1.21 [-2.05 to -0.38]	0.005	58	< 0.001
Silbernagel et al. 2011	-1.15 [-1.99 to -0.31]	0.007	59	< 0.001
Stanhope et al. 2009	-1.15 [-2.02 to -0.27]	0.010	59	< 0.001
Stanhope et al. 2015 (HFCS10)	-1.16 [-2.00 to -0.31]	0.007	59	< 0.001
Tang et al. 2020 (20 g)	-1.17 [-2.01 to -0.33]	0.006	59	< 0.001
Tang et al. 2020 (40 g)	-1.18 [-2.01 to -0.34]	0.005	59	< 0.001
Tang et al. 2020 (60 g)	-1.16 [-1.99 to -0.32]	0.006	59	< 0.001

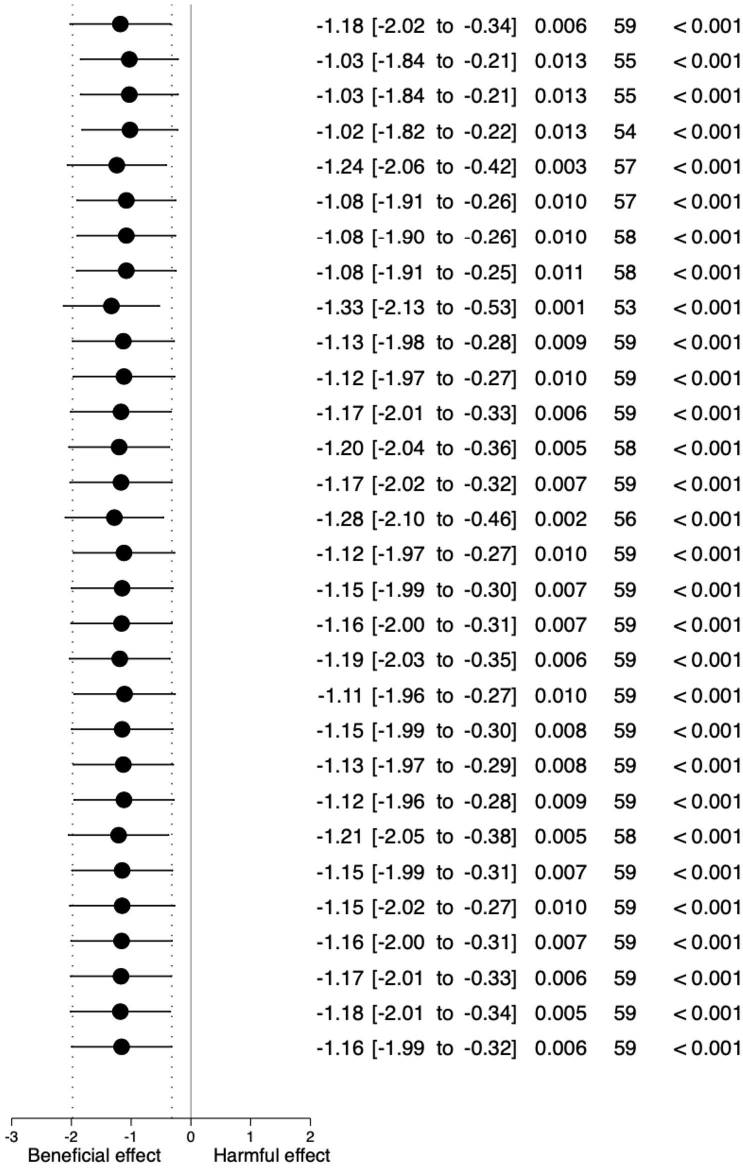

Influence analysis: removal of each trial, one at a time and recalculation of the overall effect and heterogeneity

CI=confidence interval; CNTRL=control; FRC=fructose; Frc17.5= 17.5% fructose; Frc25=25% fructose; HCL=hypercholesterolemic; HFCS10=10% high fructose corn syrup HFCS17.5=17.5% high fructose corn syrup; HFCS25=25% high fructose corn syrup; HF=high fructose; HS=high sucrose; MF=medium fructose; NSB=non-nutritive sweetened beverage; P=prunes; PF=pomegranate juice; SB=symbiotic beverage; SPJ=symbiotic pomegranate juice; SUC=sucrose; T2D=type 2 diabetes

S19 Fig. Sensitivity analysis of the systematic removal of each trial for the effect of important food sources of fructose-containing sugars on DBP (mmHg) in subtraction trials.

Influence analysis
Diastolic blood pressure (mmHg) in subtraction trials

Trial removed	Mean difference with 95% CI	P_{effect}	I^2 (%)	P_Q
**Overall**	-0.09 [-0.75 to 0.56]	0.796	62	0.005
Al-Dujaili et al. 2017	-0.14 [-0.73 to 0.45]	0.634	57	0.016
Campos et al. 2015 (G1)	-0.09 [-0.76 to 0.58]	0.798	66	0.003
Campos et al. 2015 (G2)	-0.06 [-0.72 to 0.60]	0.857	64	0.004
Ebbeling et al. 2020 (ASB)	-0.05 [-0.73 to 0.63]	0.890	65	0.004
Ebbeling et al. 2020 (water)	-0.02 [-0.68 to 0.64]	0.961	63	0.005
Hernandez-Cordero et al. 2014	-0.33 [-1.50 to 0.84]	0.577	66	0.003
Tate et al. 2012 (NSB)	-0.04 [-0.72 to 0.64]	0.908	65	0.004
Tate et al. 2012 (water)	-0.04 [-0.71 to 0.63]	0.910	65	0.004
Vazquez-Duran et al. 2016 (NSB)	-0.42 [-1.22 to 0.38]	0.298	53	0.031
Vazquez-Duran et al. 2016 (water)	0.15 [-0.60 to 0.90]	0.691	49	0.049

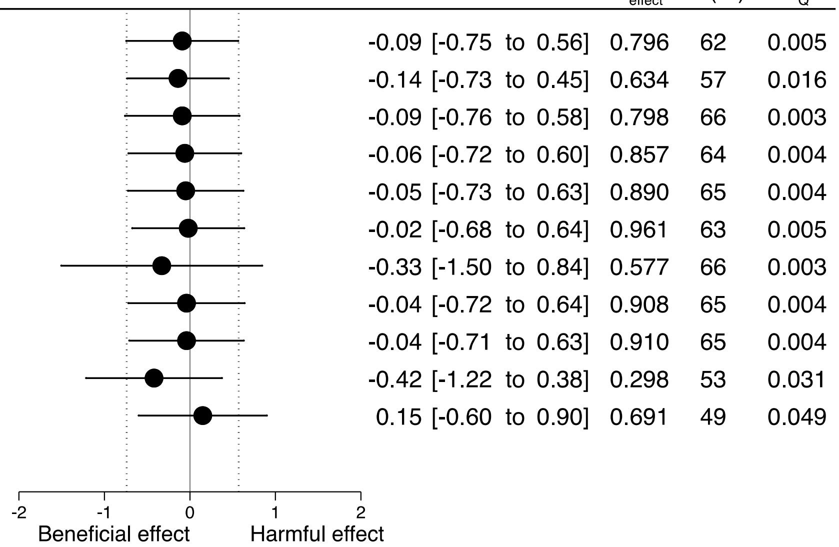

Influence analysis: removal of each trial, one at a time and recalculation of the overall effect and heterogeneity

CI=confidence interval; ASB=artificially sweetened beverage; G1=group 1; G2=group 2; NSB=non-nutritive sweetened beverage

S20 Fig. Sensitivity analysis of the systematic removal of each trial for the effect of important food sources of fructose-containing sugars on DBP (mmHg) in *ad libitum* trials.

Influence analysis

Diastolic blood pressure (mmHg) in ad libitum trials

Trial removed	Mean difference with 95% CI	P_{effect}	I^2 (%)	P_Q
**Overall**	1.14 [-0.66 to 2.94]	0.214	0	0.618
Markey et al. 2016	3.69 [0.46 to 6.92]	0.025	0	1.000
Poppitt et al. 2002 (LFCC vs. LFSC)	1.02 [-0.82 to 2.86]	0.278	0	0.523
Raben et al. 1997 (C, fat)	0.94 [-0.91 to 2.79]	0.320	0	0.582
Raben et al. 1997 (C, starch)	0.94 [-0.91 to 2.79]	0.320	0	0.582
Raben et al. 1997 (PO, fat)	0.94 [-0.91 to 2.79]	0.320	0	0.582
Raben et al. 1997 (PO, starch)	1.01 [-0.85 to 2.87]	0.286	0	0.517

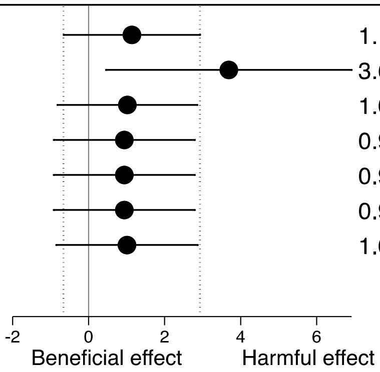

Influence analysis: removal of each trial, one at a time and recalculation of the overall effect and heterogeneity

CI=confidence interval; C=control; LFCC=low fat complex carbohydrates; LFSC=low fat simple carbohydrates; PO=post obese

S21 Fig. Sensitivity analysis of the systematic removal of each trial for the effect of SSBs on SBP (mmHg) in substitution trials.

Influence analysis

Systolic blood pressure (mmHg) in substitution SSB trials

Trial removed	Mean difference with 95% CI	P_{effect}	I^2 (%)	P_Q
**Overall**	0.39 [-0.86 to 1.63]	0.538	4	0.407
Aeberli et al. 2011 (HG vs. HF)	0.20 [-1.20 to 1.60]	0.783	10	0.346
Aeberli et al. 2011 (HG vs. HS)	0.25 [-0.98 to 1.48]	0.695	0	0.466
Aeberli et al. 2011 (MG vs. MF)	0.61 [-0.75 to 1.97]	0.378	4	0.405
Aeberli et al. 2013 (HG vs HF)	0.39 [-0.92 to 1.70]	0.559	8	0.367
Aeberli et al. 2013 (HG vs HS)	0.35 [-0.97 to 1.67]	0.601	9	0.353
Chiu et al., 2020	-0.32 [-1.78 to 1.14]	0.667	0	0.585
Engel et al. 2017	0.36 [-0.83 to 1.55]	0.549	0	0.461
Geidl-Flueck et al. 2021 (GLC vs FRC)	0.35 [-1.00 to 1.70]	0.612	9	0.348
Geidl-Flueck et al. 2021 (GLC vs SUC)	0.49 [-0.78 to 1.76]	0.446	4	0.402
Hieronimus et al. 2020 (Frc25 vs. Glc 25)	0.81 [-0.40 to 2.02]	0.193	0	0.677
Hieronimus et al. 2020 (HFCS25 vs. Glc25)	0.45 [-0.89 to 1.79]	0.507	7	0.374
Koivisto & Yki-Jävinen 1993	0.29 [-1.01 to 1.59]	0.667	7	0.369
Sigala et al. 2020	0.32 [-1.06 to 1.69]	0.650	10	0.341
Silbernagel et al. 2011	0.60 [-0.59 to 1.79]	0.327	0	0.455
Stanhope et al. 2009	0.24 [-1.14 to 1.61]	0.735	10	0.341
Swarbrick et al. 2008	0.29 [-1.04 to 1.62]	0.672	10	0.339

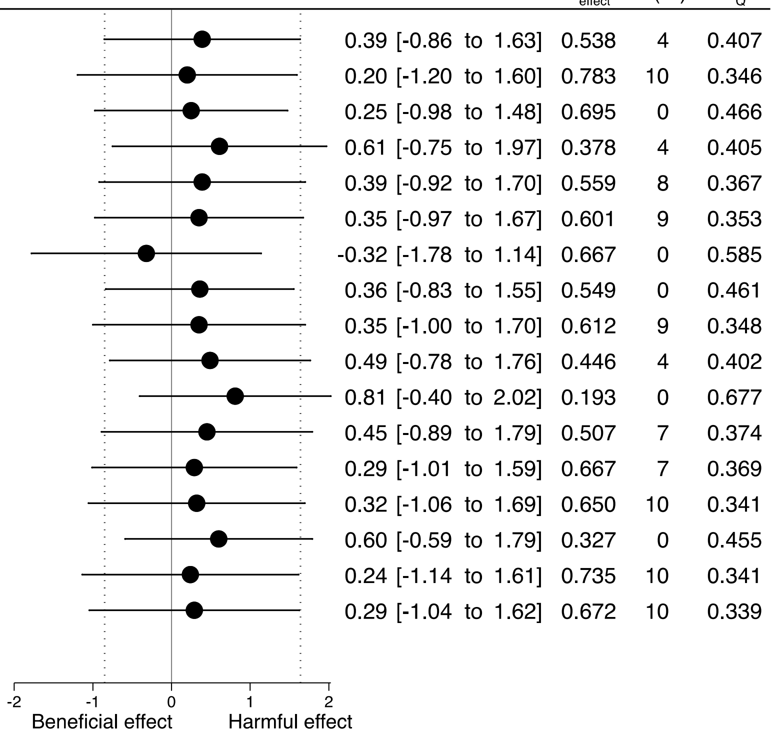

Influence analysis: removal of each trial, one at a time and recalculation of the overall effect and heterogeneity

CI=confidence interval; FRC=fructose; GLC=glucose; HF=high fructose; HFCS=high fructose corn syrup; HG=high glucose; HS=high sucrose; MF=medium fructose; MG=medium glucose; SUC=sucrose

S22 Fig. Sensitivity analysis of the systematic removal of each trial for the effect of sweetened dairy on SBP (mmHg) in substitution trials.

Influence analysis

Systolic blood pressure (mmHg) in substitution sweetened dairy trials

Trial removed	Mean difference with 95% CI	P_{effect}	I^2 (%)	P_Q
**Overall**	2.18 [-0.49 to 4.85]	0.110	0	0.694
Angelopoulos et al. 2015 (FRC)	2.09 [-1.20 to 5.37]	0.213	0	0.395
Angelopoulos et al. 2015 (HFCS)	1.52 [-1.73 to 4.77]	0.359	0	0.615
Angelopoulos et al. 2015 (SUC)	2.93 [-0.34 to 6.20]	0.079	0	0.724

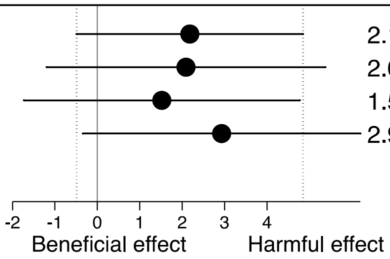

Influence analysis: removal of each trial, one at a time and recalculation of the overall effect and heterogeneity

CI=confidence interval; FRC=fructose; HFCS=high fructose corn syrup; SUC=sucrose

S23 Fig. Sensitivity analysis of the systematic removal of each trial for the effect of fruit on SBP (mmHg) in substitution trials.

Influence analysis

Systolic blood pressure (mmHg) in substitution fruit trials

Trial removed	Mean difference with 95% CI	P_{effect}	I^2 (%)	P_Q
**Overall**	-2.13 [-4.79 to 0.53]	0.116	74	< 0.001
Agebratt et al. 2016	-1.83 [-4.66 to 1.00]	0.207	75	< 0.001
Amani et al. 2014	-1.83 [-4.51 to 0.84]	0.180	75	< 0.001
Beisner et al. 2020 (OB, fruit)	-2.46 [-5.34 to 0.42]	0.093	76	< 0.001
Beisner et al. 2020 (lean, fruit)	-2.50 [-5.34 to 0.34]	0.084	76	< 0.001
Du et al. 2019	-1.91 [-4.62 to 0.80]	0.168	76	< 0.001
Hegde et al. 2013	-2.02 [-4.85 to 0.81]	0.162	76	< 0.001
Lehtonen et al. 2011 (bilberry)	-2.48 [-5.66 to 0.70]	0.126	76	< 0.001
Madero et al. 2011	-2.75 [-5.48 to -0.02]	0.047	63	0.004
Navaei et al. 2019	-2.19 [-5.22 to 0.83]	0.157	76	< 0.001
Puupponen-Pimia et al. 2013	-2.40 [-5.15 to 0.35]	0.087	76	< 0.001
Singh et al. 1997	-0.76 [-2.64 to 1.12]	0.425	37	0.109

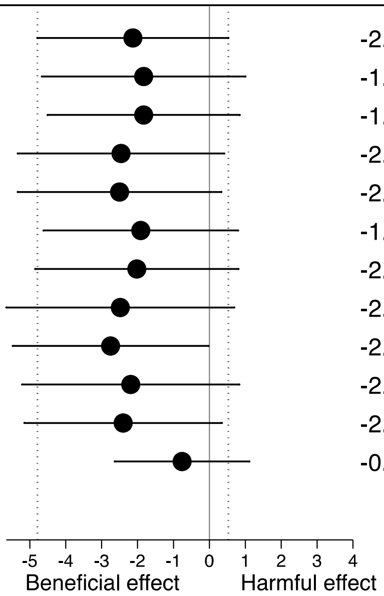

Influence analysis: removal of each trial, one at a time and recalculation of the overall effect and heterogeneity

CI=confidence interval; OB=obese

S24 Fig. Sensitivity analysis of the systematic removal of each trial for the effect of dried fruit on SBP (mmHg) in substitution trials.

Influence analysis

Systolic blood pressure (mmHg) in substitution dried fruit trials

Trial removed	Mean difference with 95% CI	P_{effect}	I^2 (%)	P_Q
**Overall**	-2.22 [-4.99 to 0.55]	0.116	33	0.165
Anderson et al. 2014	-1.46 [-4.22 to 1.30]	0.301	24	0.246
Bays et al. 2015	-1.40 [-3.87 to 1.07]	0.269	17	0.301
Kaliora et al. 2016	-2.59 [-4.85 to -0.32]	0.025	6	0.381
Kanellos et al. 2014	-1.96 [-4.85 to 0.93]	0.184	37	0.148
Kanellos et al. 2017	-2.44 [-5.71 to 0.83]	0.144	43	0.107
Lehtonen et al. 2011 (seaberry)	-2.95 [-6.51 to 0.61]	0.104	36	0.157
Puglisi et al. 2008 (run-in vs. R alone)	-2.31 [-5.45 to 0.83]	0.149	42	0.109
Puglisi et al. 2008 (walk vs. R+walk)	-2.37 [-5.44 to 0.71]	0.131	43	0.107

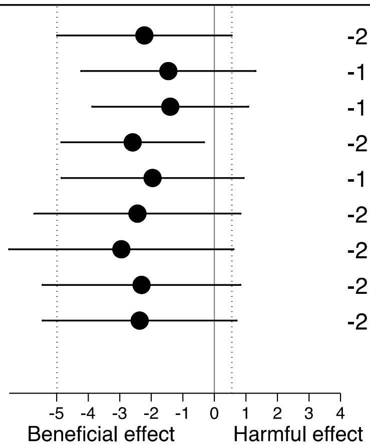

Influence analysis: removal of each trial, one at a time and recalculation of the overall effect and heterogeneity

CI=confidence interval; R=raisin

S25 Fig. Sensitivity analysis of the systematic removal of each trial for the effect of sweets and desserts on SBP (mmHg) in substitution trials.

Influence analysis

Systolic blood pressure (mmHg) in substitution sweets and desserts trials

**Trial removed**	Mean difference with 95% CI	P_{effect}	I^2 (%)	P_Q
**Overall**	-1.29 [-3.47 to 0.90]	0.248	0	0.817
Claesson et al. 2009	-1.19 [-3.52 to 1.14]	0.317	0	0.729
Dikariyanto et a. 2020	-1.45 [-4.18 to 1.27]	0.298	0	0.727
Hallfrisch et al. 1983 (C, 15%)	-1.26 [-3.46 to 0.94]	0.259	0	0.726
Hallfrisch et al. 1983 (C, 7.5%)	-1.26 [-3.46 to 0.94]	0.259	0	0.726
Hallfrisch et al. 1983 (HI, 15%)	-0.97 [-3.34 to 1.40]	0.422	0	0.779
Hallfrisch et al. 1983 (HI, 7.5%)	-0.79 [-3.16 to 1.58]	0.514	0	0.860
Israel et al. 1983 (18%)	-1.77 [-4.07 to 0.53]	0.131	0	0.922
Israel et al. 1983 (33%)	-1.59 [-3.87 to 0.69]	0.172	0	0.825

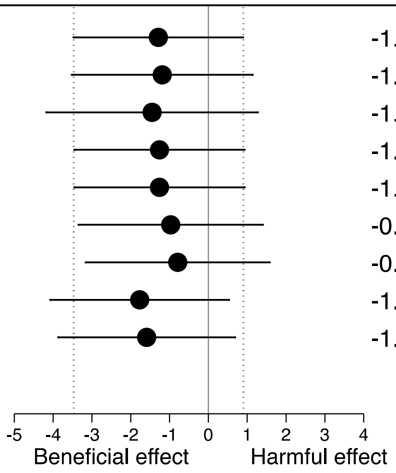

Influence analysis: removal of each trial, one at a time and recalculation of the overall effect and heterogeneity

CI=confidence interval; HI=hyper insulinemic

S26 Fig. Sensitivity analysis of the systematic removal of each trial for the effect of added nutritive (caloric) sweetener on SBP (mmHg) in substitution trials.

Influence analysis

Systolic blood pressure (mmHg) in substitution added nutritive (caloric) sweetener trials

Trial removed	Mean difference with 95% CI	P_{effect}	I^2 (%)	P_Q
**Overall**	-1.67 [-3.99 to 0.66]	0.158	0	0.855
Beisner et al. 2020 (OB, HFS)	-1.67 [-4.15 to 0.81]	0.186	0	0.759
Beisner et al. 2020 (lean, HFS)	-1.79 [-4.19 to 0.62]	0.145	0	0.779
Cooper et al. 1988	-1.80 [-4.34 to 0.74]	0.164	0	0.768
Despland et al. 2017 (honey)	-1.91 [-4.42 to 0.60]	0.135	0	0.796
Despland et al. 2017 (sugars)	-2.15 [-4.66 to 0.35]	0.092	0	0.901
Koh et al. 1988 (IGTS)	-0.41 [-3.30 to 2.48]	0.782	0	0.991
Koh et al. 1988 (NGTS)	-1.66 [-4.03 to 0.71]	0.167	0	0.759

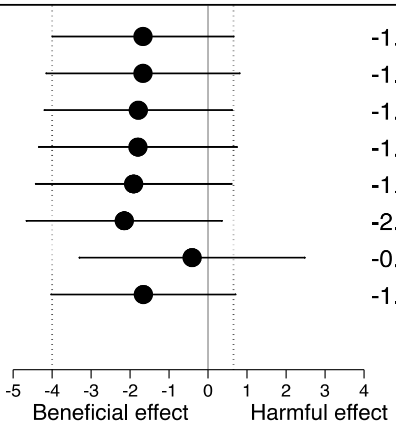

Influence analysis: removal of each trial, one at a time and recalculation of the overall effect and heterogeneity

CI, confidence interval; HFS=high fructose syrup; IGTS, impaired glucose tolerance; NGTS, normal glucose tolerance; OB=obese

S27 Fig. Sensitivity analysis of the systematic removal of each trial for the effect of mixed sources (with SSBs) on SBP (mmHg) in substitution trials.

Influence analysis

Systolic blood pressure (mmHg) in substitution mixed sources (with SSBs) trials

Trial removed	Mean difference with 95% CI	P_{effect}	I^2 (%)	P_Q
**Overall**	1.48 [-0.91 to 3.87]	0.224	43	0.050
Black et al. 2006	1.93 [-0.53 to 4.39]	0.125	41	0.069
Brymora et al. 2012 (basal diet)	1.25 [-1.32 to 3.82]	0.340	46	0.040
Brymora et al. 2012 (resumed diet)	1.53 [-1.09 to 4.15]	0.253	48	0.033
Dominiguez-Coello et al. 2020	1.50 [-1.29 to 4.29]	0.292	48	0.033
Jalilvand et al. 2020	1.52 [-1.27 to 4.31]	0.285	48	0.033
Lewis et al. 2013	1.11 [-1.48 to 3.70]	0.400	43	0.055
Luukkonen et al. 2018 (SF)	1.58 [-0.91 to 4.07]	0.214	47	0.035
Luukkonen et al. 2018 (UnSF)	1.62 [-0.83 to 4.07]	0.196	46	0.039
Madero et al. 2015	1.64 [-0.99 to 4.27]	0.221	47	0.036
Nier et al. 2018	1.41 [-1.08 to 3.90]	0.268	47	0.035
Schwimmer et al. 2019	1.13 [-1.37 to 3.63]	0.375	44	0.053
Surwit et al. 1997	2.03 [-0.02 to 4.08]	0.053	24	0.206
Vos et al. 2009	1.10 [-0.78 to 2.99]	0.252	16	0.288

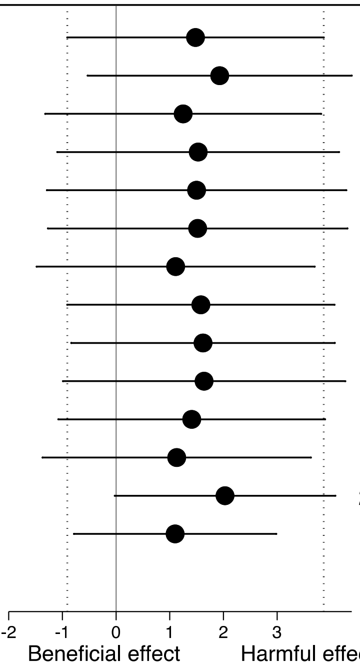

Influence analysis: removal of each trial, one at a time and recalculation of the overall effect and heterogeneity

CI=confidence interval; SF=saturated fat, UnSF=unsaturated fat

S28 Fig. Sensitivity analysis of the systematic removal of each trial for the effect of mixed sources (without SSBs) on SBP (mmHg) in substitution trials.

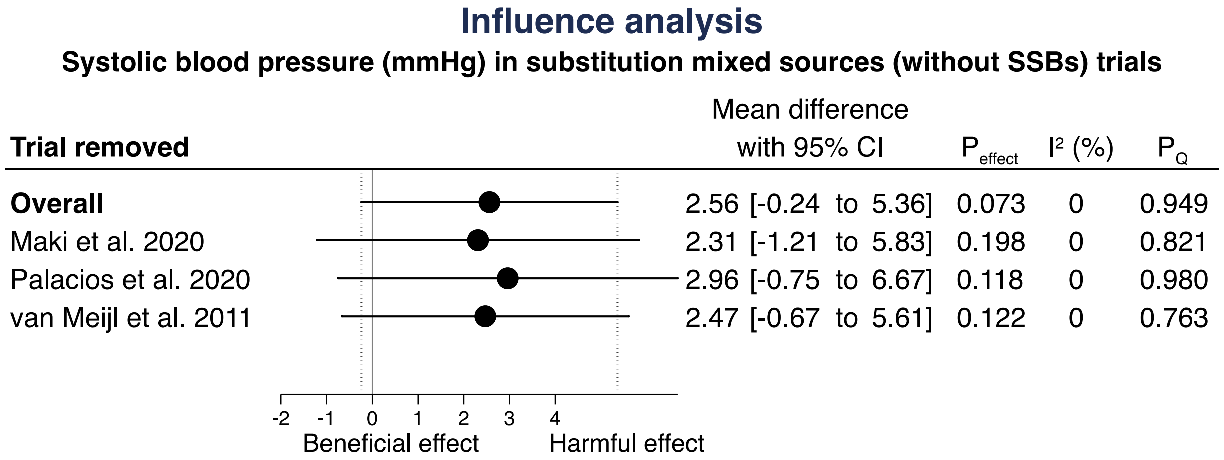

Influence analysis: removal of each trial, one at a time and recalculation of the overall effect and heterogeneity

CI=confidence interval

S29 Fig. Sensitivity analysis of the systematic removal of each trial for the effect of SSBs on SBP (mmHg) in addition trials.

Influence analysis

Systolic blood pressure (mmHg) in addition SSB trials

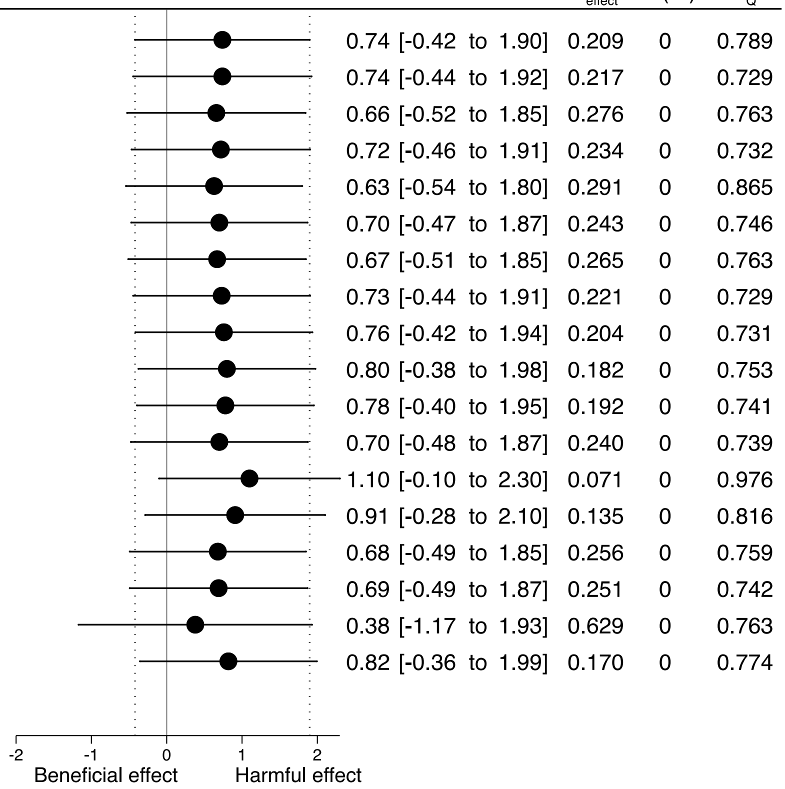

Trial removed	Mean difference with 95% CI	P_{effect}	I^2 (%)	P_Q
**Overall**	0.74 [-0.42 to 1.90]	0.209	0	0.789
Aeberli et al. 2011 (control vs. HF)	0.74 [-0.44 to 1.92]	0.217	0	0.729
Aeberli et al. 2011 (control vs. HS)	0.66 [-0.52 to 1.85]	0.276	0	0.763
Aeberli et al. 2011 (control vs. MF)	0.72 [-0.46 to 1.91]	0.234	0	0.732
Engel et al. 2017 (NSB)	0.63 [-0.54 to 1.80]	0.291	0	0.865
Engel et al. 2017 (water)	0.70 [-0.47 to 1.87]	0.243	0	0.746
Geidl-Flueck et al. 2021 (CNTRL vs FRC)	0.67 [-0.51 to 1.85]	0.265	0	0.763
Geidl-Flueck et al. 2021 (CNTRL vs SUC)	0.73 [-0.44 to 1.91]	0.221	0	0.729
Hieronimus et al. 2020 (Frc17.5)	0.76 [-0.42 to 1.94]	0.204	0	0.731
Hieronimus et al. 2020 (Frc25)	0.80 [-0.38 to 1.98]	0.182	0	0.753
Hieronimus et al. 2020 (HFCS17.5)	0.78 [-0.40 to 1.95]	0.192	0	0.741
Hieronimus et al. 2020 (HFCS25)	0.70 [-0.48 to 1.87]	0.240	0	0.739
Njike et al. 2011 (placebo)	1.10 [-0.10 to 2.30]	0.071	0	0.976
Njike et al. 2011 (sugar-sweetened)	0.91 [-0.28 to 2.10]	0.135	0	0.816
Sigala et al. 2020	0.68 [-0.49 to 1.85]	0.256	0	0.759
Silbernagel et al. 2011	0.69 [-0.49 to 1.87]	0.251	0	0.742
Stanhope et al. 2009	0.38 [-1.17 to 1.93]	0.629	0	0.763
Stanhope et al. 2015 (HFCS10)	0.82 [-0.36 to 1.99]	0.170	0	0.774

Influence analysis: removal of each trial, one at a time and recalculation of the overall effect and heterogeneity

CI=confidence interval; CNTRL=control; FRC=fructose; Frc17.5=17.5% fructose; Frc25=25% fructose; HF=high fructose; HFCS10=10% high fructose corn syrup; HFCS17.5=17.5% high fructose corn syrup; HFCS25=25% high fructose corn syrup; HS=high sucrose; MF=medium fructose; NSB=non-nutritive sweetened beverage; SUC=sucrose

S30 Fig. Sensitivity analysis of the systematic removal of each trial for the effect of 100% fruit juice on SBP (mmHg) in addition trials.

Influence analysis

Systolic blood pressure (mmHg) in addition 100% fruit juice trials

Trial removed	Mean difference with 95% CI	P_{effect}	I^2 (%)	P_Q
**Overall**	-3.74 [-5.28 to -2.20]	< 0.001	53	0.006
Abedini et al. 2020	-3.37 [-4.83 to -1.91]	< 0.001	47	0.023
Aghababaee et al. 2015	-4.01 [-5.68 to -2.35]	< 0.001	53	0.009
Alatas et al. 2019	-3.66 [-5.32 to -2.00]	< 0.001	56	0.004
Amagase et al. 2009	-3.92 [-5.49 to -2.35]	< 0.001	54	0.006
Asgary et al. 2014	-3.49 [-5.10 to -1.88]	< 0.001	53	0.009
Banini et al. 2006	-3.76 [-5.34 to -2.18]	< 0.001	57	0.004
Esmaeilinezhad et al. 2020 (PJ vs. placebo)	-3.65 [-5.37 to -1.94]	< 0.001	55	0.005
Esmaeilinezhad et al. 2020 (SPJ vs. SB)	-3.87 [-5.78 to -1.96]	< 0.001	57	0.004
Kojadinovic et al. 2017	-3.85 [-5.39 to -2.30]	< 0.001	54	0.006
Lazavi et al. 2018	-3.43 [-4.78 to -2.08]	< 0.001	41	0.048
Neto et al. 2017	-3.79 [-5.41 to -2.17]	< 0.001	56	0.004
Ravn-Haren et al. 2013 (control vs. clear juice)	-3.81 [-5.45 to -2.17]	< 0.001	56	0.004
Ravn-Haren et al. 2013 (control vs. cloudy juice)	-3.94 [-5.56 to -2.32]	< 0.001	55	0.006
Ravn-Haren et al. 2013 (pomace vs. clear juice)	-4.07 [-5.61 to -2.53]	< 0.001	50	0.015
Ravn-Haren et al. 2013 (pomace vs. cloudy juice)	-3.74 [-5.38 to -2.10]	< 0.001	57	0.004
Shema-Didi et al. 2014	-3.57 [-5.07 to -2.07]	< 0.001	52	0.011

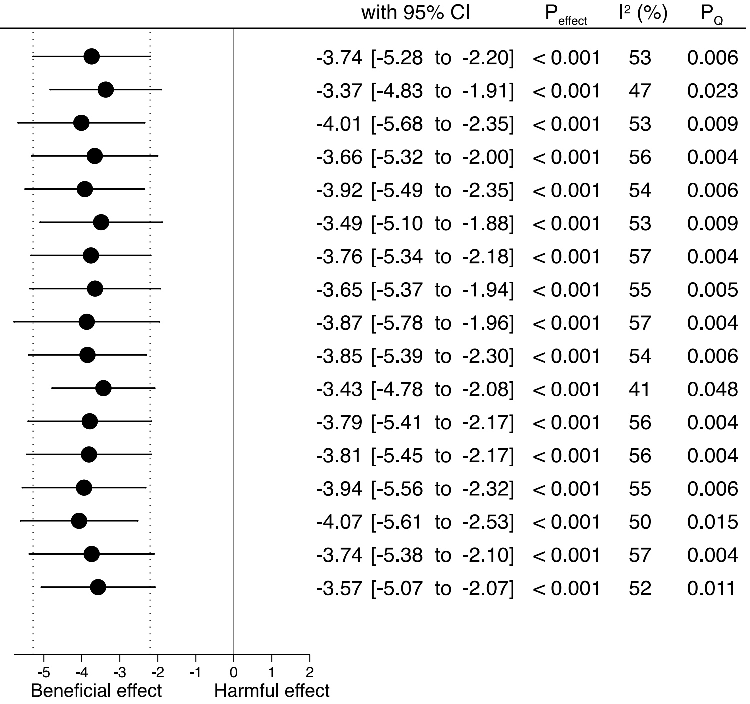

Influence analysis: removal of each trial, one at a time and recalculation of the overall effect and heterogeneity

CI=confidence interval; PJ=pomegranate juice; SB=symbiotic beverage SPJ=symbiotic pomegranate juice

S31 Fig. Sensitivity analysis of the systematic removal of each trial for the effect of fruit on SBP (mmHg) in addition trials.

Influence analysis

Systolic blood pressure (mmHg) in addition fruit trials

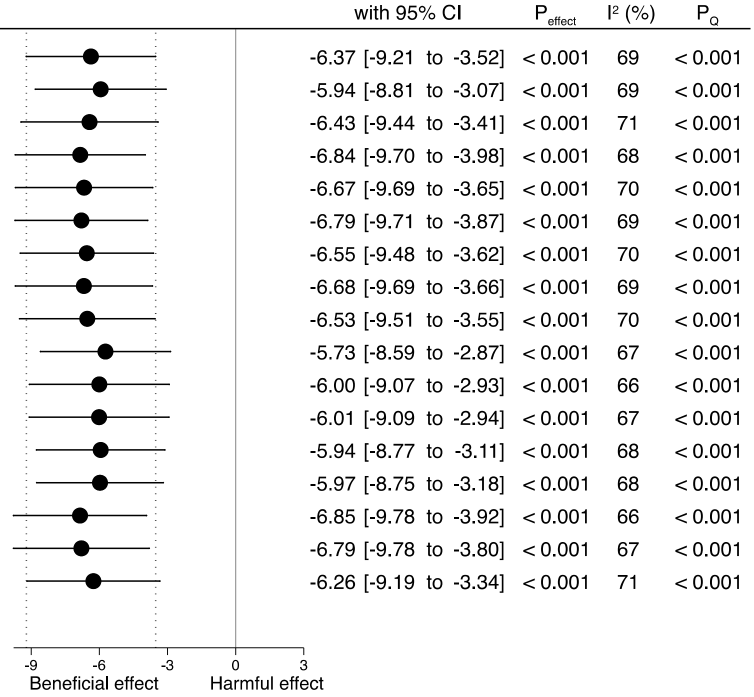

Trial removed	Mean difference with 95% CI	P_{effect}	I^2 (%)	P_Q
**Overall**	-6.37 [-9.21 to -3.52]	< 0.001	69	< 0.001
Amani et al. 2014	-5.94 [-8.81 to -3.07]	< 0.001	69	< 0.001
Basu et al. 2010 (blueberry)	-6.43 [-9.44 to -3.41]	< 0.001	71	< 0.001
Basu et al. 2010 (strawberry)	-6.84 [-9.70 to -3.98]	< 0.001	68	< 0.001
Cressey et al. 2014 (HCL, 250g)	-6.67 [-9.69 to -3.65]	< 0.001	70	< 0.001
Cressey et al. 2014 (HCL, 500g)	-6.79 [-9.71 to -3.87]	< 0.001	69	< 0.001
Cressey et al. 2014 (T2D, 250g)	-6.55 [-9.48 to -3.62]	< 0.001	70	< 0.001
Dow et al. 2012	-6.68 [-9.69 to -3.66]	< 0.001	69	< 0.001
Franck et al. 2020	-6.53 [-9.51 to -3.55]	< 0.001	70	< 0.001
Karlsen et al. 2013 (hypertensives)	-5.73 [-8.59 to -2.87]	< 0.001	67	< 0.001
Karlsen et al. 2013 (normal/high-normal BP)	-6.00 [-9.07 to -2.93]	< 0.001	66	< 0.001
Karlsen et al. 2013 (optimal BP)	-6.01 [-9.09 to -2.94]	< 0.001	67	< 0.001
Kumari et al. 2016 (with peel)	-5.94 [-8.77 to -3.11]	< 0.001	68	< 0.001
Kumari et al. 2016 (without peel)	-5.97 [-8.75 to -3.18]	< 0.001	68	< 0.001
Ravn-Haren et al. 2013 (control vs. whole apple)	-6.85 [-9.78 to -3.92]	< 0.001	66	< 0.001
Ravn-Haren et al. 2013 (pomace vs. whole apple)	-6.79 [-9.78 to -3.80]	< 0.001	67	< 0.001
Schell et al. 2019	-6.26 [-9.19 to -3.34]	< 0.001	71	< 0.001

Influence analysis: removal of each trial, one at a time and recalculation of the overall effect and heterogeneity

CI=confidence interval; HCL=hypercholesterolemic; T2D=type 2 diabetes

S32 Fig. Sensitivity analysis of the systematic removal of each trial for the effect of dried fruit on SBP (mmHg) in addition trials.

Influence analysis

Systolic blood pressure (mmHg) in addition dried fruit trials

**Trial removed**	Mean difference with 95% CI	P_{effect}	I^2 (%)	P_Q
**Overall**	-1.96 [-4.69 to 0.77]	0.160	0	0.970
Ahmed et al. 2010	-2.17 [-5.13 to 0.79]	0.151	0	0.942
Al-Dashti et al. 2019 (run-in vs. 2P)	-1.68 [-4.79 to 1.43]	0.290	0	0.943
Al-Dashti et al. 2019 (run-in vs. 6P)	-2.01 [-4.89 to 0.87]	0.172	0	0.926
Al-Dashti et al. 2019 (washout vs. 2P)	-2.22 [-5.07 to 0.63]	0.127	0	0.972
Al-Dashti et al. 2019 (washout vs. 6P)	-1.99 [-5.31 to 1.33]	0.241	0	0.924
Irannejad et al. 2020	-1.66 [-4.56 to 1.25]	0.264	0	0.970

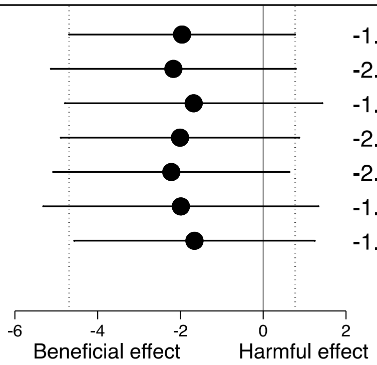

Influence analysis: removal of each trial, one at a time and recalculation of the overall effect and heterogeneity

CI=confidence interval; P=prunes

S33 Fig. Sensitivity analysis of the systematic removal of each trial for the effect of sweetened cereal grains and bars on SBP (mmHg) in addition trials.

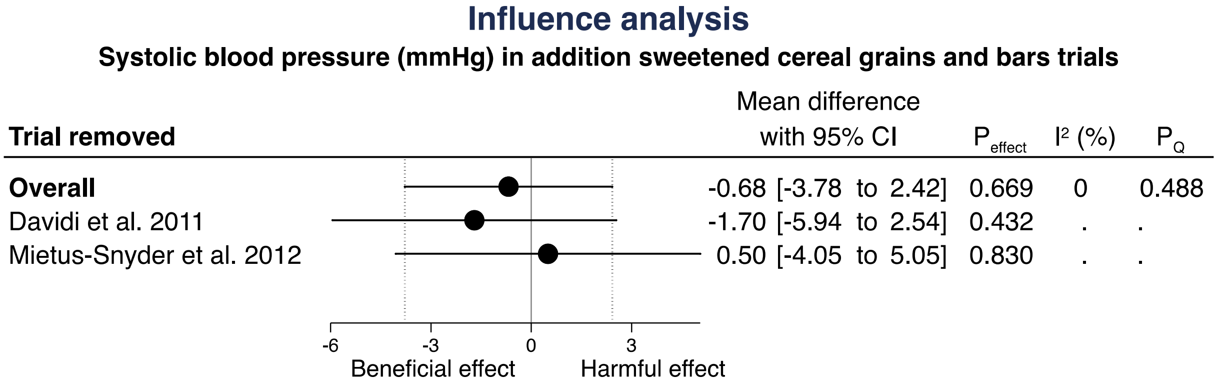

Influence analysis: removal of each trial, one at a time and recalculation of the overall effect and heterogeneity

CI=confidence interval

S34 Fig. Sensitivity analysis of the systematic removal of each trial for the effect of sweets and desserts on SBP (mmHg) in addition trials.

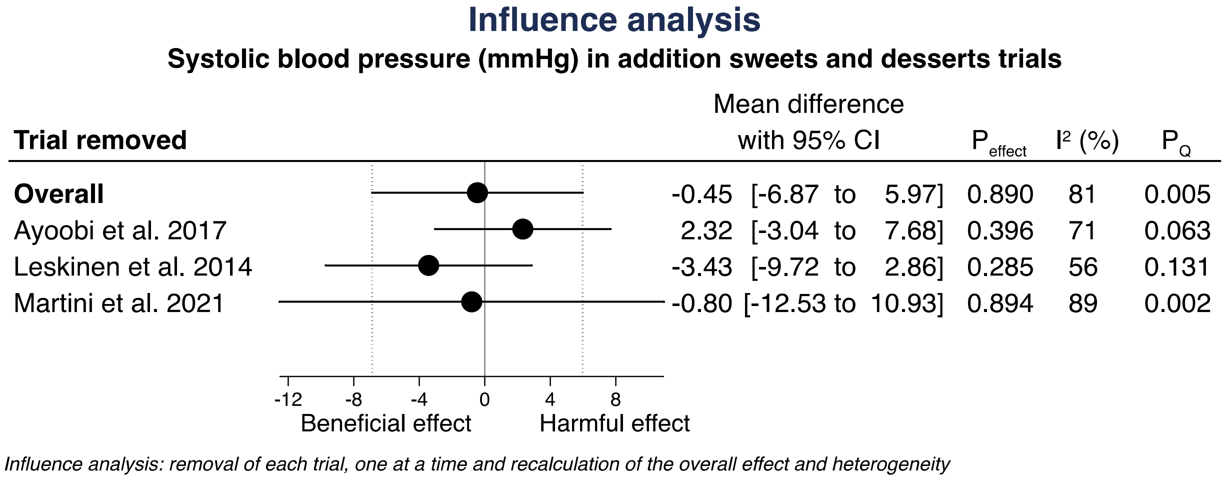

CI=confidence interval

S35 Fig. Sensitivity analysis of the systematic removal of each trial for the effect of added nutritive (caloric) sweetener on SBP (mmHg) in addition trials.

Influence analysis

Systolic blood pressure (mmHg) in addition added nutritive (caloric) sweetener trials

Trial removed	Mean difference with 95% CI	P_{effect}	I^2 (%)	P_Q
**Overall**	4.58 [-2.04 to 11.20]	0.176	0	0.891
Tang et al. 2020 (20 g)	5.58 [-2.45 to 13.61]	0.173	0	0.837
Tang et al. 2020 (40 g)	3.60 [-4.60 to 11.80]	0.389	0	0.784
Tang et al. 2020 (60 g)	4.50 [-3.61 to 12.61]	0.277	0	0.631

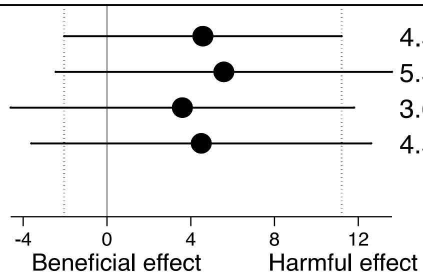

Influence analysis: removal of each trial, one at a time and recalculation of the overall effect and heterogeneity

CI=confidence interval

S36 Fig. Sensitivity analysis of the systematic removal of each trial for the effect of SSBs on SBP (mmHg) in subtraction trials.

Influence analysis

Systolic Blood Pressure in subtraction SSB trials

Trial removed	Mean difference with 95% CI	P_{effect}	I^2 (%)	P_Q
**Overall**	-0.47 [-2.70 to 1.76]	0.676	50	0.053
Al-Dujaili et al. 2017	-1.51 [-2.86 to -0.16]	0.029	0	0.981
Campos et al. 2015 (G1)	-0.39 [-2.70 to 1.92]	0.740	56	0.035
Campos et al. 2015 (G2)	-0.46 [-2.81 to 1.89]	0.705	57	0.031
Ebbeling et al. 2020 (ASB)	-0.20 [-2.74 to 2.34]	0.876	54	0.041
Ebbeling et al. 2020 (water)	-0.62 [-3.25 to 2.01]	0.643	57	0.031
Hernandez-Cordero et al. 2014	-0.29 [-3.14 to 2.56]	0.840	48	0.075
Tate et al. 2012 (NSB)	-0.33 [-2.84 to 2.18]	0.796	56	0.034
Tate et al. 2012 (water)	-0.22 [-2.69 to 2.25]	0.859	55	0.039

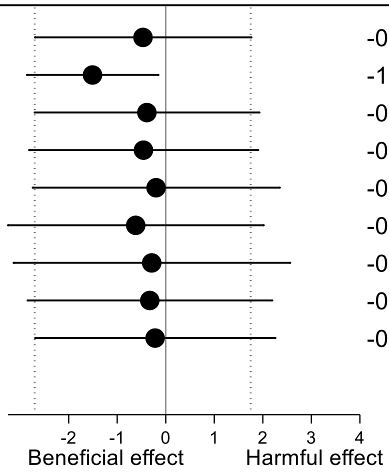

Influence analysis: removal of each trial, one at a time and recalculation of the overall effect and heterogeneity

ASB=artificially sweetened beverage; CI=confidence interval; G1=group1; G2=group 2; NSB=non-nutritive sweetened beverage

S37 Fig. Sensitivity analysis of the systematic removal of each trial for the effect of mixed sources (with SSBs) on SBP (mmHg) in subtraction trials*.

Influence analysis

Systolic blood pressure in subtraction mixed sources (with SSBs) trials

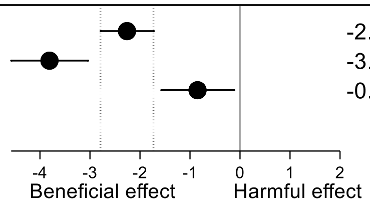

Trial removed	Mean difference with 95% CI	P_{effect}	I^2 (%)	P_Q
**Overall**	-2.26 [-2.79 to -1.73]	< 0.001	97	< 0.001
Vazquez-Duran et al. 2016 (NSB)	-3.81 [-4.57 to -3.04]	< 0.001	.	.
Vazquez-Duran et al. 2016 (water)	-0.85 [-1.58 to -0.13]	0.022	.	.

Influence analysis: removal of each trial, one at a time and recalculation of the overall effect and heterogeneity

*fixed effects model

CI=confidence interval; NSB=non-nutritive sweetened beverage

S38 Fig. Sensitivity analysis of the systematic removal of each trial for the effect of SSBs on DBP (mmHg) in addition trials.

Influence analysis

Diastolic blood pressure (mmHg) in addition SSB trials

Trial removed	Mean difference with 95% CI	P_{effect}	I^2 (%)	P_Q
**Overall**	0.23 [-0.71 to 1.17]	0.630	0	0.868
Aeberli et al. 2011 (control vs. HF)	0.20 [-0.75 to 1.15]	0.682	0	0.830
Aeberli et al. 2011 (control vs. HS)	0.25 [-0.71 to 1.21]	0.605	0	0.825
Aeberli et al. 2011 (control vs. MF)	0.29 [-0.67 to 1.25]	0.558	0	0.840
Engel et al. 2017 (NSB)	0.12 [-0.83 to 1.07]	0.798	0	0.961
Engel et al. 2017 (water)	0.20 [-0.74 to 1.14]	0.685	0	0.842
Geidl-Flueck et al. 2021 (CNTRL vs FRC)	0.21 [-0.73 to 1.15]	0.670	0	0.830
Geidl-Flueck et al. 2021 (CNTRL vs SUC)	0.23 [-0.72 to 1.18]	0.633	0	0.822
Hieronimus et al. 2020 (Frc17.5)	0.11 [-0.84 to 1.06]	0.820	0	0.920
Hieronimus et al. 2020 (Frc25)	0.21 [-0.74 to 1.16]	0.659	0	0.823
Hieronimus et al. 2020 (HFCS17.5)	0.20 [-0.75 to 1.15]	0.675	0	0.827
Hieronimus et al. 2020 (HFCS25)	0.16 [-0.80 to 1.12]	0.744	0	0.859
Njike et al. 2011 (placebo)	0.15 [-0.82 to 1.13]	0.770	0	0.843
Njike et al. 2011 (sugar-sweetened)	0.25 [-0.72 to 1.22]	0.608	0	0.824
Sigala et al. 2020	0.14 [-0.82 to 1.10]	0.767	0	0.881
Silbernagel et al. 2011	0.25 [-0.70 to 1.20]	0.607	0	0.828
Stanhope et al. 2009	1.07 [-0.20 to 2.35]	0.101	0	0.973
Stanhope et al. 2015 (HFCS10)	0.24 [-0.71 to 1.19]	0.622	0	0.822

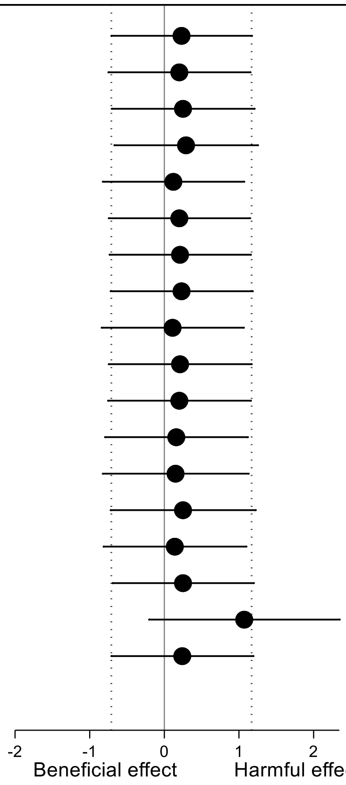

Influence analysis: removal of each trial, one at a time and recalculation of the overall effect and heterogeneity

CI=confidence interval; CNTRL=control; FRC=fructose; Frc17.5=17.5% fructose; Frc25=25% fructose; HF=high fructose; HFCS10=10% high fructose corn syrup; HFCS17.5=17.5% high fructose corn syrup; HFCS25=25% high fructose corn syrup; HS=high sucrose; MF=medium fructose; NSB=non-nutritive sweetened beverage; SUC=sucrose

S39 Fig. Sensitivity analysis of the systematic removal of each trial for the effect of 100% fruit juice on DBP (mmHg) in addition trials.

Influence analysis

Diastolic blood pressure (mmHg) in addition 100% fruit juice trials

Trial removed	Mean difference with 95% CI	P_{effect}	I^2 (%)	P_Q
**Overall**	-2.06 [-3.46 to -0.65]	0.004	47	0.019
Abedini et al. 2020	-1.90 [-3.36 to -0.44]	0.011	48	0.019
Aghababaee et al. 2015	-2.42 [-3.82 to -1.03]	< 0.001	37	0.073
Alatas et al. 2019	-2.02 [-3.59 to -0.45]	0.012	51	0.013
Amagase et al. 2009	-2.20 [-3.69 to -0.71]	0.004	49	0.017
Asgary et al. 2014	-1.91 [-3.42 to -0.40]	0.013	49	0.017
Banini et al. 2006	-2.10 [-3.54 to -0.65]	0.004	50	0.013
Esmaeilinezhad et al. 2020 (PJ vs. placebo)	-1.81 [-3.25 to -0.37]	0.013	44	0.034
Esmaeilinezhad et al. 2020 (SPJ vs. SB)	-1.84 [-3.31 to -0.38]	0.014	46	0.027
Kojadinovic et al. 2017	-2.34 [-3.56 to -1.12]	< 0.001	32	0.116
Lazavi et al. 2018	-1.87 [-3.25 to -0.49]	0.008	45	0.031
Neto et al. 2017	-2.14 [-3.57 to -0.71]	0.003	49	0.016
Ravn-Haren et al. 2013 (control vs. clear juice)	-2.01 [-3.53 to -0.49]	0.010	50	0.013
Ravn-Haren et al. 2013 (control vs. cloudy juice)	-2.15 [-3.64 to -0.66]	0.005	50	0.014
Ravn-Haren et al. 2013 (pomace vs. clear juice)	-2.28 [-3.71 to -0.84]	0.002	46	0.025
Ravn-Haren et al. 2013 (pomace vs. cloudy juice)	-1.99 [-3.50 to -0.48]	0.010	50	0.014
Shema-Didi et al. 2014	-1.99 [-3.43 to -0.55]	0.007	50	0.014

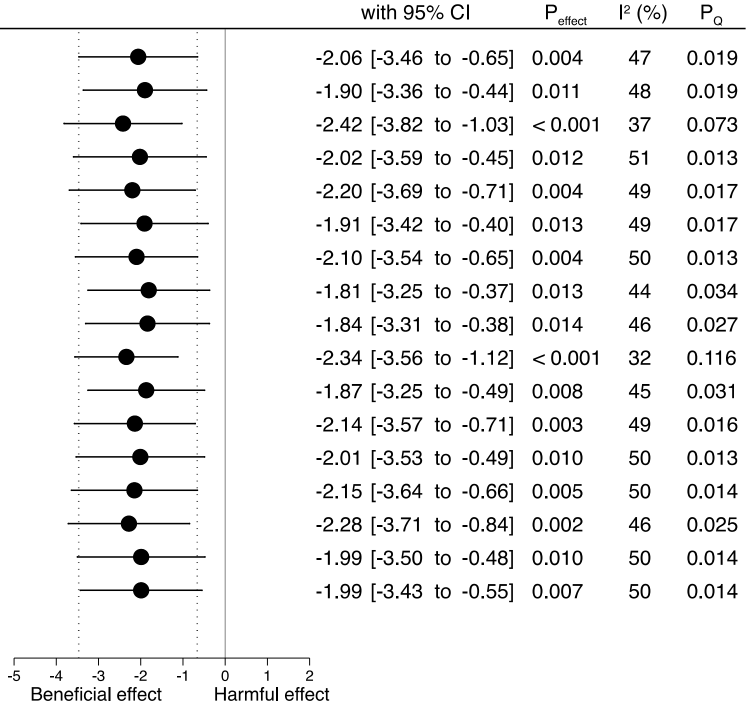

Influence analysis: removal of each trial, one at a time and recalculation of the overall effect and heterogeneity

CI=confidence interval; SB=symbiotic beverage; SPJ= symbiotic pomegranate juice; PJ=pomegranate juice

S40 Fig. Sensitivity analysis of the systematic removal of each trial for the effect of fruit on DBP (mmHg) in addition trials.

Influence analysis

Diastolic blood pressure (mmHg) in addition fruit trials

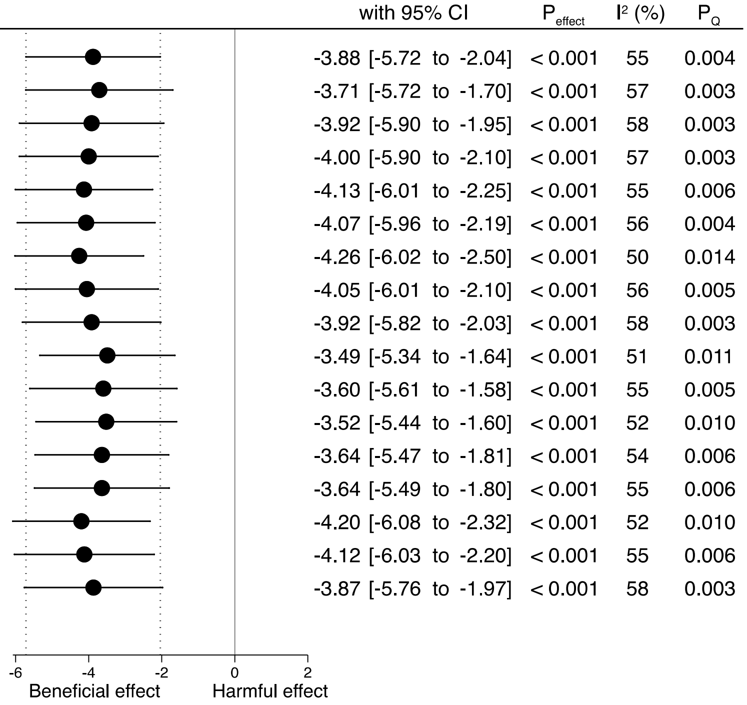

Trial removed	Mean difference with 95% CI	P_{effect}	I^2 (%)	P_Q
**Overall**	-3.88 [-5.72 to -2.04]	< 0.001	55	0.004
Amani et al. 2014	-3.71 [-5.72 to -1.70]	< 0.001	57	0.003
Basu et al. 2010a (blueberry)	-3.92 [-5.90 to -1.95]	< 0.001	58	0.003
Basu et al. 2010b (strawberry)	-4.00 [-5.90 to -2.10]	< 0.001	57	0.003
Cressey et al. 2014 (HCL, 250g)	-4.13 [-6.01 to -2.25]	< 0.001	55	0.006
Cressey et al. 2014 (HCL, 500g)	-4.07 [-5.96 to -2.19]	< 0.001	56	0.004
Cressey et al. 2014 (T2D, 250g)	-4.26 [-6.02 to -2.50]	< 0.001	50	0.014
Dow et al. 2012	-4.05 [-6.01 to -2.10]	< 0.001	56	0.005
Franck et al. 2020	-3.92 [-5.82 to -2.03]	< 0.001	58	0.003
Karlsen et al. 2013 (hypertensives)	-3.49 [-5.34 to -1.64]	< 0.001	51	0.011
Karlsen et al. 2013 (normal/high-normal BP)	-3.60 [-5.61 to -1.58]	< 0.001	55	0.005
Karlsen et al. 2013 (optimal BP)	-3.52 [-5.44 to -1.60]	< 0.001	52	0.010
Kumari et al. 2016 (with peel)	-3.64 [-5.47 to -1.81]	< 0.001	54	0.006
Kumari et al. 2016 (without peel)	-3.64 [-5.49 to -1.80]	< 0.001	55	0.006
Ravn-Haren et al. 2013 (control vs. whole apple)	-4.20 [-6.08 to -2.32]	< 0.001	52	0.010
Ravn-Haren et al. 2013 (pomace vs. whole apple)	-4.12 [-6.03 to -2.20]	< 0.001	55	0.006
Schell et al. 2019	-3.87 [-5.76 to -1.97]	< 0.001	58	0.003

Influence analysis: removal of each trial, one at a time and recalculation of the overall effect and heterogeneity

CI, confidence interval; HCL=hypercholesterolemic; T2D=type 2 diabetes

S41 Fig. Sensitivity analysis of the systematic removal of each trial for the effect of dried fruit on DBP (mmHg) in addition trials.

Influence analysis

Diastolic blood pressure (mmHg) in addition dried fruit trials

Trial removed	Mean difference with 95% CI	P_{effect}	I^2 (%)	P_Q
**Overall**	-0.60 [-1.79 to 0.59]	0.321	0	0.874
Ahmed et al. 2010	-0.90 [-2.69 to 0.89]	0.320	0	0.805
Al-Dashti et al. 2019 (run-in vs. 2P)	-0.58 [-1.85 to 0.69]	0.371	0	0.772
Al-Dashti et al. 2019 (run-in vs. 6P)	-0.52 [-1.75 to 0.72]	0.407	0	0.818
Al-Dashti et al. 2019 (washout vs. 2P)	-0.69 [-1.92 to 0.54]	0.270	0	0.830
Al-Dashti et al. 2019 (washout vs. 6P)	-0.36 [-1.67 to 0.95]	0.593	0	0.907
Irannejad et al. 2020	-0.69 [-1.91 to 0.53]	0.265	0	0.861

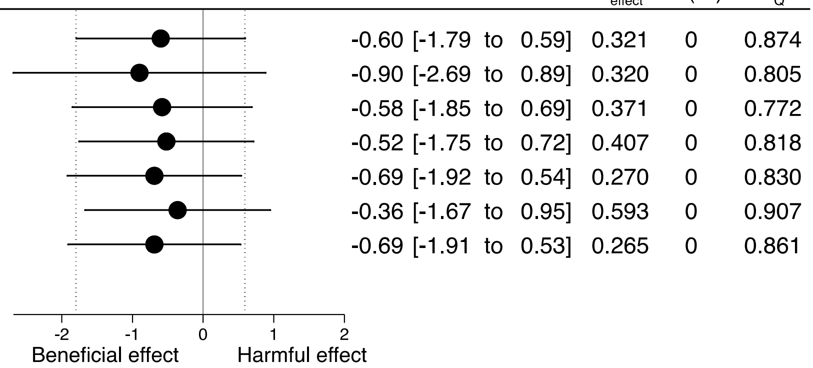

Influence analysis: removal of each trial, one at a time and recalculation of the overall effect and heterogeneity

CI, confidence interval; P=prunes

S42 Fig. Sensitivity analysis of the systematic removal of each trial for the effect of sweetened cereal grains and bars on DBP (mmHg) in addition trials.

Influence analysis

Diastolic blood pressure (mmHg) in addition sweeteend cereal grains and bars trials

Trial removed	Mean difference with 95% CI	P_{effect}	I^2 (%)	P_Q
**Overall**	-0.80 [-3.64 to 2.04]	0.578	46	0.175
Davidi et al. 2011	-2.70 [-6.44 to 1.04]	0.157	.	.
Mietus-Snyder et al. 2012	0.30 [-1.90 to 2.50]	0.789	.	.

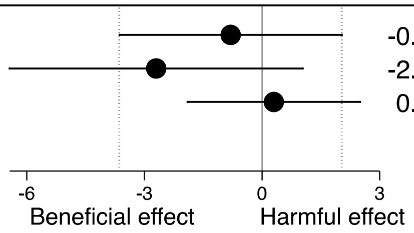

Influence analysis: removal of each trial, one at a time and recalculation of the overall effect and heterogeneity

CI=confidence interval

S43 Fig. Sensitivity analysis of the systematic removal of each trial for the effect of sweets and desserts on DBP (mmHg) in addition trials.

Influence analysis

Diastolic blood pressure (mmHg) in addition sweets and desserts trials

Trial removed	Mean difference with 95% CI	P_{effect}	I^2 (%)	P_Q
**Overall**	-0.38 [-7.43 to 6.67]	0.917	88	< 0.001
Ayoobi et al. 2017	2.14 [-5.99 to 10.27]	0.605	91	< 0.001
Leskinen et al. 2014	-3.39 [-7.33 to 0.55]	0.092	31	0.229
Martini et al. 2021	0.25 [-12.09 to 12.59]	0.968	92	< 0.001

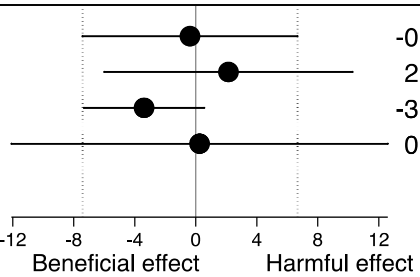

Influence analysis: removal of each trial, one at a time and recalculation of the overall effect and heterogeneity

CI=confidence interval

S44 Fig. Sensitivity analysis of the systematic removal of each trial for the effect of added nutritive (caloric) sweetener on DBP (mmHg) in addition trials.

Influence analysis

Diastolic blood pressure (mmHg) in addition added nutritive (caloric) sweetener trials

Trial removed	Mean difference with 95% CI	P_{effect}	I^2 (%)	P_Q
**Overall**	2.49 [-2.88 to 7.86]	0.364	0	0.909
Tang et al. 2020 (20 g)	2.73 [-3.85 to 9.31]	0.416	0	0.676
Tang et al. 2020 (40 g)	1.67 [-4.92 to 8.25]	0.620	0	0.918
Tang et al. 2020 (60 g)	3.07 [-3.50 to 9.64]	0.360	0	0.752

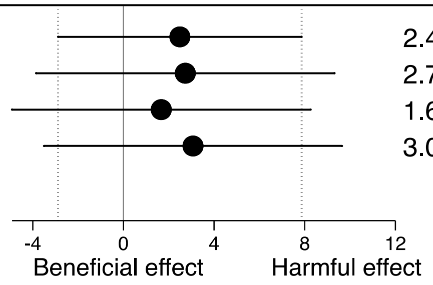

Influence analysis: removal of each trial, one at a time and recalculation of the overall effect and heterogeneity

CI=confidence interval

S45 Fig. Sensitivity analysis of the systematic removal of each trial for the effect of SSBs on DBP (mmHg) in subtraction trials.

Influence analysis

Diastolic Blood Pressure in subtraction SSB trials

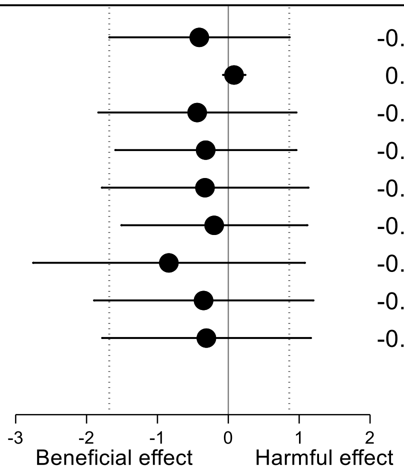

**Trial removed**	Mean difference with 95% CI	P_{effect}	I^2 (%)	P_Q
**Overall**	-0.41 [-1.68 to 0.86]	0.530	29	0.198
Al-Dujaili et al. 2017	0.08 [-0.07 to 0.23]	0.274	0	0.523
Campos et al. 2015 (G1)	-0.44 [-1.84 to 0.96]	0.535	39	0.135
Campos et al. 2015 (G2)	-0.32 [-1.59 to 0.96]	0.627	31	0.192
Ebbeling et al. 2020 (ASB)	-0.33 [-1.78 to 1.12]	0.653	34	0.166
Ebbeling et al. 2020 (water)	-0.20 [-1.51 to 1.11]	0.770	26	0.227
Hernandez-Cordero et al. 2014	-0.84 [-2.75 to 1.08]	0.392	27	0.220
Tate et al. 2012 (NSB)	-0.35 [-1.90 to 1.20]	0.659	34	0.168
Tate et al. 2012 (water)	-0.31 [-1.78 to 1.16]	0.676	33	0.175

Influence analysis: removal of each trial, one at a time and recalculation of the overall effect and heterogeneity

ASB=artificially sweetened beverage; CI=confidence interval; G1=group1; G2=group 2; NSB=non-nutritive sweetened beverage

S46 Fig. Sensitivity analysis of the systematic removal of each trial for the effect of mixed sources (with SSBs) on DBP (mmHg) in subtraction trials.

Influence analysis

Diastolic Blood Pressure in subtraction mixed sources (with SSBs) trials

Trial removed	Mean difference with 95% CI	P_{effect}	I^2 (%)	P_Q
**Overall**	0.05 [-1.51 to 1.61]	0.953	93	< 0.001
Vazquez-Duran et al. 2016 (NSB)	-0.75 [-1.35 to -0.15]	0.014	.	.
Vazquez-Duran et al. 2016 (water)	0.84 [0.24 to 1.44]	0.006	.	.

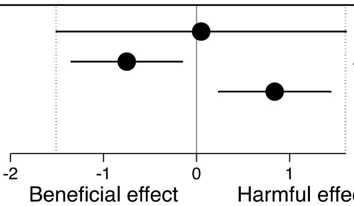

Influence analysis: removal of each trial, one at a time and recalculation of the overall effect and heterogeneity

CI=confidence interval; NSB=non-nutritive sweetened beverage

S47 Fig (part 1 of 3). Subgroup analyses for the effect of important food sources of fructose-containing sugars on SBP (mmHg) in substitution trials.

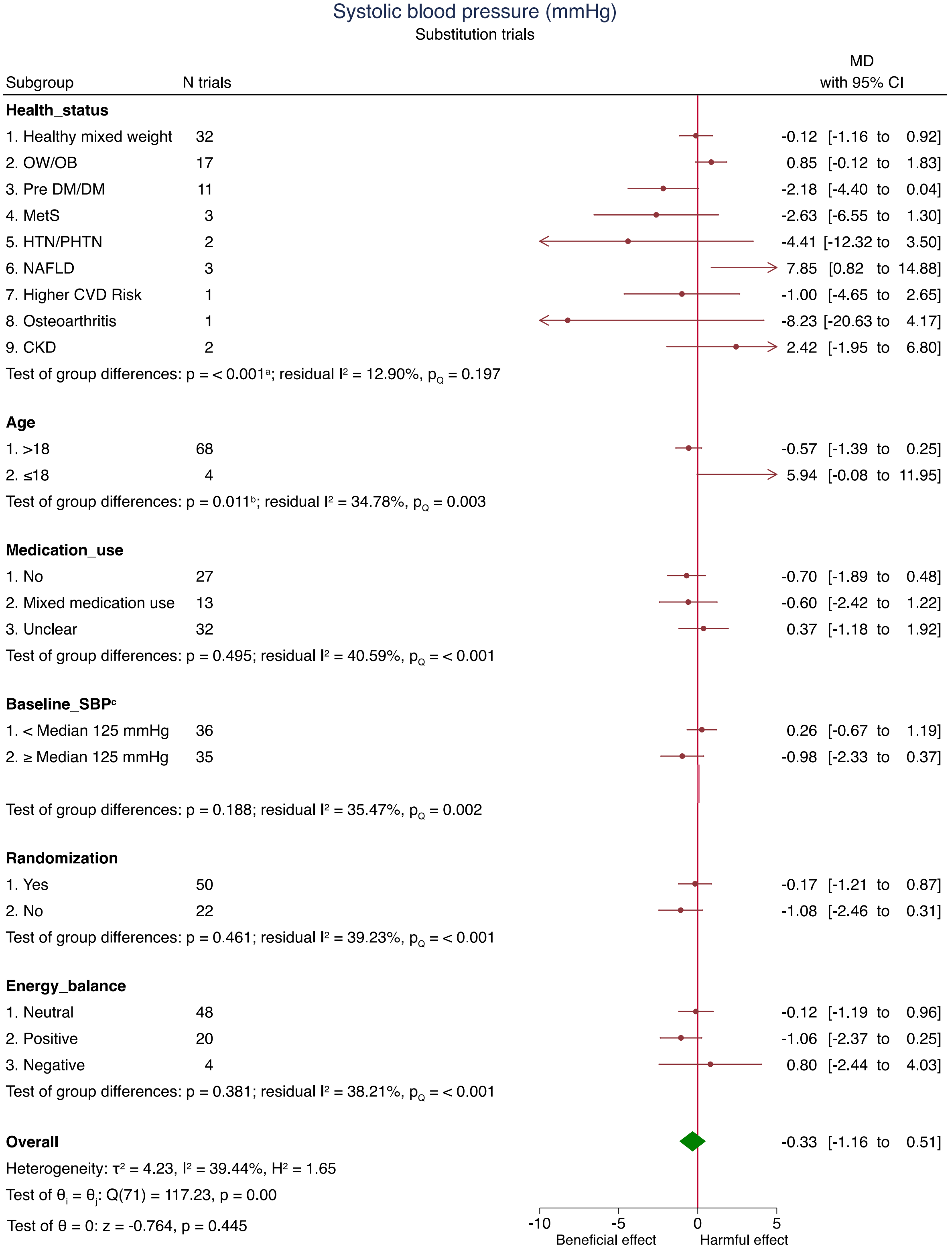

[a]Pairwise between-subgroup mean differences (95% confidence intervals) for health status were as follows: (2 vs. 1) 0.908 mmHg (-0.685, 2.5 mmHg); (3 vs. 1) -1.78 mmHg (-3.94, 0.369 mmHg); (4 vs. 1) -2.49 mmHg (-5.92, 0.369 mmHg); (5 vs. 1) -5.81 mmHg (-9.07, -2.54 mmHg); (6 vs. 1) 6.7 mmHg (2.15, 11.2 mmHg); (7 vs. 1) -0.871 mmHg (-5.17, 3.43 mmHg); (8 vs. 1) -8.1 mmHg (-20.7, 4.51 mmHg); (9 vs. 1) 2.56 mmHg (-2.17, 7.29 mmHg); (3 vs. 2) -2.69 mmHg (-4.85, -0.531 mmHg); (4 vs. 2) -3.4 mmHg (-6.83, 0.0377 mmHg); (5 vs. 2) -6.71 mmHg (-9.98, -3.45 mmHg); (6 vs. 2) 5.79 mmHg (1.23, 10.3 mmHg); (7 vs. 2) -1.78 mmHg (-6.08, 2.52 mmHg); (8 vs. 2) -9.01 mmHg (-21.6, 3.6 mmHg); (9 vs. 2) 1.65 (-3.08, 6.39 mmHg); (4 vs. 3) -7.06 mmHg (-4.43, 3.02mmHg); (5 vs. 3) -4.02 mmHg (-7.6, -0.448 mmHg); (6 vs. 3) 8.48 mmHg (3.7, 13.3 mmHg); (7 vs. 3) 0.913 mmHg (-3.62, 5.45 mmHg); (8 vs. 3) -6.32 mmHg (-19, 6.38 mmHg); (9 vs. 3) 4.34 mmHg (-0.604, 9.29 mmHg); (5 vs. 4) -3.32 mmHg (-7.78, 1.14 mmHg); (6 vs. 4) 9.19 mmHg (3.71, 14.7 mmHg); (7 vs. 4) 1.62 mmHg (-3.65, 6.88 mmHg); (8 vs. 4) -5.61 mmHg (-18.6, 7.36 mmHg); (9 vs. 4) 5.05 mmHg (-0.573, 10.7 mmHg); (6 vs. 5) 12.5 mmHg (7.13, 17.9 mmHg); (7 vs. 5) 4.94 mmHg (-0.223, 10.1 mmHg); (8 vs. 5) -2.29 mmHg (-15.2, 10.6 mmHg); (9 vs. 5) 8.37 mmHg (2.84, 13.9 mmHg); (7 vs. 6) -7.57 mmHg (-13.6, -1.51 mmHg); (8 vs. 6) -14.8 mmHg (-28.1, -1.48 mmHg); (9 vs. 6) -4.13 mmHg (-10.5, 2.23 mmHg); (9 vs. 6) -4.13 mmHg (-10.5, 2.23 mmHg); (8 vs. 7) -7.23 mmHg (-20.5, 6 mmHg); (9 vs. 7) 3.43 mmHg (-2.76, 9.62 mmHg); (9 vs. 8) 10.7 mmHg (-2.71, 24 mmHg)
[b]Pairwise between-subgroup mean differences (95% confidence intervals) for age were as follows: (2 vs. 1) 4.64 mmHg (1.07, 8.21 mmHg).
[c]N=1 trial missing data from baseline SBP.
The green diamond represents the pooled estimate for the overall primary analysis of food sources of fructose-containing sugars on DBP. Within subgroup mean differences are the pooled effect estimates represented by a red circle. 95% confidence intervals are represented by the line through the circle. Data are expressed as mean differences with 95% confidence intervals using the generic inverse-variance method and random effects DerSimonian-Laird model. Inter-study heterogeneity was assessed using the Cochrane Q statistic and quantified using the I^2 statistic, with significance set at $p<0.100$ and $I^2 \geq 50\%$ considered to be evidence of substantial heterogeneity. $p<0.050$ indicates that the effect size differed between levels of the subgroup.
CI=confidence interval; CKD=chronic kidney disease; DM=diabetes mellitus HTN=hypertensive; OW/OB=overweight/obese; MetS=metabolic syndrome; NAFLD= non-alcoholic fatty liver disease; PHTN=pre-hypertensive

S47 Fig (part 2 of 3). Subgroup analyses for the effect of important food sources of fructose-containing sugars on SBP (mmHg) in substitution trials.

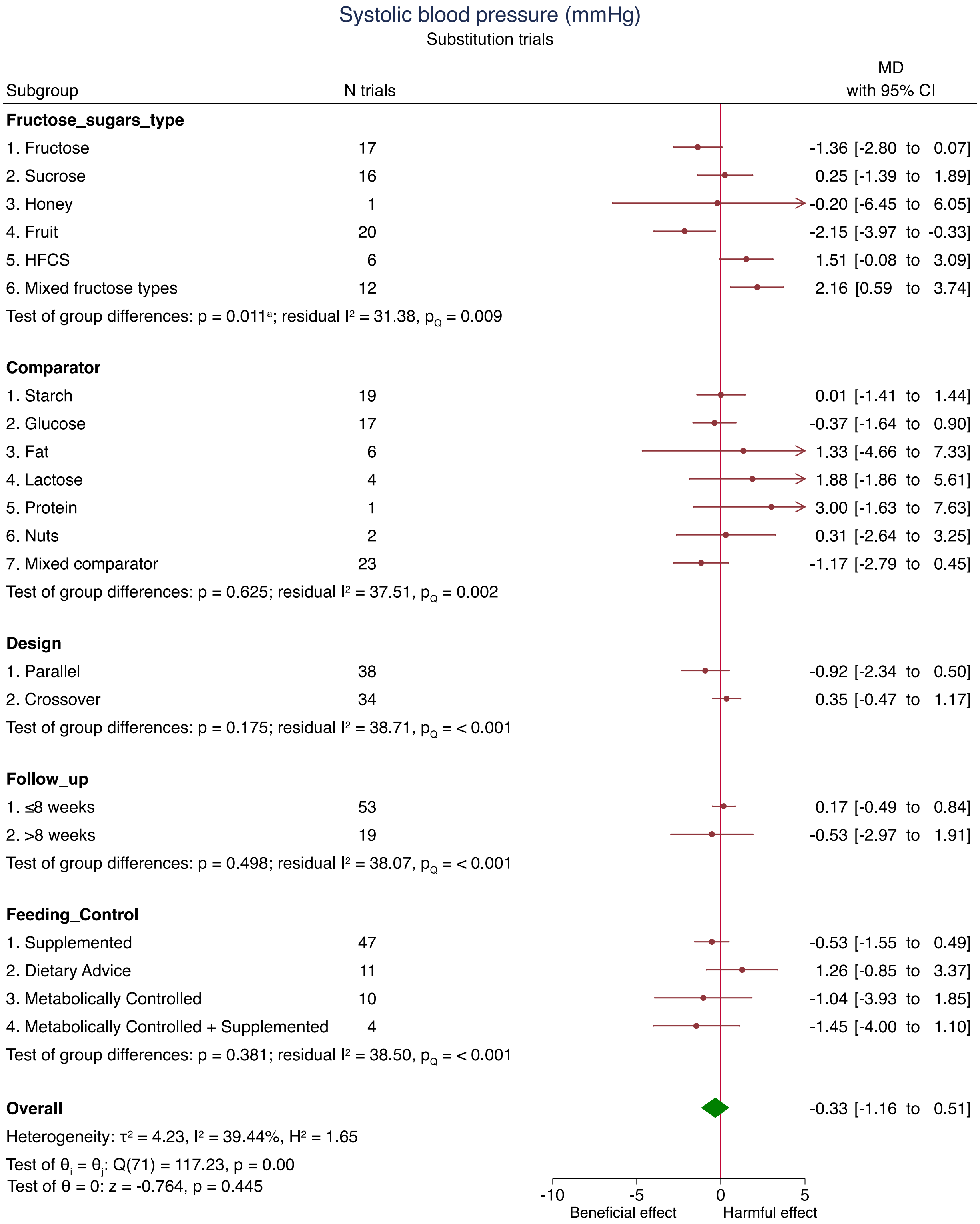

[a]Pairwise between-subgroup mean differences (95% confidence intervals) for fructose sugars type were as follows: (2 vs. 1) 1.62 mmHg (-0.835, 4.07 mmHg); (3 vs. 1) 1.17 mmHg (-6.22, 8.56 mmHg); (4 vs. 1) -0.577 mmHg (-2.88, 1.73 mmHg); (5 vs. 1) 2.49 mmHg (-0.563, 5.54 mmHg); (6 vs. 1) 3.69 mmHg (1.01, 6.37 mmHg); (3 vs. 2) -0.449 mmHg (-7.8, 6.9 mmHg); (4 vs. 2) -2.2 mmHg (-4.38, -0.011 mmHg); (5 vs. 2) 0.869 mmHg (-2.09, 3.38 mmHg); (6 vs. 2) 2.07 mmHg (-0.502, 4.65 mmHg); (4 vs. 3) -1.75 mmHg (-9.05, 5.56 mmHg); (5 vs. 3) 1.32 mmHg (-6.25, 8.89 mmHg); (6 vs. 3) 2.52 mmHg (-4.91, 9.95 mmHg); (5 vs. 4) 3.07 mmHg (0.226, 5.9 mmHg); (6 vs. 4) 4.27 mmHg (1.84, 6.71 mmHg); (6 vs. 5) 1.21 mmHg (-1.94, 4.36)

The green diamond represents the pooled estimate for the overall primary analysis of food sources of fructose-containing sugars on DBP. Within subgroup mean differences are the pooled effect estimates represented by a red circle. 95% confidence intervals are represented by the line through the circle. Data are expressed as mean differences with 95% confidence intervals using the generic inverse-variance method and random effects DerSimonian-Laird model. Inter-study heterogeneity was assessed using the Cochrane Q statistic and quantified using the I^2 statistic, with significance set at $p<0.100$ and $I^2 \geq 50\%$ considered to be evidence of substantial heterogeneity. $p<0.050$ indicates that the effect size differed between levels of the subgroup.

CI=confidence interval; HFCS=high fructose corn syrup

S47 Fig (part 3 of 3). Subgroup analyses for the effect of important food sources of fructose-containing sugars on SBP (mmHg) in substitution trials.

Systolic blood pressure (mmHg)

Substitution trials

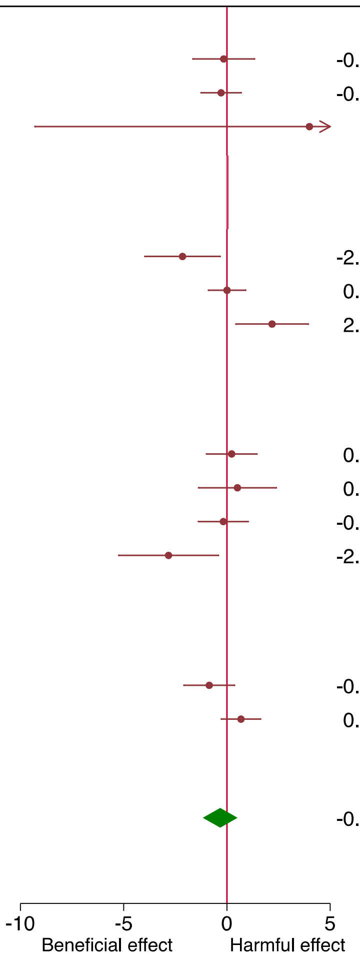

Subgroup	N trials	MD with 95% CI
**Dose**[a]		
1. ≤ 10%E	27	-0.15 [-1.64 to 1.34]
2. > 10%E	44	-0.28 [-1.25 to 0.69]
Test of group differences: p = 0.806; residual I^2 = 40.33%, p_Q = < 0.001		
**Regulatory_designation**		
1. Naturally occurring	20	-2.15 [-3.97 to -0.33]
2. Added	41	0.01 [-0.89 to 0.90]
3. Mixed regulatory designations	11	2.19 [0.44 to 3.94]
Test of group differences: p = 0.004; residual I^2 = 33.69%, p_Q = 0.004		
**Funding**		
1. Agency	30	0.23 [-0.99 to 1.45]
2. Industry	15	0.51 [-1.37 to 2.39]
3. Agency + Industry	16	-0.17 [-1.38 to 1.03]
4. Not Reported	11	-2.82 [-5.23 to -0.41]
Test of group differences: p = 0.030; residual I^2 = 28.63%, p_Q = 0.016		
**Type_of_mean_difference**		
1. Change from baseline	42	-0.86 [-2.08 to 0.37]
2. End differences	30	0.68 [-0.27 to 1.63]
Test of group differences: p = 0.130; residual I^2 = 37.68%, p_Q = < 0.001		
**Overall**		-0.33 [-1.16 to 0.51]
Heterogeneity: τ^2 = 4.23, I^2 = 39.44%, H^2 = 1.65		
Test of $\theta_i = \theta_j$: Q(71) = 117.23, p = 0.00		
Test of θ = 0: z = -0.764, p = 0.445		

[a]N=1 trial missing data for dose.

[b]Pairwise between-subgroup mean differences (95% confidence intervals) for regulatory designation were as follows: (2 vs. 1) 1.88 mmHg (0.0774, 3.68 mmHg); (3 vs. 1) 4.31 mmHg (1.73, 6.9 mmHg); (3 vs. 2) 2.43 mmHg (0.0353, 4.83 mmHg)

[c]Pairwise between-subgroup mean differences (95% confidence intervals) funding were as follows: (2 vs. 1) 0.423 mmHg (-1.68, 2.53 mmHg); (3 vs. 1) -0.486 mmHg (-2.4, 1.43 mmHg); (4 vs. 1) -3.34 mmHg (-5.79, -0.887 mmHg); (3 vs. 2) -0.909 mmHg (-3.08, 1.26 mmHg); (4 vs. 2) -3.76 mmHg (-6.42, -1.11 mmHg); (4 vs. 3) -2.85 mmHg (-5.36, -0.344 mmHg)

The green diamond represents the pooled estimate for the overall primary analysis of food sources of fructose-containing sugars on DBP. Within subgroup mean differences are the pooled effect estimates represented by a red circle. 95% confidence intervals are represented by the line through the circle. Data are expressed as mean differences with 95% confidence intervals using the generic inverse-variance method and random effects DerSimonian-Laird model. Inter-study heterogeneity was assessed using the Cochrane Q statistic and quantified

using the I^2 statistic, with significance set at $p<0.100$ and $I^2 \geq 50\%$ considered to be evidence of substantial heterogeneity. $p<0.050$ indicates that the effect size differed between levels of the subgroup.
%E=percent of daily energy intake; CI=confidence interval.

S48 Fig. Risk of bias (using The Cochrane Collaboration Tool) subgroup analysis for the effect of important food sources of fructose-containing sugars on SBP (mmHg) in substitution trials.

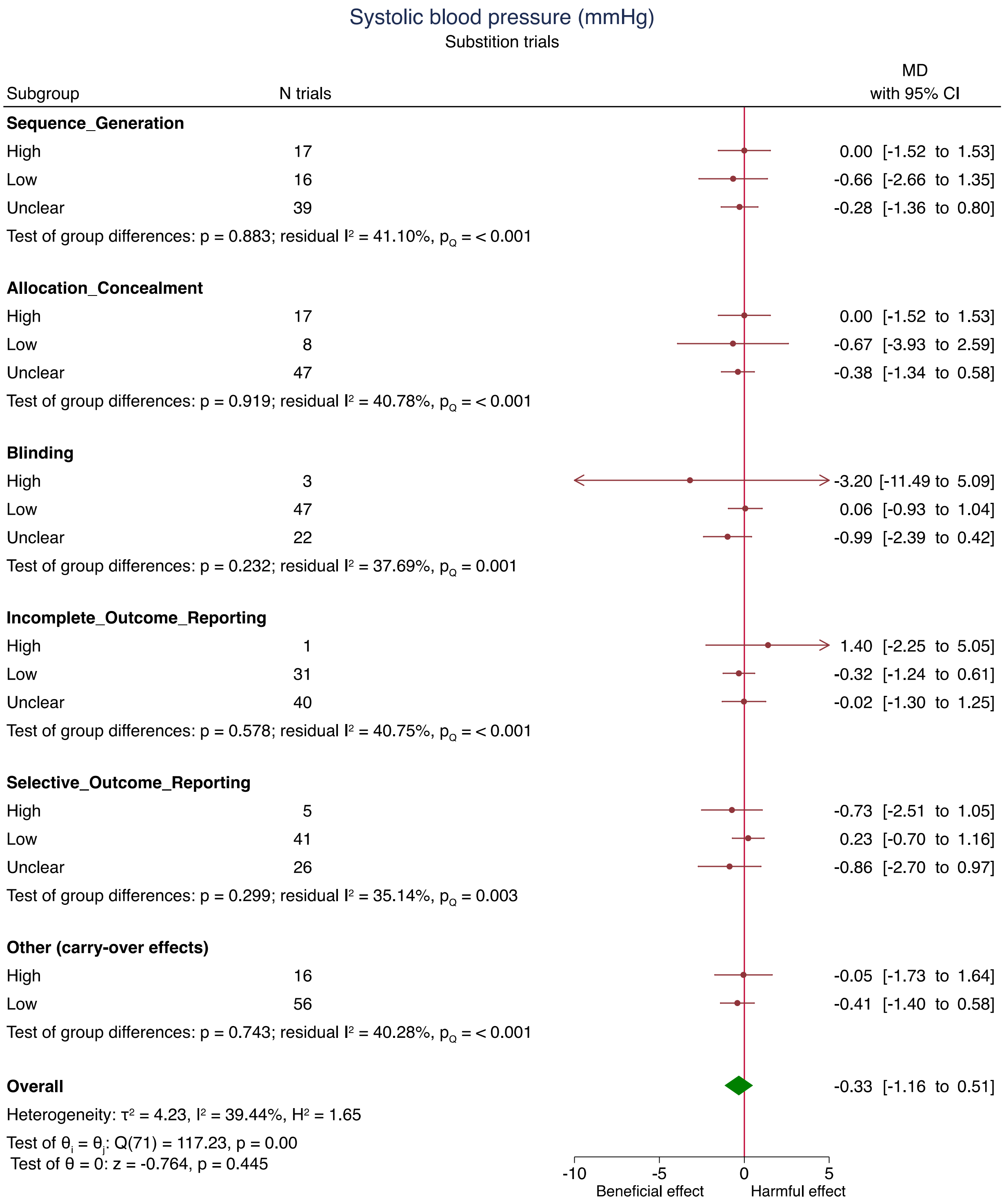

The green diamond represents the pooled estimate for the overall primary analysis of food sources of fructose-containing sugars on DBP. Within subgroup mean differences are the pooled effect estimates represented by a red circle. 95% confidence intervals are represented by the line through the circle. Data are expressed as mean differences with 95% confidence intervals using the generic inverse-variance method and random effects DerSimonian-Laird model. Inter-study heterogeneity was assessed using the Cochrane Q statistic and quantified using the I^2 statistic, with significance set at $p<0.100$ and $I^2\geq 50\%$ considered to be evidence of substantial heterogeneity. $p<0.050$ indicates that the effect size differed between levels of the subgroup.
CI=confidence interval; het=heterogeneity.

S49 Fig (part 1 of 3). Subgroup analyses for the effect of important food sources of fructose-containing sugars on SBP (mmHg) in addition trials.

Systolic blood pressure (mmHg)

Addition trials

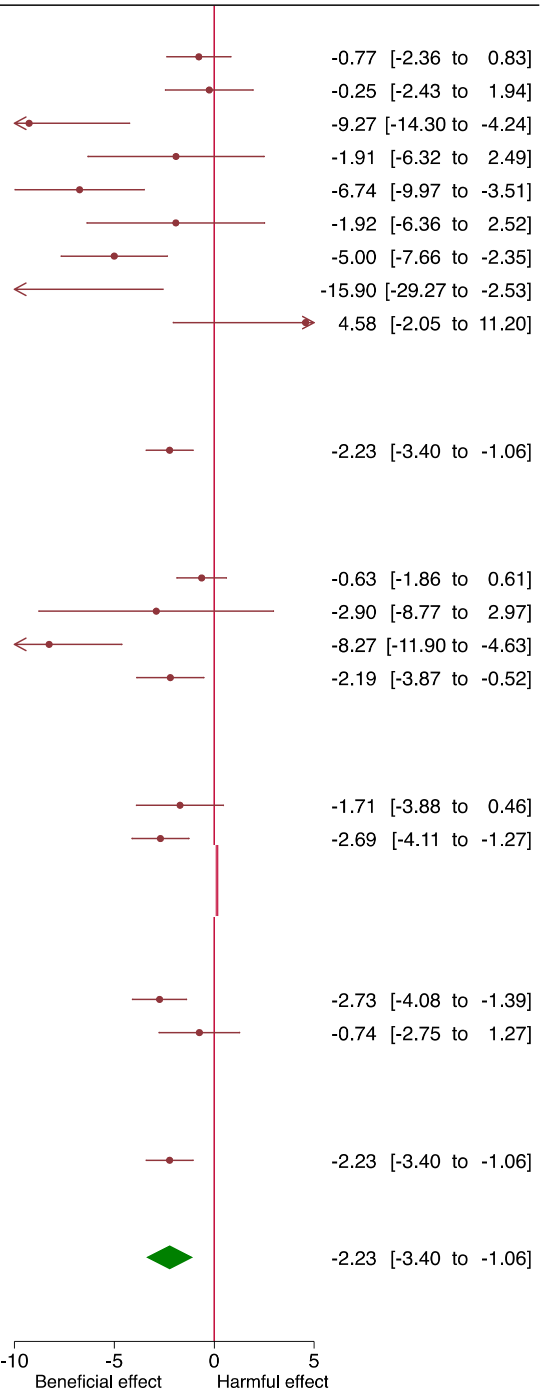

Subgroup	N trials	MD with 95% CI
**Health_status**		
1. Healthy mixed weight	31	-0.77 [-2.36 to 0.83]
2. OW/OB	8	-0.25 [-2.43 to 1.94]
3. Pre DM/DM	6	-9.27 [-14.30 to -4.24]
4. MetS	4	-1.91 [-6.32 to 2.49]
5. HTN/PHTN	6	-6.74 [-9.97 to -3.51]
6. Higher CVD Risk	2	-1.92 [-6.36 to 2.52]
7. PCOS	3	-5.00 [-7.66 to -2.35]
8. Hemodialysis	1	-15.90 [-29.27 to -2.53]
9. HIV	3	4.58 [-2.05 to 11.20]
Test of group differences: p = < 0.001[a]; residual I^2 = 55.37%, p_Q = < 0.001		
**Age**		
1. >18	64	-2.23 [-3.40 to -1.06]
**Medication_use**		
1. No	35	-0.63 [-1.86 to 0.61]
2. Yes	1	-2.90 [-8.77 to 2.97]
3. Mixed medication use	10	-8.27 [-11.90 to -4.63]
4. Unclear	18	-2.19 [-3.87 to -0.52]
Test of group differences: p = < 0.001[b]; residual I^2 = 53.23%, p_Q = < 0.001		
**Baseline_SBP[c]**		
1. < Median 122 mmHg	25	-1.71 [-3.88 to 0.46]
2. ≥ Median 122 mmHg	33	-2.69 [-4.11 to -1.27]
Test of group differences: p = 0.455; residual I^2 = 64.09%, p_Q = < 0.001		
**Randomization**		
1. Yes	48	-2.73 [-4.08 to -1.39]
2. No	16	-0.74 [-2.75 to 1.27]
Test of group differences: p = 0.139; residual I^2 = 65.82%, p_Q = < 0.001		
**Energy_balance**		
2. Positive	64	-2.23 [-3.40 to -1.06]
**Overall**		-2.23 [-3.40 to -1.06]
Heterogeneity: τ^2 = 12.43, I^2 = 69.32%, H^2 = 3.26		
Test of $\theta_i = \theta_j$: Q(63) = 205.32, p = 0.00		
Test of θ = 0: z = -3.743, p = 0.000		

[a]Pairwise between-subgroup mean differences (95% confidence intervals) for health status were as follows: (2 vs. 1) 0.493 mmHg (-2.43, 3.42 mmHg); (3 vs. 1) -8.38 mmHg (-13.2, -3.58 mmHg); (4 vs. 1) -0.901 mmHg (-6.28, 4.48 mmHg); (5 vs. 1) -5.99 mmHg (-9.31, -2.67 mmHg); (6 vs. 1) -0.967 mmHg (-7.12, 5.18 mmHg); (7 vs. 1) -4.65 mmHg (-8.61, -0.694 mmHg); (8 vs. 1) -15.1 mmHg (-29.7, -0.585 mmHg); (9 vs. 1) 5.34 mmHg (-2.15, 12.8 mmHg); (3 vs. 2) -8.88 mmHg (-14.1, -3.65 mmHg); (4 vs. 2) -1.39 mmHg (-7.16, 4.37 mmHg); (5 vs. 2) -6.48 mmHg (-10.4, -2.57 mmHg); (6 vs. 2) -1.46 mmHg (-7.95, 5.03 mmHg); (7 vs. 2) -5.14 mmHg (-9.61, -0.678 mmHg); (8 vs. 2) -15.6 mmHg (-30.3, -0.931 mmHg); (9 vs. 2) 4.85 (-2.93, 12.6 mmHg); (4 vs. 3) 7.48 mmHg (0.571, 14.4 mmHg); (5 vs. 3) 2.39 mmHg (-3.07, 7.85 mmHg); (6 vs. 3) 7.42 mmHg (-0.111, 14.9 mmHg); (7 vs. 3) 3.73 mmHg (-2.14, 9.6 mmHg); (8 vs. 3) -6.74 mmHg (-21.9, 8.43 mmHg); (9 vs. 3) 13.7 mmHg (5.07, 22.4 mmHg); (5 vs. 4) -5.09 mmHg (-11.1, 0.887 mmHg); (6 vs. 4) -0.0662 mmHg (-7.98, 7.84 mmHg); (7 vs. 4) -3.75 mmHg (-10.1, 2.6 mmHg); (8 vs. 4) -14.2 mmHg (-29.6, 1.14 mmHg); (9 vs. 4) 6.24 mmHg (-2.75, 15.2 mmHg); (6 vs. 5) 5.02 mmHg (-1.66, 11.7 mmHg); (7 vs. 5) 1.34 mmHg (-3.4, 6.08 mmHg); (8 vs. 5) -9.13 mmHg (-23.9, 5.64 mmHg); (9 vs. 5) 11.3 mmHg (3.4, 19.3 mmHg); (7 vs. 6) -3.68 mmHg (-10.7, 3.33 mmHg); (8 vs. 6) -14.2 mmHg (-29.8, 1.5 mmHg); (9 vs. 6) 6.31 mmHg (-3.17, 15.8 mmHg); (8 vs. 7) -10.5 mmHg (-25.4, 4.45 mmHg); (9 vs. 7) 9.99 mmHg (1.77, 18.2 mmHg); (9 vs. 8) 20.5 mmHg (4.24, 36.7 mmHg)
[b]Pairwise between-subgroup mean differences (95% confidence intervals) for medication use were as follows: (2 vs. 1) -2.22 mmHg (-10, 5.69 mmHg); (3 vs. 1) -7.42 mmHg (-10.4, -4.47 mmHg); (4 vs. 1) -1.46 mmHg (-3.68, 0.749 mmHg); (3 vs. 2) -5.21 mmHg (-13.4, 3.01 mmHg); (4 vs. 2) 0.751 mmHg (-7.23, 8.73 mmHg); (4 vs. 3) 5.96 mmHg (2.8, 9.11)
[c]N=6 trials missing data for baseline SBP.
The green diamond represents the pooled estimate for the overall primary analysis of food sources of fructose-containing sugars DBP. Within subgroup mean differences are the pooled effect estimates represented by a red circle. 95% confidence intervals are represented by the line through the circle. Data are expressed as mean differences with 95% confidence intervals using the generic inverse-variance method and random effects DerSimonian-Laird model. Inter-study heterogeneity was assessed using the Cochrane Q statistic and quantified using the I^2 statistic, with significance set at p<0.100 and $I^2 \geq 50\%$ considered to be evidence of substantial heterogeneity. p<0.050 indicates that the effect size differed between levels of the subgroup.
CI=confidence interval; CVD=cardiovascular disease, DM=diabetes mellitus; HIV=human immunodeficiency virus, HTN=hypertensive; MetS=metabolic syndrome; OW/OB=overweight/obese; PCOS=poly-cystic ovarian syndrome, PHTN=pre-hypertensive

S49 Fig (part 2 of 3). Subgroup analyses for the effect of important food sources of fructose-containing sugars on SBP (mmHg) in addition trials.

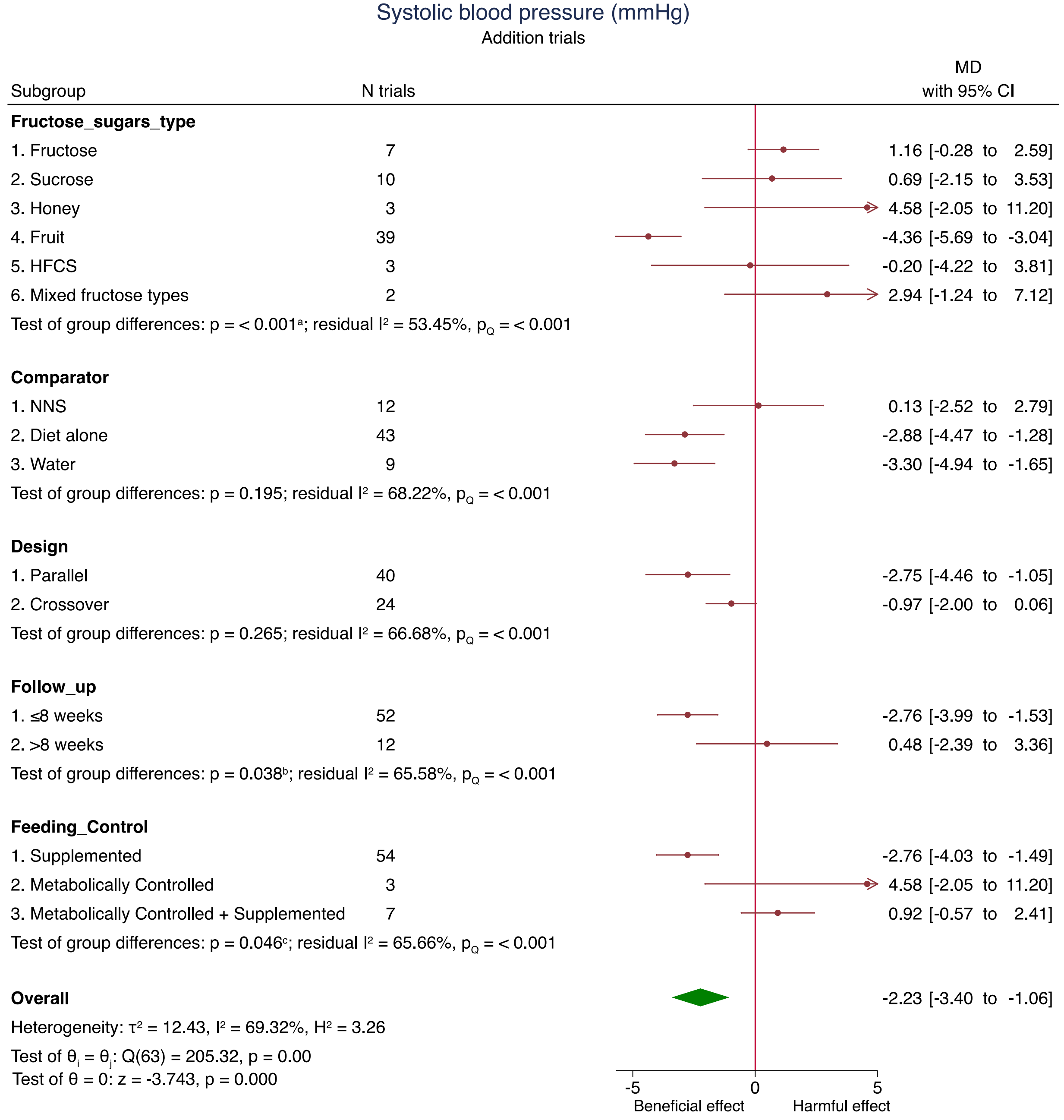

[a]Pairwise between-subgroup mean differences (95% confidence intervals) for fructose sugars type were as follows: (2 vs. 1) -0.446 mmHg (-4.19, 3.3 mmHg); (3 vs. 1) 3.49 mmHg (-4.28, 11.3 mmHg); (4 vs. 1) -5.42 mmHg (-8.49, -2.34 mmHg); (5 vs. 1) -1.3 mmHg (-7.01, 4.42 mmHg); (6 vs. 1) 1.82 mmHg (-3.53, 71.6 mmHg); (3 vs. 2) 3.94 mmHg (-3.72, 11.6 mmHg); (4 vs. 2) -4.97 mmHg (-7.74, -2.2 mmHg); (5 vs. 2) -0.852 mmHg (-6.41, 4.71 mmHg); (6 vs. 2) 2.26 mmHg (-2.91, 7.44 mmHg); (4 vs. 3) -8.91 mmHg (-16.3, -1.55 mmHg); (5 vs. 3) -4.79 mmHg (-13.6, 4.01 mmHg); (6 vs. 3) -1.67 mmHg (-10.2, 6.89 mmHg); (5 vs. 4) 4.12 mmHg (-1.02, 9.25 mmHg); (6 vs. 4) 7.23 mmHg (2.52, 12 mmHg); (6 vs. 5) 3.12 mmHg (-3.63, 9.86).

[b]Pairwise between-subgroup mean differences (95% confidence intervals) for follow up were as follows: (2 vs. 1) 3.24 mmHg (0.183, 6.29 mmHg).
The green diamond represents the pooled estimate for the overall primary analysis of food sources of fructose-containing sugars on DBP. Within subgroup mean differences are the pooled effect estimates represented by a red circle. 95% confidence intervals are represented by the line through the circle. Data are expressed as mean differences with 95% confidence intervals using the generic inverse-variance method and random effects DerSimonian-Laird model. Inter-study heterogeneity was assessed using the Cochrane Q statistic and quantified using the I^2 statistic, with significance set at $p<0.100$ and $I^2 \geq 50\%$ considered to be evidence of substantial heterogeneity. $p<0.050$ indicates that the effect size differed between levels of the subgroup.
CI=confidence interval; HFCS=high fructose corn syrup; NNS=non-nutritive sweetener

S49 Fig (part 3 of 3). Subgroup analyses for the effect of important food sources of fructose-containing sugars on SBP (mmHg) in addition trials.

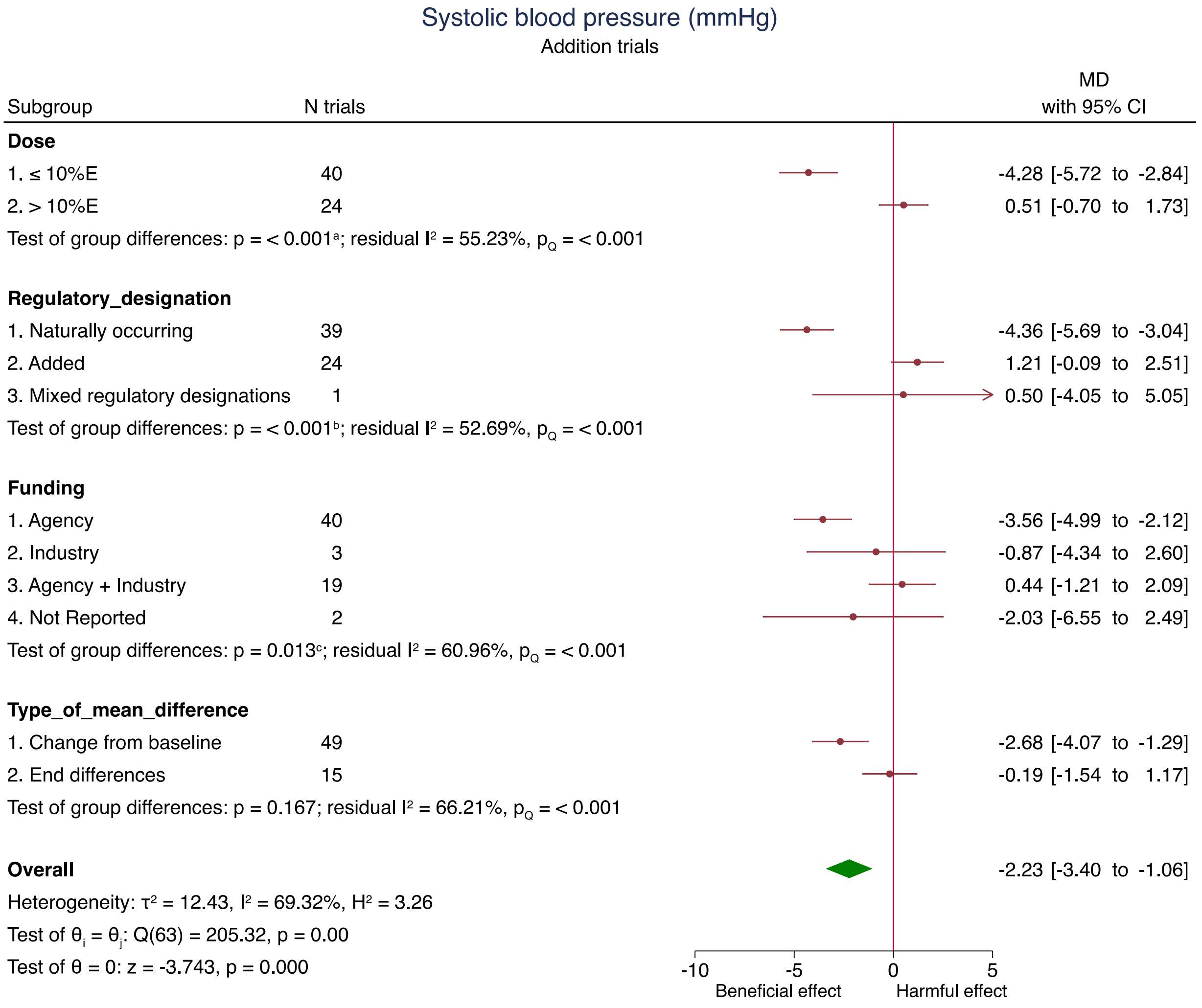

[a]Pairwise between-subgroup mean differences (95% confidence intervals) for dose were as follows: (2 vs. 1) 4.93 mmHg (2.92, 6.95 mmHg).
[b]Pairwise between-subgroup mean differences (95% confidence intervals) for regulatory designation were as follows: (2 vs. 1) 5.52 mmHg (3.49, 7.55 mmHg); (3 vs. 1) 4.83 mmHg (-2.02, 11.7 mmHg); (3 vs. 2) -0.687 mmHg (-7.62, 6.24 mmHg).
[c]Pairwise between-subgroup mean differences (95% confidence intervals) for funding were as follows: (2 vs. 1) 2.52 mmHg (-2.63, 7.67 mmHg); (3 vs. 1) 3.92 mmHg (1.56, 6.27 mmHg); (4 vs. 1) 1.62 mmHg (-4.68, 7.92 mmHg); (3 vs. 2) 1.4 mmHg (-3.94, 6.73 mmHg); (4 vs 2) -0.898 mmHg (-8.82, 7.02 mmHg); (4 vs. 3) -2.3 mmHg (-8.75, 4.16 mmHg).
The green diamond represents the pooled estimate for the overall primary analysis of food sources of fructose-containing sugars on DBP. Within subgroup mean differences are the pooled effect estimates represented by a red circle. 95% confidence intervals are represented by the line through the circle. Data are expressed as mean differences with 95% confidence intervals using the generic inverse-variance method and random effects DerSimonian-Laird model. Inter-study heterogeneity was assessed using the Cochrane Q statistic and quantified

using the I^2 statistic, with significance set at $p<0.100$ and $I^2 \geq 50\%$ considered to be evidence of substantial heterogeneity. $p<0.050$ indicates that the effect size differed between levels of the subgroup.
%E=percent of daily energy intake; CI=confidence interval.

S50 Fig. Risk of bias (using The Cochrane Collaboration Tool) subgroup analysis for the effect of important food sources of fructose-containing sugars on SBP (mmHg) in addition trials.

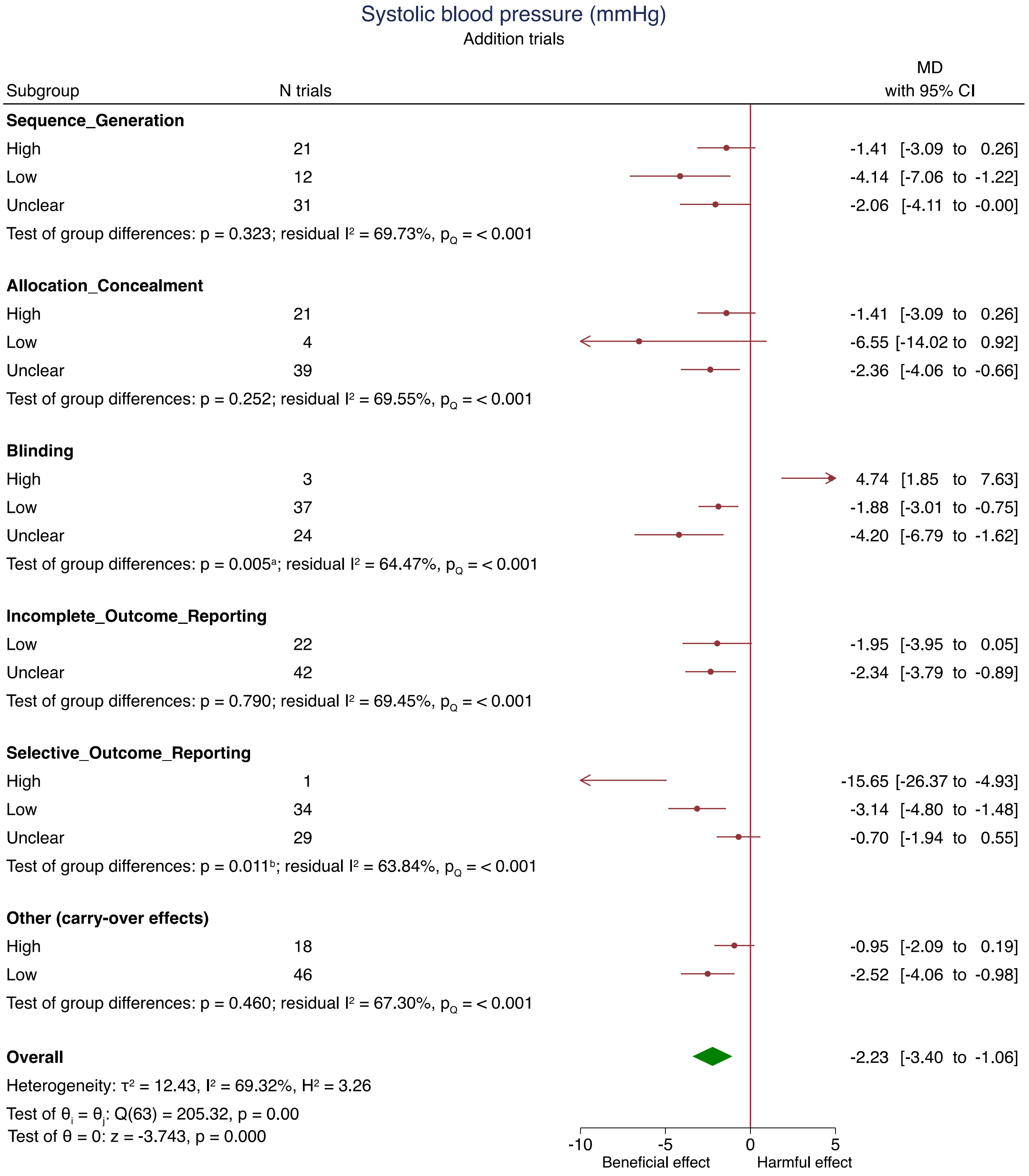

[a]Pairwise between-subgroup mean differences (95% confidence intervals) for blinding were as follows: (low vs. high) -6.55 mmHg (-11.8, -1.25 mmHg); (unclear vs. high) -8.72 mmHg (-14.2, -3.26 mmHg); (unclear vs. low) -2.17 mmHg (-4.54, 0.206 mmHg).

[b]Pairwise between-subgroup mean differences (95% confidence intervals) for selective outcome reporting were as follows: (low vs. high) 12.5 mmHg (-0.002, 25 mmHg); (unclear vs. high) 14.9 mmHg (2.36, 27.4 mmHg); (unclear vs. low) 2.38 mmHg (0.156, 4.6 mmHg).
The green diamond represents the pooled estimate for the overall primary analysis of food sources of fructose-containing sugars on SBP. Within subgroup mean differences are the pooled effect estimates represented by a red circle. 95% confidence intervals are represented by the line through the circle. Data are expressed as mean differences with 95% confidence intervals using the generic inverse-variance method and random effects DerSimonian-Laird model. Inter-study heterogeneity was assessed using the Cochrane Q statistic and quantified using the I^2 statistic, with significance set at $p<0.100$ and $I^2 \geq 50\%$ considered to be evidence of substantial heterogeneity. $p<0.050$ indicates that the effect size differed between levels of the subgroup.
CI=confidence interval;het=heterogeneity.

S51 Fig (part 1 of 3). Subgroup analyses for the effect of important food sources of fructose-containing sugars on SBP (mmHg) in subtraction trials.

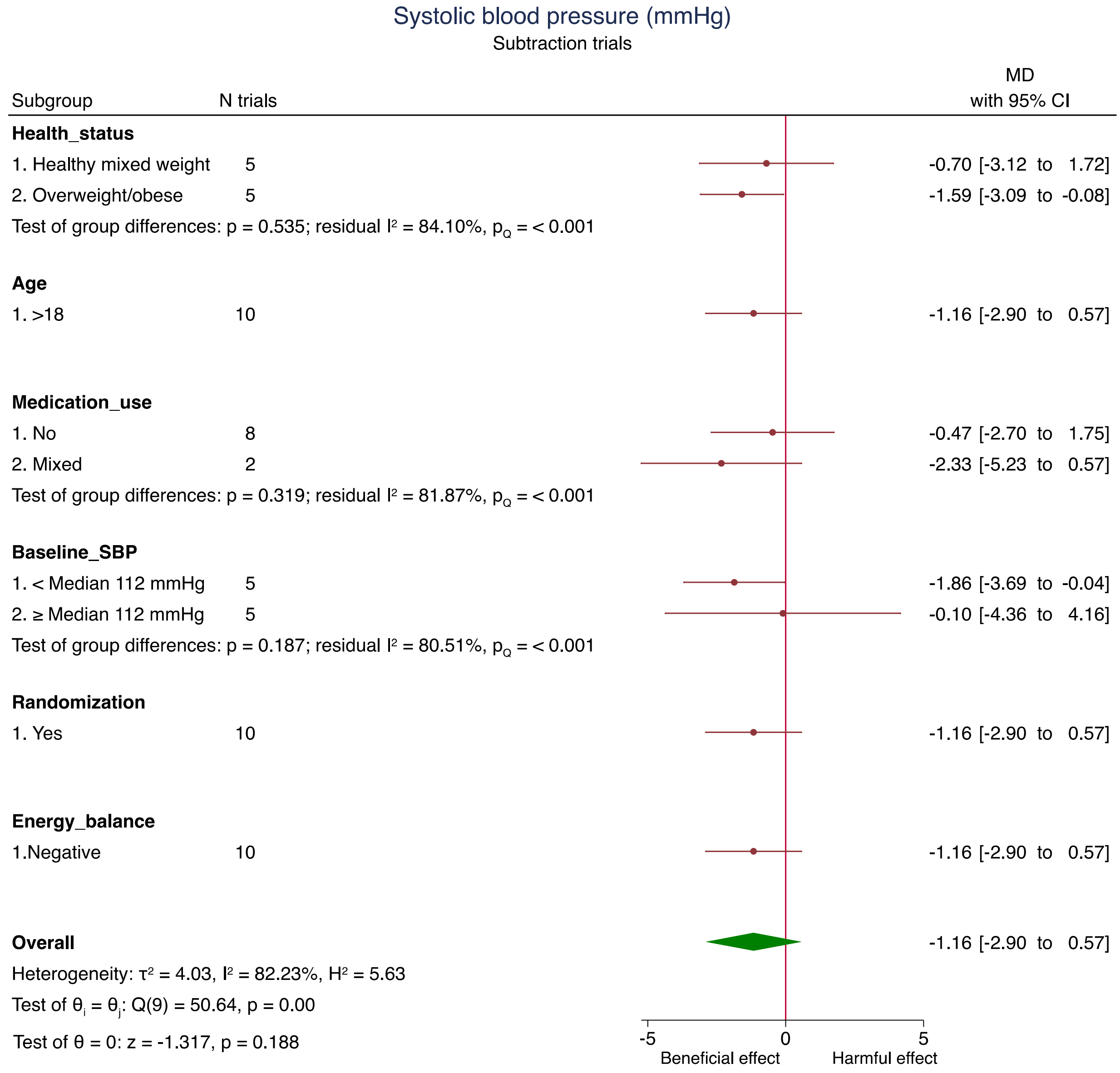

The green diamond represents the pooled estimate for the overall primary analysis of food sources of fructose-containing sugars on SBP. Within subgroup mean differences are the pooled effect estimates represented by a red circle. 95% confidence intervals are represented by the line through the circle. Data are expressed as mean differences with 95% confidence intervals using the generic inverse-variance method and random effects DerSimonian-Laird model. Inter-study heterogeneity was assessed using the Cochrane Q statistic and quantified using the I^2 statistic, with significance set at p<0.100 and $I^2 \geq 50\%$ considered to be evidence of substantial heterogeneity. p<0.050 indicates that the effect size differed between levels of the subgroup. CI=confidence interval; het=heterogeneity.

S51 Fig (part 2 of 3). Subgroup analyses for the effect of important food sources of fructose-containing sugars on SBP (mmHg) in subtraction trials.

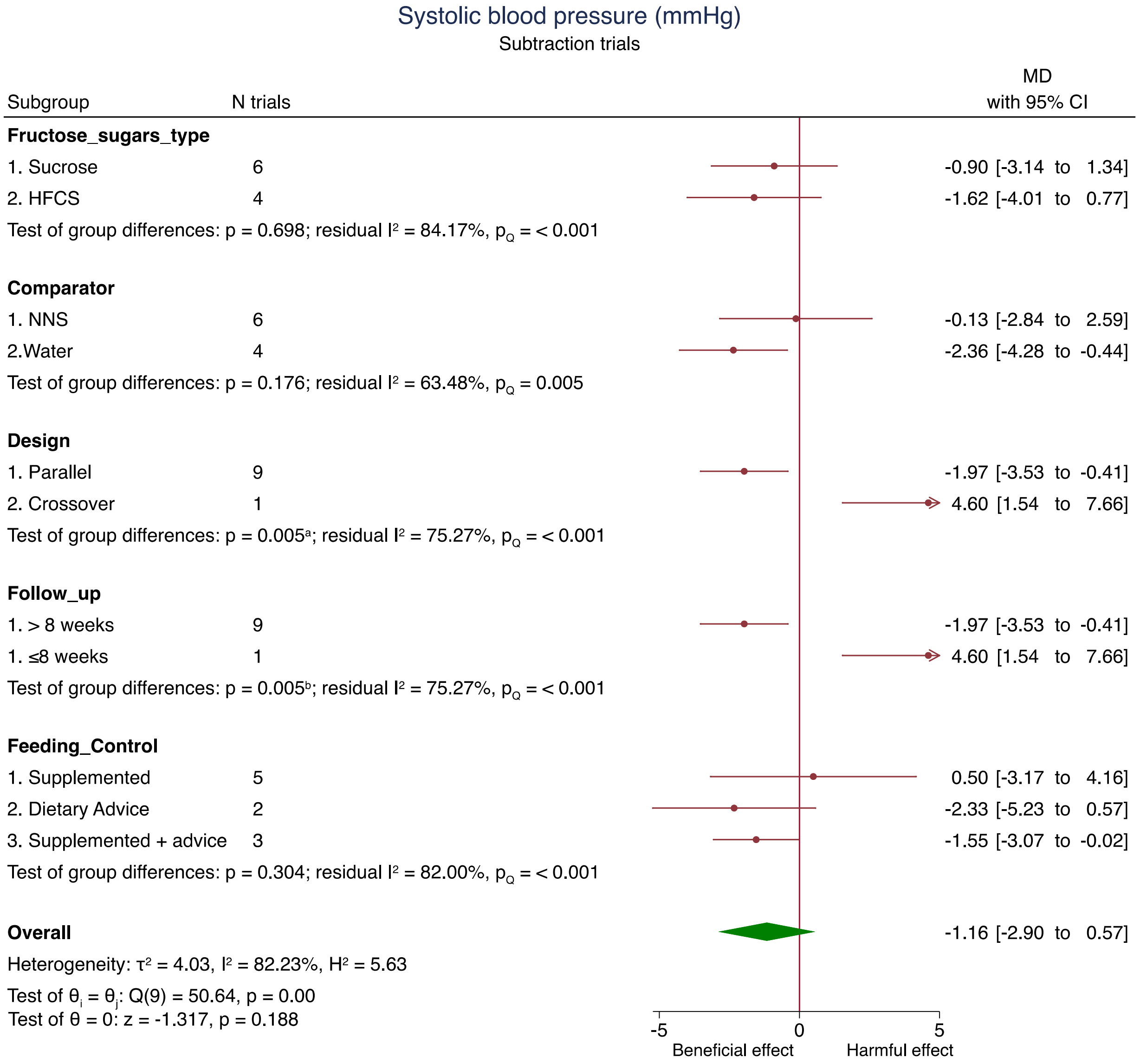

[a]Pairwise between-subgroup mean differences (95% confidence intervals) for design were as follows: (2 vs. 1) 6.57 mmHg (1.95, 11.2 mmHg).

[b]Pairwise between-subgroup mean differences (95% confidence intervals) for follow-up were as follows: (2 vs. 1) -6.57 mmHg (-11.2, -1.95mmHg).

The green diamond represents the pooled estimate for the overall primary analysis of food sources of fructose-containing sugars on SBP. Within subgroup mean differences are the pooled effect estimates represented by a red circle. 95% confidence intervals are represented by the line through the circle. Data are expressed as mean differences with 95% confidence intervals using the generic inverse-variance method and random effects DerSimonian-Laird model. Inter-study heterogeneity was assessed using the Cochrane Q statistic and quantified using the I^2 statistic, with significance set at $p<0.100$ and $I^2 \geq 50\%$ considered to be evidence of substantial heterogeneity. $p<0.050$ indicates that the effect size differed between levels of the subgroup.
CI=confidence interval; HFCS=high fructose corn syrup.

S51 Fig (part 3 of 3). Subgroup analyses for the effect of important food sources of fructose-containing sugars on SBP (mmHg) in subtraction trials.

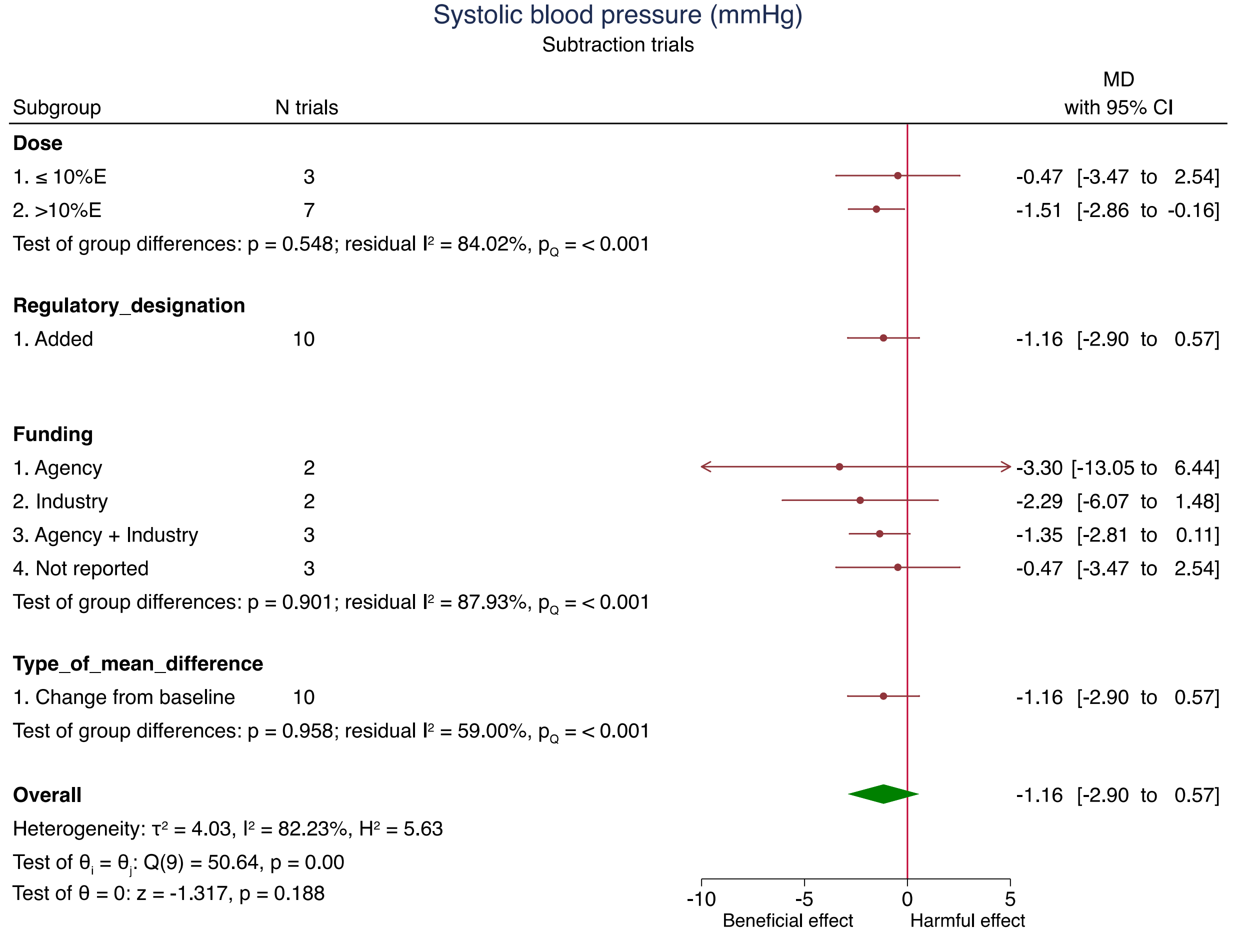

The green diamond represents the pooled estimate for the overall primary analysis of food sources of fructose-containing sugars on SBP. Within subgroup mean differences are the pooled effect estimates represented by a red circle. 95% confidence intervals are represented by the line through the circle. Data are expressed as mean differences with 95% confidence intervals using the generic inverse-variance method and random effects DerSimonian-Laird model. Inter-study heterogeneity was assessed using the Cochrane Q statistic and quantified using the I^2 statistic, with significance set at p<0.100 and I^2≥50% considered to be evidence of substantial heterogeneity. p<0.050 indicates that the effect size differed between levels of the subgroup.
%E=percent of daily energy intake; CI=confidence interval; het=heterogeneity.

S52 Fig. Risk of bias (using The Cochrane Collaboration Tool) subgroup analysis for the effect of important food sources of fructose-containing sugars on SBP (mmHg) in subtraction trials.

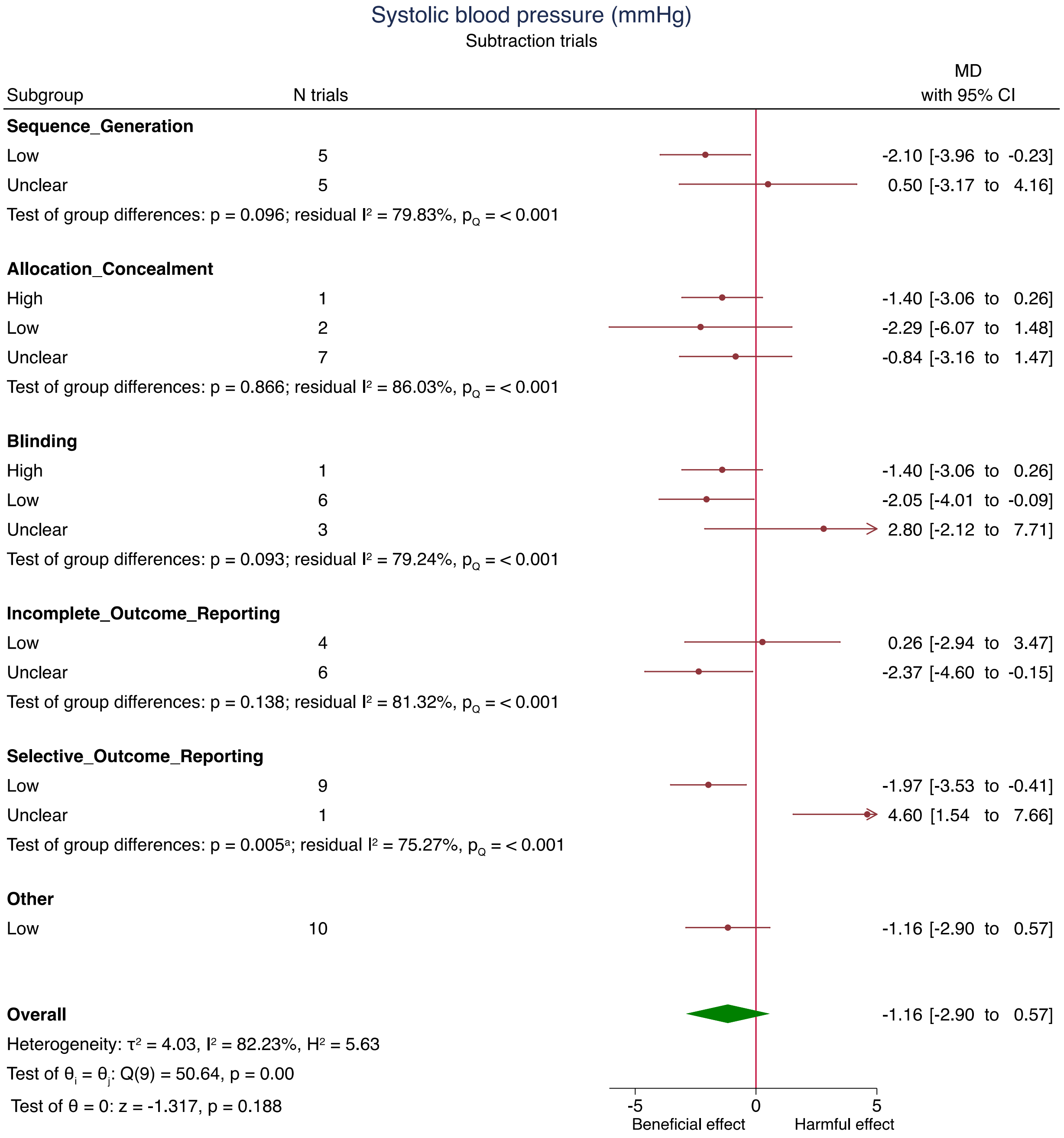

[a]Pairwise between-subgroup mean differences (95% confidence intervals) for selective outcome reporting were as follows: (unclear vs. low) 6.57 mmHg (1.95, 11.2 mmHg).

The green diamond represents the pooled estimate for the overall primary analysis of food sources of fructose-containing sugars on SBP. Within subgroup mean differences are the pooled effect estimates represented by a red circle. 95% confidence intervals are represented by the line through the circle. Data are expressed as mean differences with 95% confidence intervals using the generic inverse-variance method and random effects DerSimonian-Laird model. Inter-study heterogeneity was assessed using the Cochrane Q statistic and quantified

using the I^2 statistic, with significance set at p<0.100 and $I^2 \geq 50\%$ considered to be evidence of substantial heterogeneity. p<0.050 indicates that the effect size differed between levels of the subgroup.
CI=confidence interval; het=heterogeneity.

S53 Fig (part 1 of 3). Subgroup analyses for the effect of important food sources of fructose-containing sugars on DBP (mmHg) in substitution trials.

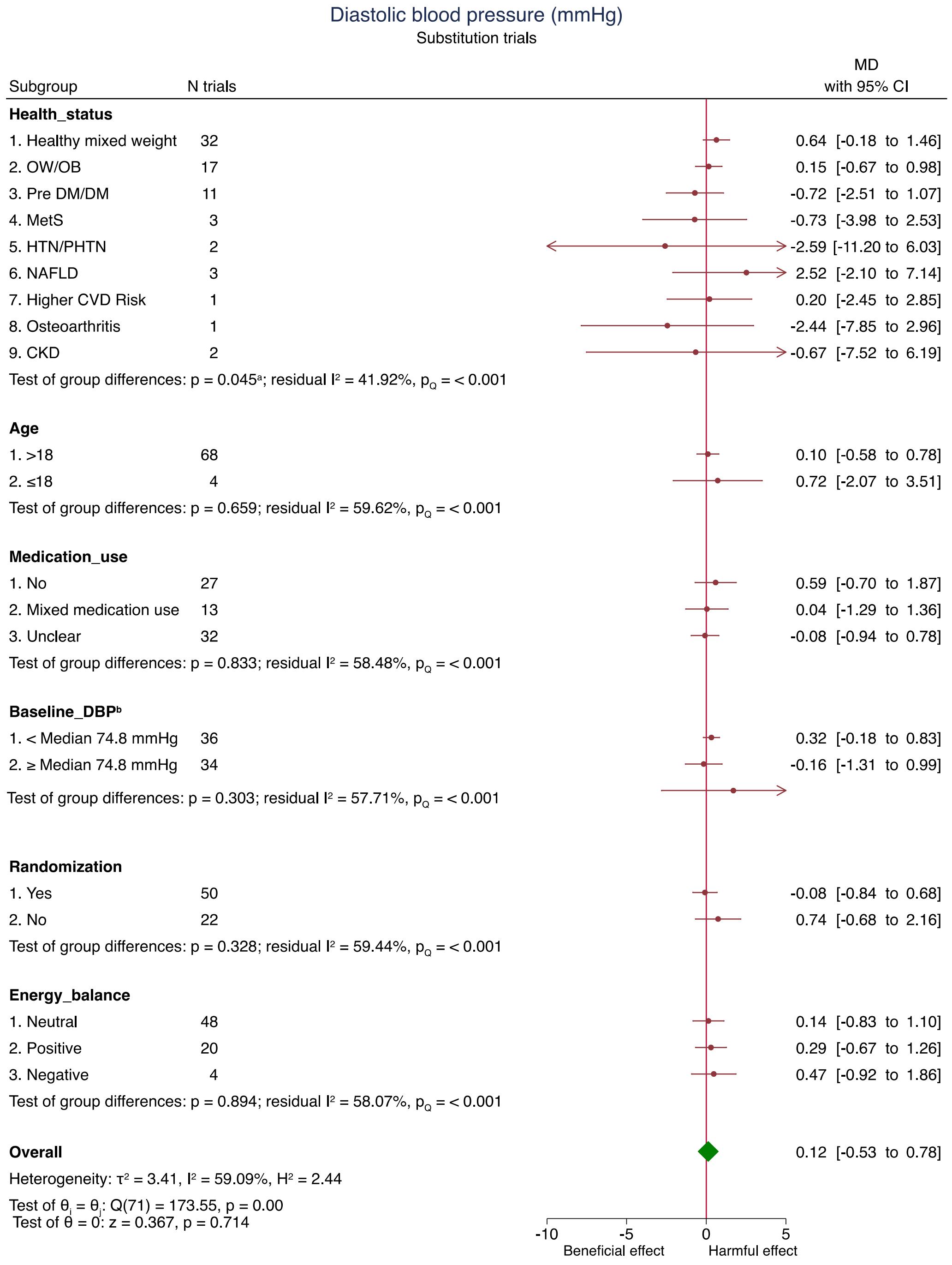

[a]Pairwise between-subgroup mean differences (95% confidence intervals) for health status were as follows: (2 vs. 1) -4.77 mmHg (-1.88, 0.93 mmHg); (3 vs. 1) -1.46 mmHg (-3.33, 0.405 mmHg); (4 vs. 1) -1.3 mmHg (-3.99, 1.4 mmHg); (5 vs. 1) -4.8 mmHg (-7.64, -1.97 mmHg); (6 vs. 1) 2.25 mmHg (-1.58, 6.07 mmHg); (7 vs. 1) -5.25 mmHg (-4.49, 3.44 mmHg); (8 vs. 1) -3.17 mmHg (-9.33, 2.99 mmHg); (9 vs. 1) -1.83 mmHg (-5.49, 1.83 mmHg); (3 vs. 2) -0.984 mmHg (-2.88, 0.912 mmHg); (4 vs. 2) -0.818 mmHg (-3.53, 1.9 mmHg); (5 vs. 2) -4.32 mmHg (-7.18, -1.47

mmHg); (6 vs. 2) 2.72 mmHg (-1.12, 6.57 mmHg); (7 vs. 2) -0.479 mmHg (-4.03, 3.93 mmHg); (8 vs. 2) -2.69 mmHg (-8.86, 3.48 mmHg); (9 vs. 2) -1.36 mmHg (-5.03, 2.32 mmHg); (4 vs. 3) 0.166 mmHg (-2.81, 3.14 mmHg); (5 vs. 3) -3.34 mmHg (-6.45, -0.232 mmHg); (6 vs. 3) 3.71 mmHg (-0.325, 7.74 mmHg); (7 vs. 3) 0.936 mmHg (-3.23, 5.1 mmHg); (8 vs. 3) -1.71 mmHg (-7.99, 5.48 mmHg); (9 vs. 3) -0.372 mmHg (-4.24, 3.5 mmHg); (5 vs. 4) -3.51 mmHg (-7.17, 0.159 mmHg); (6 vs. 4) 3.54 mmHg (-0.934, 8.02 mmHg); (7 vs. 4) 0.77 mmHg (-3.82, 5.37 mmHg); (8 vs. 4) -1.87 mmHg (-8.45, 4.71 mmHg); (9 vs. 4) -0.537 mmHg (-4.87, 3.79 mmHg); (6 vs. 5) 7.05 mmHg (2.48, 8.96 mmHg); (6 vs. 5) 7.05 mmHg (2.48, 11.6 mmHg); (7 vs. 5) 4.28 mmHg (-0.404, 8.96 mmHg); (8 vs. 5) 1.63 mmHg (-5.01, 8.27 mmHg); (9 vs. 5) 1.63 mmHg (-5.01, 8.27 mmHg); (7 vs. 6) -2.77 mmHg (-8.11, 2.57 mmHg); (8 vs. 6) -5.41 mmHg (-12.5, 1.71 mmHg); (9 vs. 6) -4.08 mmHg (-9.19, 1.04 mmHg); (8 vs. 7) -2.64 mmHg (-9.84, 4.55 mmHg); (9 vs. 7) -1.31 mmHg (-6.53, 3.91 mmHg); (9 vs. 8) 1.34 mmHg (-5.7, 8.37 mmHg)

[b]N=1 trial missing for baseline DBP data.

The green diamond represents the pooled estimate for the overall primary analysis of food sources of fructose-containing sugars on DBP. Within subgroup mean differences are the pooled effect estimates represented by a red circle. 95% confidence intervals are represented by the line through the circle. Data are expressed as mean differences with 95% confidence intervals using the generic inverse-variance method and random effects DerSimonian-Laird model. Inter-study heterogeneity was assessed using the Cochrane Q statistic and quantified using the I^2 statistic, with significance set at $p<0.100$ and $I^2 \geq 50\%$ considered to be evidence of substantial heterogeneity. $p<0.050$ indicates that the effect size differed between levels of the subgroup.

CI=confidence interval; CKD=chronic kidney disease; DM=diabetes mellitus HTN=hypertensive; OW/OB=overweight/obese; MetS=metabolic syndrome; NAFLD= non-alcoholic fatty liver disease; PHTN=pre-hypertensive

S53 Fig (part 2 of 3). Subgroup analyses for the effect of important food sources of fructose-containing sugars on DBP (mmHg) in substitution trials.

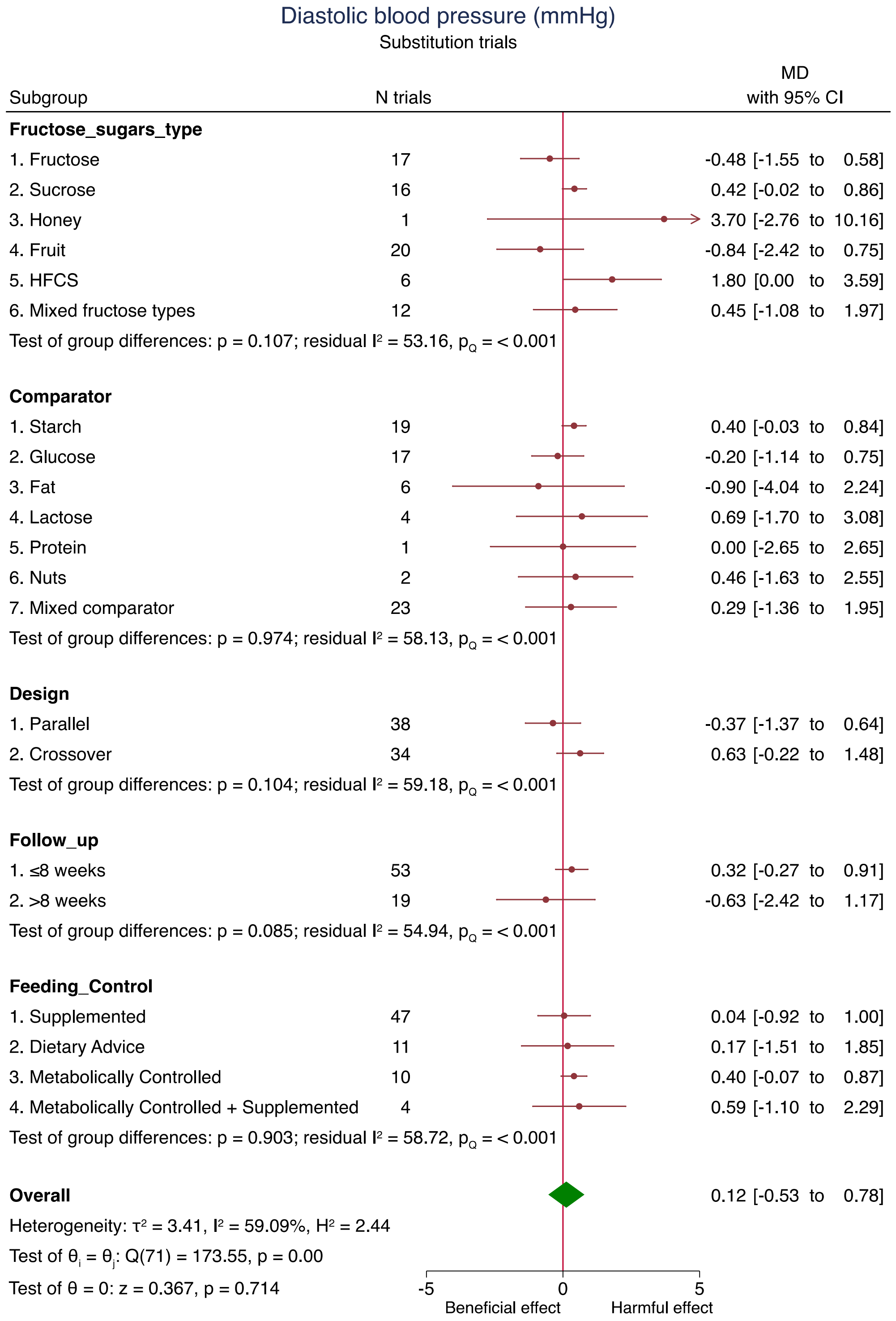

The green diamond represents the pooled estimate for the overall primary analysis of food sources of fructose-containing sugars DBP. Within subgroup mean differences are the pooled effect estimates represented by a red circle. 95% confidence intervals are represented by the line through the circle. Data are expressed as mean differences with 95% confidence intervals using the generic inverse-variance method and random effects DerSimonian-Laird model. Inter-study heterogeneity was assessed using the Cochrane Q statistic and quantified using the I^2 statistic, with significance set at $p<0.100$ and $I^2 \geq 50\%$ considered to be evidence of substantial heterogeneity. $p<0.050$ indicates that the effect size differed between levels of the subgroup.
CI=confidence interval; HFCS=high fructose corn syrup

S53 Fig (part 3 of 3). Subgroup analyses for the effect of important food sources of fructose-containing sugars on DBP (mmHg) in substitution trials.

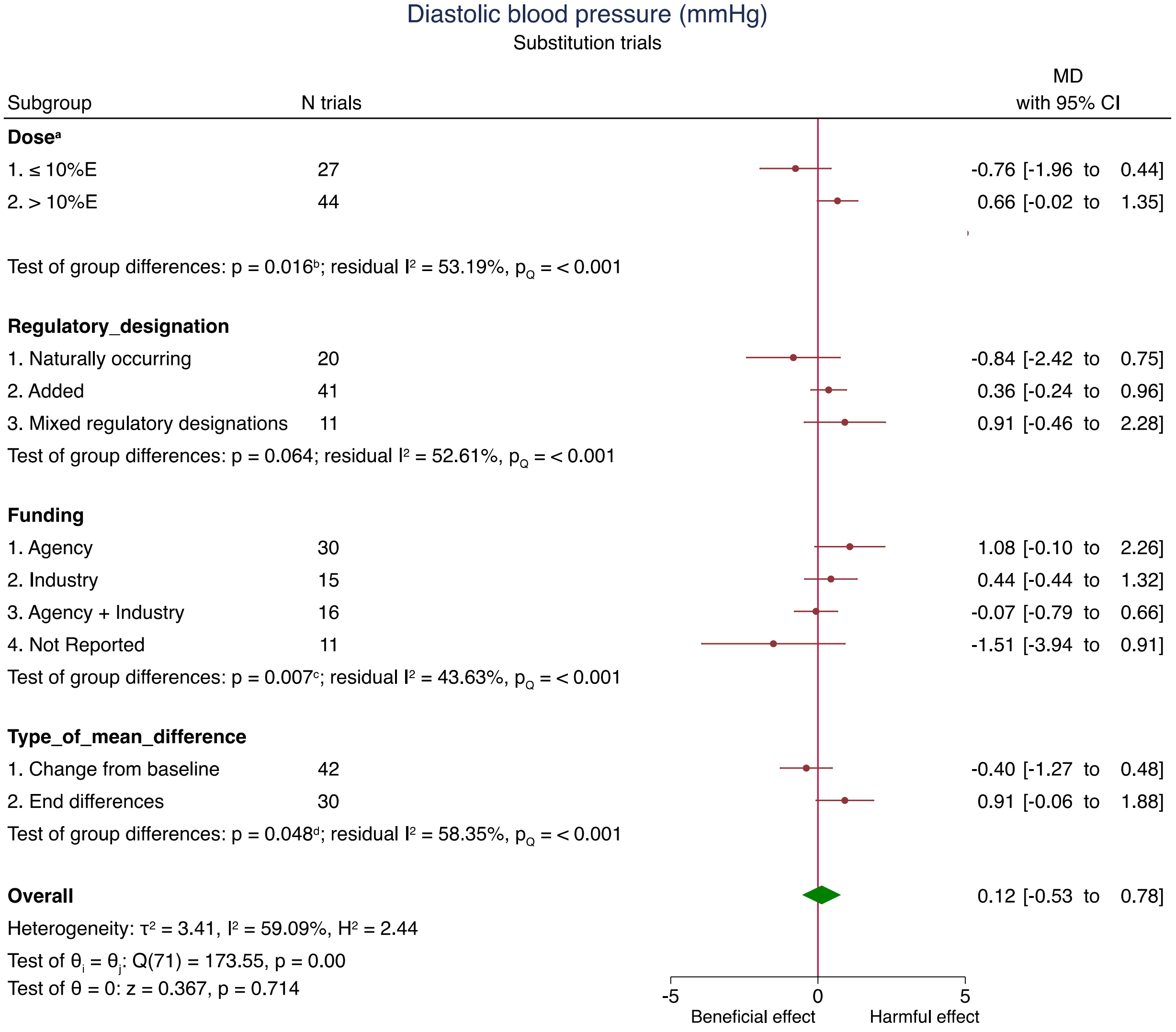

[a]N=1 trial missing data for dose. Pairwise between-subgroup mean differences (95% confidence intervals) for dose were as follows: (2 vs. 1) 1.66 mmHg (0.386, 2.94 mmHg)

[b]Pairwise between-subgroup mean differences (95% confidence intervals) for funding were as follows: (2 vs. 1) -0.763 mmHg (-2.38, 0.855 mmHg); (3 vs. 1) -1.05 mmHg (-2.53, 0.435 mmHg); (4 vs. 1) -3.33 (-5.22, -1.45 mmHg); (3 vs. 2) -0.287 mmHg (-1.9. 1.33 mmHg); (4 vs. 2) -2.57 mmHg (-4.56, -0.581 mmHg); (4 vs. 3) -2.28 mmHg (-4.17, -0.402 mmHg)

[c]Pairwise between-subgroup mean differences (95% confidence intervals) for type of mean difference were as follows: (2 vs. 1) 1.37 mmHg (0.0121, 2.74 mmHg)

estimate for the overall primary analysis of food sources of fructose-containing sugars on DBP. Within subgroup mean differences are the pooled effect estimates represented by a red circle. 95% confidence intervals are represented by the line through the circle. Data are expressed as mean differences with 95% confidence intervals using the generic inverse-variance method and random effects DerSimonian-Laird model. Inter-study heterogeneity was assessed using the Cochrane Q statistic and quantified using the I^2 statistic, with significance set at $p<0.100$ and $I^2 \geq 50\%$ considered to be evidence of substantial heterogeneity. $p<0.050$ indicates that the effect size differed between levels of the subgroup.

%E=percent of daily energy intake; CI=confidence interval.

S54 Fig. Risk of bias (using The Cochrane Collaboration Tool) subgroup analysis for the effect of important food sources of fructose-containing sugars on DBP (mmHg) in substitution trials.

Diastolic blood pressure (mmHg)

Substition trials

Subgroup	N trials	MD with 95% CI
**Sequence_Generation**		
High	17	1.51 [0.07 to 2.94]
Low	16	0.23 [-1.82 to 2.29]
Unclear	39	-0.34 [-0.95 to 0.27]
Test of group differences: p = 0.092; residual I^2 = 57.15%, p_Q = < 0.001		
**Allocation_Concealment**		
High	17	1.51 [0.07 to 2.94]
Low	8	-0.35 [-3.82 to 3.11]
Unclear	47	-0.11 [-0.70 to 0.48]
Test of group differences: p = 0.041[a]; residual I^2 = 51.13%, p_Q = < 0.001		
**Blinding**		
High	3	0.40 [-4.68 to 5.48]
Low	47	0.68 [-0.14 to 1.51]
Unclear	22	-1.21 [-2.14 to -0.27]
Test of group differences: p = 0.058; residual I^2 = 57.37%, p_Q = < 0.001		
**Incomplete_Outcome_Reporting**		
High	1	-0.30 [-2.80 to 2.20]
Low	31	0.40 [-0.51 to 1.31]
Unclear	40	-0.06 [-1.01 to 0.88]
Test of group differences: p = 0.678; residual I^2 = 60.20%, p_Q = < 0.001		
**Selective_Outcome_Reporting**		
High	5	-1.21 [-2.36 to -0.06]
Low	41	0.70 [0.04 to 1.37]
Unclear	26	-0.78 [-2.30 to 0.75]
Test of group differences: p = 0.022[b]; residual I^2 = 52.38%, p_Q = < 0.001		
**Other (carry-over effects)**		
High	16	2.08 [0.48 to 3.69]
Low	56	-0.29 [-0.99 to 0.42]
Test of group differences: p = 0.007[c]; residual I^2 = 56.97%, p_Q = < 0.001		
**Overall**		0.12 [-0.53 to 0.78]

Heterogeneity: τ^2 = 3.41, I^2 = 59.09%, H^2 = 2.44

Test of $\theta_i = \theta_j$: Q(71) = 173.55, p = 0.00

Test of θ = 0: z = 0.367, p = 0.714

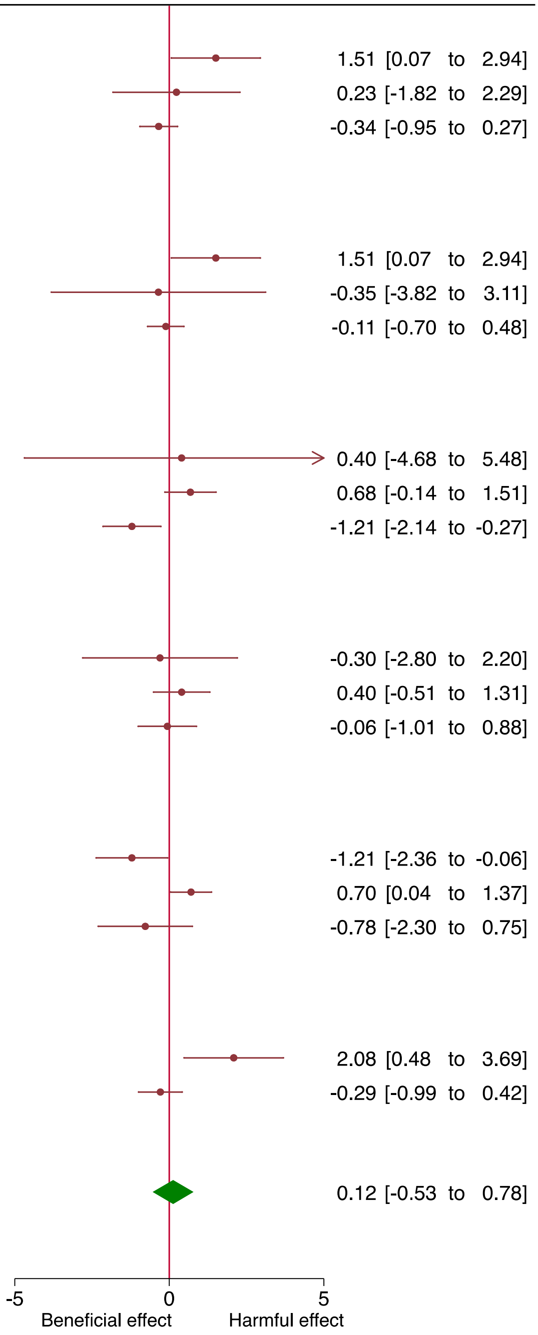

[a]Pairwise between-subgroup mean differences (95% confidence intervals) for allocation concealment reporting were as follows: (low vs. high) -2.74 mmHg, (-5.03, -0.459 mmHg); (unclear vs. high) -1.57 mmHg (-3.11, -0.0321 mmHg); (unclear vs. low) 1.17 mmHg (-0.823, 3.16 mmHg)
[b]Pairwise between-subgroup mean differences (95% confidence intervals) for selective outcome reporting were as follows: (low vs. high) 2.06 mmHg (-0.314, 4.44 mmHg); (unclear vs. high) 0.312 mmHg (-2.18, 2.8 mmHg); (unclear vs. low) -1.75 mmHg (-3.13, -0.37 mmHg)
[c]Pairwise between-subgroup mean differences (95% confidence intervals) for other (carry-over effects) were as follows: (low vs. high) -2.37 mmHg (-4.09, -0.645 mmHg)
The green diamond represents the pooled estimate for the overall primary analysis of food sources of fructose-containing sugars on DBP. Within subgroup mean differences are the pooled effect estimates represented by a red circle. 95% confidence intervals are represented by the line through the circle. Data are expressed as mean differences with 95% confidence intervals using the generic inverse-variance method and random effects DerSimonian-Laird model. Inter-study heterogeneity was assessed using the Cochrane Q statistic and quantified using the I^2 statistic, with significance set at $p<0.100$ and $I^2 \geq 50\%$ considered to be evidence of substantial heterogeneity. $p<0.050$ indicates that the effect size differed between levels of the subgroup.
CI=confidence interval; het=heterogeneity.

S55 Fig (part 1 of 3). Subgroup analyses for the effect of important food sources of fructose-containing sugars on DBP (mmHg) in addition trials.

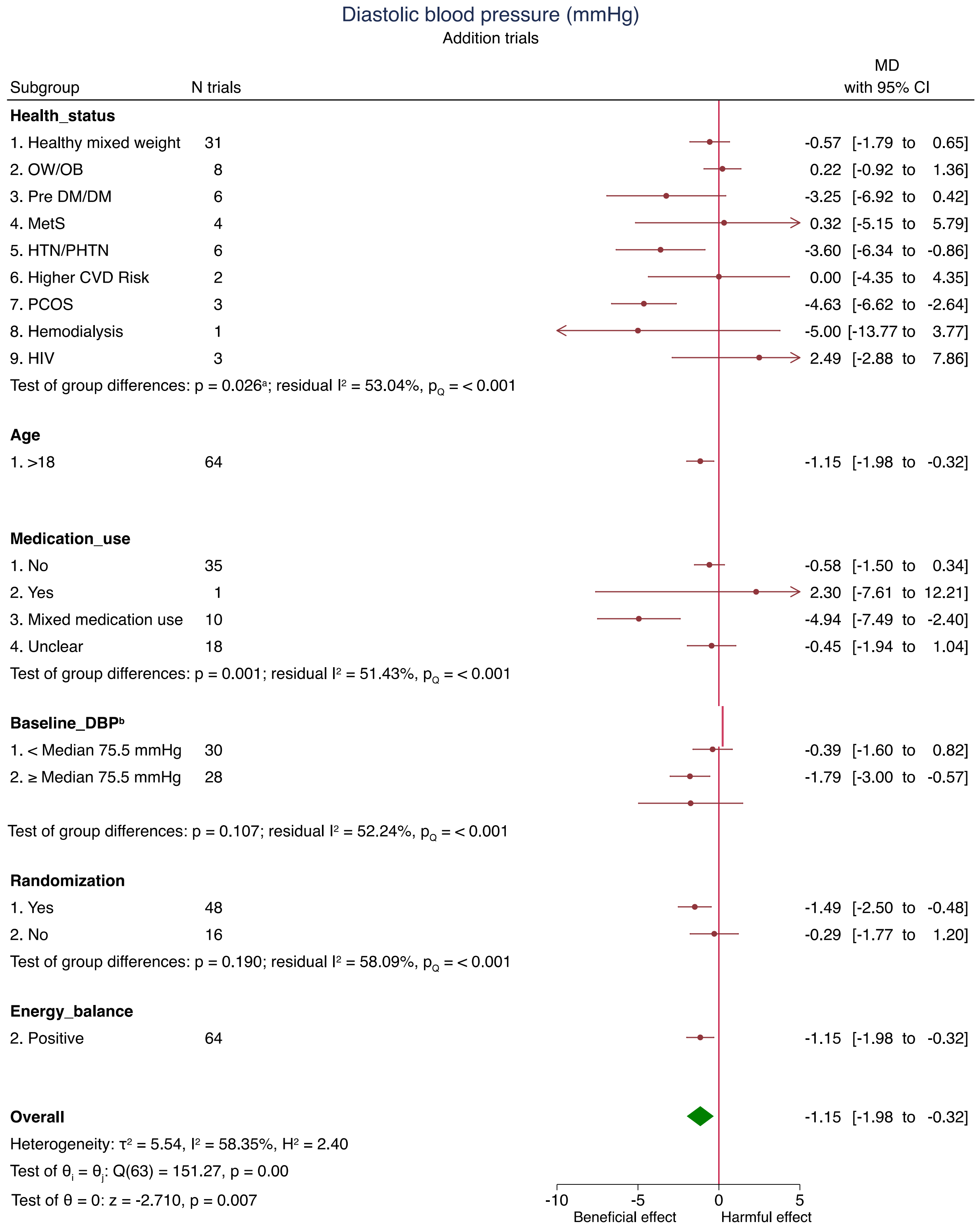

[a]Pairwise between-subgroup mean differences (95% confidence intervals) for health status were as follows: (2 vs. 1) 1.05 mmHg (-1.17, 3.28 mmHg); (3 vs. 1) -2.82 mmHg (-6.06, 0.425 mmHg); (4 vs. 1) 0.585 mmHg (-3.59, 4.76 mmHg); (5 vs. 1) -2.97 mmHg (-5.46, -0.477 mmHg); (6 vs. 1) 0.589 mmHg (-4.84, 6.01 mmHg); (7 vs. 1) -4.04 mmHg (-7.43, -0.66 mmHg); (8 vs. 1) -4.41 mmHg (-14.2, 5.39 mmHg); (9 vs. 1) 3.08 mmHg (-2.93, 9.09 mmHg); (3 vs. 2) -3.87 mmHg (-7.46, -0.284 mmHg); (4 vs. 2) -0.486 mmHg (-4.92, 3.98 mmHg); (5 vs. 2) -4.02 mmHg (-6.95, -1.1 mmHg); (5 vs. 2) -4.02 mmHg (-6.95, -1.1 mmHg); (6 vs. 2) -0.464 mmHg (-8.81, -1.38 mmHg); (7 vs. 2) -5.1 mmHg (-8.81, -1.38 mmHg); (8 vs. 2) -5.46 mmHg (-15.4, 4.45 mmHg); (9 vs. 2) 2.02 mmHg (-4.18, 8.23 mmHg); (4 vs. 3) 3.4 mmHg (-1.63, 8.44 mmHg); (5 vs. 3) -0.149 mmHg (-3.91, 3.61 mmHg); (6 vs. 3) 3.41 mmHg (-3.7, 9.52 mmHg); (7 vs. 3) -1.22 mmHg (-5.63, 3.18 mmHg); (8 vs. 3) -1.59 mmHg (-11.8, 8.6 mmHg); (9 vs. 3) 5.9 mmHg (-0.738, 12.5 mmHg); (5 vs. 4) -3.55 mmHg (-8.14, 1.03 mmHg); (6 vs. 4) 0.004 mmHg (-6.65, 6.66 mmHg); (7 vs. 4) -4.63 mmHg (-9.76, 0.498 mmHg); (8 vs. 4) -5 mmHg (-15.5, 5.53 mmHg); (9 vs. 4) 2.49 (-4.64, 9.63 mmHg); (6 vs. 5) 3.56 mmHg (-2.19, 9.31 mmHg); (7 vs. 5) -1.08 mmHg (-4.96, 2.8 mmHg); (8 vs. 5) -1.44 mmHg (-11.4, 8.54 mmHg); (9 vs. 5) 6.05 mmHg (-0.256, 12.3 mmHg); (7 vs. 6) -4.63 mmHg (-10.8, 1.55 mmHg); (8 vs. 6) -5 mmHg (-16.1, 6.08 mmHg); (9 vs. 6) 2.49 mmHg (-5.44, 10.4 mmHg); (8 vs. 7) -0.367 mmHg (-10.6, 9.87 mmHg); (9 vs. 7) 7.12 mmHg (0.417, 13.8 mmHg); (9 vs. 8) 7.49 mmHg (-3.89, 18.9 mmHg)
[b]Pairwise between-subgroup mean differences (95% confidence intervals) for medication use were as follows: (2 vs. 1) 2.84 mmHg (-7.93, 13.6 mmHg); (3 vs. 1) -4.32 mmHg (-6.57, -2.06 mmHg); (4 vs. 1) 0.0341 mmHg (-1.73, 1.8 mmHg); (3 vs. 2) -7.16 mmHg (-18.1, 3.74 mmHg); (4 vs. 2) -2.8 mmHg (-13.6, 8 mmHg); (4 vs. 3) 4.35 mmHg (1.92, 6.78 mmHg).
[c]N=6 trials missing baseline DBP data.
The green diamond represents the pooled estimate for the overall primary analysis of food sources of fructose-containing sugars on DBP. Within subgroup mean differences are the pooled effect estimates represented by a red circle. 95% confidence intervals are represented by the line through the circle. Data are expressed as mean differences with 95% confidence intervals using the generic inverse-variance method and random effects DerSimonian-Laird model. Inter-study heterogeneity was assessed using the Cochrane Q statistic and quantified using the I^2 statistic, with significance set at $p<0.100$ and $I^2 \geq 50\%$ considered to be evidence of substantial heterogeneity. $p<0.050$ indicates that the effect size differed between levels of the subgroup.
CI=confidence interval; CVD=cardiovascular disease, DM=diabetes mellitus; HIV=human immunodeficiency virus, HTN=hypertensive; MetS=metabolic syndrome; OW/OB=overweight/obese; PCOS=poly-cystic ovarian syndrome, PHTN=pre-hypertensive

S55 Fig (part 2 of 3). Subgroup analyses for the effect of important food sources of fructose-containing sugars on DBP (mmHg) in addition trials.

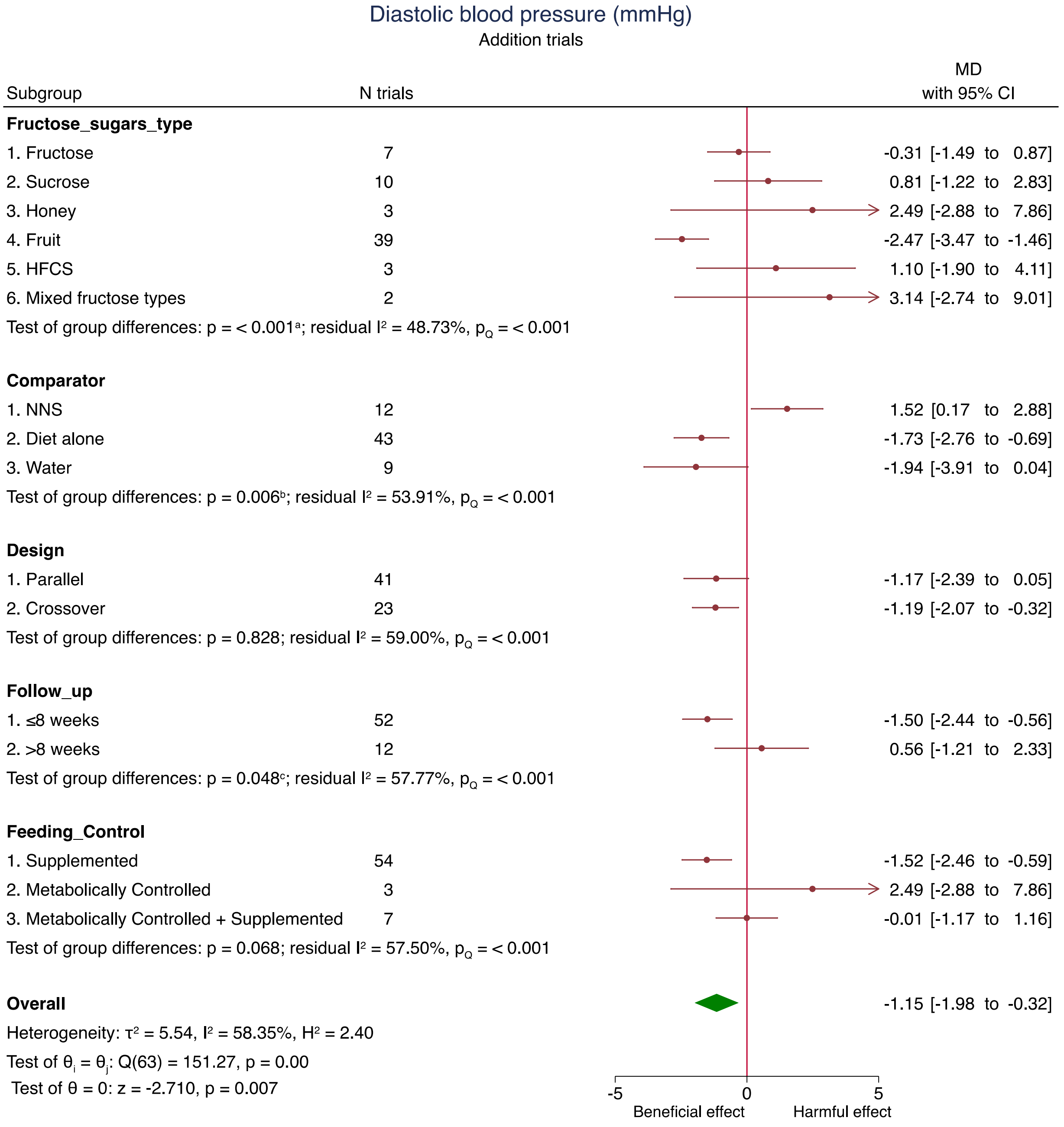

[a]Pairwise between-subgroup mean differences (95% confidence intervals) for fructose sugars type were as follows: (2 vs. 1) 0.408 mmHg (-2.66, 3.4 mmHg); (3 vs. 1) 2.1 mmHg (-4.19, 8.3 mmHg); (4 vs. 1) -2.86 mmHg (-5.39, -0.34 mmHg); (5 vs. 1) 0.701 mmHg (-3.74, 5.1 mmHg); (6 vs. 1) 2.44 mmHg (-1.72, 6 mmHg); (3 vs. 2) 1.69 mmHg (-4.48, 7.8 mmHg); (4 vs. 2) -3.27 mmHg (-5.48, -1 mmHg); (5 vs. 2) 0.293 mmHg (-3.98, 4.5 mmHg); (6 vs. 2) 2.03 mmHg (-1.94, 6.0 mmHg); (4 vs. 3) -4.96 mmHg (-10.9, 0.95 mmHg); (5 vs. 3) -1.4 mmHg (-8.35, 5.5 mmHg); (6 vs. 3) 0.344

mmHg (-6.43, 7.1 mmHg); (5 vs. 4) 3.57 mmHg (-0.332, 7.4 mmHg); (6 vs. 4) 5.31 mmHg (1.73, 8.8 mmHg); (6 vs. 5) 1.74 mmHg (-3.37, 6.8 mmHg)

[b]Pairwise between-subgroup mean differences (95% confidence intervals) for comparator were as follows: (2 vs. 1) -3.34 mmHg (-5.48, -1.21 mmHg); (3 vs. 1) -3.54 mmHg (-6.29, -0.793 mmHg); (3 vs. 2) -0.197 mmHg (-2.42, 2.02 mmHg.

[c]Pairwise between-subgroup mean differences (95% confidence intervals) for follow-up were as follows: (2 vs. 1) 2.36 mmHg (0.0212, 4.69 mmHg).

The green diamond represents the pooled estimate for the overall primary analysis of food sources of fructose-containing sugars on DBP. Within subgroup mean differences are the pooled effect estimates represented by a red circle. 95% confidence intervals are represented by the line through the circle. Data are expressed as mean differences with 95% confidence intervals using the generic inverse-variance method and random effects DerSimonian-Laird model. Inter-study heterogeneity was assessed using the Cochrane Q statistic and quantified using the I^2 statistic, with significance set at $p<0.100$ and $I^2 \geq 50\%$ considered to be evidence of substantial heterogeneity. $p<0.050$ indicates that the effect size differed between levels of the subgroup.

CI=confidence interval; HFCS=high fructose corn syrup; NNS=non-nutritive sweetener

S55 Fig (part 3 of 3). Subgroup analyses for the effect of important food sources of fructose-containing sugars on DBP (mmHg) in addition trials.

Diastolic blood pressure (mmHg)

Addition trials

Subgroup	N trials	MD with 95% CI
**Dose**		
1. ≤ 10%E	40	-2.42 [-3.46 to -1.37]
2. > 10%E	24	0.76 [-0.31 to 1.82]
Test of group differences: $p = < 0.001^{a}$; residual $I^2 = 51.02\%$, $p_Q = < 0.001$		
**Regulatory_designation**		
1. Naturally occurring	39	-2.47 [-3.47 to -1.46]
2. Added	24	1.07 [-0.10 to 2.24]
3. Mixed regulatory designations	1	0.30 [-1.90 to 2.50]
Test of group differences: $p = < 0.001^{b}$; residual $I^2 = 48.32\%$, $p_Q = < 0.001$		
**Funding**		
1. Agency	40	-1.88 [-3.04 to -0.73]
2. Industry	3	-1.26 [-3.74 to 1.21]
3. Agency + Industry	19	0.25 [-1.02 to 1.52]
4. Not Reported	2	-0.29 [-1.88 to 1.30]
Test of group differences: $p = 0.110$; residual $I^2 = 55.18\%$, $p_Q = < 0.001$		
**Type_of_mean_difference**		
1. Change from baseline	49	-1.12 [-2.21 to -0.04]
2. End differences	15	-1.19 [-2.08 to -0.30]
Test of group differences: $p = 0.958$; residual $I^2 = 59.00\%$, $p_Q = < 0.001$		
**Overall**		-1.15 [-1.98 to -0.32]
Heterogeneity: $\tau^2 = 5.54$, $I^2 = 58.35\%$, $H^2 = 2.40$		
Test of $\theta_i = \theta_j$: $Q(63) = 151.27$, $p = 0.00$		
Test of $\theta = 0$: $z = -2.710$, $p = 0.007$		

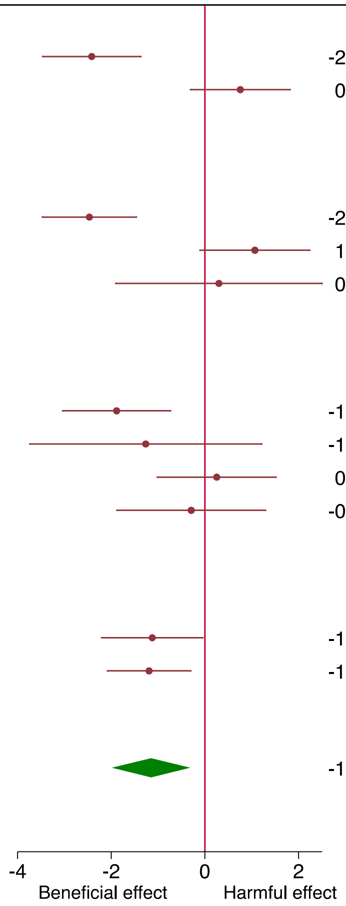

[a]Pairwise between-subgroup mean differences (95% confidence intervals) for dose were as follows: (2 vs. 1) 3.31 mmHg (1.71, 4.9 mmHg)

[b]Pairwise between-subgroup mean differences (95% confidence intervals) for regulatory designation were as follows: (2 vs. 1) 3.61 mmHg (1.98, 5.23 mmHg); (3 vs. 1) 2.77 mmHg (-1.8, 7.35 mmHg); (3 vs. 2) -0.831 mmHg (-5.5, 3.84 mmHg).

The green diamond represents the pooled estimate for the overall primary analysis of food sources of fructose-containing sugars on DBP. Within subgroup mean differences are the pooled effect estimates represented by a red circle. 95% confidence intervals are represented by the line through the circle. Data are expressed as mean differences with 95% confidence intervals using the generic inverse-variance method and random effects DerSimonian-Laird model. Inter-study heterogeneity was assessed using the Cochrane Q statistic and quantified using the I^2 statistic, with significance set at $p<0.100$ and $I^2 \geq 50\%$ considered to be evidence of substantial heterogeneity. $p<0.050$ indicates that the effect size differed between levels of the subgroup.

%E=percent of daily energy intake; CI=confidence interval.

S56 Fig. Risk of bias (using The Cochrane Collaboration Tool) subgroup analysis for the effect of important food sources of fructose-containing sugars on DBP (mmHg) in addition trials.

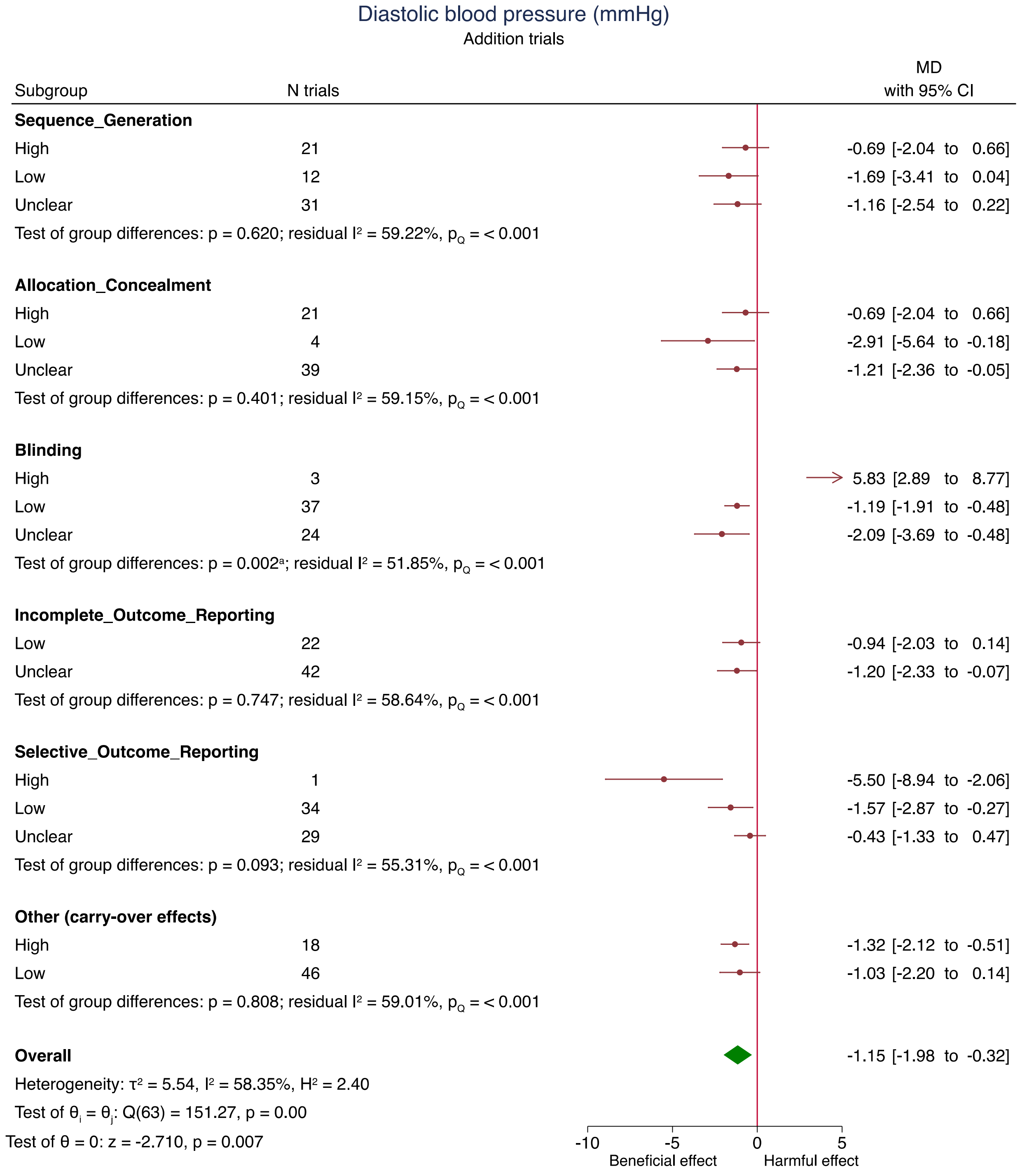

[a]Pairwise between-subgroup mean differences (95% confidence intervals) for blinding were as follows: (low vs. high) -6.61 mmHg (-10.8, -2.44 mmHg); (unclear vs. high) -7.69 mmHg (-11.9, -3.46 mmHg); (unclear vs. low) -1.08 mmHg (-2.7, 0.539 mmHg).

The green diamond represents the pooled estimate for the overall primary analysis of food sources of fructose-containing sugars on DBP. Within subgroup mean differences are the pooled effect estimates represented by a red circle. 95% confidence intervals are represented by the line through the circle. Data are expressed as mean differences with 95% confidence intervals using the generic inverse-variance method and random effects DerSimonian-Laird model. Inter-study heterogeneity was assessed using the Cochrane Q statistic and quantified using the I^2 statistic, with significance set at p<0.100 and $I^2 \geq 50\%$ considered to be evidence of substantial heterogeneity. p<0.050 indicates that the effect size differed between levels of the subgroup.
CI=confidence interval;het=heterogeneity.

S57 Fig (part 1 of 3). Subgroup analyses for the effect of important food sources of fructose-containing sugars on DBP (mmHg) in subtraction trials.

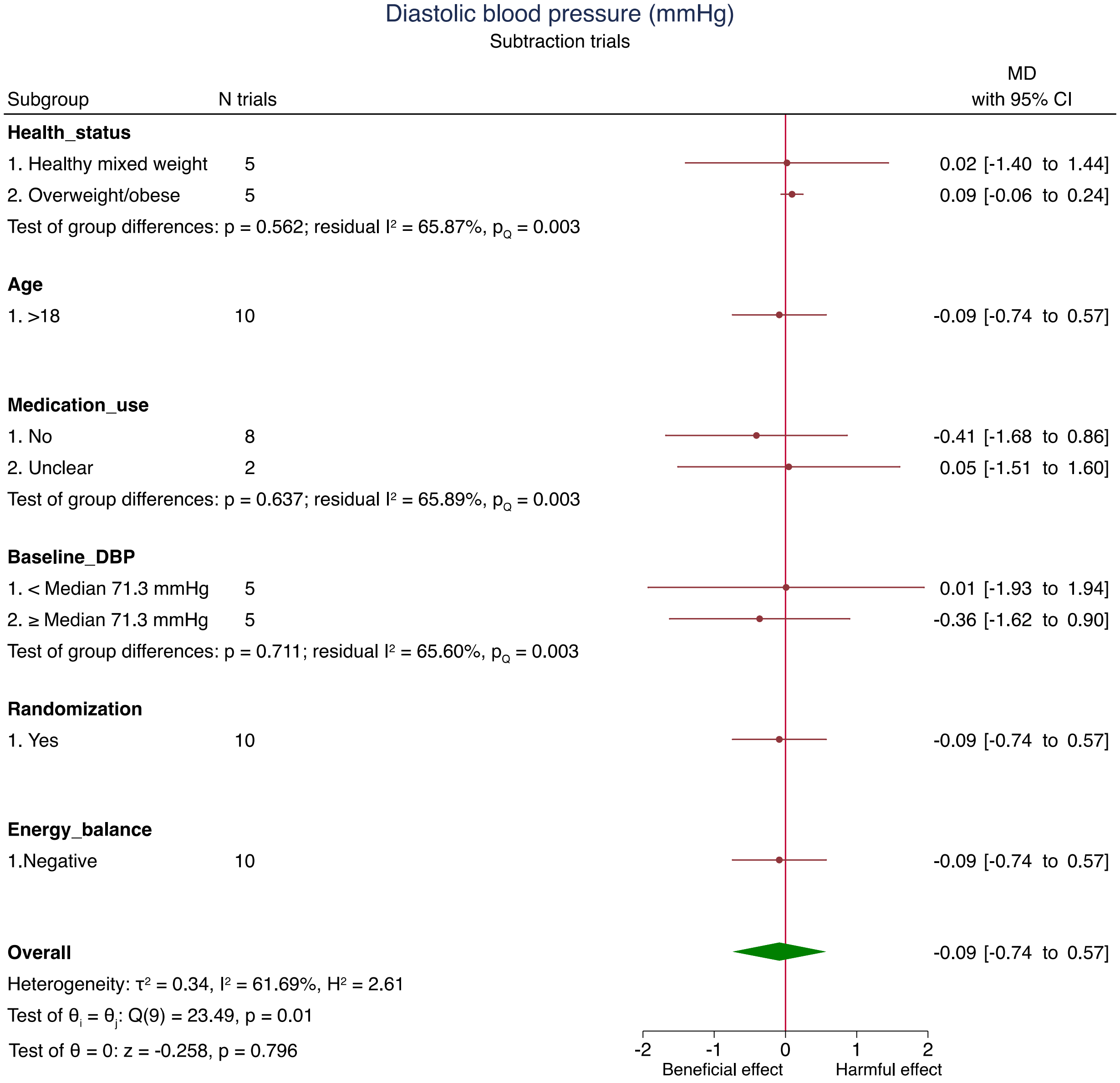

The green diamond represents the pooled estimate for the overall primary analysis of food sources of fructose-containing sugars on DBP. Within subgroup mean differences are the pooled effect estimates represented by a red circle. 95% confidence intervals are represented by the line through the circle. Data are expressed as mean differences with 95% confidence intervals using the generic inverse-variance method and random effects DerSimonian-Laird model. Inter-study heterogeneity was assessed using the Cochrane Q statistic and quantified using the I^2 statistic, with significance set at $p<0.100$ and $I^2 \geq 50\%$ considered to be evidence of substantial heterogeneity. $p<0.050$ indicates that the effect size differed between levels of the subgroup. CI=confidence interval; het=heterogeneity.

S57 Fig (part 2 of 3). Subgroup analyses for the effect of important food sources of fructose-containing sugars on DBP (mmHg) in subtraction trials.

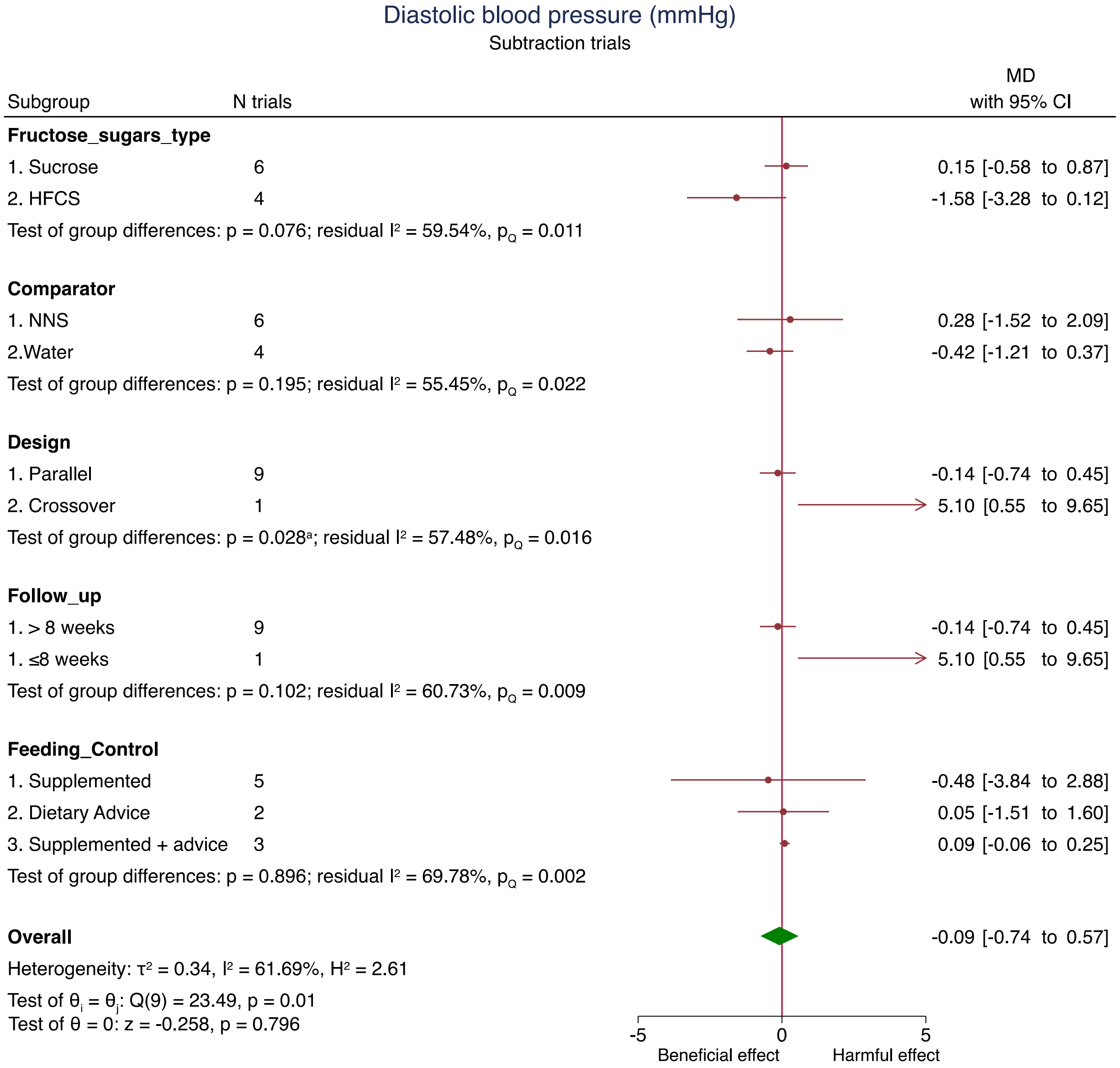

[a]Pairwise between-subgroup mean differences (95% confidence intervals) for design were as follows: (2 vs. 1) 5.24 mmHg (0.553, 9.94 mmHg).

The green diamond represents the pooled estimate for the overall primary analysis of food sources of fructose-containing sugars on DBP. Within subgroup mean differences are the pooled effect estimates represented by a red circle. 95% confidence intervals are represented by the line through the circle. Data are expressed as mean differences with 95% confidence intervals using the generic inverse-variance method and random effects DerSimonian-Laird model. Inter-study heterogeneity was assessed using the Cochrane Q statistic and quantified using the I^2 statistic, with significance set at p<0.100 and I^2≥50% considered to be evidence of substantial heterogeneity. p<0.050 indicates that the effect size differed between levels of the subgroup.
CI=confidence interval; HFCS=high fructose corn syrup.

S57 Fig (part 3 of 3). Subgroup analyses for the effect of important food sources of fructose-containing sugars on DBP (mmHg) in subtraction trials.

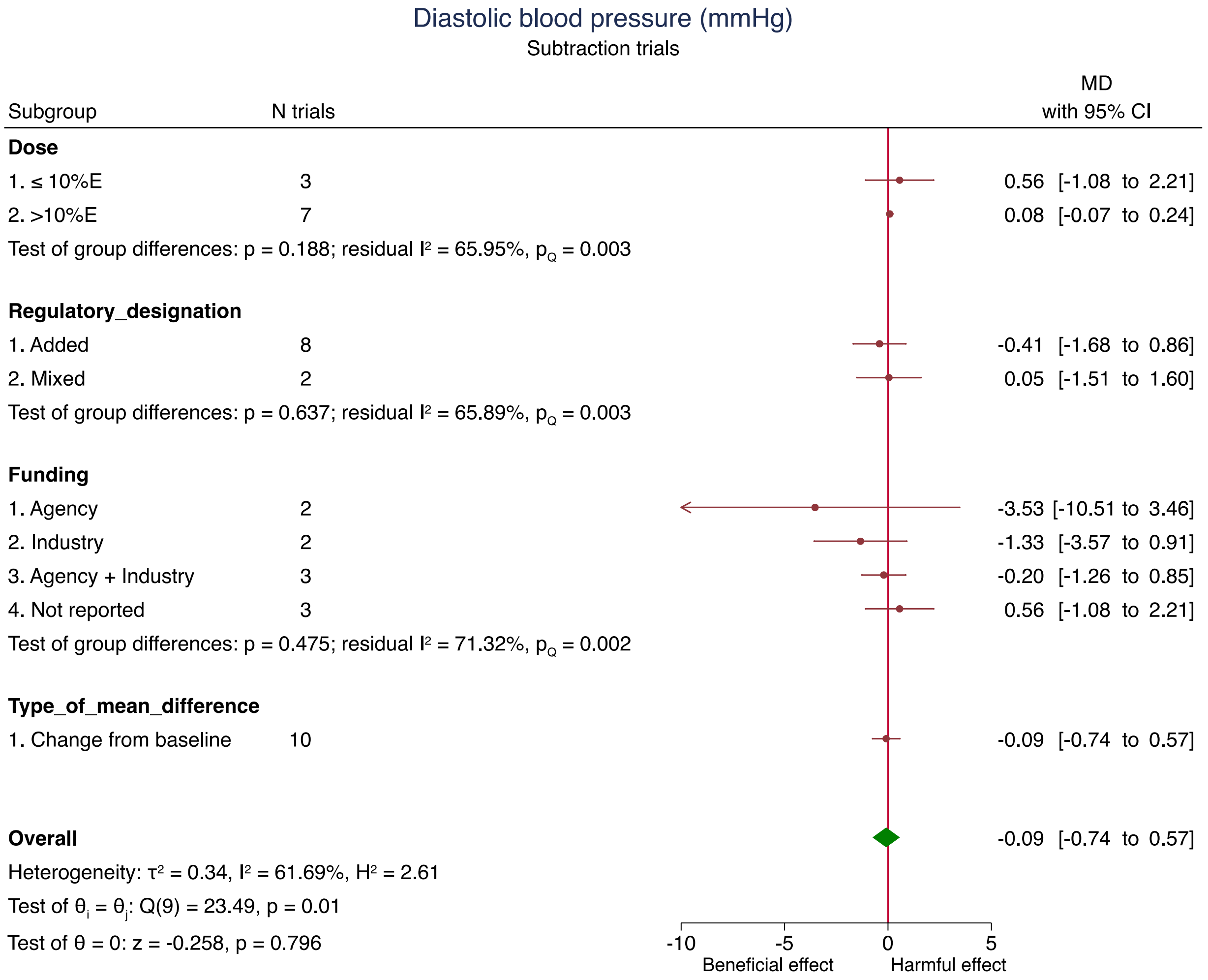

The green diamond represents the pooled estimate for the overall primary analysis of food sources of fructose-containing sugars on DBP. Within subgroup mean differences are the pooled effect estimates represented by a red circle. 95% confidence intervals are represented by the line through the circle. Data are expressed as mean differences with 95% confidence intervals using the generic inverse-variance method and random effects DerSimonian-Laird model. Inter-study heterogeneity was assessed using the Cochrane Q statistic and quantified using the I^2 statistic, with significance set at p<0.100 and $I^2 \geq 50\%$ considered to be evidence of substantial heterogeneity. p<0.050 indicates that the effect size differed between levels of the subgroup.
%E=percent of daily energy intake; CI=confidence interval.

S58 Fig. Risk of bias (using The Cochrane Collaboration Tool) subgroup analysis for the effect of important food sources of fructose-containing sugars on DBP (mmHg) in subtraction trials.

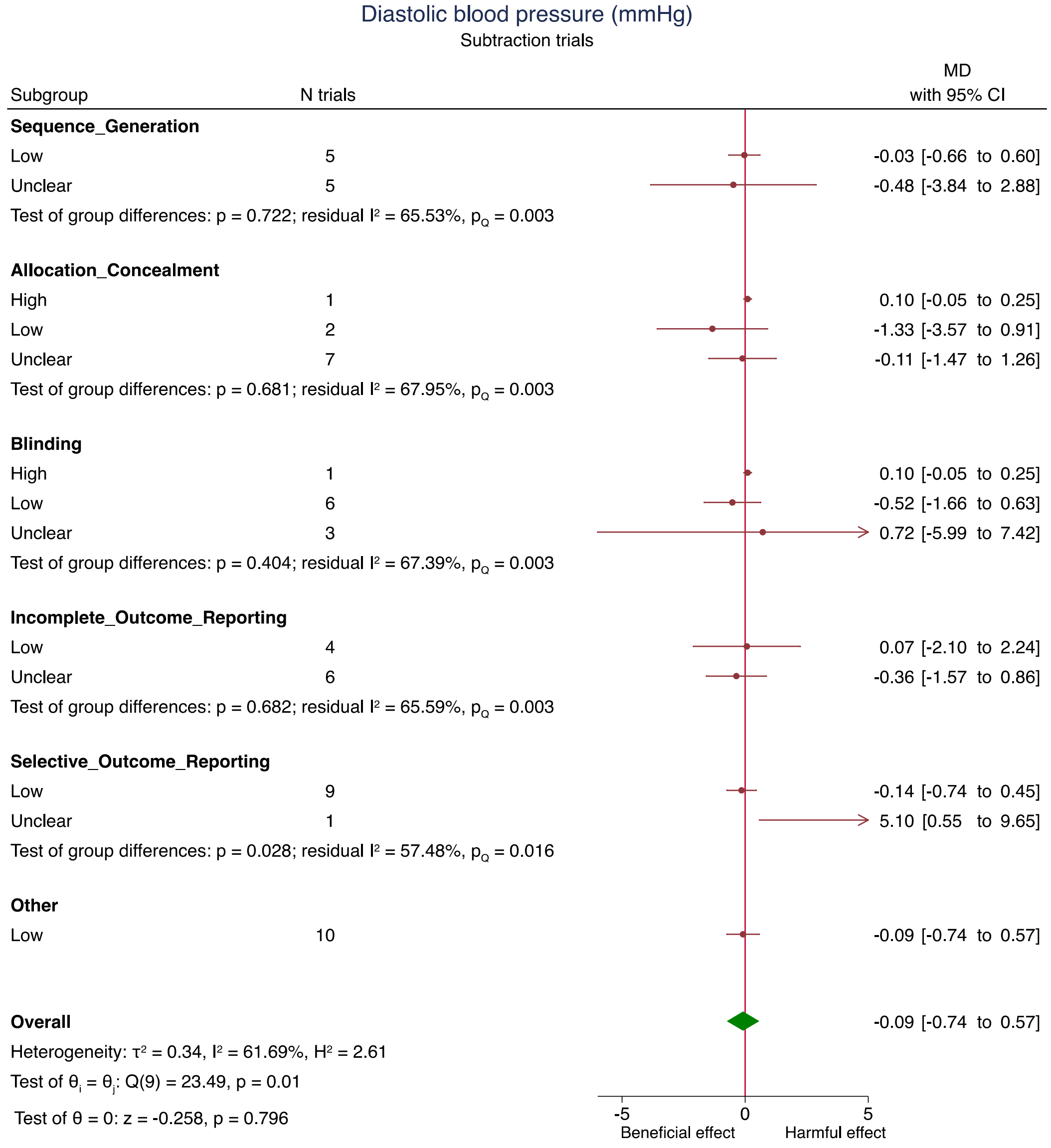

[a]Pairwise between-subgroup mean differences (95% confidence intervals) for selective outcome reporting were as follows: (unclear vs. low) 5.24 mmHg (0.553, 9.94 mmHg).

The green diamond represents the pooled estimate for the overall primary analysis of food sources of fructose-containing sugars on DBP. Within subgroup mean differences are the pooled effect estimates represented by a red circle. 95% confidence intervals are represented by the line through the circle. Data are expressed as mean differences with 95% confidence intervals using the generic inverse-variance method and random effects DerSimonian-Laird model. Inter-study heterogeneity was assessed using the Cochrane Q statistic and quantified using the I^2 statistic, with significance set at p<0.100 and I^2≥50% considered to be evidence of substantial heterogeneity. p<0.050 indicates that the effect size differed between levels of the subgroup.

CI=confidence interval.

S59 Fig (part 1 of 3). Subgroup analyses for the effect of SSBs on SBP (mmHg) in substitution trials.

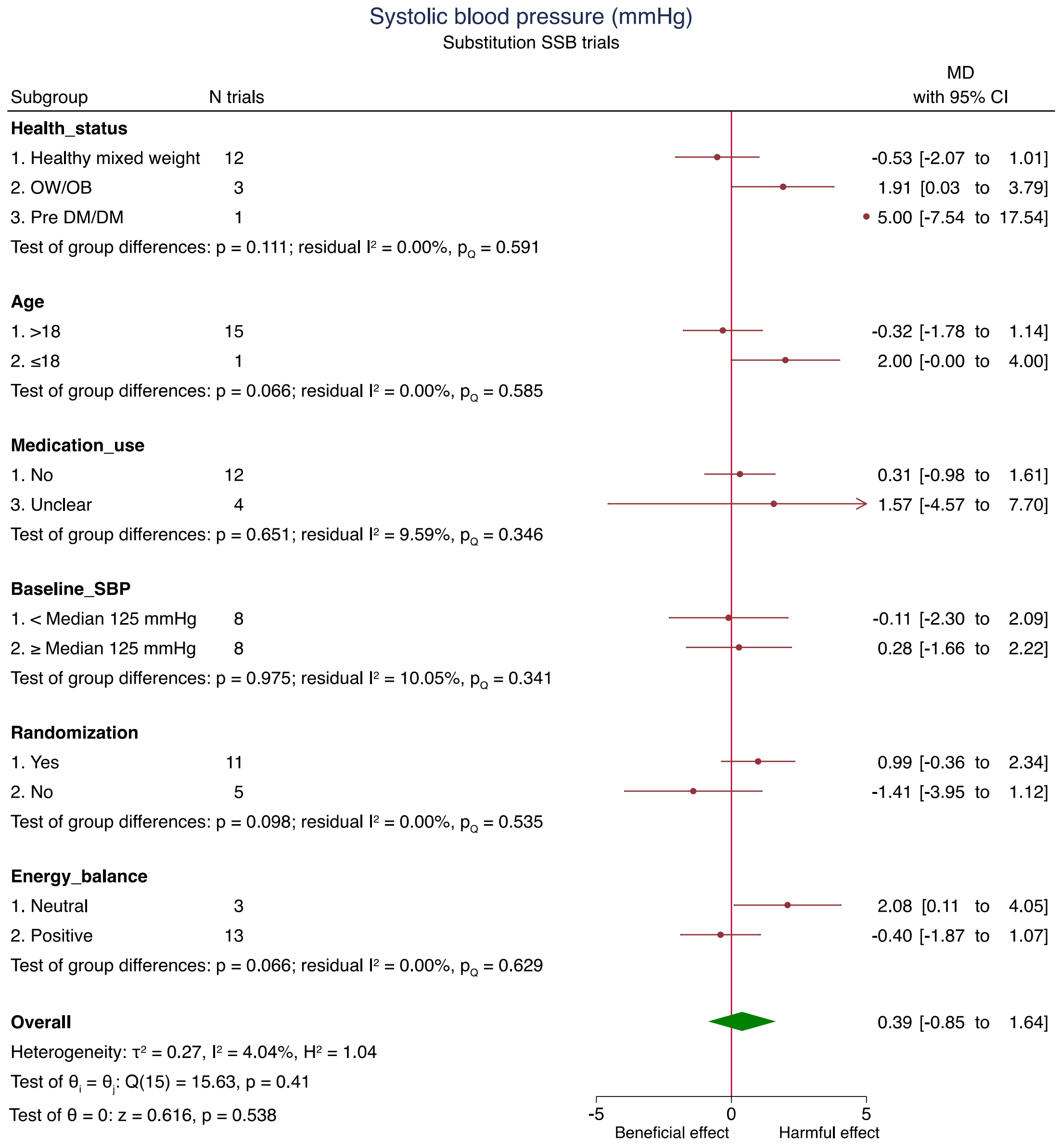

The green diamond represents the pooled estimate for the overall primary analysis of SSB food sources of fructose-containing sugars on SBP. Within subgroup mean differences are the pooled effect estimates represented by a red circle. 95% confidence intervals are represented by the line through the circle. Data are expressed as mean differences with 95% confidence intervals using the generic inverse-variance method and random effects DerSimonian-Laird model. Inter-study heterogeneity was assessed using the Cochrane Q statistic and quantified using the I^2 statistic, with significance set at p<0.100 and I^2≥50% considered to be evidence of substantial heterogeneity. p<0.050 indicates that the effect size differed between levels of the subgroup. CI=confidence interval, DM=diabetes mellitus

S59 Fig (part 2 of 3). Subgroup analyses for the effect of SSBs on SBP (mmHg) in substitution trials.

Systolic blood pressure (mmHg)

Substitution SSB trials

Subgroup	N trials	MD with 95% CI
**Fructose_sugars_type**		
1. Fructose	9	-0.97 [-2.83 to 0.89]
2. Sucrose	5	1.29 [-1.56 to 4.15]
5. HFCS	2	1.09 [-1.81 to 3.99]
Test of group differences: p = 0.186; residual I² = 3.50%, p_Q = 0.399		
**Comparator**		
1. Starch	1	5.00 [-7.54 to 17.54]
2. Glucose	12	-0.62 [-2.12 to 0.87]
4. Lactose	2	2.23 [0.29 to 4.17]
7. Mixed comparator	1	3.00 [-24.44 to 30.44]
Test of group differences: p = 0.126; residual I² = 0.00, p_Q = 0.624		
**Design**		
1. Parallel	8	-1.27 [-3.38 to 0.84]
2. Crossover	8	1.28 [-0.14 to 2.70]
Test of group differences: p = 0.063; residual I² = 3.81%, p_Q = 0.626		
**Follow_up**		
1. ≤8 weeks	14	0.18 [-1.14 to 1.49]
2. >8 weeks	2	2.72 [-1.84 to 7.28]
Test of group differences: p = 0.301; residual I² = 4.08%, p_Q = 0.403		
**Feeding_Control**		
1. Supplemented	11	0.99 [-0.36 to 2.34]
3. Metabolically Controlled	1	3.00 [-24.44 to 30.44]
4. Metabolically Controlled + Supplemented	4	-1.45 [-4.00 to 1.10]
Test of group differences: p = 286; residual I² = 4.17%, p_Q = 0.462		
**Overall**		0.39 [-0.85 to 1.64]
Heterogeneity: τ^2 = 0.27, I² = 4.04%, H² = 1.04		
Test of $\theta_i = \theta_j$: Q(15) = 15.63, p = 0.41		
Test of θ = 0: z = 0.616, p = 0.538		

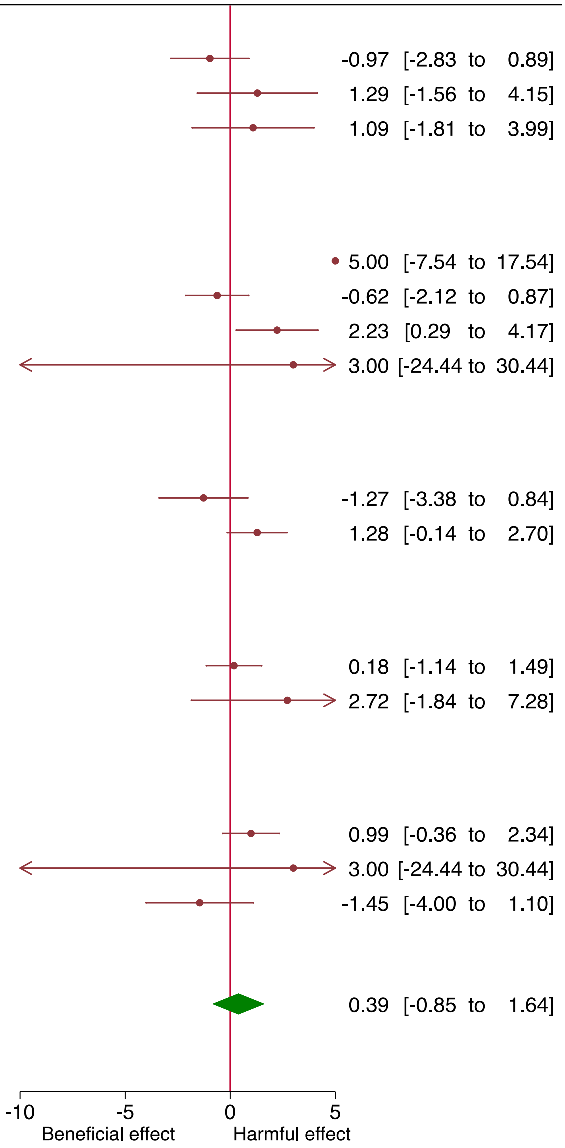

The green diamond represents the pooled estimate for the overall primary analysis of SSB food sources of fructose-containing sugars on SBP. Within subgroup mean differences are the pooled effect estimates represented by a red circle. 95% confidence intervals are represented by the line through the circle. Data are expressed as mean differences with 95% confidence intervals using the generic inverse-variance method and random effects DerSimonian-Laird model. Inter-study heterogeneity was assessed using the Cochrane Q statistic and quantified using the I^2 statistic, with significance set at p<0.100 and I^2≥50% considered to be evidence of substantial heterogeneity. p<0.050 indicates that the effect size differed between levels of the subgroup. CI=confidence interval.

S59 Fig (part 3 of 3). Subgroup analyses for the effect of SSBs on SBP (mmHg) in substitution trials.

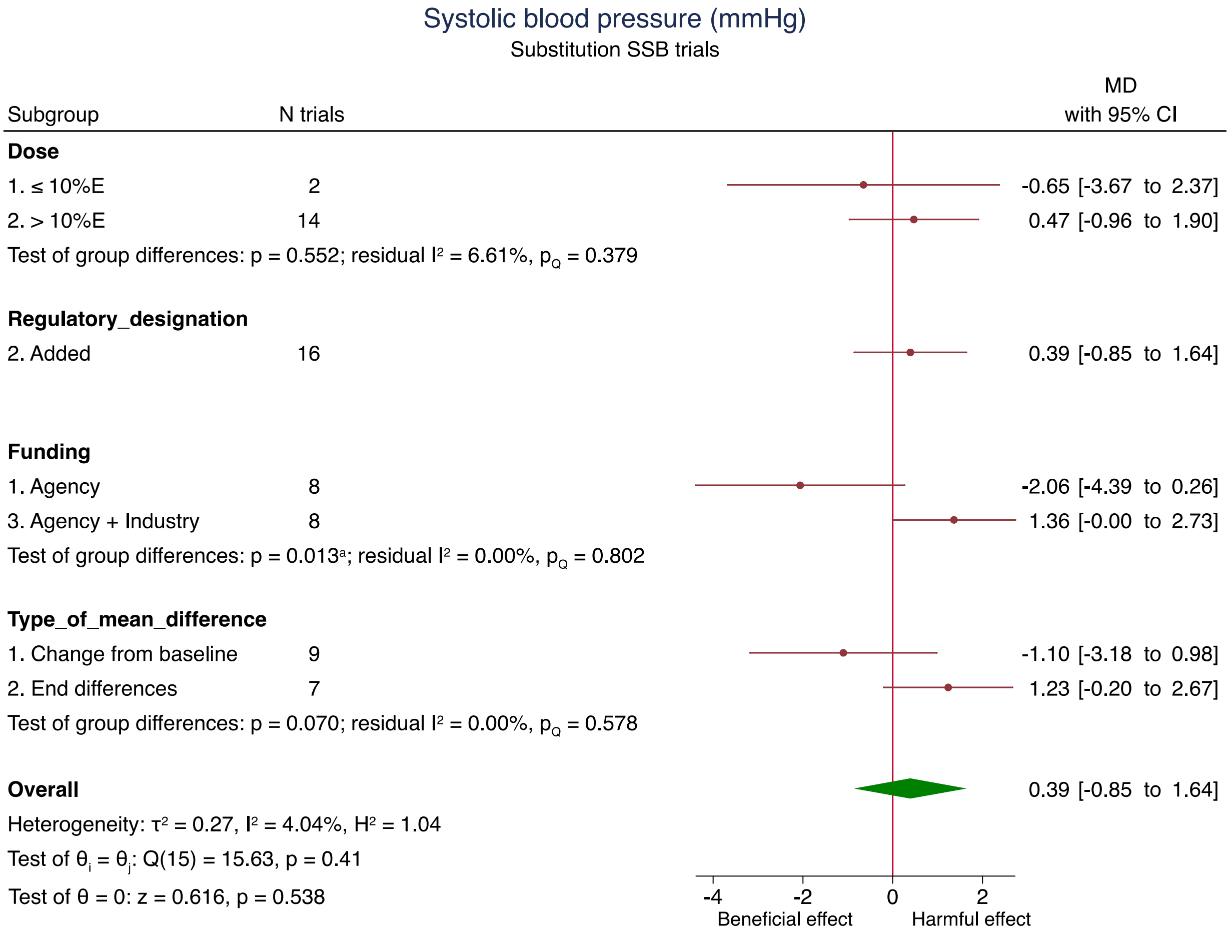

[a]Pairwise between-subgroup mean differences (95% confidence intervals) for design were as follows: (3 vs. 1) 3.42 mmHg (0.727, 6.12 mmHg).

The green diamond represents the pooled estimate for the overall primary analysis of SSB food sources of fructose-containing sugars on SBP. Within subgroup mean differences are the pooled effect estimates represented by a red circle. 95% confidence intervals are represented by the line through the circle. Data are expressed as mean differences with 95% confidence intervals using the generic inverse-variance method and random effects DerSimonian-Laird model. Inter-study heterogeneity was assessed using the Cochrane Q statistic and quantified using the I^2 statistic, with significance set at $p<0.100$ and $I^2 \geq 50\%$ considered to be evidence of substantial heterogeneity. $p<0.050$ indicates that the effect size differed between levels of the subgroup.
CI=confidence interval.

S60 Fig. Risk of bias (using The Cochrane Collaboration Tool) subgroup analysis for the effect of SSBs on SBP (mmHg) in substitution trials.

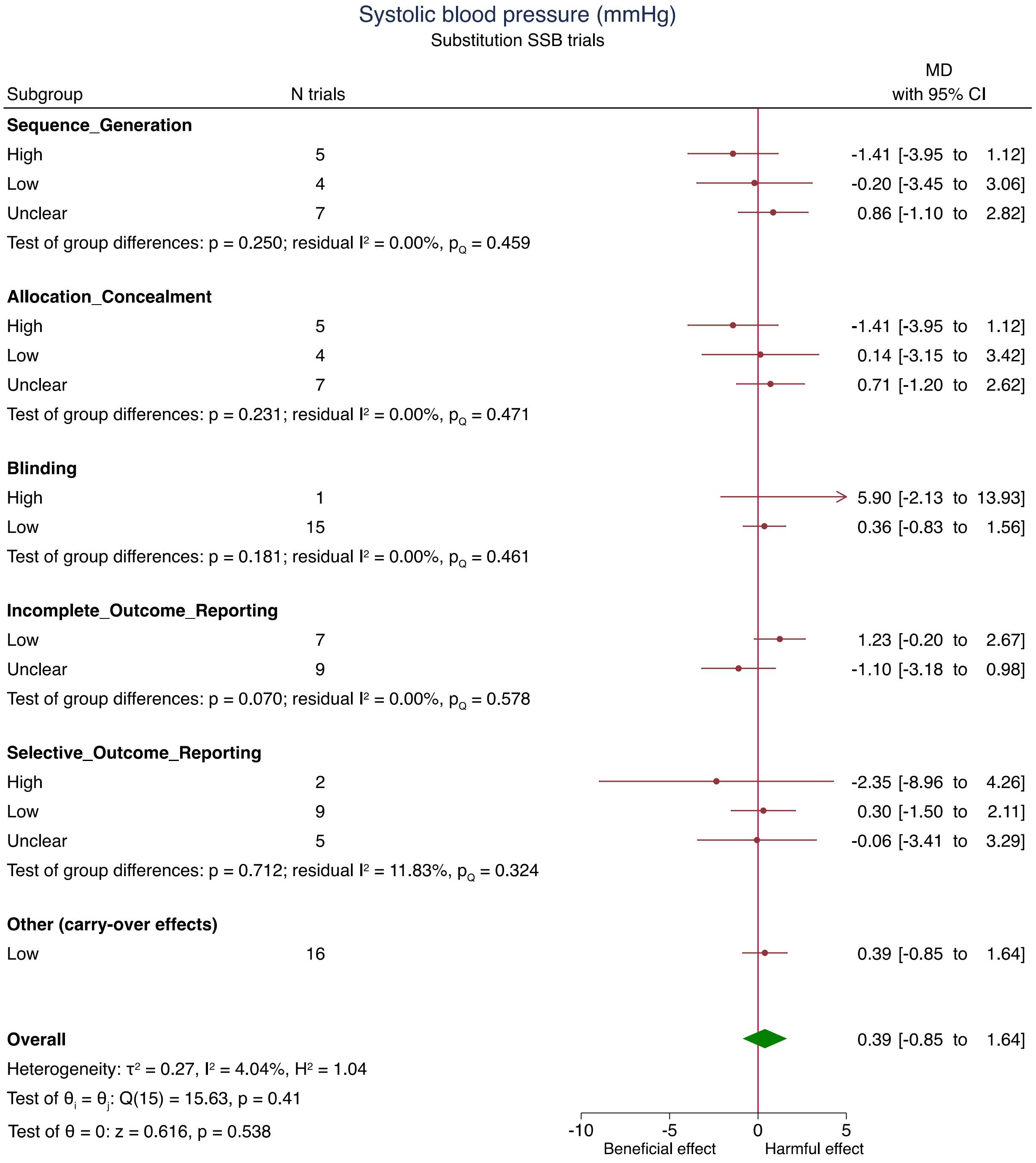

The green diamond represents the pooled estimate for the overall primary analysis of SSB food sources of fructose-containing sugars on SBP. Within subgroup mean differences are the pooled effect estimates represented by a red circle. 95% confidence intervals are represented by the line through the circle. Data are expressed as mean differences with 95% confidence intervals using the generic inverse-variance method and random effects DerSimonian-Laird model. Inter-study heterogeneity was assessed using the Cochrane Q statistic and quantified

using the I^2 statistic, with significance set at $p<0.100$ and $I^2 \geq 50\%$ considered to be evidence of substantial heterogeneity. $p<0.050$ indicates that the effect size differed between levels of the subgroup. CI=confidence interval.

S61 Fig (part 1 of 3). Subgroup analyses for the effect of fruit on SBP (mmHg) in substitution trials.

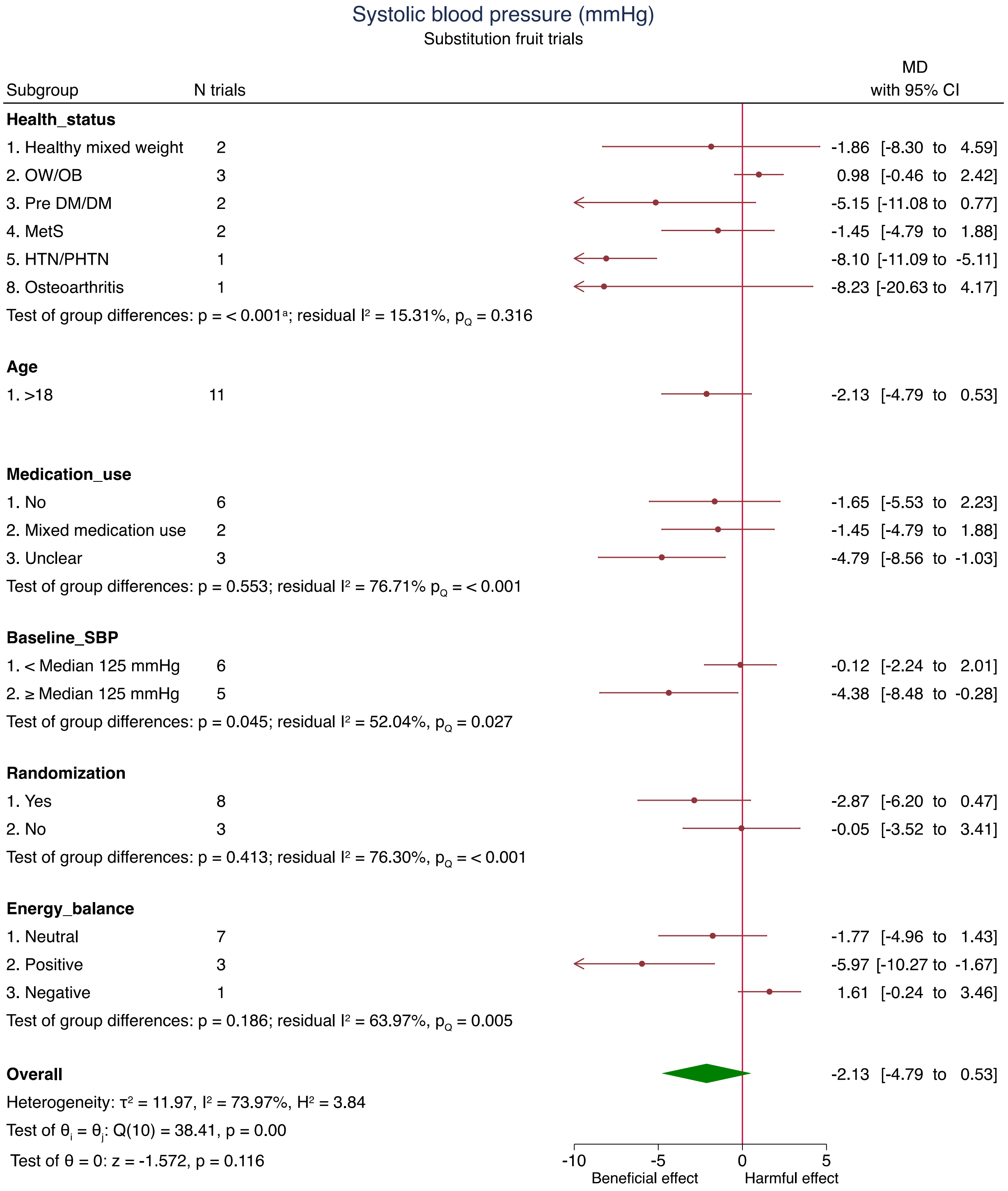

[a]Pairwise between-subgroup mean differences (95% confidence intervals) for health status were as follows: (2 vs. 1) 3.12 mmHg (-1.29, 7.54 mmHg); (3 vs. 1) -2.97 mmHg (-10.3, 4.32 mmHg); (4 vs. 1) 0.887 mmHg (-4.55, 6.32 mmHg); (5 vs. 1) -5.86 mmHg (-11.1, -0.573 mmHg); (6 vs. 1) -5.99 mmHg (-19.1, 7.16 mmHg); (3 vs. 2) -6.09 mmHg (-12.5, 0.267 mmHg); (4 vs. 2) -2.24 mmHg (-6.34, 1.87 mmHg); (5 vs. 2) -8.98 mmHg (-12.9, -5.08 mmHg); (6 vs. 2) -

9.11 mmHg (-21.8, 3.54 mmHg); (4 vs. 3) 3.86 mmHg (-3.25, 11 mmHg); (5 vs. 3) -2.89 mmHg (-9.88, 4.11 mmHg); (5 vs. 3) -2.89 mmHg (-9.88, 4.11 mmHg); (6 vs. 3) -3.02 mmHg (-16.9, 10.9 mmHg); (5 vs. 4) -6.75 mmHg (-11.8, -1.72 mmHg); (6 vs. 4) -6.88 mmHg (-19.9, 6.17 mmHg); (6 vs. 5) -0.13 mmHg (-13.1, 12.9 mmHg).

[b]Pairwise between-subgroup mean differences (95% confidence intervals) for baseline SBP were as follows: (2 vs. 1) -4.24 mmHg (-8.04, -0.0907 mmHg)

The green diamond represents the pooled estimate for the overall primary analysis of fruit food sources of fructose-containing sugars on SBP. Within subgroup mean differences are the pooled effect estimates represented by a red circle. 95% confidence intervals are represented by the line through the circle. Data are expressed as mean differences with 95% confidence intervals using the generic inverse-variance method and random effects DerSimonian-Laird model. Inter-study heterogeneity was assessed using the Cochrane Q statistic and quantified using the I^2 statistic, with significance set at $p<0.100$ and $I^2 \geq 50\%$ considered to be evidence of substantial heterogeneity. $p<0.050$ indicates that the effect size differed between levels of the subgroup.

CI=confidence interval.

S61 Fig (part 2 of 3). Subgroup analyses for the effect of fruit on SBP (mmHg) in substitution trials.

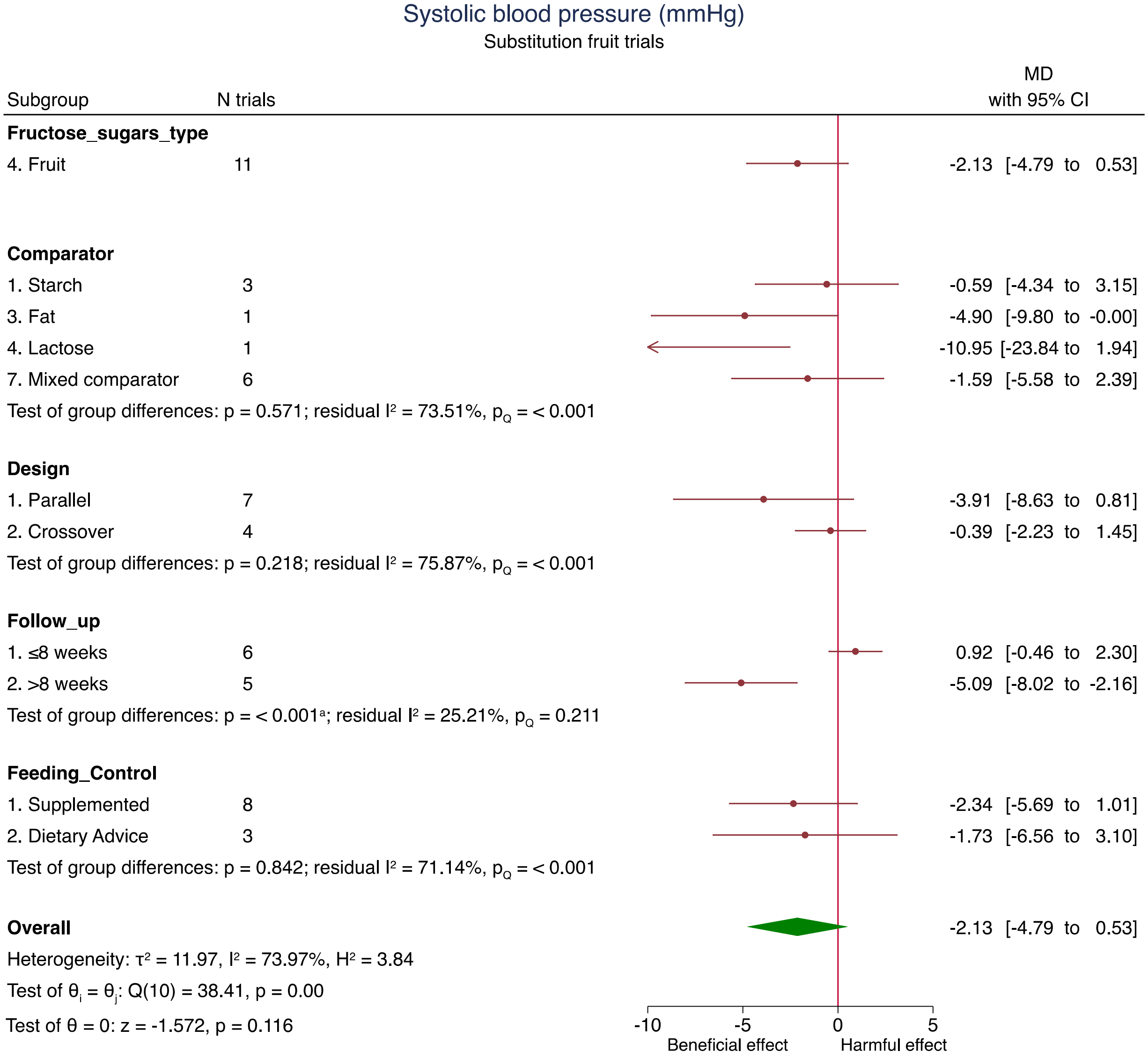

[a]Pairwise between-subgroup mean differences (95% confidence intervals) for follow up were as follows: (2 vs. 1) -5.91 mmHg (-9.01, -2.82 mmHg)

The green diamond represents the pooled estimate for the overall primary analysis of fruit food sources of fructose-containing sugars on SBP. Within subgroup mean differences are the pooled effect estimates represented by a red circle. 95% confidence intervals are represented by the line through the circle. Data are expressed as mean differences with 95% confidence intervals using the generic inverse-variance method and random effects DerSimonian-Laird model. Inter-study heterogeneity was assessed using the Cochrane Q statistic and quantified using the I^2 statistic, with significance set at p<0.100 and I^2≥50% considered to be evidence of substantial heterogeneity. p<0.050 indicates that the effect size differed between levels of the subgroup. CI=confidence interval.

S61 Fig (part 3 of 3). Subgroup analyses for the effect of fruit on SBP (mmHg) in substitution trials.

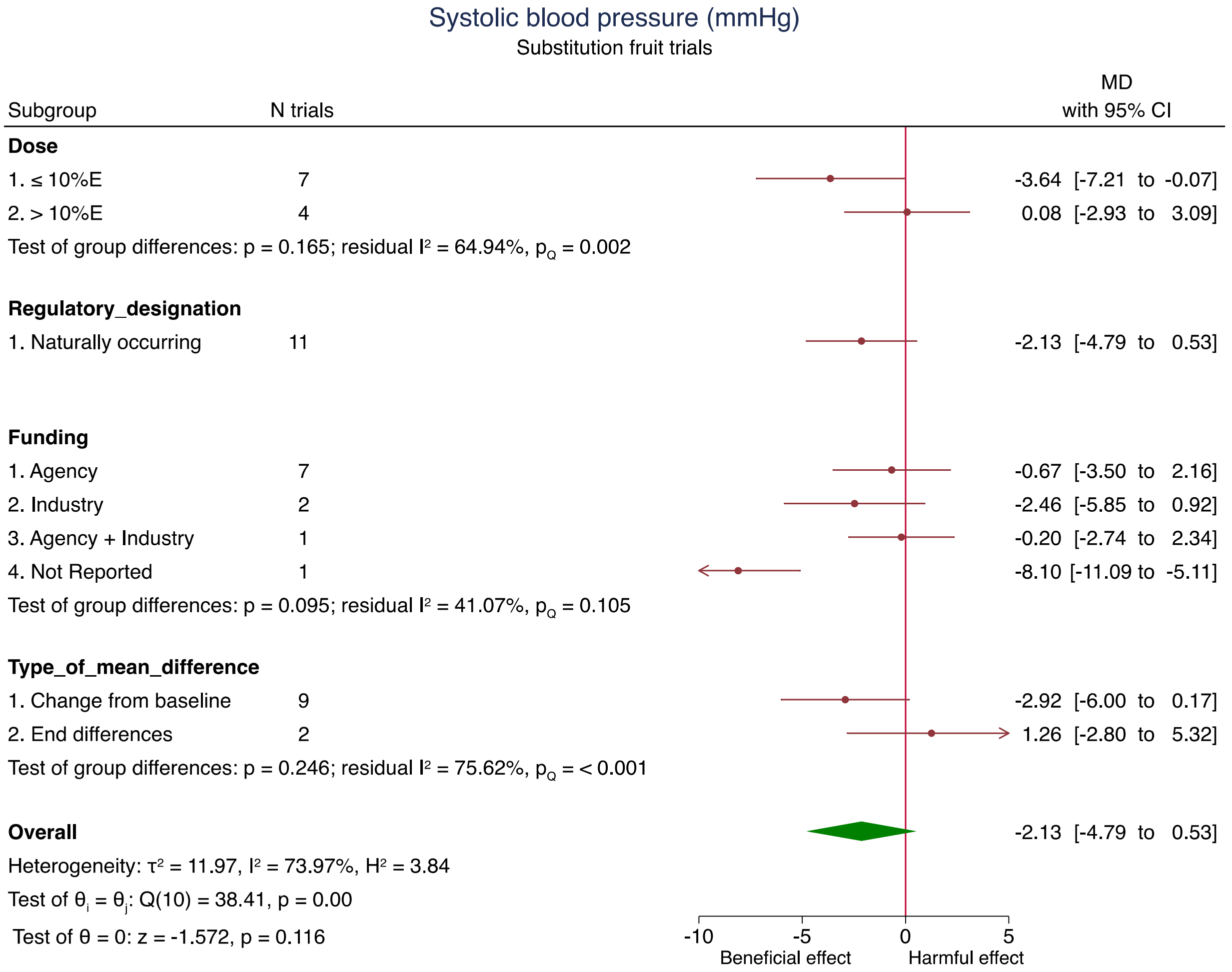

The green diamond represents the pooled estimate for the overall primary analysis of fruit food sources of fructose-containing sugars on SBP. Within subgroup mean differences are the pooled effect estimates represented by a red circle. 95% confidence intervals are represented by the line through the circle. Data are expressed as mean differences with 95% confidence intervals using the generic inverse-variance method and random effects DerSimonian-Laird model. Inter-study heterogeneity was assessed using the Cochrane Q statistic and quantified using the I^2 statistic, with significance set at p<0.100 and $I^2 \geq 50\%$ considered to be evidence of substantial heterogeneity. p<0.050 indicates that the effect size differed between levels of the subgroup. CI=confidence interval.

S62 Fig. Risk of bias (using The Cochrane Collaboration Tool) subgroup analysis for the effect of fruit on SBP (mmHg) in substitution trials.

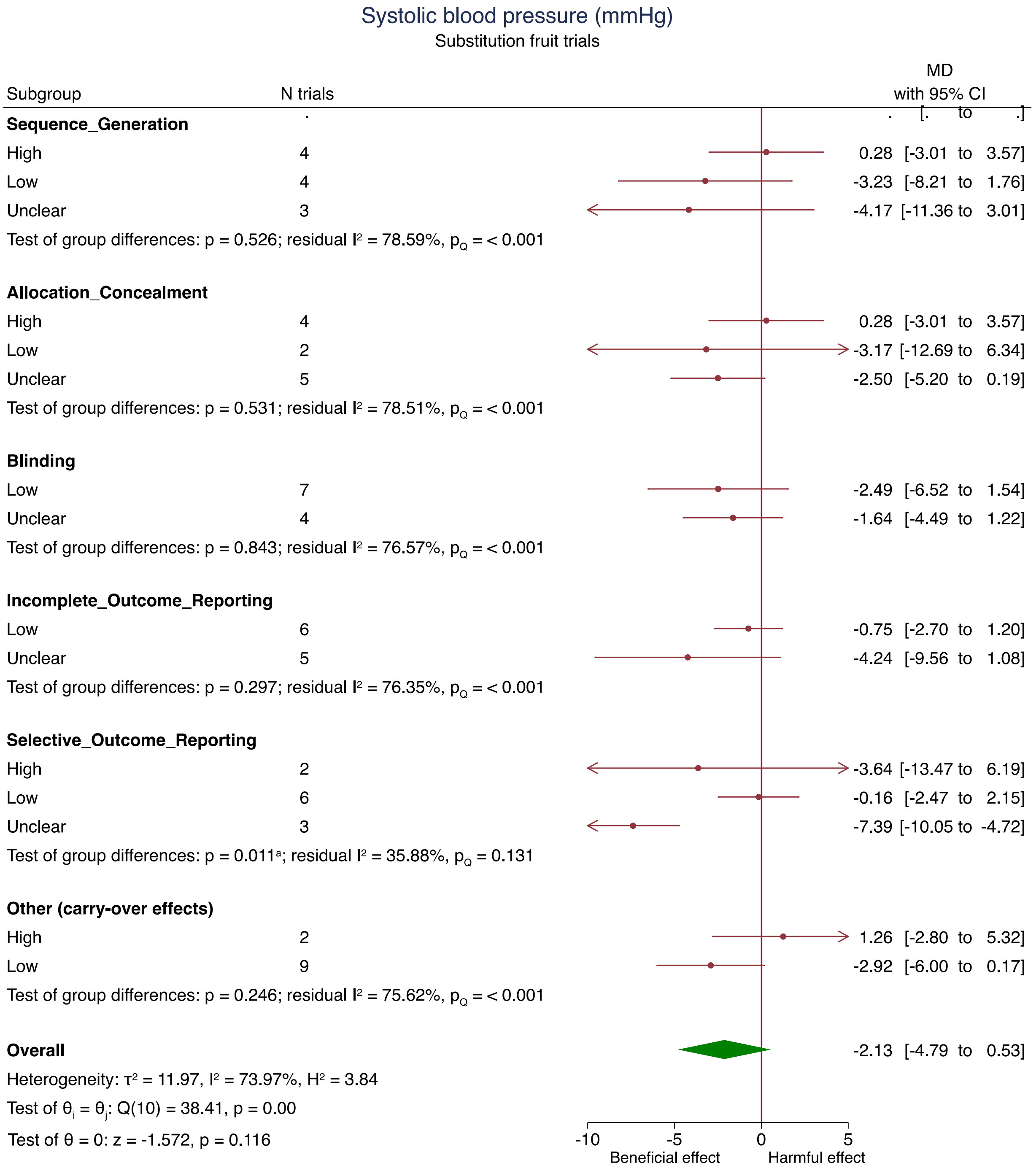

[a]Pairwise between-subgroup mean differences (95% confidence intervals) for selective outcome reporting were as follows: (low vs. high) 1.08 mmHg (-3.72, 5.86 mmHg); (unclear vs. high) -5.73 mmHg (-11.4, -0.0897 mmHg); (unclear vs. low) -6.01 mmHg (-11.3, -2.34 mmHg).

The green diamond represents the pooled estimate for the overall primary analysis of fruit food sources of fructose-containing sugars on SBP. Within subgroup mean differences are the pooled effect estimates represented by a red circle. 95% confidence intervals are represented by the line through the circle. Data are expressed as

mean differences with 95% confidence intervals using the generic inverse-variance method and random effects DerSimonian-Laird model. Inter-study heterogeneity was assessed using the Cochrane Q statistic and quantified using the I^2 statistic, with significance set at $p<0.100$ and $I^2 \geq 50\%$ considered to be evidence of substantial heterogeneity. $p<0.050$ indicates that the effect size differed between levels of the subgroup.
CI=confidence interval.

S63 Fig (part 1 of 3). Subgroup analyses for the effect of mixed sources (with SSBs) on SBP (mmHg) in substitution trials.

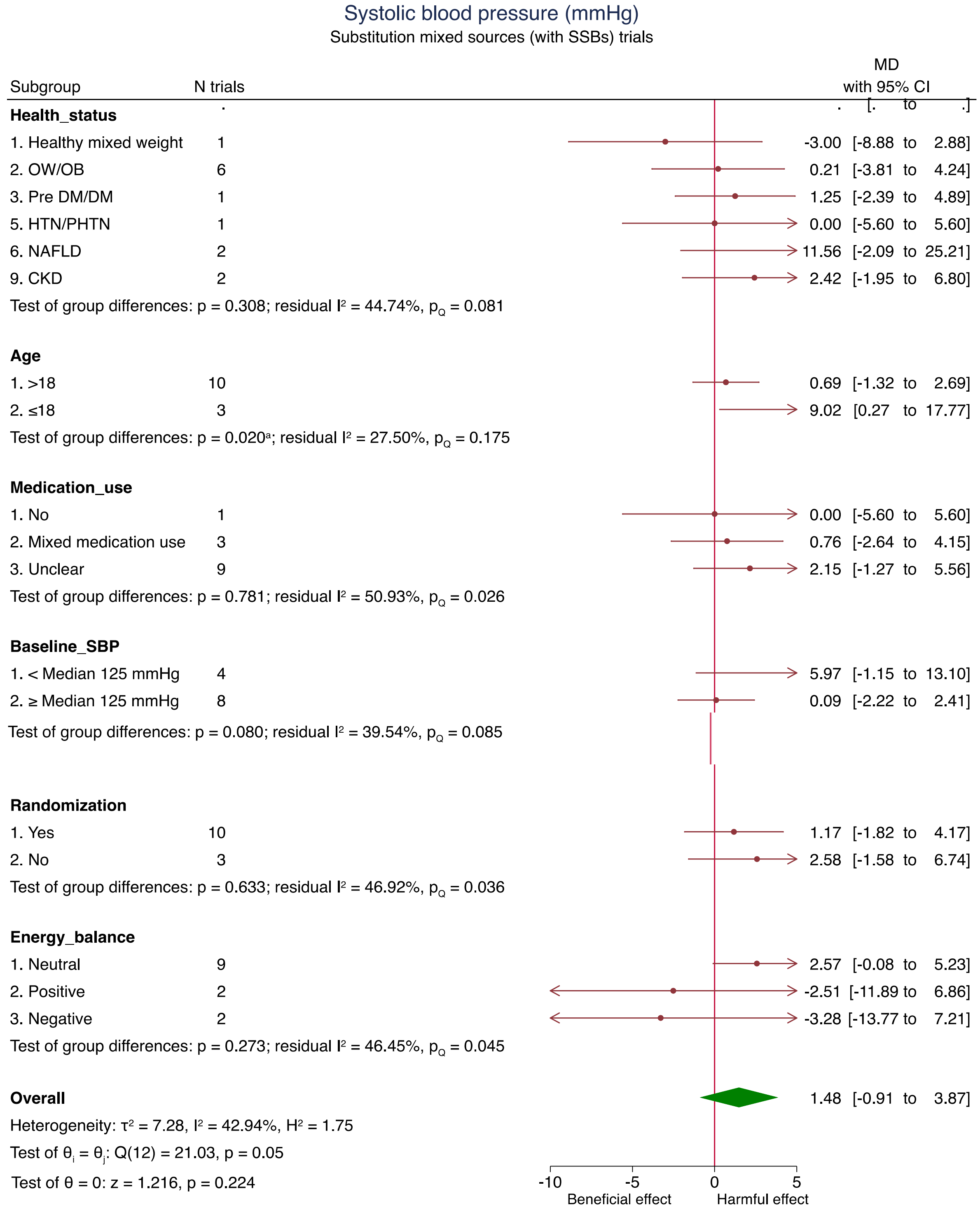

[a]Pairwise between-subgroup mean differences (95% confidence intervals) for design were as follows: (2 vs. 1) 7.58 mmHg (1.21, 14 mmHg).
[b]N=1 trial missing baseline SBP data.
The green diamond represents the pooled estimate for the overall primary analysis of SSB (and other food sources) food sources of fructose-containing sugars on SBP. Within subgroup mean differences are the pooled effect estimates represented by a red circle. 95% confidence intervals are represented by the line through the circle. Data are expressed as mean differences with 95% confidence intervals using the generic inverse-variance method and random effects DerSimonian-Laird model. Inter-study heterogeneity was assessed using the Cochrane Q statistic and quantified using the I^2 statistic, with significance set at $p<0.100$ and $I^2 \geq 50\%$ considered to be evidence of substantial heterogeneity. $p<0.050$ indicates that the effect size differed between levels of the subgroup. CI=confidence interval.

S63 Fig (part 2 of 3). Subgroup analyses for the effect of mixed sources (with SSBs) on SBP (mmHg) in substitution trials.

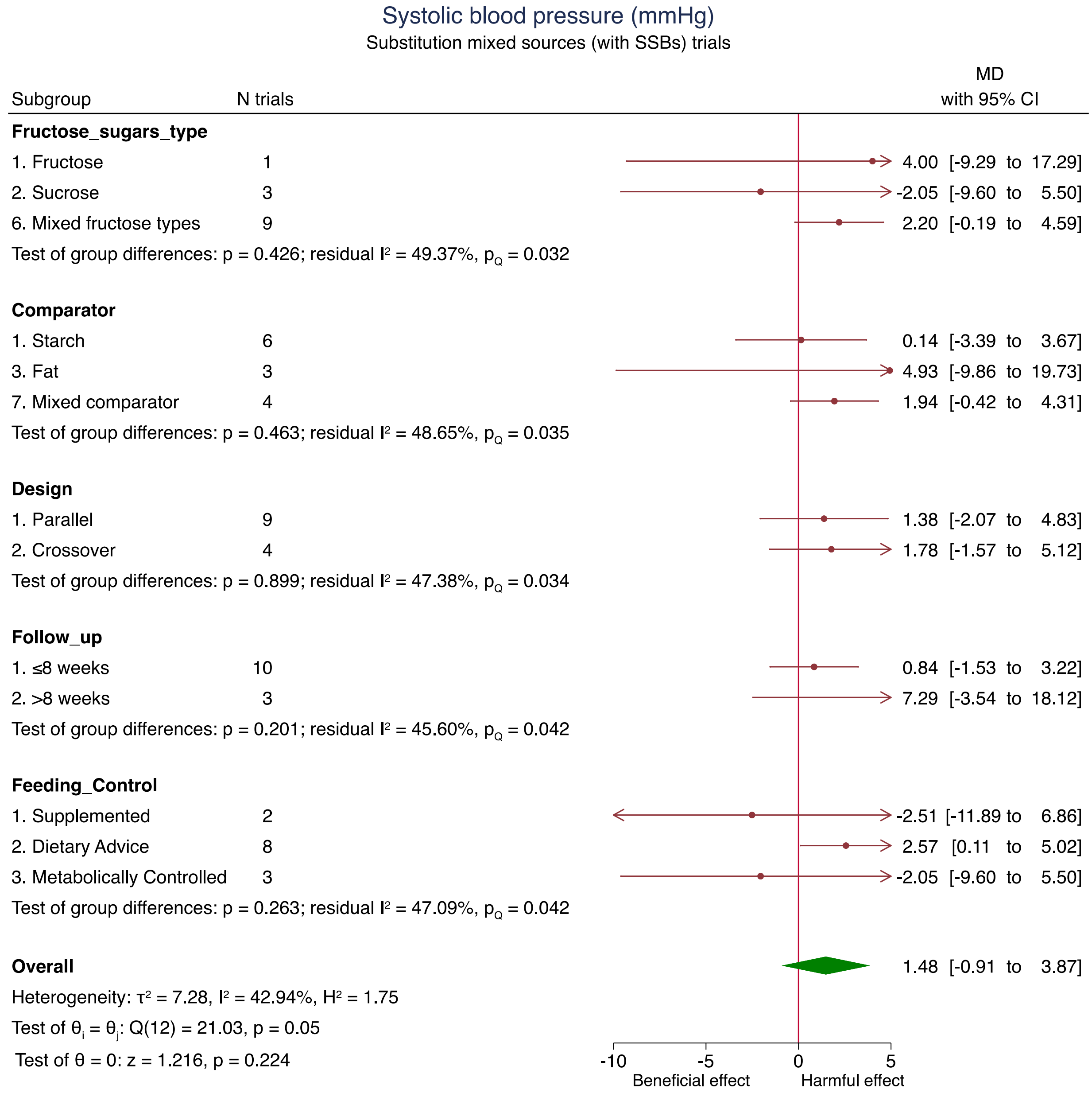

The green diamond represents the pooled estimate for the overall primary analysis of SSB (and other food sources) food sources of fructose-containing sugars on SBP. Within subgroup mean differences are the pooled effect estimates represented by a red circle. 95% confidence intervals are represented by the line through the circle. Data are expressed as mean differences with 95% confidence intervals using the generic inverse-variance method and random effects DerSimonian-Laird model. Inter-study heterogeneity was assessed using the Cochrane Q statistic and quantified using the I^2 statistic, with significance set at p<0.100 and I^2≥50% considered to be evidence of substantial heterogeneity. p<0.050 indicates that the effect size differed between levels of the subgroup. CI=confidence interval.

S63 Fig (part 3 of 3). Subgroup analyses for the effect of mixed sources (with SSBs) on SBP (mmHg) in substitution trials.

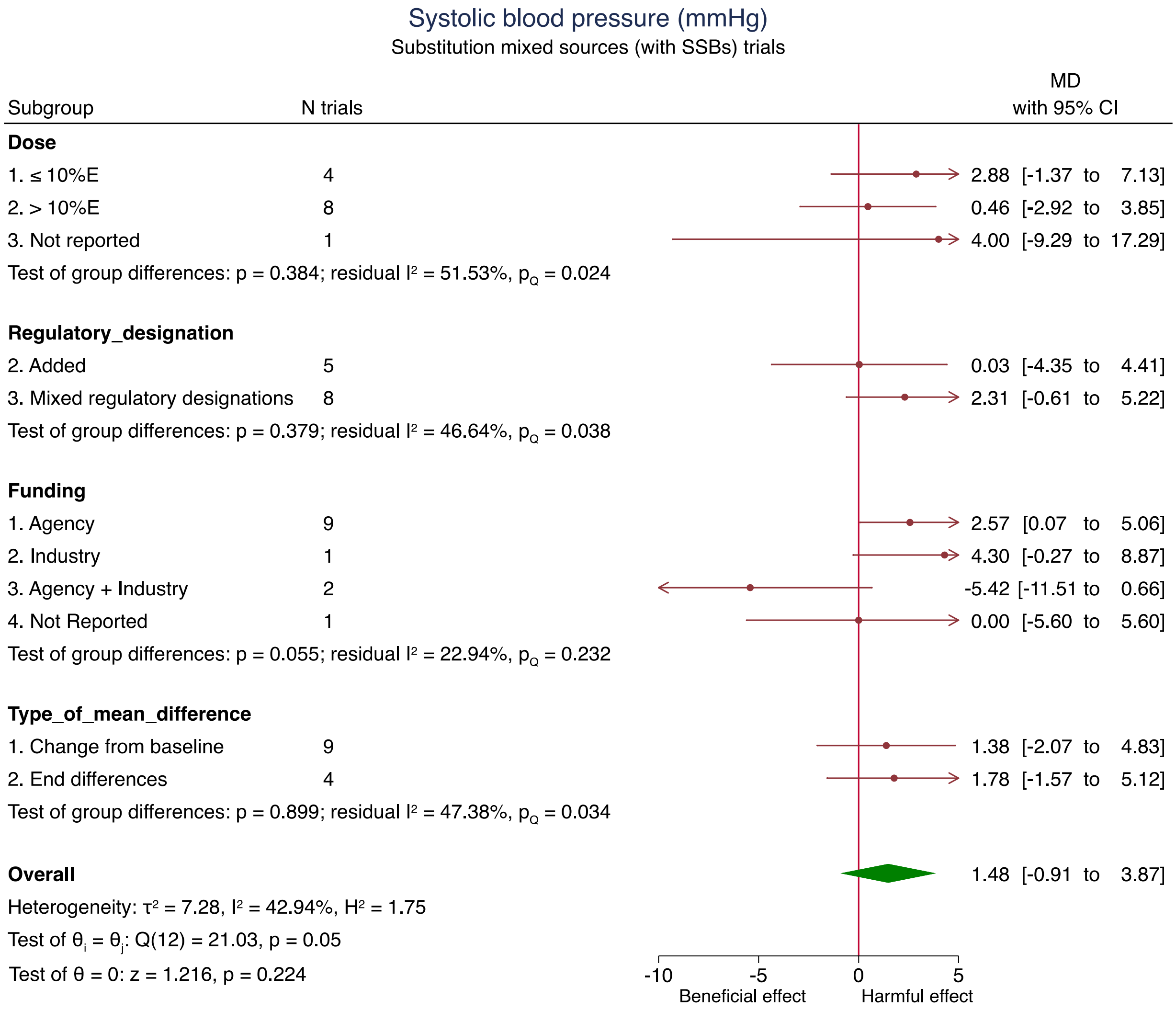

The green diamond represents the pooled estimate for the overall primary analysis of SSB (and other food sources) food sources of fructose-containing sugars on SBP. Within subgroup mean differences are the pooled effect estimates represented by a red circle. 95% confidence intervals are represented by the line through the circle. Data are expressed as mean differences with 95% confidence intervals using the generic inverse-variance method and random effects DerSimonian-Laird model. Inter-study heterogeneity was assessed using the Cochrane Q statistic and quantified using the I^2 statistic, with significance set at p<0.100 and $I^2 \geq 50\%$ considered to be evidence of substantial heterogeneity. p<0.050 indicates that the effect size differed between levels of the subgroup.

S64 Fig. Risk of bias (using The Cochrane Collaboration Tool) subgroup analysis for the effect of mixed sources (with SSBs) on SBP (mmHg) in substitution trials.

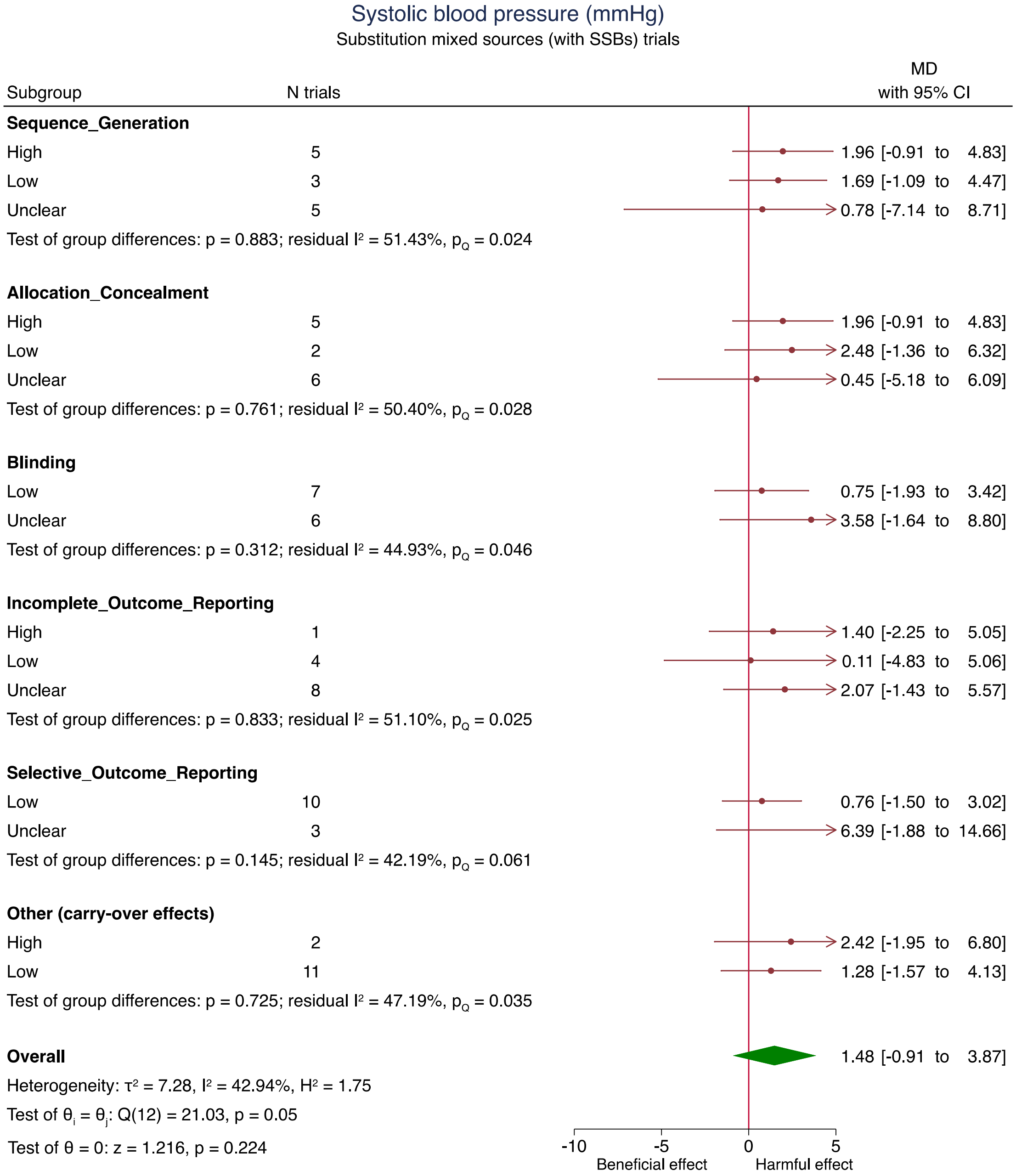

The green diamond represents the pooled estimate for the overall primary analysis of SSB (and other food sources) food sources of fructose-containing sugars on SBP. Within subgroup mean differences are the pooled effect estimates represented by a red circle. 95% confidence intervals are represented by the line through the circle. Data are expressed as mean differences with 95% confidence intervals using the generic inverse-variance method and random effects DerSimonian-Laird model. Inter-study heterogeneity was assessed using the Cochrane Q statistic and quantified using the I^2 statistic, with significance set at p<0.100 and I^2≥50% considered to be evidence of substantial heterogeneity. p<0.050 indicates that the effect size differed between levels of the subgroup.

S65 Fig (part 1 of 3). Subgroup analyses for the effect of SSBs on SBP (mmHg) in addition trials.

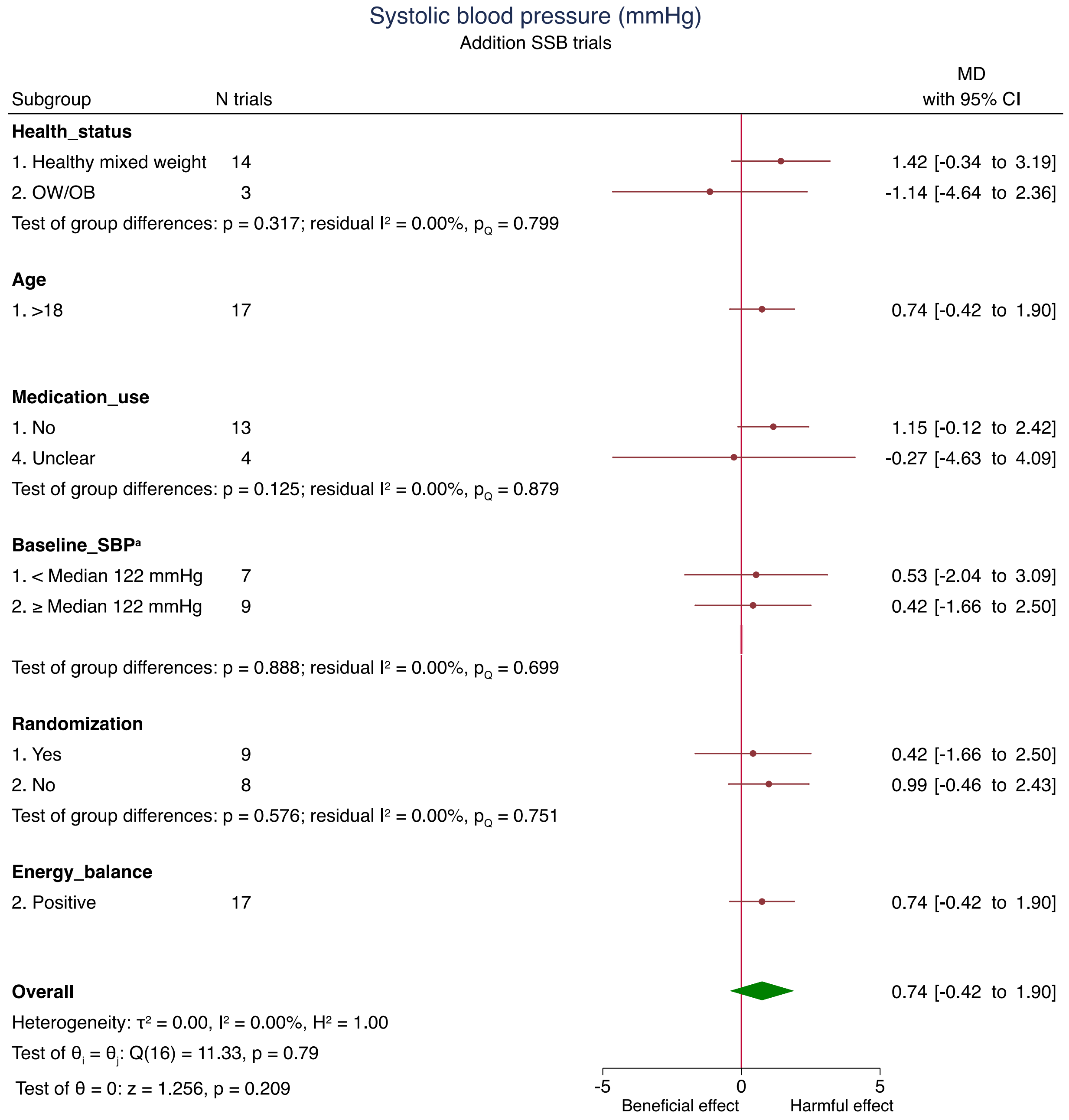

[a]N=1 trial missing baseline SBP data.

The green diamond represents the pooled estimate for the overall primary analysis of SSB food sources of fructose-containing sugars on SBP. Within subgroup mean differences are the pooled effect estimates represented by a red circle. 95% confidence intervals are represented by the line through the circle. Data are expressed as mean differences with 95% confidence intervals using the generic inverse-variance method and random effects DerSimonian-Laird model. Inter-study heterogeneity was assessed using the Cochrane Q statistic and quantified using the I^2 statistic, with significance set at p<0.100 and I^2≥50% considered to be evidence of substantial heterogeneity. p<0.050 indicates that the effect size differed between levels of the subgroup.

CI=confidence interval; OB=obese; OW=overweight.

S65 Fig (part 2 of 3). Subgroup analyses for the effect of SSBs on SBP (mmHg) in addition trials.

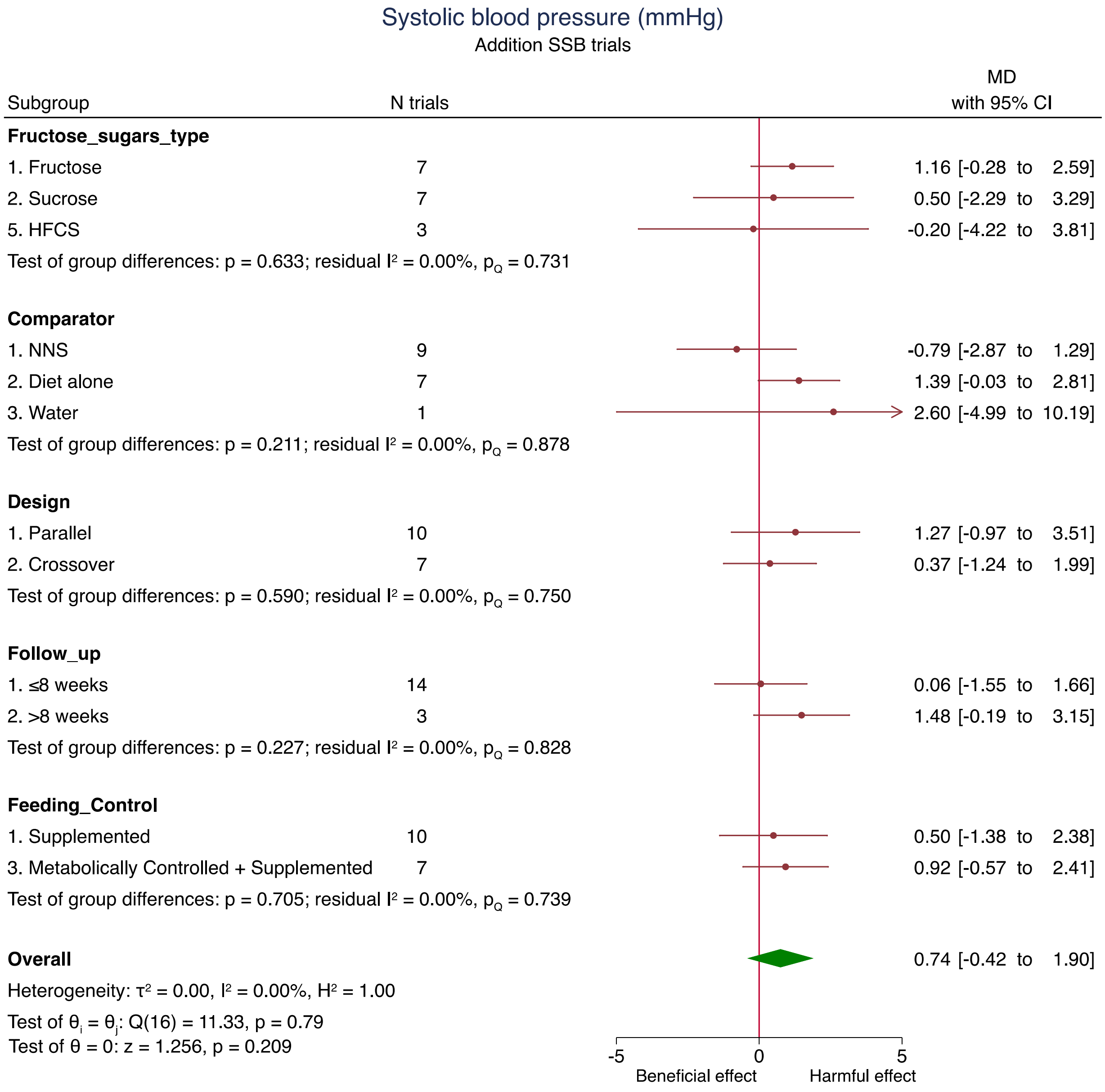

The green diamond represents the pooled estimate for the overall primary analysis of SSB food sources of fructose-containing sugars on SBP. Within subgroup mean differences are the pooled effect estimates represented by a red circle. 95% confidence intervals are represented by the line through the circle. Data are expressed as mean differences with 95% confidence intervals using the generic inverse-variance method and random effects DerSimonian-Laird model. Inter-study heterogeneity was assessed using the Cochrane Q statistic and quantified using the I^2 statistic, with significance set at p<0.100 and I^2≥50% considered to be evidence of substantial heterogeneity. p<0.050 indicates that the effect size differed between levels of the subgroup. CI=confidence interval; HFCS=high fructose corn syrup; NNS=non-nutritive sweetener.

S65 Fig (part 3 of 3). Subgroup analyses for the effect of SSBs on SBP (mmHg) in addition trials.

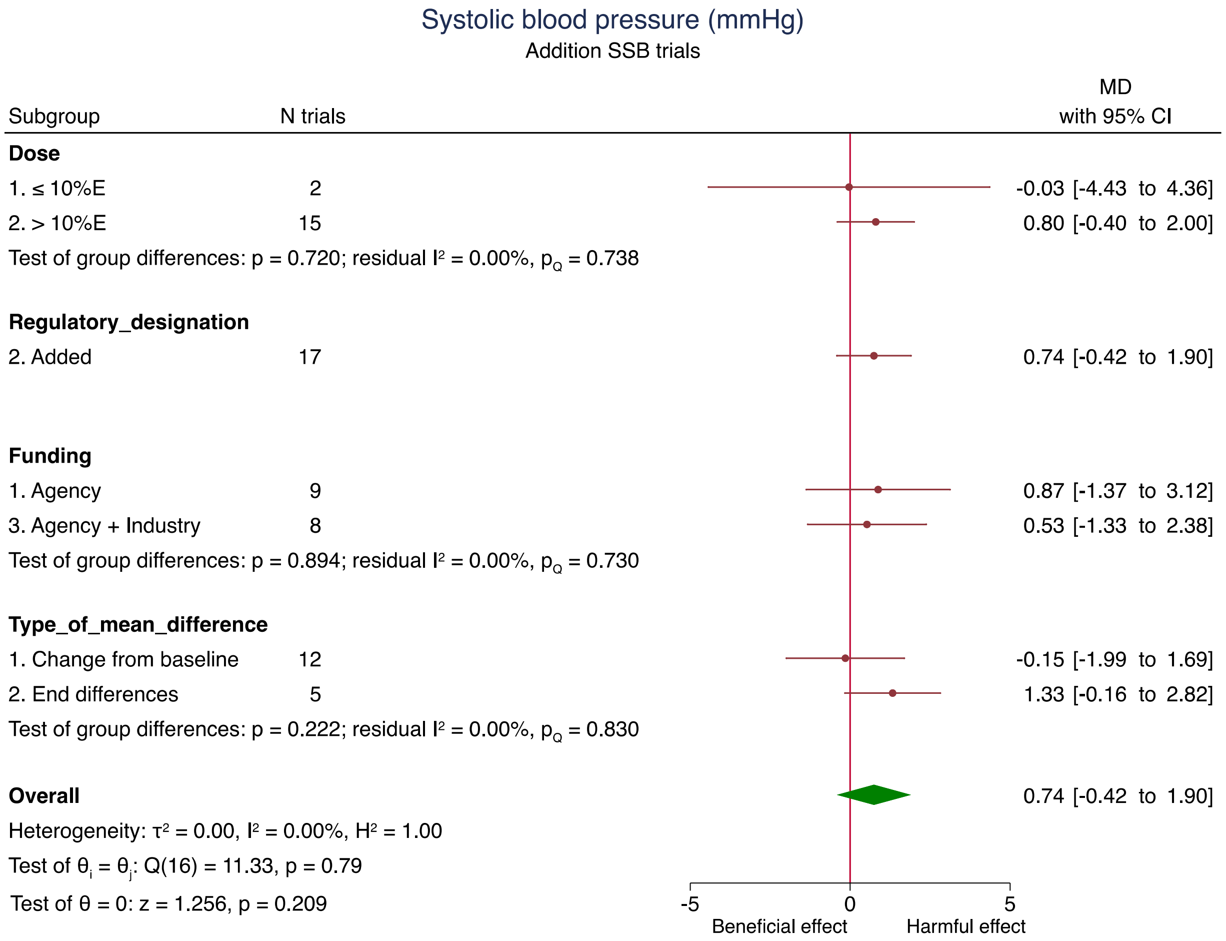

The green diamond represents the pooled estimate for the overall primary analysis of SSB food sources of fructose-containing sugars on SBP. Within subgroup mean differences are the pooled effect estimates represented by a red circle. 95% confidence intervals are represented by the line through the circle. Data are expressed as mean differences with 95% confidence intervals using the generic inverse-variance method and random effects DerSimonian-Laird model. Inter-study heterogeneity was assessed using the Cochrane Q statistic and quantified using the I^2 statistic, with significance set at p<0.100 and $I^2 \geq 50\%$ considered to be evidence of substantial heterogeneity. p<0.050 indicates that the effect size differed between levels of the subgroup.
%E=percent of total daily energy intake; CI=confidence interval.

S66 Fig. Risk of bias (using The Cochrane Collaboration Tool) subgroup analysis for the effect of SSBs on SBP (mmHg) in addition trials.

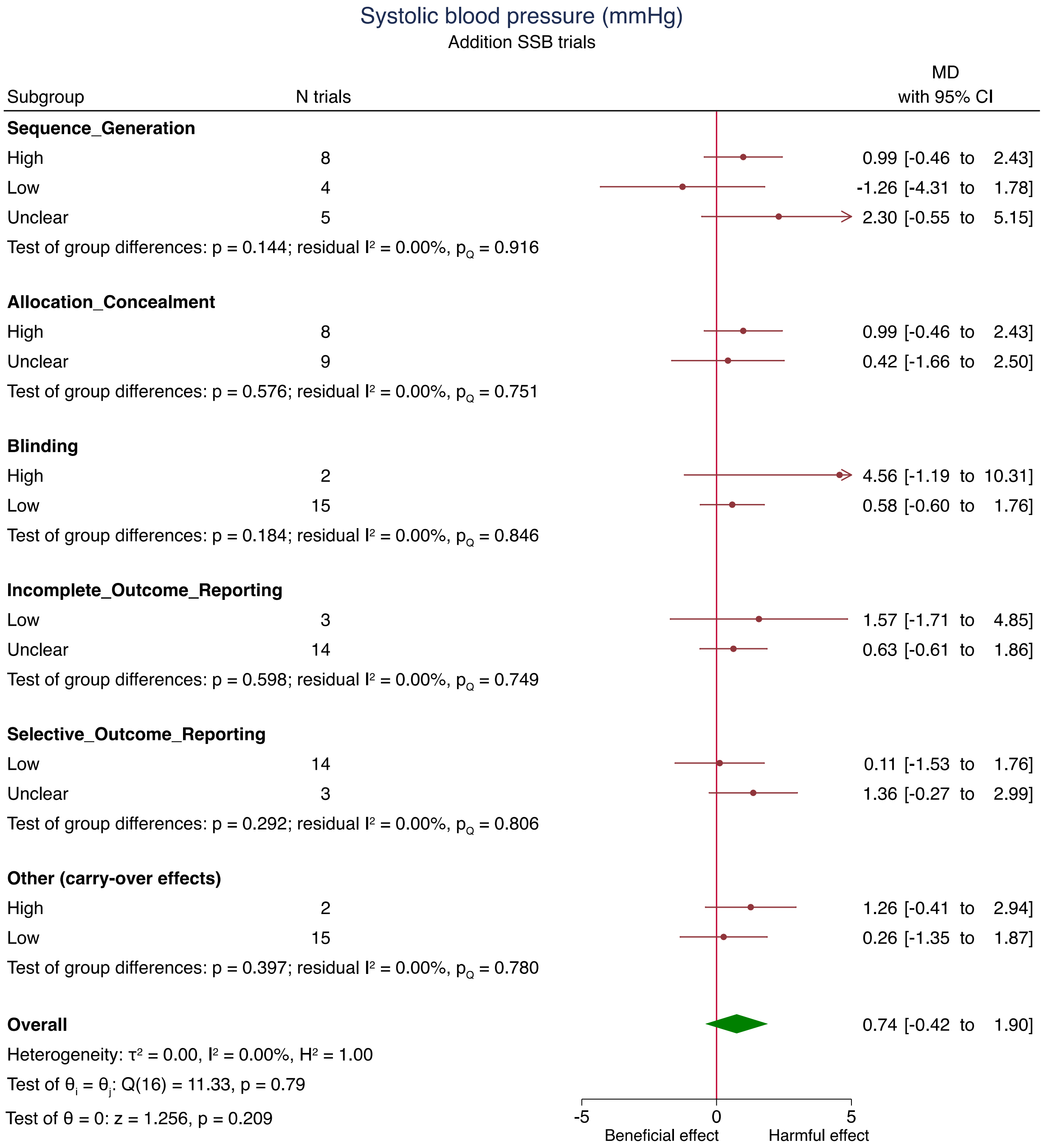

The green diamond represents the pooled estimate for the overall primary analysis of SSB food sources of fructose-containing sugars on SBP. Within subgroup mean differences are the pooled effect estimates represented by a red circle. 95% confidence intervals are represented by the line through the circle. Data are expressed as mean differences with 95% confidence intervals using the generic inverse-variance method and random effects DerSimonian-Laird model. Inter-study heterogeneity was assessed using the Cochrane Q statistic and quantified

using the I^2 statistic, with significance set at $p<0.100$ and $I^2 \geq 50\%$ considered to be evidence of substantial heterogeneity. $p<0.050$ indicates that the effect size differed between levels of the subgroup. CI=confidence interval.

S67 Fig (part 1 of 3). Subgroup analyses for the effect of 100% fruit juice on SBP (mmHg) in addition trials.

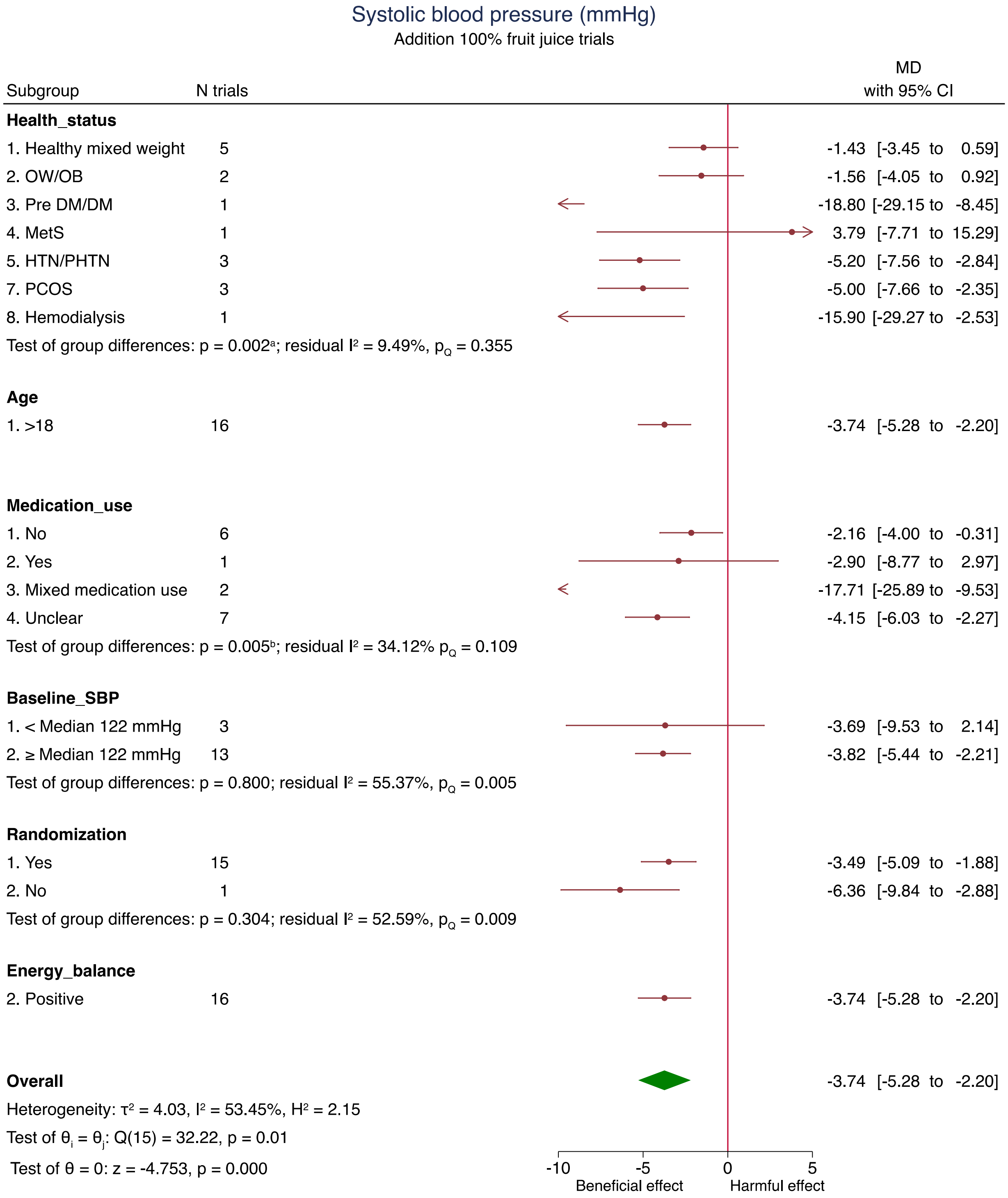

[a]Pairwise between-subgroup mean differences (95% confidence intervals) for health status were as follows: (2 vs. 1) -0.131 mmHg (-3.6, 3.33 mmHg); (3 vs. 1) -17.4 mmHg (-28, -6.73 mmHg); (4 vs. 1) 5.23 mmHg (-6.53, 17 mmHg); (5 vs. 1) -3.73 mmHg (-6.98, -0.479 mmHg); (7 vs. 1) -2.78 mmHg (-5.32, -0.233 mmHg); (8 vs. 1) -14.5 mmHg (-28, -0.875 mmHg); (3 vs. 2) -17.2 mmHg (-28, -6.45 mmHg); (4 vs. 2) 5.36 mmHg (-6.53, 17.2 mmHg); (5 vs. 2) -3.6

mmHg (-7.3, 0.105 mmHg); (7 vs. 2) -2.65 mmHg (-5.75, 0.456 mmHg); (8 vs. 2) -14.3 mmHg (-28, -0.628 mmHg); (4 vs. 3) 22.6 mmHg (7.02, 38.2 mmHg); (5 vs. 3) 13.6 mmHg (2.92, 24.3 mmHg); (7 vs. 3) 14.6 mmHg (4.06, 25.1 mmHg); (8 vs. 3) 2.9 mmHg (-14.1, 19.9 mmHg); (5 vs. 4) -8.96 mmHg (-20.8, 2.87 mmHg); (7 vs. 4) -8.01 mmHg (-19.7, 3.65 mmHg); (8 vs. 4) -19.7 mmHg (-37.4, -1.97 mmHg); (7 vs. 5) 0.953 mmHg (-1.91, 3.82 mmHg); (8 vs. 5) -10.7 mmHg (24.4, 2.92 mmHg); (8 vs. 7) -11.7 mmHg (-25.2, 1.82 mmHg)

[b]Pairwise between-subgroup mean differences (95% confidence intervals) for medication use were as follows: (2 vs. 1) -0.763 mmHg (-7.52, 6 mmHg); (3 vs. 1) -15.6 mmHg (-24.2, -6.9 mmHg); (4 vs. 1) -1.94 mmHg (-4.61, 0.738 mmHg); (3 vs. 2) -14.8 mmHg (-25.4, -4.22 mmHg); (4 vs. 2) -1.17 mmHg (-7.81, 5.46 mmHg); (4 vs. 3) 13.6 mmHg (5.06, 22.2 mmHg).

The green diamond represents the pooled estimate for the overall primary analysis of 100% fruit juice food sources of fructose-containing sugars on SBP. Within subgroup mean differences are the pooled effect estimates represented by a red circle. 95% confidence intervals are represented by the line through the circle. Data are expressed as mean differences with 95% confidence intervals using the generic inverse-variance method and random effects DerSimonian-Laird model. Inter-study heterogeneity was assessed using the Cochrane Q statistic and quantified using the I^2 statistic, with significance set at $p<0.100$ and $I^2 \geq 50\%$ considered to be evidence of substantial heterogeneity. $p<0.050$ indicates that the effect size differed between levels of the subgroup. CI=confidence interval; HTN=hypertensive; OB=obese; OW=overweight; PHTN=pre-hypertensive.

S67 Fig (part 2 of 3). Subgroup analyses for the effect of 100% fruit juice on SBP (mmHg) in addition trials.

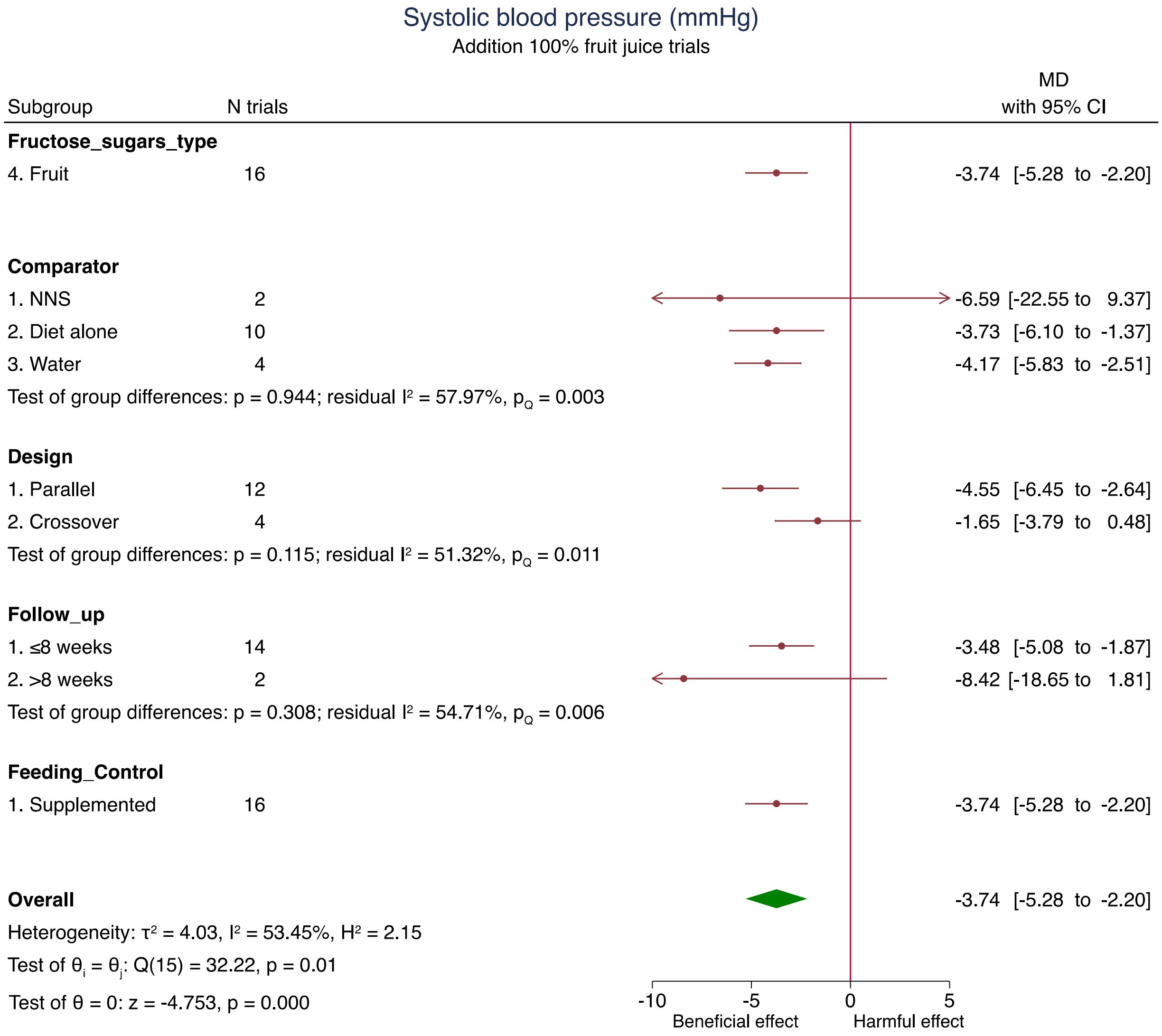

The green diamond represents the pooled estimate for the overall primary analysis of 100% fruit juice food sources of fructose-containing sugars on SBP. Within subgroup mean differences are the pooled effect estimates represented by a red circle. 95% confidence intervals are represented by the line through the circle. Data are expressed as mean differences with 95% confidence intervals using the generic inverse-variance method and random effects DerSimonian-Laird model. Inter-study heterogeneity was assessed using the Cochrane Q statistic and quantified using the I^2 statistic, with significance set at $p<0.100$ and $I^2 \geq 50\%$ considered to be evidence of substantial heterogeneity. $p<0.050$ indicates that the effect size differed between levels of the subgroup. CI=confidence interval; NNS=non-nutritive sweetener.

S67 Fig (part 3 of 3). Subgroup analyses for the effect of 100% fruit juice on SBP (mmHg) in addition trials.

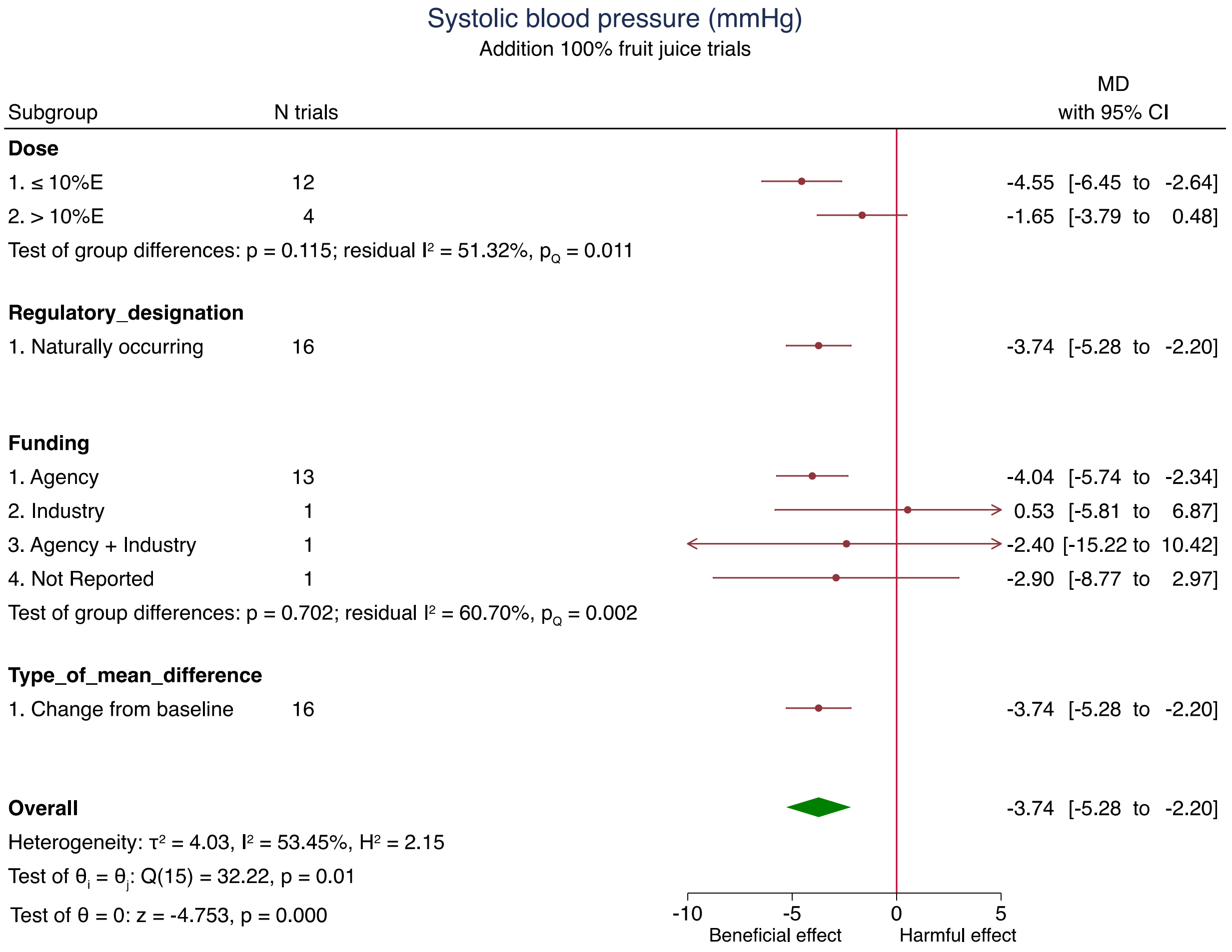

The green diamond represents the pooled estimate for the overall primary analysis of 100% fruit juice food sources of fructose-containing sugars on SBP. Within subgroup mean differences are the pooled effect estimates represented by a red circle. 95% confidence intervals are represented by the line through the circle. Data are expressed as mean differences with 95% confidence intervals using the generic inverse-variance method and random effects DerSimonian-Laird model. Inter-study heterogeneity was assessed using the Cochrane Q statistic and quantified using the I^2 statistic, with significance set at p<0.100 and I^2≥50% considered to be evidence of substantial heterogeneity. p<0.050 indicates that the effect size differed between levels of the subgroup. %E=percent of total daily energy intake; CI=confidence interval.

S68 Fig. Risk of bias (using The Cochrane Collaboration Tool) subgroup analysis for the effect of 100% fruit juice on SBP (mmHg) in addition trials.

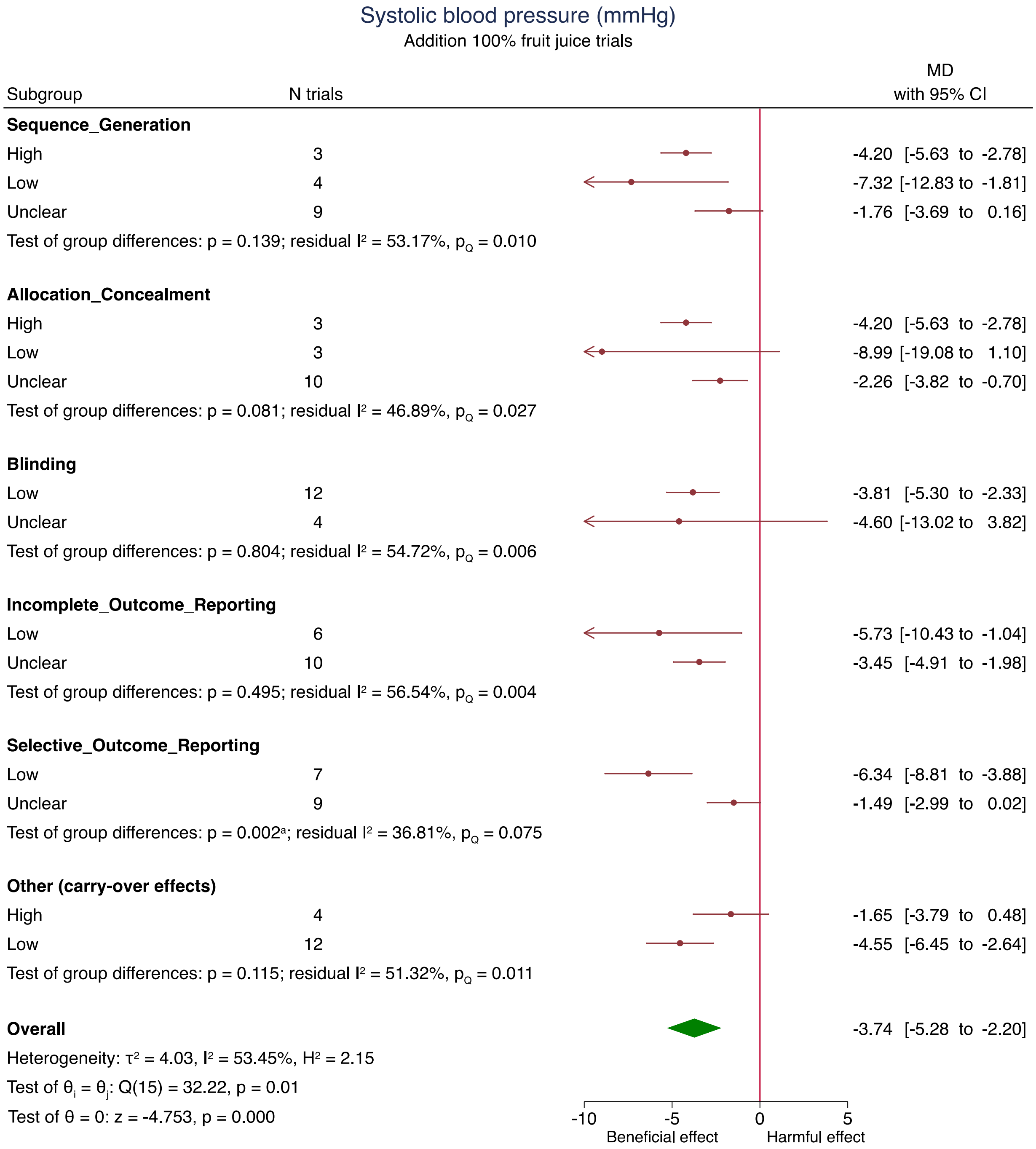

[a]Pairwise between-subgroup mean differences (95% confidence intervals) for selective outcome reporting use were as follows: (unclear vs. low) 4.22 mmHg (1.53, 6.91 mmHg).
The green diamond represents the pooled estimate for the overall primary analysis of 100% fruit juice food sources of fructose-containing sugars on SBP. Within subgroup mean differences are the pooled effect estimates represented by a red circle. 95% confidence intervals are represented by the line through the circle. Data are

expressed as mean differences with 95% confidence intervals using the generic inverse-variance method and random effects DerSimonian-Laird model. Inter-study heterogeneity was assessed using the Cochrane Q statistic and quantified using the I^2 statistic, with significance set at $p<0.100$ and $I^2 \geq 50\%$ considered to be evidence of substantial heterogeneity. $p<0.050$ indicates that the effect size differed between levels of the subgroup. CI=confidence interval.

S69 Fig (part 1 of 3). Subgroup analyses for the effect of fruit on SBP (mmHg) in addition trials.

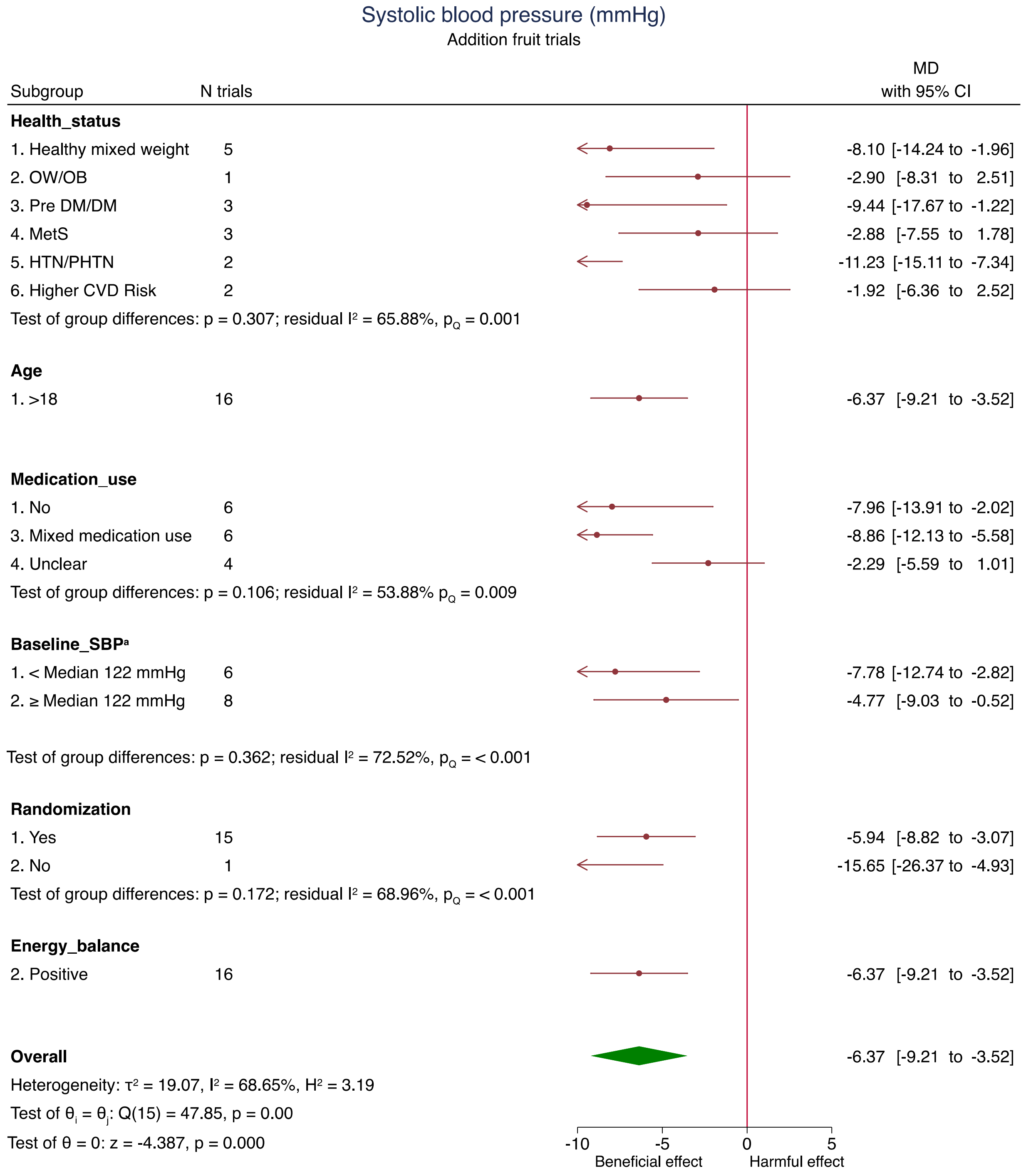

[a]N=2 trials missing baseline SBP data.

The green diamond represents the pooled estimate for the overall primary analysis of fruit food sources of fructose-containing sugars on SBP. Within subgroup mean differences are the pooled effect estimates represented by a red circle. 95% confidence intervals are represented by the line through the circle. Data are expressed as mean differences with 95% confidence intervals using the generic inverse-variance method and random effects DerSimonian-Laird model. Inter-study heterogeneity was assessed using the Cochrane Q statistic and quantified

using the I^2 statistic, with significance set at p<0.100 and $I^2 \geq 50\%$ considered to be evidence of substantial heterogeneity. p<0.050 indicates that the effect size differed between levels of the subgroup.
CI=confidence interval; HTN=hypertensive; MetS=metabolic syndrome; OB=obese; OW=overweight; PHTN=pre-hypertensive.

S69 Fig (part 2 of 3). Subgroup analyses for the effect of fruit on SBP (mmHg) in addition trials.

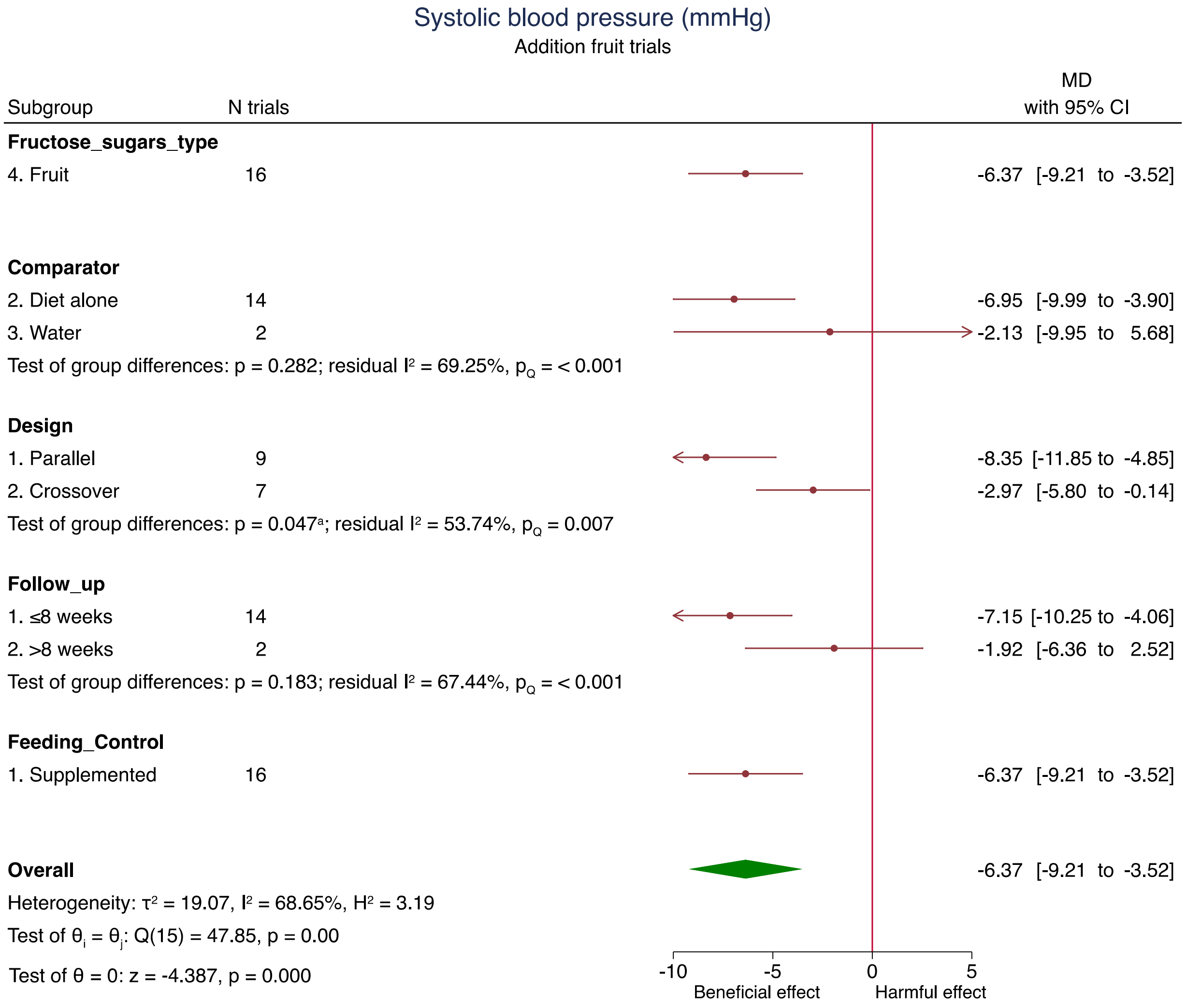

[a]Pairwise between-subgroup mean differences (95% confidence intervals) for design were as follows: (2 vs. 1) 4.92 mmHg (0.0758, 9.76 mmHg).

The green diamond represents the pooled estimate for the overall primary analysis of fruit food sources of fructose-containing sugars on SBP. Within subgroup mean differences are the pooled effect estimates represented by a red circle. 95% confidence intervals are represented by the line through the circle. Data are expressed as mean differences with 95% confidence intervals using the generic inverse-variance method and random effects DerSimonian-Laird model. Inter-study heterogeneity was assessed using the Cochrane Q statistic and quantified using the I^2 statistic, with significance set at p<0.100 and $I^2 \geq 50\%$ considered to be evidence of substantial heterogeneity. p<0.050 indicates that the effect size differed between levels of the subgroup. CI=confidence interval.

S69 Fig (part 3 of 3). Subgroup analyses for the effect of fruit on SBP (mmHg) in addition trials.

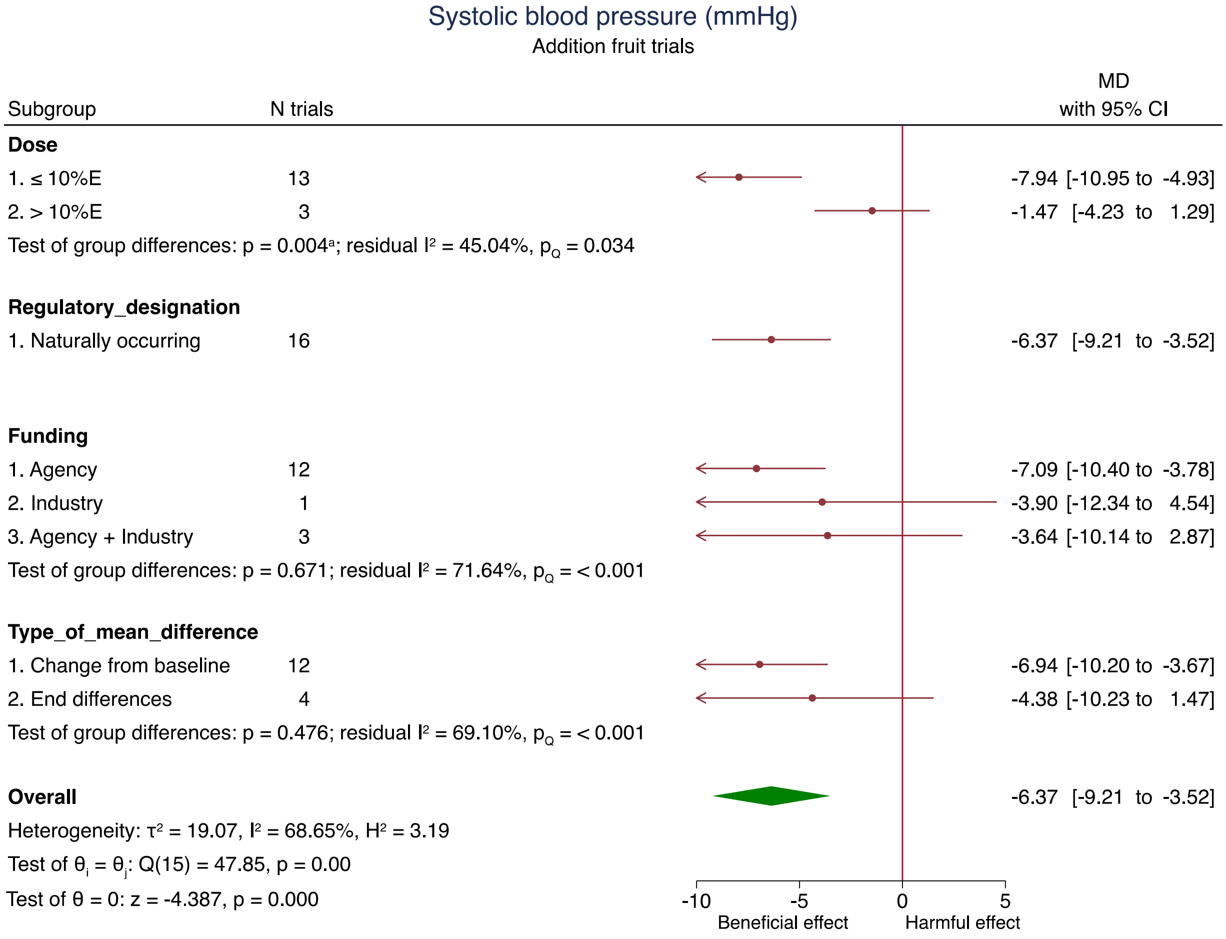

[a]Pairwise between-subgroup mean differences (95% confidence intervals) for dose were as follows: (2 vs. 1) 7.23 mmHg (2.26, 12.2 mmHg).
The green diamond represents the pooled estimate for the overall primary analysis of fruit food sources of fructose-containing sugars on SBP. Within subgroup mean differences are the pooled effect estimates represented by a red circle. 95% confidence intervals are represented by the line through the circle. Data are expressed as mean differences with 95% confidence intervals using the generic inverse-variance method and random effects DerSimonian-Laird model. Inter-study heterogeneity was assessed using the Cochrane Q statistic and quantified using the I^2 statistic, with significance set at p<0.100 and I^2≥50% considered to be evidence of substantial heterogeneity. p<0.050 indicates that the effect size differed between levels of the subgroup.
%E=percent of total daily energy intake; CI=confidence interval.

S70 Fig. Risk of bias (using The Cochrane Collaboration Tool) subgroup analysis for the effect of fruit on SBP (mmHg) in addition trials.

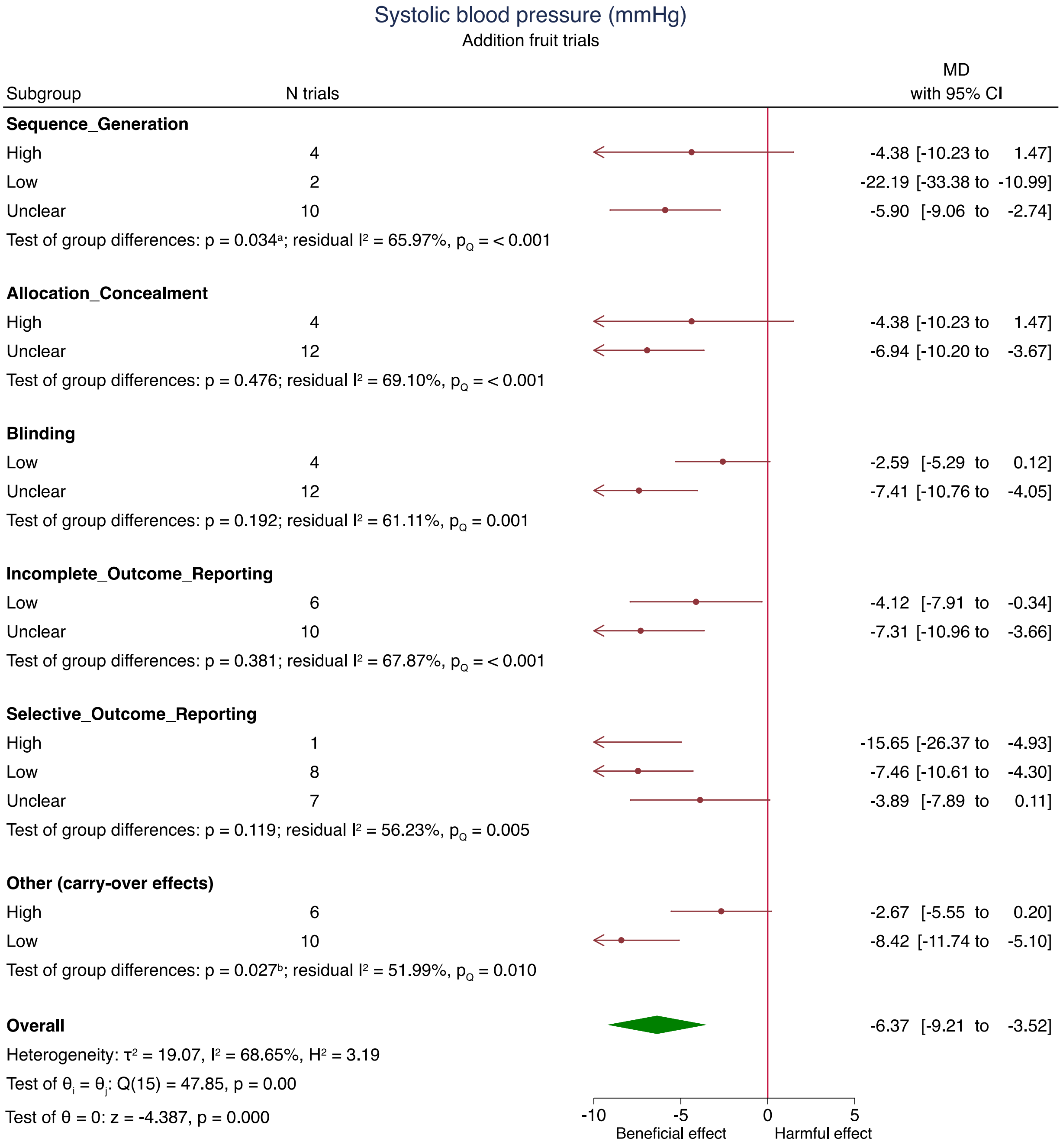

[a]Pairwise between-subgroup mean differences (95% confidence intervals) for sequence generation were as follows: (low vs. high) -18 mmHg (-31.8, -4.17 mmHg); (unclear vs. high) -1.55 mmHg (-8.09, 4.99 mmHg); (unclear vs. low) 16.4 mmHg (3.49, 29.4 mmHg).

[b]Pairwise between-subgroup mean differences (95% confidence intervals) for other were as follows: (low vs. high) -5.42 mmHg (-10.2, -0.629 mmHg).

The green diamond represents the pooled estimate for the overall primary analysis of fruit food sources of fructose-containing sugars on SBP. Within subgroup mean differences are the pooled effect estimates represented by a red circle. 95% confidence intervals are represented by the line through the circle. Data are expressed as

mean differences with 95% confidence intervals using the generic inverse-variance method and random effects DerSimonian-Laird model. Inter-study heterogeneity was assessed using the Cochrane Q statistic and quantified using the I^2 statistic, with significance set at $p<0.100$ and $I^2 \geq 50\%$ considered to be evidence of substantial heterogeneity. $p<0.050$ indicates that the effect size differed between levels of the subgroup.
CI=confidence interval.

S71 Fig (part 1 of 3). Subgroup analyses for the effect of SSBs on DBP (mmHg) in addition trials.

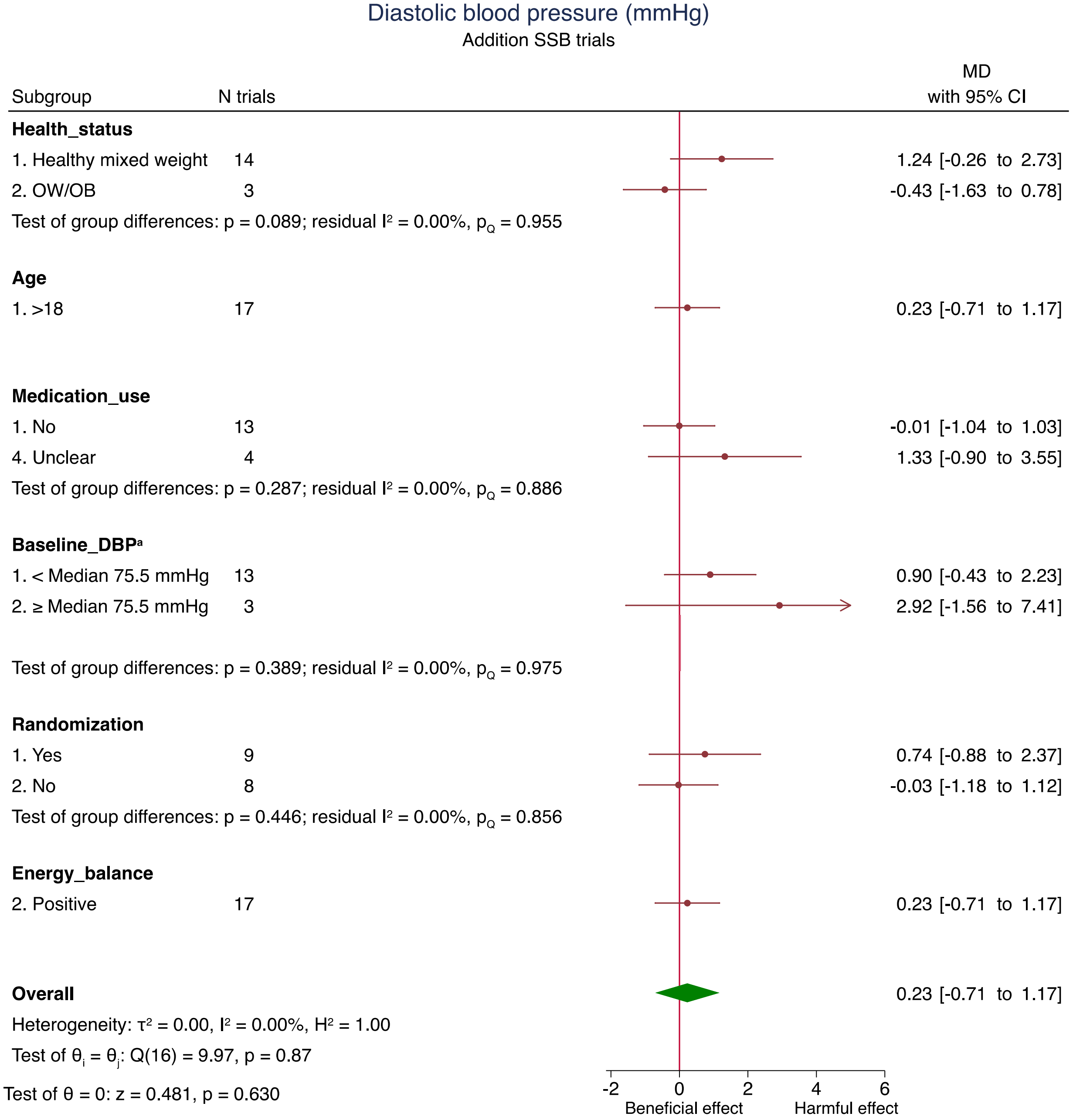

[a]N=1 trial missing baseline DBP data.

The green diamond represents the pooled estimate for the overall primary analysis of SSB food sources of fructose-containing sugars on DBP. Within subgroup mean differences are the pooled effect estimates represented by a red circle. 95% confidence intervals are represented by the line through the circle. Data are expressed as mean differences with 95% confidence intervals using the generic inverse-variance method and random effects DerSimonian-Laird model. Inter-study heterogeneity was assessed using the Cochrane Q statistic and quantified using the I^2 statistic, with significance set at p<0.100 and I^2≥50% considered to be evidence of substantial heterogeneity. p<0.050 indicates that the effect size differed between levels of the subgroup.

CI=confidence interval; OB=obese; OW=overweight.

S71 Fig (part 2 of 3). Subgroup analyses for the effect of SSBs on DBP (mmHg) in addition trials.

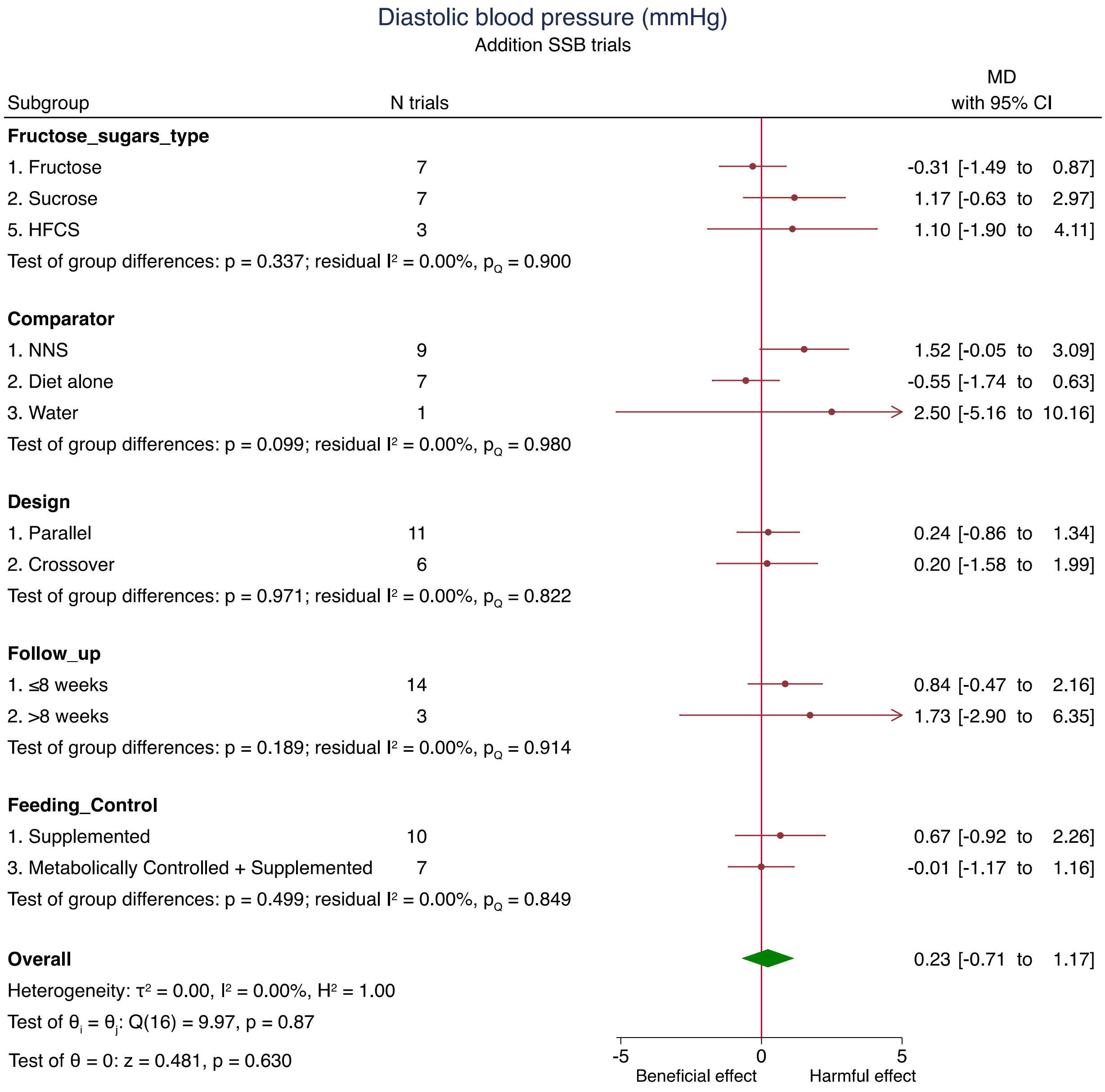

The green diamond represents the pooled estimate for the overall primary analysis of SSB food sources of fructose-containing sugars on DBP. Within subgroup mean differences are the pooled effect estimates represented by a red circle. 95% confidence intervals are represented by the line through the circle. Data are expressed as mean differences with 95% confidence intervals using the generic inverse-variance method and random effects DerSimonian-Laird model. Inter-study heterogeneity was assessed using the Cochrane Q statistic and quantified using the I^2 statistic, with significance set at p<0.100 and I^2≥50% considered to be evidence of substantial heterogeneity. p<0.050 indicates that the effect size differed between levels of the subgroup. CI=confidence interval; HFCS=high fructose corn syrup.

S71 Fig (part 3 of 3). Subgroup analyses for the effect of SSBs on DBP (mmHg) in addition trials.

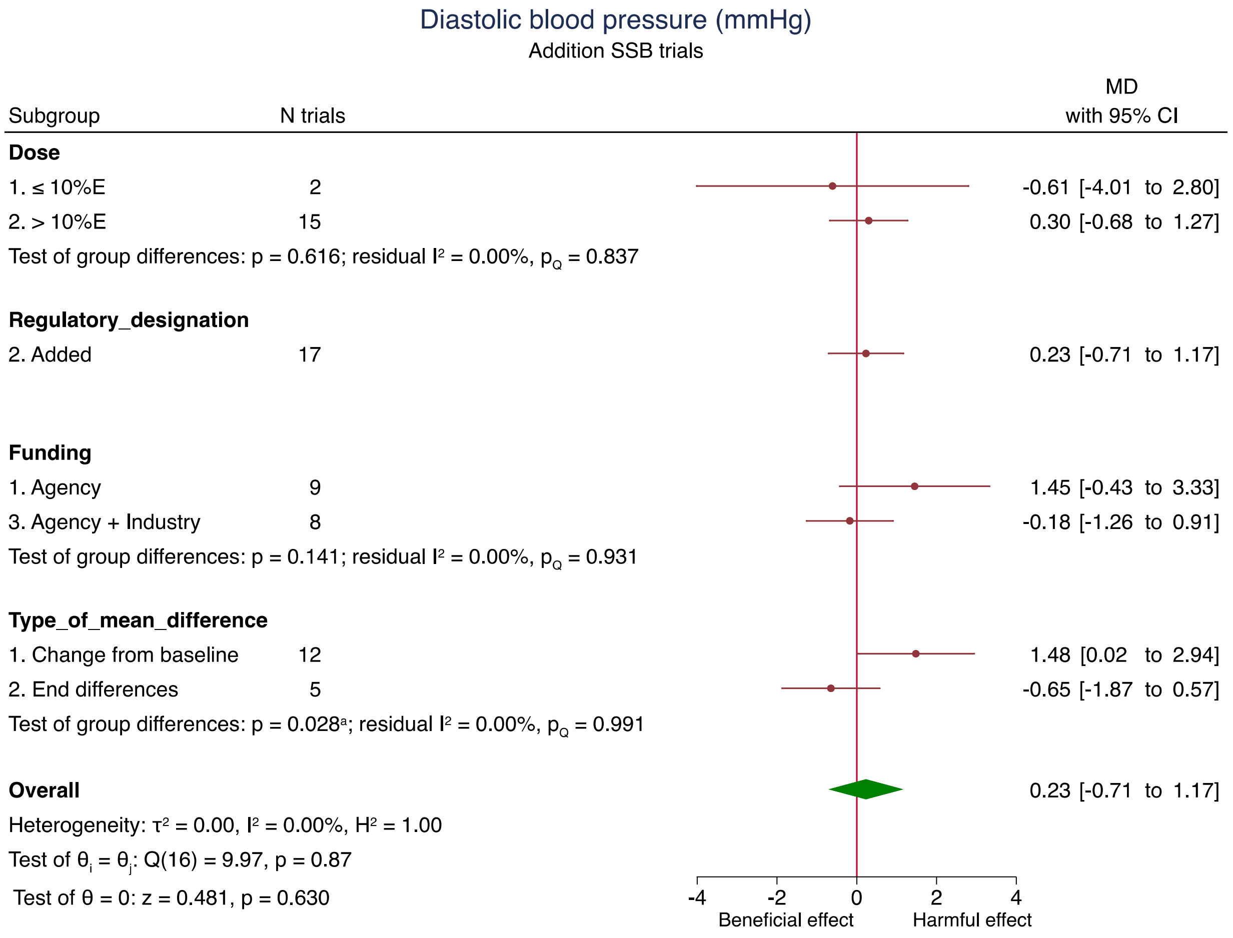

[a]Pairwise between-subgroup mean differences (95% confidence intervals) for type of mean difference were as follows: (2 vs. 1) -2.13 mmHg (-4.04, -0.226 mmHg).
The green diamond represents the pooled estimate for the overall primary analysis of SSB food sources of fructose-containing sugars on DBP. Within subgroup mean differences are the pooled effect estimates represented by a red circle. 95% confidence intervals are represented by the line through the circle. Data are expressed as mean differences with 95% confidence intervals using the generic inverse-variance method and random effects DerSimonian-Laird model. Inter-study heterogeneity was assessed using the Cochrane Q statistic and quantified using the I^2 statistic, with significance set at p<0.100 and $I^2 \geq 50\%$ considered to be evidence of substantial heterogeneity. p<0.050 indicates that the effect size differed between levels of the subgroup.
%E=percent of total daily energy intake; CI=confidence interval.

S72 Fig. Risk of bias (using The Cochrane Collaboration Tool) subgroup analysis for the effect of SSBs on DBP (mmHg) in addition trials.

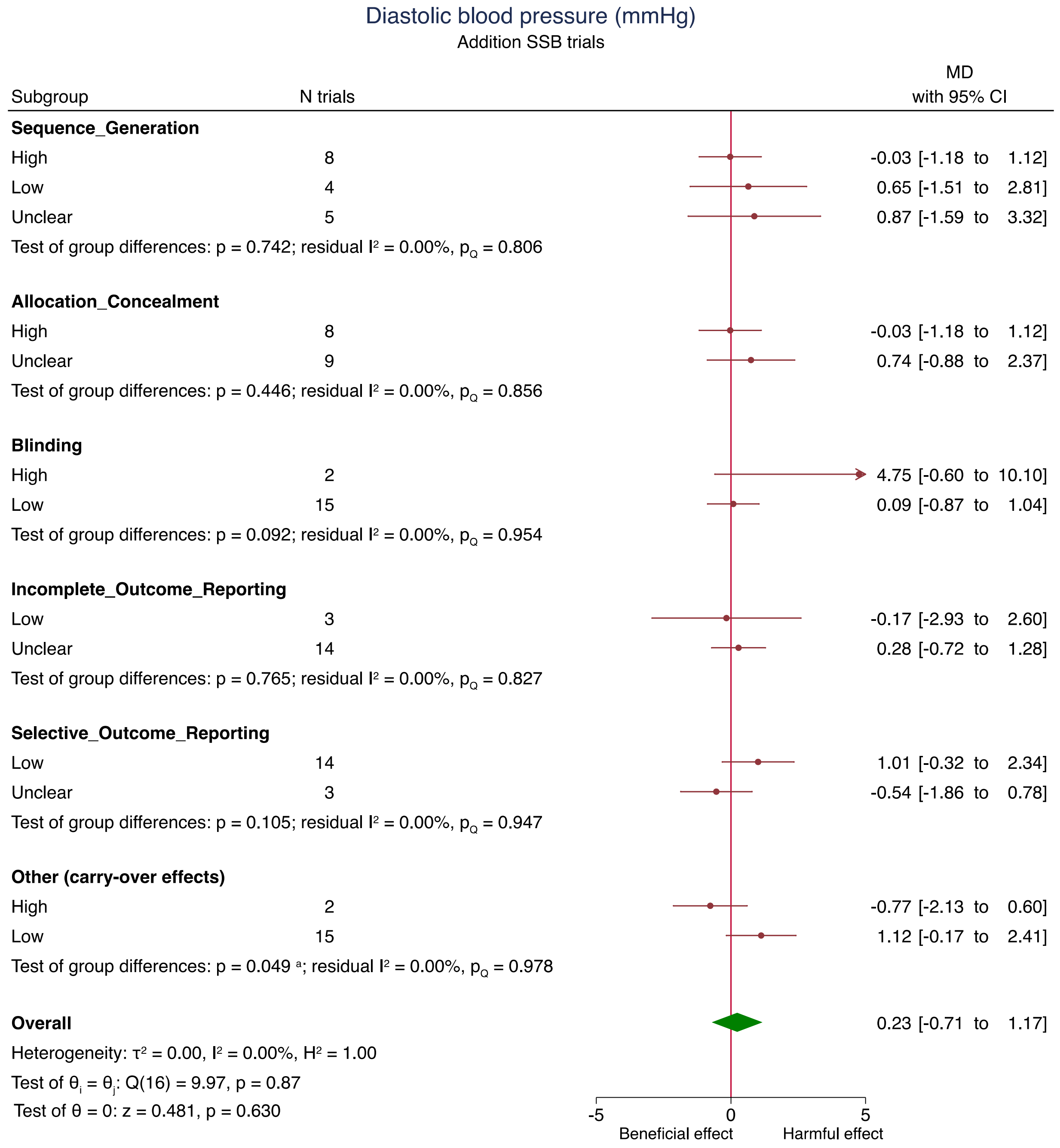

[a]Pairwise between-subgroup mean differences (95% confidence intervals) for other were as follows: (low vs. high) 1.89 mmHg (0.0108, 3.77 mmHg).

The green diamond represents the pooled estimate for the overall primary analysis of SSB food sources of fructose-containing sugars on DBP. Within subgroup mean differences are the pooled effect estimates represented by a red circle. 95% confidence intervals are represented by the line through the circle. Data are expressed as mean differences with 95% confidence intervals using the generic inverse-variance method and random effects DerSimonian-Laird model. Inter-study heterogeneity was assessed using the Cochrane Q statistic and quantified

using the I^2 statistic, with significance set at $p<0.100$ and $I^2 \geq 50\%$ considered to be evidence of substantial heterogeneity. $p<0.050$ indicates that the effect size differed between levels of the subgroup. CI=confidence interval.

S73 Fig (part 1 of 3). Subgroup analyses for the effect of 100% fruit juice on DBP (mmHg) in addition trials.

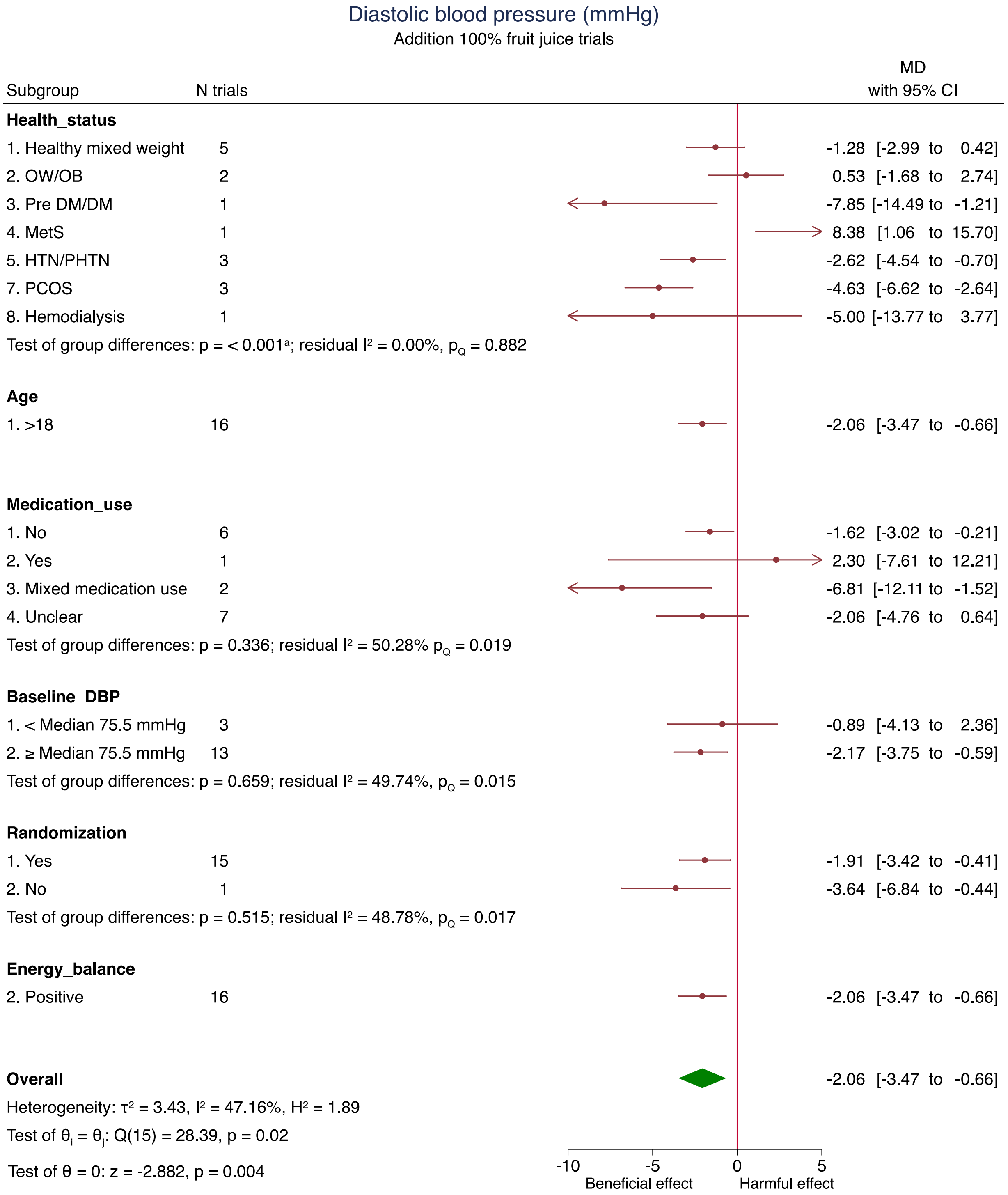

[a]Pairwise between-subgroup mean differences (95% confidence intervals) for health status were as follows: (2 vs. 1) 1.81 mmHg (-0.978, 4.61 mmHg); (3 vs. 1) -6.57 mmHg (-13.4, 0.294 mmHg); (4 vs. 1) 9.66 mmHg (2.15, 17.2 mmHg); (5 vs. 1) -1.34 mmHg (-3.91, 1.23 mmHg); (7 vs. 1) -3.35 mmHg (-5.97, -0.721 mmHg); (8 vs. 1) -3.72 mmHg

(-12.6, 5.22 mmHg); (3 vs. 2) -8.38 mmHg (-15.4, -1.38 mmHg); (4 vs. 2) 7.85 mmHg (0.206, 15.5 mmHg); (5 vs. 2) -3.15 mmHg (-6.08, -0.226 mmHg); (7 vs. 2) -5.16 mmHg (-8.13, -2.19 mmHg); (8 vs. 2) -5.53 mmHg (-14.6, 3.51 mmHg); (4 vs. 3) 16.2 mmHg (6.35, 26.1 mmHg); (5 vs. 3) 5.23 mmHg (-1.69, 12.1 mmHg); (7 vs. 3) 3.22 mmHg (-3.72, 10.2 mmHg); (5 vs. 4) -11 mmHg (-18.6, -3.44 mmHg); (7 vs. 4) -13 mmHg (-20.6, -5.46 mmHg); (8 vs. 4) -13.4 mmHg (-24.8, -1.96 mmHg); (7 vs. 5) -2.01 mmHg (-4.78, 0.76 mmHg); (8 vs. 5) -2.38 mmHg (-11.4, 6.6 mmHg); (8 vs. 7) -0.37 mmHg (-9.36, 8.62 mmHg).

The green diamond represents the pooled estimate for the overall primary analysis of 100% fruit juice food sources of fructose-containing sugars on DBP. Within subgroup mean differences are the pooled effect estimates represented by a red circle. 95% confidence intervals are represented by the line through the circle. Data are expressed as mean differences with 95% confidence intervals using the generic inverse-variance method and random effects DerSimonian-Laird model. Inter-study heterogeneity was assessed using the Cochrane Q statistic and quantified using the I^2 statistic, with significance set at $p<0.100$ and $I^2 \geq 50\%$ considered to be evidence of substantial heterogeneity. $p<0.050$ indicates that the effect size differed between levels of the subgroup. CI=confidence interval; HTN=hypertensive; OB=obese; OW=overweight; PHTN=pre-hypertensive.

S73 Fig (part 2 of 3). Subgroup analyses for the effect of 100% fruit juice on DBP (mmHg) in addition trials.

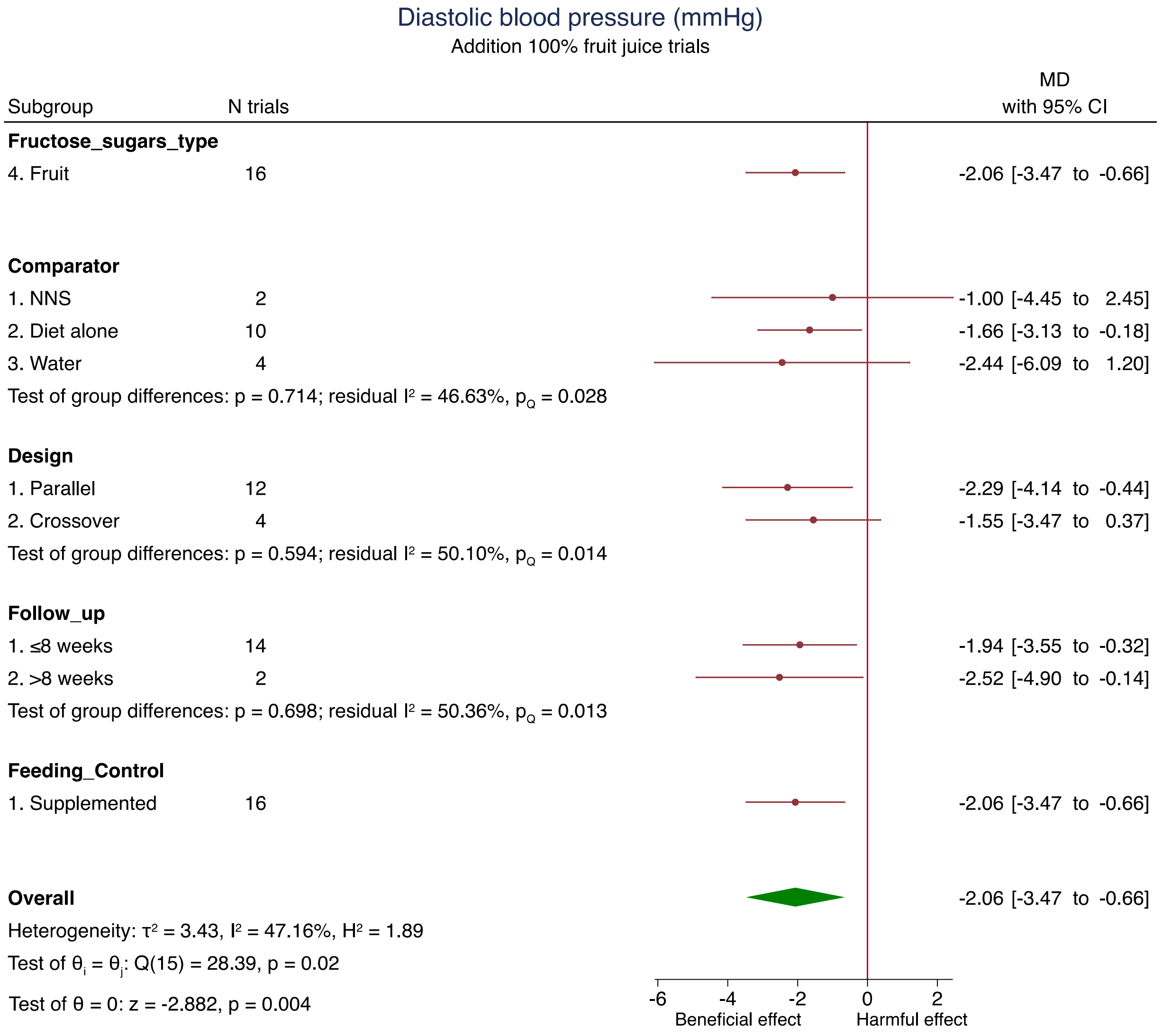

The green diamond represents the pooled estimate for the overall primary analysis of 100% fruit juice food sources of fructose-containing sugars on DBP. Within subgroup mean differences are the pooled effect estimates represented by a red circle. 95% confidence intervals are represented by the line through the circle. Data are expressed as mean differences with 95% confidence intervals using the generic inverse-variance method and random effects DerSimonian-Laird model. Inter-study heterogeneity was assessed using the Cochrane Q statistic and quantified using the I^2 statistic, with significance set at p<0.100 and $I^2 \geq 50\%$ considered to be evidence of substantial heterogeneity. p<0.050 indicates that the effect size differed between levels of the subgroup. CI=confidence interval; NNS=non-nutritive sweetener.

S73 Fig (part 3 of 3). Subgroup analyses for the effect of 100% fruit juice on DBP (mmHg) in addition trials.

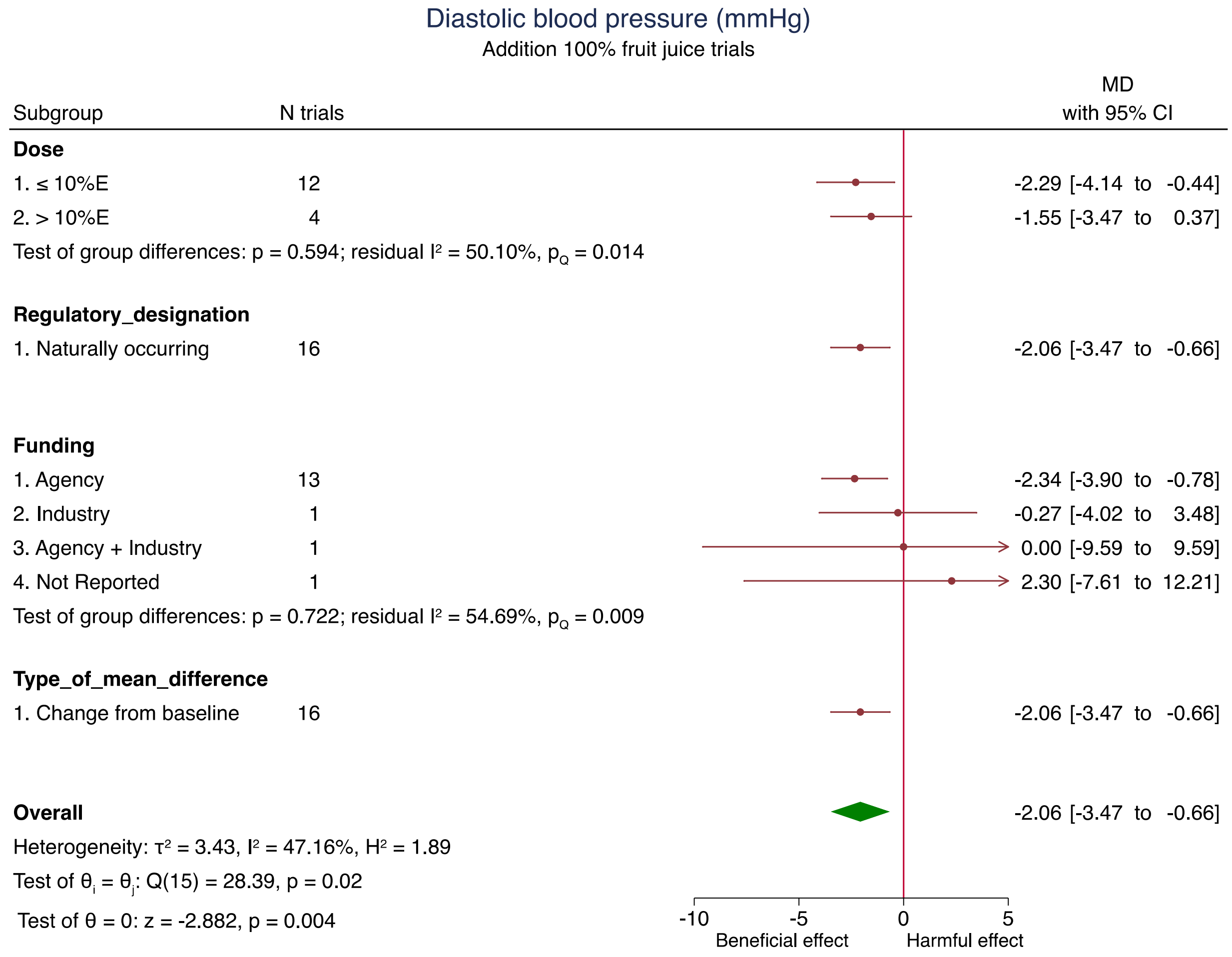

The green diamond represents the pooled estimate for the overall primary analysis of 100% fruit juice of food sources of fructose-containing sugars on DBP. Within subgroup mean differences are the pooled effect estimates represented by a red circle. 95% confidence intervals are represented by the line through the circle. Data are expressed as mean differences with 95% confidence intervals using the generic inverse-variance method and random effects DerSimonian-Laird model. Inter-study heterogeneity was assessed using the Cochrane Q statistic and quantified using the I^2 statistic, with significance set at p<0.100 and $I^2 \geq 50\%$ considered to be evidence of substantial heterogeneity. p<0.050 indicates that the effect size differed between levels of the subgroup. %E=percent of total daily energy intake; CI=confidence interval.

S74 Fig. Risk of bias (using The Cochrane Collaboration Tool) subgroup analysis for the effect of 100% fruit juice on DBP (mmHg) in addition trials.

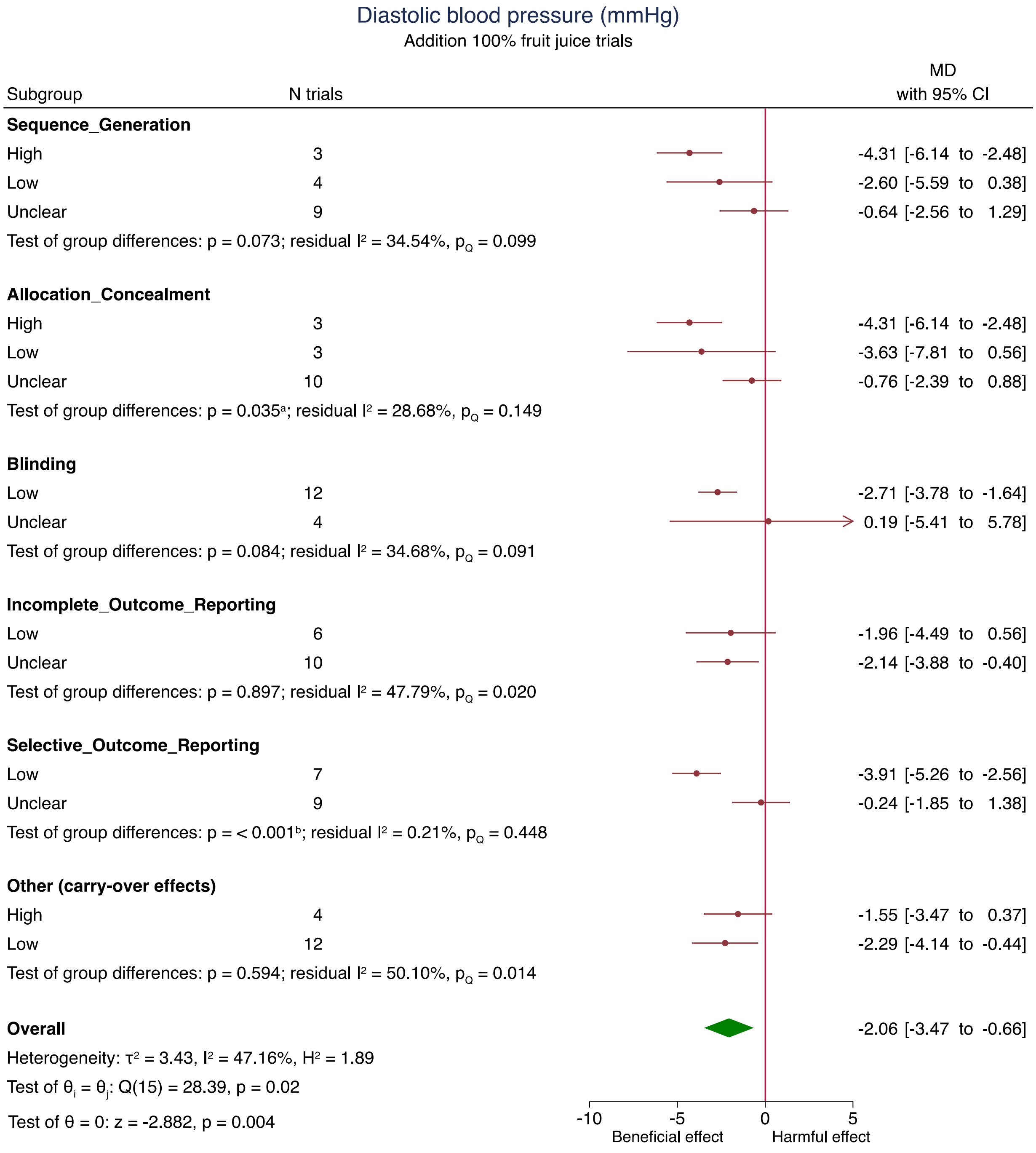

[a]Pairwise between-subgroup mean differences (95% confidence intervals) for allocation concealment were as follows: (low vs. high) 1.1 mmHg (-2.74, 4.93 mmHg); (unclear vs. high) 3.53 mmHg (0.73, 6.43 mmHg); (unclear vs. low) 2.44 mmHg (-0.986, 5.86 mmHg).

[a]Pairwise between-subgroup mean differences (95% confidence intervals) for selective outcome reporting were as follows: (unclear vs. low) 3.65 mmHg (1.76, 5.54 mmHg).

The green diamond represents the pooled estimate for the overall primary analysis of 100% fruit juice food sources of fructose-containing sugars on DBP. Within subgroup mean differences are the pooled effect estimates represented by a red circle. 95% confidence intervals are represented by the line through the circle. Data are expressed as mean differences with 95% confidence intervals using the generic inverse-variance method and random effects DerSimonian-Laird model. Inter-study heterogeneity was assessed using the Cochrane Q statistic and quantified using the I^2 statistic, with significance set at $p<0.100$ and $I^2 \geq 50\%$ considered to be evidence of substantial heterogeneity. $p<0.050$ indicates that the effect size differed between levels of the subgroup. CI=confidence interval.

S75 Fig (part 1 of 3). Subgroup analyses for the effect of fruit on DBP (mmHg) in addition trials.

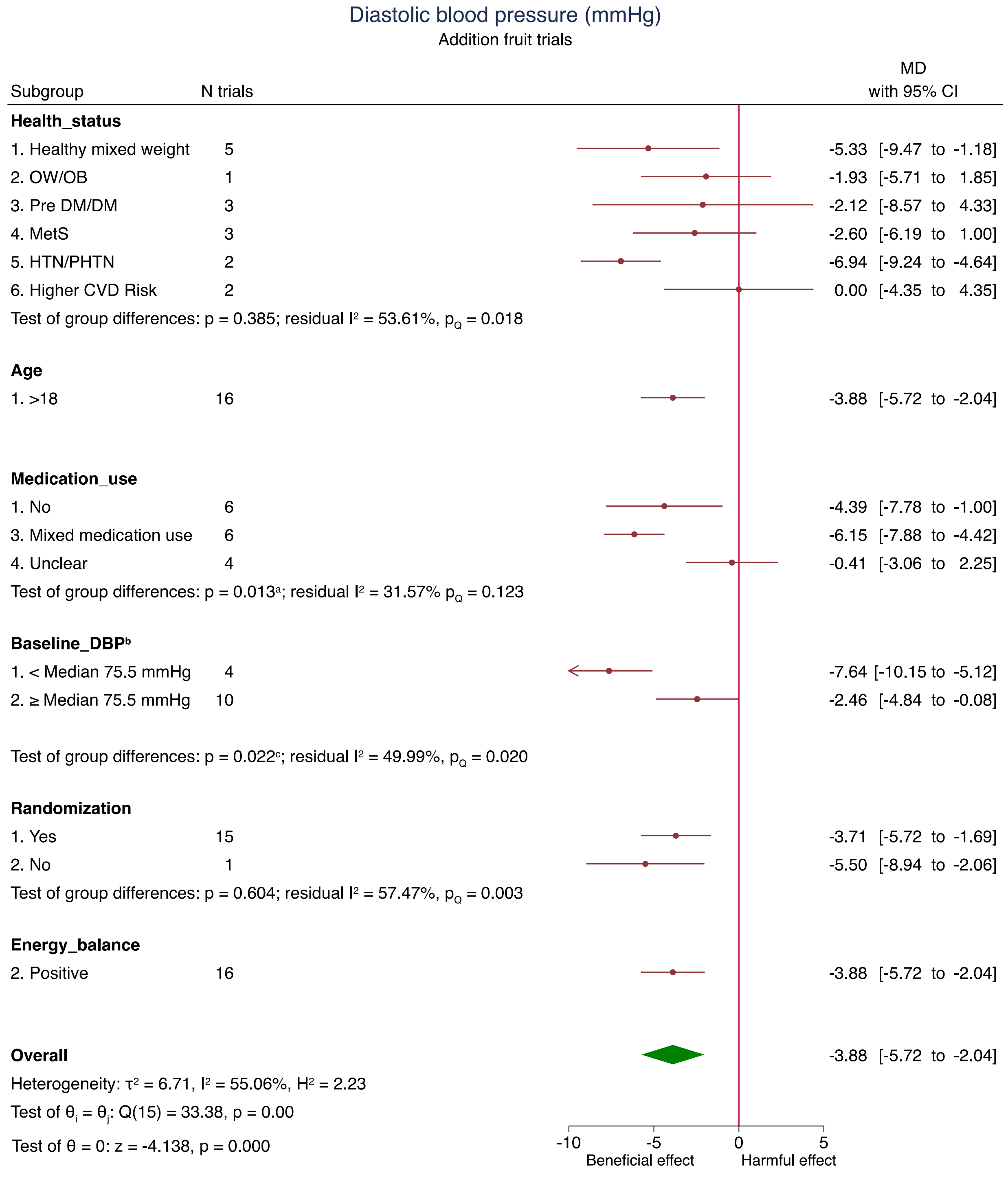

[a]Pairwise between-subgroup mean differences (95% confidence intervals) for medication use were as follows: (3 vs. 1) -2.02 mmHg (-5.4, 1.36 mmHg); (4 vs. 1) 3.8 mmHg (-0.294, 7.9 mmHg); (4 vs. 3) 5.82 mmHg (1.93, 9.71 mmHg).

[b]N=2 trials missing data for baseline DBP.
[c]Pairwise between-subgroup mean differences (95% confidence intervals) for baseline DBP were as follows: (2 vs. 1) 5.55 mmHg (0.805, 10.3 mmHg).
The green diamond represents the pooled estimate for the overall primary analysis of fruit food sources of fructose-containing sugars on SBP. Within subgroup mean differences are the pooled effect estimates represented by a red circle. 95% confidence intervals are represented by the line through the circle. Data are expressed as mean differences with 95% confidence intervals using the generic inverse-variance method and random effects DerSimonian-Laird model. Inter-study heterogeneity was assessed using the Cochrane Q statistic and quantified using the I^2 statistic, with significance set at $p<0.100$ and $I^2 \geq 50\%$ considered to be evidence of substantial heterogeneity. $p<0.050$ indicates that the effect size differed between levels of the subgroup.
CI=confidence interval; HTN=hypertensive; MetS=metabolic syndrome; OB=obese; OW=overweight; PHTN=pre-hypertensive.

S75 Fig (part 2 of 3). Subgroup analyses for the effect of fruit on DBP (mmHg) in addition trials.

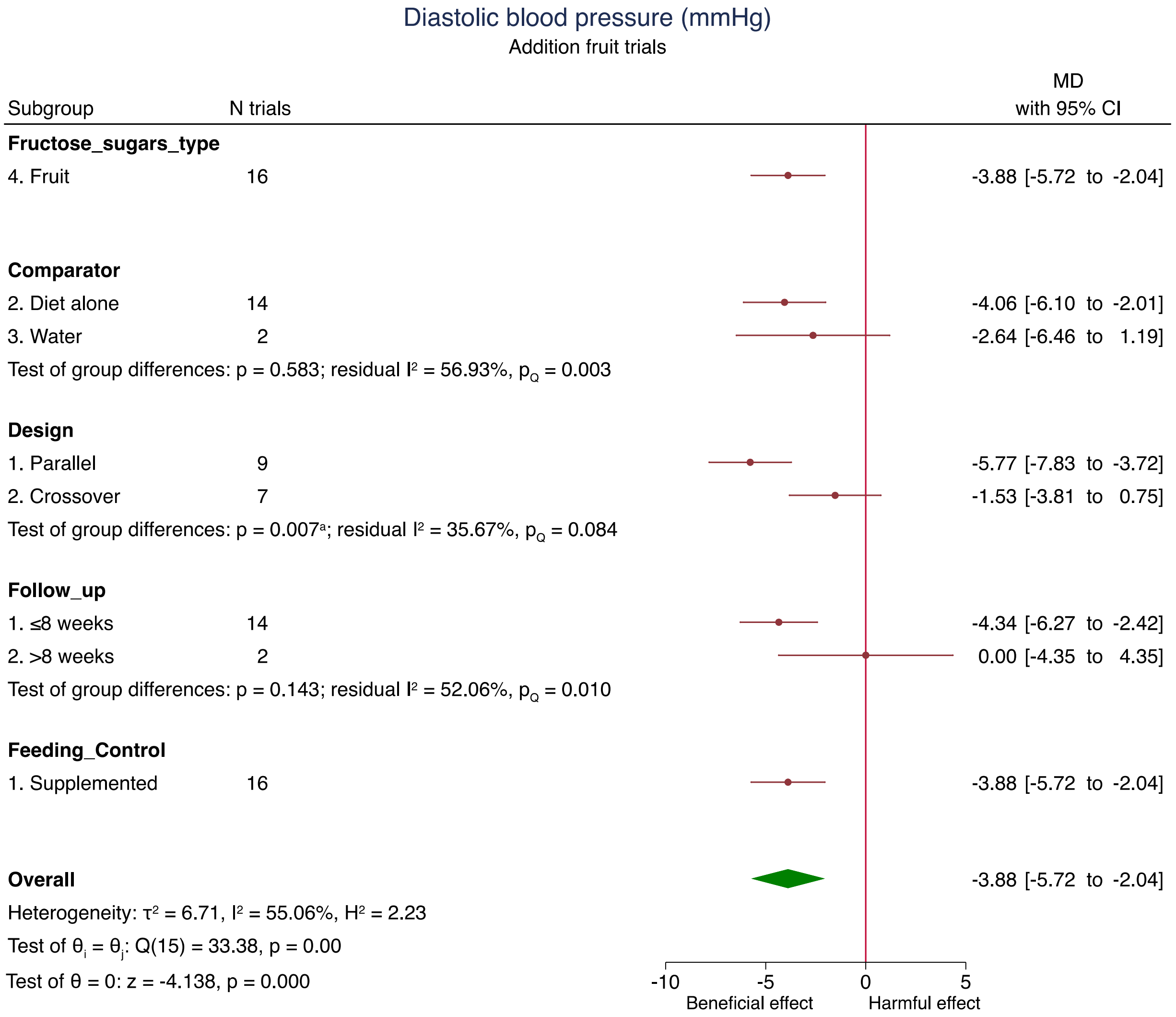

[a]Pairwise between-subgroup mean differences (95% confidence intervals) for design were as follows: (2 vs. 1) 4.3 mmHg (1.19, 7.4 mmHg).

The green diamond represents the pooled estimate for the overall primary analysis of fruit food sources of fructose-containing sugars on SBP. Within subgroup mean differences are the pooled effect estimates represented by a red circle. 95% confidence intervals are represented by the line through the circle. Data are expressed as mean differences with 95% confidence intervals using the generic inverse-variance method and random effects DerSimonian-Laird model. Inter-study heterogeneity was assessed using the Cochrane Q statistic and quantified using the I^2 statistic, with significance set at $p<0.100$ and $I^2 \geq 50\%$ considered to be evidence of substantial heterogeneity. $p<0.050$ indicates that the effect size differed between levels of the subgroup.

CI=confidence interval.

S75 Fig (part 3 of 3). Subgroup analyses for the effect of fruit on DBP (mmHg) in addition trials.

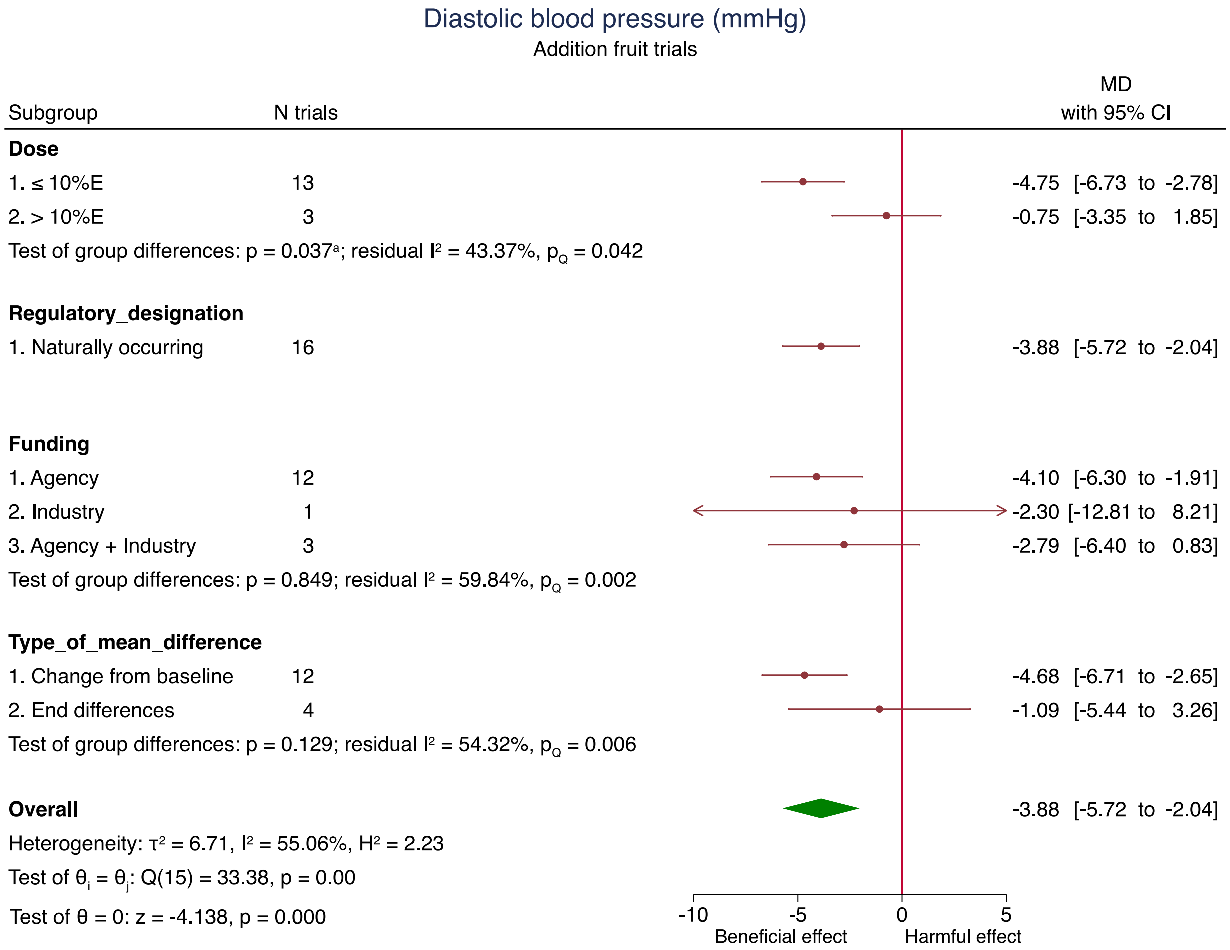

[a]Pairwise between-subgroup mean differences (95% confidence intervals) for dose were as follows: (2 vs. 1) 4.28 mmHg (0.259, 8.31 mmHg).
The green diamond represents the pooled estimate for the overall primary analysis of fruit food sources of fructose-containing sugars on SBP. Within subgroup mean differences are the pooled effect estimates represented by a red circle. 95% confidence intervals are represented by the line through the circle. Data are expressed as mean differences with 95% confidence intervals using the generic inverse-variance method and random effects DerSimonian-Laird model. Inter-study heterogeneity was assessed using the Cochrane Q statistic and quantified using the I^2 statistic, with significance set at p<0.100 and I^2≥50% considered to be evidence of substantial heterogeneity. p<0.050 indicates that the effect size differed between levels of the subgroup.
%E=percent of total daily energy intake; CI=confidence interval.

S76 Fig. Risk of bias (using The Cochrane Collaboration Tool) subgroup analysis for the effect of fruit on DBP (mmHg) in addition trials.

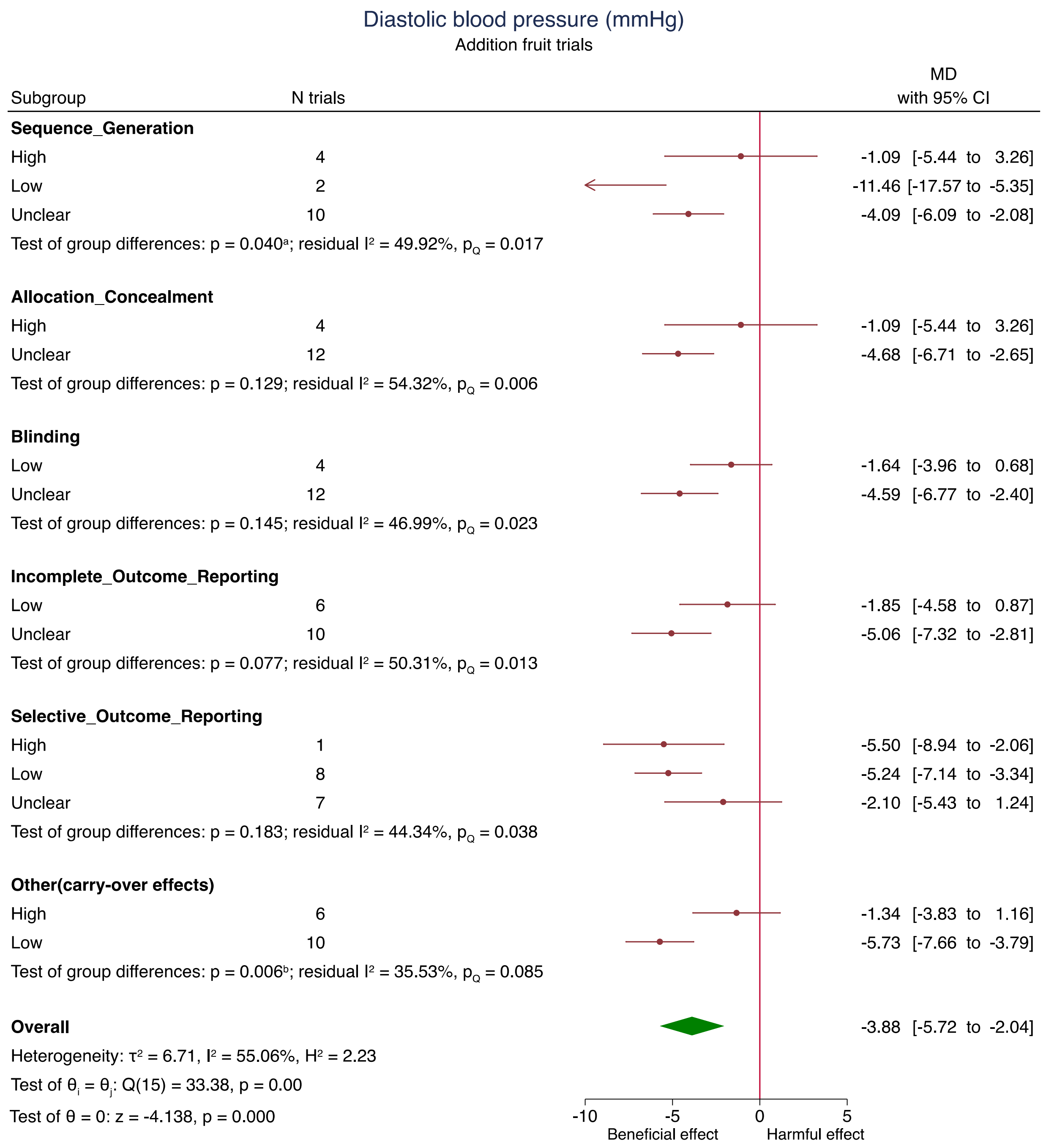

[a]Pairwise between-subgroup mean differences (95% confidence intervals) for sequence generation were as follows: (low vs. high) -10 mmHg (-17.8, -2.24 mmHg); (unclear vs. high) -2.61 mmHg (-6.73, 1.51 mmHg); (unclear vs. low) 7.4 mmHg (0.194, 14.6 mmHg)

[b]Pairwise between-subgroup mean differences (95% confidence intervals) for other were as follows: (low vs. high) -4.36 mmHg (-7.48, -1.23 mmHg).

The green diamond represents the pooled estimate for the overall primary analysis of fruit food sources of fructose-containing sugars on SBP. Within subgroup mean differences are the pooled effect estimates represented by a red circle. 95% confidence intervals are represented by the line through the circle. Data are expressed as mean differences with 95% confidence intervals using the generic inverse-variance method and random effects DerSimonian-Laird model. Inter-study heterogeneity was assessed using the Cochrane Q statistic and quantified using the I^2 statistic, with significance set at $p<0.100$ and $I^2 \geq 50\%$ considered to be evidence of substantial heterogeneity. $p<0.050$ indicates that the effect size differed between levels of the subgroup.
%E=percent of total daily energy intake; CI=confidence interval.

S77 Fig. Continuous meta-regression analysis for the effect of important food sources of fructose-containing sugars on SBP (mmHg) in substitution trials.

Continuous meta-regression
in substitution trials

Subgroup	Range	Trials	Beta [95% CI]	P_{MD}	Residual I^2 (%)	P_Q
Baseline systolic blood pressure (mmHg)[a]	104.2—166.7	71	-0.13 [-0.19 to -0.07]	<0.001	17	0.111
Follow-up (weeks)	1.0—52.1	72	-0.01 [-0.13 to 0.11]	0.923	39	0.001
Food source of fructose containing sugars dose (%E)[b]	1.2—58.0	71	-0.03 [-0.12 to 0.06]	0.560	41	<0.001
Mean age (years)[c]	7.7—63.4	70	-0.06 [-0.12 to 0.00]	0.066	37	0.001

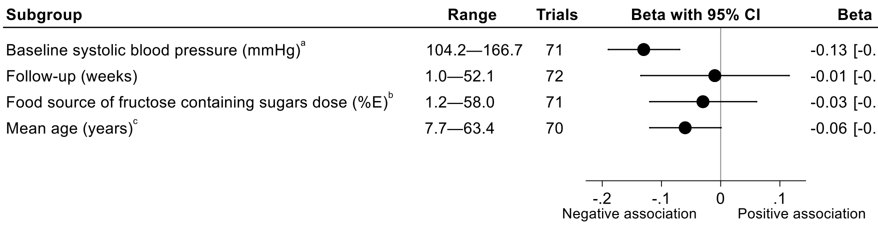

Data is presented as between group mean difference (95% confidence intervals) for a 1-unit change in the predictor variable. ß–coefficients were estimated using continuous meta-regression analysis. A positive ß-coefficient implies an increase in SBP with the food source of fructose-containing sugars intervention as the subgroup variable increases, and a negative ß-coefficient implies a decrease in SBP. Residual I2 reports inter-study heterogeneity not explained by the subgroup and was estimated using the Cochrane Q statistic.

[a]N=1 trials missing data for baseline SBP.

[b]N=1 trial missing data for dose.

[c]N=2 trials missing data for mean age.

%E=percentage of total energy intake; CI, confidence interval.

S78 Fig. Continuous meta-regression analysis for the effect of important food sources of fructose-containing sugars on SBP (mmHg) in addition trials.

Continuous meta-regression

Systolic blood pressure in addition trials

Subgroup	Range	Trials	Beta with 95% CI	Beta [95% CI]	P_{MD}	Residual I^2 (%)	P_Q
Baseline systolic blood pressure[a] (mmHg)	111.5—153.0	58		-0.19 [-0.34 to -0.04]	0.013	63	<0.001
Follow-up (weeks)	2.0—52.0	64		-0.01 [-0.20 to 0.18]	0.889	70	<0.001
Food source of fructose containing sugars dose (%E)	1.1—26.0	64		0.34 [0.21 to 0.47]	<0.001	53	<0.001
Mean age[b] (years)	21.5—66.5	62		-0.15 [-0.25 to -0.05]	0.005	69	<0.001

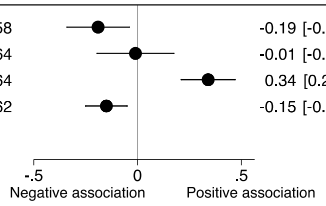

Data is presented as between group mean difference (95% confidence intervals) for a 1-unit change in the predictor variable. ß–coefficients were estimated using continuous meta-regression analysis. A positive ß-coefficient implies an increase in SBP with the food source of fructose-containing sugars intervention as the subgroup variable increases, and a negative ß-coefficient implies a decrease in SBP. Residual I2 reports inter-study heterogeneity not explained by the subgroup and was estimated using the Cochrane Q statistic.

[a]N=6 trials missing data for baseline SBP.

[b]N=2 trials missing data for mean age.

%E=percentage of total energy intake; CI, confidence interval.

S79 Fig. Continuous meta-regression analysis for the effect of important food sources of fructose-containing sugars on SBP (mmHg) in subtraction trials.

Continuous meta-regression
in subtraction trials

Subgroup	Range	Trials	Beta [95% CI]	P_{MD}	Residual I^2 (%)	P_Q
Baseline systolic blood pressure (mmHg)	101.0—127.4	10	0.00 [-0.24 to 0.24]	0.995	84	<0.001
Follow-up (weeks)	1.0—52.1	10	-0.08 [-0.20 to 0.04]	0.192	83	<0.001
Food source of fructose containing sugars dose (%E)	3.0—20.0	10	-0.11 [-0.41 to 0.19]	0.464	84	<0.001
Mean age (years)	21.7—42.3	10	-0.01 [-0.29 to 0.27]	0.966	84	<0.001

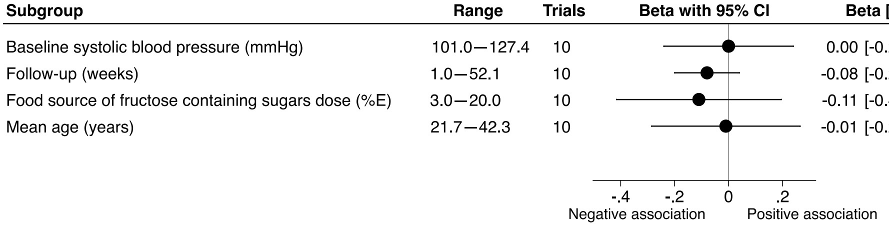

Data is presented as between group mean difference (95% confidence intervals) for a 1-unit change in the predictor variable. ß–coefficients were estimated using continuous meta-regression analysis. A positive ß-coefficient implies an increase in SBP with the food source of fructose-containing sugars intervention as the subgroup variable increases, and a negative ß-coefficient implies a decrease in SBP. Residual I2 reports inter-study heterogeneity not explained by the subgroup and was estimated using the Cochrane Q statistic.

S80 Fig. Continuous meta-regression analysis for the effect of important food sources of fructose-containing sugars on DBP (mmHg) in substitution trials.

Continuous meta-regression
in substitution trials

Subgroup	Range	Trials	Beta with 95% CI	Beta [95% CI]	P_{MD}	Residual I^2 (%)	P_Q
Baseline diastolic blood pressure (mmHg)[a]	60.0—106.8	71		-0.10 [-0.17 to -0.04]	0.003	45	<0.001
Follow-up (weeks)	1.0—52.1	72		-0.08 [-0.17 to 0.01]	0.071	52	<0.001
Food source of fructose containing sugars dose (%E)[b]	1.2—58.0	71		0.05 [-0.01 to 0.11]	0.131	57	<0.001
Mean age (years)[c]	7.7—63.4	70		-0.07 [-0.11 to -0.03]	0.003	55	<0.001

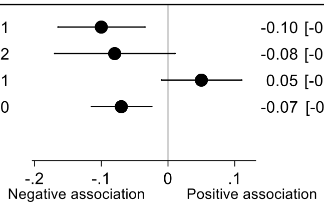

Data is presented as between group mean difference (95% confidence intervals) for a 1-unit change in the predictor variable. ß–coefficients were estimated using continuous meta-regression analysis. A positive ß-coefficient implies an increase in DBP with the food source of fructose-containing sugars intervention as the subgroup variable increases, and a negative ß-coefficient implies a decrease in DBP. Residual I2 reports inter-study heterogeneity not explained by the subgroup and was estimated using the Cochrane Q statistic.

[a]N=1 trials missing data for baseline DBP.

[b]N=1 trial missing data for dose.

[c]N=2 trials missing data for mean age.

%E=percentage of total energy intake; CI, confidence interval.

S81 Fig. Continuous meta-regression analysis for the effect of important food sources of fructose-containing sugars on DBP (mmHg) in addition trials.

Continuous meta-regression

Diastolic blood pressure in addition trials

Subgroup	Range	Trials	Beta [95% CI]	P_{MD}	Residual I^2 (%)	P_Q
Baseline diastolic blood pressure[a] (mmHg)	63.1—98.4	58	-0.19 [-0.30 to -0.08]	0.001	46	<0.001
Follow-up (weeks)	2.0—52.0	64	0.00 [-0.13 to 0.13]	0.986	59	<0.001
Food source of fructose containing sugars dose (%E)	1.0—26.0	64	0.24 [0.13 to 0.34]	<0.001	50	<0.001
Mean age[b] (years)	21.5—66.5	62	-0.06 [-0.13 to 0.01]	0.117	60	<0.001

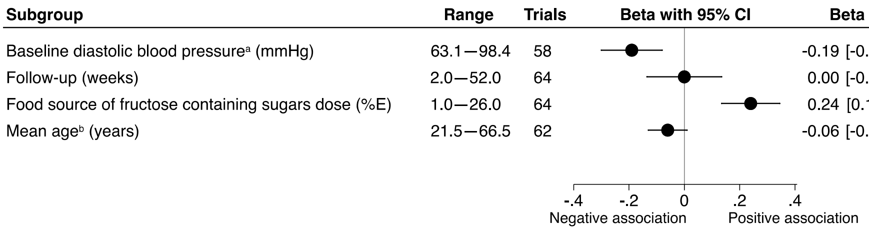

Data is presented as between group mean difference (95% confidence intervals) for a 1-unit change in the predictor variable. ß–coefficients were estimated using continuous meta-regression analysis. A positive ß-coefficient implies an increase in DBP with the food source of fructose-containing sugars intervention as the subgroup variable increases, and a negative ß-coefficient implies a decrease in DBP. Residual I2 reports inter-study heterogeneity not explained by the subgroup and was estimated using the Cochrane Q statistic.

[a]N=6 trials missing data for baseline DBP.

[b]N=2 trials missing data for mean age.

%E=percentage of total energy intake; CI, confidence interval.

S82 Fig. Continuous meta-regression analysis for the effect of important food sources of fructose-containing sugars on DBP (mmHg) in subtraction trials.

Continuous meta-regression

Diastolic blood pressure in subtraction trials

Subgroup	Range	Trials	Beta [95% CI]	P_{MD}	Residual I² (%)	P_Q
Baseline diastolic blood pressure (mmHg)	66.6—81.6	10	-0.06 [-0.28 to 0.16]	0.593	65	0.004
Follow-up (weeks)	1.0—52.1	10	-0.07 [-0.16 to 0.02]	0.137	66	0.003
Food source of fructose containing sugars dose (%E)	3.0—20.0	10	-0.08 [-0.24 to 0.09]	0.311	66	0.003
Mean age (years)	21.7—42.3	10	-0.05 [-0.19 to 0.09]	0.498	66	0.003

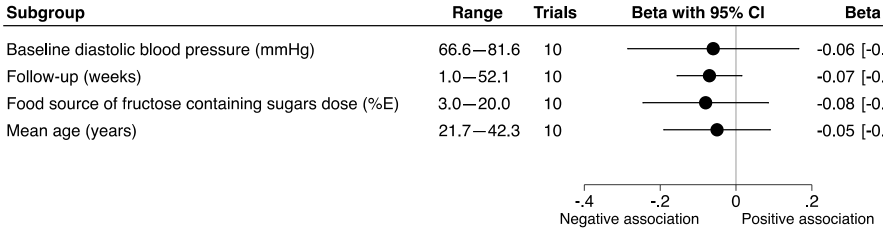

Data is presented as between group mean difference (95% confidence intervals) for a 1-unit change in the predictor variable. ß–coefficients were estimated using continuous meta-regression analysis. A positive ß-coefficient implies an increase in DBP with the food source of fructose-containing sugars intervention as the subgroup variable increases, and a negative ß-coefficient implies a decrease in DBP. Residual I2 reports inter-study heterogeneity not explained by the subgroup and was estimated using the Cochrane Q statistic.
%E=percentage of total energy intake; CI, confidence interval.

S83 Fig. Continuous meta-regression analysis for the effect of SSBs on SBP (mmHg) in substitution trials.

Continuous meta-regression
in substitution SSB trials

Subgroup	Range	Trials	Beta [95% CI]	P_{MD}	Residual I^2 (%)	P_Q
Baseline systolic blood pressure (mmHg)	114.7—139.5	16	0.14 [-0.12 to 0.41]	0.323	6	0.385
Follow-up (weeks)	2.0—26.1	16	0.20 [-0.11 to 0.51]	0.221	1	0.438
Food source of fructose containing sugars dose (%E)	6.9—25.0	16	-0.01 [-0.22 to 0.20]	0.949	10	0.339
Mean age (years)	15.3—61.0	16	0.01 [-0.13 to 0.15]	0.897	10	0.341

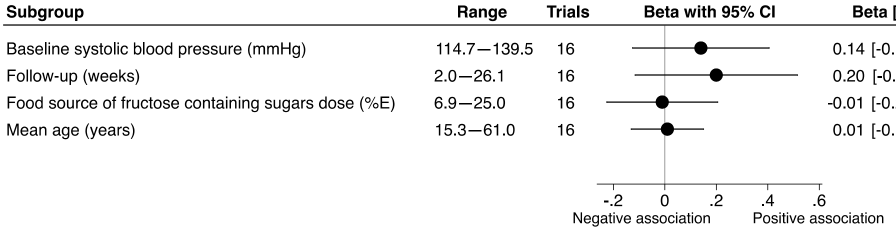

Data is presented as between group mean difference (95% confidence intervals) for a 1-unit change in the predictor variable. ß–coefficients were estimated using continuous meta-regression analysis. A positive ß-coefficient implies an increase in SBP with the food source of fructose-containing sugars intervention as the subgroup variable increases, and a negative ß-coefficient implies a decrease in SBP. Residual I2 reports inter-study heterogeneity not explained by the subgroup and was estimated using the Cochrane Q statistic. %E=percentage of total energy intake; CI, confidence interval.

S84 Fig. Continuous meta-regression analysis for the effect of fruit on SBP (mmHg) in substitution trials.

Continuous meta-regression
in substitution fruit trials

Subgroup	Range	Trials	Beta [95% CI]	P_{MD}	Residual I² (%)	P_Q
Baseline systolic blood pressure (mmHg)	104.2—166.7	11	-0.15 [-0.22 to -0.08]	<0.001	16	0.297
Follow-up (weeks)	1.0—24.0	11	-0.45 [-0.62 to -0.27]	<0.001	5	0.399
Food source of fructose containing sugars dose (%E)	1.7—20.0	11	0.28 [-0.13 to 0.69]	0.180	70	<0.001
Mean age (years)	23.5—59.0	11	-0.11 [-0.32 to 0.10]	0.324	72	<0.001

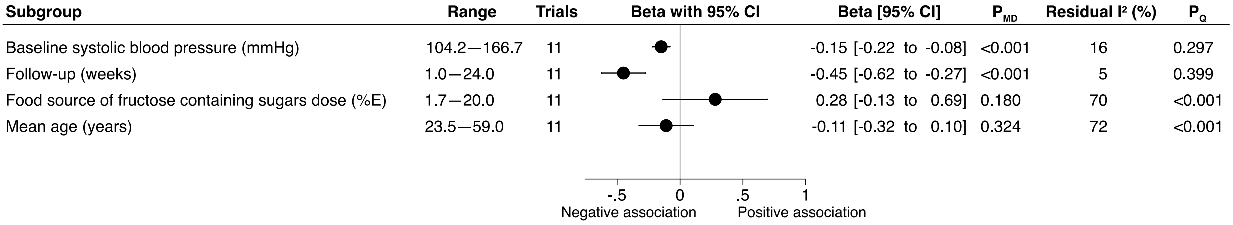

Data is presented as between group mean difference (95% confidence intervals) for a 1-unit change in the predictor variable. ß–coefficients were estimated using continuous meta-regression analysis. A positive ß-coefficient implies an increase in SBP with the food source of fructose-containing sugars intervention as the subgroup variable increases, and a negative ß-coefficient implies a decrease in SBP. Residual I2 reports inter-study heterogeneity not explained by the subgroup and was estimated using the Cochrane Q statistic. %E=percentage of total energy intake; CI, confidence interval.

S85 Fig. Continuous meta-regression analysis for the effect of mixed sources (with SSBs) on SBP (mmHg) in substitution trials.

Continuous meta-regression
in substitution mixed sources (with SSBs) trials

Subgroup	Range	Trials	Beta [95% CI]	P_{MD}	Residual I^2 (%)	P_Q
Baseline systolic blood pressure (mmHg)	108.8—135.5	12	-0.44 [-0.82 to -0.06]	0.024	30	0.164
Follow-up (weeks)	3.0—52.1	13	0.16 [-0.09 to 0.40]	0.199	44	0.048
Food source of fructose containing sugars dose (%E)	5.0—58.0	12	-0.22 [-0.38 to -0.06]	0.009	27	0.185
Mean age (years)	7.7—59.0	13	-0.10 [-0.26 to 0.06]	0.225	44	0.049

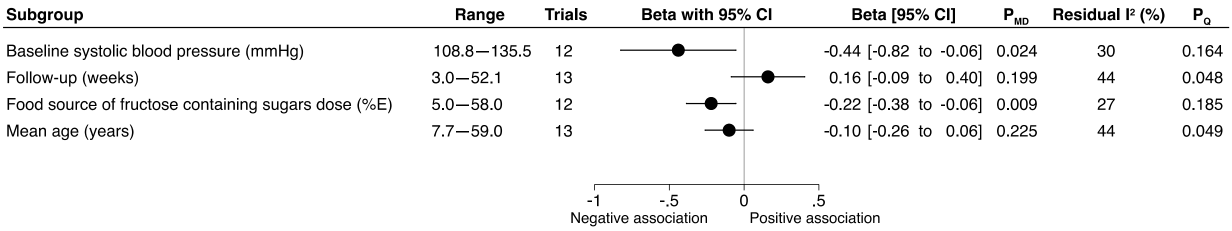

Data is presented as between group mean difference (95% confidence intervals) for a 1-unit change in the predictor variable. ß–coefficients were estimated using continuous meta-regression analysis. A positive ß-coefficient implies an increase in SBP with the food source of fructose-containing sugars intervention as the subgroup variable increases, and a negative ß-coefficient implies a decrease in SBP. Residual I2 reports inter-study heterogeneity not explained by the subgroup and was estimated using the Cochrane Q statistic.
%E=percentage of total energy intake; CI, confidence interval.
[a]N=1 trial missing data for baseline SBP.
[b]N=1 trial missing data for dose.
%E=percentage of total energy intake; CI, confidence interval.

S86 Fig. Continuous meta-regression analysis for the effect of SSBs on SBP (mmHg) in addition trials.

Continuous meta-regression
in addition SSB trials

Subgroup	Range	Trials	Beta with 95% CI	Beta [95% CI]	P_{MD}	Residual I^2 (%)	P_Q
Baseline systolic blood pressure[a] (mmHg)	113.3—130.3	16		0.10 [-0.18 to 0.38]	0.507	0	0.731
Follow-up (weeks)	2.0—26.1	17		0.16 [-0.07 to 0.39]	0.181	0	0.848
Food source of fructose containing sugars dose (%E)	6.9—26.0	17		-0.01 [-0.23 to 0.21]	0.893	0	0.730
Mean age (years)	22.7—53.0	17		-0.03 [-0.12 to 0.06]	0.473	0	0.766

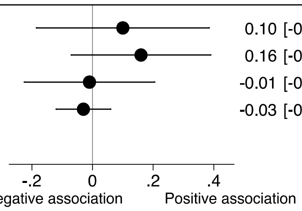

Data is presented as between group mean difference (95% confidence intervals) for a 1-unit change in the predictor variable. ß–coefficients were estimated using continuous meta-regression analysis. A positive ß-coefficient implies an increase in SBP with the food source of fructose-containing sugars intervention as the subgroup variable increases, and a negative ß-coefficient implies a decrease in SBP. Residual I2 reports inter-study heterogeneity not explained by the subgroup and was estimated using the Cochrane Q statistic.

[a]N=1 trial missing data for baseline SBP.

%E=percentage of total energy intake; CI, confidence interval.

S87 Fig. Continuous meta-regression analysis for the effect of 100% fruit juice on SBP (mmHg) in addition trials.

Continuous meta-regression

in addition 100% fruit juice trials

Subgroup	Range	Trials	Beta [95% CI]	P_{MD}	Residual I^2 (%)	P_Q
Baseline systolic blood pressure (mmHg)	116.2—140.4	16	-0.20 [-0.46 to 0.06]	0.126	52	0.011
Follow-up (weeks)	2.0—52.0	16	-0.27 [-0.54 to 0.00]	0.047	51	0.012
Food source of fructose containing sugars dose (%E)	1.7—12.6	16	0.36 [-0.05 to 0.77]	0.087	52	0.010
Mean age[a] (years)	25.2—66.5	14	-0.07 [-0.26 to 0.12]	0.471	60	0.003

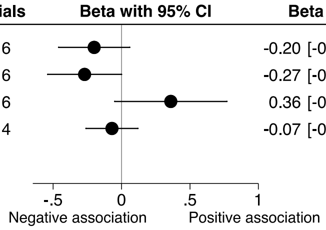

Data is presented as between group mean difference (95% confidence intervals) for a 1-unit change in the predictor variable. ß–coefficients were estimated using continuous meta-regression analysis. A positive ß-coefficient implies an increase in SBP with the food source of fructose-containing sugars intervention as the subgroup variable increases, and a negative ß-coefficient implies a decrease in SBP. Residual I2 reports inter-study heterogeneity not explained by the subgroup and was estimated using the Cochrane Q statistic.

[a]N=2 trials missing data for mean age.

%E=percentage of total energy intake; CI, confidence interval.

S88 Fig. Continuous meta-regression analysis for the effect of fruit on SBP (mmHg) in addition trials.

Continuous meta-regression
in addition fruit trials

Subgroup	Range	Trials	Beta [95% CI]	P_{MD}	Residual I^2 (%)	P_Q
Baseline systolic blood pressure[a] (mmHg)	111.5—153.0	14	-0.11 [-0.38 to 0.16]	0.424	73	<0.001
Follow-up (weeks)	4.0—12.0	16	0.16 [-1.00 to 1.32]	0.782	69	<0.001
Food source of fructose containing sugars dose (%E)	2.2—10.6	16	0.96 [0.23 to 1.69]	0.009	45	0.029
Mean age (years)	21.5—56.5	16	-0.14 [-0.40 to 0.12]	0.273	55	0.005

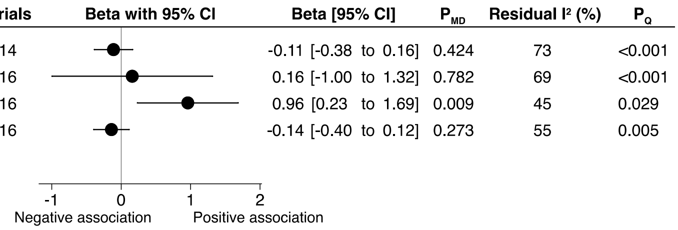

Data is presented as between group mean difference (95% confidence intervals) for a 1-unit change in the predictor variable. ß–coefficients were estimated using continuous meta-regression analysis. A positive ß-coefficient implies an increase in SBP with the food source of fructose-containing sugars intervention as the subgroup variable increases, and a negative ß-coefficient implies a decrease in SBP. Residual I2 reports inter-study heterogeneity not explained by the subgroup and was estimated using the Cochrane Q statistic.
[a]N=2 trials missing data for baseline SBP.
%E=percentage of total energy intake; CI, confidence interval.

S89 Fig. Continuous meta-regression analysis for the effect of SSBs on DBP (mmHg) in addition trials.

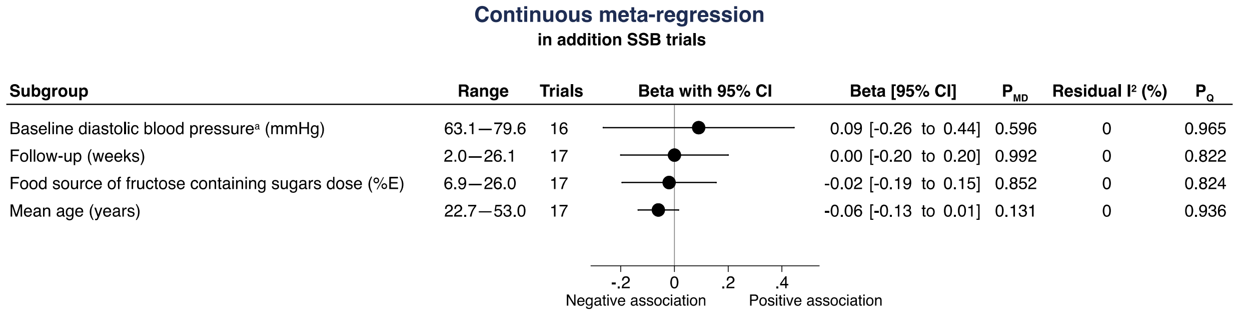

Data is presented as between group mean difference (95% confidence intervals) for a 1-unit change in the predictor variable. ß–coefficients were estimated using continuous meta-regression analysis. A positive ß-coefficient implies an increase in DBP with the food source of fructose-containing sugars intervention as the subgroup variable increases, and a negative ß-coefficient implies a decrease in DBP. Residual I2 reports inter-study heterogeneity not explained by the subgroup and was estimated using the Cochrane Q statistic.

[a]N=1 trial missing data for baseline DBP.

%E=percentage of total energy intake; CI, confidence interval.

S90 Fig. Continuous meta-regression analysis for the effect of 100% fruit juice on DBP (mmHg) in addition trials.

Continuous meta-regression
in addition 100% fruit juice trials

Subgroup	Range	Trials	Beta with 95% CI	Beta [95% CI]	P_{MD}	Residual I^2 (%)	P_Q
Baseline diastolic blood pressure (mmHg)	68.0—96.5	16		-0.14 [-0.32 to 0.04]	0.141	40	0.052
Follow-up (weeks)	2.0—52.0	16		-0.08 [-0.27 to 0.12]	0.399	49	0.016
Food source of fructose containing sugars dose (%E)	1.0—12.6	16		0.21 [-0.15 to 0.57]	0.259	49	0.018
Mean age[a] (years)	25.2—66.5	14		0.06 [-0.10 to 0.22]	0.457	52	0.015

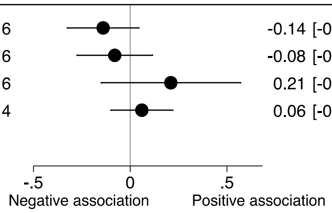

Data is presented as between group mean difference (95% confidence intervals) for a 1-unit change in the predictor variable. ß–coefficients were estimated using continuous meta-regression analysis. A positive ß-coefficient implies an increase in DBP with the food source of fructose-containing sugars intervention as the subgroup variable increases, and a negative ß-coefficient implies a decrease in DBP. Residual I2 reports inter-study heterogeneity not explained by the subgroup and was estimated using the Cochrane Q statistic.
[a]N=2 trials missing data for mean age.
%E=percentage of total energy intake; CI, confidence interval.

S91 Fig. Continuous meta-regression analysis for the effect of fruit on DBP (mmHg) in addition trials.

Continuous meta-regression
in addition fruit trials

Subgroup	Range	Trials	Beta [95% CI]	P_{MD}	Residual I^2 (%)	P_Q
Baseline diastolic blood pressure[a] (mmHg)	70.4—98.4	14	-0.12 [-0.43 to 0.19]	0.451	60	0.002
Follow-up (weeks)	4.0—12.0	16	-0.13 [-0.93 to 0.67]	0.754	55	0.005
Food source of fructose containing sugars dose (%E)	2.2—10.6	16	0.68 [0.15 to 1.21]	0.012	32	0.113
Mean age (years)	21.5—56.5	16	-0.04 [-0.21 to 0.13]	0.696	51	0.013

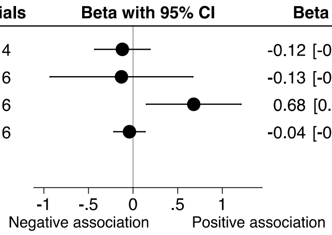

Data is presented as between group mean difference (95% confidence intervals) for a 1-unit change in the predictor variable. ß–coefficients were estimated using continuous meta-regression analysis. A positive ß-coefficient implies an increase in DBP with the food source of fructose-containing sugars intervention as the subgroup variable increases, and a negative ß-coefficient implies a decrease in DBP. Residual I2 reports inter-study heterogeneity not explained by the subgroup and was estimated using the Cochrane Q statistic.
[a]N=2 trials missing data for baseline DBP.
%E=percentage of total energy intake; CI, confidence interval.

S92 Fig. Linear meta-regression analyses for the effect of food sources of fructose-containing sugars dose on SBP (mmHg) in substitution trials.

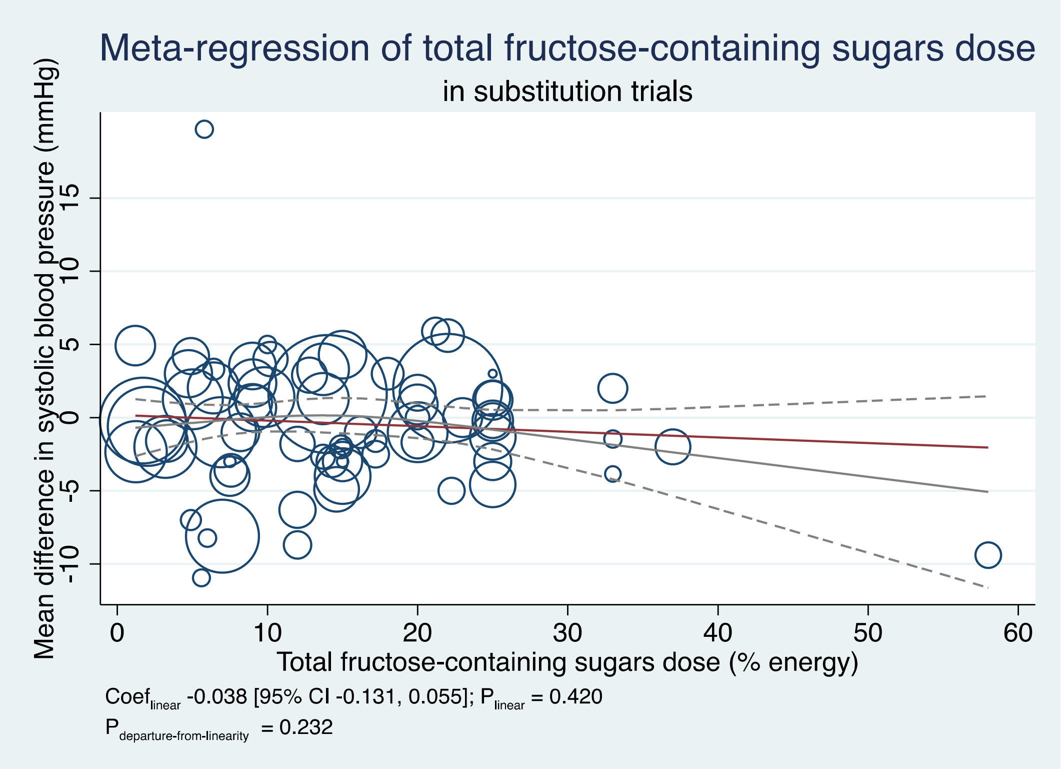

Individual trials are represented by the circles, with their weight in the overall analysis represented by the size of the circles. The straight line represents the estimate dose response for the amount of fructose-containing sugars consumed (% of total energy intake) and the dashed lines represent the upper and lower 95% Confidence Intervals.

S93 Fig. Linear meta-regression analyses for the effect of food sources of fructose-containing sugars dose on SBP (mmHg) in addition trials.

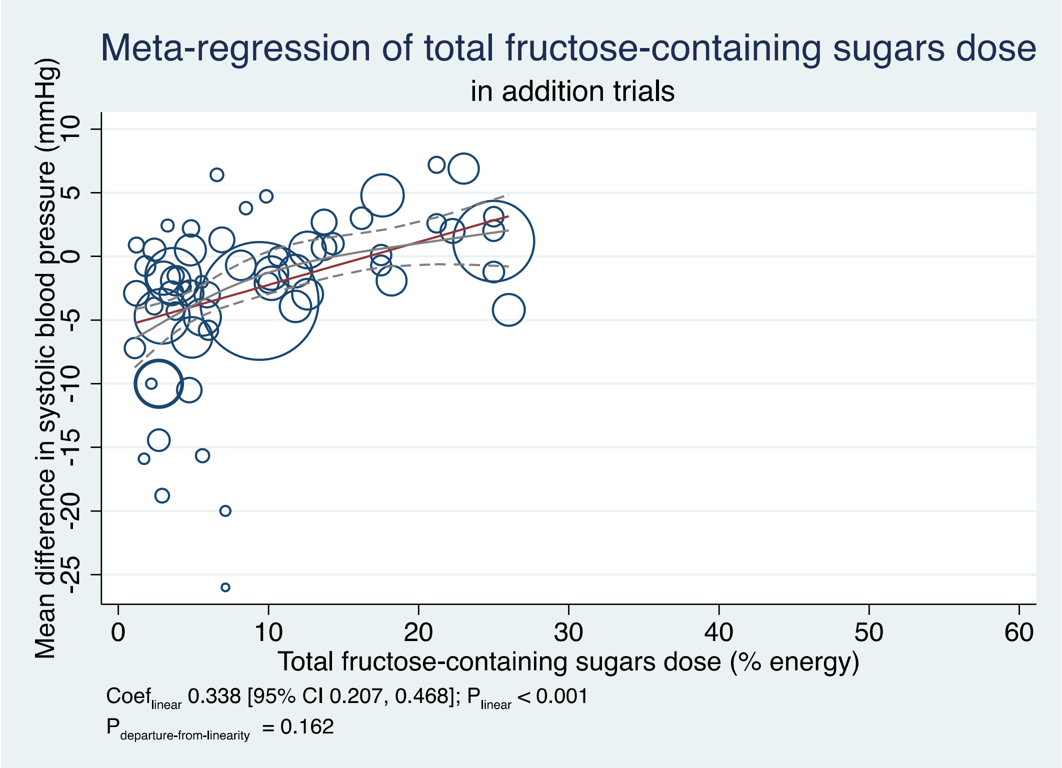

Individual trials are represented by the circles, with their weight in the overall analysis represented by the size of the circles. The straight line represents the estimate dose response for the amount of fructose-containing sugars consumed (% of total energy intake) and the dashed lines represent the upper and lower 95% Confidence Intervals.

S94 Fig. Linear meta-regression analyses for the effect of food sources of fructose-containing sugars dose on SBP (mmHg) in subtraction trials.

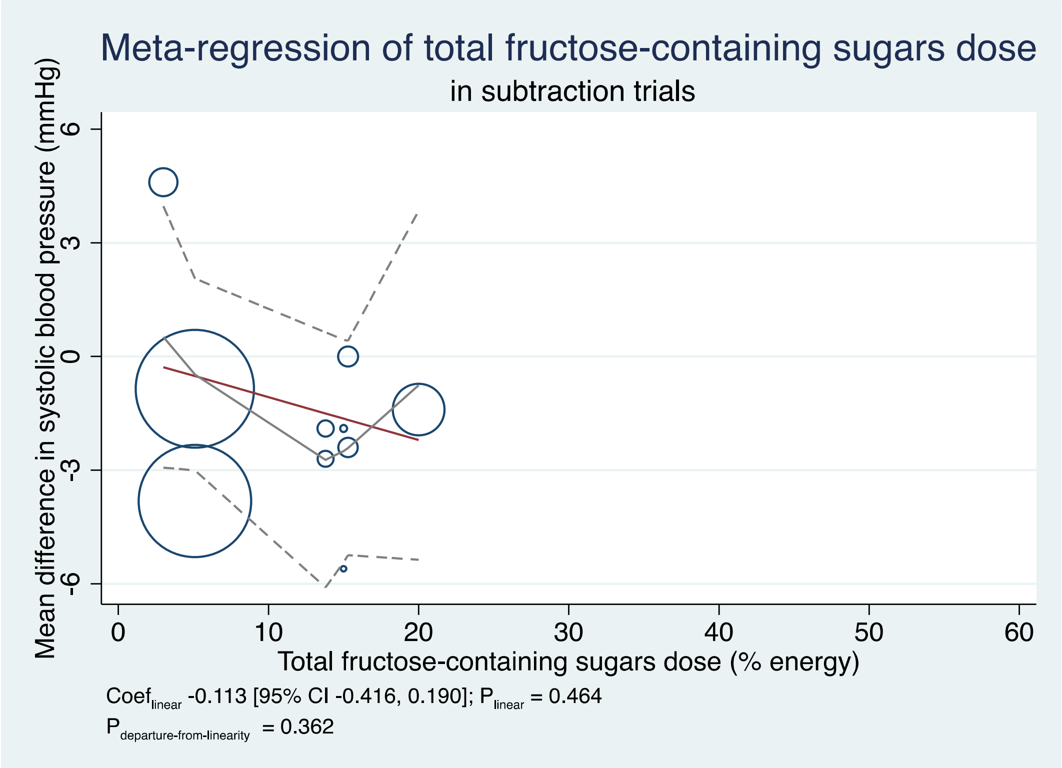

Individual trials are represented by the circles, with their weight in the overall analysis represented by the size of the circles. The straight line represents the estimate dose response for the amount of fructose-containing sugars consumed (% of total energy intake) and the dashed lines represent the upper and lower 95% Confidence Intervals.

S95 Fig. Linear meta-regression analyses for the effect of food sources of fructose-containing sugars dose on SBP (mmHg) in *ad libitum* trials.

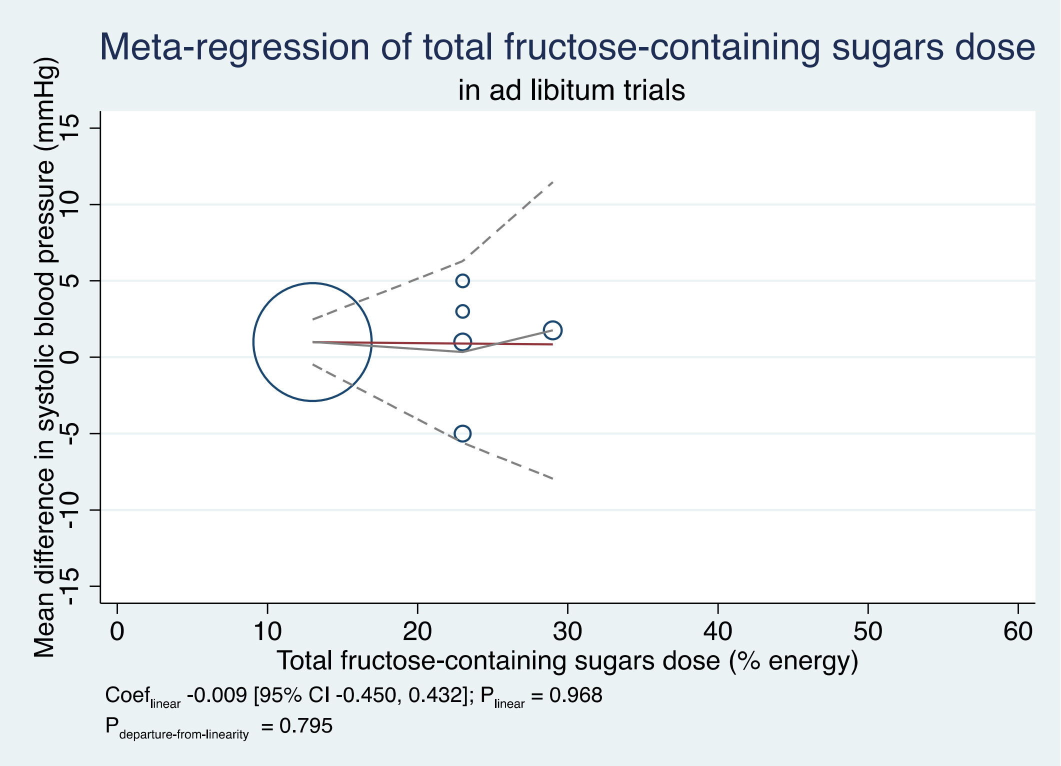

Individual trials are represented by the circles, with their weight in the overall analysis represented by the size of the circles. The straight line represents the estimate dose response for the amount of fructose-containing sugars consumed (% of total energy intake) and the dashed lines represent the upper and lower 95% Confidence Intervals.

S96 Fig. Linear meta-regression analyses for the effect of food sources of fructose-containing sugars dose on DBP (mmHg) in substitution trials.

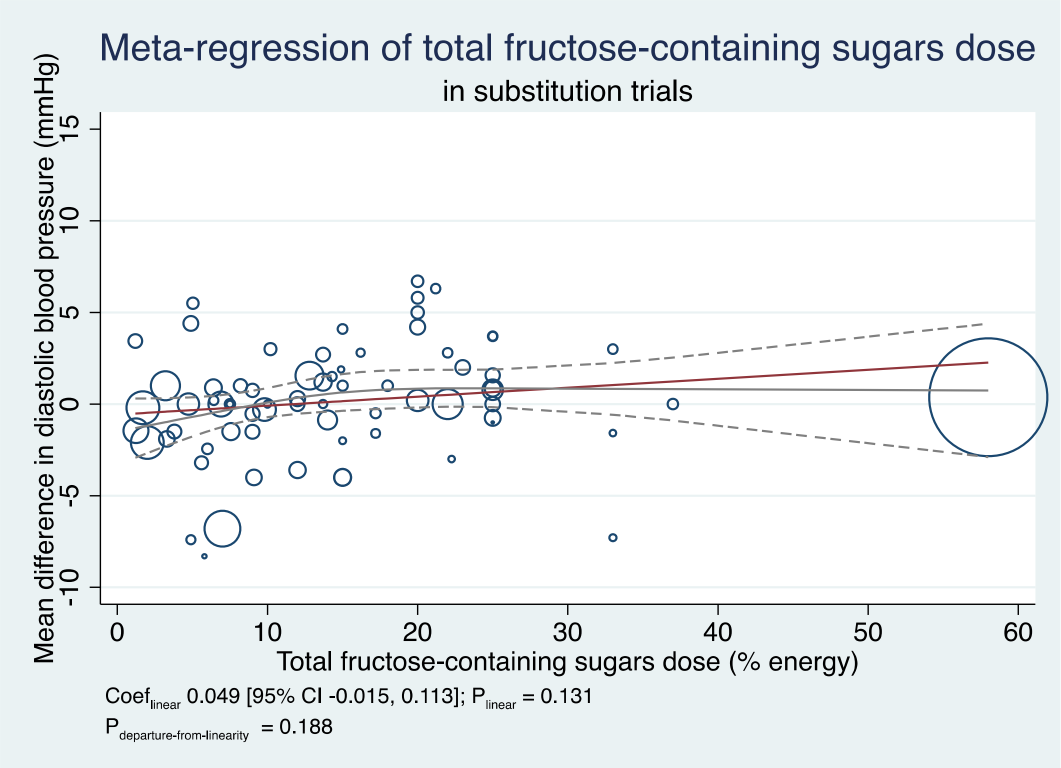

Individual trials are represented by the circles, with their weight in the overall analysis represented by the size of the circles. The straight line represents the estimate dose response for the amount of fructose-containing sugars consumed (% of total energy intake) and the dashed lines represent the upper and lower 95% Confidence Intervals.

S97 Fig. Linear meta-regression analyses for the effect of food sources of fructose-containing sugars dose on DBP (mmHg) in addition trials.

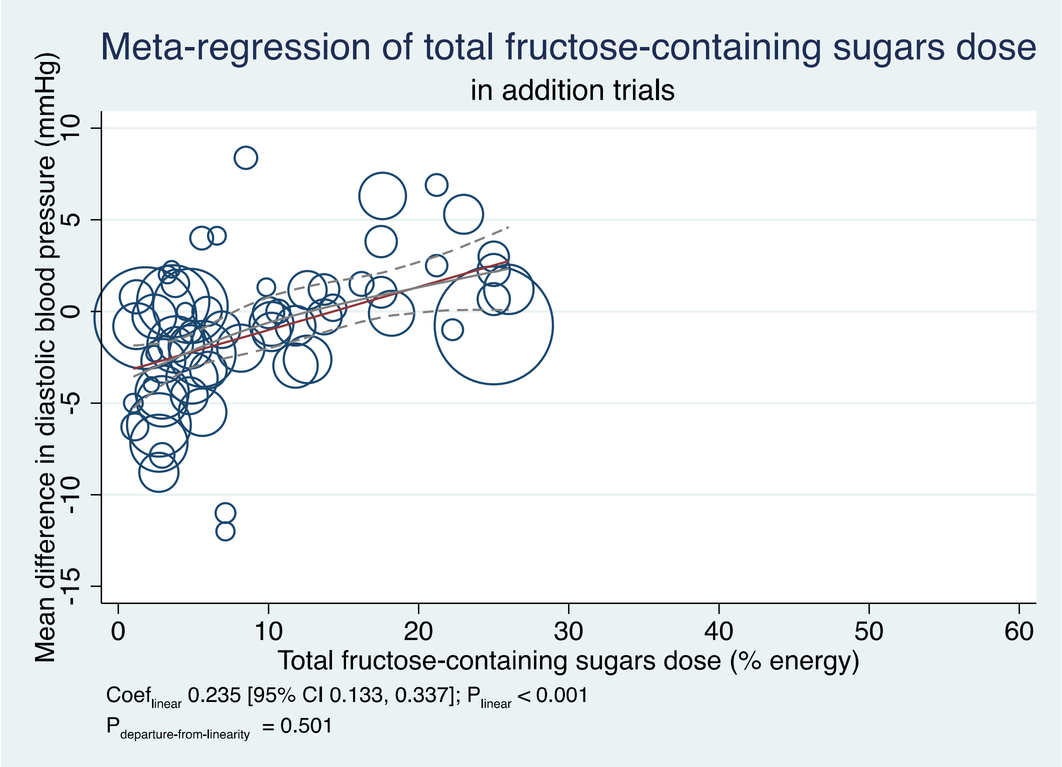

Individual trials are represented by the circles, with their weight in the overall analysis represented by the size of the circles. The straight line represents the estimate dose response for the amount of fructose-containing sugars consumed (% of total energy intake) and the dashed lines represent the upper and lower 95% Confidence Intervals.

S98 Fig. Linear meta-regression analyses for the effect of food sources of fructose-containing sugars dose on DBP (mmHg) in subtraction trials.

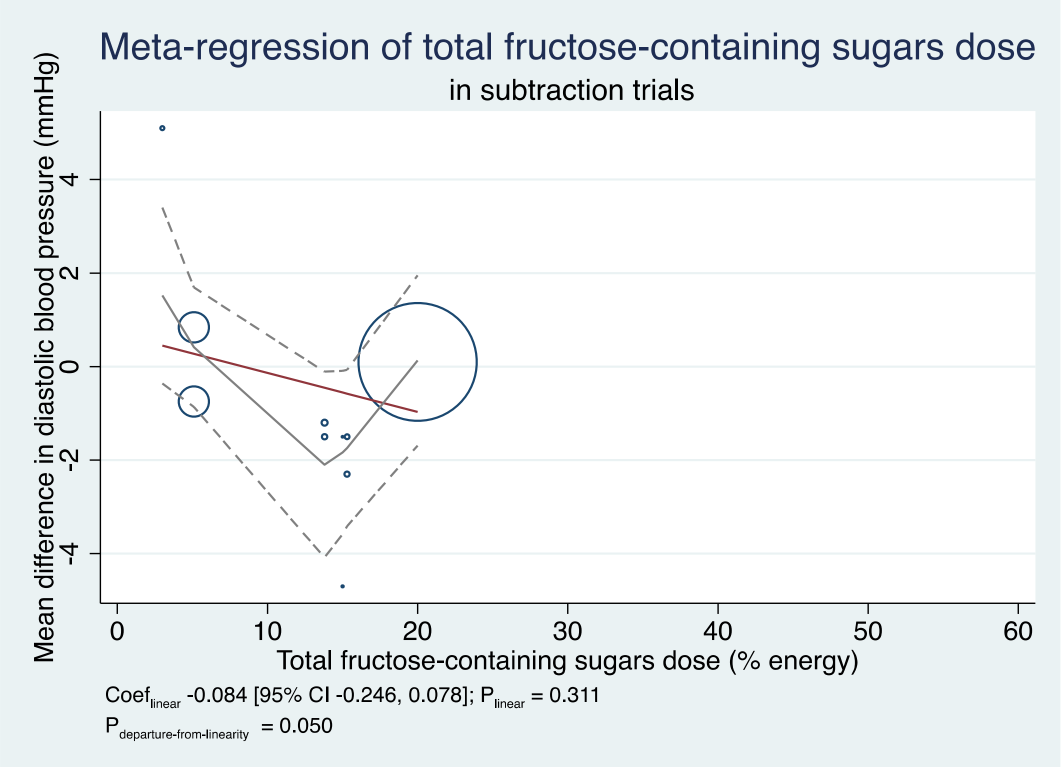

Individual trials are represented by the circles, with their weight in the overall analysis represented by the size of the circles. The straight line represents the estimate dose response for the amount of fructose-containing sugars consumed (% of total energy intake) and the dashed lines represent the upper and lower 95% Confidence Intervals.

S99 Fig. Linear meta-regression analyses for the effect of food sources of fructose-containing sugars dose on DBP (mmHg) in *ad libitum* trials.

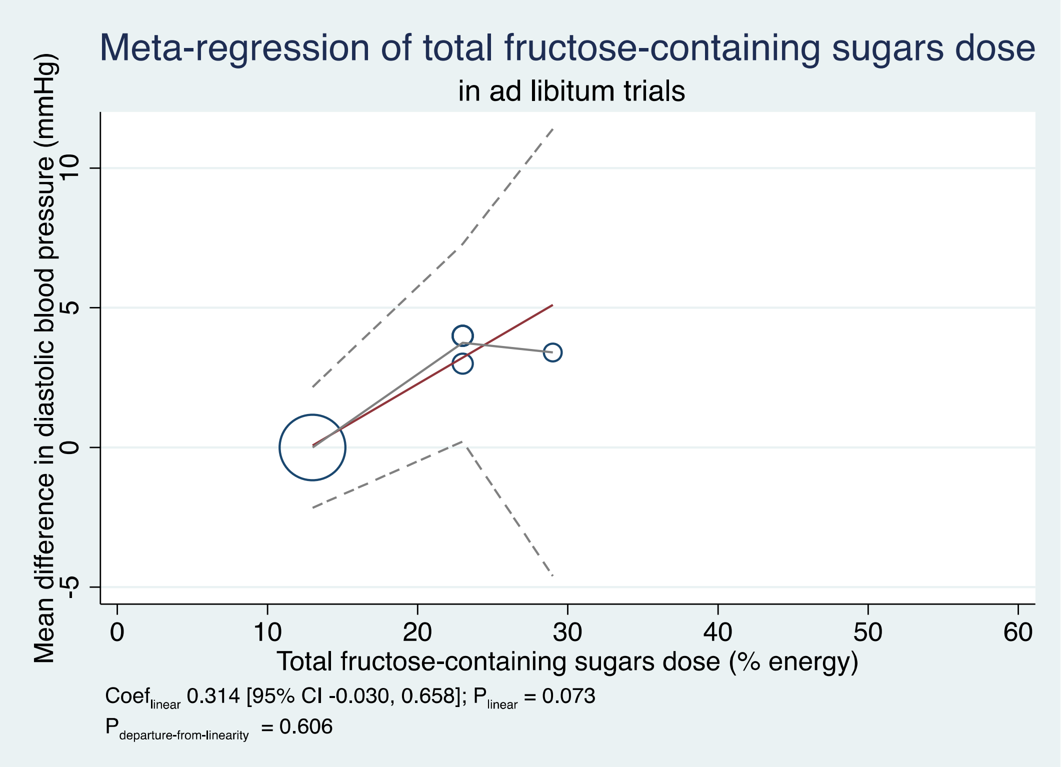

Individual trials are represented by the circles, with their weight in the overall analysis represented by the size of the circles. The straight line represents the estimate dose response for the amount of fructose-containing sugars consumed (% of total energy intake) and the dashed lines represent the upper and lower 95% Confidence Intervals.

S100 Fig. Linear meta-regression analyses for the effect of individual food sources of fructose-containing sugars dose on SBP (mmHg) in substitution trials.

A

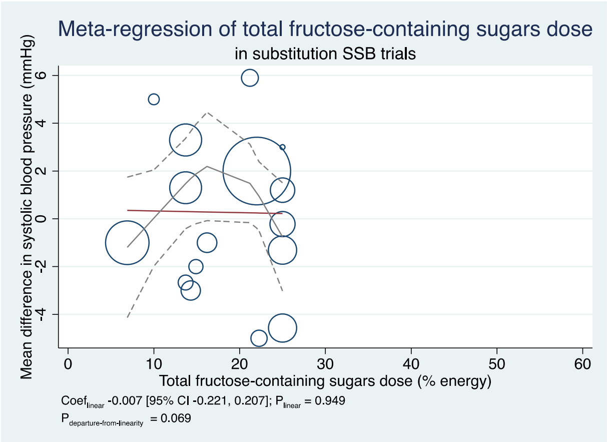

B

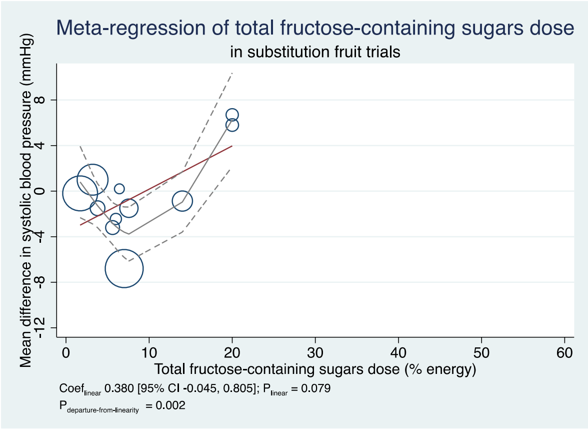

C

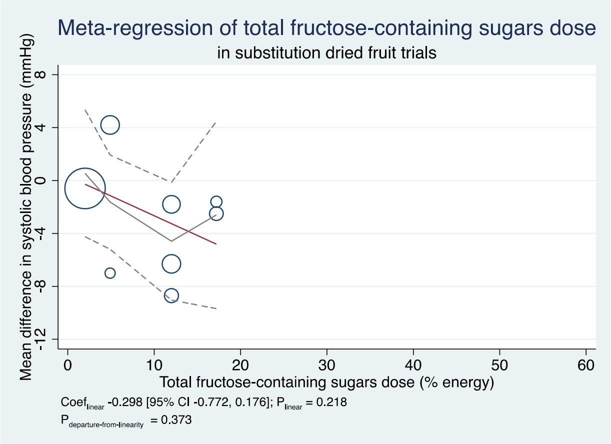

D

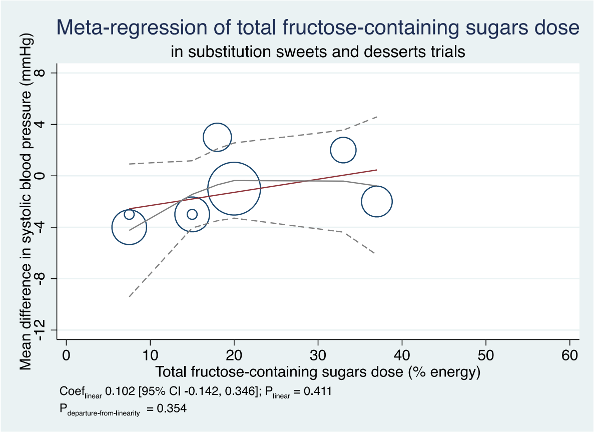

E

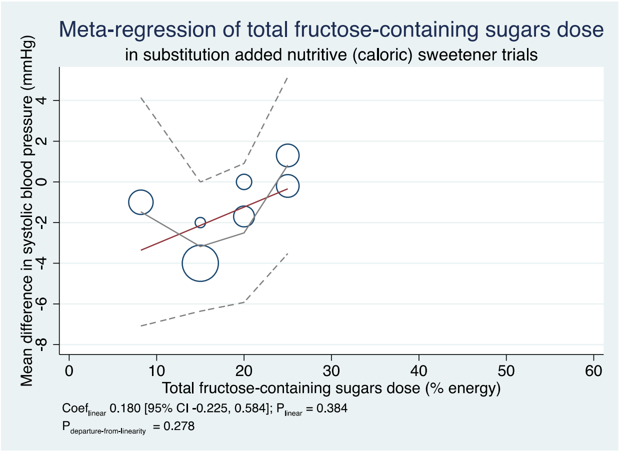

F

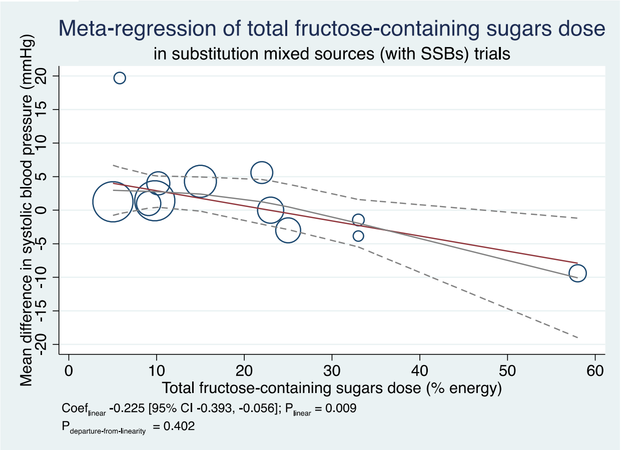

Individual trials are represented by the circles, with their weight in the overall analysis represented by the size of the circles. The straight line represents the estimate dose response for amount of fructose-containing sugars consumed (% of total energy intake) and the dashed lines represent the upper and lower 95% Confidence Intervals.
Panel A: SSB, B: fruit; C: dried fruit; D: sweets and desserts; E: added nutritive (caloric) sweetener; F: mixed sources (with SSBs) with outlier removed.
CI=confidence interval; coef=coefficient; SSB=sugar-sweetened beverage.

S101 Fig. Linear meta-regression analyses for the effect of individual food sources of fructose-containing sugars dose on SBP (mmHg) in addition trials.

A

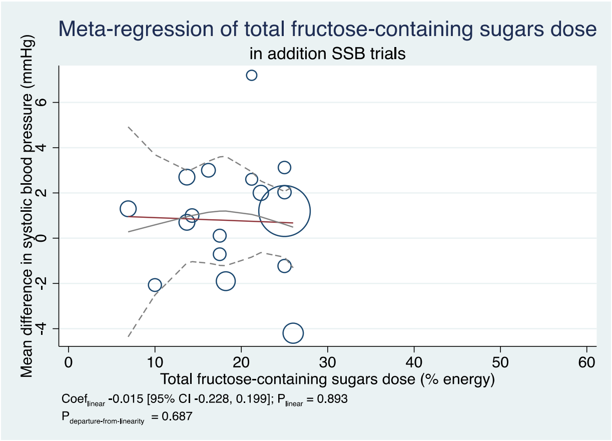

B

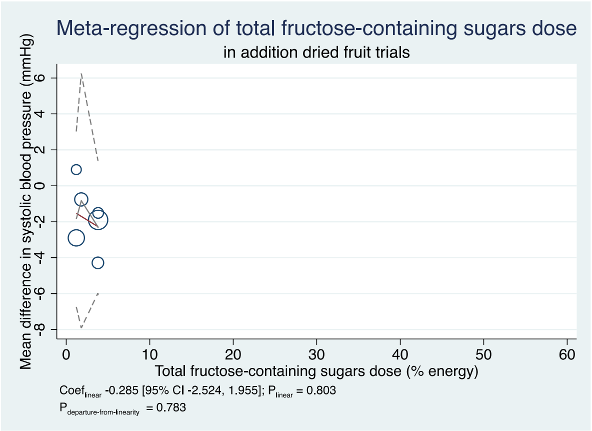

C

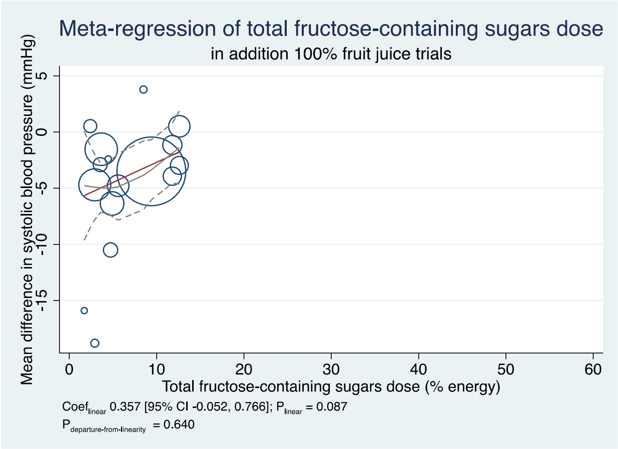

D

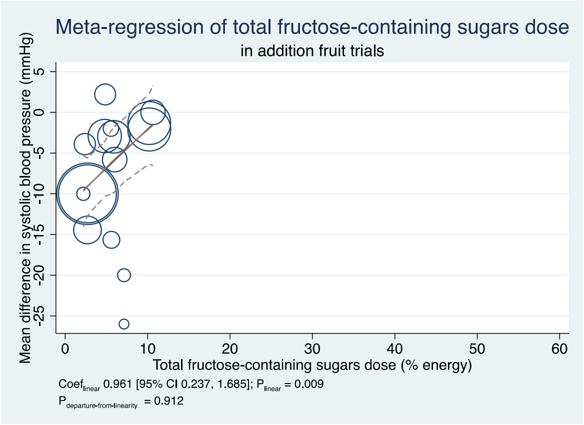

Individual trials are represented by the circles, with their weight in the overall analysis represented by the size of the circles. The straight line represents the estimate dose response for amount of fructose-containing sugars consumed (% of total energy intake) and the dashed lines represent the upper and lower 95% Confidence Intervals.

Panel A: SSB; B: dried fruit; C: 100% fruit juice; D: fruit -To convert this significant coefficient to represent per serving of fruit, multiply by 5 since 1 serving of fruit (apple (1 medium) or strawberries (2cups) is ~4%E, 1 banana is ~6%E) is about 5%E of a 2000kcal diet. Thus, the equation can be expressed as 0.961mmHg (95% CI: 0.237 to 1.685) * 5 = 4.8mmHg (95% CI: 1.2, 8.5) per serving (5%E) of fruit.

CI=confidence interval; coef=coefficient; SSB=sugar-sweetened beverage.

S102 Fig. Linear meta-regression analyses for the effect of SSB dose on SBP (mmHg) in subtraction trials.

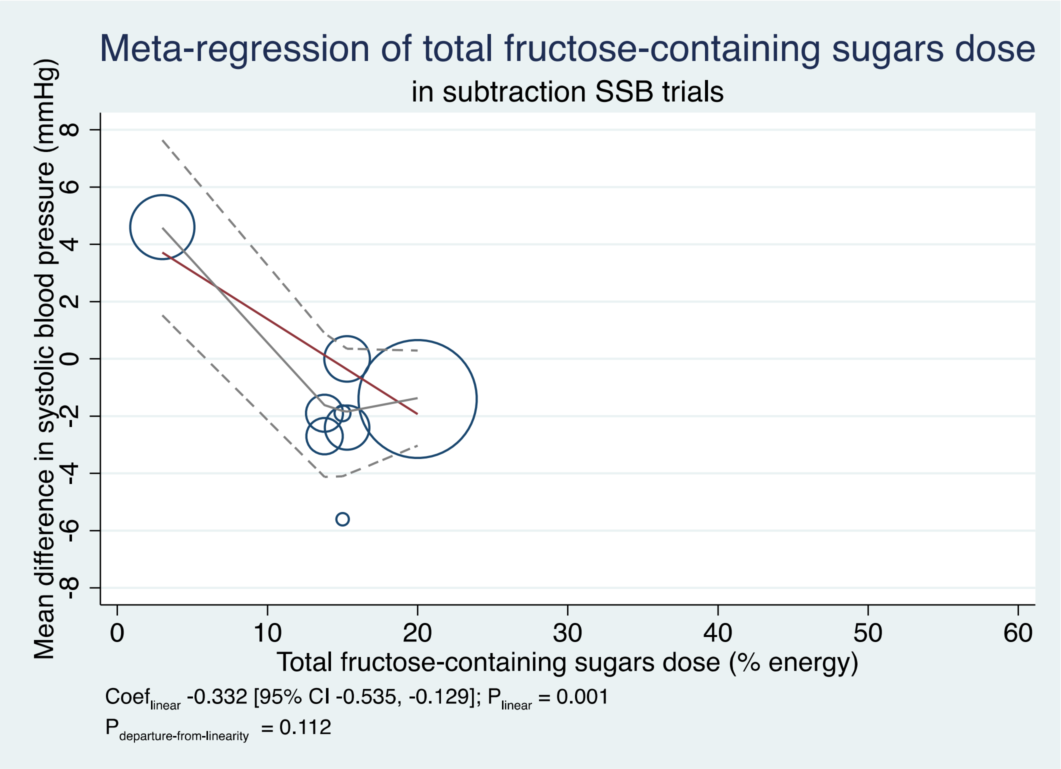

Individual trials are represented by the circles, with their weight in the overall analysis represented by the size of the circles. The straight line represents the estimate dose response for amount of fructose-containing sugars consumed (% of total energy intake) and the dashed lines represent the upper and lower 95% Confidence Intervals.

To convert coefficient to represent per serving of SSB, multiply by 8 since 1 can (355mL) of cola is ~40g sugar or about 8%E of a 2000kcal diet. Thus, the equation can be expressed as -0.332mmHg (95% CI: -0.535 to -0.129) * 8 = -2.66mmHg (95% CI: -4.28, -1.03) per serving (355ml, 8%E) of SSB.

CI=confidence interval; coef=coefficient; SSB=sugar-sweetened beverage.

S103 Fig. Linear meta-regression analyses for the effect of individual food sources of fructose-containing sugars dose on DBP (mmHg) in addition trials.

A

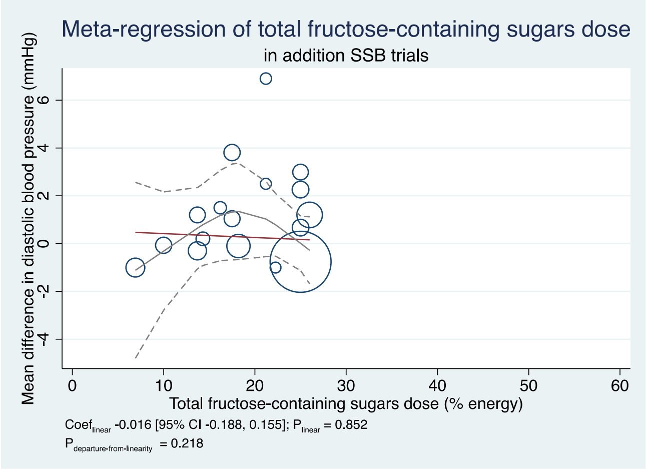

B

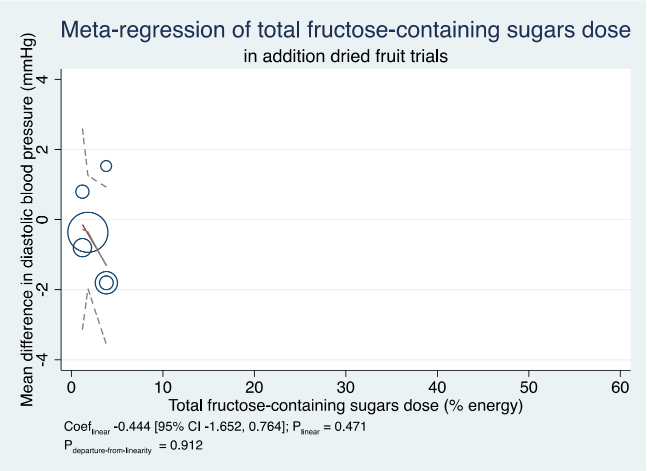

C

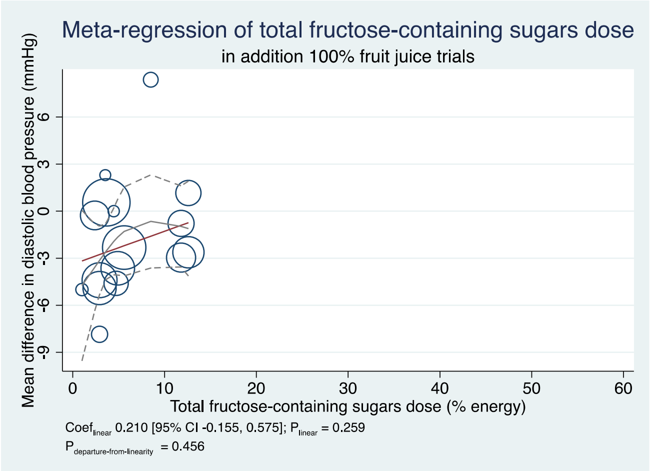

D

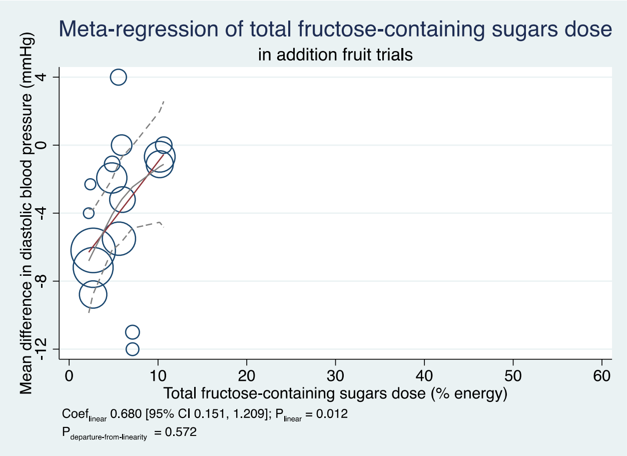

Individual trials are represented by the circles, with their weight in the overall analysis represented by the size of the circles. The straight line represents the estimate dose response for amount of fructose-containing sugars consumed (% of total energy intake) and the dashed lines represent the upper and lower 95% Confidence Intervals.
Panel A: SSB; B: dried fruit; C: 100% fruit juice; D: fruit -To convert this significant coefficient to represent per serving of fruit, multiply by 5 since 1 serving of fruit (apple (1 medium) or strawberries (2cups) is ~4%E, 1 banana is ~6%E) is about 5%E of a 2000kcal diet. Thus, the equation can be expressed as 0.680mmHg (95% CI: 0.151 to 1.209) * 5 = 3.4mmHg (95% CI: 0.8, 6.1) per serving (5%E) of fruit.
CI=confidence interval; coef=coefficient; SSB=sugar-sweetened beverage.

S104 Fig. Linear meta-regression analyses for the effect of individual food sources of fructose-containing sugars dose on DBP (mmHg) in subtraction trials.

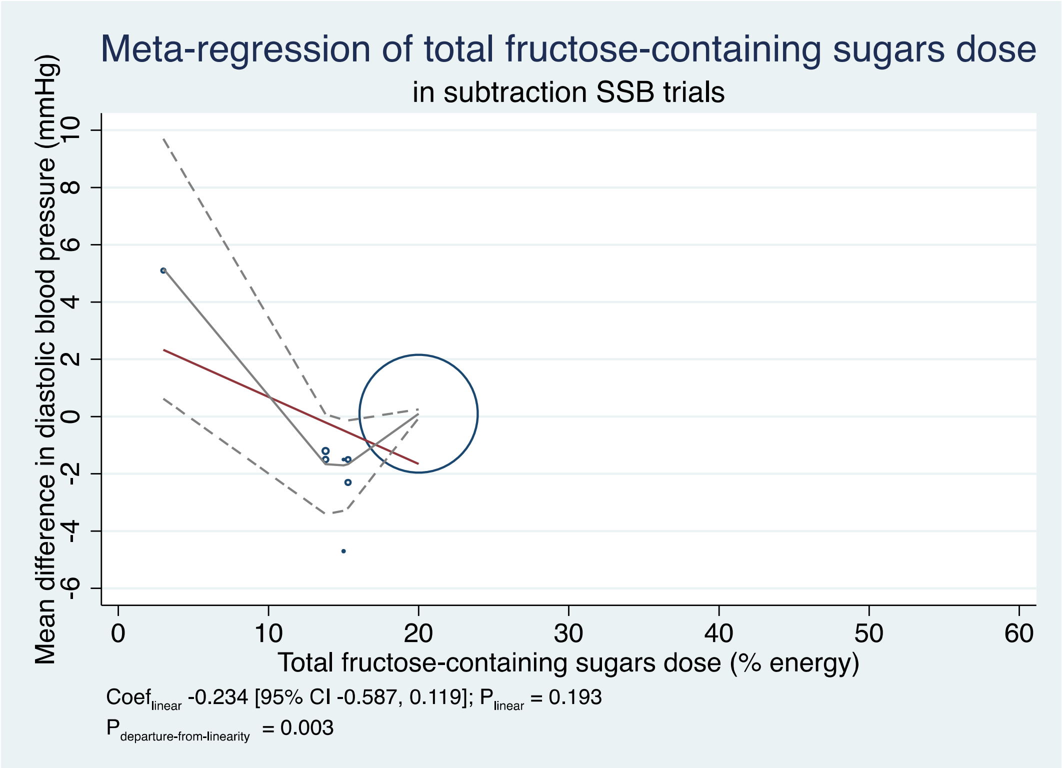

Individual trials are represented by the circles, with their weight in the overall analysis represented by the size of the circles. The straight line represents the estimate dose response for amount of fructose-containing sugars consumed (% of total energy intake) and the dashed lines represent the upper and lower 95% Confidence Intervals.

CI=confidence interval; coef=coefficient; SSB=sugar-sweetened beverage.

S105 Fig. Non-linear dose-response analysis using public thresholds of 5, 10, and 25% of energy for the effect of food sources of fructose-containing sugars dose (% of total energy) on SBP (mmHg) in substitution trials.

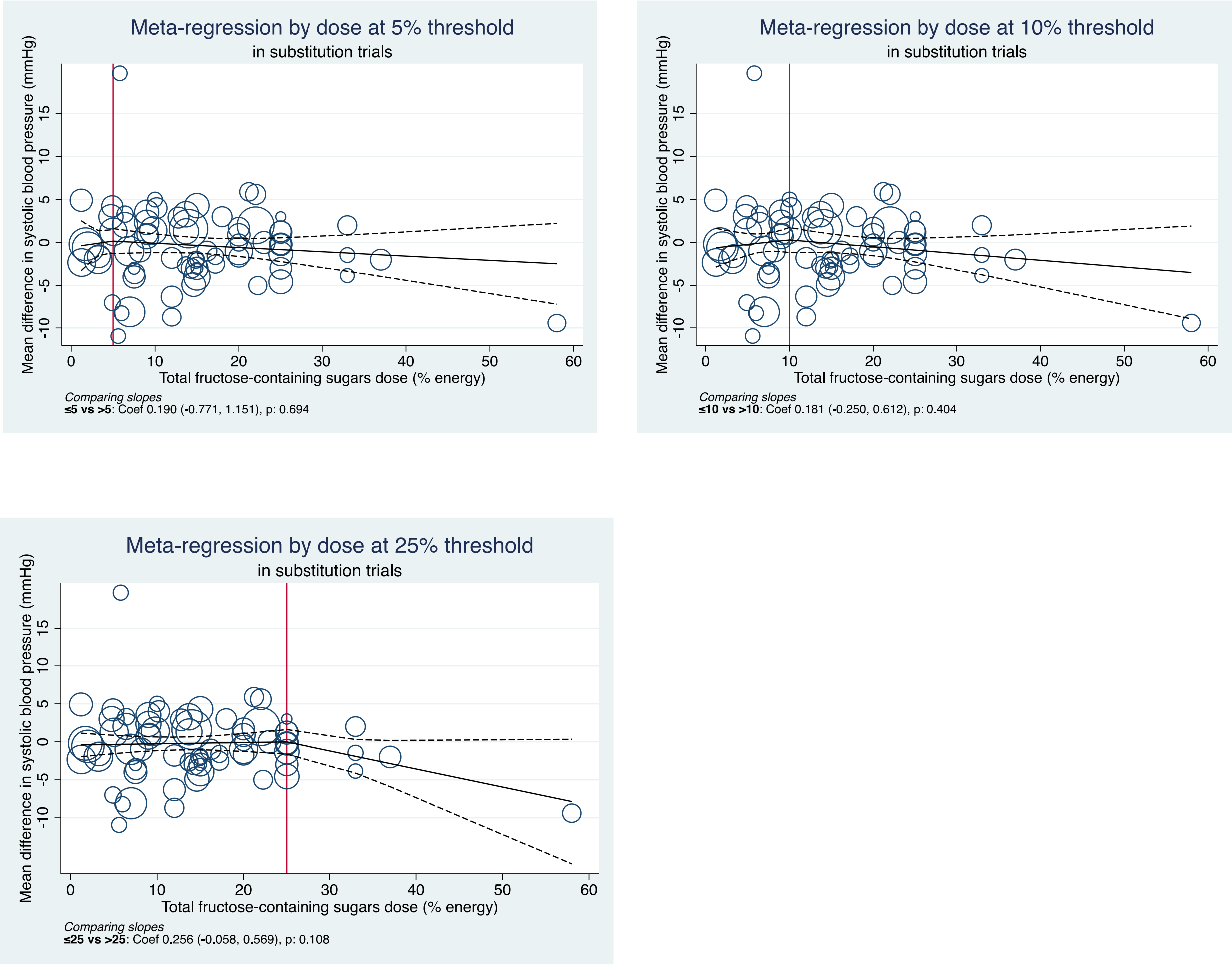

Individual trials are represented by the circles, with their weight in the overall analysis represented by the size of the circles. The horizontal straight line represents the estimate dose response for amount of fructose-containing sugars consumed (% of total energy intake), and the dashed lines represent the upper and lower 95% confidence intervals. The vertical straight lines represent the threshold knots.
coef=coefficient.

S106 Fig. Non-linear dose-response analysis using public thresholds of 5, 10, and 25% of energy for the effect of food sources of fructose-containing sugars dose (% of total energy) on SBP (mmHg) in addition trials.

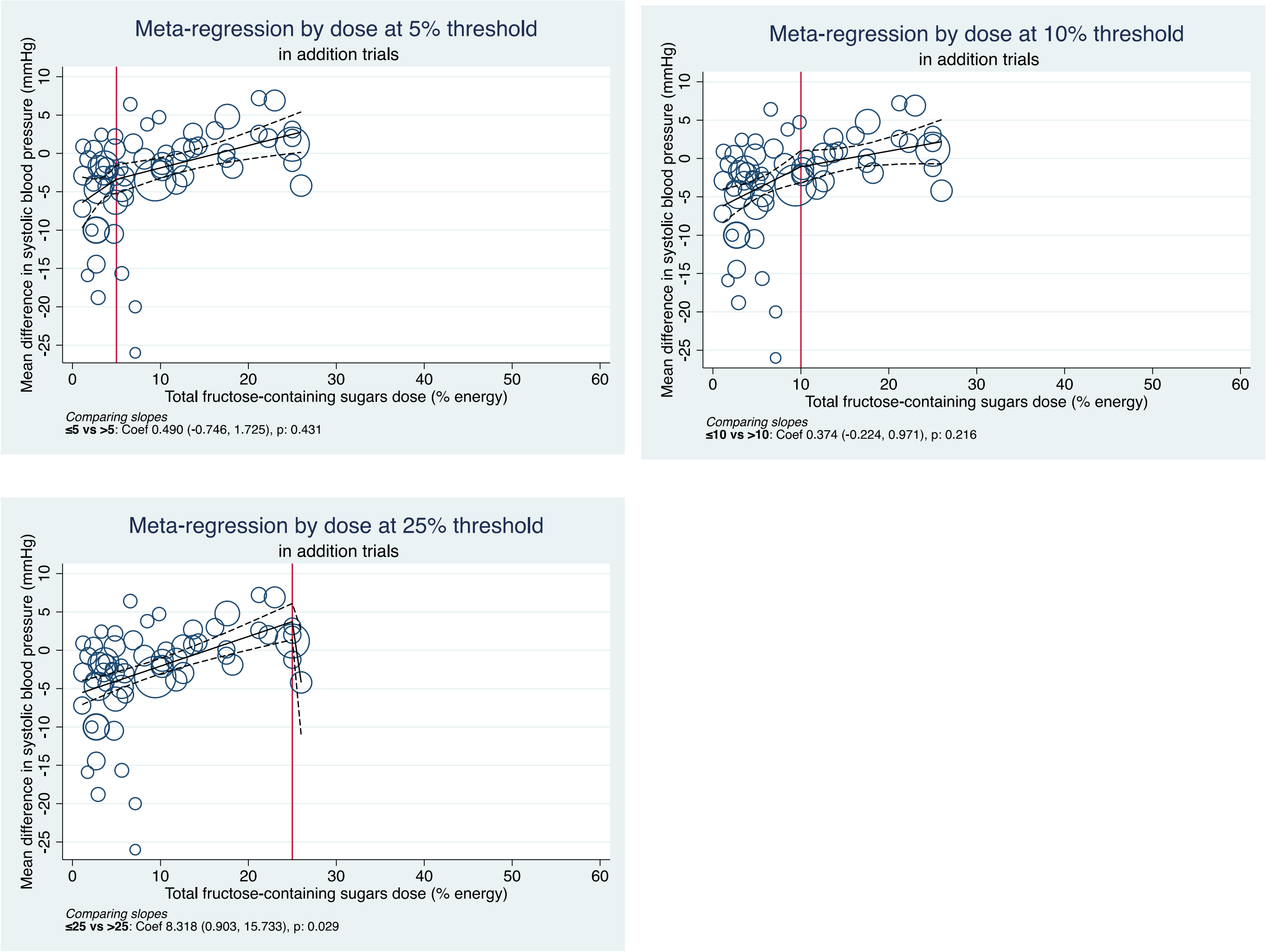

Individual trials are represented by the circles, with their weight in the overall analysis represented by the size of the circles. The horizontal straight line represents the estimate dose response for amount of fructose-containing sugars consumed (% of total energy intake), and the dashed lines represent the upper and lower 95% confidence intervals. The vertical straight lines represent the threshold knots.
coef=coefficient.

S107 Fig. Non-linear dose-response analysis using public thresholds of 5 and 10% of energy for the effect of food sources of fructose-containing sugars dose (% of total energy) on SBP (mmHg) in subtraction trials.

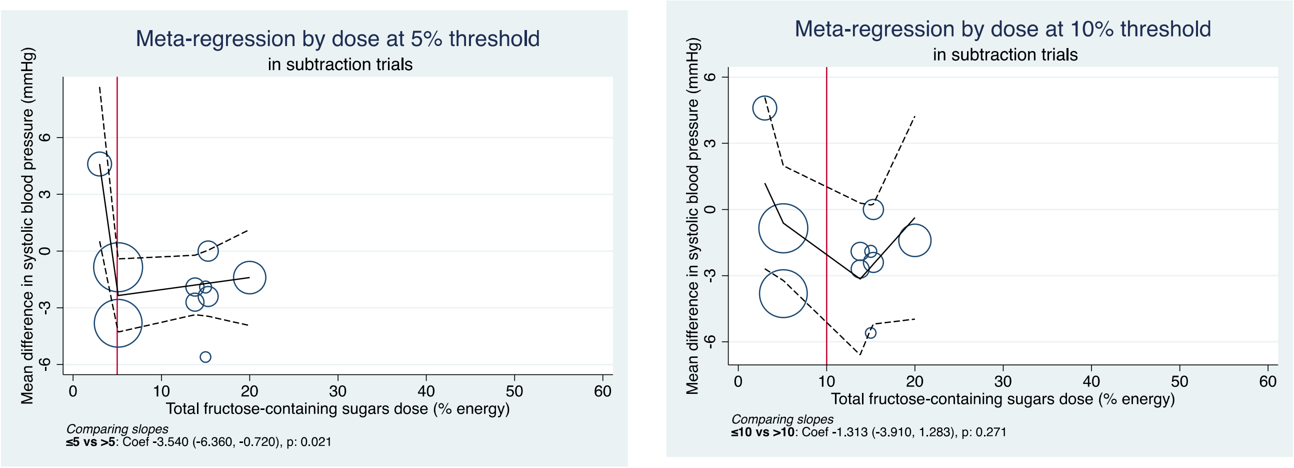

Individual trials are represented by the circles, with their weight in the overall analysis represented by the size of the circles. The horizontal straight line represents the estimate dose response for amount of fructose-containing sugars consumed (% of total energy intake), and the dashed lines represent the upper and lower 95% confidence intervals. The vertical straight lines represent the threshold knots.

Non-linear dose-response analysis using public thresholds of 25% was not conducted as there were no trials included which had a dose greater than 25% of total energy.

coef=coefficient.

S108 Fig. Non-linear dose-response analysis using public thresholds of 25% of energy for the effect of food sources of fructose-containing sugars dose (% of total energy) on SBP (mmHg) in *ad libitum* trials.

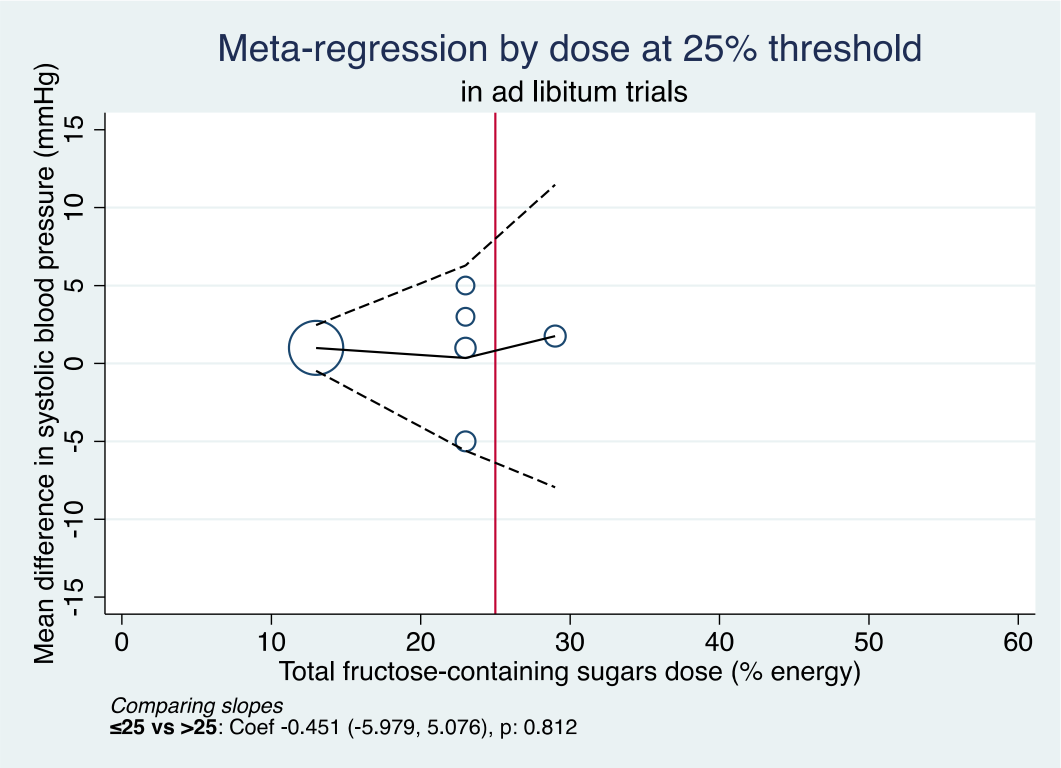

Individual trials are represented by the circles, with their weight in the overall analysis represented by the size of the circles. The horizontal straight line represents the estimate dose response for amount of fructose-containing sugars consumed (% of total energy intake), and the dashed lines represent the upper and lower 95% confidence intervals. The vertical straight lines represent the threshold knots.
Non-linear dose-response analysis using public thresholds of 5% and 10% of energy was not conducted as there were no trials included which had a dose less than 10% of total energy.
coef=coefficient.

S109 Fig. Non-linear dose-response analysis using public thresholds of 5, 10, and 25% of energy for the effect of food sources of fructose-containing sugars dose (% of total energy) on DBP (mmHg) in substitution trials.

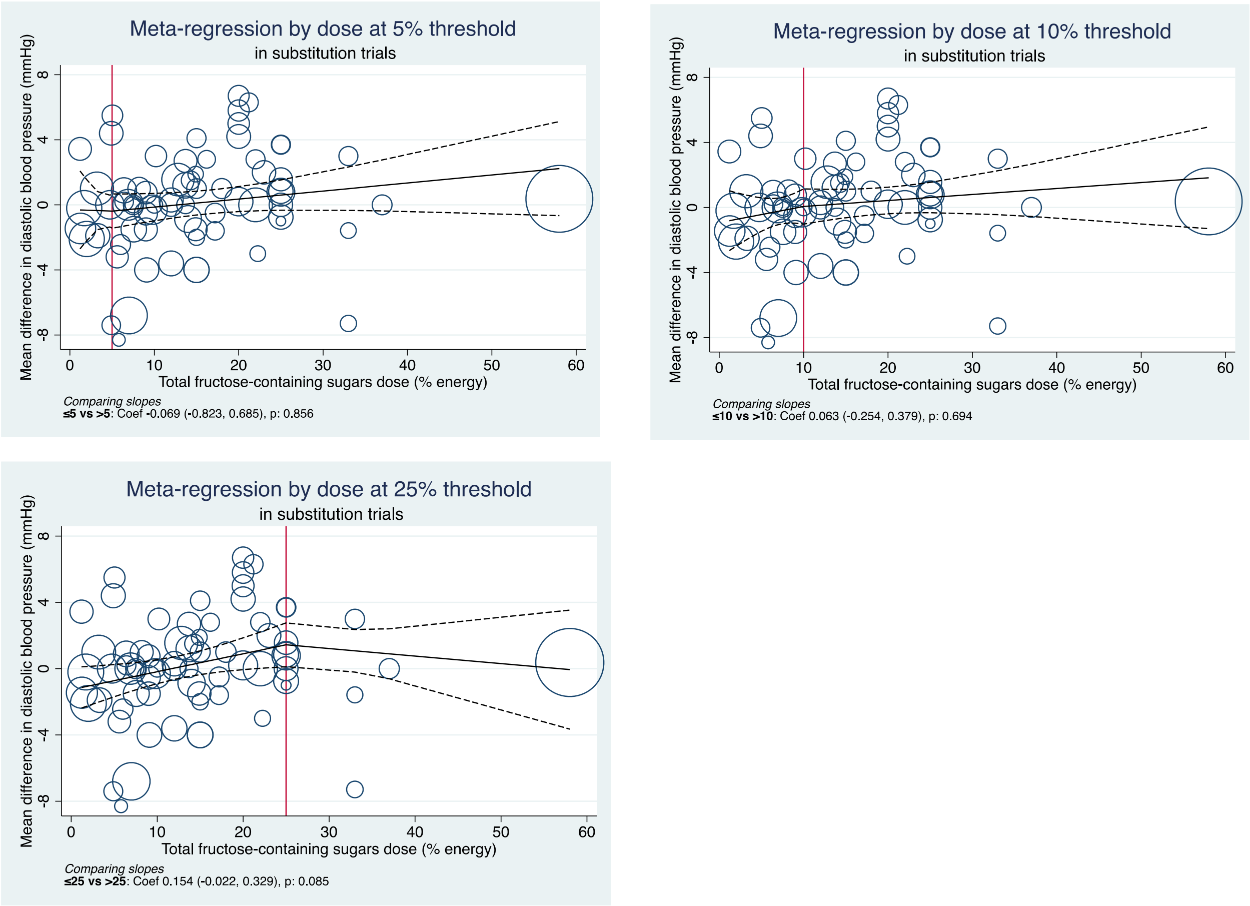

Individual trials are represented by the circles, with their weight in the overall analysis represented by the size of the circles. The horizontal straight line represents the estimate dose response for amount of fructose-containing sugars consumed (% of total energy intake), and the dashed lines represent the upper and lower 95% confidence intervals. The vertical straight lines represent the threshold knots.
coef=coefficient.

S110 Fig. Non-linear dose-response analysis using public thresholds of 5, 10, and 25% of energy for the effect of food sources of fructose-containing sugars dose (% of total energy) on DBP (mmHg) in addition trials.

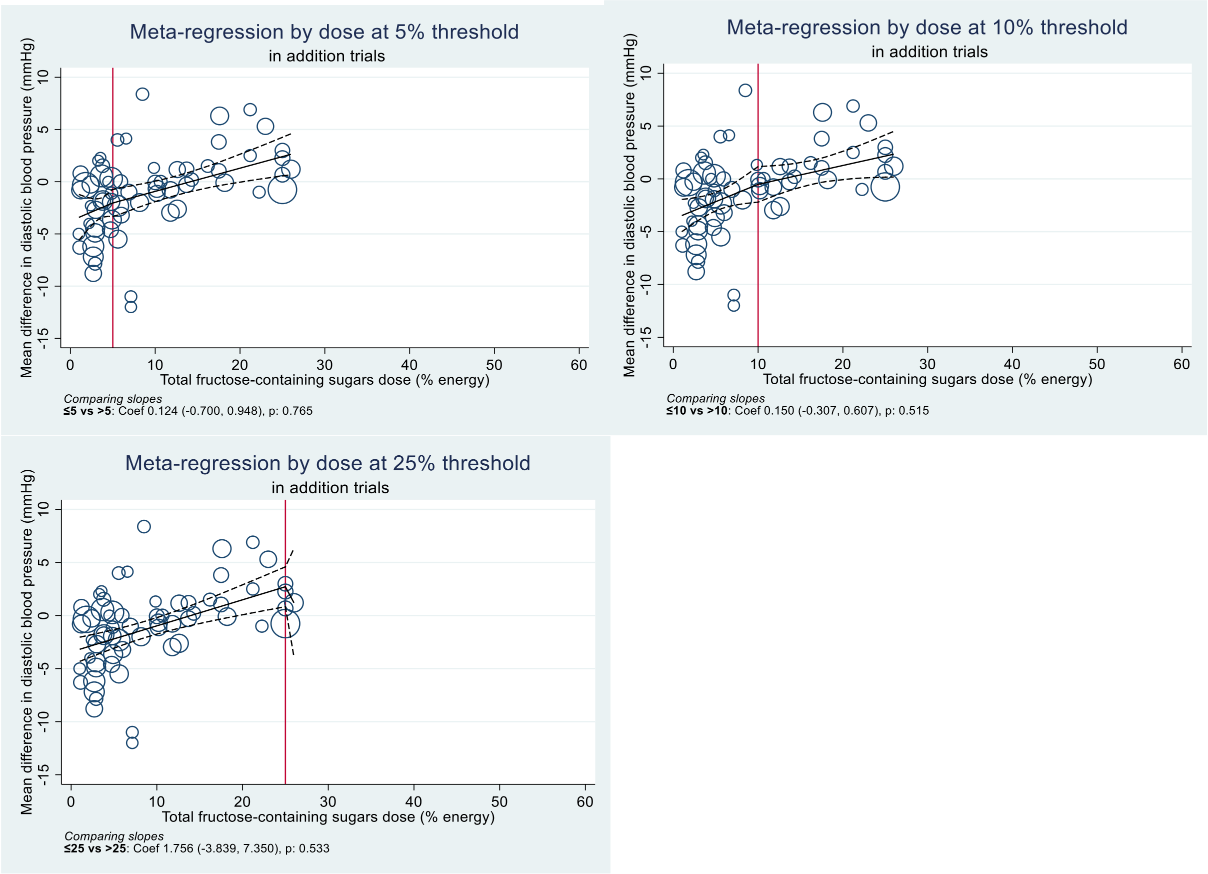

Individual trials are represented by the circles, with their weight in the overall analysis represented by the size of the circles. The horizontal straight line represents the estimate dose response for amount of fructose-containing sugars consumed (% of total energy intake), and the dashed lines represent the upper and lower 95% confidence intervals. The vertical straight lines represent the threshold knots.
coef=coefficient.

S111 Fig. Non-linear dose-response analysis using public thresholds of 5 and 10% of energy for the effect of food sources of fructose-containing sugars dose (% of total energy) on DBP (mmHg) in subtraction trials.

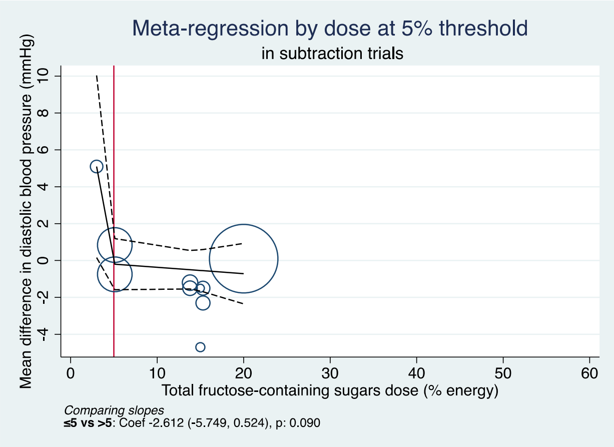

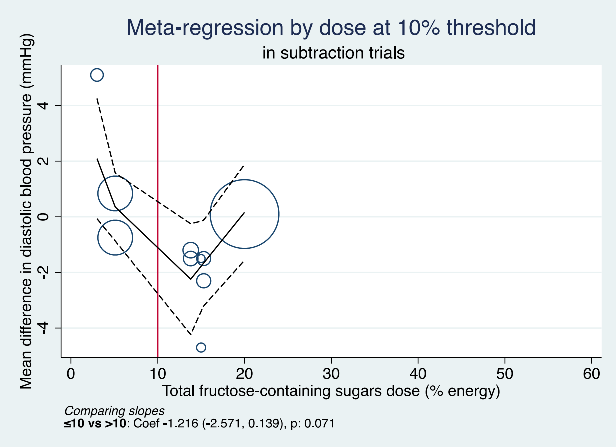

Individual trials are represented by the circles, with their weight in the overall analysis represented by the size of the circles. The horizontal straight line represents the estimate dose response for amount of fructose-containing sugars consumed (% of total energy intake), and the dashed lines represent the upper and lower 95% confidence intervals. The vertical straight lines represent the threshold knots.

Non-linear dose-response analysis using public thresholds of 25% was not conducted as there were no trials included which had a dose greater than 25% of total energy.

coef=coefficient.

S112 Fig. Non-linear dose-response analysis using public thresholds of 25% of energy for the effect of food sources of fructose-containing sugars dose (% of total energy) on DBP (mmHg) in *ad libitum* trials.

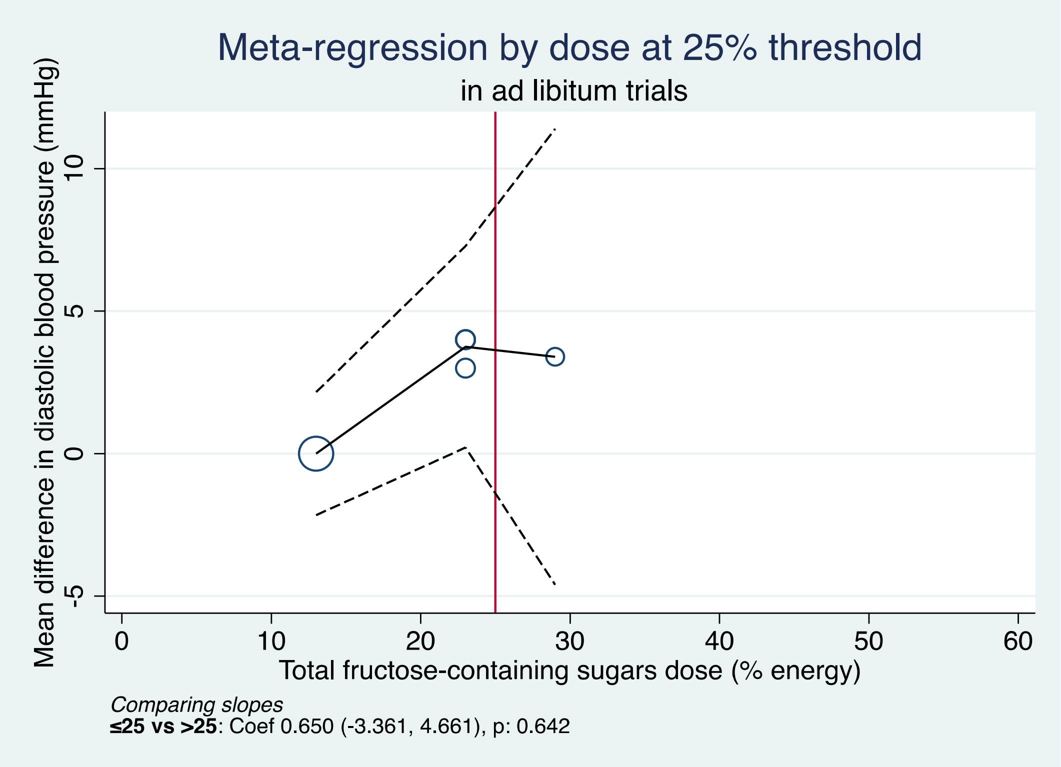

Individual trials are represented by the circles, with their weight in the overall analysis represented by the size of the circles. The horizontal straight line represents the estimate dose response for amount of fructose-containing sugars consumed (% of total energy intake), and the dashed lines represent the upper and lower 95% confidence intervals. The vertical straight lines represent the threshold knots.

Non-linear dose-response analysis using public thresholds of 5% and 10% of energy was not conducted as there were no trials included which had a dose less than 10% of total energy.

coef=coefficient.

S113 Fig. Non-linear dose-response analysis using public threshold of 10% of energy for the effect of SSB sugars dose (% of total energy) on SBP (mmHg) in substitution trials.

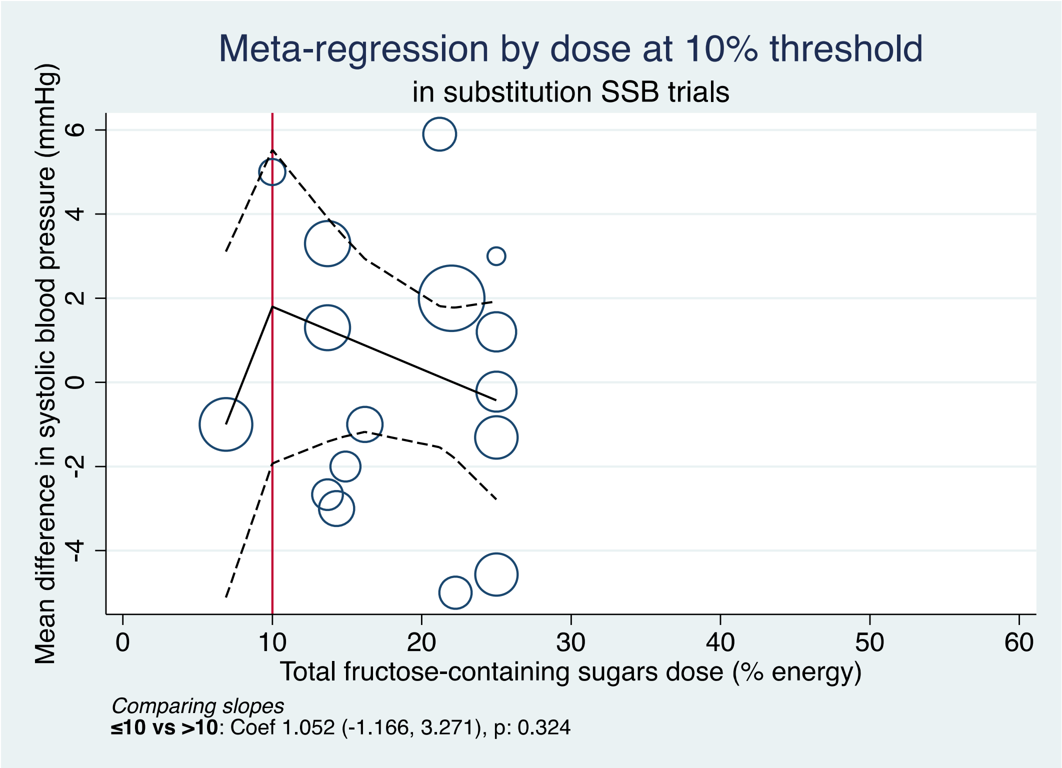

Individual trials are represented by the circles, with their weight in the overall analysis represented by the size of the circles. The horizontal straight line represents the estimate dose response for amount of fructose-containing sugars consumed (% of total energy intake), and the dashed lines represent the upper and lower 95% confidence intervals. The vertical straight lines represent the threshold knots.

Non-linear dose-response analysis using public thresholds of 5% of energy was not conducted as there were no trials included which had a dose less than 5% of total energy. Non-linear dose-response analysis using public thresholds of 25% of energy was not conducted as there were no trials included which had a dose greater than 25% of total energy.

coef=coefficient; SSB=sugar sweetened beverage.

S114 Fig. Non-linear dose-response analysis using public thresholds of 5 and 10% of energy for the effect of fruit sugars dose (% of total energy) on SBP (mmHg) in substitution trials.

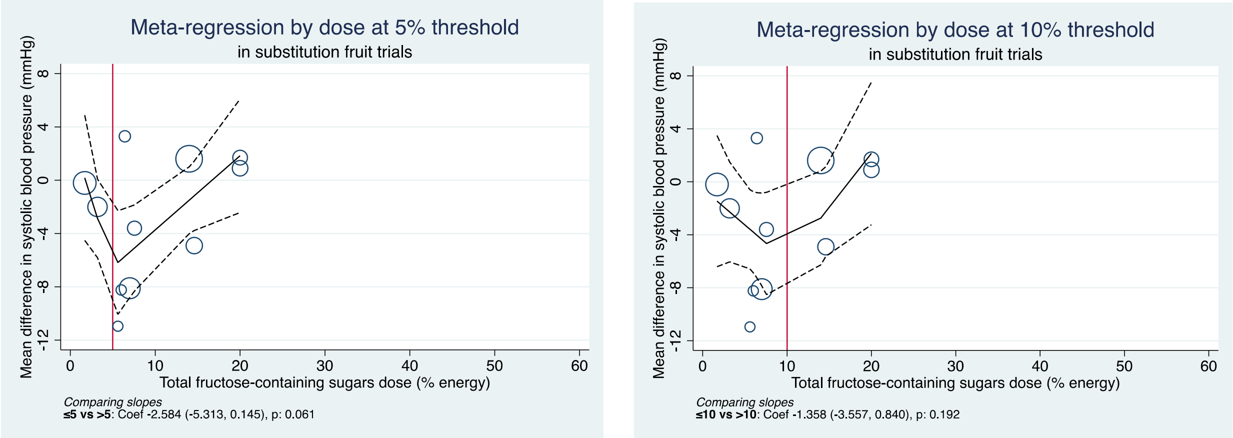

Individual trials are represented by the circles, with their weight in the overall analysis represented by the size of the circles. The horizontal straight line represents the estimate dose response for amount of fructose-containing sugars consumed (% of total energy intake), and the dashed lines represent the upper and lower 95% confidence intervals. The vertical straight lines represent the threshold knots.

Non-linear dose-response analysis using public thresholds of 25% of energy was not conducted as there were no trials included which had a dose greater than 25% of total energy.

coef=coefficient

S115 Fig. Non-linear dose-response analysis using public thresholds of 5 and 10% of energy for the effect of dried fruit sugars dose (% of total energy) on SBP (mmHg) in substitution trials.

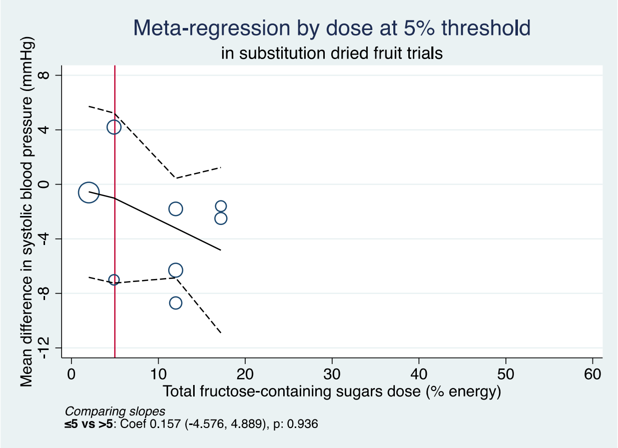

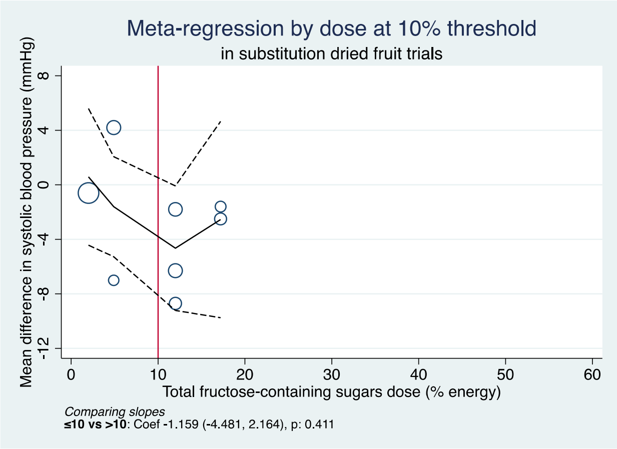

Individual trials are represented by the circles, with their weight in the overall analysis represented by the size of the circles. The horizontal straight line represents the estimate dose response for amount of fructose-containing sugars consumed (% of total energy intake), and the dashed lines represent the upper and lower 95% confidence intervals. The vertical straight lines represent the threshold knots.

Non-linear dose-response analysis using public thresholds of 25% of energy was not conducted as there were no trials included which had a dose greater than 25% of total energy.

coef=coefficient

S116 Fig. Non-linear dose-response analysis using public thresholds of 10 and 25% of energy for the effect of sweets and desserts sugars dose (% of total energy) on SBP (mmHg) in substitution trials.

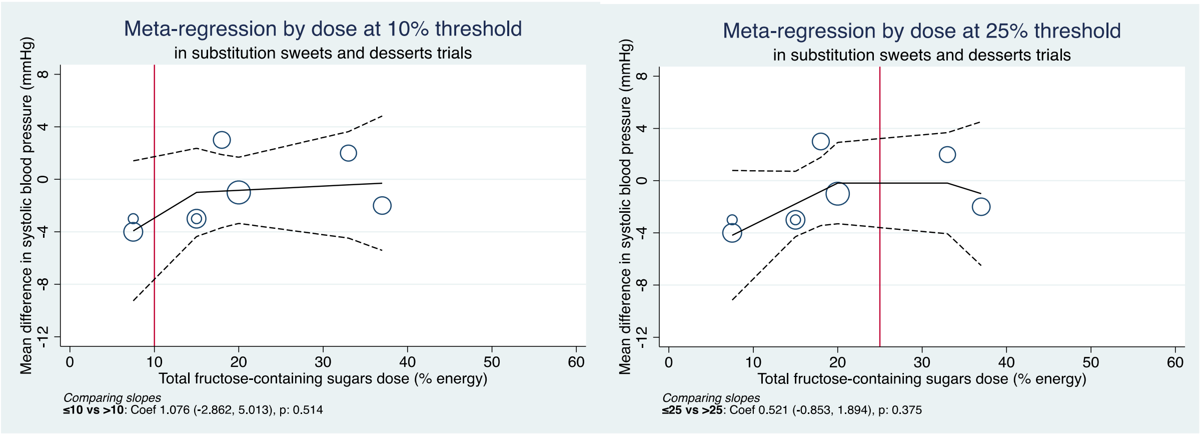

Individual trials are represented by the circles, with their weight in the overall analysis represented by the size of the circles. The horizontal straight line represents the estimate dose response for amount of fructose-containing sugars consumed (% of total energy intake), and the dashed lines represent the upper and lower 95% confidence intervals. The vertical straight lines represent the threshold knots.
Non-linear dose-response analysis using public thresholds of 5% of energy was not conducted as there were no trials included which had a dose less than 5% of total energy.
coef=coefficient

S117 Fig. Non-linear dose-response analysis using public thresholds of 10% of energy for the effect of added nutritive (caloric) sweetener sugars dose (% of total energy) on SBP (mmHg) in substitution trials.

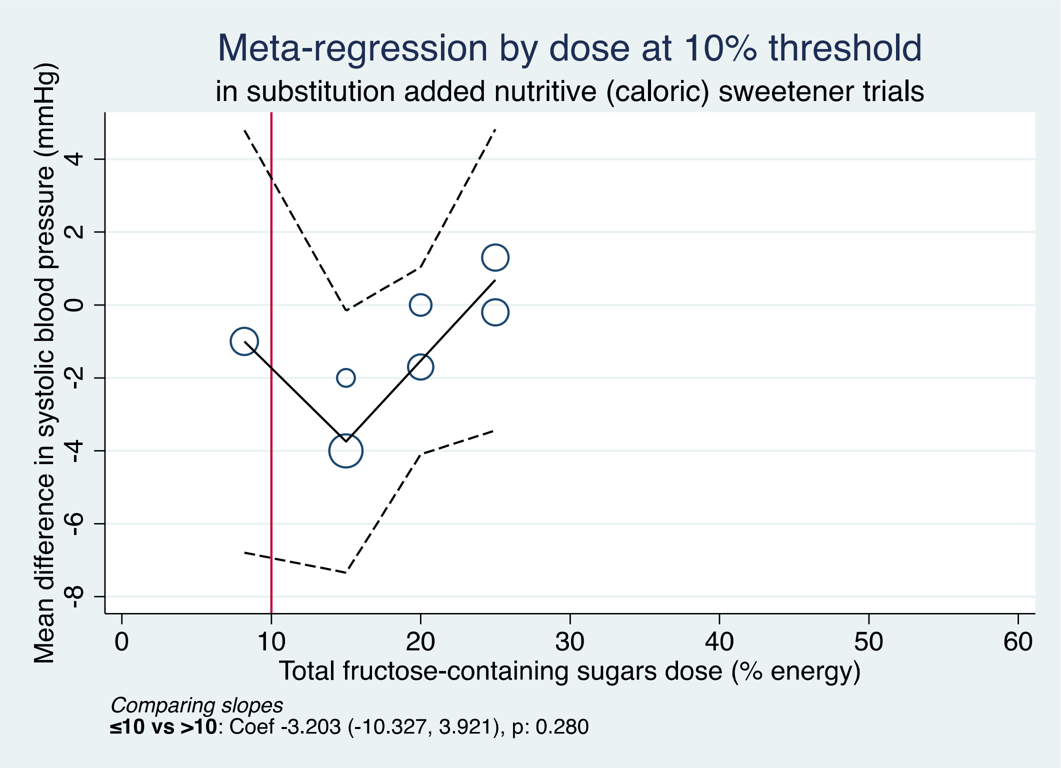

Individual trials are represented by the circles, with their weight in the overall analysis represented by the size of the circles. The horizontal straight line represents the estimate dose response for amount of fructose-containing sugars consumed (% of total energy intake), and the dashed lines represent the upper and lower 95% confidence intervals. The vertical straight lines represent the threshold knots.

Non-linear dose-response analysis using public thresholds of 5% of energy was not conducted as there were no trials included which had a dose less than 5% of total energy. Non-linear dose-response analysis using public thresholds of 25% of energy was not conducted as there were no trials included which had a dose greater than 25% of total energy.

coef=coefficient

S118 Fig. Non-linear dose-response analysis using public thresholds of 10 and 25% of energy for the effect of mixed sources (with SSBs) sugars dose (% of total energy) on SBP (mmHg) in substitution trials.

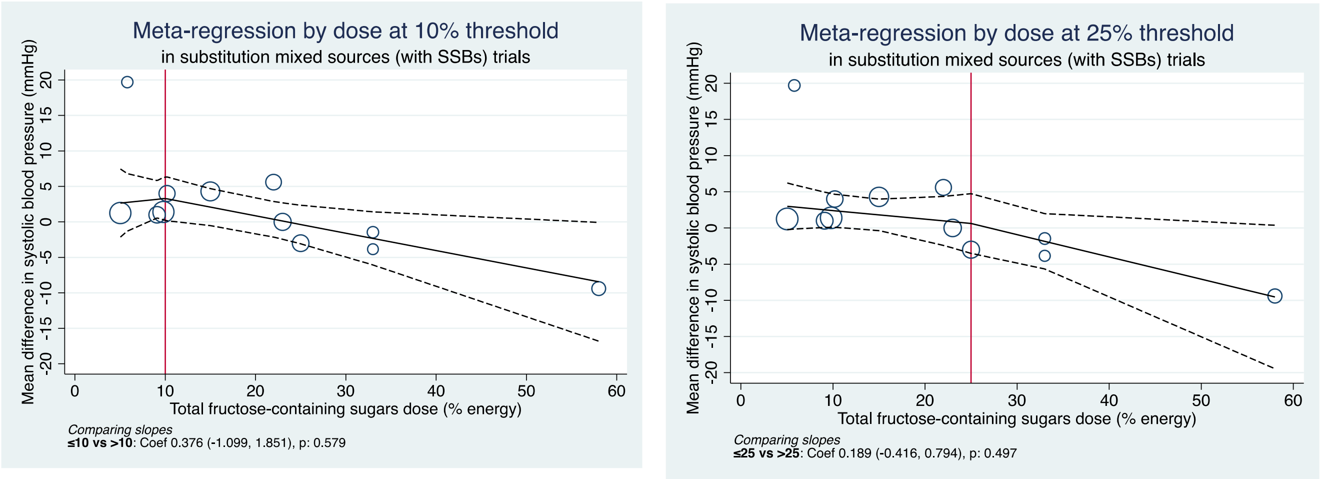

Individual trials are represented by the circles, with their weight in the overall analysis represented by the size of the circles. The horizontal straight line represents the estimate dose response for amount of fructose-containing sugars consumed (% of total energy intake), and the dashed lines represent the upper and lower 95% confidence intervals. The vertical straight lines represent the threshold knots.
Non-linear dose-response analysis using public thresholds of 5% of energy was not conducted as there were no trials included which had a dose less than 5% of total energy.
coef=coefficient; SSB=sugar sweetened beverage.

S119 Fig: Non-linear dose-response analysis using public thresholds of 10 and 25% of energy for the effect of SSB sugars dose (% of total energy) on SBP (mmHg) in addition trials.

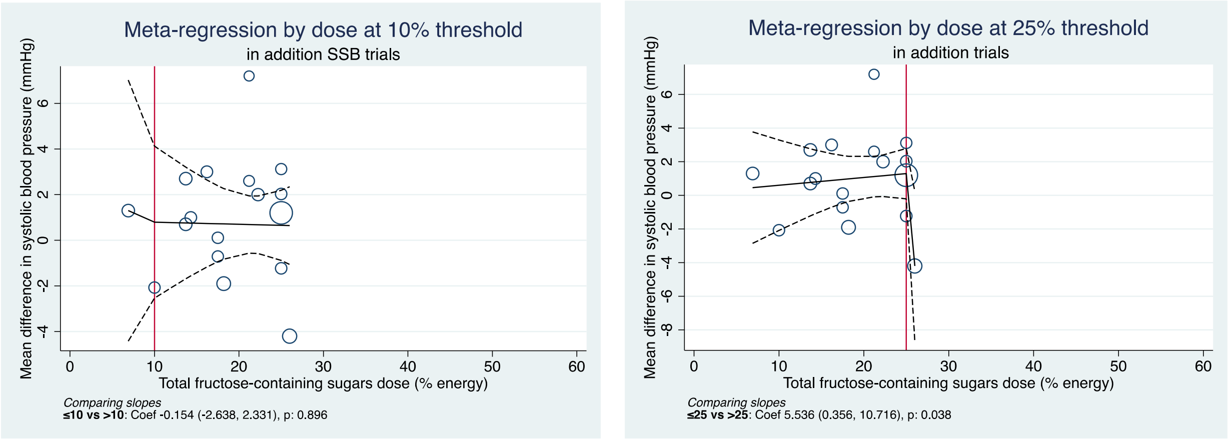

Individual trials are represented by the circles, with their weight in the overall analysis represented by the size of the circles. The horizontal straight line represents the estimate dose response for amount of fructose-containing sugars consumed (% of total energy intake), and the dashed lines represent the upper and lower 95% confidence intervals. The vertical straight lines represent the threshold knots.

Non-linear dose-response analysis using public thresholds of 5% of energy was not conducted as there were no trials included which had a dose less than 5% of total energy.

coef=coefficient; SSB=sugar sweetened beverage.

S120 Fig. Non-linear dose-response analysis using public thresholds of 5 and 10% of energy for the effect of 100% fruit juice sugars dose (% of total energy) on SBP (mmHg) in addition trials.

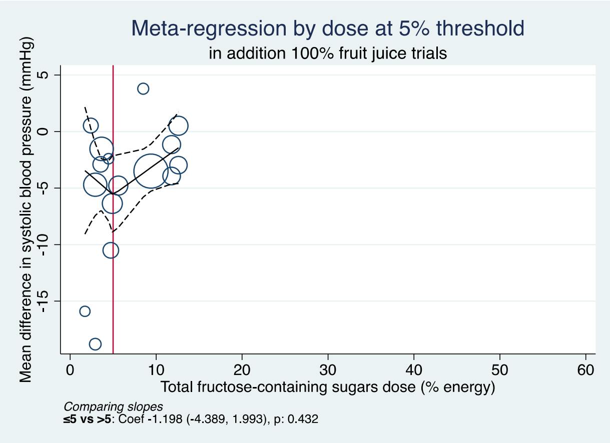

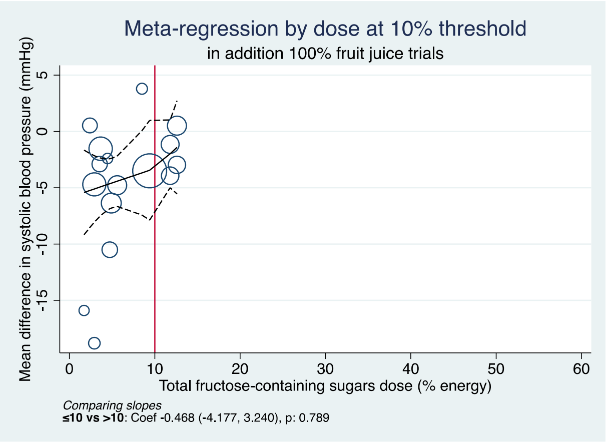

Individual trials are represented by the circles, with their weight in the overall analysis represented by the size of the circles. The horizontal straight line represents the estimate dose response for amount of fructose-containing sugars consumed (% of total energy intake), and the dashed lines represent the upper and lower 95% confidence intervals. The vertical straight lines represent the threshold knots.
Non-linear dose-response analysis using public thresholds of 25% of energy was not conducted as there were no trials included which had a dose greater than 25% of total energy.
coef=coefficient.

S121 Fig. Non-linear dose-response analysis using public thresholds of 5 and 10% of energy for the effect of fruit sugars dose (% of total energy) on SBP (mmHg) in addition trials.

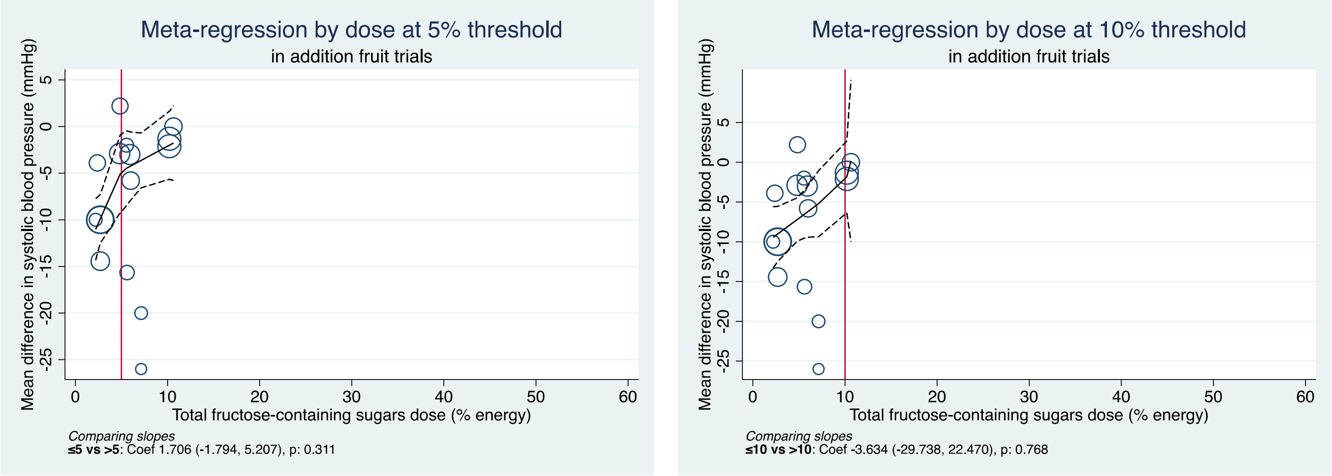

Individual trials are represented by the circles, with their weight in the overall analysis represented by the size of the circles. The horizontal straight line represents the estimate dose response for amount of fructose-containing sugars consumed (% of total energy intake), and the dashed lines represent the upper and lower 95% confidence intervals. The vertical straight lines represent the threshold knots.
Non-linear dose-response analysis using public thresholds of 25% of energy was not conducted as there were no trials included which had a dose greater than 25% of total energy.
coef=coefficient.

S122 Fig. Non-linear dose-response analysis using public thresholds of 5 and 10% of energy for the effect of dried fruit sugars dose (% of total energy) on SBP (mmHg) in addition trials.

Note: the sugars dose for each trial of dried fruit was <5% of energy, so these dose response analyses could not be conducted.

S123 Fig. Non-linear dose-response analysis using public thresholds of 5 and 10% of energy for the effect of SSB sugars dose (% of total energy) on SBP (mmHg) in subtraction trials.

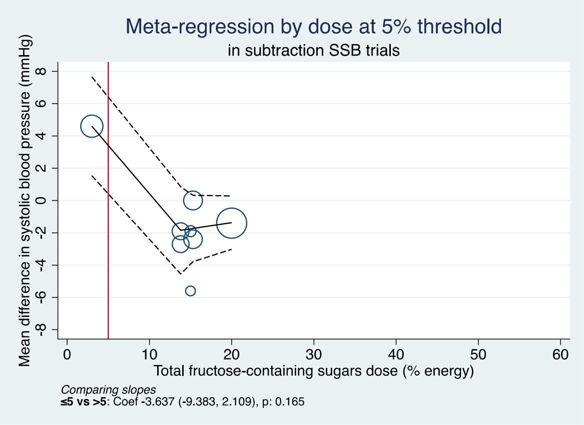

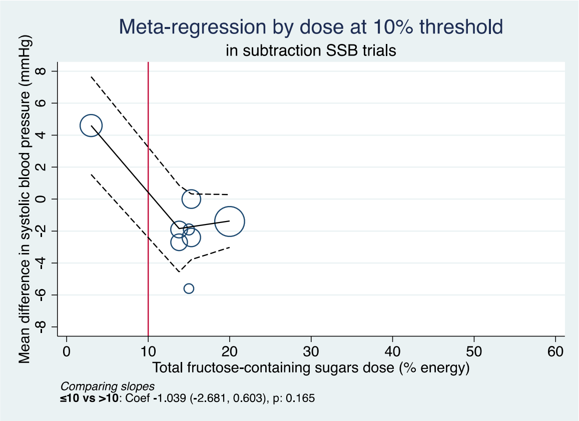

Individual trials are represented by the circles, with their weight in the overall analysis represented by the size of the circles. The horizontal straight line represents the estimate dose response for amount of fructose-containing sugars consumed (% of total energy intake), and the dashed lines represent the upper and lower 95% confidence intervals. The vertical straight lines represent the threshold knots.

Non-linear dose-response analysis using public thresholds of 25% of energy was not conducted as there were no trials included which had a dose greater than 25% of total energy.

coef=coefficient; SSB=sugar sweetened beverage.

S124 Fig. Non-linear dose-response analysis using public thresholds of 10 and 25% of energy for the effect of SSB sugars dose (% of total energy) on DBP (mmHg) in addition trials.

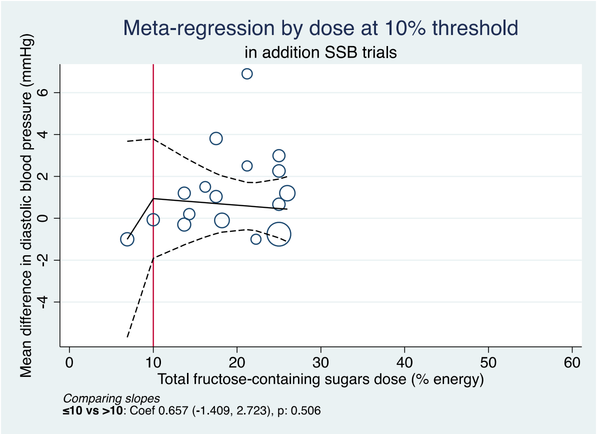

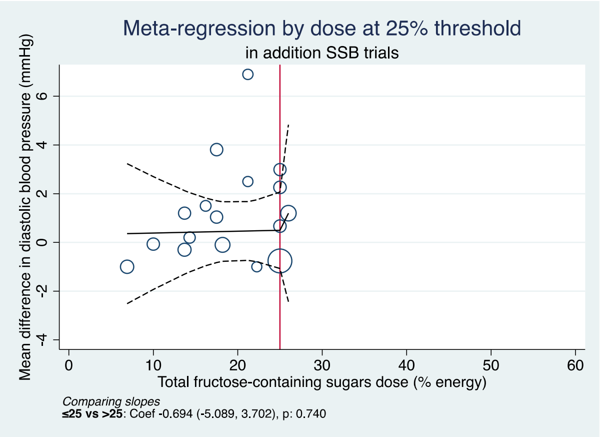

Individual trials are represented by the circles, with their weight in the overall analysis represented by the size of the circles. The horizontal straight line represents the estimate dose response for amount of fructose-containing sugars consumed (% of total energy intake), and the dashed lines represent the upper and lower 95% confidence intervals. The vertical straight lines represent the threshold knots.
Non-linear dose-response analysis using public thresholds of 5% of energy was not conducted as there were no trials included which had a dose less than 5% of total energy.
coef=coefficient; SSB=sugar sweetened beverage.

S125 Fig. Non-linear dose-response analysis using public thresholds of 5 and 10% of energy for the effect of 100% fruit juice sugars dose (% of total energy) on DBP (mmHg) in addition trials.

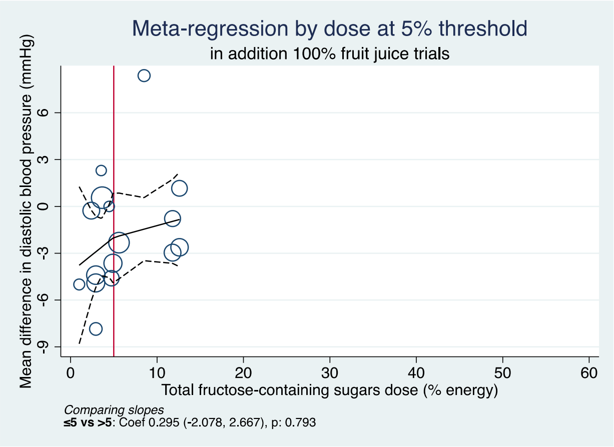

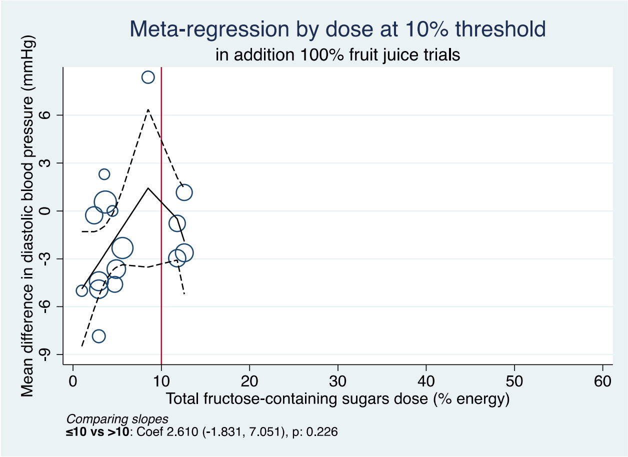

Individual trials are represented by the circles, with their weight in the overall analysis represented by the size of the circles. The horizontal straight line represents the estimate dose response for amount of fructose-containing sugars consumed (% of total energy intake), and the dashed lines represent the upper and lower 95% confidence intervals. The vertical straight lines represent the threshold knots.
Non-linear dose-response analysis using public thresholds of 25% of energy was not conducted as there were no trials included which had a dose greater than 25% of total energy.
coef=coefficient.

S126 Fig. Non-linear dose-response analysis using public thresholds of 5 and 10% of energy for the effect of fruit sugars dose (% of total energy) on DBP (mmHg) in addition trials.

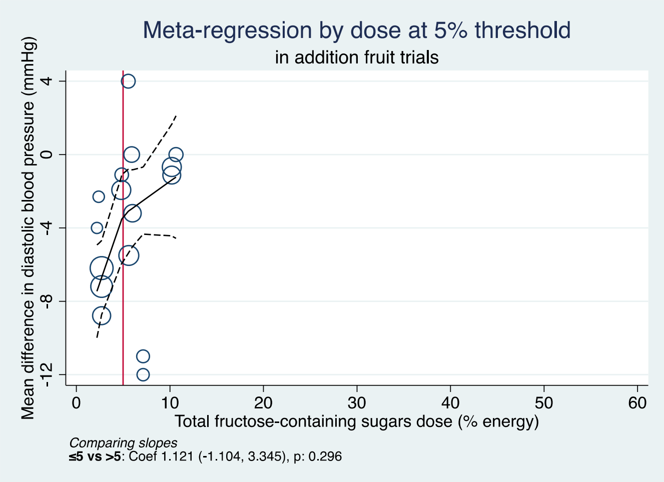

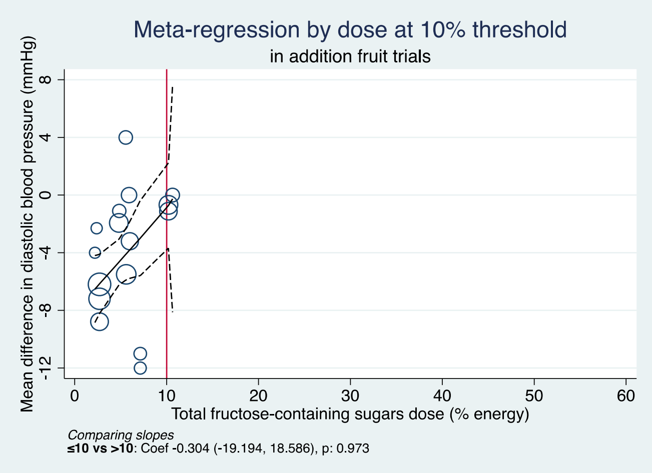

Individual trials are represented by the circles, with their weight in the overall analysis represented by the size of the circles. The horizontal straight line represents the estimate dose response for amount of fructose-containing sugars consumed (% of total energy intake), and the dashed lines represent the upper and lower 95% confidence intervals. The vertical straight lines represent the threshold knots.
Non-linear dose-response analysis using public thresholds of 25% of energy was not conducted as there were no trials included which had a dose greater than 25% of total energy.
coef=coefficient.

S127 Fig. Non-linear dose-response analysis using public thresholds of 5 and 10% of energy for the effect of dried fruit sugars dose (% of total energy) on DBP (mmHg) in addition trials.

Note: the sugars dose for each trial of dried fruit was <5% of energy, so these dose response analyses could not be conducted.

S128 Fig. Non-linear dose-response analysis using public thresholds of 5 and 10% of energy for the effect of SSB sugars dose (% of total energy) on DBP (mmHg) in subtraction trials.

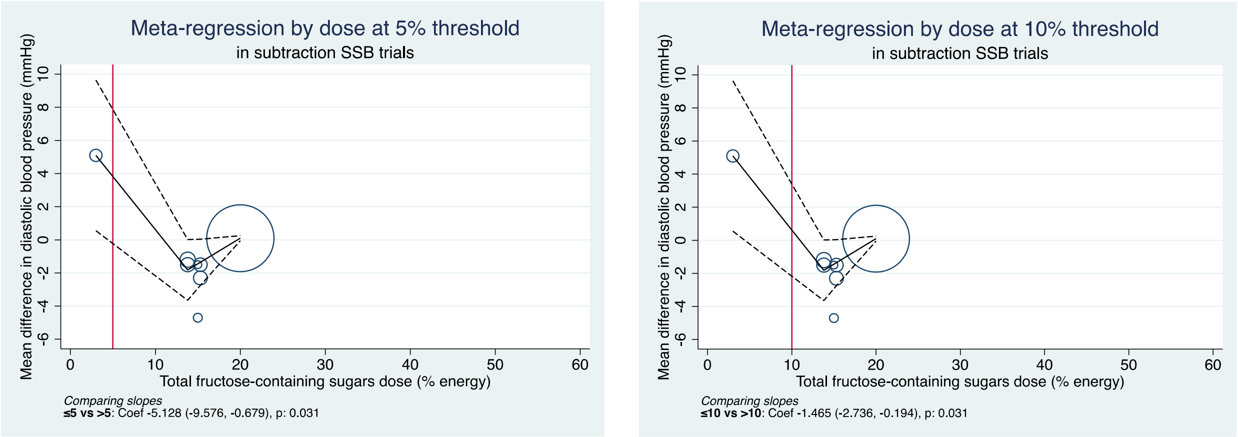

Individual trials are represented by the circles, with their weight in the overall analysis represented by the size of the circles. The horizontal straight line represents the estimate dose response for amount of fructose-containing sugars consumed (% of total energy intake), and the dashed lines represent the upper and lower 95% confidence intervals. The vertical straight lines represent the threshold knots.

Non-linear dose-response analysis using public thresholds of 25% of energy was not conducted as there were no trials included which had a dose greater than 25% of total energy.

coef=coefficient; SSB=sugar sweetened beverage.

S129 Fig. Publication bias funnel plots for the effect of important food sources of fructose-containing sugars on SBP (mmHg) in substitution trials.

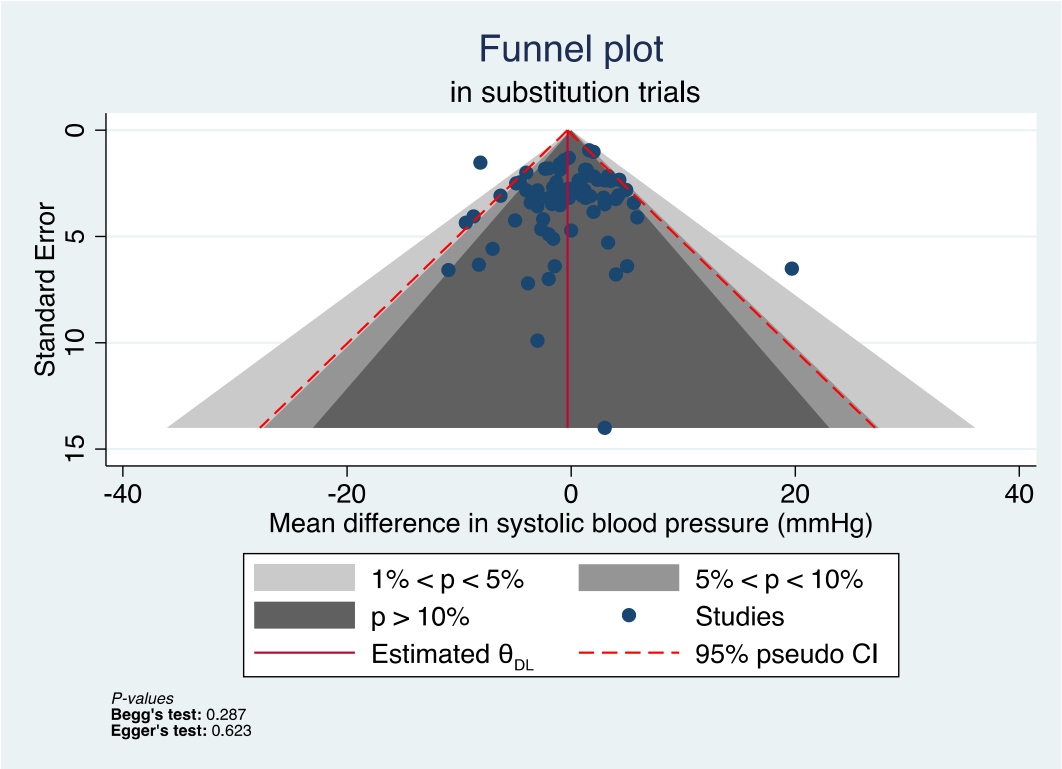

Contour-enhanced funnel plot is a scatterplot of each trial weighted mean difference on the x-axis with the standard error representing precision on the y-axis. The vertical solid red line represents the pooled effect estimate and the dashed red lines represent the pseudo-95% confidence limits. The blue dots represent individual trials. The contour regions define the regions for the test of significance of individual trial effect size for a given p-value range >0.100 (dark grey), 0.500 to <0.100 (medium grey), 0.010 to <0.500 (light grey), <0.0100 (white)]. The contour-enhanced funnel plots may suggest funnel-plot asymmetry is due to publication bias when less precise (smaller) trials are missing in the non-significant regions. Quantitative assessment of publication bias was also performed using Egger's and Begg's tests set at a significance level of p<0.100.
CI=confidence interval.

S130 Fig. Publication bias funnel plots for the effect of important food sources of fructose-containing sugars on SBP (mmHg) in addition trials.

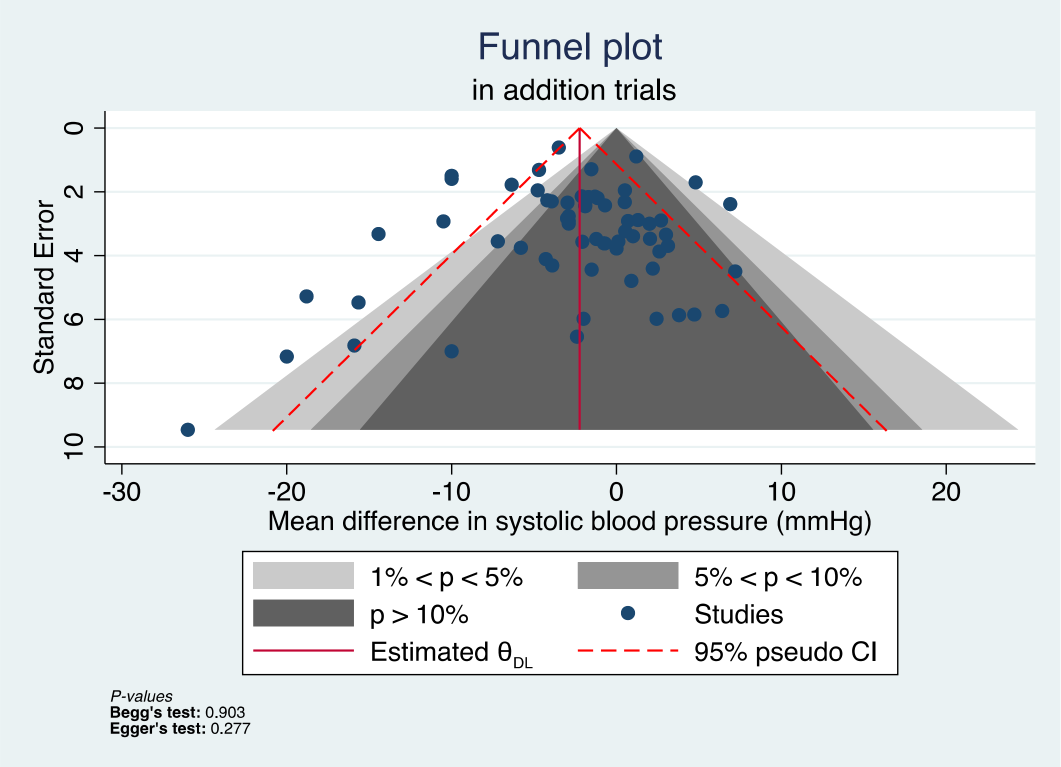

Contour-enhanced funnel plot is a scatterplot of each trial weighted mean difference on the x-axis with the standard error representing precision on the y-axis. The vertical solid red line represents the pooled effect estimate and the dashed red lines represent the pseudo-95% confidence limits. The blue dots represent individual trials. The contour regions define the regions for the test of significance of individual trial effect size for a given p-value range >0.100 (dark grey), 0.500 to <0.100 (medium grey), 0.010 to <0.500 (light grey), <0.0100 (white)]. The contour-enhanced funnel plots may suggest funnel-plot asymmetry is due to publication bias when less precise (smaller) trials are missing in the non-significant regions. Quantitative assessment of publication bias was also performed using Egger's and Begg's tests set at a significance level of p<0.100.
CI=confidence interval.

S131 Fig. Publication bias funnel plots for the effect of important food sources of fructose-containing sugars on SBP (mmHg) in subtraction trials.

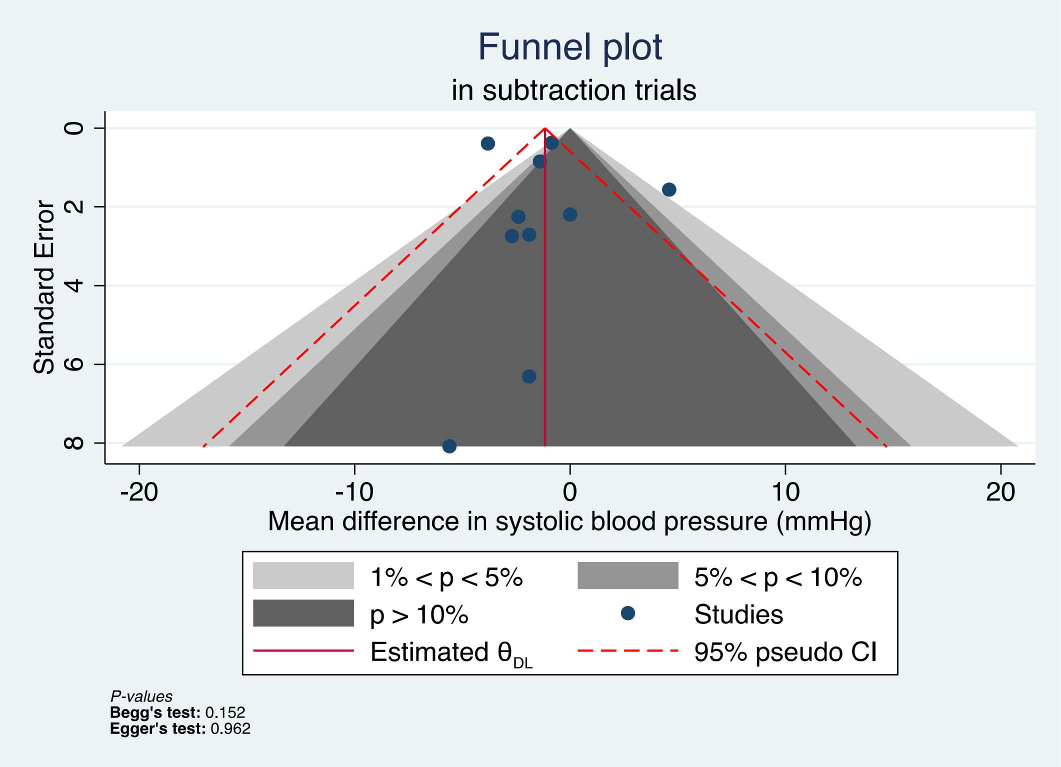

Contour-enhanced funnel plot is a scatterplot of each trial weighted mean difference on the x-axis with the standard error representing precision on the y-axis. The vertical solid red line represents the pooled effect estimate and the dashed red lines represent the pseudo-95% confidence limits. The blue dots represent individual trials. The contour regions define the regions for the test of significance of individual trial effect size for a given p-value range >0.100 (dark grey), 0.500 to <0.100 (medium grey), 0.010 to <0.500 (light grey), <0.0100 (white)]. The contour-enhanced funnel plots may suggest funnel-plot asymmetry is due to publication bias when less precise (smaller) trials are missing in the non-significant regions. Quantitative assessment of publication bias was also performed using Egger's and Begg's tests set at a significance level of p<0.100.
CI=confidence interval.

S132 Fig. Publication bias funnel plots for the effect of important food sources of fructose-containing sugars on DBP (mmHg) in substitution trials.

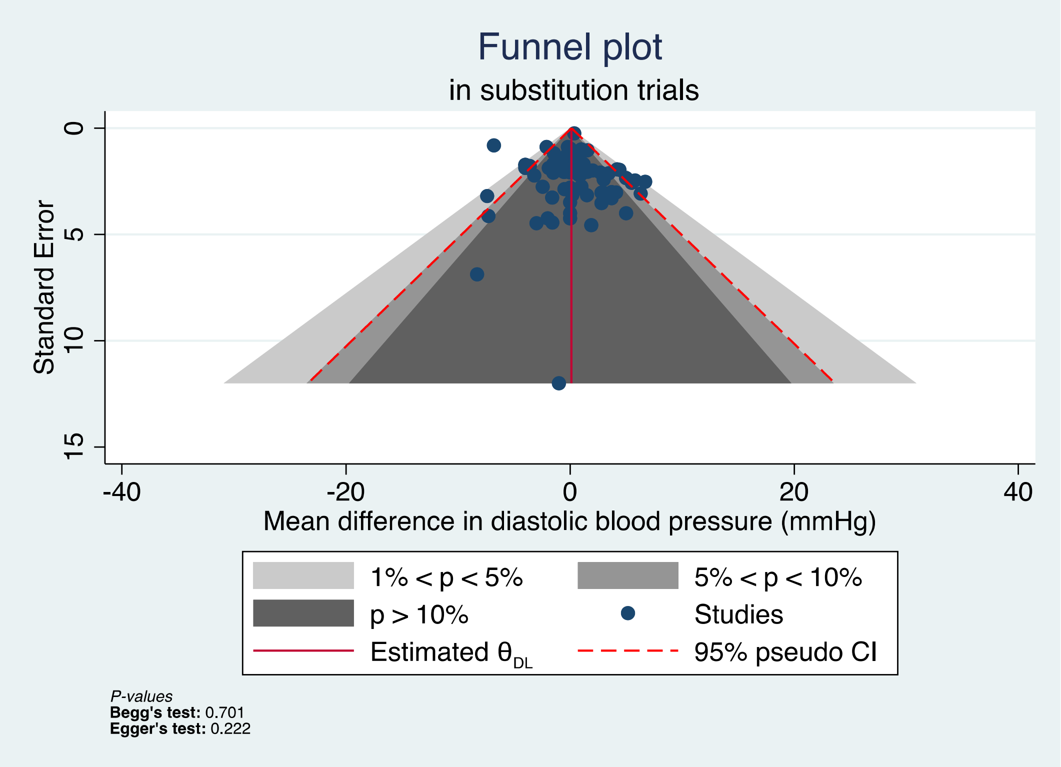

Contour-enhanced funnel plot is a scatterplot of each trial weighted mean difference on the x-axis with the standard error representing precision on the y-axis. The vertical solid red line represents the pooled effect estimate and the dashed red lines represent the pseudo-95% confidence limits. The blue dots represent individual trials. The contour regions define the regions for the test of significance of individual trial effect size for a given p-value range >0.100 (dark grey), 0.500 to <0.100 (medium grey), 0.010 to <0.500 (light grey), <0.0100 (white)]. The contour-enhanced funnel plots may suggest funnel-plot asymmetry is due to publication bias when less precise (smaller) trials are missing in the non-significant regions. Quantitative assessment of publication bias was also performed using Egger's and Begg's tests set at a significance level of p<0.100.
CI=confidence interval.

S133 Fig. Publication bias funnel plots for the effect of important food sources of fructose-containing sugars on DBP (mmHg) in addition trials.

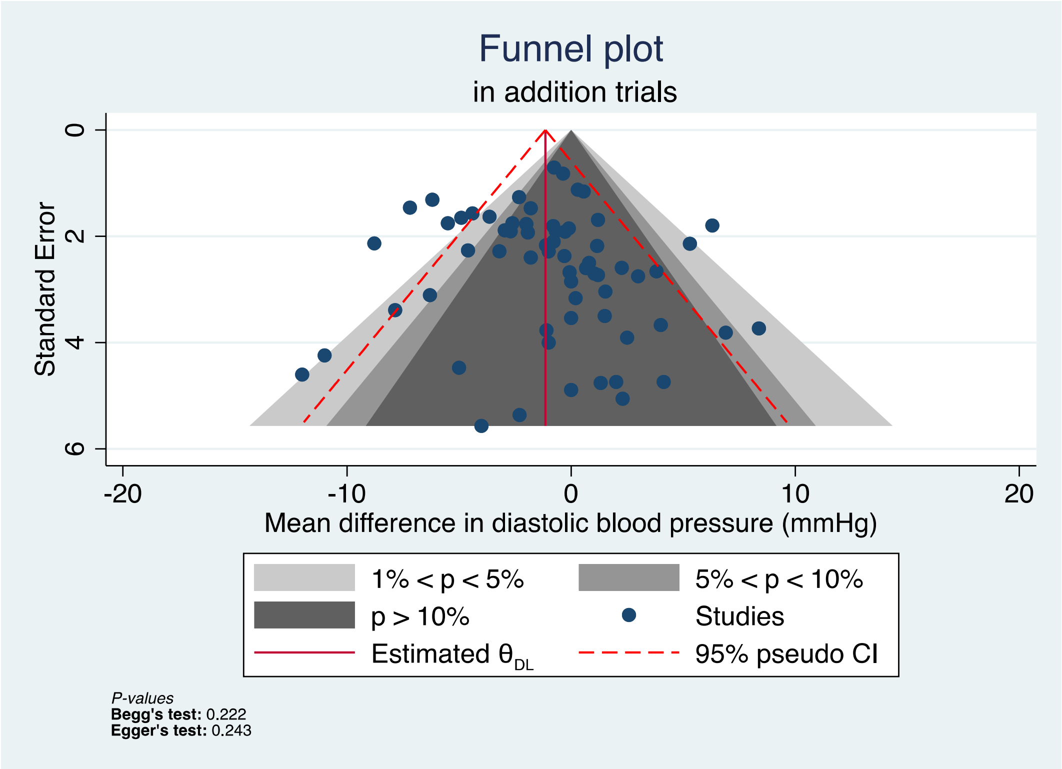

Contour-enhanced funnel plot is a scatterplot of each trial weighted mean difference on the x-axis with the standard error representing precision on the y-axis. The vertical solid red line represents the pooled effect estimate and the dashed red lines represent the pseudo-95% confidence limits. The blue dots represent individual trials. The contour regions define the regions for the test of significance of individual trial effect size for a given p-value range >0.100 (dark grey), 0.500 to <0.100 (medium grey), 0.010 to <0.500 (light grey), <0.0100 (white)]. The contour-enhanced funnel plots may suggest funnel-plot asymmetry is due to publication bias when less precise (smaller) trials are missing in the non-significant regions. Quantitative assessment of publication bias was also performed using Egger's and Begg's tests set at a significance level of p<0.100.
CI=confidence interval.

S134 Fig. Publication bias funnel plots for the effect of important food sources of fructose-containing sugars on DBP (mmHg) in subtraction trials.

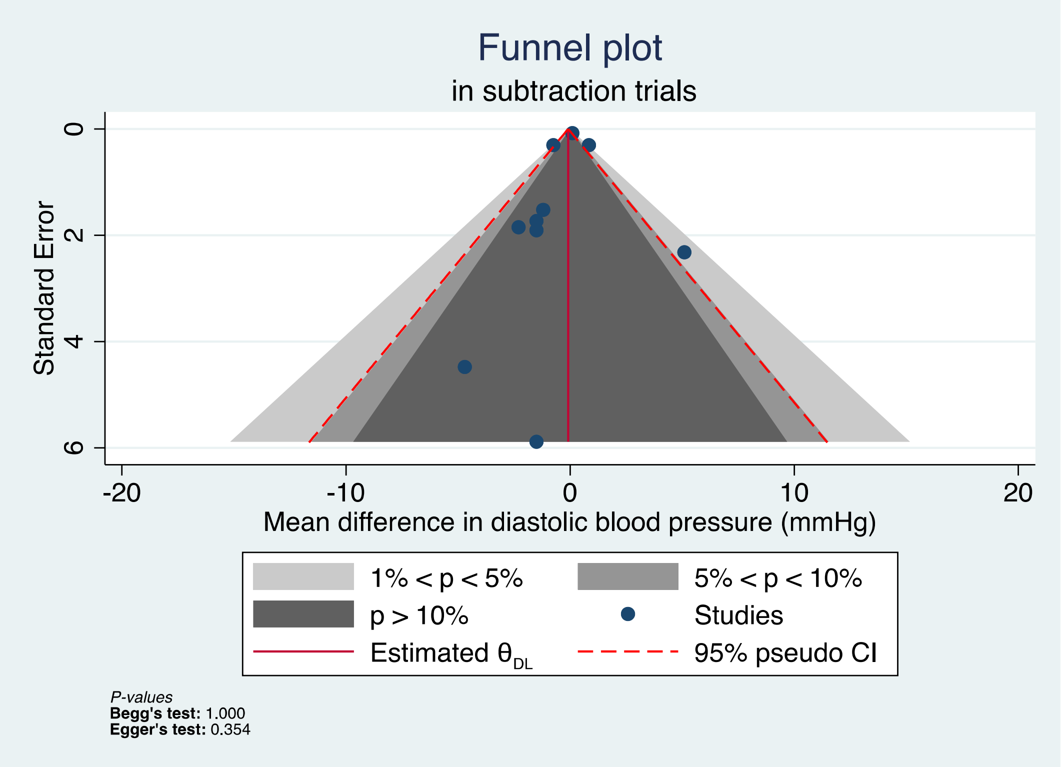

Contour-enhanced funnel plot is a scatterplot of each trial weighted mean difference on the x-axis with the standard error representing precision on the y-axis. The vertical solid red line represents the pooled effect estimate and the dashed red lines represent the pseudo-95% confidence limits. The blue dots represent individual trials. The contour regions define the regions for the test of significance of individual trial effect size for a given p-value range >0.100 (dark grey), 0.500 to <0.100 (medium grey), 0.010 to <0.500 (light grey), <0.0100 (white)]. The contour-enhanced funnel plots may suggest funnel-plot asymmetry is due to publication bias when less precise (smaller) trials are missing in the non-significant regions. Quantitative assessment of publication bias was also performed using Egger's and Begg's tests set at a significance level of p<0.100.
CI=confidence interval.

S135 Fig. Publication bias funnel plots for the effect of SSBs on SBP (mmHg) in substitution trials.

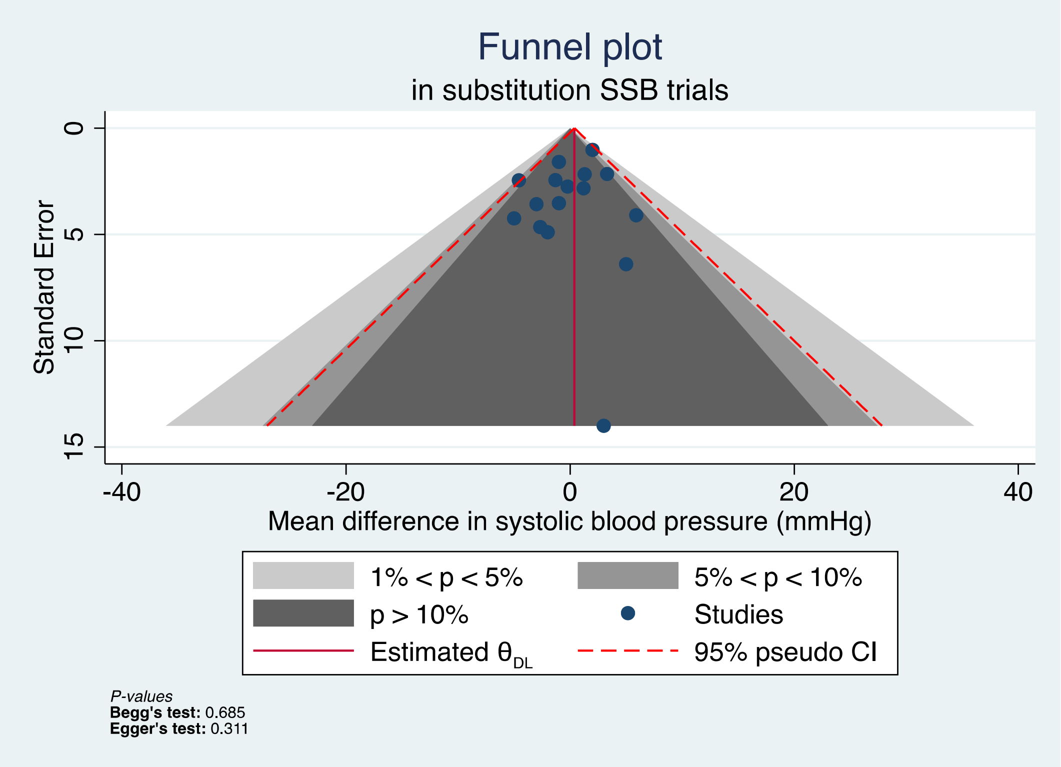

Contour-enhanced funnel plot is a scatterplot of each trial weighted mean difference on the x-axis with the standard error representing precision on the y-axis. The vertical solid red line represents the pooled effect estimate and the dashed red lines represent the pseudo-95% confidence limits. The blue dots represent individual trials. The contour regions define the regions for the test of significance of individual trial effect size for a given p-value range >0.100 (dark grey), 0.500 to <0.100 (medium grey), 0.010 to <0.500 (light grey), <0.0100 (white)]. The contour-enhanced funnel plots may suggest funnel-plot asymmetry is due to publication bias when less precise (smaller) trials are missing in the non-significant regions. Quantitative assessment of publication bias was also performed using Egger's and Begg's tests set at a significance level of p<0.100.
CI=confidence interval; SSB=sugar sweetened beverage.

S136 Fig. Publication bias funnel plots for the effect of fruit on SBP (mmHg) in substitution trials.

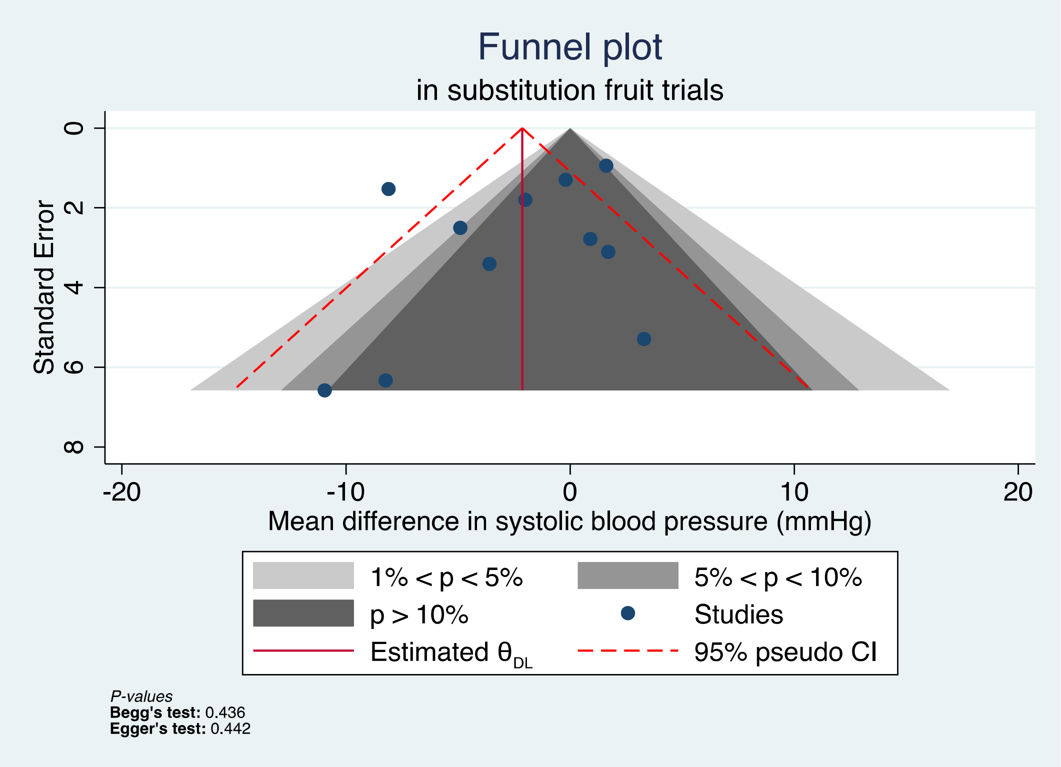

Contour-enhanced funnel plot is a scatterplot of each trial weighted mean difference on the x-axis with the standard error representing precision on the y-axis. The vertical solid red line represents the pooled effect estimate and the dashed red lines represent the pseudo-95% confidence limits. The blue dots represent individual trials. The contour regions define the regions for the test of significance of individual trial effect size for a given p-value range >0.100 (dark grey), 0.500 to <0.100 (medium grey), 0.010 to <0.500 (light grey), <0.0100 (white)]. The contour-enhanced funnel plots may suggest funnel-plot asymmetry is due to publication bias when less precise (smaller) trials are missing in the non-significant regions. Quantitative assessment of publication bias was also performed using Egger's and Begg's tests set at a significance level of p<0.100.
CI=confidence interval.

S137 Fig. Publication bias funnel plots for the effect of mixed sources (with SSBs) on SBP (mmHg) in substitution trials.

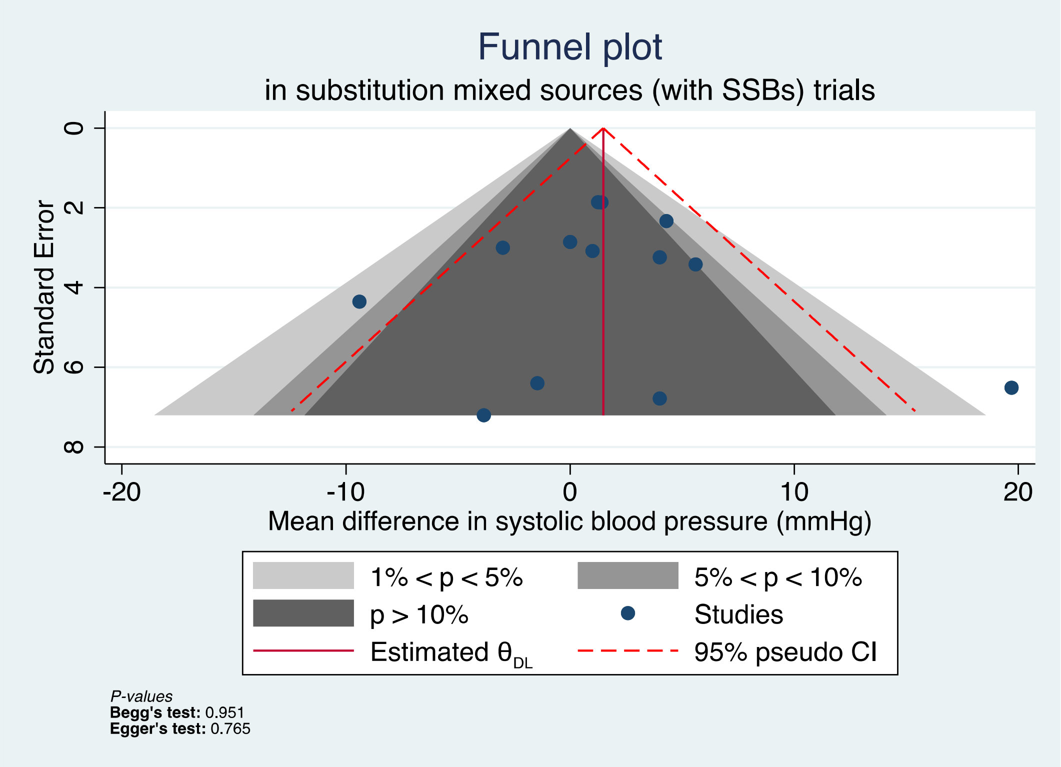

Contour-enhanced funnel plot is a scatterplot of each trial weighted mean difference on the x-axis with the standard error representing precision on the y-axis. The vertical solid red line represents the pooled effect estimate and the dashed red lines represent the pseudo-95% confidence limits. The blue dots represent individual trials. The contour regions define the regions for the test of significance of individual trial effect size for a given p-value range >0.100 (dark grey), 0.500 to <0.100 (medium grey), 0.010 to <0.500 (light grey), <0.0100 (white)]. The contour-enhanced funnel plots may suggest funnel-plot asymmetry is due to publication bias when less precise (smaller) trials are missing in the non-significant regions. Quantitative assessment of publication bias was also performed using Egger's and Begg's tests set at a significance level of p<0.100.
CI=confidence interval; SSB=sugar sweetened beverage.

S138 Fig. Publication bias funnel plots for the effect of SSBs on SBP (mmHg) in addition trials.

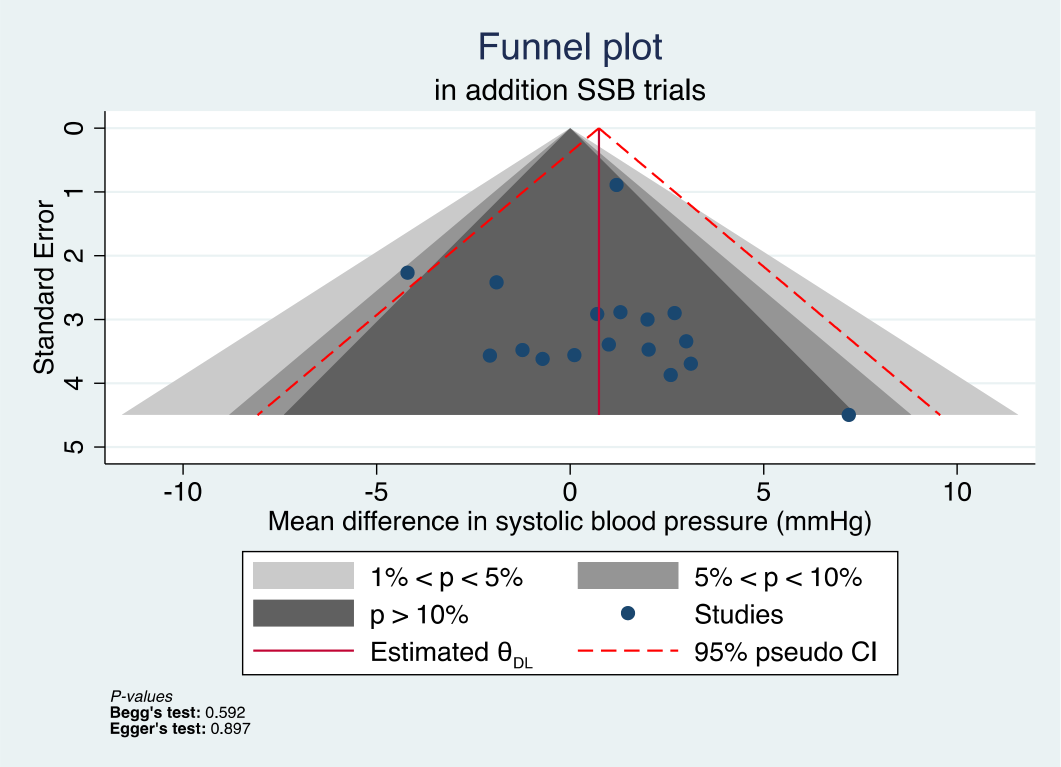

Contour-enhanced funnel plot is a scatterplot of each trial weighted mean difference on the x-axis with the standard error representing precision on the y-axis. The vertical solid red line represents the pooled effect estimate and the dashed red lines represent the pseudo-95% confidence limits. The blue dots represent individual trials. The contour regions define the regions for the test of significance of individual trial effect size for a given p-value range >0.100 (dark grey), 0.500 to <0.100 (medium grey), 0.010 to <0.500 (light grey), <0.0100 (white)]. The contour-enhanced funnel plots may suggest funnel-plot asymmetry is due to publication bias when less precise (smaller) trials are missing in the non-significant regions. Quantitative assessment of publication bias was also performed using Egger's and Begg's tests set at a significance level of p<0.100.
CI=confidence interval; SSB=sugar sweetened beverage.

S139 Fig. Publication bias funnel plots for the effect of 100% fruit juice on SBP (mmHg) in addition trials.

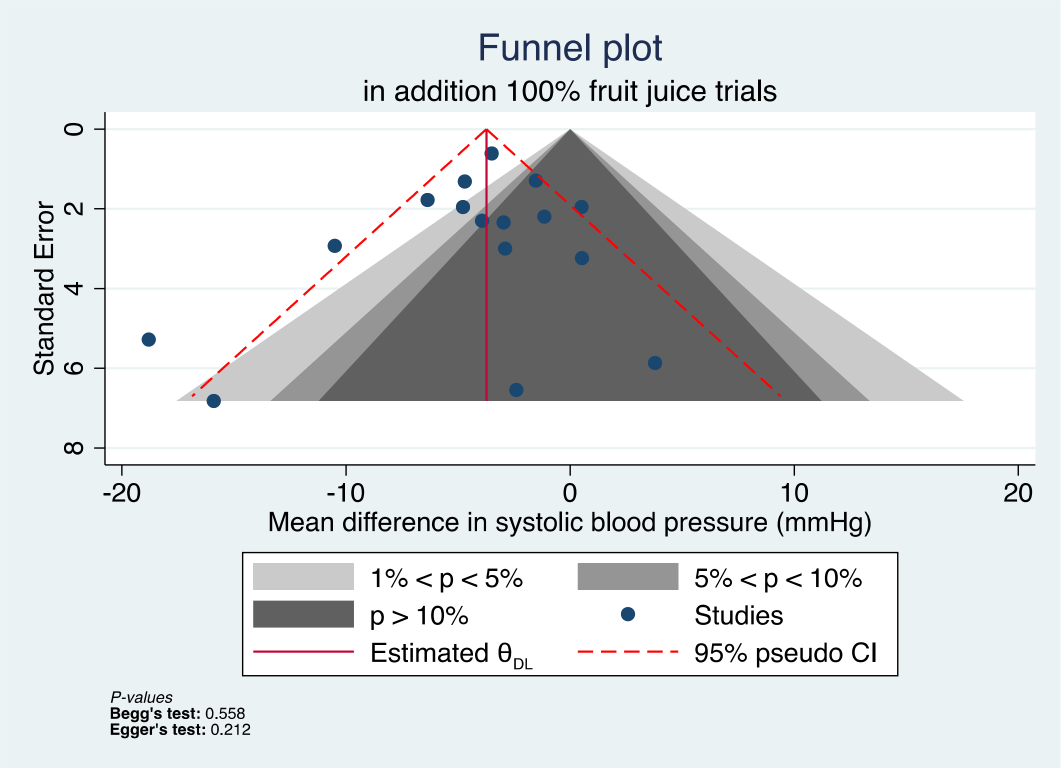

Contour-enhanced funnel plot is a scatterplot of each trial weighted mean difference on the x-axis with the standard error representing precision on the y-axis. The vertical solid red line represents the pooled effect estimate and the dashed red lines represent the pseudo-95% confidence limits. The blue dots represent individual trials. The contour regions define the regions for the test of significance of individual trial effect size for a given p-value range >0.100 (dark grey), 0.500 to <0.100 (medium grey), 0.010 to <0.500 (light grey), <0.0100 (white)]. The contour-enhanced funnel plots may suggest funnel-plot asymmetry is due to publication bias when less precise (smaller) trials are missing in the non-significant regions. Quantitative assessment of publication bias was also performed using Egger's and Begg's tests set at a significance level of p<0.100.
CI=confidence interval.

S140 Fig. Publication bias funnel plots for the effect of fruit on SBP (mmHg) in addition trials.

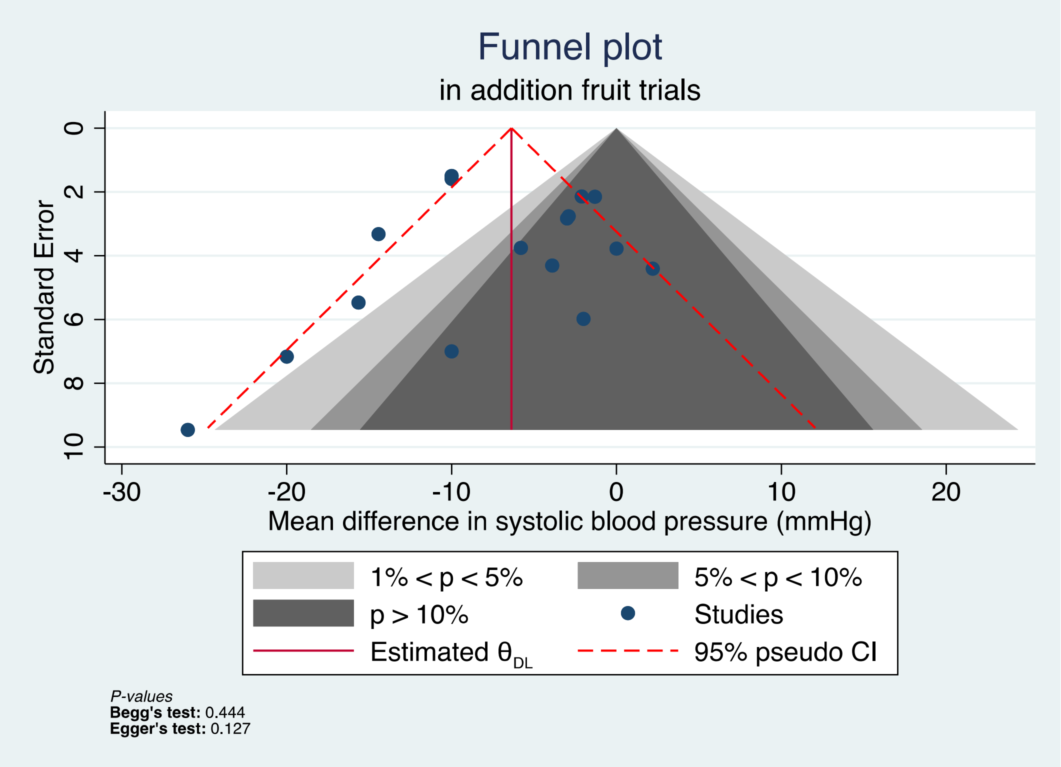

Contour-enhanced funnel plot is a scatterplot of each trial weighted mean difference on the x-axis with the standard error representing precision on the y-axis. The vertical solid red line represents the pooled effect estimate and the dashed red lines represent the pseudo-95% confidence limits. The blue dots represent individual trials. The contour regions define the regions for the test of significance of individual trial effect size for a given p-value range >0.100 (dark grey), 0.500 to <0.100 (medium grey), 0.010 to <0.500 (light grey), <0.0100 (white)]. The contour-enhanced funnel plots may suggest funnel-plot asymmetry is due to publication bias when less precise (smaller) trials are missing in the non-significant regions. Quantitative assessment of publication bias was also performed using Egger's and Begg's tests set at a significance level of p<0.100.
CI=confidence interval.

S141 Fig. Publication bias funnel plots for the effect of SSBs on DBP (mmHg) in addition trials.

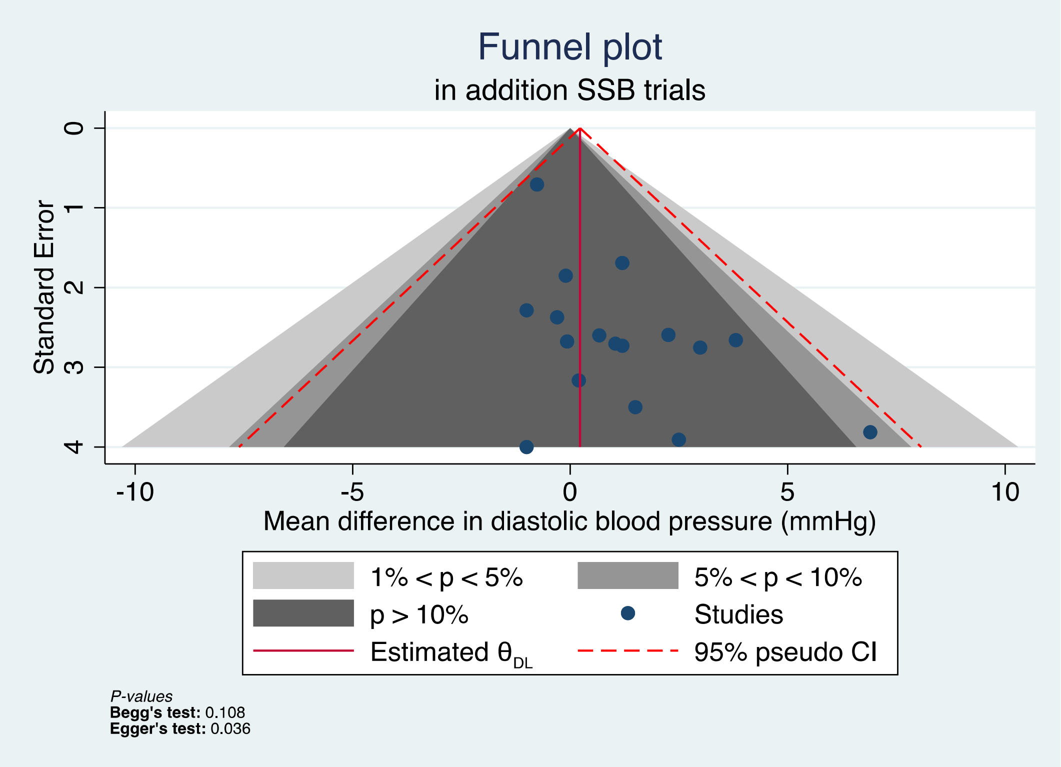

Contour-enhanced funnel plot is a scatterplot of each trial weighted mean difference on the x-axis with the standard error representing precision on the y-axis. The vertical solid red line represents the pooled effect estimate and the dashed red lines represent the pseudo-95% confidence limits. The blue dots represent individual trials. The contour regions define the regions for the test of significance of individual trial effect size for a given p-value range >0.100 (dark grey), 0.500 to <0.100 (medium grey), 0.010 to <0.500 (light grey), <0.0100 (white)]. The contour-enhanced funnel plots may suggest funnel-plot asymmetry is due to publication bias when less precise (smaller) trials are missing in the non-significant regions. Quantitative assessment of publication bias was also performed using Egger's and Begg's tests set at a significance level of p<0.100.
CI=confidence interval; SSB=sugar sweetened beverage.

S142 Fig. Trim and Fill funnel plot for the effect of SSBs on DBP (mmHg) in addition trials.

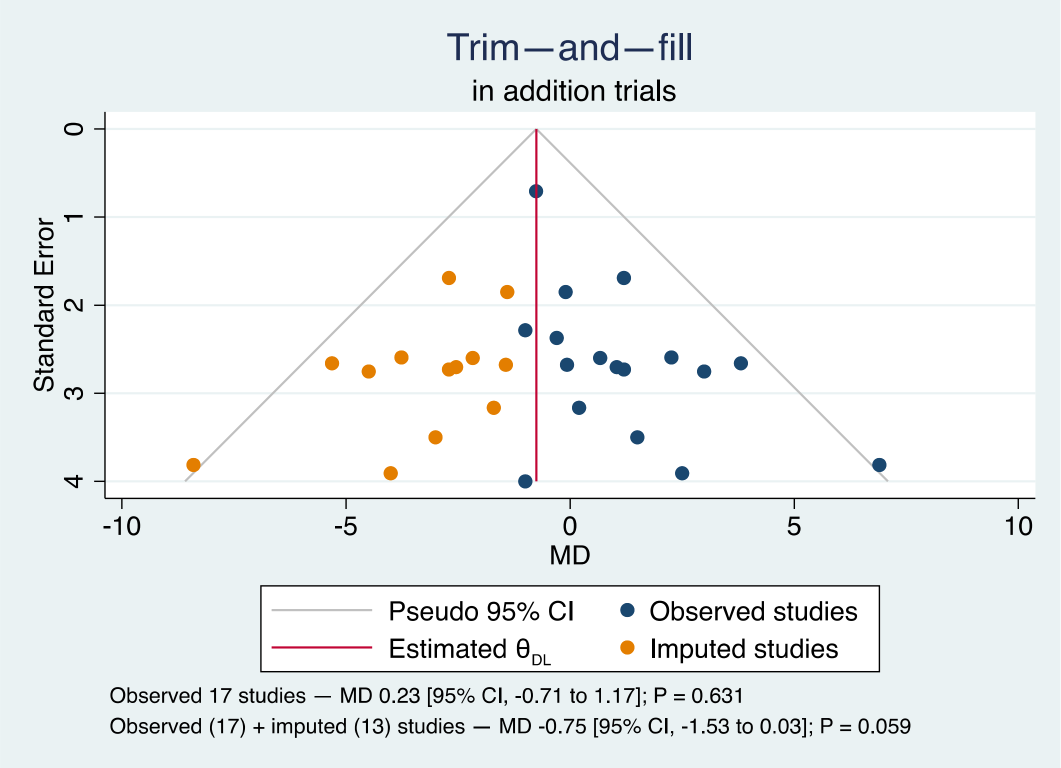

The horizontal line represents the pooled effect estimate expressed as mean difference. The diagonal lines represent the pseudo-95% confidence limits, the circles represent the effect estimate for each included trial, and squares represent the effect estimate for each imputed “missed” study. Imputed random mean difference is provided, p<0.100 is considered evidence of small-study effects.
CI=confidence interval; MD=mean difference

S143 Fig. Publication bias funnel plots for the effect of 100% fruit juice on DBP (mmHg) in addition trials.

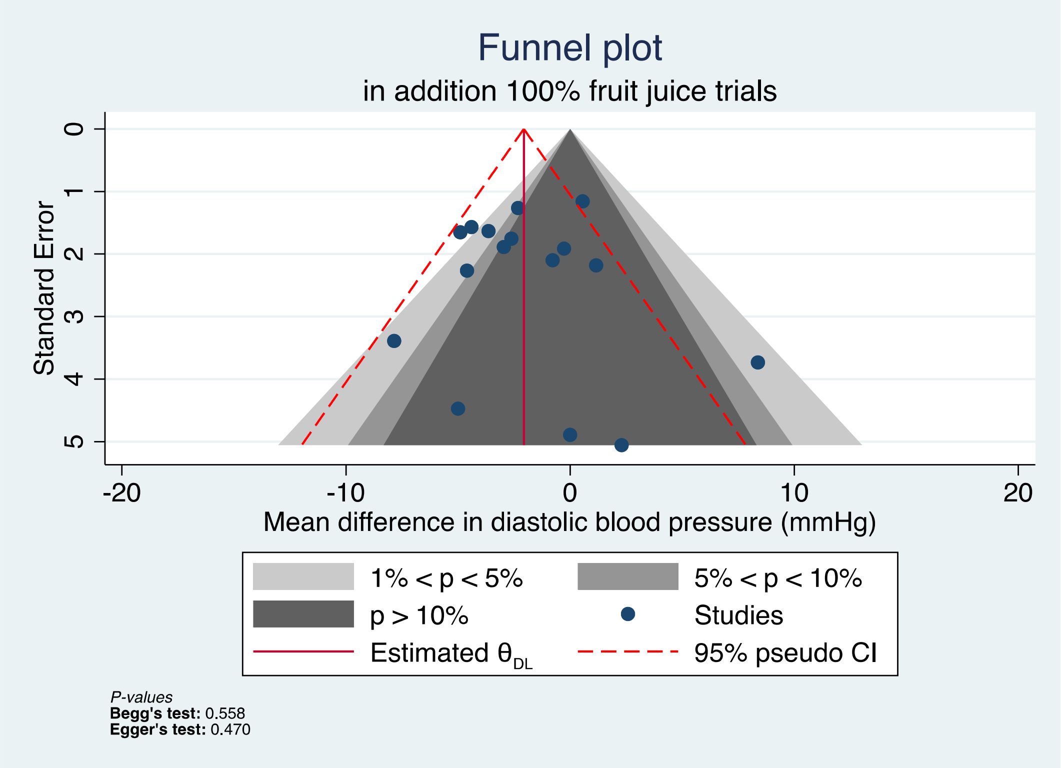

Contour-enhanced funnel plot is a scatterplot of each trial weighted mean difference on the x-axis with the standard error representing precision on the y-axis. The vertical solid red line represents the pooled effect estimate and the dashed red lines represent the pseudo-95% confidence limits. The blue dots represent individual trials. The contour regions define the regions for the test of significance of individual trial effect size for a given p-value range >0.100 (dark grey), 0.500 to <0.100 (medium grey), 0.010 to <0.500 (light grey), <0.0100 (white)]. The contour-enhanced funnel plots may suggest funnel-plot asymmetry is due to publication bias when less precise (smaller) trials are missing in the non-significant regions. Quantitative assessment of publication bias was also performed using Egger's and Begg's tests set at a significance level of p<0.100.
CI=confidence interval.

S144 Fig. Publication bias funnel plots for the effect of fruit on DBP (mmHg) in addition trials.

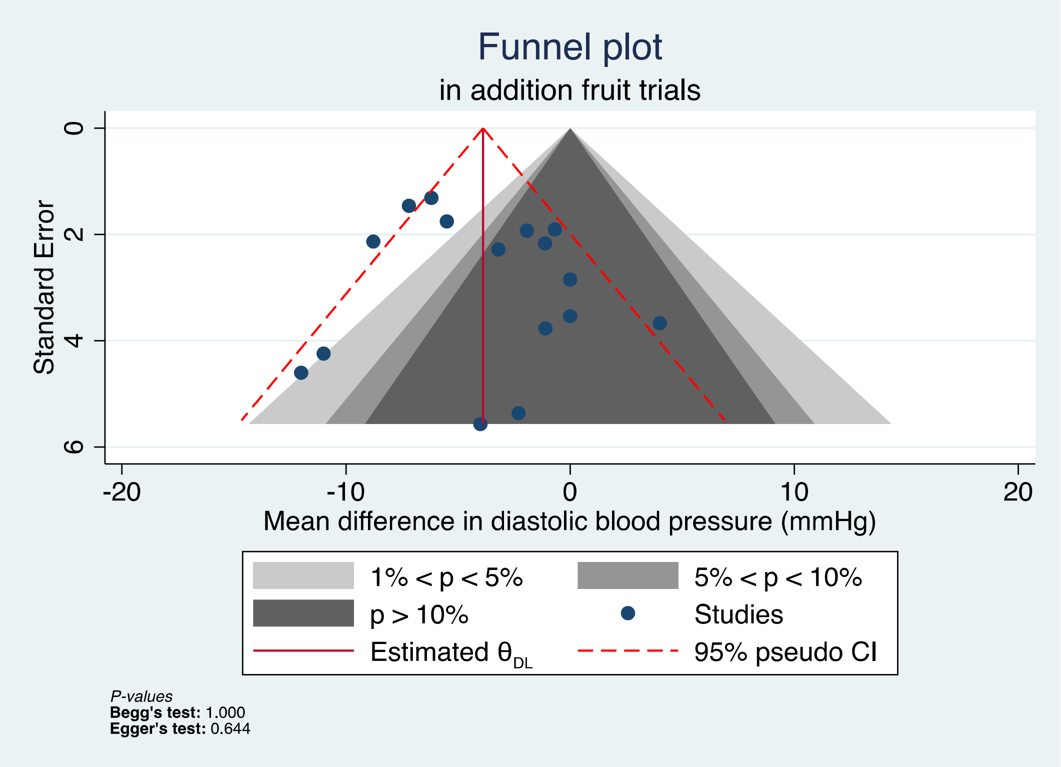

Contour-enhanced funnel plot is a scatterplot of each trial weighted mean difference on the x-axis with the standard error representing precision on the y-axis. The vertical solid red line represents the pooled effect estimate and the dashed red lines represent the pseudo-95% confidence limits. The blue dots represent individual trials. The contour regions define the regions for the test of significance of individual trial effect size for a given p-value range >0.100 (dark grey), 0.500 to <0.100 (medium grey), 0.010 to <0.500 (light grey), <0.0100 (white)]. The contour-enhanced funnel plots may suggest funnel-plot asymmetry is due to publication bias when less precise (smaller) trials are missing in the non-significant regions. Quantitative assessment of publication bias was also performed using Egger's and Begg's tests set at a significance level of p<0.100.
CI=confidence interval.

S145 Fig. Post-hoc subgroup analyses for the effect of important food sources of fructose-containing sugars on SBP (mmHg) in substitution trials.

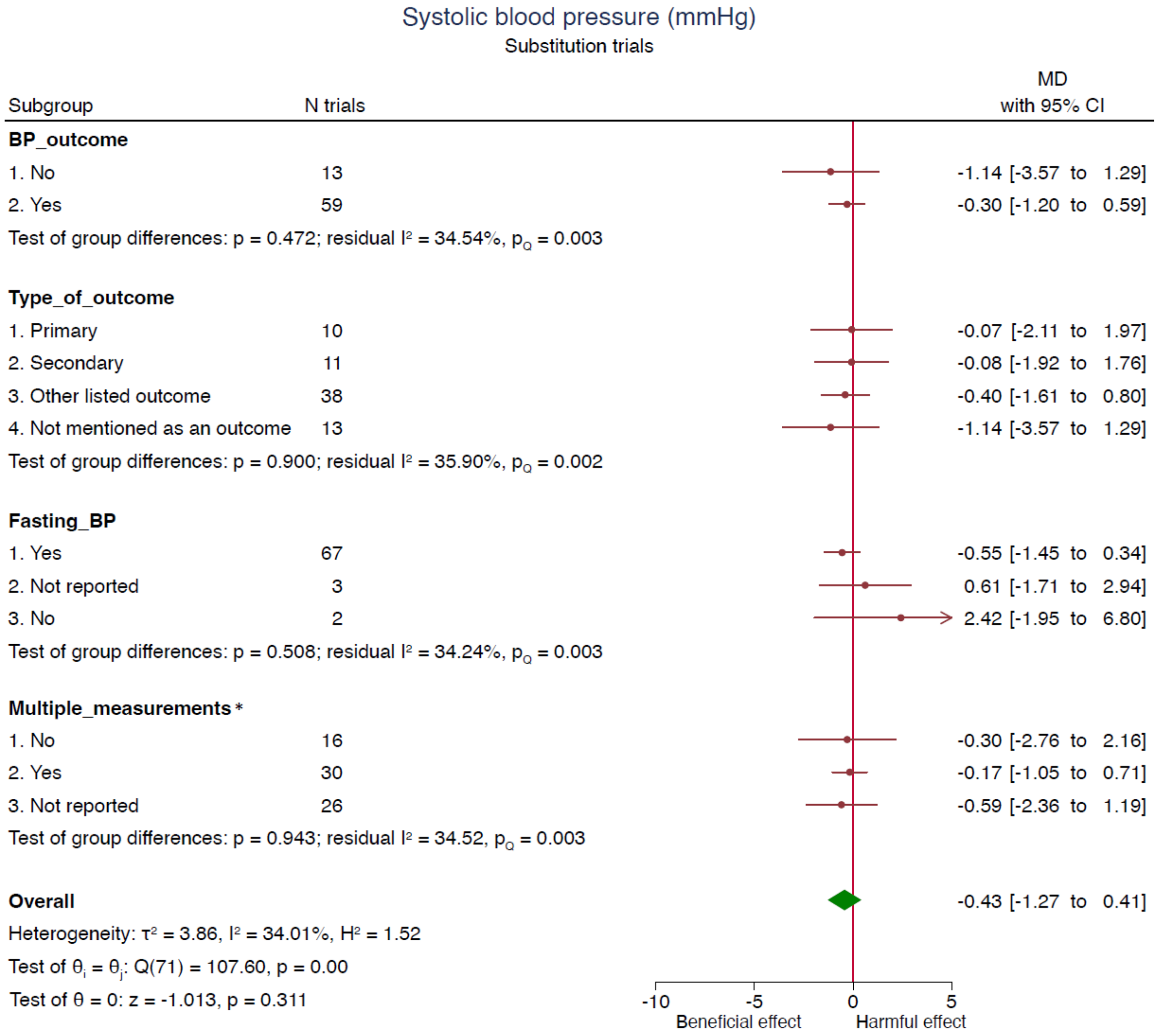

The green diamond represents the pooled estimate for the overall primary analysis of food sources of fructose-containing sugars on SBP. Within subgroup mean differences are the pooled effect estimates represented by a red circle. 95% confidence intervals are represented by the line through the circle. Data are expressed as mean differences with 95% confidence intervals using the generic inverse-variance method and random effects DerSimonian-Laird model. Inter-study heterogeneity was assessed using the Cochrane Q statistic and quantified using the I^2 statistic, with significance set at p<0.100 and $I^2 \geq 50\%$ considered to be evidence of substantial heterogeneity. p<0.050 indicates that the effect size differed between levels of the subgroup.
*fasted with multiple measurements taken
BP=blood pressure; CI=confidence interval; MD=mean difference

S146 Fig. Post-hoc subgroup analyses for the effect of important food sources of fructose-containing sugars on SBP (mmHg) in addition trials

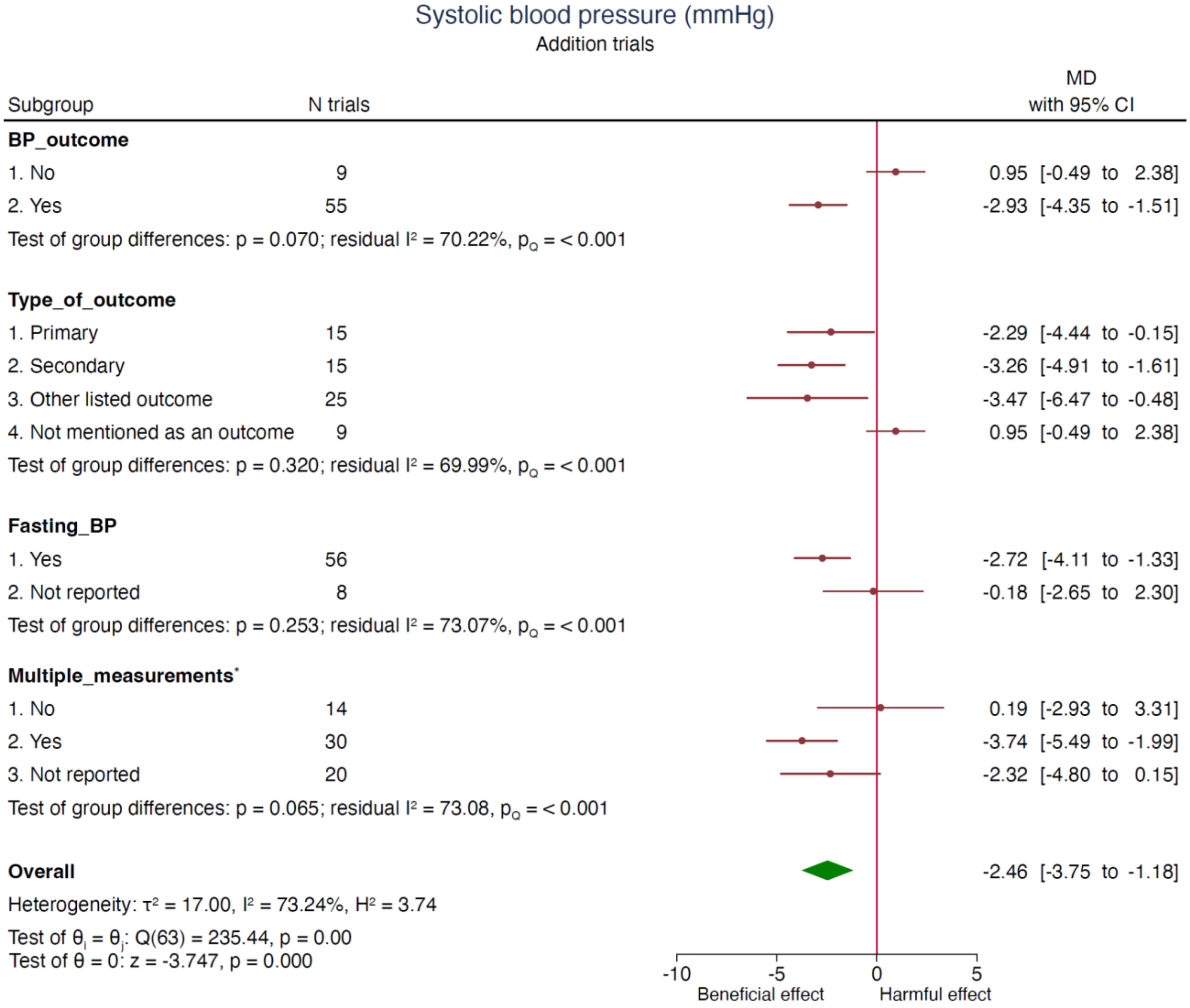

The green diamond represents the pooled estimate for the overall primary analysis of food sources of fructose-containing sugars on SBP. Within subgroup mean differences are the pooled effect estimates represented by a red circle. 95% confidence intervals are represented by the line through the circle. Data are expressed as mean differences with 95% confidence intervals using the generic inverse-variance method and random effects DerSimonian-Laird model. Inter-study heterogeneity was assessed using the Cochrane Q statistic and quantified using the I^2 statistic, with significance set at p<0.100 and I^2≥50% considered to be evidence of substantial heterogeneity. p<0.050 indicates that the effect size differed between levels of the subgroup.

*fasted with multiple measurements taken

BP=blood pressure; CI=confidence interval; MD=mean difference

S147 Fig. Post-hoc subgroup analyses for the effect of important food sources of fructose-containing sugars on SBP (mmHg) in subtraction trials

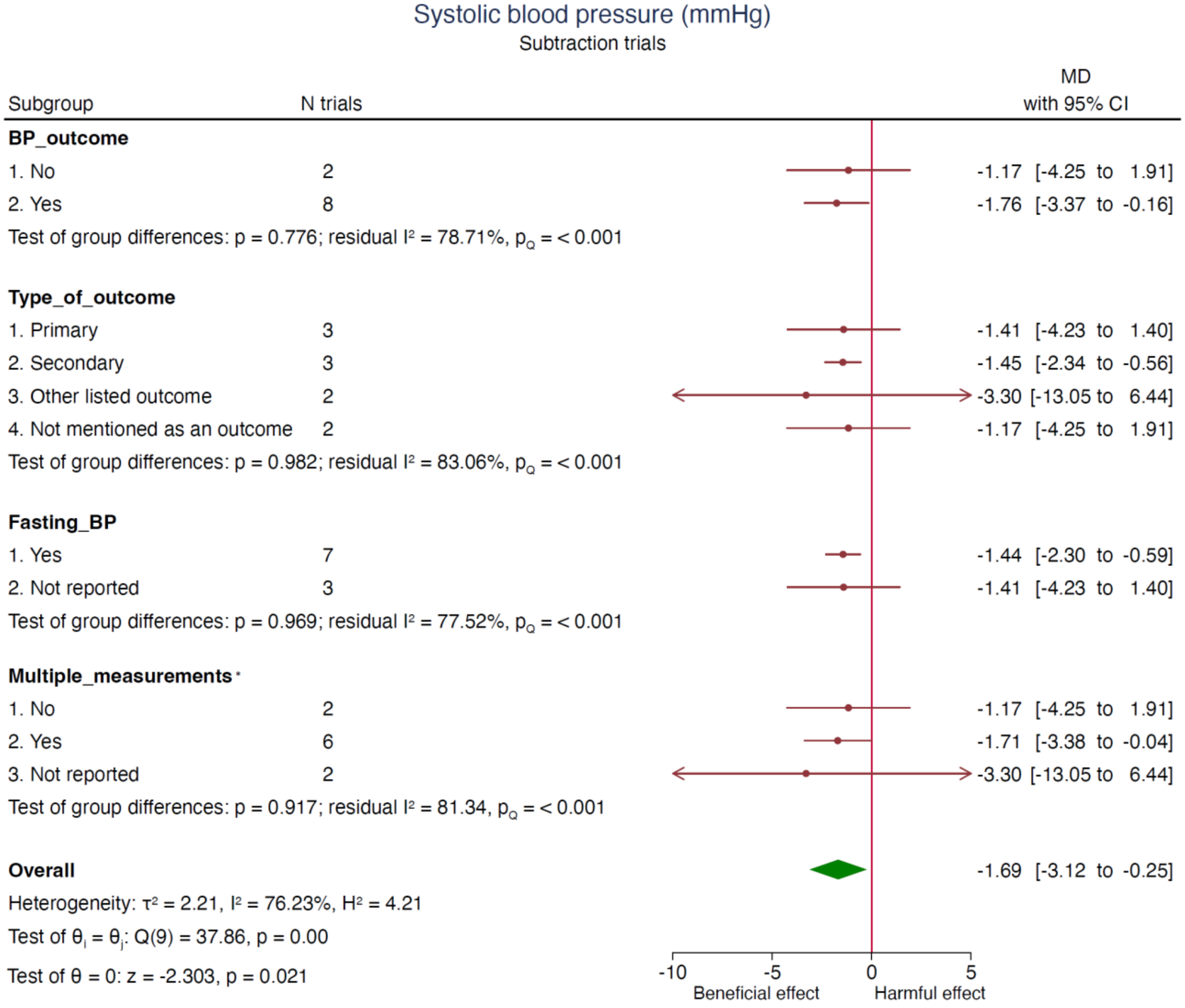

The green diamond represents the pooled estimate for the overall primary analysis of food sources of fructose-containing sugars on SBP. Within subgroup mean differences are the pooled effect estimates represented by a red circle. 95% confidence intervals are represented by the line through the circle. Data are expressed as mean differences with 95% confidence intervals using the generic inverse-variance method and random effects DerSimonian-Laird model. Inter-study heterogeneity was assessed using the Cochrane Q statistic and quantified using the I^2 statistic, with significance set at p<0.100 and $I^2 \geq 50\%$ considered to be evidence of substantial heterogeneity. p<0.050 indicates that the effect size differed between levels of the subgroup.

*fasted with multiple measurements taken

BP=blood pressure; CI=confidence interval; MD=mean difference

S148 Fig. Post-hoc subgroup analyses for the effect of important food sources of fructose-containing sugars on DBP (mmHg) in substitution trials.

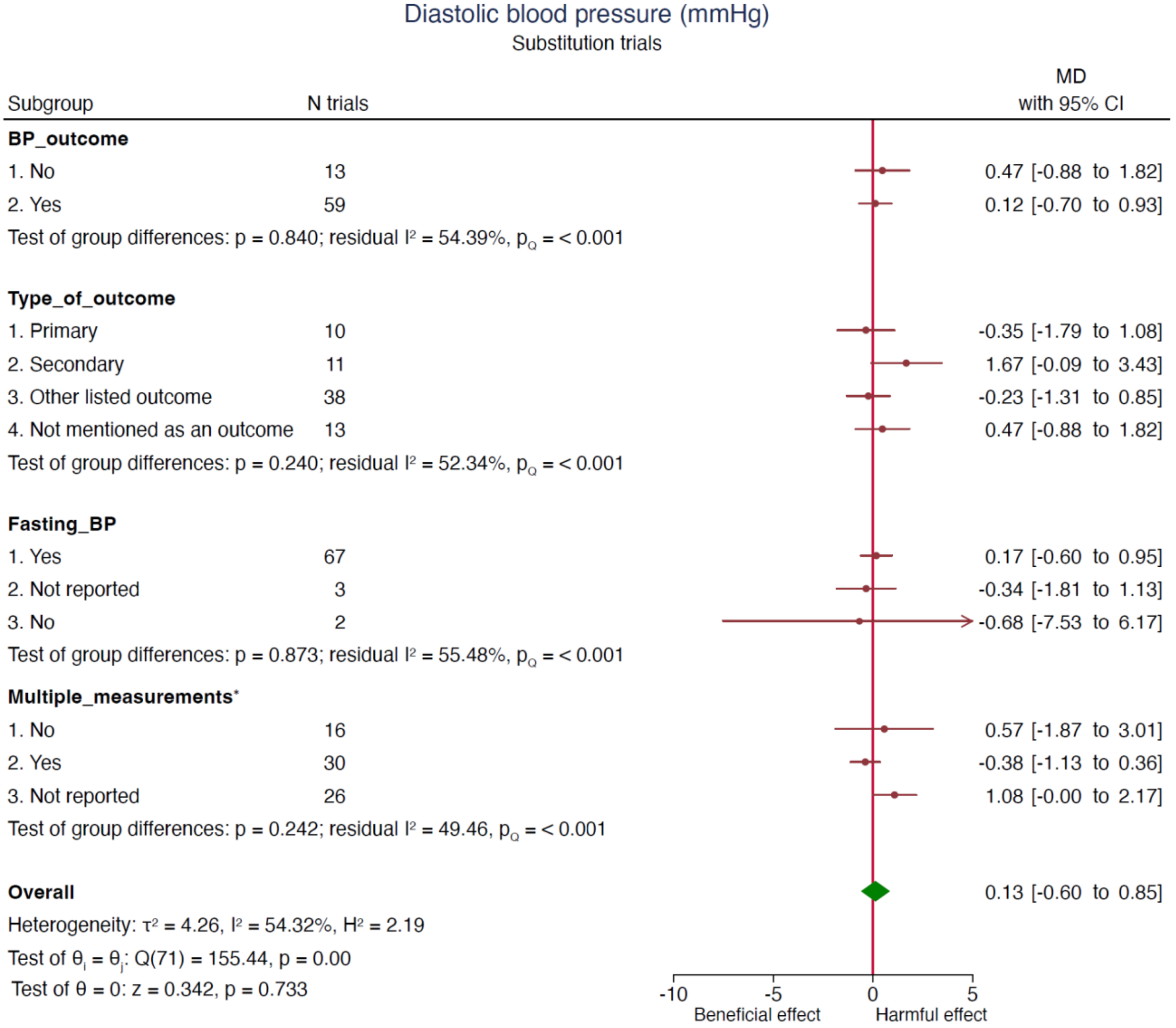

The green diamond represents the pooled estimate for the overall primary analysis of food sources of fructose-containing sugars on DBP. Within subgroup mean differences are the pooled effect estimates represented by a red circle. 95% confidence intervals are represented by the line through the circle. Data are expressed as mean differences with 95% confidence intervals using the generic inverse-variance method and random effects DerSimonian-Laird model. Inter-study heterogeneity was assessed using the Cochrane Q statistic and quantified using the I^2 statistic, with significance set at $p<0.100$ and $I^2 \geq 50\%$ considered to be evidence of substantial heterogeneity. $p<0.050$ indicates that the effect size differed between levels of the subgroup.

*fasted with multiple measurements taken

BP=blood pressure; CI=confidence interval; MD=mean difference

S149 Fig. Post-hoc subgroup analyses for the effect of important food sources of fructose-containing sugars on DBP (mmHg) in addition trials.

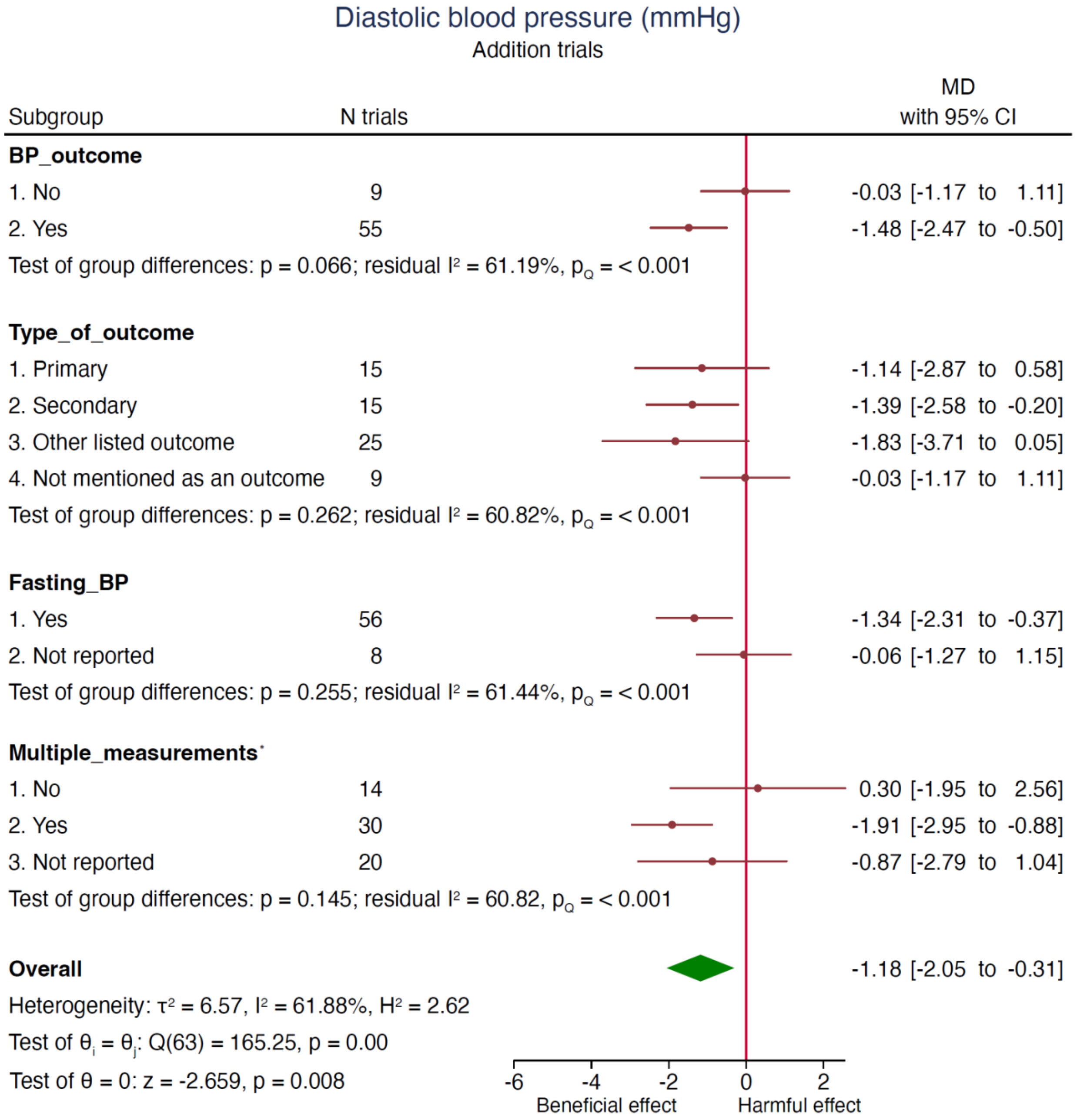

The green diamond represents the pooled estimate for the overall primary analysis of food sources of fructose-containing sugars on DBP. Within subgroup mean differences are the pooled effect estimates represented by a red circle. 95% confidence intervals are represented by the line through the circle. Data are expressed as mean differences with 95% confidence intervals using the generic inverse-variance method and random effects DerSimonian-Laird model. Inter-study heterogeneity was assessed using the Cochrane Q statistic and quantified using the I^2 statistic, with significance set at p<0.100 and $I^2 \geq 50\%$ considered to be evidence of substantial heterogeneity. p<0.050 indicates that the effect size differed between levels of the subgroup.

*fasted with multiple measurements taken

BP=blood pressure; CI=confidence interval; MD=mean difference

S150 Fig. Post-hoc subgroup analyses for the effect of important food sources of fructose-containing sugars on DBP (mmHg) in subtraction trials.

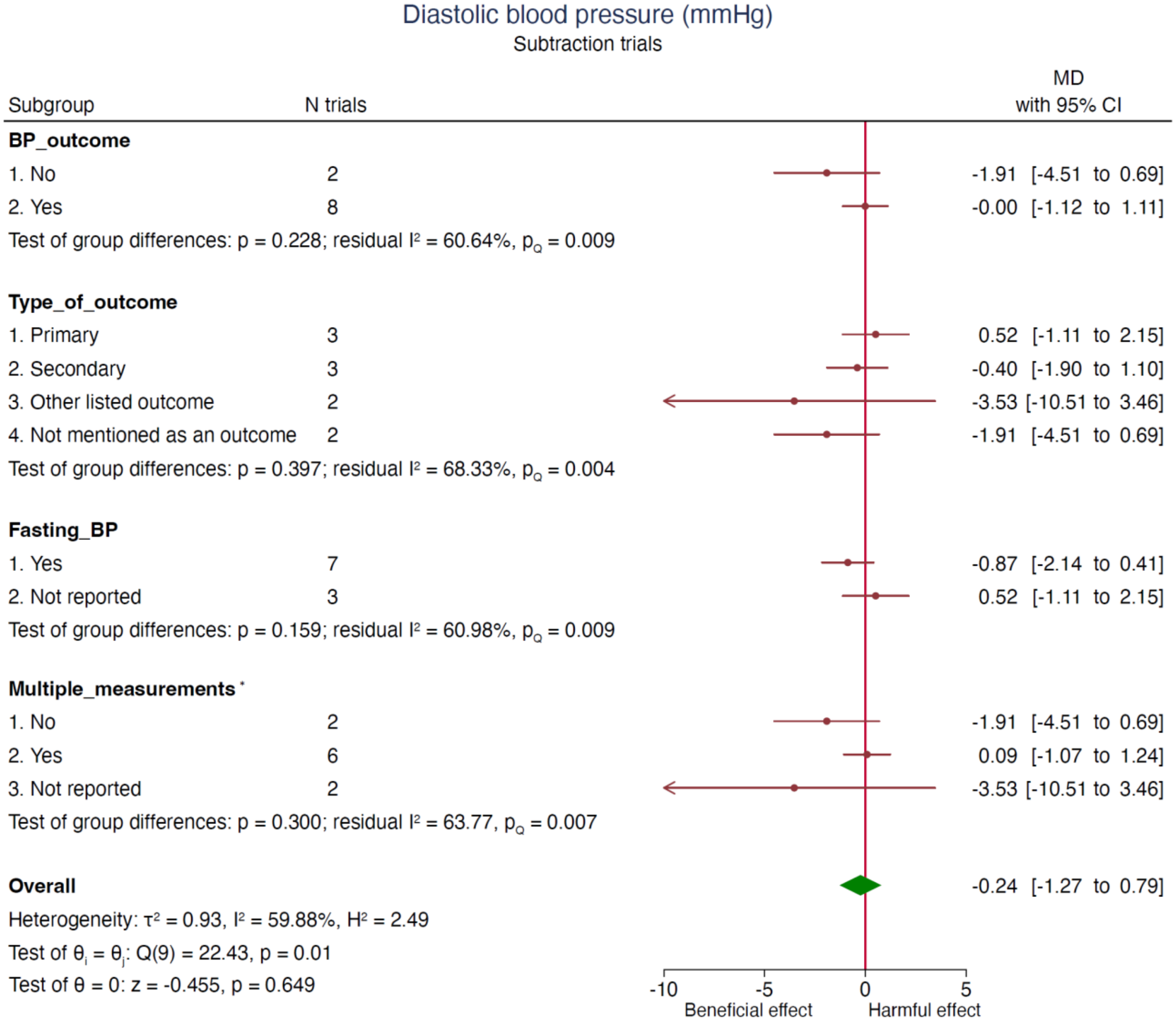

The green diamond represents the pooled estimate for the overall primary analysis of food sources of fructose-containing sugars on DBP. Within subgroup mean differences are the pooled effect estimates represented by a red circle. 95% confidence intervals are represented by the line through the circle. Data are expressed as mean differences with 95% confidence intervals using the generic inverse-variance method and random effects DerSimonian-Laird model. Inter-study heterogeneity was assessed using the Cochrane Q statistic and quantified using the I^2 statistic, with significance set at p<0.100 and I^2≥50% considered to be evidence of substantial heterogeneity. p<0.050 indicates that the effect size differed between levels of the subgroup.
*fasted with multiple measurements taken
BP=blood pressure; CI=confidence interval; MD=mean difference

S151 Fig. Post-hoc subgroup analyses for the effect of SSBs on SBP (mmHg) in substitution trials.

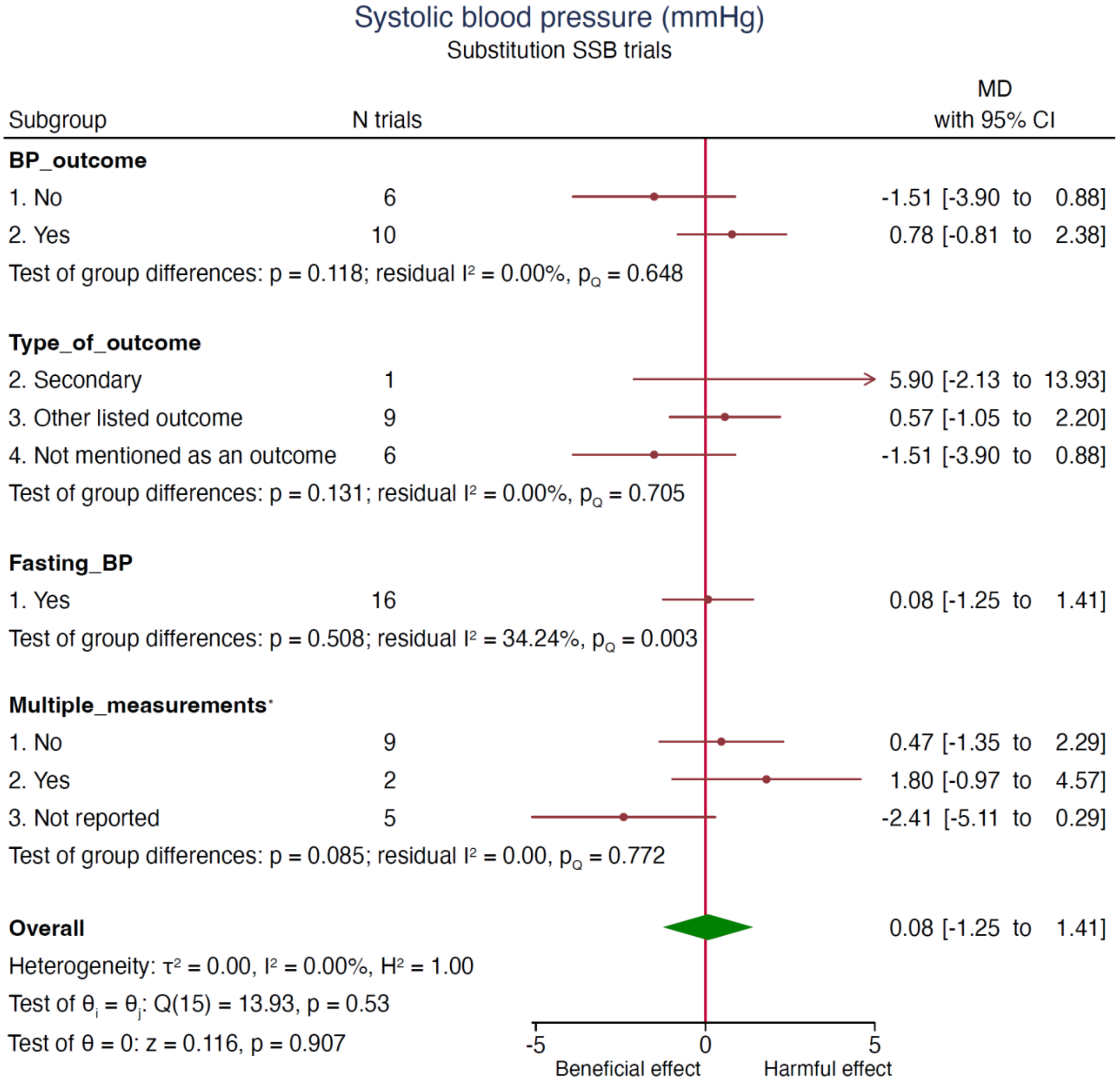

The green diamond represents the pooled estimate for the overall primary analysis of food sources of fructose-containing sugars on SBP. Within subgroup mean differences are the pooled effect estimates represented by a red circle. 95% confidence intervals are represented by the line through the circle. Data are expressed as mean differences with 95% confidence intervals using the generic inverse-variance method and random effects DerSimonian-Laird model. Inter-study heterogeneity was assessed using the Cochrane Q statistic and quantified using the I^2 statistic, with significance set at $p<0.100$ and $I^2 \geq 50\%$ considered to be evidence of substantial heterogeneity. $p<0.050$ indicates that the effect size differed between levels of the subgroup.

*fasted with multiple measurements taken

BP=blood pressure; CI=confidence interval; MD=mean difference, SSB=sugar sweetened beverages

S152 Fig. Post-hoc subgroup analyses for the effect of Fruit on SBP (mmHg) in substitution trials.

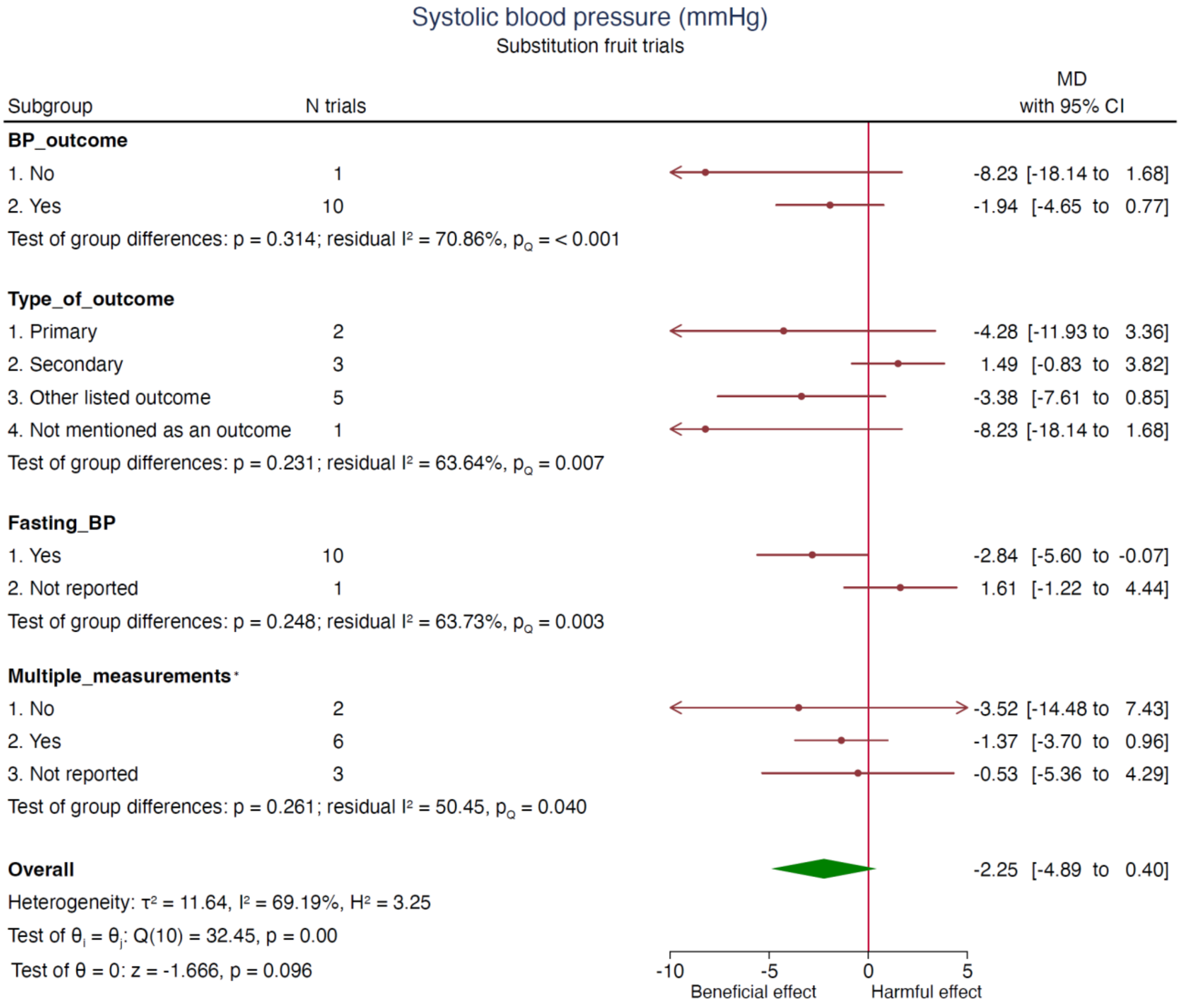

The green diamond represents the pooled estimate for the overall primary analysis of food sources of fructose-containing sugars on SBP. Within subgroup mean differences are the pooled effect estimates represented by a red circle. 95% confidence intervals are represented by the line through the circle. Data are expressed as mean differences with 95% confidence intervals using the generic inverse-variance method and random effects DerSimonian-Laird model. Inter-study heterogeneity was assessed using the Cochrane Q statistic and quantified using the I^2 statistic, with significance set at p<0.100 and I^2≥50% considered to be evidence of substantial heterogeneity. p<0.050 indicates that the effect size differed between levels of the subgroup.
*fasted with multiple measurements taken
BP=blood pressure; CI=confidence interval; MD=mean difference

S153 Fig. Post -hoc subgroup analyses for the effect of Mixed sources (with SSBs) on SBP (mmHg) in substitution trials.

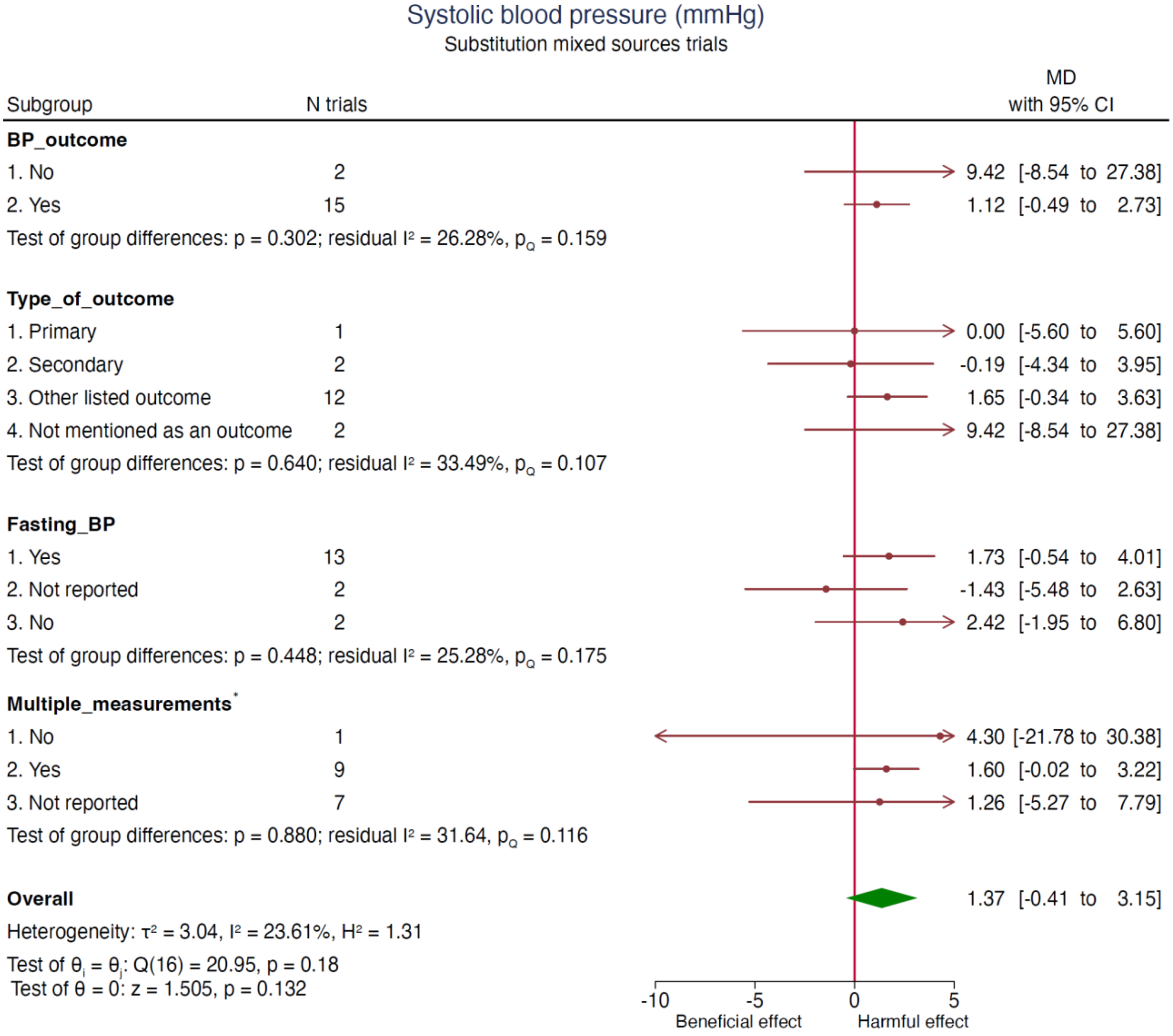

The green diamond represents the pooled estimate for the overall primary analysis of food sources of fructose-containing sugars on SBP. Within subgroup mean differences are the pooled effect estimates represented by a red circle. 95% confidence intervals are represented by the line through the circle. Data are expressed as mean differences with 95% confidence intervals using the generic inverse-variance method and random effects DerSimonian-Laird model. Inter-study heterogeneity was assessed using the Cochrane Q statistic and quantified using the I^2 statistic, with significance set at p<0.100 and $I^2 \geq 50\%$ considered to be evidence of substantial heterogeneity. p<0.050 indicates that the effect size differed between levels of the subgroup.

*fasted with multiple measurements taken

BP=blood pressure; CI=confidence interval; MD=mean difference, SSB=sugar sweetened beverages

S154 Fig. Post-hoc subgroup analyses for the effect of SSBs on SBP (mmHg) in addition trials.

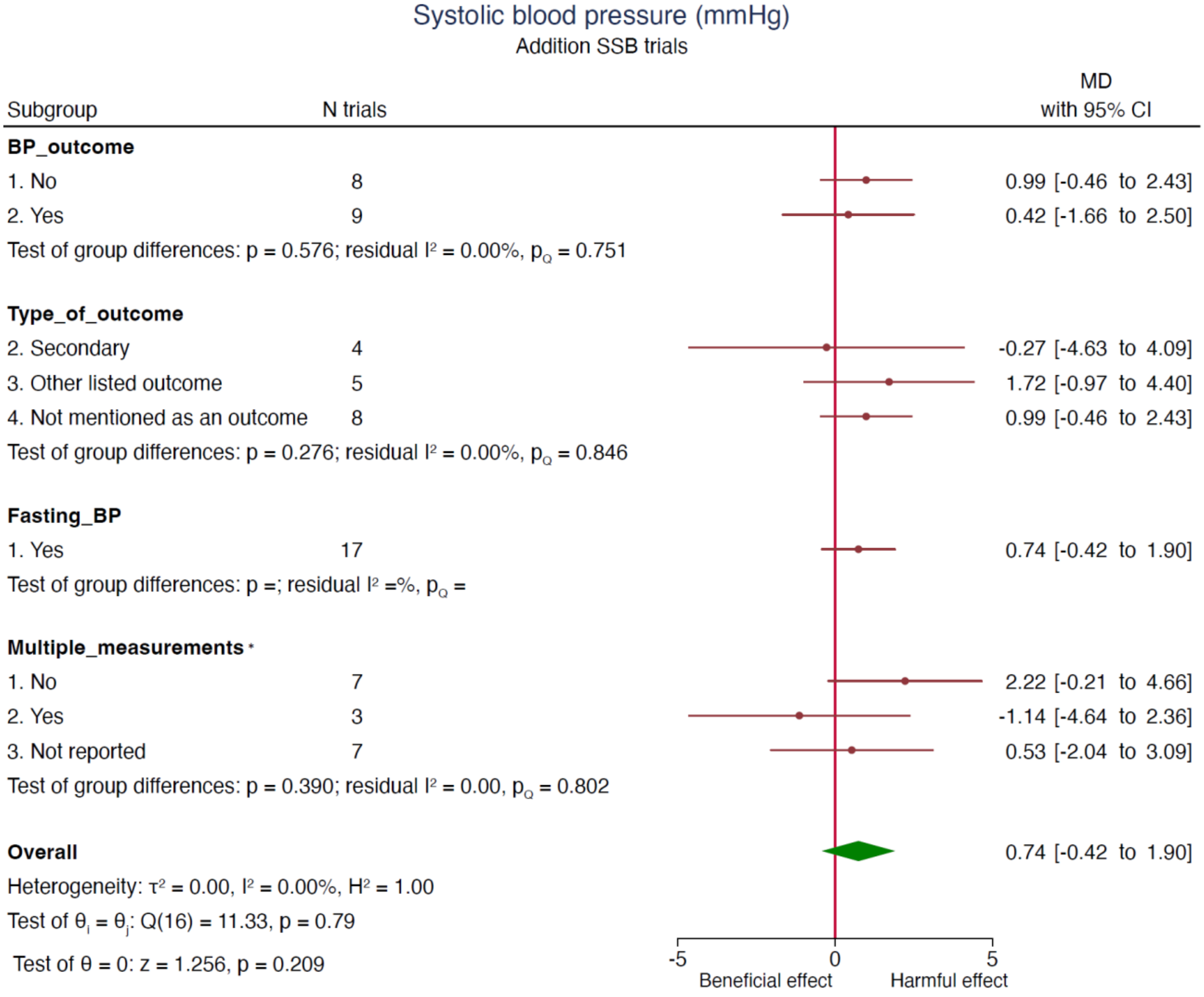

The green diamond represents the pooled estimate for the overall primary analysis of food sources of fructose-containing sugars on SBP. Within subgroup mean differences are the pooled effect estimates represented by a red circle. 95% confidence intervals are represented by the line through the circle. Data are expressed as mean differences with 95% confidence intervals using the generic inverse-variance method and random effects DerSimonian-Laird model. Inter-study heterogeneity was assessed using the Cochrane Q statistic and quantified using the I^2 statistic, with significance set at p<0.100 and $I^2 \geq 50\%$ considered to be evidence of substantial heterogeneity. p<0.050 indicates that the effect size differed between levels of the subgroup.

*fasted with multiple measurements taken

BP=blood pressure; CI=confidence interval; MD=mean difference, SSB=sugar sweetened beverages

S155 Fig. Post-hoc subgroup analyses for the effect of Fruit on SBP (mmHg) in addition trials.

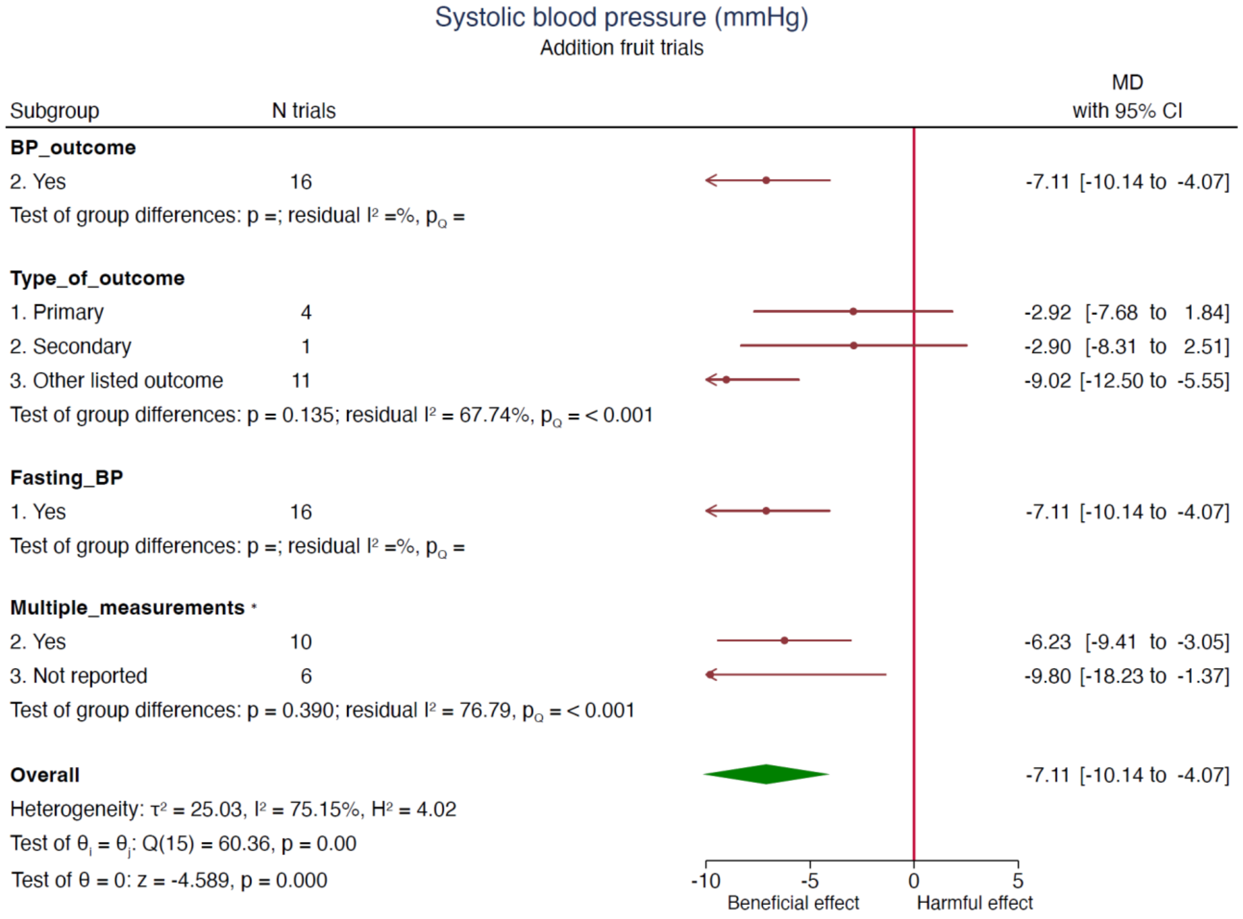

The green diamond represents the pooled estimate for the overall primary analysis of food sources of fructose-containing sugars on SBP. Within subgroup mean differences are the pooled effect estimates represented by a red circle. 95% confidence intervals are represented by the line through the circle. Data are expressed as mean differences with 95% confidence intervals using the generic inverse-variance method and random effects DerSimonian-Laird model. Inter-study heterogeneity was assessed using the Cochrane Q statistic and quantified using the I^2 statistic, with significance set at p<0.100 and I^2≥50% considered to be evidence of substantial heterogeneity. p<0.050 indicates that the effect size differed between levels of the subgroup.

*fasted with multiple measurements taken

BP=blood pressure; CI=confidence interval; MD=mean difference

S156 Fig. Post-hoc subgroup analyses for the effect of 100% Fruit Juice on SBP (mmHg) in addition trials.

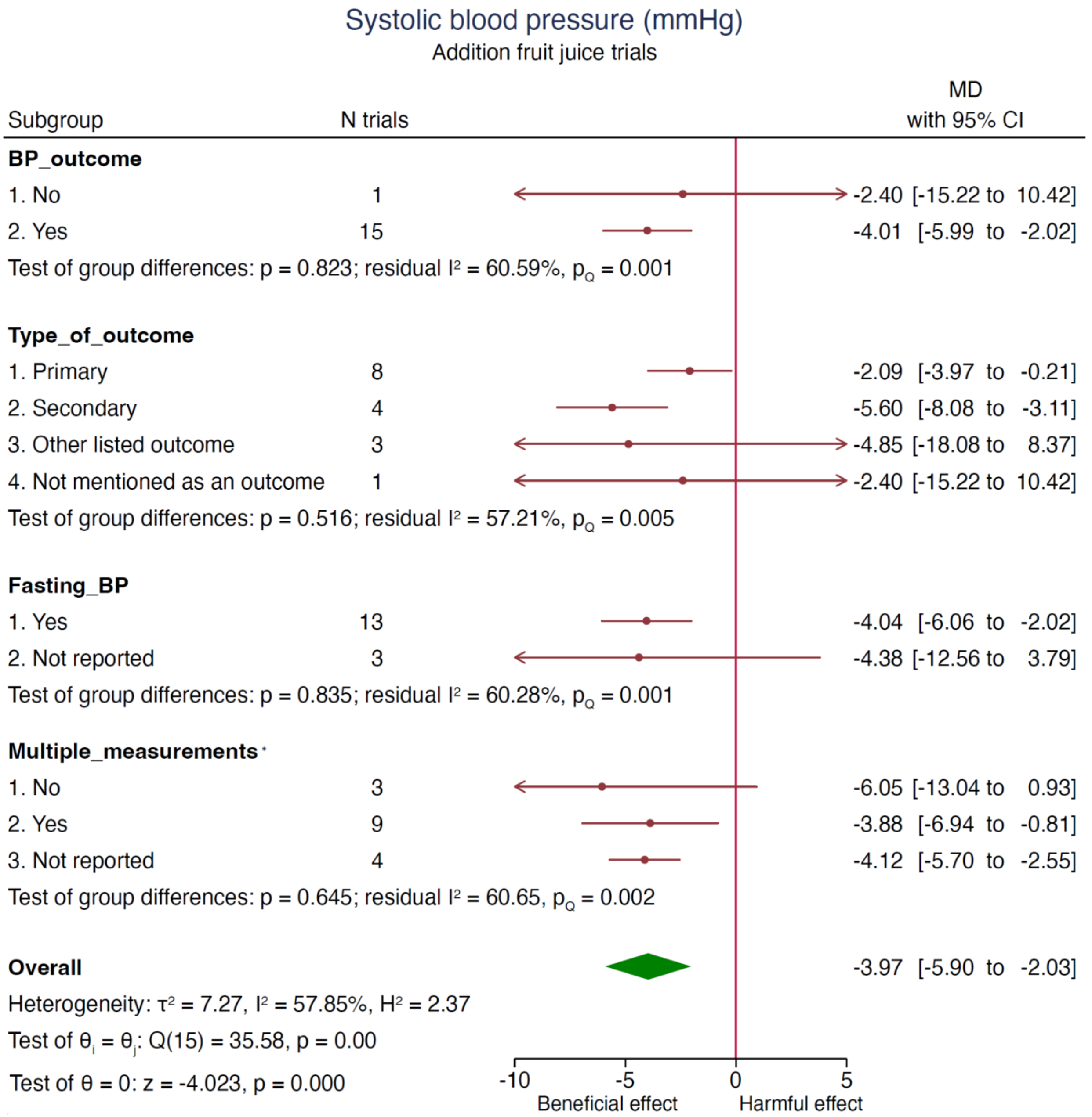

The green diamond represents the pooled estimate for the overall primary analysis of food sources of fructose-containing sugars on SBP. Within subgroup mean differences are the pooled effect estimates represented by a red circle. 95% confidence intervals are represented by the line through the circle. Data are expressed as mean differences with 95% confidence intervals using the generic inverse-variance method and random effects DerSimonian-Laird model. Inter-study heterogeneity was assessed using the Cochrane Q statistic and quantified using the I^2 statistic, with significance set at p<0.100 and $I^2 \geq 50\%$ considered to be evidence of substantial heterogeneity. p<0.050 indicates that the effect size differed between levels of the subgroup.

*fasted with multiple measurements taken

BP=blood pressure; CI=confidence interval; MD=mean difference

S157 Fig. Post-hoc subgroup analyses for the effect of SSBs on DBP (mmHg) in addition trials.

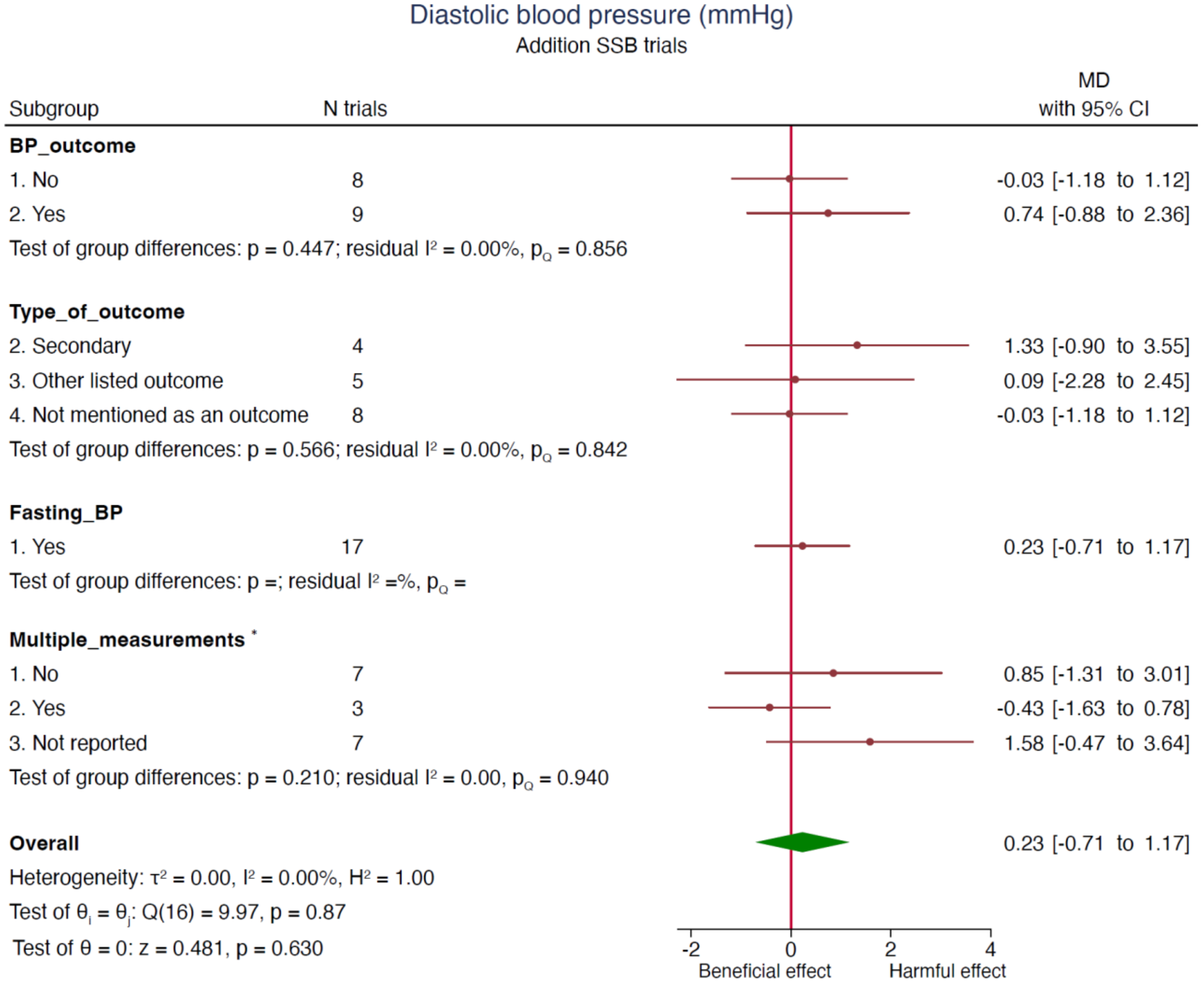

The green diamond represents the pooled estimate for the overall primary analysis of food sources of fructose-containing sugars on DBP. Within subgroup mean differences are the pooled effect estimates represented by a red circle. 95% confidence intervals are represented by the line through the circle. Data are expressed as mean differences with 95% confidence intervals using the generic inverse-variance method and random effects DerSimonian-Laird model. Inter-study heterogeneity was assessed using the Cochrane Q statistic and quantified using the I^2 statistic, with significance set at p<0.100 and $I^2 \geq 50\%$ considered to be evidence of substantial heterogeneity. p<0.050 indicates that the effect size differed between levels of the subgroup.
*fasted with multiple measurements taken
BP=blood pressure; CI=confidence interval; MD=mean difference, SSB=sugar sweetened beverages

S158 Fig. Post-hoc subgroup analyses for the effect of Fruit on DBP (mmHg) in addition trials.

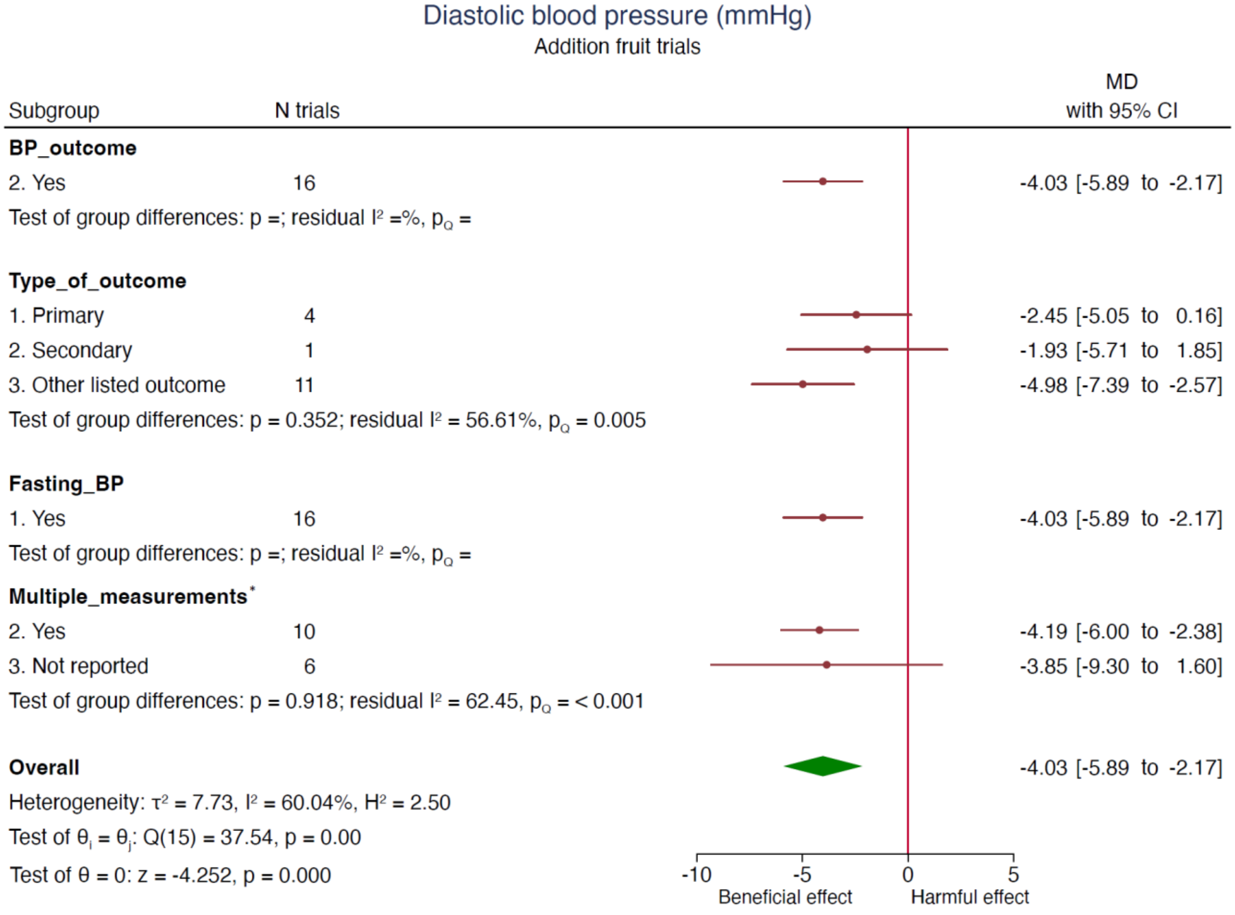

The green diamond represents the pooled estimate for the overall primary analysis of food sources of fructose-containing sugars on DBP. Within subgroup mean differences are the pooled effect estimates represented by a red circle. 95% confidence intervals are represented by the line through the circle. Data are expressed as mean differences with 95% confidence intervals using the generic inverse-variance method and random effects DerSimonian-Laird model. Inter-study heterogeneity was assessed using the Cochrane Q statistic and quantified using the I^2 statistic, with significance set at p<0.100 and $I^2 \geq 50\%$ considered to be evidence of substantial heterogeneity. p<0.050 indicates that the effect size differed between levels of the subgroup.

*fasted with multiple measurements taken

BP=blood pressure; CI=confidence interval; MD=mean difference

S159 Fig. Post-hoc subgroup analyses for the effect of 100% Fruit Juice on DBP (mmHg) in addition trials.

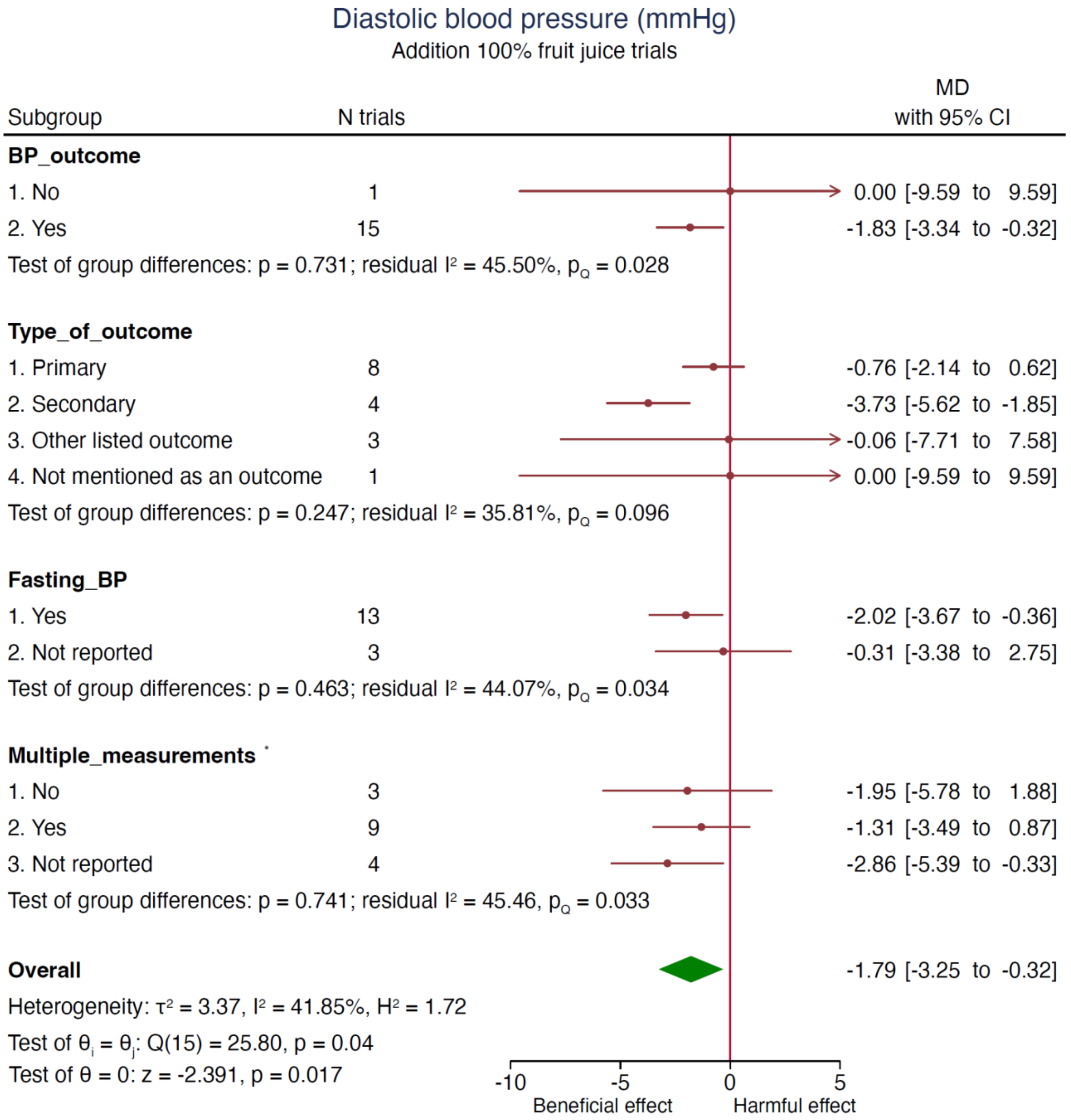

The green diamond represents the pooled estimate for the overall primary analysis of food sources of fructose-containing sugars on DBP. Within subgroup mean differences are the pooled effect estimates represented by a red circle. 95% confidence intervals are represented by the line through the circle. Data are expressed as mean differences with 95% confidence intervals using the generic inverse-variance method and random effects DerSimonian-Laird model. Inter-study heterogeneity was assessed using the Cochrane Q statistic and quantified using the I^2 statistic, with significance set at p<0.100 and $I^2 \geq 50\%$ considered to be evidence of substantial heterogeneity. p<0.050 indicates that the effect size differed between levels of the subgroup.

*fasted with multiple measurements taken

BP=blood pressure; CI=confidence interval; MD=mean difference
